# miR-150 Regulates Differentiation and Cytolytic Effector Function in CD8+ T cells

**DOI:** 10.1038/srep16399

**Published:** 2015-11-09

**Authors:** Norah L. Smith, Erin M. Wissink, Andrew Grimson, Brian D. Rudd

**Affiliations:** 1 Department of Microbiology and Immunology, Cornell University, Ithaca, NY 14853; 2 Department of Molecular Biology and Genetics, Cornell University, Ithaca, NY 14853

## Abstract

MicroRNAs regulate most mammalian genes, and they control numerous aspects of immune system development and function. Their precise roles in the CD8+ T cell response, however, remain unclear. In this report, we show that in the absence of the microRNA miR-150, CD8+ T cells fail to undergo robust expansion and differentiation into short-lived terminal effector cells in response to primary infection with *Listeria monocytogenes* or Vaccinia virus. Notably, even after transitioning into the memory pool, miR-150^−/−^ cells still mount a weaker recall response to secondary infection, and remain less differentiated than their wild-type counterparts. Transcriptome analysis shows miR-150 gene targets are globally upregulated in cells lacking miR-150, and amongst these targets, we found misregulation of genes associated with proliferation and effector cell function. These transcriptome data suggest that miR-150 deficient CD8+ T cells are less efficient in killing infected cells, which we validate experimentally. Together, these results reveal a cell-intrinsic role for miR-150 in the regulation of effector CD8+ T cell fate and function.

CD8+ T cells are essential in providing immune protection against intracellular pathogens. Following infection, a small number of naïve CD8+ T cells undergo massive clonal expansion to generate millions of effector CD8+ T cells, which provide immune protection by secreting cytokines such as IFNγ or producing cytolytic molecules to kill target cells. However, there is considerable phenotypic and functional heterogeneity in the effector CD8+ T cell pool, and individual cells exist along a spectrum of differentiation states^1^. A cell’s differentiation state can be elucidated by examining expression of KLRG1, CD27 and IL-7Rα/CD127, levels of which distinguish terminally differentiated effector cells (KLRG1^hi^, CD127^lo^, CD27^lo^) from those that are less differentiated (KLRG1^lo^, CD127^hi^, CD27^hi^)^2^,^3^. Importantly, our understanding of the cell intrinsic factors driving effector CD8+ T cell differentiation remains incomplete.

Historically, the intrinsic factors receiving most attention have been proteins involved in transcription and signal transduction. More recently, it has become clear that members of a class of small regulatory RNAs, the microRNAs (miRNAs), are also important^4^. In the absence of the miRNA biogenesis enzyme Dicer, and thus essentially all miRNAs, CD8+ T cells are unable to develop^5^. If Dicer is deleted after thymic selection, CD8+ T cells are generated but fail to respond to infection^6^. These data implicate one or more miRNAs as key regulators of CD8+ T cell fate. MiRNAs function as negative regulators of gene expression, predominantly acting to accelerate decay of their mRNA targets^7^,^8^. More than half of mammalian genes contain evolutionarily conserved miRNA target sites within their 3′UTRs^8^, implying that most gene regulatory pathways incorporate miRNA-mediated regulation. While it is clear that miRNAs are required for the activation and differentiation of CD8+ T cells during infection^9^, the challenges that remain are to identify which specific miRNAs are critically involved, and to determine how specific miRNAs mediate their effects.

In this study, we profiled miRNAs in naïve CD8+ T cells from TCR transgenic mice and found that miR-150 was the most abundantly represented miRNA. While miR-150 has been implicated in the development and function of B cells^10^, NK cells^11^ and iNKT cells^11^,^12^, its role in the CD8+ T response to infection remains unclear. To address this knowledge gap, we transferred equal numbers of wild-type and miR-150^−/−^ CD8+ T cells into congenic mice and compared their ability to respond to acute and chronic pathogens. Collectively, these studies show that miR-150 is required for pathogen-induced CD8+ T cell differentiation.

## Results

### miR-150 is a cell-intrinsic factor required for robust effector CD8+ T cell proliferation and differentiation

**Figure 1:**
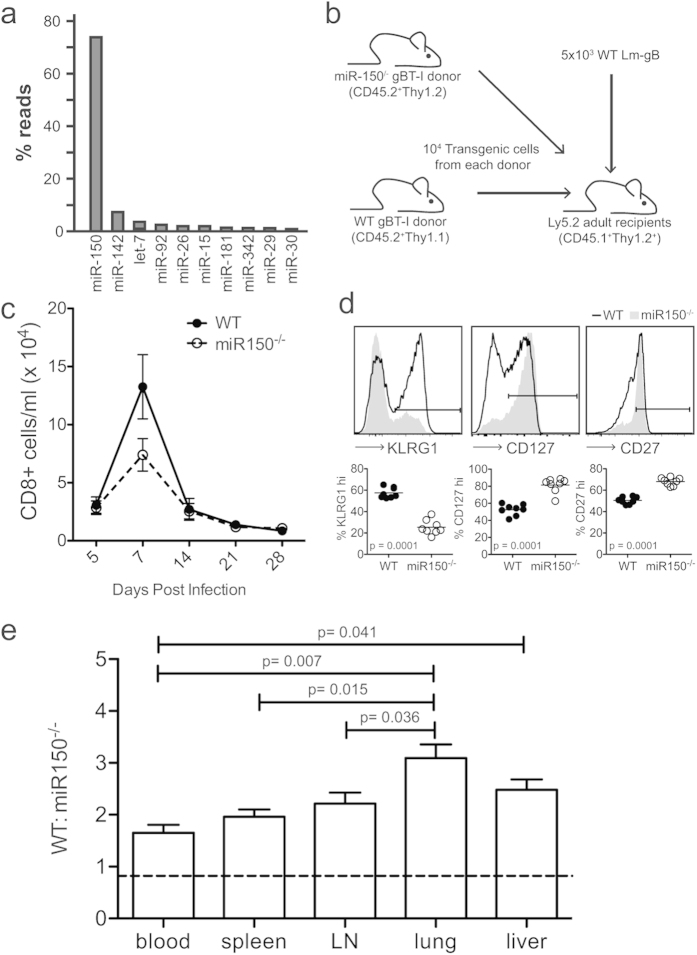
Prominently expressed miR-150 affects effector CD8+ T cell fate. (**a**) Relative amounts of the ten most abundant miRNAs in WT gBT-I cells, as determined by small RNA sequencing. (**b**) Schematic of dual adoptive transfer protocol. gBT-I cells were isolated from the spleens of congenically marked WT and miR-150^−/−^ mice and transferred into congenic recipients, who were subsequently infected with 5 × 10^3^ CFU *Lm*-gB. (**c**) CD8+ T cells in the blood of WT/miR150^−/−^ co-transfer recipients during time course of primary *Lm*-gB infection. (**d**) Flow cytometric histograms and (*bottom*) statistical evaluation of KLRG1, CD127 and CD27 at 7 dpi. Data representative of two experiments, *n* = 8–12. Statistics: paired Student *t* test. (**e**) Tissue distribution of donor gBT-I cells in lymphoid and non-lymphoid tissues expressed as a ratio of WT to miR-150^−/−^ cells 7 dpi. Dashed line indicates input ratio of donor cells. Data from two experiments, *n* = 14. Statistics: one-way ANOVA with Boniferroni’s multiple comparisons post-test.

To identify specific miRNAs that regulate CD8+ T cell functions, we isolated CD8+ T cells from naïve gBT-I TCR transgenic mice (cells specific for HSV1 Kb-restricted epitope gB_498-505_) and profiled genome-wide miRNAs using small RNA sequencing. We focused our analysis on the ten most highly expressed miRNAs, which each comprised at least 1% of the miRNA-matching sequences. Strikingly, we found that miR-150 made up >70% of miRNA-matching reads (Fig. 1a), making it the most highly represented miRNA in these cells.

To determine the biological function of miR-150 in CD8+ T cells, we generated gBT-I miR-150 knockout mice (miR-150^−/−^). These mice were then used as a source of donor cells for adoptive transfer experiments, which allowed us to focus specifically on cell-intrinsic differences in CD8+ T cells. We co-transferred equal numbers of WT and miR-150^−/−^ cells into congenic mice and infected the recipients with recombinant *Listeria monocytogenes* expressing the cognate peptide (SSIEFARL) for the gBT-I TCR (*Lm*-gB) (Fig. 1b). This strategy allowed us to directly compare CD8+ T cells lacking miR-150 to WT cells, under conditions with matched amounts of antigen and inflammation. Both populations of donor cells were tracked longitudinally in the blood at various times post-infection. While comparable numbers of miR-150^−/−^ and WT cells were observed early after infection (5 dpi), significantly more WT cells were present at the peak of the primary response (7 dpi) (Fig. 1c). This data suggest that miR-150 is required for the host to mount a robust CD8+ T cell response after infection.

An important control for these experiments is to ensure that colonization of miR-150 and WT cells in recipient mice is comparable. To test this, equal numbers of miR-150^−/−^ and WT cells were co-transferred into uninfected recipient mice and the percentage of WT to miR-150^−/−^ cells in the lymph node, spleen and blood were determined the next day. As shown in supplemental Figure S1, the percentages of WT to miR-150^−/−^ cells in various tissues were similar to the input percentage. These data indicate that the seeding efficiency of miR-150^−/−^ and WT cells is similar.

We suspected that fewer miR-150^−/−^ cells accumulate after infection because of differences in proliferation, survival, or both. To differentiate between these underlying mechanisms, we first assessed proliferation by pulsing recipient mice with BrdU on 6 dpi and measuring incorporation in the donor cells at 7 dpi. As shown in supplemental Figure S2, approximately one-third fewer miR-150^−/−^ effector CD8+ T cells were actively proliferating during this time. We next examined the percentage of donor cells undergoing apoptosis and found similar amounts of annexin V labeling on WT and miR150^−/−^ cells (supplemental Fig. S2). These results indicate that fewer miR-150^−/−^ cells accumulate because they exhibit reduced proliferation during infection, not because of differences in cell death.

Given that WT cells undergo more sustained cell proliferation than miR-150^−/−^ cells, we next asked whether they exhibit a different phenotype at the peak of the response. Effector cells that are more terminally differentiated express higher levels of KLRG1 but lower amounts of CD27 and CD127^2^,^3^. When we examined expression of these markers on donor CD8+ T cells in the spleen at 7 dpi, we observed striking differences between WT and miR-150^−/−^ cells. The WT cells were comprised of significantly more terminally differentiated effectors (KLRG1+, CD127-, CD27-), whereas the population of miR150^−/−^ effector cells was far less differentiated (Fig. 1d). Together, these results demonstrate a cell-intrinsic requirement for miR-150 in the expansion and differentiation of primary CD8+ T cells after infection.

The altered levels of proliferation and differentiation observed in miR-150^−/−^ cells raised the possibility that these cells might be preferentially recruited to peripheral sites of infection. To examine potential differences in cell migration, we assessed the distribution of donor cells in various peripheral organs at the peak of the response. Donor cells were isolated from lymphoid (spleen, lymph node and blood) and non-lymphoid (lung and liver) tissues from co-transfer recipients on 7 dpi. Interestingly, the same 2–3 fold enrichment of WT cells was maintained in all tissues, although this difference was particularly pronounced in the lung (Fig. 1e). Moreover, the less differentiated phenotype of miR-150^−/−^ cells was observed in all tissues (supplemental Figure S3). Collectively, these data indicate that miR-150^−/−^ cells have impaired proliferative potential irrespective of their tissue location.

### miR-150 regulates effector CD8 T cell fate independent of naïve phenotype

**Figure 2:**
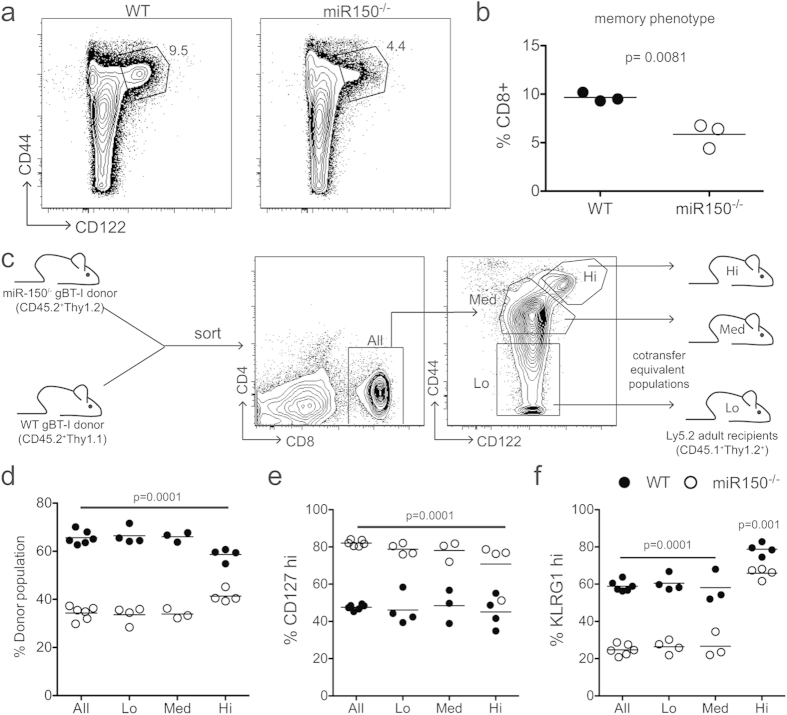
miR-150 acts on effector cells independently of naïve phenotype (**a**) Contour plots of CD44 and CD122 expression in naïve WT and miR-150^−/−^ splenocytes. Gated population represents naïve memory phenotype cells (CD44^hi^CD122^hi^), numbers displayed represent percentage of CD8+ cells with that phenotype (**b**) Statistical evaluation of memory phenotype as identified in (**a**). Data representative of two experiments, *n* = 3–4. Statistics: paired Student *t* test. (**c**) Schematic for dual adoptive transfer of equivalent populations. gBT-I cells were isolated from the spleens of congenically marked WT and miR-150^−/−^ mice, and sorted for low, medum or hi expression of CD44. Equal numbers of cells (10^4^) from like populations from WT and miR-150^−/−^ mice were co-transferred into congenic recipients who where then infected with 5 × 10^3^ CFU *Lm*-gB, (**d**–**f**) Relative percentages of donor, CD127^hi^ and KLRG1^hi^ from groups indicated in (**c**) on 7 dpi. Data from two experiments, *n* = 4. Statistics: two-way ANOVA with Boniferroni’s multiple comparisons post-test.

We next investigated whether the altered behavior of miR-150^−/−^ and WT cells during infection is linked to phenotypic differences present prior to infection. Although CD8+ T cells from uninfected gBT-I mice have never encountered their cognate peptide, approximately 10–20% of the starting population exhibits a memory phenotype (CD44^hi^ CD122^hi^). This is biologically significant because the subset of CD8+ CD44^hi^ CD122^hi^ memory phenotype cells responds more quickly to stimulation than the CD8+ CD44^lo^ CD122^lo^ naïve phenotype cells^13^. Therefore, based upon their expression of CD44 and CD122, we characterized the number of memory phenotype cells in uninfected WT and miR-150^−/−^ cells. Although we recovered equivalent numbers of CD8+ T cells from the spleens of WT and miR-150^−/−^ gBT-I mice, mice lacking miR-150 were comprised of significantly fewer memory phenotype cells (Fig. 2a,b).

These phenotypic differences raised the possibility that WT cells undergo more robust effector cell differentiation than miR-150^−/−^ cells because they have greater numbers of memory phenotype cells prior to infection. To test this, we set up a dual transfer experiment where we co-transferred equal numbers of WT and miR-150^−/−^ cells with the same specific starting phenotypes: CD44 CD44^lo^CD122^lo^ (Lo), CD44^int^CD122^lo^ (Med) or CD44^hi^CD122^hi^ (Hi) (Fig. 2c). Recipient mice were then infected with *Lm-gB*, and the number and phenotype of donor cells were evaluated at the peak of the response. Importantly, even when equivalent starting populations were compared directly to each other, miR-150^−/−^ cells still behaved differently than their WT counterparts. That is, in all naïve subsets examined (Lo, Med and Hi), miR-150^−/−^ cells made up a smaller proportion of the donor population (Fig. 2d), and expressed significantly more CD127 (Fig. 2e) and significantly less KLRG1 (Fig. 2f). Notably, memory phenotype cells gave rise to larger numbers of KLRG1+ effector cells compared to naïve phenotype cells; however, the memory phenotype cells from WT mice still become more terminally differentiated than those from miR-150^−/−^ mice. These results indicate that miR-150 is critical for effector differentiation, regardless of initial (naïve or memory) phenotype.

To determine if naive miR-150^−/−^ cells exhibit any other obvious phenotypic differences from WT cells, we also compared expression levels of markers associated with cell differentiation (CD127, CD62L, CD69), homeostatic proliferation (CD49d, CD11a, Ly6C) and TCR expression (Vb8, TCRβ, CD3) (supplemental Figure S4). The WT and miR-150^−/−^ cells expressed comparable amounts of all tested markers, suggesting that differences in cell differentiation between WT and miR-150^−/−^ cells only arise after microbial challenge and are not linked to the amount of TCR expressed on the surface of cells prior to infection, nor to other differences within naïve cells.

### miR-150 regulates effector CD8+ T cell fate under diverse infection conditions

**Figure 3:**
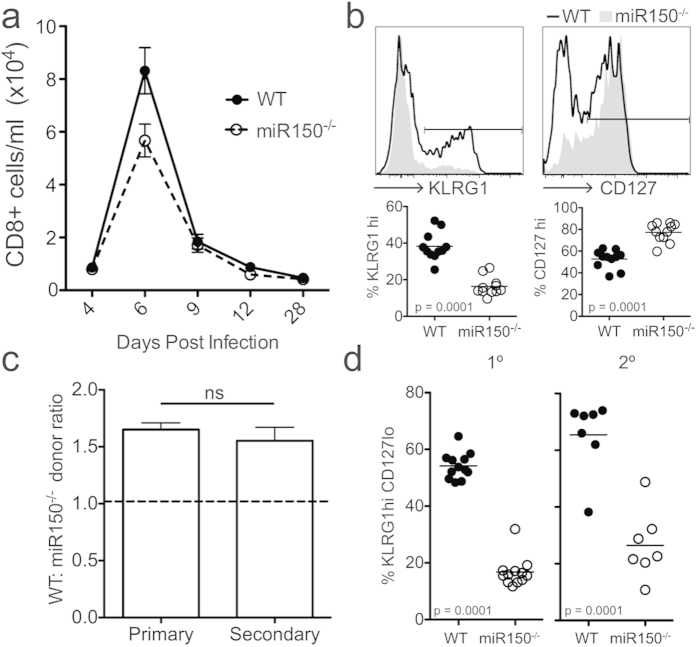
miR-150 regulates CD8+ T cell effector fate under diverse infection conditions. (**a**) Donor CD8+ T cells in the blood of WT/miR150^−/−^ co-transfer recipients during time course of primary VACV-gB infection. (**b**) Flow cytometric histograms (*top*) and statistical evaluation (*bottom*) of KLRG1 and CD127 expression at 6 dpi. (**c**) Ratio of WT:miR150^−/−^ T cells at the peak primary (7 dpi) and secondary response (5 dpi); dashed line indicates starting ratio. (**d**) Percentage of indicated donor cells with a terminally differentiated phenotype (KLRG1^hi^ CD127^lo^) at the peak primary and secondary responses to *Lm*-gB. Data representative of 2 experiments, *n* = 8–12. Statistics: paired Student *t* test.

All of our studies up to this point were performed in the context of bacterial infection. This is notable since effector and memory cell differentiation is influenced by pathogen-specific milieu^14^. To determine whether impaired effector cell differentiation is unique to bacterial infections, we also examined the ability of WT and miR-150^−/−^ cells to respond to a recombinant vaccinia virus that expresses the gB peptide (VACV-gB). Compared to *LM-*gB infections, VACV-gB drives a more diminished CD8+ T cell response, comprised of fewer KLRG1+ effector cells. However, the differences in effector development between WT and miR-150 cells that were observed with *LM-gB* were largely recapitulated in the context of VACV-gB: miR-150^−/−^ cells again failed to undergo robust expansion and exhibited a less differentiated phenotype compared to WT cells (Fig. 3a,b). Therefore, miR-150 regulates effector CD8+ T cell differentiation in a pathogen independent manner.

### miR-150 is required for generating memory CD8+ T cell responses

At the peak of the primary CD8+ T cells response, more terminally differentiated effector cells tend to be short-lived and fail to persist in the memory pool. Thus, we considered whether the phenotypic skewing of miR-150^−/−^ cells towards less differentiated effector cells after primary infection (Fig. 1d) might favor their later expansion during secondary infection. To test this, we co-transferred WT and miR-150^−/−^ cells into congenic recipients, as before. The next day, we infected recipient mice with Lm-gB and allowed them to transition to the memory pool. At 28 days post-infection, we re-challenged the same recipient mice with *Lm-gB,* and found that memory miR-150^−/−^ cells still expanded less than their WT counterparts (Fig. 3c). We also examined the phenotype of memory CD8+ T cells at the peak of the recall response. The majority (~70%) of WT cells exhibited a terminally differentiated phenotype, whereas only a small percentage (~30%) of memory miR-150^−/−^ cells were skewed towards this subset (Fig. 3d). This data demonstrates that miR-150 is required for both primary and memory CD8+ T cell responses.

### Loss of miR-150 alters the transcriptional program of effector CD8+ T cells

To gain insight into how miR-150 alters the fate of CD8+ T cells after infection, we sorted donor CD8+ cells from dual adoptive transfer recipients at 7dpi with *Lm-*gB and profiled their transcriptomes using RNA-Seq and small RNA sequencing. Using small RNA sequencing, we confirmed the deletion of miR-150 and observed that expression levels of other miRNAs correlated strongly and significantly when compared between WT and miR-150^−/−^ samples (Pearson r = 0.89; p < 2.2 × 10^−16^; data not shown). These data strongly suggest that phenotypes observed in miR-150^−/−^ CD8+ T cells relate directly to the loss of this miRNA alone.

**Figure 4:**
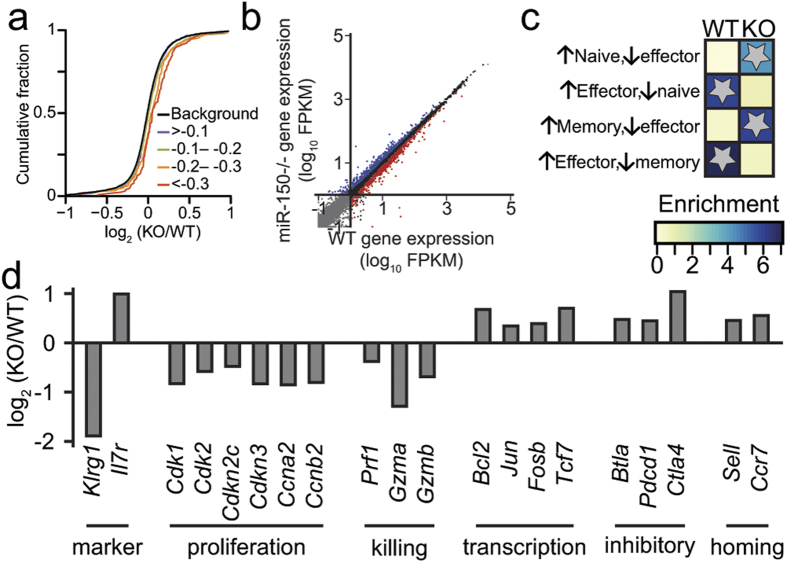
Transcriptome differences in effector miR-150^−/−^ CD8+ T cells at 7 days post-infection. (**a**) Cumulative distributions of mRNA fold-change between miR-150^−/−^ and WT cells for predicted miR-150 targets, binned by predicted strength, and background. Stronger targets are significantly different (context score −0.1 and −0.2 = p-value <0.05, −0.2, −0.3 = p-vlaue <0.05, context score <−0.3 = p-values <10^–4^). (**b**) Expression levels of all genes. The 462 genes whose expression is significantly higher in WT cells are red and the 380 genes whose expression is significantly higher in miR-150^−/−^ cells are blue. (**c**) Genes differentially expressed in miR-150^−/−^ cells compared to gene sets associated with naïve, effector, or memory cells. Significance was determined by a one-sided Fisher exact test (*denotes P < 10^−10^). (**d**) Expression differences between miR-150^−/−^ and WT cells for a subset of genes expressed in CD8+ T cells. All have a FDR <5%.

Next, we used mRNA sequencing to examine the 1,938 expressed predicted direct targets of miR-150. As expected, targets of miR-150 were significantly upregulated in miR-150^−/−^ cells compared to targets of other miRNAs (Fig. 4a). Importantly, transcripts predicted to be stronger targets of miR-150 were more highly upregulated than weaker targets (Fig. 4a), further suggesting that miR-150 is acting directly on genes with predicted target sites. Direct targets include a wide variety of genes, which were only modestly (less than two-fold) upregulated in miR-150^−/−^ cells (Supplemental Table S1). Therefore, it is likely that miR-150^−/−^ phenotypes are not driven solely by altered expression of one gene, but rather by miR-150’s ability to fine-tune^15^ the expression of many genes simultaneously. Additionally, these modest changes in expression of many direct targets likely propagate downstream to changes in expression of indirect targets. In particular, we examined the expression of *Myb,* which was previously shown to be a direct target of miR-150 and underlie miR-150-dependent development of B, NK, and NKT cells^10^. However, we surprisingly did not observe significant differences in *Myb* expression between miR-150^−/−^ and WT donor cells (Supplemental Table S2 and Supplemental Figure 5).

We expanded our analysis to identify indirect targets of miR-150. There are 842 genes with significant differential expression in miR-150^−/−^ cells, 380 of which are upregulated, and 462 of which are downregulated (Fig. 4b,d), demonstrating that a large number of genes from many pathways are part of the miR-150 gene regulatory network. To better understand the possible roles of miR-150 targets, both direct and indirect, we compared the differentially expressed genes to sets of genes that define naïve, effector, and memory CD8+ T cells, as curated by the Human Immunology Project Consortium^16^,^17^. Genes upregulated in miR-150^−/−^ cells significantly overlap with those preferentially found in naïve and memory cells, whereas downregulated genes significantly overlap with genes that define effector cells (Fig. 4c). For example, miR-150^−/−^ cells have decreased amounts of *Klrg1* and more *Il7r* (CD127), consistent with their surface phenotype (Fig. 1d). Additionally, cell-cycle related genes, including *Cdk1*, *Cdk2*, *Ccna2*, and *Ccnb2*, are downregulated in miR-150^−/−^ cells (Fig. 4d). miR-150^−/−^ cells also preferentially expressed transcription factors typically found in less differentiated effector cells, such as *Jun*, *Fosb*, *Tcf7*, and *Bcl2*^1^, and expressed elevated levels of inhibitory receptors (Fig. 4d). One such receptor, *Btla,* is amongst the direct targets of miR-150. Moreover, other negative regulators such as *Pdcd1* and *Ctla* are also enriched in miR-150^−/−^ cells, again suggesting that many modestly dysregulated genes contribute to the resulting cellular defects in miR-150^−/−^ cells. Lastly, to assess effector function in miR-150^−/−^ cells, we also examined expression of cytolytic genes and found levels of the cytotoxic proteases *Gzma* and *Gzmb* reduced in miR-150^−/−^ cells (Fig. 4d). Altogether, our RNA-Seq data shows that miR-150 plays a significant role in regulating the transcriptome of CD8+ T cells during infection.

### miR-150 expression contributes to CD8+ killing efficiency

**Figure 5:**
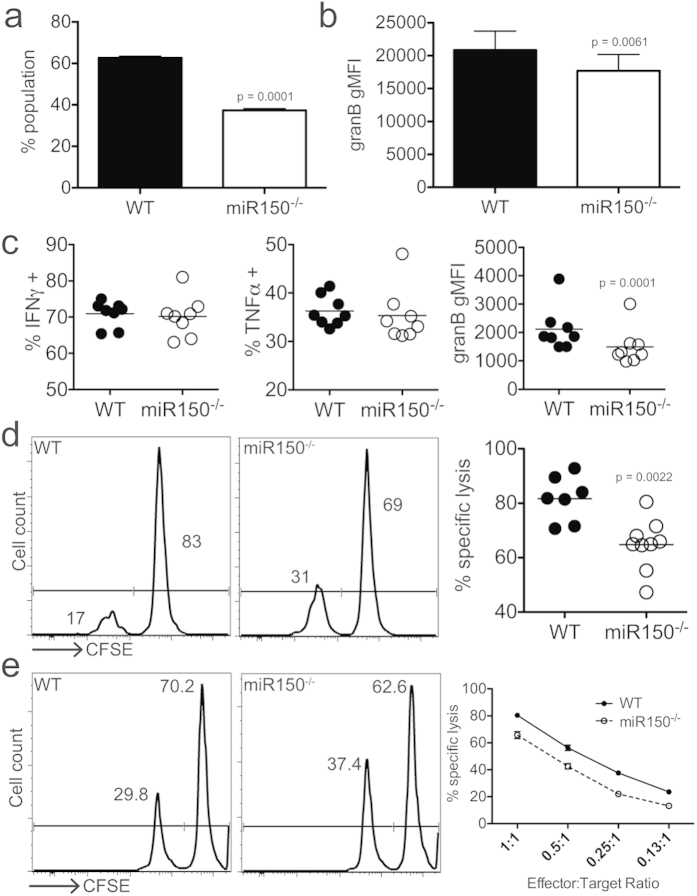
CD8+ T cell-mediated killing efficiency depends on miR-150 expression. (**a**) Percentage of total cell population from *in vitro* stimulation (72 hrs) in co-culture of WT and miR150^−/−^ cells (starting ratio 1:1). (**b**) granzyme B expression in cells stimulated *in vitro*. (**c**) IFNγ, TNFα and granzyme B production in *ex vivo* restimulated effector CD8+ T cells (7 dpi). Assessment of *in vivo* (**d**) or *in vitro* (**e**) killing. (*left*) Histograms showing relative percentages of specific (CFSE^lo^) and irrelevant (CFSE^hi^) target cells. (*right*) Evaluation of specific lysis of targets by donor CD8+ T cells. Representative of 2 experiments, n = 3 (a,b,e; error bars represent SD) or *n* = 4–8 (**c**,**d**). Statistics: paired (**a**–**c**) or unpaired (**d**) Student *t* test.

The increased expression of negative regulators, combined with lower expression of granzymes, suggested that cytolytic functions were impaired in miR-150^−/−^ cells. To test this possibility directly, we co-cultured both WT and miR-150^−/−^ donor populations *in vitro* (1:1 ratio) and compared their ability to produce granzyme B following stimulation. At 3 days post-stimulation, we found the ratio of donor cells was skewed in favor of WT cells, which also made more granzyme B (Fig. 5a,b). We also assessed effector functions *in vivo*, by restimulating donor cells from mice infected with *Lm*-gB. Interestingly, WT and miR-150^−/−^ cells made similar amounts of the effector cytokines IFNγ and TNFα; however, miR-150^−/−^ cells again produced significantly less granzyme B (Fig. 5c). To determine if less granzyme B expression resulted in reduced killing efficiency, we assessed miR-150^−/−^ and WT cell-mediated elimination of gB_498-505_-coated target cells *in vivo*. We performed single transfers into congenic recipients and assessed killing function of each donor population 7 days after *Lm*-gB infection. Mice that received miR-150^−/−^ cells displayed significantly less specific killing compared to those that received WT cells (Fig. 5d).

Importantly, since we had already established that miR-150^−/−^ cells fail to expand as robustly as WT cells (Fig. 1c), we could not determine whether the differences seen in killing were due to differences in effector cell population size in the recipients, rather than altered killing efficiency in miR-150^−/−^ cells. To address this, we evaluated killing in an *in vitro* assay where we could tightly control effector to target ratios. We again found that miR-150^−/−^ cells were less capable in killing specific targets (Fig. 5e). Together, these data confirm that in addition to proliferating less than WT cells, miR-150^−/−^ cells are impaired in effector function, even when assessed on a per cell basis.

## Discussion

Our study uncovers a surprising role for miR-150 in the differentiation of effector CD8+ T cells. Previously, miR-150 was shown to serve as a tumor suppressor and restrain uncontrolled cell division in T cell acute lymphoblastic leukemia (T-ALL) cell lines and NK/T-cell lymphoma cells^18^,^19^. Consistent with a growth-restricting role in cancer cells, miR-150 was also shown to limit proliferation in B cells by downregulating expression of *Myb*^10^. Thus, we suspected that CD8+ T cells lacking miR-150 would respond more vigorously to infection. Instead, our data shows that naïve miR150^−/−^ CD8+ T cells fail to undergo robust expansion and consequently become less differentiated effector cells with reduced cytotoxic killing capabilities. After contraction, some miR-150^−/−^ cells enter the memory pool but still fail to mount a robust recall response to repeat infections and remain less differentiated than their WT counterparts. These findings provide a cautionary note about ascribing the function of miRNAs in CD8+ T cells, based upon their role in transformed cells or other immune cell types.

Other studies have described a wide variety of roles for miR-150 in the immune system, which appears to depend upon the cell context. For example, deletion of miR-150 in mice results in the accumulation of splenic B cells^10^ but a decrease in the number of NK and iNKT cells^11^,^12^. In terms of function, miR-150 is required for robust IFNγ secretion in NK cells after stimulation^11^ but negatively regulates the production of cytokines in iNKT cells^12^. Despite the fact that miR-150 exhibits such divergent roles in various immune cell types, its mechanism is often ascribed to its regulation of the transcription factor *Myb.* However, we did not observe significant differences in the expression of *Myb* in miR-150^−/−^ versus WT CD8+ T cells. It is worth noting that because *Myb* generally acts to increase the proliferation rate, an increase in *Myb* expression would not be consistent with the observed phenotype in miR-150^−/−^ effector CD8+ T cells. Regardless, it is interesting to speculate on why the regulation of *Myb* by miR-150 appears to be dependent upon the cell context.

One possibility is that *Myb* expression is tightly regulated via feedback loops in CD8+ T cells, so its transcription is repressed to restrain *Myb* levels in the absence of negative regulation by miR-150. Although such a model would explain the equivalent levels of *Myb* in WT and miR-150^−/−^ cells, it still implies that the phenotypes we observed in the absence of miR-150 are not attributed to alterations in *Myb*. Alternatively, miRNA regulation of *Myb* in CD8+ T cells could occur primarily through translation repression, rather than through mRNA decay, and thus not be detectable by transcriptome profiling. It is worth noting, though, that regulation of *Myb* by miR-150 in iNKT cells and cancer cell lines does occur by accelerated mRNA decay^12^,^20^. Another possible explanation for why *Myb* expression was unchanged in miR-150^−/−^ cells could be that the 3′ untranslated region (3′UTR) isoform differs among cell types. Given that proliferating lymphocytes tend to have shortened 3′UTRs^21^, it would be interesting to determine if the isoform of *Myb* that is expressed in CD8+ T cells contains the miR-150 target site. A final possibility is that miR-150 target sites within *Myb* are masked by RNA binding proteins expressed in CD8+ T cells. Nevertheless, it is clear that Myb transcript levels are maintained at normal levels in miR-150^−/−^ cells.

Given that altered CD8+ T cell differentiation in miR-150^−/−^ cells appears to be independent of *Myb*, it is important to consider other targets that may be involved. Previous work has shown that increasing miR-150 expression in CD8+ T cells caused repression of CD25 (Il2ra)^22^, a receptor that transmits inflammatory signaling to the cell, after activation. These results predict that miR-150^−/−^ cells would express higher levels of CD25 expression, undergo more proliferation and rapidly become terminally differentiated. However, we observed less proliferation and differentiation by miR-150^−/−^ cells after infection and we were not able to detect a significant difference in CD25 expression between miR-150^−/−^ and WT cells. These discrepancies could be because the former study overexpressed miR-150 in CD8+ T cells via lentiviral transduction, whereas we used cells that were deficient in miR-150: overexpression of a miRNA could cause additional targeting that is not observed when endogenous levels of the miRNA are present. Additionally, lentiviral transductions must be performed on cells that have been activated *in vitro* for 1–2 days, precluding examination of the roles of miRNAs during priming. Thus, additional studies may be warranted to confirm the importance of CD25 regulation by miR-150 in CD8+ T cells.

We also considered miR-150 targets that were previously identified in other studies, including *Notch3*, *Akt2*, and *Dkc1*^18^,^19^. However, none of these targets appear to be differentially expressed in CD8+ T cells lacking miR-150. In examining the transcriptional differences between WT and KO CD8+ T cells, we observed a fairly small number of direct targets, all of which had modest differences in expression. In contrast, there were significantly more indirect targets (842 genes) that showed more dramatic changes in gene expression. This data suggest that the regulation of transcripts by miR-150 in CD8+ T cells is likely complex, involving multiple miRNA:target gene interactions, together with extensive alterations downstream of the direct targets.

Many miRNAs have regulated expression during effector cell differentiation^22^,^23^, thus, it is interesting to consider how this network of miRNAs works together in order to ensure a proper response to infection. Like miR-150, miR-155 and miR-17~92 are required for effector cell differentiation^24^,^25^,^26^,^27^. These miRNAs may regulate different aspects of the CD8+ T cell response, but they may also have redundancy, thus protecting the cell from misregulation of any single miRNA. Further studies into how these miRNAs interact will provide additional understanding of how CD8+ T cells regulate effector cell functions.

Clearly, more studies are needed to clarify how and when miR-150 regulates effector CD8+ T cell differentiation during microbial challenge. Earlier work showed that miR-150 is rapidly downregulated in CD8+ T cells following stimulation with peptide, but expression levels return when peptide is removed and cells are incubated with IL-15 for a few days^28^. In mice infected with LCMV, miR-150 was found to be significantly downregulated in effector CD8+ T cells at 5 dpi compared to naïve cells, but expression levels increased at later time points (8 dpi and >60 days)^23^. These data imply a reciprocal relationship, whereby elevated levels of antigen or inflammation drives the down-regulation of miR-150 expression in effector CD8+ T cells. Given that a number of negative regulators (*BTLA, CTLA4, PD-1, IKZF2*) were more abundantly expressed in miR-150^−/−^ CD8+ T cells, it is interesting to speculate that high amounts of antigen or inflammation during chronic infection may lead to the up-regulation of negative regulators through the sustained repression of miR-150. In this way, miR-150 may serve as a rheostat and limit immunopathology during infectious scenarios involving high amounts of viral replication. Although this remains to be tested with pathogens that elicit a high level of viremia, our current study is an important first step in identifying miR-150 as a key regulator of the fate and function of CD8+ T cells.

## Methods

### Mice

Adult (2–4 months) male mice were used in all experiments. Mice were maintained under pathogen-free conditions at Cornell University College of Veterinary Medicine, accredited by the American Association of Accreditation of Laboratory Animal Care. The Institutional Animal Care and Use Committee at Cornell University approved all protocols for mouse usage. MiR-150^−/−^ mice were purchased from Jackson Laboratory and crossed to gBT-I TCR transgenic mice (TCRαβ specific for the HSV-1 glycoprotein B_498-505_ peptide SSIEFARL) that were provided by Dr. Janko Nikolich-Zugich (University of Arizona, AZ). B6-Ly5.2/Cr and Thy1.1 mice were purchased from the National Cancer Institute and Jackson Laboratory, respectively.

### Adoptive transfers and Infections

Single cell suspensions of splenocytes from gBT-I miR-150^−/−^ and wild-type gBT-I (WT) mice were obtained by manual tissue dissociation through sieves. Untouched, naïve CD8+ cells were isolated by negative magnetic selection: Splenocytes were labeled with biotinylated antibodies to CD4 (GK1.5), CD19 (eBio1D3), CD16/32 (93), MHC-II I-A/I-D (M5/114.15.2) and Ter119 (Ter119) (Ebioscienes or Biolegend). Subsequently, cells bearing these markers were removed using magnetic streptavidin microbeads (Miltenyi). Equal numbers of each type of cell (1 × 10^4^) were co-transferred intravenously (i.v.) into congenic B6-Ly5.2/Cr recipients. The next day we infected mice with either *Listeria monocytogenes* (*Lm*-gB) (5 × 10^3^ CFU, i.v.) or vaccinia virus (VACV-gB) (2 × 10^5^ PFU, i.p.), as previously described^29^,^30^. For co-transfers of phenotypically equivalent populations of miR-150^−/−^ and WT CD8+ T cells, cells were magnetically enriched for untouched CD8+ cells and then sorted with an Aria cell sorter based on CD44 and CD122 expression patterns. Then equal numbers of miR-150^−/−^ and WT cells from each corresponding subset were transferred into recipient mice, which were subsequently infected with *LM-gB*.

### RNA sequencing

Cells were sorted with an Aria cell sorter to >95% purity into TRIzol (Life-technologies). For the small RNA libraries, 450 ng of total RNA were used to generate libraries using the TruSeq Small RNA Sample Preparation Kit (Illumina). The resulting libraries were sequenced on the Illumina Hi-Seq platform, generating 50 bp reads. Reads were filtered out if they failed Illumina’s quality control (had a tag of “Y”), lacked the adapter sequence (TGGAAT), were too short after having the linker trimmed (<15 nt), or had an ambiguous base call in the trimmed sequence. Trimmed reads that passed our quality control were matched to the genome (mm9) using Bowtie (Langmead), and genome-matching reads were then aligned to mature mouse hairpin sequences from MiRBase (version 20) (Kozomara). For each hairpin, we found the number of matching reads, then used the reads that matched the dominant isoform of that hairpin in subsequent analysis.

For the mRNA libraries, 200 ng of total RNA were used to generate libraries using the TruSeq RNA Sample Preparation Kit v2 (Illumina). The resulting libraries were sequenced on the Illumina He-Seq platform, generating 100 bp reads. We trimmed off nucleotides from the ends if they had Phred quality scores <20. Reads that were at least 20 nt long after trimming were aligned to the genome (mm9) using Tophat (Trapnell 2009). We used the mm9 GTF file provided by UCSC (available from the Tophat website). Differential expression of genes between samples was determined using CuffDiff (Trapnell 2013) with a false discovery rate of 5%.

These data were deposited in the Gene Expression Omnibus, under accession number GSE64687.

### Predicting microRNA target sites

Targets of miR-150 were predicted as genes with a context + score <−0.2 from TargetScan^31^,^32^. The background set was comprised of targets of other well-conserved miRNAs^8^ with a context + score ≤−0.2. Two-sided Kolmogorov–Smirnov tests were used to determine significant differences in targeting.

### Flow Cytometry

Fluorochrome-labeled monoclonal antibodies were purchased from Biolegend, Ebioscience, BD Biosciences or Life Technologies. The following clones were used: anti-CD8α (53-6.7), anti-CD4 (RM4-5), anti-CD45.1 (A20), anti-CD45.2 (104), anti-CD90.1/Thy1.1 (OX-7), anti-KLRG1 (2F1), anti-CD127 (A7R34). anti-CD27 (LG.7F9), anti-CD62L (mel14), anti-CXCR3 (CXCR3-173), anti-CD44 (IM7), anti-IFNγ (XMG1.2), anti-TNFα (MP6-XT22) and anti-granzyme B (GB11). Foxp3 staining buffer set was used to examine expression of intracellular proteins according to manufacturer’s instructions (eBioscience). Data was collected using an LSR II flow cytometer (BD Biosciences) and analyzed with FlowJo (Treestar).

### *In vitro* stimulation

CD8+ T cells from congenically marked gBT-I WT and gBT-I miR-150^−/−^ were positively selected from splenocytes using CD8 microbeads (Miltenyi) according to manufacturer’s instructions. CD8+ T cells from WT and miR-150^−/−^ were mixed 1:1 and were labeled with CFSE (5 mM). Cells were washed to remove excess CFSE and then 1 × 10^5^ cells were stimulated with 10^−9^ M gB peptide in the presence of 10 ng/ml IL-2. After 72 hours, cells were stained for surface markers followed by viability dye staining, using Fixable viability dye eFluor780 (eBioscience) according to manufacturer’s instructions. Cells were then fixed and stained for granzyme B using eBiocience’s FoxP3 staining buffer set.

### Killing assays

To prepare target cells, splenocytes from congenically marked mice were isolated. Half of the cells were loaded with gB peptide (1 μM) (specific targets) while the other half were not (non-specific targets). Following peptide loading, specific targets were labeled with 5 nM CFSE while non-specific targets were labeled with 5 pM CFSE and were then mixed in a 1:1 ratio. *In vivo*, 1 × 10^7^ CFSE-labeled target cells were transferred (i.v.) into infected single adoptive transfer recipients 7 dpi and specific killing assessed in lung at 30 minutes. *In vitro*, 7 dpi donor effector cells were isolated from single adoptive transfer recipients via congenic marker expression. Briefly, donor cells were recovered from recipients by isolating splenocytes, staining with anti-CD45.2-APC and performing magnetic selection with anti-APC microbeads (Miltenyi). These effector cells were mixed with target (T) cells at indicated E:T ratios. Specific killing assessed after 8 hours.

### Statistics

Individual values were plotted and the mean indicated with a horizontal line. Statistical tests performed for specific experiments indicated in figure legends and p values are reported in the figures.

## Additional Information

**How to cite this article**: Smith, N. L. *et al.* miR 150 Regulates Differentiation and Cytolytic Effector Function in CD8+ T cells. *Sci. Rep.* **5**, 16399; doi: 10.1038/srep16399 (2015).

## Supplementary Material

Supplementary Information
