# Supplementary material for: miR-150 Regulates Differentiation and Cytolytic Effector Function in CD8+ T cells
Source: Sci Rep. 2015 Nov 9;5:16399. doi: 10.1038/srep16399 (PMC4637875; doi:10.1038/srep16399)
Supplement: Supplementary Information [file srep16399-s1.pdf]

miR-150 Regulates Differentiation and Cytolytic Effector Function in CD8+ T cells

Norah L. Smith[1], Erin M. Wissink[2], Andrew Grimson[2,*] and Brian D. Rudd[1,*]

Supplemental Information

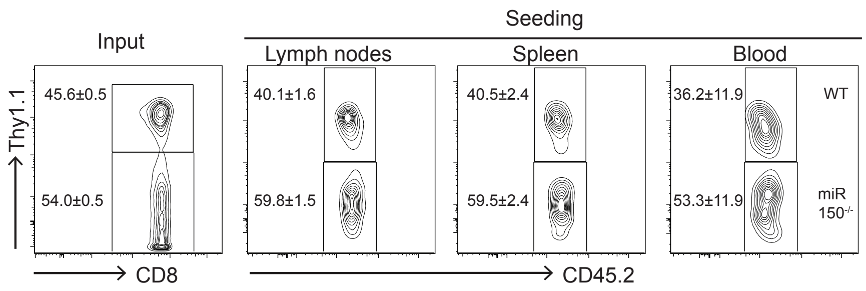

Figure S1. Contour plots with ratio of WT (Thy1.1+) and miR-150$^{-/-}$ (Thy1.1-) cells at time of adoptive transfer (*left*) and recovered from indicated recipient tissues 24 hours after transfer. Representative of two experiments, *n*=4. Average percentage of each population is displayed within gates ± S.D.

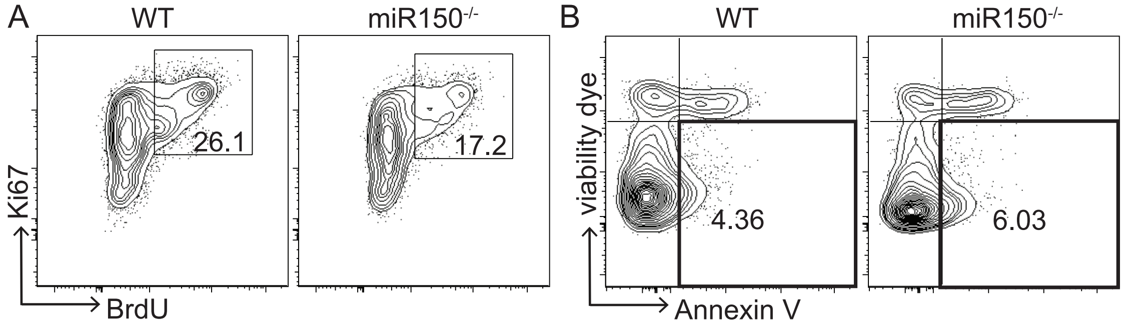

Figure S2. (A) Representative flow cytometric plots showing Ki67 and BrdU incorporation (7 dpi). (B) Representative flow cytometric plots of annexin V apoptosis staining (7 dpi). Negative staining for fixable viability dye efluor-780 was used to determine cell viability. (C) Representative flow cytometric histograms of CD44 and CD62L expression in naïve WT and miR-150$^{-/-}$ CD8+ T cells. Data are representative of at least two independent experiments, *n*=8.

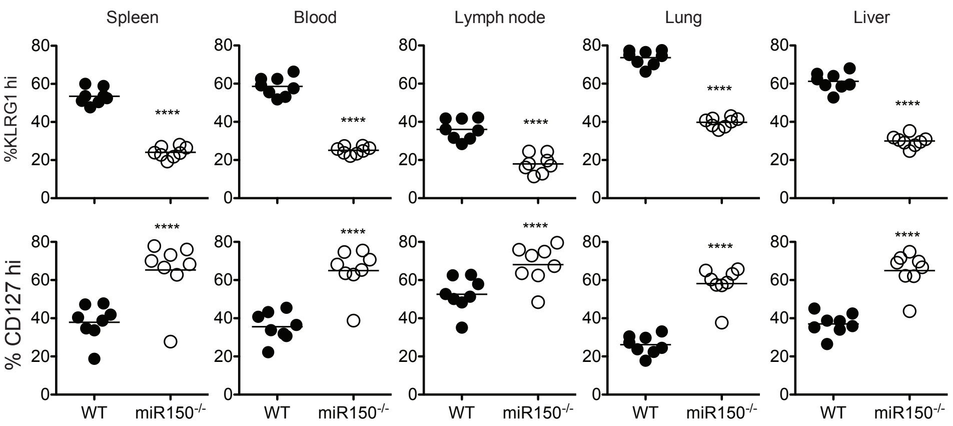

Figure S3. Effector cell surface phenotype of CD8+ T cells recovered from indicated tissues of co-transfer recipients 7 dpi. Data representative of 2 independent experiments, n=8. ****, p=0.0001, paired student t test.

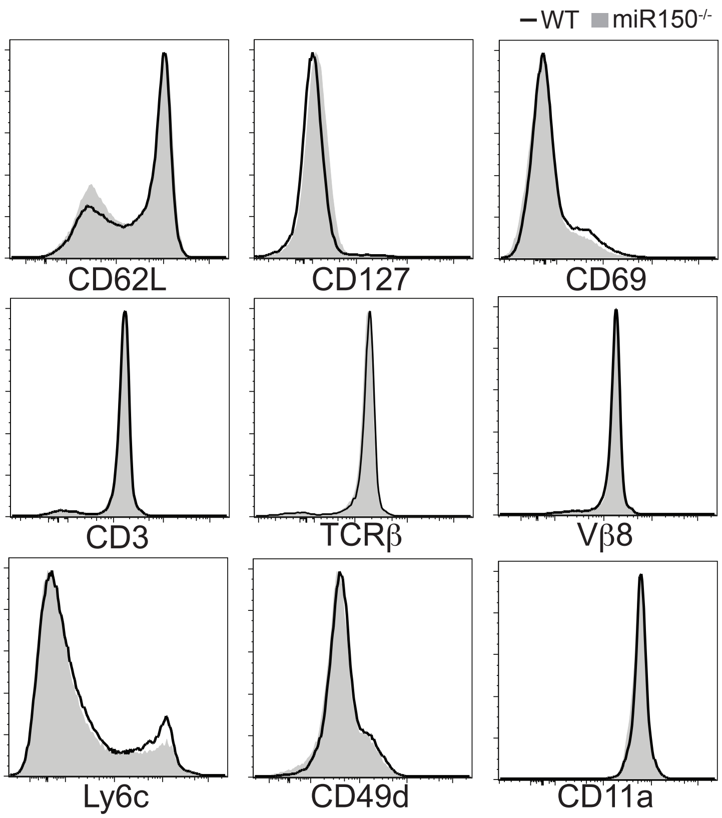

Figure S4. Representative histograms of surface markers in CD8+ splenocytes from naïve WT or miR150$^{-/-}$ gBT-I mice. Data representative of 2 independent experiments, n=4.

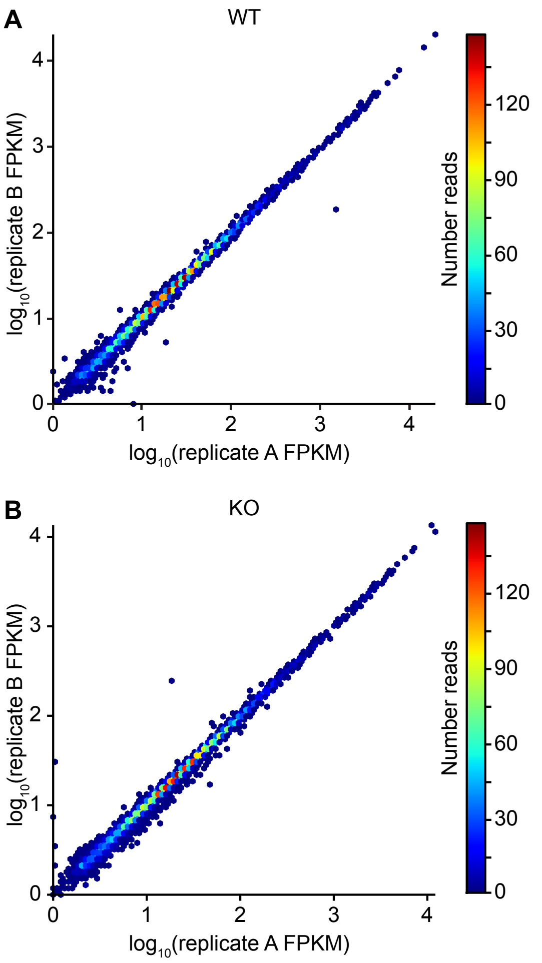

Figure S5. (A) FPKM values for genes that are coding and expressed (average FPKM >1) compared between biological replicates of WT cells. Pearson r>0.998, p<10^-15. (B) FPKM values for genes that are coding and expressed (average FPKM >1) compared between biological replicates of miR-150$^{-/-}$ cells. Pearson r>0.997, p<10^-15.

Supplemental Table S1. Differentially expressed miR-150 targets in WT and miR-150$^{-/-}$(KO) effector CD8+ T cells.

Gene	wt FPKM	KO FPKM	q value	log2 (KO/wt)	Context+ score
Adam19	49.5868	75.4729	0.000236	0.596188	-0.339
Btla	11.116	15.814	0.004073	0.472749	-0.402
Cd55	11.8494	20.0132	0.000236	0.709595	-0.24
Dirc2	2.84849	4.46119	0.001222	0.504923	-0.274
Dynlt3	4.88756	7.62214	0.003439	0.550376	-0.307
Fam174b	3.08904	6.5917	0.000236	0.892661	-0.411
Fn3k	1.22602	2.81961	0.015108	0.778959	-0.339
Gstt3	0.911744	1.81065	0.014843	0.556014	-0.479
Ikzf2	0.613104	1.69963	0.000236	0.742922	-0.245
Il18r1	168.659	248.039	0.001762	0.553734	-0.335
Lca5	1.28134	2.22315	0.002109	0.49859	-0.253
Mapk11	5.4152	10.0096	0.000236	0.779196	-0.213
Pim2	38.9578	59.4826	0.000236	0.598043	-0.262
Prrt2	1.34347	2.23175	0.017799	0.463669	-0.217
Sgms1	19.4662	31.3661	0.000236	0.66124	-0.203
Treml2	1.14707	2.72622	0.000236	0.795344	-0.204
Wbscr27	2.03491	3.05238	0.031072	0.417116	-0.242
Zscan12	1.11573	1.82421	0.011485	0.416692	-0.22

Supplemental Table S2. Expressed genes and FPKM values for WT and miR-150$^{-/-}$(KO) effector CD8+ T cells.

Gene	wt FPKM	KO FPKM	log2(KO/wt)	p value	q value	Sig
0610007P14Rik	35.8338	38.8185	0.115421	0.45855	0.616026	no
0610009B22Rik	8.4556	9.12655	0.110162	0.654	0.782569	no
0610009O20Rik	17.5443	19.4828	0.151199	0.3214	0.476267	no
0610010F05Rik	3.43524	3.7028	0.108207	0.5572	0.704152	no
0610010K14Rik	109.814	100.285	-0.130953	0.3791	0.538101	no
0610011F06Rik	21.7053	24.5578	0.178129	0.33705	0.493234	no
0610030E20Rik	14.2573	13.6488	-0.0629176	0.6647	0.790268	no
0610031J06Rik	99.7067	95.8185	-0.057387	0.6832	0.803975	no
0610037L13Rik	13.7562	14.3382	0.0597861	0.7342	0.83966	no
1110001J03Rik	29.8878	28.2844	-0.0795511	0.72125	0.830732	no
1110004E09Rik	16.166	17.0201	0.0742759	0.6855	0.805688	no
1110004F10Rik	70.5943	67.5087	-0.0644778	0.6547	0.782942	no
1110007C09Rik	27.218	32.191	0.242099	0.16825	0.293032	no
1110008F13Rik	51.4058	50.6038	-0.0226857	0.9316	0.962642	no
1110008L16Rik	2.75807	3.49465	0.341491	0.3055	0.458344	no
1110008P14Rik	71.4964	80.4221	0.169722	0.2849	0.435218	no
1110012L19Rik	17.4381	16.4035	-0.0882454	0.62495	0.759761	no
1110032A03Rik	1.72435	1.61315	-0.0961739	0.75215	0.851893	no
1110034G24Rik	10.8108	10.6614	-0.0200734	0.91225	0.9519	no
1110037F02Rik	24.4804	22.8228	-0.10115	0.47715	0.633216	no
1110038F14Rik	34.5615	32.2764	-0.0986868	0.54095	0.690253	no
1110057K04Rik	28.7798	24.8999	-0.208916	0.16725	0.291831	no
1110058L19Rik	27.9301	27.6046	-0.016911	0.9266	0.959991	no
1110059E24Rik	14.1733	12.2361	-0.212043	0.2392	0.384172	no
1110059G10Rik	4.70244	4.42152	-0.0888681	0.6241	0.759094	no
1110065P20Rik	9.7184	10.0344	0.0461578	0.9185	0.955413	no
1190002N15Rik	10.3868	12.3059	0.244597	0.10695	0.204241	no
1190007I07Rik	7.61867	6.8513	-0.153162	0.5706	0.715208	no
1200014J11Rik	13.2947	12.8206	-0.0523946	0.7211	0.830668	no
1500009L16Rik	22.9839	35.0084	0.607082	0.00025	0.00103712	yes
1500012F01Rik	102.713	93.6133	-0.13384	0.57355	0.717733	no
1600002H07Rik	3.55192	3.61944	0.0271663	0.9007	0.945017	no
1600002K03Rik	25.8916	31.2271	0.270315	0.1483	0.264727	no
1600012H06Rik	14.9289	12.4022	-0.267515	0.20595	0.343796	no
1600014C10Rik	31.0591	25.7654	-0.269581	0.06165	0.129706	no
1600016N20Rik	2.6409	2.82821	0.0988592	0.7366	0.841519	no
1700001O22Rik	2.86051	2.12574	-0.428309	0.1695	0.294766	no
1700009P17Rik	1.47147	1.07431	-0.453849	0.2894	0.440196	no
1700010I14Rik	2.34496	3.38295	0.528717	0.03615	0.0826436	no
1700017B05Rik	15.2503	22.656	0.571057	5.00E-05	0.000236281	yes
1700019G17Rik	3.06565	2.43662	-0.331315	0.1419	0.255689	no
1700020D05Rik	1.06422	1.67547	0.654761	0.3118	0.465451	no
1700020L24Rik	17.1543	15.7536	-0.122892	0.5171	0.669724	no
1700021F05Rik	12.1164	9.57029	-0.340327	0.1173	0.220164	no
1700021K19Rik	5.98803	5.55467	-0.108379	0.50185	0.65604	no
1700025G04Rik	8.71723	5.48573	-0.668186	5.00E-05	0.000236281	yes
1700026L06Rik	1.34105	0.704835	-0.928003	0.1512	0.268945	no
1700029I15Rik	1.44874	1.41869	-0.0302394	0.9078	0.949278	no
1700029J07Rik	0.491483	1.00562	1.03287	0.0062	0.0180576	yes

1700030K09Rik	3.41983	3.96472	0.213296	0.72	0.829861	no
1700037C18Rik	4.29959	4.79242	0.156556	0.552	0.699685	no
1700037H04Rik	15.5088	16.704	0.107107	0.5484	0.696673	no
1700052N19Rik	12.9571	12.9075	-0.00552972	0.97255	0.984885	no
1700056E22Rik	2.723	2.95987	0.120339	0.7134	0.825368	no
1700066M21Rik	4.40437	3.72888	-0.240192	0.2124	0.352089	no
1700088E04Rik	5.68602	5.83959	0.0384477	0.9368	0.965724	no
1700094D03Rik	4.51047	4.96373	0.138144	0.8482	0.913382	no
1700102P08Rik	1.38585	1.29876	-0.0936367	0.8173	0.894617	no
1700109H08Rik	1.51245	2.23983	0.566497	0.5819	0.724854	no
1700123O20Rik	23.2204	22.4795	-0.0467817	0.9224	0.957889	no
1810009A15Rik	75.3041	68.5524	-0.135521	0.40955	0.568563	no
1810013L24Rik	16.4834	17.792	0.110216	0.44465	0.602409	no
1810022K09Rik	148	144.241	-0.037118	0.83205	0.903643	no
1810026J23Rik	17.3777	17.0518	-0.0273161	0.8945	0.94117	no
1810030O07Rik	31.6132	29.1381	-0.117624	0.4176	0.576202	no
1810037I17Rik	83.2103	78.2442	-0.0887784	0.543	0.69218	no
1810043G02Rik	4.30932	5.44257	0.336831	0.12345	0.229274	no
1810043H04Rik	31.9964	32.5223	0.0235212	0.90715	0.948893	no
1810055G02Rik	1.49429	1.26452	-0.24087	0.4457	0.603356	no
2010012O05Rik	17.4355	7.45816	-1.22513	5.00E-05	0.000236281	yes
2010015L04Rik	0.908523	1.17362	0.369374	0.337	0.493202	no
2010107E04Rik	421.748	377.626	-0.159425	0.2841	0.434231	no
2010111I01Rik	30.0795	33.0731	0.13688	0.4614	0.618489	no
2200002D01Rik	1.2035	1.29047	0.100662	0.93665	0.965654	no
2210011C24Rik	1.84405	2.29303	0.314377	0.412	0.57092	no
2210013O21Rik	21.931	22.0468	0.00760182	0.96995	0.983692	no
2210016F16Rik	17.9993	19.891	0.144171	0.3402	0.49667	no
2210016L21Rik	28.9578	25.9433	-0.158592	0.32325	0.478245	no
2210018M11Rik	17.1597	15.6557	-0.132338	0.3688	0.527374	no
2210404O09Rik	1.86973	2.40052	0.360519	0.16025	0.281966	no
2300009A05Rik	18.0394	18.0945	0.00439936	0.98955	0.993761	no
2310003H01Rik	5.73181	5.84695	0.0286933	0.87375	0.92906	no
2310009B15Rik	8.20821	4.73614	-0.793355	0.0153	0.0395135	yes
2310011J03Rik	37.5517	35.873	-0.0659776	0.65935	0.786544	no
2310022A10Rik	12.9875	12.1192	-0.099827	0.5352	0.685568	no
2310022B05Rik	1.9408	2.5836	0.412731	0.06495	0.135198	no
2310033P09Rik	22.0431	22.6596	0.0397974	0.8125	0.891603	no
2310035C23Rik	14.7189	15.1037	0.0372351	0.7929	0.879239	no
2310036O22Rik	160.763	147.714	-0.122136	0.39135	0.550431	no
2310039H08Rik	11.1114	12.0125	0.112496	0.62175	0.757188	no
2310045N01Rik	93.6669	102.122	0.124688	0.4152	0.573957	no
2310047M10Rik	11.3438	10.8778	-0.0605131	0.7282	0.835319	no
2310057M21Rik	7.56868	7.5692	9.88E-05	0.9936	0.995999	no
2310061I04Rik	19.9755	20.48	0.0359841	0.8367	0.906434	no
2310067B10Rik	14.9831	14.245	-0.0728755	0.6115	0.748879	no
2410002F23Rik	14.9457	17.0075	0.186433	0.243	0.387831	no
2410004B18Rik	28.9508	24.7081	-0.228617	0.17735	0.305638	no
2410015M20Rik	88.0771	85.7412	-0.0387774	0.8019	0.884923	no
2410016O06Rik	10.9255	12.0284	0.138753	0.40215	0.56117	no
2410076I21Rik	1.02699	0.651651	-0.656245	0.78525	0.874329	no
2410089E03Rik	4.36869	4.4768	0.0352664	0.8196	0.89605	no
2410127L17Rik	19.1111	19.3513	0.018023	0.9067	0.948633	no
2410131K14Rik	5.68991	6.00662	0.0781469	0.67105	0.795166	no
2510002D24Rik	22.1068	26.4862	0.260754	0.1181	0.221359	no
2510003E04Rik	11.4562	11.9247	0.0578348	0.7308	0.837264	no
2510039O18Rik	56.402	54.3279	-0.0540537	0.7007	0.816361	no

2610002J02Rik	31.9807	31.4847	-0.0225518	0.8874	0.936912	no
2610002M06Rik	5.52696	5.32105	-0.0547755	0.73135	0.837636	no
2610008E11Rik	5.58569	5.78968	0.0517497	0.769	0.863206	no
2610015P09Rik	1.89747	2.60477	0.457079	0.03525	0.0808585	no
2610018G03Rik	48.3034	47.5252	-0.0234325	0.87375	0.92906	no
2610020H08Rik	21.6443	26.5903	0.29691	0.0533	0.114734	no
2610044O15Rik8	2.37258	2.34671	-0.0158201	0.96255	0.979918	no
2610301B20Rik	4.33235	5.58385	0.366109	0.0691	0.142259	no
2610318N02Rik	8.99185	6.90525	-0.380925	0.072	0.147225	no
2610507B11Rik	38.2227	41.5214	0.119428	0.4025	0.561448	no
2610524H06Rik	14.7486	12.4614	-0.24311	0.2194	0.360518	no
2610528A11Rik	4.04166	0.913098	-2.14611	0.00105	0.00375607	yes
2700029M09Rik	54.7569	48.6286	-0.171235	0.25555	0.400427	no
2700049A03Rik	2.56355	2.73881	0.0954031	0.6012	0.740802	no
2700060E02Rik	93.8625	99.0439	0.0775188	0.587	0.729148	no
2700062C07Rik	13.2033	12.0854	-0.127626	0.5093	0.662808	no
2700081O15Rik	3.58565	2.80987	-0.351732	0.5343	0.684751	no
2700089E24Rik	12.2584	12.638	0.0439993	0.8325	0.903937	no
2700094K13Rik	96.371	79.9345	-0.269781	0.0833	0.166214	no
2700097O09Rik	3.52293	3.46378	-0.0244292	0.9285	0.961	no
2810004N23Rik	22.9142	26.5025	0.209888	0.1975	0.332926	no
2810006K23Rik	10.2323	9.20235	-0.153054	0.40285	0.561836	no
2810021J22Rik	3.15186	2.62393	-0.264475	0.15455	0.273779	no
2810403A07Rik	30.0166	28.3185	-0.0840123	0.5664	0.711701	no
2810408M09Rik	10.1647	12.1837	0.261388	0.1327	0.243045	no
2810417H13Rik	63.1645	33.6636	-0.907923	0.0031	0.00983478	yes
2810428I15Rik	39.6324	44.8074	0.177059	0.30965	0.463104	no
2810474O19Rik	18.8491	23.0469	0.290072	0.0442	0.0980121	no
3010026O09Rik	3.55291	3.61551	0.0251981	0.92715	0.960307	no
3110001I22Rik	5.5058	5.10492	-0.109064	0.81125	0.890853	no
3110002H16Rik	42.3645	41.821	-0.0186272	0.9275	0.960472	no
3110009E18Rik	11.3074	8.72263	-0.374435	0.1991	0.334983	no
3110040N11Rik	27.2574	27.1231	-0.00712815	0.9673	0.982218	no
3110043O21Rik	1.55285	1.80494	0.217038	0.3764	0.535205	no
3110052M02Rik	5.30998	5.31479	0.00130569	0.99625	0.997296	no
3110057O12Rik	4.69607	4.80949	0.03443	0.85785	0.919663	no
3110062M04Rik	5.61464	5.40717	-0.0543182	0.76495	0.860549	no
3830403N18Rik	1.82672	3.45007	0.917373	0.0107	0.0290897	yes
3830406C13Rik	18.2853	17.3676	-0.0742806	0.63315	0.766285	no
4632428N05Rik	106.761	116.189	0.122091	0.3841	0.543156	no
4632434I11Rik	2.3274	1.82394	-0.351658	0.11835	0.221769	no
4833420G17Rik	42.9832	39.982	-0.104421	0.4725	0.629079	no
4833439L19Rik	26.1223	24.7284	-0.0791123	0.61745	0.753955	no
4921524J17Rik	15.7827	15.6857	-0.00888671	0.95665	0.976752	no
4930402H24Rik	4.8295	5.0589	0.0669492	0.69005	0.809055	no
4930404N11Rik	1.48722	2.55327	0.779731	0.16265	0.285349	no
4930427A07Rik	7.67883	6.05848	-0.341932	0.04535	0.100175	no
4930430F08Rik	2.55896	2.9697	0.214758	0.37395	0.532623	no
4930453N24Rik	69.8824	69.2991	-0.0120928	0.93455	0.964438	no
4930486L24Rik	14.4841	14.4266	-0.00574108	0.973	0.985143	no
4930503L19Rik	15.3265	13.6436	-0.16781	0.30405	0.456848	no
4930523C07Rik	29.9594	27.4577	-0.125798	0.4301	0.588389	no
4930579G24Rik	11.4667	7.4988	-0.612718	0.00095	0.00343861	yes
4931406C07Rik	2.28518	2.0238	-0.175244	0.80605	0.887519	no
4931406P16Rik	12.3366	11.9668	-0.0439087	0.76795	0.862522	no
4931414P19Rik	4.78798	4.39458	-0.123692	0.52045	0.672545	no
4931428F04Rik	2.17077	2.71407	0.322247	0.12775	0.235716	no

4932438A13Rik	25.0578	26.6306	0.0878293	0.5357	0.685997	no
4933411K20Rik	7.60379	8.66746	0.18889	0.233	0.376933	no
4933426M11Rik	20.8809	22.4793	0.106416	0.45065	0.608075	no
4933427D14Rik	11.1124	8.76533	-0.342293	0.02715	0.0648868	no
4933434E20Rik	36.6043	36.7107	0.00418757	0.99165	0.99492	no
5031414D18Rik	1.0487	1.34071	0.354391	0.23095	0.374381	no
5031439G07Rik	2.11318	2.52552	0.257167	0.19345	0.327413	no
5430427O19Rik	5.69883	5.80392	0.0263623	0.87635	0.930717	no
5730455P16Rik	5.47203	5.04627	-0.116858	0.4828	0.638397	no
5730507C01Rik	1.0063	1.17777	0.227004	0.45495	0.612694	no
5730508B09Rik	18.9463	19.3919	0.0335387	0.83605	0.906063	no
5830411N06Rik	1.58281	2.04664	0.370767	0.12665	0.234097	no
5830415F09Rik	4.55252	5.38625	0.242615	0.2764	0.425428	no
5830418K08Rik	9.2405	9.30409	0.00989281	0.9606	0.978837	no
6030458C11Rik	13.1262	15.6924	0.257624	0.08275	0.165266	no
6330403K07Rik	10.014	6.17551	-0.69739	0.00035	0.0014053	yes
6330409D20Rik	1.60965	1.55189	-0.0527229	0.91395	0.952853	no
6330416G13Rik	8.97877	8.96296	-0.00254239	0.989	0.993518	no
6720489N17Rik	1.12893	0.554337	-1.02612	0.00565	0.0166536	yes
8030462N17Rik	9.2774	8.59147	-0.110815	0.4974	0.651804	no
8430419L09Rik	6.61275	7.05578	0.0935544	0.5708	0.715408	no
9030025P20Rik	1.48597	1.33372	-0.155943	0.58875	0.730589	no
9030617O03Rik	3.27262	7.01119	1.09921	5.00E-05	0.000236281	yes
9030624J02Rik	27.1028	25.8531	-0.0681075	0.62935	0.763126	no
9130011E15Rik	6.40826	6.09685	-0.0718686	0.6899	0.808973	no
9130019O22Rik	0.897863	1.13263	0.335112	0.2111	0.350527	no
9130023H24Rik	2.41086	2.16108	-0.157796	0.7167	0.827517	no
9130401M01Rik	15.2124	14.4095	-0.0782284	0.65805	0.785511	no
9430015G10Rik	9.29827	9.22276	-0.0117647	0.9425	0.968799	no
9430016H08Rik	12.4215	13.5979	0.130535	0.5542	0.701604	no
9430038I01Rik	5.30775	6.00476	0.178006	0.4638	0.620845	no
9530068E07Rik	33.1338	35.3238	0.0923359	0.5215	0.673556	no
9630033F20Rik	12.1816	11.5966	-0.071009	0.6427	0.773651	no
9930012K11Rik	6.33892	6.28856	-0.0115086	0.94885	0.97238	no
9930021J03Rik	5.16313	4.69537	-0.137009	0.37405	0.53269	no
9930104L06Rik	5.13735	5.70355	0.150836	0.41015	0.569104	no
9930111J21Rik1	29.6371	32.9931	0.154756	0.31815	0.472482	no
a	1.39568	1.02437	-0.446234	0.41555	0.57426	no
A130010J15Rik	2.43648	2.38542	-0.0305548	0.9698	0.983653	no
A230046K03Rik	13.4538	14.6177	0.119701	0.40665	0.56556	no
A230050P20Rik	12.0708	13.139	0.122326	0.5054	0.659399	no
A430005L14Rik	25.6359	24.5011	-0.0653181	0.69715	0.81408	no
A430033K04Rik	2.35003	3.1041	0.401496	0.0607	0.128028	no
A430078G23Rik	37.3551	31.6523	-0.238996	0.09025	0.177646	no
A430107P09Rik	2.63613	1.42053	-0.891993	0.00175	0.00592472	yes
A530054K11Rik	5.93733	5.29661	-0.164746	0.3117	0.465361	no
A630001G21Rik	47.3392	43.3345	-0.12752	0.3808	0.539787	no
A630007B06Rik	10.1514	9.92412	-0.0326702	0.8415	0.9094	no
A630033H20Rik	6.92402	5.49533	-0.333404	0.06095	0.128482	no
A730008H23Rik	11.8273	10.0541	-0.234343	0.5553	0.702417	no
A830080D01Rik	7.23946	5.95007	-0.282976	0.09595	0.186864	no
AA414768	3.5498	1.60837	-1.14214	0.00035	0.0014053	yes
AA415398	0.976522	1.11383	0.189806	0.5902	0.73177	no
AA467197	23.8479	18.6943	-0.351267	0.0837	0.166866	no
AA987161	7.22339	6.90623	-0.0647768	0.6808	0.802355	no
Aaas	30.5781	27.2935	-0.163942	0.285	0.435314	no
Aacs	4.43146	5.42189	0.291012	0.1114	0.211122	no

Aaed1	24.5935	28.2553	0.200241	0.2414	0.385984	no
Aagab	28.7887	29.1361	0.0173049	0.9045	0.947357	no
Aak1	19.6949	20.2504	0.0401287	0.7746	0.867107	no
Aamdc	22.1848	22.7558	0.0366629	0.849	0.913881	no
Aamp	81.5278	79.2853	-0.0402383	0.77655	0.868339	no
Aar2	5.52297	4.98941	-0.146575	0.4006	0.559616	no
Aars	27.8947	24.7467	-0.172756	0.22575	0.368431	no
Aars2	4.62722	4.54333	-0.026396	0.8877	0.937033	no
Aarsd1	20.4266	21.0937	0.0463629	0.7848	0.874075	no
Aasdh	3.45933	3.65782	0.0804903	0.6689	0.793564	no
Aasdhppt	7.25086	7.63331	0.0741561	0.66795	0.792797	no
Aatf	14.7606	16.1964	0.133924	0.41275	0.571521	no
AB124611	70.9444	76.7646	0.113754	0.43205	0.590244	no
Abca2	52.0896	59.6521	0.195578	0.1657	0.289713	no
Abca3	6.81365	12.5177	0.877465	5.00E-05	0.000236281	yes
Abca7	34.1567	32.531	-0.0703563	0.6196	0.755587	no
Abcb10	2.85935	3.26972	0.193481	0.29985	0.452165	no
Abcb1a	11.2847	7.75339	-0.541467	4.00E-04	0.00158415	yes
Abcb1b	10.4932	11.215	0.0959827	0.52565	0.677169	no
Abcb6	1.16048	1.28362	0.145501	0.59455	0.735355	no
Abcb7	11.1832	10.947	-0.0308069	0.8336	0.904561	no
Abcb8	7.22895	8.6444	0.25798	0.1272	0.234922	no
Abcb9	4.84355	5.07559	0.0675117	0.70675	0.820782	no
Abcc1	5.71501	7.50064	0.392259	0.0112	0.030266	yes
Abcc10	4.55678	4.93324	0.11452	0.48815	0.64323	no
Abcc4	9.72636	9.79294	0.00984293	0.94715	0.971428	no
Abcc5	11.8795	12.9087	0.119875	0.552	0.699685	no
Abcd1	19.5575	17.8118	-0.134894	0.36785	0.526367	no
Abcd3	4.96977	5.06959	0.0286894	0.8738	0.929085	no
Abcd4	10.8708	9.1064	-0.255505	0.12485	0.231412	no
Abce1	30.1208	33.5845	0.157036	0.27985	0.429483	no
Abcf1	71.5709	71.555	-0.000320853	0.9986	0.998812	no
Abcf2	22.2428	24.1411	0.118155	0.4273	0.585659	no
Abcf3	14.5681	16.2585	0.158389	0.2945	0.446003	no
Abcg1	13.0316	13.2755	0.0267517	0.855	0.917876	no
Abcg2	1.02368	1.40454	0.456333	0.12665	0.234097	no
Abcg3	11.6553	8.75663	-0.412538	0.01105	0.0299157	yes
Abhd1	1.68458	2.04058	0.276585	0.8855	0.93597	no
Abhd10	10.361	10.3723	0.00157102	0.9943	0.996304	no
Abhd11	25.0624	24.7798	-0.0163615	0.92325	0.95818	no
Abhd12	24.0201	23.1995	-0.0501471	0.7481	0.849282	no
Abhd13	9.22693	9.54502	0.0488978	0.74985	0.850448	no
Abhd14a	4.35955	7.46213	0.775407	0.00145	0.00501121	yes
Abhd14b	8.31725	10.2438	0.300566	0.1165	0.218983	no
Abhd15	1.54872	2.88034	0.895167	2.00E-04	0.0008488	yes
Abhd16a	32.64	32.8657	0.00994239	0.94755	0.97174	no
Abhd17a	53.9086	54.399	0.0130664	0.9279	0.960632	no
Abhd17b	14.3304	12.827	-0.159895	0.31675	0.470879	no
Abhd17c	7.63457	7.5101	-0.0237151	0.89525	0.941567	no
Abhd2	27.6131	30.2385	0.131034	0.36425	0.522519	no
Abhd4	2.59707	3.14191	0.274755	0.21665	0.35733	no
Abhd5	8.44108	7.82827	-0.108733	0.50645	0.660292	no
Abhd6	3.70643	3.86634	0.0609377	0.7856	0.874428	no
Abhd8	42.7107	50.7068	0.247581	0.08675	0.171859	no
Abi1	71.1941	65.5614	-0.118911	0.4076	0.566479	no
Abi2	3.80634	5.90327	0.633107	3.00E-04	0.00122185	yes
Abi3	4.52828	4.03386	-0.166803	0.40855	0.567498	no

Abl1	10.3859	10.6073	0.0304323	0.8314	0.903229	no
Abl2	4.33057	5.11933	0.241398	0.1051	0.201302	no
Ablim1	184.141	196.241	0.091812	0.54085	0.690225	no
Ablim2	1.81211	2.51291	0.471688	0.02895	0.0684975	no
Abr	39.7491	36.5154	-0.122416	0.38835	0.547542	no
Abracl	467.094	382.952	-0.28655	0.04305	0.0958625	no
Abt1	8.43313	7.73081	-0.12545	0.4237	0.58207	no
Abtb1	31.4732	31.1394	-0.0153837	0.9225	0.957904	no
Abtb2	29.6266	23.0721	-0.360747	0.01215	0.0324285	yes
Acaa1a	44.2082	44.2516	0.00141582	0.99155	0.994877	no
Acaa2	40.1163	35.9674	-0.157499	0.30135	0.453854	no
Acaca	5.08921	5.06482	-0.00692885	0.96575	0.981322	no
Acad10	1.59851	1.83046	0.195478	0.40305	0.561993	no
Acad11	4.09558	4.51129	0.139473	0.44825	0.605658	no
Acad8	7.78921	8.57714	0.139021	0.411	0.569914	no
Acad9	8.21922	7.96208	-0.0458567	0.7751	0.867419	no
Acadl	55.7068	59.1886	0.0874667	0.5376	0.687582	no
Acadm	24.874	29.5881	0.250383	0.1008	0.194544	no
Acads	24.0786	22.3526	-0.107307	0.48635	0.641553	no
Acadsb	4.29273	4.26365	-0.00980612	0.95485	0.975894	no
Acadvl	35.06	32.5719	-0.106195	0.4688	0.625462	no
Acap1	216.771	204.817	-0.081832	0.57015	0.714822	no
Acap2	24.3643	22.0083	-0.146722	0.3973	0.556393	no
Acap3	21.6291	23.2355	0.10336	0.47335	0.629783	no
Acat1	18.2156	19.1572	0.0727047	0.6219	0.757279	no
Acat2	9.60076	12.3909	0.368061	0.0305	0.071539	no
Acat3	13.5829	14.9007	0.133586	0.7642	0.860034	no
Acbd3	21.6117	21.477	-0.00901766	0.95035	0.973324	no
Acbd4	8.05969	7.7646	-0.0538134	0.7691	0.863264	no
Acbd5	13.6328	12.8216	-0.0885088	0.56395	0.709734	no
Acbd6	39.6401	37.9059	-0.0645386	0.68095	0.802451	no
Accs	3.84692	3.41663	-0.171126	0.3512	0.508543	no
Acd	35.4852	34.5047	-0.040424	0.87675	0.930946	no
Acer3	2.10121	1.81917	-0.207938	0.3306	0.486285	no
Acin1	94.3666	95.1428	0.0118187	0.9408	0.967968	no
Acly	83.3148	78.2166	-0.0910983	0.5143	0.66715	no
Acn9	4.61579	5.19541	0.17066	0.64245	0.773482	no
Aco1	11.3982	11.6728	0.0343516	0.82185	0.897384	no
Aco2	70.2274	67.1585	-0.0644626	0.64725	0.77722	no
Acot11	4.3773	3.29033	-0.411807	0.01605	0.0411688	yes
Acot13	36.7228	39.2386	0.0955986	0.59045	0.731926	no
Acot2	5.69529	7.04304	0.30643	0.10365	0.199048	no
Acot7	99.2028	121.3	0.290132	0.04335	0.0964211	no
Acot8	25.4136	23.6827	-0.101771	0.54565	0.694167	no
Acot9	46.2258	44.1263	-0.0670595	0.65835	0.785763	no
Acox1	13.2801	11.2672	-0.237136	0.223	0.364981	no
Acox3	15.1077	14.7565	-0.0339351	0.82055	0.896589	no
Acp1	15.1342	14.7351	-0.0385564	0.8016	0.884717	no
Acp2	3.24369	3.70469	0.191717	0.54005	0.689603	no
Acp5	193.707	178.24	-0.120054	0.38975	0.54888	no
Acp6	9.26145	9.99937	0.1106	0.5386	0.688287	no
Acpp	0.837909	1.11987	0.41846	0.12865	0.237055	no
Acrbp	7.29262	7.60341	0.0602104	0.7777	0.86913	no
Acsbg1	54.9751	65.9338	0.262239	0.06415	0.133829	no
Acsf2	6.57195	6.97841	0.0865764	0.6207	0.756432	no
Acsf3	5.45376	6.21274	0.18798	0.32505	0.480244	no
Acsl4	21.454	20.9304	-0.0356462	0.8052	0.887012	no

Acsl5	58.331	56.665	-0.0418049	0.7665	0.861524	no
Acss1	29.333	29.3116	-0.00105242	0.9948	0.996551	no
Acss2	51.0866	36.9305	-0.468134	0.0012	0.00423153	yes
Actb	2799.83	2312.89	-0.275643	0.1879	0.320208	no
Actg1	2442.13	2224.57	-0.134612	0.5056	0.659562	no
Actl6a	36.4387	34.4329	-0.081684	0.5787	0.722117	no
Actn1	41.5732	83.9081	1.01316	5.00E-05	0.000236281	yes
Actn2	2.09469	4.6135	1.13913	5.00E-05	0.000236281	yes
Actn4	106.06	94.988	-0.159063	0.2671	0.414458	no
Actr10	24.9132	25.1683	0.0146981	0.9158	0.953785	no
Actr1a	77.729	69.3372	-0.164823	0.2438	0.388712	no
Actr1b	35.3246	34.061	-0.0525535	0.724	0.832541	no
Actr2	223.986	212.305	-0.0772705	0.59765	0.737871	no
Actr3	734.031	683.295	-0.103334	0.51645	0.669238	no
Actr5	15.1019	15.162	0.00572689	0.97075	0.984008	no
Actr6	11.5638	11.8498	0.0352487	0.8448	0.911458	no
Actr8	21.434	20.7672	-0.0455899	0.76535	0.860738	no
Acvr1b	2.71913	3.53508	0.378599	0.0604	0.127596	no
Acy1	13.729	15.6262	0.186738	0.2806	0.430363	no
Acyp1	23.9245	26.406	0.142381	0.45515	0.612858	no
Acyp2	2.09063	1.90594	-0.133438	0.76205	0.858559	no
Ada	3.31729	3.2327	-0.0372663	0.8767	0.930935	no
Adal	5.66127	5.72823	0.0169631	0.9444	0.969834	no
Adam10	44.7283	43.4924	-0.0404244	0.7772	0.868861	no
Adam15	2.93934	3.30536	0.169316	0.40725	0.56606	no
Adam17	35.6691	30.6949	-0.216675	0.16795	0.29264	no
Adam19	49.5868	75.4729	0.606002	5.00E-05	0.000236281	yes
Adam8	16.9139	24.9599	0.561401	2.00E-04	0.0008488	yes
Adam9	3.81935	3.83027	0.00411858	0.9825	0.989933	no
Adamts10	82.9134	80.4215	-0.0440238	0.758	0.855758	no
Adamts13	26.8367	10.1954	-1.39629	5.00E-05	0.000236281	yes
Adamts14	5.50986	5.31245	-0.0526376	0.7483	0.8494	no
Adamts6	3.28796	3.55402	0.11226	0.5293	0.680443	no
Adap1	17.8444	17.7097	-0.0109374	0.94145	0.968284	no
Adar	30.9483	32.0296	0.049543	0.7252	0.833364	no
Adarb2	1.08198	1.03372	-0.0658347	0.79435	0.880156	no
Adat1	2.63244	2.32881	-0.176805	0.42375	0.582093	no
Adat2	4.52705	4.36433	-0.052812	0.8832	0.934631	no
Adat3	5.5691	6.6013	0.245305	0.6099	0.747568	no
Adck1	7.90468	8.8626	0.165022	0.34915	0.506457	no
Adck2	13.7592	14.4773	0.0733988	0.62835	0.762306	no
Adck3	2.25418	2.41375	0.0986695	0.6388	0.770718	no
Adck4	9.60803	11.9648	0.316487	0.05975	0.126489	no
Adck5	4.98585	5.06131	0.0216726	0.9713	0.984255	no
Adcy3	13.9923	15.2271	0.122009	0.5182	0.670607	no
Adcy6	0.450844	1.03391	1.19742	5.00E-05	0.000236281	yes
Adcy7	115.996	123.185	0.0867551	0.5496	0.697721	no
Add1	93.6737	101.905	0.121506	0.38895	0.548082	no
Add3	105.006	101.035	-0.0556089	0.7014	0.816826	no
Adh5	69.146	71.6273	0.0508648	0.72415	0.832646	no
Adi1	14.1321	16.0477	0.183393	0.27075	0.418667	no
Adipor1	64.2827	63.7643	-0.0116812	0.93145	0.962557	no
Adipor2	30.1578	34.8254	0.207606	0.148	0.264272	no
Adk	6.07461	8.65112	0.510094	0.0106	0.0288545	yes
Adnp	27.9394	27.3812	-0.0291139	0.8393	0.907885	no
Adnp2	8.24247	8.18135	-0.010738	0.9438	0.969593	no
Ado	4.77495	4.71082	-0.0195048	0.908	0.949374	no

Adora2a	39.2822	37.8246	-0.0545476	0.70635	0.820533	no
Adpgk	11.1104	15.1045	0.443071	0.00665	0.0191807	yes
Adprh	85.0958	77.0689	-0.142938	0.32155	0.476419	no
Adprhl2	22.7577	22.5044	-0.0161514	0.91785	0.955019	no
Adprm	25.3497	22.2738	-0.186618	0.24785	0.3931	no
Adrb1	2.6297	1.40958	-0.899635	0.001	0.00359708	yes
Adrb2	15.753	13.546	-0.217765	0.17465	0.301855	no
Adrbk1	276.119	283.279	0.0369376	0.8002	0.883821	no
Adrm1	67.6583	64.0093	-0.0799846	0.58195	0.724903	no
Adsl	18.1415	18.9143	0.0601824	0.68415	0.804851	no
Adss	53.9159	53.5898	-0.00875256	0.94955	0.972844	no
Aebp2	95.4212	97.1489	0.0258878	0.90255	0.946129	no
Aen	11.4119	15.2974	0.422755	0.0071	0.0203241	yes
Aes	454.833	428.995	-0.084376	0.5541	0.701528	no
Aff1	10.6652	9.85649	-0.11377	0.43495	0.593081	no
Aff3	5.3951	10.5643	0.96947	5.00E-05	0.000236281	yes
Aff4	10.8036	11.7296	0.118636	0.4091	0.568128	no
Afg3l1	19.1316	17.8568	-0.0994898	0.493	0.647746	no
Afg3l2	23.3866	26.5338	0.182144	0.2134	0.353357	no
Afmid	19.7488	17.476	-0.176386	0.35485	0.512451	no
Afp	1.22214	2.08873	0.773221	0.0092	0.0255019	yes
Aftph	36.8972	37.1304	0.00909019	0.9498	0.972973	no
Aga	13.5396	14.6573	0.114434	0.51665	0.669362	no
Agap2	37.6662	38.916	0.047093	0.7367	0.841578	no
Agap3	16.6367	16.1226	-0.0452867	0.7638	0.859817	no
Agbl5	5.37009	5.26199	-0.0293387	0.92905	0.961217	no
Ager	2.63159	2.1887	-0.26586	0.34925	0.506571	no
Agfg1	43.3301	52.0154	0.263569	0.062	0.130307	no
Agfg2	25.9663	20.1998	-0.362301	0.0169	0.0430564	yes
Aggf1	31.5746	29.9111	-0.0780797	0.5899	0.731566	no
Agk	9.8477	10.8521	0.140119	0.4058	0.564823	no
Agl	1.445	1.25585	-0.202413	0.28535	0.435651	no
Ago1	9.63022	9.16582	-0.0713037	0.62865	0.762539	no
Ago2	16.2052	17.6304	0.12161	0.3902	0.549295	no
Ago3	2.4939	2.98608	0.259851	0.12615	0.233362	no
Agpat1	45.5935	47.3422	0.0542998	0.70525	0.819674	no
Agpat2	8.18923	8.26474	0.013242	0.94845	0.972239	no
Agpat3	51.5935	46.1391	-0.161199	0.25465	0.3993	no
Agpat4	15.7781	17.7972	0.173726	0.2872	0.437746	no
Agpat5	5.0125	6.34802	0.340779	0.04725	0.103671	no
Agpat6	16.5902	16.8722	0.0243172	0.8649	0.923867	no
Agps	13.3712	15.2748	0.192027	0.18015	0.309534	no
Agrn	1.16171	1.39236	0.261285	0.2026	0.339448	no
Agtpbp1	10.3169	11.3137	0.133055	0.3919	0.550996	no
Agtr1a	4.74558	0	#NAME?	5.00E-05	0.000236281	yes
Agtrap	13.7565	16.5271	0.264716	0.0761	0.154033	no
Ahctf1	14.2872	15.4359	0.11157	0.4319	0.590085	no
Ahcy	53.3106	62.0535	0.219089	0.12	0.22423	no
Ahcyl1	26.5128	24.2101	-0.131077	0.3678	0.526306	no
Ahcyl2	11.9263	12.4152	0.057953	0.6962	0.813455	no
Ahdc1	8.6786	9.32266	0.10328	0.48615	0.641373	no
Ahi1	2.99412	2.9163	-0.0379911	0.8383	0.907358	no
Ahnak	242.67	241.111	-0.00929626	0.9625	0.979895	no
Ahsa1	97.4636	89.4283	-0.124132	0.38205	0.541114	no
Ahsa2	22.6897	22.1661	-0.0336783	0.911	0.951116	no
AI182371	1.02227	0.668426	-0.61293	0.13055	0.239873	no
AI314180	16.3142	16.1992	-0.0102089	0.94435	0.969811	no

AI413582	84.4926	82.702	-0.0309034	0.83835	0.907358	no
AI462493	66.2154	70.5842	0.0921792	0.5471	0.695548	no
AI467606	71.6125	75.3199	0.0728181	0.6059	0.74441	no
AI597479	8.60187	7.64932	-0.169318	0.30005	0.452379	no
AI837181	34.9878	35.7265	0.0301434	0.8376	0.906977	no
AI846148	9.1966	9.87389	0.102518	0.5717	0.716142	no
AI987944	7.63707	7.37138	-0.0510846	0.76805	0.862565	no
Aifm1	16.8607	14.7983	-0.18824	0.231	0.374419	no
Aifm2	1.03543	1.14366	0.143434	0.6734	0.79684	no
Aig1	3.05318	3.53919	0.213107	0.46895	0.625592	no
Aim1	31.5816	28.6051	-0.142816	0.3146	0.468422	no
Aim1l	2.1258	1.74834	-0.282024	0.17065	0.296429	no
Aim2	7.12068	7.90155	0.150121	0.3946	0.553843	no
Aimp1	78.7034	70.0964	-0.167085	0.25065	0.396277	no
Aimp2	23.5548	22.2928	-0.0794462	0.81415	0.892456	no
Aip	78.669	71.674	-0.134345	0.60825	0.746168	no
AK010878	12.8489	14.7637	0.200403	0.5138	0.666686	no
Ak2	67.5544	70.1751	0.0549083	0.71505	0.826464	no
Ak3	55.4584	42.6029	-0.380456	0.00955	0.0263592	yes
Ak6	14.3082	15.3486	0.101268	0.7707	0.864414	no
Akap1	1.65911	2.27832	0.457555	0.03905	0.0882077	no
Akap10	16.1476	16.3802	0.0206381	0.88965	0.938235	no
Akap11	6.54071	6.67232	0.0287398	0.8439	0.91092	no
Akap12	2.6292	2.29402	-0.196745	0.2805	0.430285	no
Akap13	50.7598	60.0578	0.242666	0.0939	0.183495	no
Akap8	65.3101	67.4107	0.0456714	0.74415	0.846718	no
Akap8l	43.5477	42.1686	-0.0464282	0.7532	0.852466	no
Akap9	9.34298	11.364	0.282517	0.0467	0.102656	no
Akip1	18.3634	17.4373	-0.0746597	0.68625	0.806301	no
Akirin1	58.0979	54.2142	-0.0998158	0.48135	0.636993	no
Akirin2	31.1379	29.9566	-0.0557979	0.8037	0.886148	no
Akna	153.689	169.658	0.142622	0.32995	0.485623	no
Akr1a1	214.495	201.559	-0.0897417	0.5292	0.680352	no
Akr1b10	13.8651	13.0558	-0.0867697	0.62765	0.761823	no
Akr1b3	22.318	22.1159	-0.0131238	0.97245	0.98484	no
Akr1c13	21.799	26.8399	0.300115	0.0685	0.141242	no
Akr1e1	3.38893	3.37918	-0.00415501	0.9878	0.992906	no
Akr7a5	6.04772	8.31745	0.459749	0.02095	0.0519313	no
Akt1	94.0435	92.3773	-0.0257897	0.8575	0.919469	no
Akt1s1	9.06378	10.3954	0.197755	0.2855	0.435823	no
Akt2	71.1862	60.0876	-0.244531	0.0849	0.168834	no
Akt3	6.6965	6.23427	-0.103185	0.5207	0.672757	no
Aktip	10.476	8.92653	-0.230922	0.67285	0.79639	no
Alad	5.51579	5.80055	0.0726229	0.6669	0.79207	no
Alas1	10.3237	11.2743	0.127076	0.4387	0.596776	no
Alcam	6.74092	6.78051	0.00844896	0.9574	0.977108	no
Aldh16a1	15.0989	13.6719	-0.143229	0.48915	0.644151	no
Aldh18a1	24.661	24.9209	0.0151297	0.91725	0.954634	no
Aldh1b1	1.33343	1.34502	0.0124931	0.9779	0.987653	no
Aldh3a2	32.635	27.8268	-0.229948	0.10775	0.205546	no
Aldh4a1	9.91828	9.81673	-0.014848	0.92465	0.958829	no
Aldh6a1	1.68128	1.92636	0.196319	0.6433	0.774162	no
Aldh7a1	3.78882	2.96801	-0.352253	0.3563	0.514113	no
Aldh9a1	26.4861	27.9137	0.0757347	0.60145	0.741046	no
Aldoa	263.757	265.33	0.00857664	0.95055	0.973444	no
Aldoc	1.45919	2.01814	0.467853	0.12495	0.231549	no
Alg1	14.1159	15.3534	0.121241	0.57735	0.720993	no

Alg10b	18.425	18.2694	-0.0122329	0.9361	0.965285	no
Alg11	2.60299	2.5361	-0.0375584	0.8417	0.909559	no
Alg12	15.5645	15.6176	0.00491341	0.9766	0.987018	no
Alg13	3.78126	4.38136	0.212513	0.34965	0.506995	no
Alg14	4.91078	3.85871	-0.347836	0.22145	0.363041	no
Alg2	3.87563	4.02199	0.0534805	0.77695	0.86869	no
Alg3	11.5041	11.0724	-0.0551781	0.76605	0.86121	no
Alg5	31.9976	32.3096	0.0140007	0.92555	0.959382	no
Alg6	2.80064	2.23831	-0.323346	0.13685	0.249011	no
Alg8	7.54389	6.44967	-0.226084	0.2305	0.373978	no
Alg9	6.32196	6.33224	0.00234326	0.98945	0.993716	no
Alkbh1	15.4289	15.7967	0.0339935	0.92105	0.957064	no
Alkbh2	5.68727	5.28524	-0.105769	0.6703	0.79467	no
Alkbh3	19.7471	20.9962	0.0884909	0.6088	0.746674	no
Alkbh4	15.9291	16.1957	0.0239499	0.88255	0.934265	no
Alkbh5	23.7138	24.0343	0.0193667	0.89375	0.94062	no
Alkbh6	15.9365	16.9242	0.0867574	0.63865	0.770603	no
Alkbh7	9.78168	10.8777	0.153214	0.47695	0.633093	no
Alkbh8	6.75094	7.54447	0.16033	0.38095	0.539913	no
Alms1	1.08111	0.706492	-0.613767	0.00385	0.0118893	yes
Als2	5.6534	5.64529	-0.00207083	0.99055	0.994241	no
Als2cl	1.16252	0.515385	-1.17353	1.00E-04	0.000450026	yes
Alyref	62.9918	56.3848	-0.159858	0.2826	0.432556	no
Alyref2	8.71649	7.83934	-0.153015	0.45685	0.614362	no
Ambra1	16.9245	16.321	-0.0523841	0.7159	0.826999	no
Amd1	3.43043	3.25377	-0.076276	0.91805	0.95517	no
Amd2	8.141	9.07011	0.155913	0.777	0.868719	no
Amdhd2	22.8806	22.9805	0.0062849	0.96895	0.983186	no
Amfr	52.0395	50.6917	-0.0378578	0.78625	0.874889	no
Amica1	34.6405	42.417	0.292183	0.06325	0.132284	no
Amigo1	1.21234	1.06133	-0.191913	0.3956	0.554695	no
Amigo3	3.47028	3.81637	0.137151	0.8263	0.900293	no
Ammecr1	3.75332	3.97328	0.0821643	0.6702	0.794599	no
Ammecr1l	11.6524	11.2813	-0.0466951	0.7559	0.854385	no
Amn1	4.96496	4.87086	-0.0276047	0.9195	0.956073	no
Ampd2	16.3292	17.0945	0.0660843	0.65445	0.782802	no
Amz2	9.37623	10.4715	0.159388	0.32415	0.479255	no
Anapc1	21.5083	22.0635	0.0367717	0.79785	0.882289	no
Anapc10	5.62756	4.84158	-0.217031	0.2415	0.386065	no
Anapc11	19.1092	18.4859	-0.0478412	0.74265	0.845624	no
Anapc13	114.278	102.442	-0.157734	0.35525	0.512902	no
Anapc15	28.2497	22.672	-0.317323	0.0745	0.151281	no
Anapc16	43.7417	39.5096	-0.146805	0.41625	0.574879	no
Anapc2	41.2577	38.5449	-0.0981223	0.49015	0.645155	no
Anapc4	29.0201	28.0569	-0.0486954	0.73435	0.839777	no
Anapc5	98.649	80.9025	-0.286121	0.04515	0.0997989	no
Anapc7	22.0312	19.7397	-0.158445	0.2911	0.442049	no
Angel1	2.17463	3.01034	0.469159	0.02195	0.054091	no
Angel2	30.8811	31.3965	0.0238788	0.8712	0.927663	no
Angptl4	1.90551	2.00115	0.070653	0.79745	0.882095	no
Angptl6	2.74099	2.81226	0.0370342	0.92945	0.961433	no
Ank	20.1129	20.9436	0.0583841	0.6913	0.809942	no
Ankfy1	17.7229	17.3652	-0.0294159	0.834	0.904856	no
Ankhd1	20.2087	20.6246	0.0293913	0.83455	0.905188	no
Ankib1	21.8116	20.84	-0.0657388	0.6449	0.7754	no
Ankle1	3.91469	3.00622	-0.380947	0.0908	0.178559	no
Ankle2	12.2592	13.5075	0.139899	0.3506	0.507986	no

Ankmy2	14.2039	13.7542	-0.0464123	0.76735	0.862081	no
Ankra2	21.0273	18.0314	-0.221748	0.1619	0.284288	no
Ankrd10	60.6975	60.726	0.000676483	0.9951	0.996682	no
Ankrd11	59.402	59.8909	0.0118256	0.9327	0.963294	no
Ankrd12	14.8994	18.166	0.285995	0.04195	0.0937532	no
Ankrd13a	97.5172	105.862	0.118457	0.4059	0.564884	no
Ankrd13b	1.72467	2.39323	0.472636	0.04105	0.0920157	no
Ankrd13c	21.2727	20.5851	-0.0474061	0.7506	0.850943	no
Ankrd13d	5.58082	7.46587	0.419835	0.02765	0.0659032	no
Ankrd16	13.2696	12.5185	-0.0840646	0.6033	0.742402	no
Ankrd17	19.4312	20.2351	0.0584848	0.68305	0.803852	no
Ankrd23	8.2797	8.61338	0.057001	0.751	0.851219	no
Ankrd26	3.34735	3.14084	-0.091873	0.59895	0.738933	no
Ankrd27	18.8331	18.7771	-0.00429623	0.9752	0.98638	no
Ankrd28	7.49735	7.43673	-0.0117131	0.94105	0.96807	no
Ankrd29	1.10097	0.992557	-0.149554	0.59125	0.732493	no
Ankrd32	5.2802	5.18818	-0.0253631	0.88855	0.937538	no
Ankrd37	3.16226	3.63479	0.200917	0.7116	0.824109	no
Ankrd39	8.92715	9.28606	0.0568663	0.75835	0.856044	no
Ankrd40	29.0163	29.3785	0.0178981	0.90475	0.94752	no
Ankrd44	83.819	82.7882	-0.0178516	0.90125	0.94537	no
Ankrd46	14.2698	14.445	0.0175992	0.90995	0.950442	no
Ankrd49	6.94277	8.67928	0.322064	0.08925	0.176006	no
Ankrd50	6.04476	5.90387	-0.0340248	0.83455	0.905188	no
Ankrd52	20.0711	18.9107	-0.0859148	0.5437	0.692672	no
Ankrd54	27.6573	30.4984	0.141073	0.35625	0.514052	no
Ankrd9	1.65567	1.71035	0.046876	0.86105	0.92154	no
Anks1	7.09917	7.66251	0.110166	0.47295	0.629453	no
Anks3	19.9719	20.2113	0.0171955	0.91045	0.950781	no
Ankzf1	15.7237	18.9174	0.266772	0.18595	0.317444	no
Anln	3.85371	1.9083	-1.01396	5.00E-05	0.000236281	yes
Ano10	16.1989	27.3807	0.757266	5.00E-05	0.000236281	yes
Ano6	20.7325	19.0441	-0.122552	0.38855	0.547682	no
Ano8	18.148	18.5396	0.0307989	0.90085	0.945076	no
Anp32a	147.958	156.885	0.0845196	0.5547	0.701998	no
Anp32b	250.831	214.445	-0.226111	0.11295	0.213452	no
Anp32e	86.4157	72.6081	-0.251162	0.07855	0.158126	no
Antxr2	20.5249	21.594	0.0732575	0.6088	0.746674	no
Anxa1	90.2939	119.982	0.410117	0.00485	0.0145831	yes
Anxa11	86.9275	92.5648	0.0906511	0.5224	0.674299	no
Anxa2	376.618	370.59	-0.0232775	0.8717	0.927887	no
Anxa4	16.8472	9.59658	-0.811918	5.00E-05	0.000236281	yes
Anxa5	53.8285	61.5817	0.194132	0.1775	0.3058	no
Anxa6	778.821	760.934	-0.0335205	0.83695	0.906579	no
Anxa7	44.2874	45.2188	0.0300259	0.82905	0.9018	no
Anxa9	1.29907	1.1326	-0.197834	0.79655	0.881556	no
Aoc2	5.12596	5.67674	0.147241	0.4346	0.592764	no
Ap1ar	29.6867	31.0627	0.0653623	0.65215	0.781071	no
Ap1b1	47.455	48.6679	0.036411	0.80325	0.885873	no
Ap1g1	18.2451	17.9648	-0.0223365	0.8745	0.929494	no
Ap1g2	54.2684	49.9717	-0.119002	0.4613	0.618402	no
Ap1m1	87.138	83.2267	-0.0662561	0.63625	0.76864	no
Ap1s1	47.3155	44.1742	-0.0991099	0.5131	0.666169	no
Ap1s2	38.6854	33.211	-0.220125	0.13375	0.244581	no
Ap1s3	10.938	12.1848	0.155742	0.3221	0.477003	no
Ap2a1	34.3162	34.1519	-0.0069246	0.964	0.980433	no
Ap2a2	21.1756	20.7329	-0.0304862	0.83445	0.905176	no

Ap2b1	47.374	45.1322	-0.0699403	0.616	0.752796	no
Ap2m1	159.055	147.645	-0.107394	0.45635	0.613876	no
Ap2s1	211.146	188.713	-0.162043	0.2582	0.403612	no
Ap3b1	37.4897	36.3793	-0.0433778	0.75395	0.853001	no
Ap3d1	56.5663	56.9036	0.00857686	0.95335	0.975024	no
Ap3m1	16.6928	15.3442	-0.121529	0.4054	0.564389	no
Ap3m2	15.5777	16.9411	0.121052	0.41795	0.576561	no
Ap3s1	65.3703	69.4848	0.0880612	0.53995	0.689513	no
Ap3s2	8.93638	8.82658	-0.0178359	0.90745	0.949052	no
Ap4b1	16.21	15.1376	-0.0987459	0.51455	0.667389	no
Ap4e1	8.34057	8.19272	-0.025802	0.86685	0.924942	no
Ap4m1	9.50717	10.0655	0.0823275	0.8008	0.884193	no
Ap4s1	24.4481	24.3724	-0.00447771	0.98585	0.991766	no
Ap5b1	6.40779	5.99838	-0.0952535	0.58615	0.728348	no
Ap5m1	18.2346	15.9747	-0.190884	0.43365	0.591891	no
Ap5s1	8.11366	8.41632	0.0528379	0.78955	0.877137	no
Ap5z1	16.2453	16.9231	0.0589733	0.6929	0.81109	no
Apaf1	23.0015	20.6044	-0.158774	0.2619	0.408058	no
Apba3	11.7907	12.6561	0.102183	0.6113	0.748686	no
Apbb1	2.52367	2.16921	-0.218351	0.33875	0.495137	no
Apbb1ip	156.702	132.787	-0.238913	0.0914	0.179479	no
Apbb3	13.0103	12.186	-0.09443	0.75225	0.851938	no
Apc	9.36992	9.74466	0.0565739	0.69425	0.811997	no
Apeh	51.7854	54.1511	0.0644461	0.65365	0.782283	no
Apex1	3.99073	5.49295	0.46093	0.2875	0.438043	no
Apex2	7.48731	6.90836	-0.116104	0.53815	0.687986	no
Aph1a	42.2214	39.4815	-0.0967991	0.50395	0.65803	no
Api5	48.1059	44.8865	-0.0999298	0.47375	0.630149	no
Apip	8.93305	10.497	0.23275	0.29375	0.445153	no
Apitd1	7.62829	4.38672	-0.798218	0.00265	0.0085599	yes
Aplf	2.36494	1.76793	-0.419746	0.0679	0.140173	no
Aplp1	2.81283	2.61043	-0.107733	0.6409	0.772222	no
Aplp2	36.2013	36.5696	0.0146057	0.9197	0.956211	no
Apmap	26.5708	24.9618	-0.0901162	0.55235	0.700016	no
Apoa1bp	41.5214	38.1024	-0.123972	0.44875	0.606137	no
Apoa2	1.32471	0.537444	-1.30149	0.136	0.247707	no
Apobec1	3.814	3.21871	-0.24482	0.257	0.402119	no
Apobec2	29.8372	14.8298	-1.00861	5.00E-05	0.000236281	yes
Apobec3	94.4338	104.502	0.146152	0.3039	0.456711	no
Apobr	17.0122	11.655	-0.54562	3.00E-04	0.00122185	yes
Apoe	1.7707	2.40695	0.442892	0.1707	0.296487	no
Apol8	1.2925	1.66146	0.362293	0.24655	0.391725	no
Apoo	7.04058	7.57494	0.105539	0.6919	0.810403	no
Apool	17.8094	17.6387	-0.0138915	0.9408	0.967968	no
Apopt1	37.5779	40.277	0.10007	0.5401	0.689629	no
App	0.605287	2.79456	2.20693	5.00E-05	0.000236281	yes
Appbp2	15.0559	16.0487	0.0921268	0.5629	0.708779	no
Appl1	17.967	19.4674	0.11571	0.41125	0.57016	no
Appl2	11.6069	18.791	0.695056	5.00E-05	0.000236281	yes
Aprt	90.3067	89.9631	-0.0054993	0.9688	0.98309	no
Aptx	3.01941	2.95915	-0.0290842	0.8724	0.928391	no
Aqp3	8.57153	16.5319	0.947632	5.00E-05	0.000236281	yes
Aqp9	5.60765	4.9419	-0.18233	0.33135	0.487033	no
Aqr	45.4105	40.8868	-0.15139	0.2806	0.430363	no
Ar	1.41132	2.12293	0.589014	0.0187	0.0470228	yes
Araf	54.5392	55.6721	0.0296607	0.8447	0.911421	no
Arap1	26.644	27.7308	0.0576765	0.6844	0.805052	no

Arap2	29.4133	25.0974	-0.228932	0.10725	0.204714	no
Arap3	1.63786	2.35429	0.523481	0.00915	0.0253792	yes
Arc	4.06918	2.85463	-0.511435	0.01105	0.0299157	yes
Arcn1	67.9111	66.7087	-0.0257722	0.8573	0.919339	no
Arel1	21.9026	21.7931	-0.00723361	0.9595	0.978196	no
Arf1	253.673	235.642	-0.106372	0.452	0.609477	no
Arf2	8.26639	7.9432	-0.0575366	0.7395	0.843398	no
Arf3	64.0445	67.8027	0.0822699	0.55885	0.705605	no
Arf4	67.3775	60.3718	-0.158394	0.268	0.415462	no
Arf5	204.487	196.16	-0.0599782	0.6637	0.789638	no
Arf6	150.025	129.02	-0.217609	0.1285	0.236821	no
Arfgap1	24.2907	23.4391	-0.0514871	0.7278	0.835088	no
Arfgap2	40.3346	41.4115	0.0380153	0.79035	0.877736	no
Arfgap3	5.97685	5.63553	-0.0848337	0.64555	0.775838	no
Arfgef1	29.5295	32.3872	0.133269	0.34305	0.499828	no
Arfgef2	11.3583	11.2334	-0.0159533	0.90825	0.949439	no
Arfip1	19.5227	17.7256	-0.13932	0.3546	0.512163	no
Arfip2	12.4265	12.9902	0.0640057	0.6805	0.802095	no
Arfrp1	32.4181	29.3325	-0.144304	0.54895	0.697121	no
Arglu1	84.227	86.2468	0.0341882	0.8058	0.887382	no
Arhgap1	57.3422	51.5194	-0.154481	0.27445	0.423119	no
Arhgap10	12.8505	12.1485	-0.0810402	0.6123	0.749586	no
Arhgap11a	13.566	9.56308	-0.50445	0.00135	0.00470245	yes
Arhgap12	8.01503	7.95379	-0.0110661	0.9404	0.967782	no
Arhgap15	60.8133	66.3989	0.126772	0.3694	0.527975	no
Arhgap17	26.1589	21.062	-0.31266	0.03185	0.0742245	no
Arhgap18	13.6562	12.8014	-0.0932481	0.5381	0.687959	no
Arhgap19	10.9287	6.55285	-0.737934	5.00E-05	0.000236281	yes
Arhgap21	1.23439	2.66694	1.11138	5.00E-05	0.000236281	yes
Arhgap25	45.6786	41.1378	-0.151052	0.28385	0.433971	no
Arhgap26	17.2397	16.7482	-0.0417343	0.7693	0.86342	no
Arhgap27	40.7704	43.308	0.0871119	0.5733	0.717548	no
Arhgap30	158.344	157.689	-0.00597125	0.96775	0.982463	no
Arhgap31	5.51424	5.67135	0.0405315	0.79195	0.878683	no
Arhgap33	2.98304	1.98188	-0.589913	0.00305	0.00969839	yes
Arhgap39	1.83348	1.80533	-0.0223226	0.91605	0.953947	no
Arhgap4	176.389	173.735	-0.0218719	0.8798	0.932627	no
Arhgap5	0.823577	1.02771	0.319465	0.19935	0.335292	no
Arhgap9	184.145	188.874	0.0365811	0.79615	0.881293	no
Arhgdia	289.589	279.64	-0.0504338	0.7255	0.8336	no
Arhgdib	1603.95	1519.43	-0.0781001	0.6163	0.753033	no
Arhgef1	457.407	444.236	-0.0421516	0.7837	0.873167	no
Arhgef11	1.22098	1.3289	0.12219	0.6095	0.747259	no
Arhgef12	4.77491	5.57267	0.222894	0.1431	0.257253	no
Arhgef18	79.224	74.6718	-0.0853727	0.5432	0.692323	no
Arhgef2	87.7429	84.8392	-0.0485519	0.73255	0.838548	no
Arhgef25	0.839682	1.0238	0.286022	0.39615	0.555146	no
Arhgef3	113.666	101.623	-0.161574	0.26415	0.410856	no
Arhgef39	2.22047	2.7669	0.31741	0.2441	0.389085	no
Arhgef4	11.4543	9.21816	-0.313337	0.05905	0.125199	no
Arhgef6	57.7761	54.6214	-0.0810072	0.565	0.710587	no
Arhgef7	16.5769	15.7521	-0.0736302	0.6174	0.75392	no
Arid1a	63.6087	70.2341	0.142947	0.314	0.467796	no
Arid1b	9.06883	9.37996	0.048666	0.73075	0.837264	no
Arid2	10.6711	12.8536	0.26846	0.0615	0.129452	no
Arid3a	4.56626	4.62671	0.0189737	0.90545	0.947916	no
Arid3b	18.8364	18.8323	-0.000315212	0.9985	0.998741	no

Arid4a	21.9104	23.0268	0.071698	0.6188	0.754963	no
Arid4b	16.9301	19.4825	0.202589	0.15595	0.275774	no
Arid5a	23.1054	25.1189	0.120547	0.4028	0.5618	no
Arid5b	8.58706	9.31563	0.11749	0.4432	0.600986	no
Arih1	32.3569	31.1389	-0.0553524	0.7021	0.817251	no
Arih2	30.4933	30.7939	0.0141526	0.9244	0.958767	no
Arl1	62.5398	54.6475	-0.194621	0.2076	0.345879	no
Arl10	3.00992	4.148	0.46269	0.10915	0.207689	no
Arl13b	4.72015	5.35304	0.181528	0.3006	0.453024	no
Arl14ep	6.83255	7.00201	0.0353439	0.8357	0.905837	no
Arl15	5.66258	4.00026	-0.501365	0.00675	0.0194405	yes
Arl16	7.70405	7.06763	-0.124392	0.6468	0.776878	no
Arl2	8.18744	7.14462	-0.196555	0.42265	0.581057	no
Arl2bp	109.046	96.4153	-0.1776	0.20455	0.341879	no
Arl3	17.4139	16.7799	-0.0535045	0.7811	0.87126	no
Arl4a	11.1293	11.0626	-0.00867187	0.954	0.975307	no
Arl4c	240.564	234.074	-0.0394562	0.79045	0.877778	no
Arl4d	51.1141	57.202	0.162344	0.2618	0.407956	no
Arl5a	29.7036	23.2157	-0.355539	0.0124	0.0330034	yes
Arl5b	3.39307	3.56904	0.072944	0.70125	0.816773	no
Arl5c	90.4415	77.8502	-0.216283	0.1284	0.236668	no
Arl6	1.02807	1.52532	0.569167	0.117	0.219724	no
Arl6ip1	213.087	200.689	-0.0864791	0.54065	0.690082	no
Arl6ip4	50.3902	48.2065	-0.0639159	0.6718	0.795654	no
Arl6ip5	441.085	394.832	-0.159817	0.26955	0.417315	no
Arl6ip6	10.3726	10.1255	-0.0347799	0.83085	0.902951	no
Arl8a	32.7285	31.6703	-0.0474197	0.7546	0.853503	no
Arl8b	25.3488	22.9558	-0.143058	0.3283	0.48382	no
Armc1	19.341	17.1514	-0.173333	0.2472	0.392422	no
Armc10	54.1712	49.1713	-0.139709	0.3342	0.490161	no
Armc3	1.95884	1.65149	-0.246231	0.3074	0.460598	no
Armc5	15.3618	15.1475	-0.0202681	0.8976	0.943223	no
Armc6	5.47844	4.59633	-0.253285	0.5223	0.674207	no
Armc7	120.737	95.4173	-0.339545	0.04025	0.0905157	no
Armc8	11.5624	11.6066	0.00550363	0.97105	0.984157	no
Armc9	1.10879	1.40327	0.339801	0.26625	0.413348	no
Armcx2	1.66846	2.07899	0.317367	0.162	0.284442	no
Armcx3	1.92355	1.93119	0.00571882	0.9879	0.992965	no
Armcx5	6.54577	6.43088	-0.0255472	0.91225	0.9519	no
Arnt	14.851	14.4182	-0.0426769	0.7758	0.867913	no
Arntl	19.9385	20.9191	0.0692622	0.641	0.77229	no
Arpc1a	57.7624	70.2283	0.281921	0.0529	0.114005	no
Arpc1b	622.909	564.286	-0.142596	0.3346	0.490544	no
Arpc2	685.417	636.097	-0.107734	0.46495	0.62196	no
Arpc3	511.308	495.171	-0.0462678	0.74465	0.847027	no
Arpc4	170.371	162.607	-0.0672899	0.64555	0.775838	no
Arpc5	384.126	351.839	-0.126667	0.3759	0.534645	no
Arpc5l	115.032	92.7191	-0.3111	0.02985	0.0702924	no
Arpp19	19.3972	18.481	-0.0698092	0.62175	0.757188	no
Arrb1	1.83343	1.79566	-0.030027	0.8723	0.92833	no
Arrb2	184.981	207.221	0.163795	0.2562	0.401152	no
Arrdc1	89.454	84.7279	-0.0783092	0.57355	0.717733	no
Arrdc2	9.69915	10.24	0.0782866	0.63575	0.768246	no
Arrdc3	17.144	20.1329	0.231849	0.11065	0.210056	no
Arsa	6.61223	6.99362	0.0809028	0.63305	0.766217	no
Arsb	50.841	37.5852	-0.435828	0.00225	0.00740543	yes
Arsk	3.294	3.6563	0.150541	0.4194	0.577929	no

Art2a-ps	1.22784	0.40598	-1.59664	0.0139	0.0363906	yes
Art2b	13.4022	5.23785	-1.35543	5.00E-05	0.000236281	yes
Arv1	5.58109	6.46427	0.21194	0.36505	0.523422	no
Arxes2	0.879933	1.45717	0.727705	0.0528	0.113832	no
As3mt	40.6145	10.4068	-1.96447	5.00E-05	0.000236281	yes
Asah1	14.257	13.3278	-0.0972365	0.5489	0.697083	no
Asap1	17.4018	16.6595	-0.0628922	0.667	0.792141	no
Asap2	1.68917	0.827109	-1.03016	0.0348	0.0799692	no
Asb1	8.45685	8.15024	-0.053278	0.7231	0.831913	no
Asb13	17.3857	17.9344	0.0448271	0.77075	0.864428	no
Asb3	5.64432	5.68249	0.00972235	0.9567	0.976752	no
Asb6	36.0682	34.851	-0.0495291	0.7373	0.841964	no
Asb7	3.83072	3.73176	-0.0377578	0.831	0.903058	no
Asb8	12.6371	10.7298	-0.236048	0.15565	0.275354	no
Ascc1	18.9166	18.5616	-0.0273338	0.91245	0.952053	no
Ascc2	13.8803	14.5167	0.064673	0.6772	0.799648	no
Ascc3	10.0666	9.84741	-0.0317595	0.82295	0.898044	no
Asf1a	9.08463	8.58056	-0.082355	0.7142	0.825928	no
Asf1b	43.1146	21.199	-1.02418	5.00E-05	0.000236281	yes
Ash1l	8.46528	8.97917	0.085025	0.5526	0.700194	no
Ash2l	17.3791	18.4288	0.0846046	0.56595	0.71135	no
Asl	20.5737	21.7925	0.0830274	0.6048	0.743577	no
Asna1	55.2459	56.0582	0.0210578	0.8891	0.93788	no
Asns	3.58137	3.55616	-0.0101893	0.9667	0.981919	no
Asnsd1	59.5657	53.9834	-0.141966	0.37405	0.53269	no
Aspm	4.25747	2.44309	-0.801285	5.00E-05	0.000236281	yes
Aspscr1	49.9738	45.0874	-0.148447	0.3122	0.465851	no
Asrgl1	27.0066	24.1284	-0.162584	0.2812	0.431012	no
Ass1	36.1793	46.7404	0.369503	0.013	0.0343645	yes
Aste1	6.26086	6.3749	0.0260414	0.899	0.944076	no
Asun	24.9312	29.0489	0.220532	0.1301	0.239233	no
Asxl1	32.3453	31.5898	-0.0341002	0.81165	0.89103	no
Asxl2	13.8372	14.9071	0.107449	0.4476	0.605012	no
Atad1	40.0034	41.0982	0.0389556	0.78925	0.876956	no
Atad2	18.9285	14.0505	-0.429944	0.00235	0.00770045	yes
Atad2b	12.9675	13.55	0.0633894	0.65565	0.783746	no
Atad3a	8.70552	9.82267	0.174184	0.3126	0.466281	no
Atad5	3.35909	2.3464	-0.517619	0.0029	0.00927196	yes
Atat1	3.18972	3.29298	0.0459646	0.85365	0.916957	no
Ate1	11.5245	11.6303	0.0131858	0.9331	0.963486	no
Atf1	40.4182	35.3721	-0.192395	0.18885	0.321462	no
Atf2	16.5776	15.2148	-0.123752	0.3996	0.558606	no
Atf4	233.487	231.313	-0.0135014	0.9243	0.958705	no
Atf5	4.67926	4.24535	-0.140396	0.81835	0.895169	no
Atf6	14.1925	15.4851	0.125749	0.3802	0.539197	no
Atf6b	30.8955	32.3953	0.0683868	0.63505	0.767782	no
Atf7	5.71427	5.07342	-0.171612	0.33905	0.495411	no
Atf7ip	37.7244	36.875	-0.0328556	0.81445	0.892604	no
Atg10	3.19036	3.89991	0.289724	0.24065	0.385698	no
Atg101	30.4492	27.3432	-0.155222	0.335	0.490957	no
Atg12	32.0964	31.4388	-0.0298673	0.8378	0.907041	no
Atg13	15.5729	14.6727	-0.085906	0.569	0.713824	no
Atg14	3.99551	4.80868	0.267263	0.14035	0.253569	no
Atg16l1	20.3495	19.6629	-0.0495189	0.74005	0.843712	no
Atg16l2	30.2529	31.5781	0.0618513	0.671	0.795147	no
Atg2a	18.0072	17.4386	-0.0462891	0.74705	0.848622	no
Atg2b	7.29046	8.32909	0.19215	0.18085	0.310487	no

Atg3	43.7603	41.7579	-0.067574	0.6512	0.780304	no
Atg4a	13.678	12.5984	-0.118621	0.4689	0.625537	no
Atg4b	17.933	17.5169	-0.0338662	0.82335	0.898272	no
Atg4d	25.3452	18.3933	-0.46253	0.05235	0.113017	no
Atg5	28.6331	27.8168	-0.0417275	0.77515	0.867461	no
Atg7	16.1661	14.7829	-0.129042	0.3886	0.547742	no
Atg9a	7.47239	9.13424	0.289716	0.0728	0.148431	no
Athl1	10.1051	10.6155	0.0710844	0.6543	0.782742	no
Atic	45.1946	45.4007	0.00656446	0.96215	0.979794	no
Atl2	8.74491	7.6491	-0.193154	0.2331	0.377069	no
Atl3	22.1627	21.6646	-0.032797	0.8173	0.894617	no
Atm	12.3778	13.4933	0.12449	0.3796	0.538584	no
Atmin	12.1642	11.7741	-0.0470199	0.7511	0.851264	no
Atn1	4.16557	4.83824	0.215969	0.2147	0.354919	no
Atox1	254.516	243.113	-0.0661255	0.6621	0.788625	no
Atp10a	16.0105	17.957	0.165525	0.2467	0.391884	no
Atp10d	22.4067	22.2502	-0.0101132	0.9412	0.968182	no
Atp11a	7.89642	7.45435	-0.0831144	0.57245	0.716763	no
Atp11b	94.5852	97.9389	0.0502666	0.72935	0.836091	no
Atp11c	8.42228	8.24849	-0.0300795	0.83985	0.908243	no
Atp13a1	37.5221	40.6119	0.114165	0.4152	0.573957	no
Atp13a2	14.9994	14.5941	-0.0395231	0.78845	0.876494	no
Atp13a3	25.1451	27.1099	0.10854	0.45125	0.608721	no
Atp1a1	235.867	369.011	0.645689	5.00E-05	0.000236281	yes
Atp1b1	1.28704	0.808176	-0.671311	0.18005	0.309385	no
Atp1b3	556.332	622.729	0.162658	0.2726	0.420846	no
Atp2a1	1.49665	0.882529	-0.762019	0.26435	0.411095	no
Atp2a2	44.3796	46.2662	0.0600615	0.6684	0.793118	no
Atp2a3	210.563	169.738	-0.310939	0.0373	0.0848166	no
Atp2b1	32.5513	36.7214	0.173905	0.217	0.357807	no
Atp2b4	42.7054	45.0039	0.075631	0.5964	0.736804	no
Atp2c1	20.666	18.9996	-0.121289	0.4402	0.598194	no
Atp5a1	333.541	335.002	0.00630636	0.96775	0.982463	no
Atp5b	448.02	440.636	-0.0239748	0.86955	0.926633	no
Atp5c1	282.053	269.657	-0.064842	0.6483	0.778004	no
Atp5d	211.199	209.51	-0.0115879	0.93075	0.962129	no
Atp5e	685.612	684.788	-0.00173419	0.9903	0.994103	no
Atp5f1	184.19	170.265	-0.113409	0.4595	0.616762	no
Atp5g1	84.5621	89.6908	0.0849493	0.5685	0.713387	no
Atp5g2	414.094	414.651	0.00193955	0.98895	0.993496	no
Atp5g3	395.904	380.554	-0.0570495	0.68455	0.805134	no
Atp5h	832.358	671.823	-0.309122	0.02975	0.0701013	no
Atp5j	223.508	195.241	-0.195075	0.34105	0.497571	no
Atp5j2	317.643	302.696	-0.0695363	0.62755	0.761741	no
Atp5k	766.652	711.871	-0.106958	0.46235	0.619456	no
Atp5l	487.556	449.413	-0.117523	0.4042	0.563151	no
Atp5o	221.039	209.865	-0.0748395	0.6021	0.741507	no
Atp5s	1.45077	1.39645	-0.0550588	0.87	0.926916	no
Atp5sl	35.2689	34.6602	-0.0251135	0.86895	0.926301	no
Atp6ap1	68.166	67.0778	-0.0232174	0.86685	0.924942	no
Atp6ap2	23.3402	23.8155	0.0290811	0.8495	0.914113	no
Atp6v0a2	39.3002	35.7408	-0.136962	0.33465	0.490586	no
Atp6v0b	68.2741	63.6043	-0.102213	0.4959	0.650539	no
Atp6v0c	70.7285	71.2284	0.0101621	0.95745	0.977108	no
Atp6v0d1	53.4569	55.4909	0.0538745	0.7053	0.819691	no
Atp6v0e	155.087	147.405	-0.0732966	0.6157	0.752547	no
Atp6v1a	19.0914	17.9764	-0.0868129	0.5517	0.699518	no

Atp6v1b2	33.8647	36.8244	0.12088	0.40015	0.559142	no
Atp6v1c1	38.0315	34.8356	-0.126632	0.3852	0.544221	no
Atp6v1d	83.2499	78.9103	-0.0772343	0.5922	0.7334	no
Atp6v1e1	68.4022	57.9638	-0.238891	0.10365	0.199048	no
Atp6v1f	218.451	200.228	-0.12567	0.38105	0.540011	no
Atp6v1g1	58.1614	60.3762	0.0539177	0.7215	0.830898	no
Atp6v1g2	1.66721	1.72317	0.0476315	0.8872	0.936799	no
Atp6v1h	39.2753	41.697	0.0863196	0.54865	0.696891	no
Atp7a	2.54979	2.74411	0.105961	0.53175	0.682412	no
Atp8a1	5.2275	6.15128	0.234766	0.11845	0.221886	no
Atp8b2	72.7152	78.2203	0.105287	0.4647	0.621755	no
Atp8b4	53.519	65.1826	0.284437	0.0475	0.104139	no
Atp9b	16.3169	17.2932	0.0838399	0.5594	0.705971	no
Atpaf1	7.02028	6.84706	-0.0360443	0.8419	0.909652	no
Atpaf2	18.1159	17.8635	-0.0202428	0.89885	0.944002	no
Atpif1	96.5389	90.8832	-0.0870964	0.5832	0.72601	no
Atr	9.97705	10.8278	0.118057	0.4181	0.5767	no
Atraid	41.0346	41.0356	3.52E-05	0.9999	0.9999	no
Atrip	8.83031	8.94936	0.0193203	0.90945	0.950074	no
Atrn	5.6927	5.52369	-0.0434812	0.7735	0.866246	no
Atrnl1	1.16111	1.00601	-0.206861	0.3378	0.494116	no
Atrx	33.2903	34.1012	0.0347208	0.80995	0.890105	no
Atxn1	14.1751	18.6115	0.392837	0.0056	0.0165249	yes
Atxn10	30.089	29.0013	-0.0531181	0.71705	0.827677	no
Atxn1l	7.73781	7.33467	-0.0771935	0.607	0.745334	no
Atxn2	6.34406	6.72399	0.0839108	0.60495	0.743709	no
Atxn2l	108.881	113.909	0.065134	0.6504	0.779663	no
Atxn3	10.1176	9.34475	-0.114641	0.6109	0.748378	no
Atxn7	15.7243	16.6447	0.0820683	0.56265	0.708578	no
Atxn7l1	26.2791	24.1697	-0.355683	0.09235	0.181017	no
Atxn7l2	8.20819	7.56368	-0.117977	0.50115	0.655356	no
Atxn7l3	45.1108	44.8384	-0.00873931	0.95645	0.97668	no
Atxn7l3b	19.8942	27.3027	0.456693	0.0018	0.00607738	yes
AU019823	15.4523	16.1892	0.0672145	0.6926	0.810886	no
AU022252	3.42206	3.17404	-0.108545	0.6565	0.784258	no
AU040320	19.1627	18.5987	-0.0430979	0.79315	0.879351	no
AU041133	1.3161	1.45909	0.148803	0.59055	0.732024	no
Auh	18.9125	18.3282	-0.0452795	0.7853	0.874329	no
Aup1	143.855	136.625	-0.074394	0.6	0.739763	no
Aurka	14.0957	8.13105	-0.793743	5.00E-05	0.000236281	yes
Aurkaip1	72.4937	69.9836	-0.0508396	0.7275	0.834947	no
Aurkb	23.6387	12.637	-0.903491	5.00E-05	0.000236281	yes
Aven	34.7521	31.6573	-0.134566	0.38265	0.541724	no
Avl9	13.3001	12.52	-0.0872043	0.5385	0.688209	no
Avpi1	2.45353	2.90615	0.244249	0.4596	0.616861	no
Avpr2	1.34488	0.880475	-0.611126	0.7476	0.848946	no
AW146154	2.26224	2.26013	-0.00134907	0.99	0.993915	no
AW209491	2.95176	3.33707	0.177005	0.4405	0.598498	no
AW549877	23.469	24.9683	0.0893436	0.5281	0.679408	no
AW554918	1.99982	2.04871	0.0348479	0.855	0.917876	no
Axin1	41.5606	40.6599	-0.0316072	0.8211	0.896953	no
Axin2	6.81902	10.4668	0.618178	0.00025	0.00103712	yes
Axl	3.76831	5.50463	0.546729	0.001	0.00359708	yes
AY074887	1.14274	1.14121	-0.0019328	0.9061	0.948301	no
Azi2	47.9592	41.8771	-0.195647	0.2394	0.384423	no
Azin1	33.7192	31.7986	-0.0846104	0.5576	0.704468	no
B230219D22Rik	22.8071	22.9905	0.0115586	0.93705	0.965841	no

B2m	4296.16	3788.18	-0.181541	0.2986	0.450617	no
B3galnt2	11.3392	11.2437	-0.0121961	0.95455	0.9757	no
B3galt4	11.5558	12.015	0.0562123	0.75245	0.852029	no
B3galt6	1.94684	2.45539	0.334816	0.1341	0.245062	no
B3gat2	0.853163	1.00031	0.229557	0.8323	0.903775	no
B3gat3	65.4079	64.5345	-0.0193944	0.8916	0.939323	no
B3glct	3.89854	2.97425	-0.390407	0.031	0.0725382	no
B3gnt1	5.85205	6.13561	0.068266	0.7225	0.831616	no
B3gnt2	55.7517	51.0335	-0.127571	0.36755	0.526034	no
B3gnt3	1.36797	1.66147	0.280421	0.3332	0.489009	no
B3gnt5	15.1204	14.9168	-0.0195582	0.8949	0.941353	no
B3gnt9	3.89662	4.15303	0.0919413	0.7749	0.867278	no
B3gntl1	10.3639	9.65203	-0.102668	0.72345	0.832153	no
B4galnt1	377.432	377.892	0.00175737	0.98935	0.99368	no
B4galnt4	0.680748	2.37015	1.79978	5.00E-05	0.000236281	yes
B4galt1	62.8162	60.0066	-0.066015	0.63025	0.76385	no
B4galt3	11.0641	10.125	-0.127969	0.46375	0.620802	no
B4galt5	31.6241	27.3868	-0.207545	0.14805	0.264354	no
B4galt7	47.5422	45.2787	-0.0703789	0.6239	0.758877	no
B630005N14Rik	3.38671	3.02556	-0.162684	0.38335	0.542411	no
B9d2	16.1259	14.1452	-0.189065	0.3221	0.477003	no
Babam1	49.1791	45.6734	-0.106691	0.46785	0.624561	no
Bace1	2.5524	2.74055	0.10261	0.60045	0.740149	no
Bach1	7.03625	7.06343	0.00556186	0.9728	0.985025	no
Bach2	1.75012	2.269	0.374601	0.03635	0.0829933	no
Bad	20.7198	20.275	-0.0313083	0.88495	0.935641	no
Bag1	106.172	99.8148	-0.0890716	0.53725	0.687346	no
Bag2	2.32361	2.70099	0.217122	0.40305	0.561993	no
Bag3	4.71858	2.52086	-0.904435	2.00E-04	0.0008488	yes
Bag4	6.37503	6.54454	0.0378591	0.81505	0.892999	no
Bag5	12.902	12.75	-0.0171042	0.91695	0.954462	no
Bag6	58.2007	57.7498	-0.0112217	0.9362	0.96536	no
Bahd1	12.7779	13.1061	0.0365959	0.80755	0.888491	no
Baiap2	15.6436	12.8432	-0.284562	0.0814	0.162971	no
Baiap3	94.6156	82.874	-0.191159	0.19865	0.334449	no
Bak1	59.4119	50.3793	-0.237919	0.10045	0.194006	no
Banf1	143.952	128.705	-0.161525	0.42005	0.578531	no
Banp	6.43682	6.22929	-0.0472798	0.76595	0.861125	no
Bap1	28.5931	27.7185	-0.0448208	0.7553	0.853939	no
Bard1	3.66018	2.34046	-0.645124	0.00075	0.00278555	yes
Batf	63.479	73.3357	0.208236	0.17275	0.299289	no
Bax	121.524	138.845	0.192234	0.1789	0.307791	no
Baz1a	36.2357	36.8797	0.0254151	0.85885	0.920092	no
Baz1b	42.9264	39.2847	-0.127897	0.36285	0.521093	no
Baz2a	34.0149	33.05	-0.0415154	0.76985	0.863803	no
Baz2b	4.29141	6.80522	0.665189	5.00E-05	0.000236281	yes
Bbc3	9.33764	9.35834	0.00319406	0.9828	0.990094	no
Bbip1	23.797	20.9983	-0.180505	0.67275	0.796325	no
Bbs12	2.03386	2.06679	0.0231699	0.92565	0.959443	no
Bbs2	2.1605	3.20276	0.56795	0.00995	0.0273191	yes
Bbs4	4.58764	5.8628	0.353838	0.06275	0.131616	no
Bbs9	5.08735	6.42658	0.337139	0.05195	0.11227	no
Bbx	4.26985	4.85348	0.184833	0.22835	0.371429	no
BC003331	13.0397	11.8173	-0.14201	0.77995	0.870597	no
BC003965	8.97992	9.04462	0.0103585	0.9501	0.973139	no
BC004004	21.6977	21.8599	0.0107498	0.9423	0.968678	no
BC005537	121.304	126.13	0.0562916	0.69615	0.81341	no

BC005561	1.34346	1.80331	0.42469	0.0497	0.108233	no
BC005624	50.9083	45.4825	-0.162591	0.2776	0.426847	no
BC005764	6.41026	6.95337	0.11733	0.8654	0.92408	no
BC017158	8.09003	9.11616	0.172282	0.3127	0.4664	no
BC017643	44.2649	36.3829	-0.282902	0.0551	0.11809	no
BC018507	18.5261	19.0361	0.0391755	0.7799	0.870555	no
BC021614	73.0555	93.1694	0.350863	0.0219	0.0539961	no
BC023829	9.07429	10.7843	0.249078	0.16105	0.283091	no
BC024978	2.9214	2.89936	-0.0109282	0.98285	0.990102	no
BC027231	9.68458	9.78632	0.0150777	0.927	0.960222	no
BC029214	30.6545	30.5065	-0.00698522	0.9655	0.981181	no
BC030336	5.49817	4.90151	-0.165726	0.3147	0.468532	no
BC030867	3.2415	1.76401	-0.877805	0.00055	0.0021091	yes
BC031181	85.1369	88.2407	0.0516599	0.72	0.829861	no
BC035044	2.97745	2.2579	-0.399097	0.57175	0.716179	no
BC037034	39.3526	42.8044	0.121301	0.4071	0.565918	no
BC048403	2.4265	3.44783	0.50681	0.01855	0.0466972	yes
BC052040	5.81681	4.05298	-0.521245	0.0064	0.018565	yes
BC053749	2.56214	2.68821	0.0692967	0.76545	0.860823	no
BC055324	3.0146	2.20222	-0.453007	0.3366	0.49279	no
BC068281	2.54132	2.90798	0.194439	0.39635	0.555372	no
BC094916	35.0519	31.3225	-0.162291	0.29775	0.44968	no
Bcap29	23.8152	26.0831	0.131234	0.3922	0.55133	no
Bcap31	142.742	134.548	-0.0852869	0.5443	0.693136	no
Bcas2	72.1724	66.8253	-0.111052	0.439	0.597058	no
Bcas3	13.5747	14.5529	0.100391	0.5147	0.667571	no
Bcat2	22.167	20.8026	-0.0916514	0.55475	0.701998	no
Bccip	49.9158	51.5715	0.0470776	0.7887	0.876703	no
Bcdin3d	6.74372	6.42951	-0.0688361	0.75895	0.856543	no
Bckdha	23.667	23.2206	-0.0274737	0.86095	0.921489	no
Bckdhb	5.62471	5.39096	-0.0612352	0.78485	0.874089	no
Bckdk	11.7334	12.3332	0.0719352	0.65575	0.783786	no
Bcl10	43.6007	42.0951	-0.0506982	0.7246	0.832932	no
Bcl11b	42.7961	40.9897	-0.0622187	0.6615	0.788123	no
Bcl2	4.70118	8.12677	0.789661	5.00E-05	0.000236281	yes
Bcl2a1b	79.6681	69.6605	-0.193662	0.2092	0.347962	no
Bcl2a1d	29.6883	27.5712	-0.106734	0.5442	0.693084	no
Bcl2l1	16.1801	14.5817	-0.150054	0.3358	0.491813	no
Bcl2l11	16.114	14.7045	-0.132062	0.40305	0.561993	no
Bcl2l12	16.2821	15.7165	-0.0510022	0.79975	0.883518	no
Bcl2l13	12.5701	10.6995	-0.23245	0.1081	0.206075	no
Bcl2l2	1.82636	2.16377	0.244569	0.28125	0.431051	no
Bcl3	11.3712	10.7189	-0.0852311	0.6258	0.760415	no
Bcl6	3.08883	3.03979	-0.0230917	0.9084	0.949549	no
Bcl7a	4.55696	4.82931	0.0837461	0.63855	0.770548	no
Bcl7b	35.6531	34.6853	-0.0397032	0.7958	0.881085	no
Bcl7c	15.4581	15.0281	-0.0406993	0.8342	0.904975	no
Bcl9l	36.8078	39.9835	0.119395	0.39635	0.555372	no
Bclaf1	34.6463	35.3767	0.0300966	0.83135	0.903216	no
Bcor	16.2923	18.4448	0.179027	0.2223	0.364054	no
Bcorl1	1.7833	1.66007	-0.103308	0.5942	0.735129	no
Bcr	4.99152	6.44421	0.368524	0.01825	0.0460274	yes
Bcs1l	6.39002	6.43247	0.00955336	0.96115	0.9791	no
Bdh1	13.2249	13.9404	0.0760086	0.61895	0.755068	no
Bdp1	9.32332	10.6055	0.185896	0.1986	0.334373	no
Becn1	109.305	103.887	-0.0733384	0.60095	0.740559	no
Bend3	1.13113	1.40279	0.310537	0.15315	0.271776	no

Bend4	1.43225	1.33895	-0.0971795	0.6159	0.752739	no
Bend5	0.923188	1.32712	0.523597	0.13045	0.239752	no
Bet1	11.0996	12.0813	0.122267	0.4963	0.650834	no
Bet1l	20.2758	20.9368	0.0462817	0.7779	0.869229	no
Bfar	23.6931	23.8311	0.00837923	0.95555	0.976242	no
Bhlhb9	5.32096	6.3704	0.259699	0.1511	0.268821	no
Bhlhe40	131.897	102.499	-0.363798	0.01105	0.0299157	yes
Bicd2	19.5602	20.3777	0.0590748	0.68045	0.80209	no
Bid	17.2008	20.4523	0.249783	0.1184	0.221822	no
Bik	1.48065	1.22881	-0.268971	0.54575	0.694256	no
Bin1	92.5945	85.372	-0.117164	0.4052	0.564188	no
Bin2	459.71	427.326	-0.105384	0.47045	0.627048	no
Bin3	41.8266	35.8908	-0.220807	0.3539	0.511497	no
Birc2	31.9088	34.2164	0.100733	0.47795	0.634039	no
Birc3	19.4112	18.1763	-0.094828	0.5283	0.679554	no
Birc5	96.4331	56.3144	-0.776024	5.00E-05	0.000236281	yes
Birc6	40.3841	44.5862	0.142809	0.325	0.48018	no
Bivm	3.29503	3.90168	0.243805	0.19555	0.330262	no
Blcap	5.02369	5.09221	0.0195463	0.92165	0.957392	no
Blm	3.20153	2.58251	-0.309988	0.09915	0.191979	no
Blmh	41.4148	38.5394	-0.103813	0.4606	0.617792	no
Bloc1s1	126.299	122.514	-0.043899	0.77725	0.868861	no
Bloc1s2	56.8309	56.3205	-0.0130146	0.9326	0.963238	no
Bloc1s3	11.7294	10.4321	-0.169103	0.33735	0.493581	no
Bloc1s4	21.665	22.2632	0.039294	0.81285	0.891792	no
Bloc1s5	5.60523	5.6473	0.0107882	0.95435	0.975581	no
Bloc1s6	12.882	12.0321	-0.0984733	0.5208	0.672862	no
Blvra	30.1087	29.4324	-0.032774	0.83645	0.906302	no
Blvrb	1.86033	2.24274	0.269704	0.47975	0.635555	no
Blzf1	5.74179	6.41123	0.1591	0.34455	0.501413	no
Bmf	2.38729	2.05393	-0.216986	0.277	0.426082	no
Bmi1	9.90069	11.2649	0.186235	0.2338	0.377803	no
Bmp2k	4.46749	5.53182	0.308289	0.0532	0.114547	no
Bms1	27.7303	27.8839	0.00796974	0.956	0.976447	no
Bmyc	2.93989	3.32295	0.176704	0.6003	0.740042	no
Bnip1	17.1429	18.5814	0.116249	0.51765	0.670154	no
Bnip2	25.1652	21.1556	-0.250393	0.0797	0.160071	no
Bnip3l	63.5037	60.5838	-0.0679086	0.6368	0.769054	no
Bod1	7.8136	7.73386	-0.0147983	0.93825	0.96664	no
Bod1l	12.2865	13.8234	0.170041	0.2329	0.376806	no
Bola1	9.10937	14.2776	0.64833	0.0062	0.0180576	yes
Bola2	263.979	279.735	0.0836378	0.59625	0.736683	no
Bola3	61.5354	69.1282	0.167857	0.32645	0.481778	no
Bop1	34.7534	34.8835	0.00538988	0.97805	0.98772	no
Bora	8.28274	7.44731	-0.153388	0.38365	0.542705	no
Bpgm	12.4031	11.5415	-0.103869	0.53065	0.681521	no
Bphl	1.96067	3.11788	0.669218	0.03225	0.0749809	no
Bpnt1	7.56573	9.31216	0.299636	0.0912	0.179166	no
Bptf	22.5908	25.0502	0.149089	0.2965	0.448214	no
Braf	4.69666	4.74799	0.0156821	0.91815	0.95519	no
Brap	32.2248	32.3469	0.00545656	0.97115	0.984202	no
Brat1	11.4958	11.3558	-0.0176713	0.90765	0.949177	no
Brca1	3.83822	2.55698	-0.586	0.00115	0.00407292	yes
Brca2	1.21483	0.860764	-0.497061	0.01155	0.0310715	yes
Brcc3	30.4806	28.9911	-0.072282	0.7023	0.817402	no
Brd1	32.3812	34.5724	0.0944655	0.5036	0.657707	no
Brd2	94.2619	105.696	0.165176	0.2415	0.386065	no

Brd3	10.5216	9.05687	-0.21627	0.15125	0.26902	no
Brd4	51.3495	50.786	-0.0159193	0.915	0.953431	no
Brd7	42.7651	39.8543	-0.101699	0.47955	0.635361	no
Brd8	21.2731	20.9017	-0.0254123	0.898	0.943447	no
Brd9	62.1079	54.2049	-0.196354	0.1704	0.296075	no
Bre	35.8142	34.2485	-0.0644933	0.67655	0.799174	no
Brf1	37.1088	41.5687	0.163736	0.3126	0.466281	no
Brf2	7.70787	7.58353	-0.0234632	0.90765	0.949177	no
Bri3	20.1208	21.4184	0.0901648	0.6184	0.754697	no
Bri3bp	2.46619	3.14551	0.35101	0.0437	0.0970589	no
Bricd5	7.46553	7.22154	-0.0479376	0.9129	0.952325	no
Brip1	2.41134	1.392	-0.792675	5.00E-05	0.000236281	yes
Brix1	16.0125	16.0617	0.00442743	0.9757	0.986631	no
Brk1	148.765	142.097	-0.0661589	0.63995	0.771446	no
Brms1	52.1227	49.6949	-0.0688135	0.64875	0.778346	no
Brms1l	5.08134	4.41052	-0.204259	0.28495	0.435276	no
Brpf1	22.0465	20.8829	-0.0782263	0.5903	0.731817	no
Brpf3	11.0447	9.56428	-0.207626	0.16315	0.286098	no
Brwd1	15.3424	16.5582	0.110031	0.52605	0.67745	no
Brwd3	4.44798	4.77138	0.101254	0.52865	0.67983	no
Bscl2	116.021	100.03	-0.213946	0.13755	0.24977	no
Bsdc1	27.0217	26.1452	-0.0475711	0.7469	0.848547	no
Bsg	160.389	159.913	-0.00428298	0.977	0.987224	no
Bspry	17.7965	17.3471	-0.0368991	0.82495	0.899308	no
Bst2	62.711	70.3578	0.165992	0.27725	0.42642	no
Btaf1	10.5934	11.414	0.107635	0.45315	0.610677	no
Btbd1	32.8103	31.8264	-0.0439234	0.7577	0.855556	no
Btbd10	17.5245	14.52	-0.27133	0.0821	0.164121	no
Btbd11	5.955	6.12955	0.0416801	0.7938	0.879754	no
Btbd2	10.23	11.2014	0.130883	0.40965	0.568634	no
Btbd6	13.1365	15.7593	0.262625	0.40555	0.564553	no
Btbd7	10.4024	9.81855	-0.0833394	0.5881	0.730077	no
Btbd9	4.33951	4.42895	0.0294302	0.85515	0.917912	no
Btd	9.57669	8.82778	-0.117477	0.5182	0.670607	no
Btf3	826.517	752.571	-0.135217	0.34305	0.499828	no
Btf3l4	8.20146	7.76728	-0.0784714	0.63595	0.76837	no
Btg1	212.769	212.886	0.000795216	0.99625	0.997296	no
Btg2	68.4949	74.3014	0.117393	0.40415	0.563115	no
Btla	11.116	15.814	0.508558	0.00115	0.00407292	yes
Btrc	2.7721	2.94156	0.0856012	0.63135	0.764867	no
Bub1	9.33884	4.60749	-1.01926	5.00E-05	0.000236281	yes
Bub1b	17.1577	9.44836	-0.86072	5.00E-05	0.000236281	yes
Bub3	60.5106	53.6927	-0.172462	0.22995	0.3733	no
Bud13	12.436	11.1579	-0.156452	0.3481	0.505298	no
Bud31	77.7677	66.9004	-0.217156	0.33565	0.491664	no
Bysl	24.1538	20.5801	-0.230999	0.436	0.594076	no
Bzrap1	4.55251	3.09932	-0.55471	0.00125	0.00438681	yes
Bzw1	149.353	139.424	-0.0992453	0.48695	0.642152	no
Bzw2	35.4114	35.3833	-0.00114528	0.9942	0.996289	no
C030006K11Rik	5.99895	6.82858	0.186876	0.35715	0.514961	no
C030046E11Rik	11.6394	11.6817	0.00523209	0.9721	0.984684	no
C1d	14.6836	13.0873	-0.166041	0.2845	0.434739	no
C1galt1	24.4798	25.7843	0.0748987	0.62015	0.755957	no
C1galt1c1	8.36445	8.59222	0.0387597	0.8365	0.906314	no
C1qbp	48.6842	52.5505	0.110252	0.5047	0.658765	no
C1qtnf4	0.698395	1.19548	0.77547	0.0782	0.157588	no
C1qtnf6	4.16264	3.00365	-0.470781	0.02825	0.067118	no

C230052I12Rik	8.74683	8.91741	0.0278648	0.8816	0.933694	no
C2cd2	2.04054	2.31654	0.183021	0.3194	0.47397	no
C2cd2l	8.01102	8.54887	0.0937466	0.5489	0.697083	no
C2cd3	18.9364	17.903	-0.0809619	0.56855	0.713424	no
C2cd5	17.4204	18.1262	0.0572976	0.68775	0.807351	no
C330006A16Rik	8.54977	9.78259	0.194331	0.2508	0.396461	no
C330007P06Rik	14.1652	13.7997	-0.0377188	0.8054	0.887094	no
C330018D20Rik	4.55642	5.28381	0.213676	0.27175	0.419846	no
C330027C09Rik	12.5589	8.18917	-0.616919	5.00E-05	0.000236281	yes
C78339	3.94072	3.30856	-0.252257	0.3303	0.485976	no
C87436	7.81459	7.60013	-0.0401471	0.8109	0.890677	no
C8g	2.95043	2.46951	-0.256702	0.7758	0.867913	no
C920025E04Rik	25.604	25.3944	-0.0118571	0.9422	0.968641	no
Caap1	9.5496	10.1628	0.0897904	0.60935	0.747128	no
Cab39	95.3496	86.3716	-0.14267	0.31275	0.466426	no
Cab39l	20.3802	19.1452	-0.0901822	0.5484	0.696673	no
Cabin1	28.4079	32.5222	0.195132	0.1677	0.292509	no
Cables2	14.0595	16.1218	0.197463	0.19955	0.335548	no
Cacfd1	15.4219	14.8168	-0.0577523	0.73485	0.840145	no
Cacna2d4	1.5563	1.17261	-0.408395	0.0557	0.119202	no
Cacnb1	3.07302	5.58706	0.86243	1.00E-04	0.000450026	yes
Cacnb3	1.47864	2.4762	0.743856	0.0043	0.013125	yes
Cactin	26.8548	26.4448	-0.0221984	0.8796	0.932569	no
Cacul1	14.343	14.4113	0.00685391	0.9634	0.980274	no
Cacybp	39.68	37.947	-0.0644262	0.66205	0.788579	no
Cad	12.0985	13.6884	0.178128	0.71725	0.827787	no
Calcoco1	19.8212	20.2372	0.0299675	0.8442	0.91116	no
Calcrl	3.11375	3.07418	-0.0184479	0.91655	0.954243	no
Calhm2	8.75914	7.29034	-0.264803	0.15155	0.269445	no
Calm1	235.363	204.833	-0.200439	0.17495	0.302233	no
Calm2	315.271	265.833	-0.246075	0.0826	0.164994	no
Calm3	151.068	143.843	-0.070707	0.61085	0.748343	no
Calr	313.546	334.577	0.0936636	0.5176	0.670113	no
Calu	40.6906	35.3938	-0.2012	0.1561	0.275998	no
Camk1d	13.0641	15.767	0.271296	0.0621	0.130455	no
Camk2b	2.56357	2.40189	-0.0939837	0.6442	0.774849	no
Camk2d	11.8657	14.1986	0.258954	0.0843	0.167877	no
Camk2g	40.3011	41.84	0.0540625	0.7071	0.821075	no
Camk2n1	1.57373	1.35034	-0.220866	0.36345	0.521679	no
Camk4	11.8297	14.405	0.284164	0.04575	0.100913	no
Camkk2	4.32715	4.17097	-0.0530331	0.7615	0.858131	no
Camkmt	1.30746	1.77051	0.4374	0.20165	0.338193	no
Caml	17.5226	17.5481	0.00209486	0.9875	0.992732	no
Camsap1	3.72636	4.27322	0.197556	0.21235	0.35204	no
Camsap2	3.77008	3.82776	0.0219082	0.88935	0.937989	no
Camta1	3.12925	2.83572	-0.142105	0.5314	0.682199	no
Camta2	22.2331	23.61	0.0866851	0.5477	0.695997	no
Cand1	13.2487	13.7041	0.0487489	0.7324	0.838471	no
Cant1	33.0271	33.5134	0.0210853	0.8854	0.935946	no
Canx	62.9648	68.3402	0.118189	0.4046	0.563508	no
Cap1	508.365	442.235	-0.201054	0.18095	0.310651	no
Capg	30.0801	26.9433	-0.158884	0.32365	0.478736	no
Capn1	103.376	107.541	0.05699	0.69495	0.812483	no
Capn10	5.51458	5.41149	-0.0272272	0.88025	0.932877	no
Capn11	11.0808	3.51731	-1.65552	5.00E-05	0.000236281	yes
Capn15	8.72561	9.47551	0.118948	0.46215	0.619259	no
Capn2	116.782	90.8128	-0.362847	0.012	0.0320694	yes

Capn7	30.8619	32.5598	0.0772611	0.59055	0.732024	no
Capns1	230.497	219.222	-0.072353	0.61155	0.748901	no
Caprin1	68.437	65.3852	-0.065812	0.65705	0.784623	no
Caprin2	2.49567	2.18166	-0.193997	0.36225	0.520464	no
Capza1	250.663	222.422	-0.172451	0.2384	0.383262	no
Capza2	120.543	111.565	-0.111668	0.42935	0.587636	no
Capzb	455.348	390.966	-0.219926	0.12765	0.235562	no
Car15	3.57704	2.2323	-0.680238	0.02945	0.0695104	no
Car2	2.24148	1.5963	-0.489717	0.0872	0.172624	no
Car5b	11.737	8.97981	-0.386308	0.0153	0.0395135	yes
Card11	66.3718	59.7429	-0.151803	0.2826	0.432556	no
Card6	21.8327	23.1498	0.0845122	0.5517	0.699518	no
Carhsp1	24.7977	26.2121	0.0800267	0.58325	0.72601	no
Carkd	18.1133	17.8626	-0.0201085	0.9065	0.948565	no
Carm1	8.15563	8.95348	0.134653	0.63995	0.771446	no
Carns1	6.15071	5.84109	-0.0745158	0.6563	0.784112	no
Cars	9.47134	9.54432	0.0110744	0.94765	0.971828	no
Cars2	20.6966	20.0755	-0.0439585	0.77955	0.870357	no
Casc3	34.5578	35.697	0.0467911	0.81535	0.893175	no
Casc4	6.42445	9.28727	0.531681	0.001	0.00359708	yes
Casc5	4.32793	2.46062	-0.814655	5.00E-05	0.000236281	yes
Casd1	8.96678	10.0016	0.157573	0.3217	0.476591	no
Caskin2	1.96919	2.20675	0.164319	0.4106	0.569505	no
Casp1	61.3719	54.1452	-0.180745	0.21445	0.354638	no
Casp2	21.6555	19.8407	-0.126268	0.386	0.545024	no
Casp3	31.5361	30.2212	-0.0614442	0.67695	0.799459	no
Casp4	18.3058	18.9245	0.0479584	0.77345	0.866232	no
Casp6	19.716	19.3807	-0.0247457	0.8782	0.931855	no
Casp7	19.2438	16.3026	-0.23929	0.12345	0.229274	no
Casp8	59.2397	54.753	-0.113627	0.426	0.584399	no
Casp8ap2	12.9027	11.8531	-0.122413	0.39985	0.558878	no
Casp9	7.99231	7.1733	-0.155974	0.33465	0.490586	no
Cast	89.2939	84.3456	-0.0822493	0.5644	0.710085	no
Cat	59.9608	55.6454	-0.107757	0.45065	0.608075	no
Cbfa2t2	4.45843	4.28367	-0.0576871	0.72825	0.835319	no
Cbfa2t3	0.313353	1.19927	1.93629	5.00E-05	0.000236281	yes
Cbfb	85.1344	74.826	-0.186203	0.19265	0.326364	no
Cbl	24.0553	34.5302	0.521501	0.00015	0.000653255	yes
Cblb	24.6606	26.2239	0.0886764	0.5315	0.682265	no
Cbll1	8.42996	8.33446	-0.0164366	0.9181	0.95519	no
Cbr1	27.7416	29.0701	0.0674844	0.6895	0.808665	no
Cbr4	6.66633	7.61103	0.191199	0.4051	0.564082	no
Cbwd1	5.04122	5.51673	0.130041	0.52215	0.674125	no
Cbx1	33.7545	32.2767	-0.0645852	0.6783	0.800478	no
Cbx2	2.47236	1.87456	-0.399337	0.06335	0.132465	no
Cbx3	136.704	125.929	-0.118441	0.4061	0.565051	no
Cbx4	22.7514	25.806	0.181754	0.19965	0.335692	no
Cbx5	11.5953	10.0071	-0.212508	0.15165	0.269589	no
Cbx6	7.81909	8.11609	0.0537825	0.72345	0.832153	no
Cbx7	30.2478	34.612	0.194444	0.16995	0.295395	no
Cbx8	10.4619	11.2819	0.108863	0.56175	0.707823	no
Cby1	9.23155	11.4733	0.313629	0.119	0.222757	no
Cc2d1a	18.046	17.3121	-0.0598942	0.6943	0.812015	no
Cc2d1b	46.651	47.3768	0.0222723	0.87945	0.93248	no
Ccar1	33.222	33.9787	0.0324909	0.81645	0.893991	no
Ccar2	30.2251	30.7898	0.0267085	0.88005	0.932752	no
Ccbl1	4.74837	5.52017	0.217279	0.2966	0.448346	no

Ccdc101	23.8088	23.9314	0.00740839	0.96215	0.979794	no
Ccdc102a	8.10099	7.21256	-0.167585	0.35515	0.512779	no
Ccdc104	17.6427	16.3276	-0.11176	0.51025	0.66358	no
Ccdc107	18.2859	23.5395	0.364351	0.05215	0.11265	no
Ccdc109b	8.35108	6.09525	-0.454277	0.03285	0.0761651	no
Ccdc115	22.809	25.4831	0.159938	0.2993	0.45147	no
Ccdc117	14.4739	16.5333	0.191917	0.2071	0.34525	no
Ccdc12	164.319	160.083	-0.0376773	0.79125	0.878196	no
Ccdc124	81.8657	79.2622	-0.0466251	0.75125	0.851338	no
Ccdc125	9.60617	13.2785	0.467062	0.0467	0.102656	no
Ccdc126	2.07464	2.23854	0.109694	0.66535	0.790855	no
Ccdc127	8.20974	7.8607	-0.0626788	0.7051	0.819594	no
Ccdc130	9.97152	9.48213	-0.0726025	0.6728	0.796357	no
Ccdc132	6.24771	6.38213	0.0307109	0.8542	0.917352	no
Ccdc134	18.5608	17.8515	-0.0562092	0.7248	0.833108	no
Ccdc137	8.67974	9.39959	0.114946	0.56735	0.712412	no
Ccdc138	1.79294	2.3173	0.370117	0.1421	0.256016	no
Ccdc14	1.1813	1.09539	-0.108935	0.6676	0.792555	no
Ccdc142	6.31006	7.43125	0.235951	0.26345	0.410002	no
Ccdc15	0.912902	1.09929	0.268037	0.3377	0.49398	no
Ccdc157	1.09549	1.73078	0.659837	0.0666	0.137937	no
Ccdc163	5.97919	4.16614	-0.521239	0.03565	0.0816672	no
Ccdc166	6.76121	6.50014	-0.0568115	0.75155	0.851514	no
Ccdc167	10.8218	11.1547	0.0437104	0.9149	0.953407	no
Ccdc17	1.73519	1.79257	0.0469339	0.96525	0.98104	no
Ccdc171	1.78807	2.04143	0.191177	0.36915	0.527767	no
Ccdc173	0.813371	1.19837	0.559089	0.13535	0.246804	no
Ccdc174	19.4633	18.3413	-0.0856613	0.59835	0.738438	no
Ccdc176	0.670848	1.21476	0.85662	0.25015	0.395672	no
Ccdc18	1.11842	0.767935	-0.542408	0.03735	0.0849194	no
Ccdc181	5.68672	5.35981	-0.0854142	0.665	0.790518	no
Ccdc22	13.8691	15.1391	0.126406	0.4555	0.613095	no
Ccdc23	52.8958	49.3595	-0.0998255	0.53865	0.688326	no
Ccdc25	26.8895	26.3312	-0.030268	0.8418	0.909585	no
Ccdc28a	6.24113	6.87806	0.140194	0.53675	0.686918	no
Ccdc28b	4.02193	4.47713	0.154689	0.60395	0.742855	no
Ccdc30	2.3242	2.81541	0.276607	0.20185	0.338464	no
Ccdc32	15.7679	15.0504	-0.0671866	0.68405	0.804761	no
Ccdc34	6.5153	4.27683	-0.60729	0.0027	0.00870467	yes
Ccdc43	14.8508	14.3547	-0.0490182	0.75675	0.854908	no
Ccdc47	31.8516	33.5708	0.0758427	0.598	0.738122	no
Ccdc50	32.0574	28.2866	-0.180535	0.20615	0.344032	no
Ccdc51	4.67274	4.92435	0.0756655	0.70405	0.818778	no
Ccdc53	42.9032	42.6245	-0.00940172	0.95175	0.97415	no
Ccdc55	11.2851	12.2071	0.113308	0.4706	0.627105	no
Ccdc58	9.39383	9.0673	-0.0510406	0.8283	0.901373	no
Ccdc59	40.0117	37.7214	-0.0850399	0.5861	0.728325	no
Ccdc6	13.1305	14.3695	0.13009	0.3694	0.527975	no
Ccdc61	13.6015	13.0815	-0.0562378	0.73225	0.838367	no
Ccdc64	27.0923	30.4692	0.169467	0.263	0.409437	no
Ccdc66	13.6159	12.8835	-0.0797684	0.7644	0.860191	no
Ccdc69	37.1009	34.7042	-0.0963444	0.53095	0.681807	no
Ccdc71	15.0848	13.7695	-0.131614	0.4023	0.56128	no
Ccdc71l	13.8218	11.4537	-0.271129	0.072	0.147225	no
Ccdc77	5.32602	4.34826	-0.292618	0.63085	0.764406	no
Ccdc84	12.9648	13.215	0.0275863	0.9814	0.989348	no
Ccdc85b	8.20413	8.88707	0.115358	0.45405	0.611727	no

Ccdc86	5.72609	6.25826	0.128211	0.4765	0.63271	no
Ccdc88b	67.6435	69.2812	0.0345115	0.80385	0.886272	no
Ccdc88c	86.3658	85.628	-0.0123784	0.93205	0.962853	no
Ccdc9	26.6278	27.2252	0.0320068	0.8291	0.901827	no
Ccdc90b	2.86155	2.64458	-0.113755	0.66485	0.790407	no
Ccdc91	3.18761	4.25022	0.415063	0.04945	0.107745	no
Ccdc92	9.06133	11.1555	0.299962	0.07925	0.159353	no
Ccdc93	10.4099	9.9359	-0.0672377	0.64295	0.773847	no
Ccdc94	15.4043	13.6102	-0.178639	0.3199	0.474542	no
Ccdc97	29.6562	27.9542	-0.0852658	0.55565	0.702722	no
Cchcr1	10.7853	7.91171	-0.447006	0.00885	0.0246381	yes
Ccin	1.19636	1.13495	-0.0760268	0.8142	0.892483	no
Ccl25	1.37014	1.63159	0.251956	0.5165	0.669254	no
Ccl27a	3.64618	2.66353	-0.453047	0.41815	0.576747	no
Ccl3	178.659	65.2524	-1.45311	5.00E-05	0.000236281	yes
Ccl4	285.446	145.813	-0.969099	5.00E-05	0.000236281	yes
Ccl5	19807.6	12463.3	-0.668369	0.018	0.0454732	yes
Ccl6	2.503	1.42108	-0.816673	0.01975	0.0492996	yes
Ccl9	5.23507	2.84873	-0.877889	5.00E-05	0.000236281	yes
Ccm2	57.983	62.2494	0.10243	0.476	0.632212	no
Ccna2	49.0848	26.8185	-0.872045	5.00E-05	0.000236281	yes
Ccnb1	20.9379	11.5462	-0.858701	5.00E-05	0.000236281	yes
Ccnb2	57.9815	32.8512	-0.819648	5.00E-05	0.000236281	yes
Ccnc	15.9982	13.819	-0.211249	0.3475	0.504634	no
Ccnd2	29.0314	25.7612	-0.172411	0.2239	0.366131	no
Ccnd3	173.651	133.467	-0.379704	0.01975	0.0492996	yes
Ccndbp1	87.3966	81.5871	-0.0992371	0.48955	0.644545	no
Ccne1	20.645	14.3657	-0.523163	0.00165	0.00562393	yes
Ccne2	1.65354	0.990963	-0.738657	0.342	0.498607	no
Ccnf	10.4184	6.4639	-0.68866	1.00E-04	0.000450026	yes
Ccng1	48.0962	56.7223	0.237993	0.0929	0.181908	no
Ccng2	37.7837	29.6438	-0.350033	0.01635	0.041841	yes
Ccnh	15.6764	14.5436	-0.10821	0.51255	0.665639	no
Ccni	87.6162	77.5091	-0.176833	0.21185	0.351425	no
Ccnj	3.08541	3.44622	0.159554	0.4003	0.559285	no
Ccnk	35.9861	34.787	-0.0488941	0.7368	0.841679	no
Ccnl1	155.824	154.795	-0.00956234	0.94565	0.970482	no
Ccnl2	95.0603	96.1079	0.0158115	0.9118	0.951599	no
Ccno	1.05571	0.463176	-1.18858	0.00845	0.023657	yes
Ccnt1	15.5618	15.8869	0.0298322	0.853	0.916538	no
Ccnt2	25.1017	26.2224	0.0630142	0.6629	0.789178	no
Ccny	23.3332	25.3256	0.118208	0.40335	0.562311	no
Ccnyl1	3.10954	2.99764	-0.0528732	0.79225	0.878781	no
Ccp110	3.95411	3.02824	-0.384872	0.0314	0.073338	no
Ccpg1	39.9493	37.9237	-0.075071	0.6289	0.762711	no
Ccpg1os	4.40281	4.27238	-0.0433851	0.88945	0.938066	no
Ccr10	0.508405	1.602	1.65582	7.00E-04	0.00261609	yes
Ccr2	167.479	187.504	0.162939	0.25645	0.401445	no
Ccr5	75.1431	58.0739	-0.371751	0.00965	0.026603	yes
Ccr7	5.68635	8.8038	0.630623	0.0013	0.00454533	yes
Ccrl2	1.2001	1.04313	-0.202247	0.53005	0.68106	no
Ccrn4l	5.11454	5.73066	0.164096	0.3644	0.522649	no
Ccs	36.2989	41.7479	0.201776	0.202	0.338651	no
Ccsap	5.72506	4.96212	-0.206335	0.2676	0.414997	no
Ccser2	17.5717	15.9212	-0.142302	0.32755	0.483068	no
Cct2	209.159	198.685	-0.0741173	0.60155	0.741117	no
Cct3	85.7769	90.8937	0.0835912	0.55875	0.70553	no

Cct4	136.524	118.865	-0.199825	0.1579	0.278622	no
Cct5	175.847	163.184	-0.107823	0.43965	0.597631	no
Cct6a	68.1426	74.1833	0.122537	0.3821	0.541152	no
Cct7	95.9229	89.6506	-0.0975618	0.4988	0.653045	no
Cct8	107.69	104.603	-0.0419614	0.7966	0.881556	no
Ccz1	44.1702	42.8566	-0.0435537	0.76745	0.862111	no
Cd101	2.11358	1.68382	-0.327948	0.15295	0.271483	no
Cd151	4.39837	4.04387	-0.121235	0.57725	0.720894	no
Cd160	51.6494	43.8506	-0.236156	0.1034	0.198649	no
Cd163l1	15.824	31.8422	1.00882	5.00E-05	0.000236281	yes
Cd164	177.596	187.895	0.0813294	0.56965	0.714347	no
Cd1d1	22.1226	20.5296	-0.107816	0.4925	0.647222	no
Cd2	496.474	495.641	-0.00242459	0.9869	0.992412	no
Cd200r1	1.01373	1.17085	0.207887	0.5466	0.695112	no
Cd200r4	11.8483	10.1757	-0.219544	0.2233	0.365378	no
Cd209c	1.7878	2.02336	0.178569	0.5577	0.704519	no
Cd22	2.48474	1.60946	-0.626514	0.0042	0.0128487	yes
Cd226	76.4138	73.8759	-0.0487299	0.7301	0.836666	no
Cd244	1.5712	1.42865	-0.137212	0.56935	0.714111	no
Cd247	266.146	259.893	-0.0342972	0.8176	0.894764	no
Cd27	175.423	191.292	0.12494	0.4834	0.638927	no
Cd274	10.5048	10.8704	0.0493556	0.7509	0.85116	no
Cd28	52.5749	72.0482	0.454587	0.0015	0.00516454	yes
Cd2ap	3.37213	5.06522	0.586966	7.00E-04	0.00261609	yes
Cd2bp2	51.7157	51.6413	-0.00207662	0.988	0.992966	no
Cd320	1.92048	3.55714	0.889255	0.00025	0.00103712	yes
Cd37	245.766	232.16	-0.0821687	0.5609	0.707194	no
Cd38	5.3881	5.26433	-0.0335257	0.8545	0.917591	no
Cd3d	509.127	539.194	0.0827782	0.5618	0.707861	no
Cd3e	358.165	330.653	-0.115306	0.41845	0.577025	no
Cd3eap	4.36091	4.80496	0.139896	0.6446	0.775145	no
Cd3g	687.898	679.069	-0.0186366	0.8995	0.944432	no
Cd40lg	11.958	22.2926	0.898584	5.00E-05	0.000236281	yes
Cd44	36.766	43.2473	0.234239	0.1005	0.194092	no
Cd46	1.15455	1.27618	0.144509	0.71665	0.827514	no
Cd47	180.728	185.84	0.0402416	0.7791	0.870074	no
Cd48	658.156	596.715	-0.141387	0.3337	0.489631	no
Cd5	243.804	254.096	0.0596535	0.67375	0.797067	no
Cd52	3538.29	2989.95	-0.242932	0.10425	0.199972	no
Cd53	165.954	190.254	0.197141	0.16975	0.295128	no
Cd55	11.8494	20.0132	0.756134	5.00E-05	0.000236281	yes
Cd6	237	252.668	0.092357	0.52115	0.673215	no
Cd68	27.2851	33.1789	0.282153	0.0772	0.155947	no
Cd69	77.5439	99.0046	0.352482	0.01375	0.0360527	yes
Cd7	205.865	322.285	0.646644	5.00E-05	0.000236281	yes
Cd72	18.7523	33.2414	0.825916	0.00055	0.0021091	yes
Cd74	9.21543	10.3702	0.170325	0.3959	0.554972	no
Cd79b	5.25972	15.7399	1.58137	5.00E-05	0.000236281	yes
Cd80	4.13526	3.37488	-0.293142	0.2169	0.357667	no
Cd82	246.925	285.356	0.208693	0.14645	0.262054	no
Cd84	67.208	61.2745	-0.133346	0.35135	0.508708	no
Cd86	10.354	10.5349	0.0249846	0.88095	0.933257	no
Cd8a	561.161	501.936	-0.160911	0.301	0.453424	no
Cd8b1	1370.36	1233.12	-0.152236	0.3285	0.484034	no
Cd9	10.8483	10.3699	-0.0650648	0.7409	0.844367	no
Cd96	129.064	138.756	0.104456	0.4608	0.617919	no
Cd97	296.629	303.606	0.0335391	0.82805	0.901199	no

Cd99l2	4.06089	4.16754	0.0373985	0.83955	0.908044	no
Cdadc1	18.4524	18.2861	-0.0130551	0.93535	0.964864	no
Cdan1	8.33897	8.20823	-0.0227985	0.8798	0.932627	no
Cdc123	47.573	44.2412	-0.104754	0.4777	0.633814	no
Cdc14a	11.0779	10.6843	-0.0521844	0.7308	0.837264	no
Cdc14b	7.42835	8.6107	0.213088	0.23315	0.377107	no
Cdc16	25.6511	24.0472	-0.0931545	0.53015	0.681102	no
Cdc20	31.8309	18.3573	-0.794077	0.015	0.0388553	yes
Cdc20b	6.06003	2.47102	-1.29422	1.00E-04	0.000450026	yes
Cdc23	15.3341	13.3369	-0.201321	0.4552	0.612878	no
Cdc25a	5.12098	4.64907	-0.13948	0.42395	0.582266	no
Cdc25b	132.896	156.182	0.232932	0.1004	0.193942	no
Cdc25c	4.0357	2.26876	-0.830917	0.0014	0.00485767	yes
Cdc26	45.6618	42.829	-0.0923997	0.56855	0.713424	no
Cdc27	14.2948	13.2932	-0.104807	0.47495	0.631305	no
Cdc34	86.3945	75.4234	-0.195927	0.1733	0.300058	no
Cdc37	124.798	111.423	-0.163547	0.2464	0.391548	no
Cdc37l1	13.4446	13.6908	0.0261824	0.87025	0.927043	no
Cdc40	23.2151	23.69	0.0292096	0.8414	0.90932	no
Cdc42	483.844	429.318	-0.172497	0.2404	0.385591	no
Cdc42bpg	3.34943	3.74272	0.160168	0.3499	0.507232	no
Cdc42ep3	41.9933	47.5556	0.179458	0.21375	0.353812	no
Cdc42se1	214.115	224.528	0.068509	0.72065	0.830312	no
Cdc42se2	82.2662	82.6581	0.00685612	0.963	0.980065	no
Cdc45	16.3589	8.77918	-0.89792	5.00E-05	0.000236281	yes
Cdc5l	46.216	42.1441	-0.133062	0.354	0.511621	no
Cdc6	4.37959	2.03479	-1.10592	5.00E-05	0.000236281	yes
Cdc7	11.0853	8.09505	-0.453532	0.00585	0.01717	yes
Cdc73	21.9915	22.2807	0.0188482	0.8966	0.942579	no
Cdca2	7.50938	4.45716	-0.752568	5.00E-05	0.000236281	yes
Cdca3	41.8486	23.797	-0.814396	5.00E-05	0.000236281	yes
Cdca4	29.6977	24.2743	-0.290921	0.0491	0.107098	no
Cdca5	14.6953	7.552	-0.960428	5.00E-05	0.000236281	yes
Cdca7	11.1395	7.72397	-0.528267	0.002	0.0066678	yes
Cdca7l	20.8796	19.3296	-0.111283	0.51375	0.666645	no
Cdca8	39.4533	22.8852	-0.785729	5.00E-05	0.000236281	yes
Cdh24	6.78043	7.30703	0.107907	0.5307	0.68156	no
Cdip1	7.50069	6.9936	-0.100988	0.66055	0.787483	no
Cdipt	75.9838	88.0994	0.213441	0.1313	0.241006	no
Cdk1	19.7184	10.6824	-0.884303	5.00E-05	0.000236281	yes
Cdk10	14.1287	15.2988	0.11479	0.50115	0.655356	no
Cdk11b	62.9106	64.1171	0.0274054	0.84615	0.912062	no
Cdk12	13.1209	14.4408	0.138285	0.36825	0.526854	no
Cdk13	22.3673	21.9143	-0.0295149	0.8353	0.90564	no
Cdk16	16.1929	16.8053	0.0535466	0.72945	0.836165	no
Cdk17	47.7318	47.0489	-0.0207882	0.88515	0.935797	no
Cdk19	17.3106	16.3326	-0.0838982	0.56095	0.707232	no
Cdk2	21.8298	14.261	-0.614217	1.00E-04	0.000450026	yes
Cdk2ap1	83.9966	72.6513	-0.209341	0.1483	0.264727	no
Cdk2ap2	195.185	206.308	0.0799575	0.57185	0.716241	no
Cdk4	102.186	100.627	-0.0221736	0.87295	0.928629	no
Cdk5	22.4891	21.8805	-0.0395815	0.7975	0.882095	no
Cdk5r1	1.2056	1.22695	0.0253205	0.91915	0.955836	no
Cdk5rap1	12.836	5.98092	-1.10176	5.00E-05	0.000236281	yes
Cdk5rap2	7.8696	8.42132	0.0977558	0.52035	0.67244	no
Cdk5rap3	63.9288	62.8989	-0.0234304	0.8644	0.923487	no
Cdk6	4.63746	6.62211	0.513955	0.00735	0.0209531	yes

Cdk7	5.65895	5.54029	-0.0305725	0.87305	0.928694	no
Cdk8	14.3246	13.9371	-0.0395601	0.8019	0.884923	no
Cdk9	35.8197	33.9297	-0.0782028	0.57965	0.722944	no
Cdkal1	5.53856	5.49535	-0.0112989	0.95395	0.975285	no
Cdkl3	2.07517	1.44504	-0.522117	0.06315	0.132129	no
Cdkn1a	1.78726	1.32636	-0.430277	0.1529	0.271415	no
Cdkn1b	59.6764	56.4471	-0.0802608	0.57375	0.717894	no
Cdkn2a	2.44179	1.07529	-1.18322	0.00805	0.0226766	yes
Cdkn2aip	18.8487	16.3644	-0.203904	0.17165	0.297807	no
Cdkn2aipnl	61.0319	55.3143	-0.14191	0.32035	0.47503	no
Cdkn2c	15.571	10.9217	-0.511657	0.0084	0.0235339	yes
Cdkn2d	8.22026	6.77577	-0.2788	0.19345	0.327413	no
Cdkn3	17.3451	9.32178	-0.895852	0.00045	0.0017621	yes
Cdon	4.42532	5.79203	0.388288	0.01455	0.0378466	yes
Cdpf1	21.4835	19.0029	-0.177011	0.3281	0.483646	no
Cdr2	4.38809	4.36814	-0.00657566	0.97525	0.986416	no
Cds2	40.679	41.8986	0.0426186	0.7976	0.882137	no
Cdt1	27.5183	21.0801	-0.384507	0.01165	0.0313023	yes
Cdv3	42.9587	43.5091	0.0183673	0.8971	0.942894	no
Cdyl	6.90748	5.85172	-0.239299	0.1578	0.278488	no
Cdyl2	4.09812	4.1715	0.0256036	0.86425	0.92341	no
Ceacam16	1.42982	1.90154	0.411342	0.1977	0.333176	no
Cebpb	8.90613	9.96923	0.162683	0.3851	0.544091	no
Cebpg	11.3866	10.627	-0.0995931	0.51245	0.665533	no
Cebpz	24.2467	26.3995	0.122721	0.4168	0.575346	no
Cecr5	92.1531	76.5709	-0.267236	0.0605	0.127667	no
Cela1	4.85181	2.73475	-0.827116	0.00425	0.0129847	yes
Celf1	33.7165	31.1207	-0.115581	0.42195	0.580344	no
Celf2	34.2785	36.0299	0.0718896	0.6168	0.753448	no
Celsr1	8.33863	8.59957	0.0444553	0.7556	0.854155	no
Cenpa	66.8012	48.696	-0.456071	0.00195	0.00652174	yes
Cenpb	30.8145	32.0241	0.0555487	0.7043	0.819001	no
Cenpc1	9.89835	8.20134	-0.271329	0.09425	0.184072	no
Cenpe	10.2208	6.23755	-0.712461	5.00E-05	0.000236281	yes
Cenpf	5.72457	3.4733	-0.720862	5.00E-05	0.000236281	yes
Cenph	9.19773	4.8803	-0.914309	2.00E-04	0.0008488	yes
Cenpi	3.88084	2.473	-0.650107	0.002	0.0066678	yes
Cenpj	6.4989	5.84789	-0.152281	0.3562	0.514011	no
Cenpk	4.98969	3.53309	-0.498021	0.2921	0.443205	no
Cenpl	12.6532	8.97871	-0.494925	0.0069	0.0198135	yes
Cenpm	9.95769	5.70314	-0.804055	0.00045	0.0017621	yes
Cenpn	8.31331	4.34527	-0.935976	5.00E-05	0.000236281	yes
Cenpo	7.00433	6.50636	-0.106396	0.703	0.817934	no
Cenpp	6.00507	2.46542	-1.28435	0.01595	0.0409554	yes
Cenpq	19.8749	17.9969	-0.143194	0.3903	0.549381	no
Cenpt	8.40086	9.69321	0.206436	0.26165	0.407794	no
Cenpu	1.64965	1.14445	-0.527512	0.383	0.542078	no
Cenpv	8.18922	11.2745	0.461271	0.03145	0.0734475	no
Cenpw	8.20625	6.16594	-0.412402	0.0425	0.0948293	no
Cep104	7.61356	7.2925	-0.0621573	0.70565	0.819909	no
Cep120	21.2638	20.6512	-0.0421753	0.7697	0.863704	no
Cep128	2.36436	2.02211	-0.225591	0.25145	0.396819	no
Cep135	7.59444	7.67263	0.0147773	0.92575	0.95949	no
Cep152	6.70621	7.10126	0.0825775	0.5956	0.736112	no
Cep164	9.37954	10.9931	0.229013	0.13045	0.239752	no
Cep170	2.33697	1.98069	-0.238636	0.18675	0.318579	no
Cep170b	1.9864	2.67628	0.430071	0.01905	0.0477889	yes

Cep19	9.04131	13.2978	0.556582	0.00225	0.00740543	yes
Cep192	14.386	13.4035	-0.102055	0.4735	0.629923	no
Cep250	26.3194	24.5109	-0.102702	0.4739	0.630289	no
Cep350	19.3233	20.1225	0.0584639	0.67985	0.80169	no
Cep41	4.11524	4.68176	0.186075	0.3118	0.465451	no
Cep44	10.5568	10.1552	-0.0559597	0.76515	0.860664	no
Cep55	14.8387	8.67324	-0.774725	5.00E-05	0.000236281	yes
Cep57	25.3373	24.3465	-0.057548	0.6944	0.812078	no
Cep57l1	5.54937	5.03497	-0.140341	0.47495	0.631305	no
Cep63	12.3469	12.5081	0.0187056	0.9089	0.949752	no
Cep68	8.16214	9.86395	0.273218	0.07745	0.15638	no
Cep70	3.37691	3.46449	0.0369411	0.85885	0.920092	no
Cep72	2.03293	1.88433	-0.109509	0.62355	0.758687	no
Cep76	8.39175	9.57387	0.190131	0.22515	0.367672	no
Cep78	4.83073	4.9703	0.0410899	0.82705	0.900735	no
Cep85	13.2747	12.6667	-0.0676409	0.65385	0.782469	no
Cep89	4.50553	4.2975	-0.0682007	0.72625	0.834163	no
Cep95	17.9261	18.5482	0.0492232	0.7478	0.849091	no
Cep97	17.4307	16.8408	-0.049674	0.84895	0.913842	no
Cept1	19.6245	18.615	-0.0761911	0.75995	0.857192	no
Cercam	11.0545	9.07887	-0.284053	0.08515	0.169235	no
Cerk	7.73794	9.18128	0.246746	0.1173	0.220164	no
Cerkl	20.863	16.9337	-0.301046	0.82115	0.896953	no
Cers2	97.8831	93.1137	-0.0720657	0.66005	0.787086	no
Cers4	12.6038	11.3627	-0.149558	0.32805	0.483603	no
Cers5	47.8805	44.1914	-0.115672	0.42025	0.578739	no
Cers6	1.60927	2.57803	0.679861	0.00235	0.00770045	yes
Cetn2	64.2701	61.2952	-0.0683736	0.63895	0.770741	no
Cetn3	52.3243	51.1582	-0.0325166	0.835	0.905481	no
Cfdp1	64.805	65.4305	0.0138583	0.9209	0.956979	no
Cfl1	1986.59	1702.59	-0.222563	0.16745	0.292115	no
Cfl2	3.93768	3.76582	-0.0643817	0.74265	0.845624	no
Cflar	24.1849	24.1865	9.29E-05	0.9993	0.999342	no
Cfp	3.20726	2.65806	-0.270965	0.3012	0.453677	no
Cggbp1	44.8425	42.1806	-0.0882875	0.53145	0.68225	no
Cgrrf1	6.11231	6.39437	0.0650852	0.7701	0.863974	no
Chac2	1.70556	1.57689	-0.113159	0.8553	0.917975	no
Chaf1a	11.6788	6.93205	-0.752541	5.00E-05	0.000236281	yes
Chaf1b	23.5908	20.8751	-0.176443	0.2557	0.4006	no
Champ1	9.99955	10.6093	0.0853977	0.58205	0.724964	no
Chchd1	63.7172	63.937	0.00496809	0.97625	0.98689	no
Chchd10	2.73581	3.934	0.524027	0.09155	0.179714	no
Chchd2	142.479	135.714	-0.0701748	0.6171	0.753697	no
Chchd3	60.3369	60.7196	0.00912356	0.94775	0.97186	no
Chchd4	7.75109	8.18791	0.0790973	0.70345	0.818282	no
Chchd5	11.2827	11.3724	0.0114318	0.94945	0.972783	no
Chchd6	1.33729	1.02682	-0.381132	0.3671	0.525518	no
Chchd7	23.9531	26.7162	0.157502	0.44045	0.598453	no
Chd1	17.6767	20.5685	0.218584	0.12065	0.2252	no
Chd1l	22.7322	21.9804	-0.0485187	0.74305	0.845862	no
Chd2	28.272	28.2055	-0.00340195	0.9831	0.990255	no
Chd3	50.2886	55.0394	0.130233	0.36335	0.521546	no
Chd4	67.1672	80.7437	0.265591	0.06045	0.127596	no
Chd6	6.08633	7.4979	0.300914	0.0392	0.0884928	no
Chd7	29.4272	27.003	-0.124027	0.3797	0.538661	no
Chd8	32.1562	32.9685	0.0359908	0.79225	0.878781	no
Chd9	4.54649	5.35164	0.235228	0.11395	0.215026	no

Chek1	3.12865	1.41095	-1.14888	5.00E-05	0.000236281	yes
Chek2	4.66837	4.31905	-0.112204	0.58105	0.724166	no
Cherp	34.3006	34.4428	0.00596935	0.96785	0.982494	no
Chfr	56.2955	54.3272	-0.0513456	0.7216	0.830945	no
Chic2	55.4549	51.1033	-0.117899	0.44055	0.598519	no
Chid1	5.81718	6.65255	0.193588	0.26015	0.405896	no
Chit1	1.93539	0.486072	-1.99338	5.00E-05	0.000236281	yes
Chka	1.97227	2.30147	0.2227	0.35095	0.508295	no
Chkb	39.2509	38.5186	-0.0271724	0.86195	0.921972	no
Chm	9.85163	10.4328	0.0826929	0.58415	0.726823	no
Chml	1.50344	1.4014	-0.101401	0.62585	0.760449	no
Chmp1a	86.8024	83.2954	-0.0594984	0.6758	0.798689	no
Chmp1b	39.4221	40.8455	0.0511701	0.8081	0.888874	no
Chmp2a	141.528	127.561	-0.149903	0.29645	0.448158	no
Chmp2b	17.4407	19.1365	0.133873	0.40615	0.565098	no
Chmp3	27.895	22.8508	-0.287758	0.0557	0.119202	no
Chmp4b	172.44	159.904	-0.108886	0.44395	0.601714	no
Chmp5	72.5792	69.0152	-0.0726415	0.61845	0.754706	no
Chmp6	30.4047	26.5215	-0.197137	0.20875	0.347377	no
Chmp7	21.9613	19.9442	-0.138996	0.3501	0.507449	no
Chn2	2.86556	2.74163	-0.0637845	0.7611	0.85793	no
Chordc1	56.6437	57.8276	0.0298421	0.8359	0.905956	no
Chp1	72.5921	65.6325	-0.145403	0.29695	0.44876	no
Chpf	12.5127	12.629	0.0133409	0.9331	0.963486	no
Chpf2	18.6035	18.8292	0.0173975	0.90605	0.948291	no
Chpt1	8.90143	6.19688	-0.522495	0.01165	0.0313023	yes
Chrac1	31.7582	36.046	0.182711	0.2812	0.431012	no
Chrna2	1.39516	1.37464	-0.0213688	0.9281	0.960727	no
Chrnb1	0.7851	1.65308	1.07421	0.00255	0.00827285	yes
Chrne	1.66432	0.996329	-0.740242	0.05975	0.126489	no
Chst10	21.4183	22.9629	0.100458	0.49045	0.645441	no
Chst11	15.5796	15.7325	0.0140837	0.9205	0.956718	no
Chst12	17.8845	15.737	-0.184551	0.25935	0.404979	no
Chst14	1.31514	1.24207	-0.082468	0.7853	0.874329	no
Chst2	2.48075	1.56821	-0.661662	0.00075	0.00278555	yes
Chsy1	139.537	93.3466	-0.579978	5.00E-05	0.000236281	yes
Chtf18	4.20502	2.54344	-0.725333	7.00E-04	0.00261609	yes
Chtf8	66.3269	63.5399	-0.0619308	0.6663	0.79161	no
Chtop	89.1009	86.5624	-0.0416996	0.77165	0.86508	no
Chuk	36.4171	35.4751	-0.0378094	0.7906	0.877861	no
Churc1	95.8109	92.1377	-0.0563991	0.71785	0.828249	no
Ciao1	14.0629	14.568	0.0509054	0.73995	0.843639	no
Ciapin1	8.16954	8.92894	0.128234	0.4084	0.567312	no
Ciart	4.75899	4.35786	-0.127035	0.59215	0.733351	no
Cib1	88.1571	84.0208	-0.0693303	0.63975	0.771311	no
Cib2	1.29081	0.938699	-0.459538	0.39605	0.555039	no
Cic	33.0339	30.9089	-0.0959262	0.49945	0.653678	no
Cinp	6.60126	7.19093	0.123438	0.5142	0.667057	no
Cipc	20.0153	17.1153	-0.225813	0.12045	0.224922	no
Cir1	27.6983	27.5649	-0.00696637	0.96225	0.979811	no
Cirbp	42.2239	46.9244	0.15228	0.3148	0.468621	no
Cirh1a	16.3467	18.1012	0.147083	0.34735	0.504489	no
Cisd1	27.4231	28.2272	0.041695	0.8034	0.8859	no
Cisd2	16.1484	15.6229	-0.0477287	0.75895	0.856543	no
Cisd3	41.3937	44.6353	0.108772	0.5198	0.671902	no
Cish	8.5916	8.89125	0.0494605	0.7747	0.867136	no
Cit	3.69854	2.51418	-0.556869	0.00145	0.00501121	yes

Cited2	74.2504	65.7207	-0.176051	0.2222	0.363924	no
Ciz1	37.8706	37.2807	-0.0226479	0.8756	0.930257	no
Ckap2	10.6144	5.93291	-0.839217	5.00E-05	0.000236281	yes
Ckap2l	13.6317	7.42222	-0.877044	5.00E-05	0.000236281	yes
Ckap5	18.5865	14.5665	-0.351598	0.01575	0.0405023	yes
Ckb	1.46818	1.0851	-0.43619	0.23025	0.373649	no
Cklf	40.5046	36.5684	-0.147488	0.41625	0.574879	no
Cks1b	64.8822	38.5237	-0.752078	5.00E-05	0.000236281	yes
Cks2	129.351	102.463	-0.336186	0.0241	0.0586359	no
Clasp1	6.25943	6.49605	0.0535299	0.7278	0.835088	no
Clasp2	8.53937	7.85213	-0.121046	0.4164	0.575018	no
Clasrp	31.687	30.1058	-0.0738486	0.62055	0.756275	no
Clcc1	17.8623	16.9633	-0.0744984	0.7216	0.830945	no
Clcf1	37.5701	39.332	0.0661161	0.68755	0.807197	no
Clcn3	11.8475	13.0005	0.133984	0.36285	0.521093	no
Clcn4-2	31.6268	30.3427	-0.0597972	0.66765	0.792601	no
Clcn5	1.38998	1.25289	-0.149797	0.5243	0.675998	no
Clcn6	5.98184	5.28964	-0.177419	0.32685	0.482247	no
Clcn7	11.4598	11.7392	0.0347559	0.81865	0.895331	no
Cldn12	1.04382	1.52856	0.550306	0.0262	0.0630025	no
Cldn25	31.5773	44.7502	0.503008	0.00085	0.00311418	yes
Cldnd2	6.56783	3.93178	-0.740234	0.03115	0.0728457	no
Clec12a	0.177819	1.10086	2.63016	0.00085	0.00311418	yes
Clec16a	5.86831	5.48969	-0.0962202	0.5368	0.686932	no
Clec2d	163.545	152.945	-0.0966673	0.4966	0.651131	no
Clec2i	28.0962	31.9413	0.185048	0.2307	0.374147	no
Clec3b	1.92281	1.33353	-0.527966	0.1893	0.322088	no
Clhc1	2.34536	1.23976	-0.919751	0.01335	0.0351488	yes
Clic1	564.479	517.103	-0.126467	0.3751	0.533809	no
Clic3	1.64656	1.07677	-0.612745	0.20455	0.341879	no
Clic4	18.3289	15.1585	-0.273997	0.06595	0.136953	no
Clint1	90.7388	103.4	0.188448	0.1893	0.322088	no
Clip1	17.3229	21.0856	0.28358	0.05045	0.109595	no
Clk1	114.081	120.928	0.0840825	0.567	0.712163	no
Clk2	47.3564	44.7759	-0.0808367	0.5777	0.721175	no
Clk3	43.9013	37.3556	-0.232942	0.10615	0.202944	no
Clk4	49.5	52.0517	0.0725178	0.61615	0.752889	no
Cln3	34.2629	28.3005	-0.275824	0.05945	0.125933	no
Cln5	18.1615	16.4745	-0.140649	0.3683	0.526883	no
Cln6	8.281	8.43111	0.0259182	0.88805	0.93722	no
Cln8	1.13689	1.11077	-0.0335317	0.88	0.932727	no
Clns1a	19.0078	18.6028	-0.0310733	0.83385	0.904707	no
Clock	4.86334	5.03633	0.0504247	0.7385	0.842803	no
Clp1	21.3898	19.7691	-0.113679	0.4642	0.621275	no
Clpb	3.90664	3.95085	0.0162316	0.9263	0.959764	no
Clpp	15.301	15.5547	0.0237226	0.89215	0.939608	no
Clptm1	28.2529	26.7247	-0.0802219	0.57705	0.720733	no
Clptm1l	76.6855	76.0586	-0.0118422	0.93275	0.963294	no
Clpx	15.0409	14.6328	-0.0396779	0.7983	0.882634	no
Clspn	9.30091	5.2656	-0.820772	5.00E-05	0.000236281	yes
Clstn1	11.9163	14.9935	0.331404	0.0257	0.0619838	no
Clta	229.408	214.713	-0.0955035	0.4986	0.652916	no
Cltb	8.73623	8.53278	-0.0339944	0.8557	0.918195	no
Cltc	39.9571	35.707	-0.162244	0.2534	0.3978	no
Cluap1	16.8352	16.9698	0.0114892	0.9478	0.971897	no
Cluh	9.25315	11.933	0.36694	0.01365	0.0358344	yes
Clybl	7.06954	9.37534	0.407255	0.0511	0.110762	no

Cma1	1.98159	0.0656413	-4.91591	0.13725	0.24936	no
Cmah	58.323	60.6292	0.055948	0.69495	0.812483	no
Cmas	58.6636	57.3733	-0.0320861	0.8208	0.896737	no
Cmc1	36.6245	31.5152	-0.216762	0.53385	0.684336	no
Cmc2	17.4515	13.9901	-0.318943	0.0653	0.135827	no
Cmip	39.6016	42.8442	0.113539	0.41955	0.578068	no
Cmklr1	14.0698	7.17666	-0.971223	5.00E-05	0.000236281	yes
Cml1	4.18591	4.32222	0.0462295	0.86635	0.924646	no
Cmpk1	82.5121	67.0477	-0.299417	0.0365	0.0832765	no
Cmpk2	3.30633	5.6324	0.768519	0.00015	0.000653255	yes
Cmss1	3.3944	3.61892	0.0924035	0.84775	0.913147	no
Cmtm3	9.76104	10.1178	0.0517952	0.7732	0.866034	no
Cmtm4	1.54014	1.30767	-0.236063	0.22465	0.367111	no
Cmtm6	49.0702	50.4055	0.0387361	0.7865	0.875057	no
Cmtm7	109.925	163.206	0.570173	0.00015	0.000653255	yes
Cmtr1	45.7837	45.775	-0.000273823	0.998	0.998396	no
Cmtr2	2.45172	2.22962	-0.136996	0.51645	0.669238	no
Cnbd2	1.29217	1.42955	0.145759	0.8486	0.913632	no
Cnbp	174.381	177.188	0.0230387	0.8705	0.927099	no
Cndp2	47.6509	47.9572	0.00924373	0.94965	0.972875	no
Cnep1r1	15.7701	13.0614	-0.271882	0.09815	0.190429	no
Cnga1	1.70112	2.25189	0.404651	0.1038	0.199282	no
Cnih1	45.4468	43.1388	-0.075192	0.61755	0.754025	no
Cnih2	1.11583	1.01703	-0.133752	0.8618	0.921907	no
Cnih4	6.04535	6.42906	0.0887831	0.6103	0.747929	no
Cnn2	643.145	615.283	-0.0638926	0.6695	0.793996	no
Cnnm2	2.27037	1.84705	-0.297707	0.1848	0.315847	no
Cnnm3	6.79329	7.13004	0.0697995	0.6564	0.784205	no
Cnnm4	4.1829	3.53337	-0.243456	0.1713	0.297339	no
Cnot1	46.8062	47.1187	0.00960188	0.9488	0.972343	no
Cnot10	24.162	25.9786	0.104589	0.47255	0.62911	no
Cnot11	20.8559	19.6179	-0.0882842	0.55205	0.699711	no
Cnot2	42.88	42.7781	-0.00343083	0.98155	0.989458	no
Cnot3	46.6586	48.5166	0.0563361	0.71725	0.827787	no
Cnot4	12.8961	12.7383	-0.0177632	0.9074	0.949014	no
Cnot6	31.5984	33.648	0.0906676	0.5296	0.68073	no
Cnot6l	32.0469	35.5552	0.149876	0.28605	0.436493	no
Cnot7	31.9285	34.2902	0.102951	0.5755	0.719421	no
Cnot8	43.0009	41.906	-0.0372123	0.8042	0.886533	no
Cnp	112.659	118.904	0.0778284	0.5862	0.728359	no
Cnppd1	63.4789	63.7261	0.00560711	0.96855	0.982936	no
Cnpy2	28.3918	31.4685	0.148434	0.37275	0.531407	no
Cnpy3	22.3821	21.7596	-0.0406946	0.7982	0.882551	no
Cnpy4	16.6335	15.6125	-0.0913892	0.57545	0.719384	no
Cnr2	0.850728	1.55012	0.865609	0.00175	0.00592472	yes
Cnrip1	3.25331	5.46079	0.7472	0.0024	0.00784462	yes
Cnst	8.66915	8.82518	0.0257355	0.86845	0.926062	no
Cntf	2.49841	2.38418	-0.0675162	0.9566	0.976752	no
Cntrob	11.7073	11.703	-0.00053795	0.99765	0.998173	no
Coa3	68.6094	64.606	-0.0867377	0.5819	0.724854	no
Coa4	16.5742	15.441	-0.102173	0.6004	0.740127	no
Coa5	49.9817	46.2242	-0.112753	0.42515	0.583551	no
Coa6	29.0256	25.9185	-0.163341	0.3666	0.525015	no
Coa7	2.18612	1.907	-0.197074	0.41265	0.571461	no
Coasy	10.6912	11.6566	0.124716	0.45625	0.613777	no
Cobll1	12.0316	11.5873	-0.0542864	0.7189	0.828972	no
Cog1	23.0247	21.9944	-0.0660468	0.7183	0.828605	no

Cog2	21.218	21.485	0.0180389	0.9038	0.946947	no
Cog3	22.4988	22.0489	-0.0291408	0.83745	0.906898	no
Cog4	18.4597	16.6921	-0.145215	0.33765	0.493938	no
Cog5	16.8107	16.0241	-0.069137	0.76795	0.862522	no
Cog6	14.1556	12.2044	-0.213975	0.1708	0.296624	no
Cog7	4.91022	5.75502	0.229035	0.21255	0.35228	no
Cog8	29.4108	29.3533	-0.00282159	0.9843	0.990884	no
Coil	6.78069	7.0525	0.0567024	0.74715	0.848667	no
Col11a2	2.38119	2.03448	-0.227024	0.23015	0.373539	no
Col23a1	0.666031	1.09678	0.719607	0.0054	0.0160104	yes
Col4a3bp	30.4009	27.5352	-0.142839	0.3089	0.462227	no
Colec12	2.86516	2.83128	-0.0171624	0.9344	0.964391	no
Commd1	48.9159	45.2529	-0.112293	0.5746	0.718576	no
Commd10	18.2869	18.1436	-0.0113488	0.94425	0.969778	no
Commd2	13.7441	12.1398	-0.179064	0.25355	0.397956	no
Commd3	69.3882	71.6106	0.0454821	0.7595	0.856972	no
Commd4	90.9217	87.8202	-0.0500717	0.73695	0.841741	no
Commd5	19.0584	18.6658	-0.0300334	0.8703	0.927054	no
Commd6	47.8051	45.1025	-0.0839574	0.59485	0.735636	no
Commd7	22.4501	22.5172	0.00430599	0.97855	0.987999	no
Commd8	29.763	27.7291	-0.10212	0.46835	0.624992	no
Commd9	12.2334	11.6728	-0.0676724	0.721	0.83058	no
Comt	14.3546	15.2226	0.0847039	0.63955	0.771188	no
Comtd1	4.13548	4.5949	0.151977	0.81015	0.890172	no
Copa	102.81	99.3446	-0.0494622	0.75135	0.851397	no
Copb1	62.4342	61.2835	-0.0268363	0.8476	0.913029	no
Copb2	59.4527	59.796	0.00830825	0.95315	0.974933	no
Cope	117.687	117.538	-0.00183817	0.9896	0.993761	no
Copg1	42.7368	41.7111	-0.035049	0.81285	0.891792	no
Copg2	11.9468	10.4515	-0.192915	0.2005	0.336713	no
Cops2	34.6921	30.4875	-0.186391	0.19855	0.334305	no
Cops3	30.6431	27.6021	-0.150781	0.33875	0.495137	no
Cops4	34.5727	29.6382	-0.222178	0.14505	0.26005	no
Cops5	38.7573	36.1281	-0.101346	0.49615	0.650722	no
Cops6	72.4006	69.8256	-0.0522447	0.7232	0.832014	no
Cops7a	18.7171	17.9706	-0.0587209	0.7218	0.83104	no
Cops7b	9.33833	8.78848	-0.0875517	0.6134	0.750568	no
Cops8	36.6204	34.2017	-0.0985825	0.5105	0.66377	no
Copz1	100.685	101.444	0.0108309	0.93685	0.965734	no
Coq10a	6.66913	6.8145	0.0311079	0.86275	0.92238	no
Coq10b	16.265	14.5944	-0.15635	0.34285	0.499629	no
Coq2	16.8447	19.7224	0.227537	0.16	0.281617	no
Coq3	2.64984	2.48964	-0.0899686	0.6907	0.809521	no
Coq4	2.01357	2.51868	0.322915	0.2139	0.353994	no
Coq5	10.4291	10.9756	0.0736864	0.66935	0.793924	no
Coq6	10.146	10.5114	0.0510499	0.78075	0.871049	no
Coq7	6.44712	5.85042	-0.140114	0.59045	0.731926	no
Coq9	22.8415	22.9667	0.00788904	0.9582	0.977478	no
Coro1a	1675.15	1554.92	-0.107453	0.5261	0.677502	no
Coro1b	198.567	182.953	-0.118151	0.4004	0.559392	no
Coro1c	35.448	30.3943	-0.221907	0.1205	0.22498	no
Coro2a	10.999	13.3774	0.282426	0.06155	0.129538	no
Coro7	77.416	87.6882	0.179751	0.22275	0.364597	no
Cotl1	576.828	572.678	-0.0104177	0.942	0.968581	no
Cox10	9.42463	10.9616	0.217949	0.1872	0.319223	no
Cox11	6.00888	6.98074	0.216283	0.2467	0.391884	no
Cox14	45.1362	52.4232	0.21592	0.19685	0.332044	no

Cox15	8.53344	8.68331	0.0251174	0.8754	0.930114	no
Cox16	19.7749	19.25	-0.0388108	0.80555	0.887217	no
Cox17	275.948	264.504	-0.0611085	0.6869	0.806742	no
Cox18	13.4519	13.5996	0.0157545	0.93095	0.96228	no
Cox19	24.2245	21.6032	-0.165222	0.3736	0.532275	no
Cox20	171.439	168.138	-0.0280457	0.8565	0.91869	no
Cox4i1	602.597	568.534	-0.0839454	0.5553	0.702417	no
Cox5a	305.999	286.415	-0.095421	0.5029	0.657036	no
Cox5b	394.177	341.782	-0.205766	0.14825	0.264658	no
Cox6a1	443.706	417.847	-0.0866298	0.5399	0.689474	no
Cox6b1	551.261	497.651	-0.147602	0.29115	0.442096	no
Cox6b2	1.22592	1.63566	0.416015	0.47065	0.62716	no
Cox6c	391.53	370.408	-0.0800082	0.57385	0.717969	no
Cox7a1	2.12699	2.12458	-0.00163073	0.92685	0.960151	no
Cox7a2	143.026	124.779	-0.196904	0.1725	0.298951	no
Cox7a2l	443.172	426.983	-0.0536896	0.70025	0.816057	no
Cox7b	126.745	124.154	-0.0298022	0.83345	0.90444	no
Cox7c	210.814	196.437	-0.101902	0.50315	0.657277	no
Cox8a	548.849	490.098	-0.16334	0.2526	0.396897	no
Cpeb2	3.4856	3.14215	-0.149654	0.3731	0.531756	no
Cpeb4	10.0073	9.54887	-0.0676464	0.636	0.768391	no
Cpm	7.55552	11.5788	0.615881	5.00E-05	0.000236281	yes
Cpne1	64.9791	60.2381	-0.1093	0.48125	0.636932	no
Cpne2	2.57721	2.83409	0.137073	0.56065	0.706942	no
Cpne3	12.9855	14.2154	0.130555	0.3674	0.525862	no
Cpne7	1.03617	0.705364	-0.55482	0.10765	0.205378	no
Cpne8	23.6881	10.8012	-1.13296	5.00E-05	0.000236281	yes
Cpox	14.7155	17.2528	0.229494	0.13255	0.242815	no
Cpped1	5.20927	4.23212	-0.299702	0.1243	0.230598	no
Cpsf1	28.2112	28.7979	0.0297001	0.83865	0.9075	no
Cpsf2	19.8821	18.9169	-0.071796	0.61575	0.752582	no
Cpsf3	41.473	44.882	0.113965	0.42815	0.586437	no
Cpsf3l	31.3274	32.1982	0.0395514	0.8029	0.885625	no
Cpsf4	28.6192	27.1369	-0.076727	0.6144	0.751479	no
Cpsf6	15.7561	14.8388	-0.0865307	0.55205	0.699711	no
Cpsf7	42.9212	40.3308	-0.0898076	0.5292	0.680352	no
Cpt1a	22.5256	22.7176	0.0122432	0.9306	0.962073	no
Cpt1b	1.24815	0.720974	-0.791776	0.4972	0.651651	no
Cpt2	6.54654	5.50809	-0.249182	0.18255	0.312835	no
Cr1l	87.8776	89.5283	0.0268489	0.85225	0.915997	no
Cradd	8.08974	7.57826	-0.0942258	0.6237	0.758764	no
Cramp1l	13.356	13.3614	0.000586188	0.996	0.997158	no
Crat	1.60508	1.83196	0.190747	0.5608	0.707093	no
Crbn	27.1136	25.1661	-0.107536	0.5271	0.678406	no
Crcp	36.2349	35.0425	-0.0482741	0.75235	0.851996	no
Creb1	23.3578	21.2233	-0.138259	0.3328	0.488534	no
Creb3	25.8165	26.2487	0.0239529	0.94455	0.969931	no
Creb3l1	2.32183	1.94505	-0.255457	0.31335	0.467083	no
Creb3l2	1.57478	1.73436	0.139251	0.5711	0.715644	no
Crebbp	13.9108	14.8658	0.0957935	0.4959	0.650539	no
Crebl2	5.65057	5.3195	-0.0871053	0.63665	0.768926	no
Crebrf	9.6052	8.29298	-0.211926	0.1506	0.268107	no
Crebzf	22.1228	20.5609	-0.105629	0.5048	0.658859	no
Creg1	6.57134	8.02317	0.287985	0.11885	0.222523	no
Creld1	2.58335	3.09904	0.262575	0.28325	0.433326	no
Creld2	29.5411	28.9344	-0.0299366	0.852	0.915812	no
Crem	4.52534	3.95482	-0.194415	0.37365	0.532335	no

Crim1	7.29448	8.24102	0.176017	0.31705	0.471176	no
Crip1	1704.48	1749.29	0.037438	0.7941	0.879976	no
Crip2	6.5222	7.18901	0.140434	0.50025	0.654458	no
Cript	53.6698	42.7347	-0.328703	0.03315	0.0767624	no
Crk	11.4685	10.9747	-0.063493	0.6642	0.78994	no
Crkl	31.0116	28.5153	-0.121073	0.39595	0.554976	no
Crlf2	33.5317	32.2322	-0.0570209	0.712	0.824342	no
Crlf3	86.5236	97.0675	0.165894	0.24005	0.385195	no
Crls1	9.51663	8.6477	-0.138134	0.44715	0.604612	no
Crmp1	15.8637	12.2111	-0.377538	0.01515	0.0391806	yes
Crnkl1	25.0169	25.0529	0.00207247	0.98875	0.993395	no
Crocc	2.15895	1.83263	-0.236408	0.20825	0.3467	no
Crot	51.3928	45.862	-0.164266	0.24875	0.39412	no
Crtam	43.2391	36.2096	-0.255964	0.08245	0.164741	no
Crtap	6.97205	7.298	0.065917	0.7351	0.84039	no
Crtc1	4.21907	5.05883	0.261877	0.11205	0.212064	no
Crtc2	33.7871	35.5964	0.0752559	0.60385	0.742771	no
Crtc3	12.6084	14.2498	0.176559	0.22855	0.371634	no
Cry1	21.5757	21.7233	0.00983822	0.9431	0.96919	no
Cry2	2.62233	2.70362	0.0440451	0.81895	0.895575	no
Cryba4	3.36641	4.91363	0.545579	0.0955	0.186116	no
Crybb1	8.02748	9.75636	0.281396	0.2361	0.380562	no
Crybg3	12.5907	12.2949	-0.034296	0.8762	0.930614	no
Cryl1	3.82039	3.73355	-0.0331686	0.8963	0.94239	no
Cryz	0.934702	1.49524	0.677795	0.0595	0.126028	no
Cryzl1	9.35191	11.1895	0.258807	0.15275	0.27123	no
Cs	101.525	92.9503	-0.127302	0.37175	0.530303	no
Csad	2.5054	2.12771	-0.235741	0.3456	0.502589	no
Csde1	80.9113	71.8031	-0.172296	0.21335	0.353307	no
Cse1l	24.1516	22.5333	-0.100059	0.49545	0.650118	no
Csf2ra	1.46145	2.02301	0.469108	0.1133	0.213976	no
Csgalnact2	13.0237	10.7646	-0.274851	0.07165	0.146654	no
Csk	204.389	194.2	-0.0737751	0.60445	0.743289	no
Csnk1a1	119.146	131.439	0.141654	0.31135	0.464976	no
Csnk1d	61.4221	61.2178	-0.00480746	0.97275	0.985017	no
Csnk1e	8.76374	9.93264	0.180631	0.2829	0.432894	no
Csnk1g1	9.4797	9.59831	0.0179385	0.90415	0.947103	no
Csnk1g2	92.5434	92.6885	0.00226052	0.98495	0.991326	no
Csnk1g3	10.7767	10.9375	0.0213749	0.8921	0.939598	no
Csnk2a1	56.24	55.3157	-0.023908	0.86105	0.92154	no
Csnk2a2	14.715	13.6201	-0.111547	0.4634	0.620475	no
Csnk2b	167.028	159.634	-0.0653255	0.64705	0.777085	no
Cspp1	7.77637	7.08765	-0.13379	0.41015	0.569104	no
Csrnp1	25.0303	28.1595	0.169946	0.2447	0.389804	no
Csrnp2	1.69133	1.32598	-0.351102	0.12925	0.237899	no
Csrp1	45.9636	34.4476	-0.416088	0.00565	0.0166536	yes
Csrp2	3.33054	2.92547	-0.187087	0.56065	0.706942	no
Csrp2bp	13.3616	12.8642	-0.0547332	0.7276	0.834994	no
Cst3	110.51	113.451	0.0378888	0.8023	0.885171	no
Cst7	221.846	226.134	0.0276216	0.8478	0.913147	no
Cstb	72.6435	68.5138	-0.0844402	0.60575	0.744356	no
Cstf1	19.4325	20.4804	0.0757713	0.6287	0.762573	no
Cstf2	19.2607	18.399	-0.066032	0.6743	0.797451	no
Cstf2t	12.5738	13.919	0.146646	0.33175	0.487449	no
Cstf3	23.0056	20.3158	-0.179385	0.3048	0.457634	no
Ctage5	41.9215	45.2867	0.111397	0.43145	0.589664	no
Ctbp1	101.109	95.2713	-0.0857936	0.54725	0.695613	no

Ctbs	2.49209	2.71531	0.123762	0.6633	0.789402	no
Ctc1	4.80439	4.27676	-0.167836	0.3383	0.494653	no
Ctcf	64.7081	60.3355	-0.100938	0.47465	0.631073	no
Ctdnep1	68.6542	68.9354	0.00589666	0.97075	0.984008	no
Ctdp1	22.6198	21.6232	-0.0650018	0.65635	0.784158	no
Ctdsp1	85.5271	78.1295	-0.130514	0.3551	0.512728	no
Ctdsp2	22.0027	38.7004	0.814665	5.00E-05	0.000236281	yes
Ctdspl2	8.47537	7.79485	-0.120754	0.4271	0.585533	no
Ctla2a	320.118	344.029	0.103929	0.4601	0.617344	no
Ctla2b	105.661	112.361	0.0886965	0.5522	0.699863	no
Ctla4	13.2905	28.3497	1.09294	5.00E-05	0.000236281	yes
Ctnna1	14.5625	11.9254	-0.288212	0.05995	0.126859	no
Ctnnb1	66.1421	56.1205	-0.23704	0.0966	0.187939	no
Ctnnbip1	5.95641	4.38461	-0.441993	0.02075	0.0515134	no
Ctnnbl1	24.9952	24.8981	-0.00562089	0.9686	0.982972	no
Ctns	5.17279	4.56615	-0.179967	0.32955	0.485176	no
Ctps	8.38617	8.87303	0.0814151	0.6313	0.764846	no
Ctps2	17.0935	17.9562	0.0710399	0.62425	0.759185	no
Ctr9	16.8362	17.1714	0.0284413	0.848	0.913251	no
Ctrl	1.66319	1.27076	-0.388264	0.37835	0.537329	no
Ctsa	128.123	121.691	-0.0742984	0.6113	0.748686	no
Ctsb	50.2781	47.8686	-0.0708511	0.63605	0.768438	no
Ctsc	59.292	47.0802	-0.332717	0.01895	0.0475701	yes
Ctsd	988.541	771.277	-0.358052	0.02345	0.0572656	no
Ctse	9.24265	10.5255	0.187515	0.28105	0.430857	no
Ctso	12.1267	11.1296	-0.123777	0.4241	0.582438	no
Ctss	62.9726	68.3908	0.119079	0.4188	0.57744	no
Ctsw	509.76	552.731	0.116758	0.4177	0.576306	no
Ctsz	27.4994	39.3018	0.515192	0.02185	0.0538879	no
Ctu1	5.96757	5.77201	-0.04807	0.79265	0.879004	no
Ctu2	12.9112	13.931	0.109677	0.7751	0.867419	no
Ctxn1	2.81085	2.43499	-0.207088	0.4905	0.645459	no
Cuedc1	1.88496	1.93776	0.0398601	0.94215	0.968641	no
Cuedc2	22.1046	23.1382	0.065927	0.67965	0.801495	no
Cul1	33.1013	32.2381	-0.0381184	0.79135	0.87828	no
Cul2	8.42059	7.84954	-0.101313	0.5175	0.670021	no
Cul3	84.7741	82.9153	-0.0319845	0.8201	0.896347	no
Cul4a	17.3768	17.4749	0.00811927	0.95755	0.977134	no
Cul4b	14.4533	12.8197	-0.173031	0.2441	0.389085	no
Cul5	8.30513	8.25387	-0.0089323	0.9558	0.976356	no
Cul7	1.2554	1.09459	-0.197756	0.38365	0.542705	no
Cul9	1.07026	1.25668	0.231653	0.25775	0.403015	no
Cuta	115.719	110.813	-0.0624997	0.68135	0.802748	no
Cutc	12.0799	12.1699	0.0107071	0.95535	0.976122	no
Cux1	10.7694	12.0066	0.156892	0.37855	0.537559	no
Cwc15	151.296	148.076	-0.0310305	0.8246	0.899066	no
Cwc22	18.2945	17.8008	-0.039466	0.795	0.880586	no
Cwc25	10.7589	11.6795	0.11845	0.4453	0.602988	no
Cwc27	7.9883	8.21887	0.0410515	0.81715	0.894522	no
Cwf19l1	5.66588	5.06785	-0.160925	0.36215	0.520374	no
Cwf19l2	6.15429	6.35647	0.0466344	0.7807	0.871048	no
Cx3cr1	138.188	48.0045	-1.52539	5.00E-05	0.000236281	yes
Cxcl10	3.49485	3.57806	0.0339508	0.907	0.948831	no
Cxcr3	164.468	203.151	0.304747	0.03335	0.0771723	no
Cxcr4	6.02277	7.9393	0.398586	0.0431	0.0959497	no
Cxcr6	216.051	237.066	0.133913	0.4316	0.589846	no
Cxx1a	2.12926	2.27112	0.0930498	0.77275	0.865736	no

Cxx1b	5.05449	4.34152	-0.219366	0.37735	0.536232	no
Cxx1c	3.27664	3.49802	0.0943206	0.76	0.857221	no
Cxxc1	64.8073	63.686	-0.0251817	0.85875	0.920092	no
Cyb5	99.1005	111.032	0.164007	0.26685	0.414134	no
Cyb561	0.680404	1.17714	0.790826	0.0236	0.0575801	no
Cyb561a3	21.2362	18.4195	-0.205294	0.1666	0.290999	no
Cyb561d1	14.3594	14.5586	0.0198815	0.89295	0.9401	no
Cyb561d2	16.3256	14.8679	-0.134942	0.41375	0.572591	no
Cyb5b	27.962	26.0556	-0.101875	0.47435	0.630792	no
Cyb5d1	12.6466	12.9789	0.0374251	0.8674	0.925375	no
Cyb5d2	2.28752	2.09746	-0.125143	0.61075	0.748259	no
Cyb5r1	12.0852	10.5948	-0.189883	0.2886	0.43932	no
Cyb5r3	17.5799	16.9412	-0.0533906	0.7377	0.842257	no
Cyb5r4	62.2748	67.1263	0.108231	0.4454	0.603054	no
Cyb5rl	2.19089	2.94914	0.428779	0.11525	0.217032	no
Cyba	653.106	578.927	-0.173935	0.2196	0.360746	no
Cyc1	84.9243	87.7859	0.0478118	0.73815	0.84258	no
Cycs	29.9035	29.3323	-0.0278263	0.84335	0.910563	no
Cyfip1	5.37047	5.69876	0.0855972	0.71275	0.824832	no
Cyfip2	101.408	105.287	0.0541646	0.7088	0.822175	no
Cyhr1	39.2528	39.4375	0.00677289	0.96975	0.983653	no
Cyld	25.6798	24.264	-0.0818132	0.5605	0.706804	no
Cyp17a1	1.01143	0.0851652	-3.56999	0.012	0.0320694	yes
Cyp20a1	6.43488	6.25313	-0.0413363	0.8426	0.9101	no
Cyp2s1	1.48409	3.5305	1.25029	5.00E-05	0.000236281	yes
Cyp4f13	5.0743	4.84428	-0.0669253	0.758	0.855758	no
Cyp4f17	1.81145	1.71986	-0.074849	0.80585	0.887396	no
Cyp4v3	5.48317	6.88948	0.329387	0.06545	0.136078	no
Cyp51	6.77796	6.82192	0.0093275	0.95515	0.976045	no
Cysltr2	8.47149	6.50213	-0.381702	0.04795	0.104956	no
Cyth1	72.0804	74.2968	0.0436936	0.7525	0.852029	no
Cyth2	25.7777	26.3666	0.0325899	0.82325	0.898232	no
Cyth3	1.24025	1.5961	0.36392	0.13685	0.249011	no
Cyth4	190.413	171.811	-0.148312	0.30485	0.457671	no
Cytip	78.2887	75.7395	-0.0477593	0.738	0.842491	no
D030056L22Rik	11.9746	12.3849	0.0486055	0.77775	0.869172	no
D10Jhu81e	42.168	48.6035	0.204911	0.1737	0.300588	no
D10Wsu102e	22.2089	22.6317	0.0272079	0.8531	0.916547	no
D11Wsu47e	7.52439	6.9017	-0.124621	0.48565	0.640929	no
D130040H23Rik	1.93235	1.63638	-0.239853	0.3915	0.550576	no
D15Ertd621e	14.6611	14.9009	0.0234091	0.8679	0.925713	no
D16Ertd472e	26.8195	22.3457	-0.263288	0.0663	0.137425	no
D17H6S53E	6.41298	5.88107	-0.124916	0.5082	0.661816	no
D17Wsu104e	54.2343	51.7659	-0.0672049	0.6632	0.789362	no
D17Wsu92e	44.1904	51.821	0.229806	0.1066	0.203677	no
D19Bwg1357e	20.0216	21.8126	0.1236	0.39845	0.557462	no
D1Ertd622e	27.401	21.742	-0.333747	0.02635	0.0633006	no
D230025D16Rik	9.89622	9.69065	-0.0302838	0.86665	0.924869	no
D2hgdh	14.0298	14.7254	0.0698134	0.64825	0.777971	no
D2Wsu81e	14.7847	16.3725	0.147168	0.39315	0.552313	no
D3Ertd751e	3.08629	3.01058	-0.0358316	0.88615	0.936237	no
D430042O09Rik	4.58608	4.65451	0.0213672	0.89505	0.941426	no
D5Ertd579e	6.31162	6.46894	0.0355198	0.8144	0.892563	no
D6Wsu163e	8.5945	8.3504	-0.0415676	0.81035	0.890267	no
D8Ertd738e	174.406	170.628	-0.0315963	0.8301	0.902483	no
D8Ertd82e	26.0174	19.8886	-0.387533	0.00785	0.0221974	yes
D930015E06Rik	37.7324	32.3654	-0.22135	0.11615	0.218406	no

Daam1	1.61498	2.13352	0.401718	0.03955	0.0891973	no
Dad1	224.751	221.066	-0.0238516	0.86865	0.926163	no
Dag1	1.66969	1.95965	0.231009	0.2505	0.396111	no
Daglb	3.87275	4.55573	0.234324	0.20775	0.346088	no
Dak	7.8192	8.44722	0.111455	0.5322	0.682766	no
Dalrd3	31.4336	35.1695	0.162016	0.4138	0.572616	no
Dand5	7.49107	6.36195	-0.235701	0.2695	0.417265	no
Dap	121.25	120.092	-0.0138455	0.92255	0.957904	no
Dap3	15.5554	17.4781	0.16813	0.24785	0.3931	no
Dapk2	44.2756	18.6695	-1.24582	5.00E-05	0.000236281	yes
Dapk3	33.4116	32.1297	-0.0564404	0.7097	0.822759	no
Dapl1	21.0423	20.8338	-0.0143664	0.94965	0.972875	no
Dapp1	22.9515	22.6935	-0.0163056	0.9153	0.953532	no
Dars	46.028	46.9872	0.0297563	0.84055	0.908805	no
Dars2	6.71072	6.35556	-0.0784479	0.6421	0.773219	no
Daxx	40.5974	38.812	-0.064883	0.65285	0.781683	no
Dazap1	61.3768	61.976	0.0140176	0.9222	0.957808	no
Dazap2	330.194	310.164	-0.0902836	0.5272	0.678497	no
Dbf4	31.6904	26.0837	-0.280894	0.0568	0.121163	no
Dbi	110.884	89.2425	-0.313251	0.0435	0.0966968	no
Dbndd2	2.04367	2.24324	0.134422	0.5317	0.68241	no
Dbnl	103.798	102.844	-0.0133281	0.92765	0.960543	no
Dbp	3.24319	3.40674	0.0709772	0.8938	0.940644	no
Dbr1	17.0916	16.1594	-0.0809075	0.61275	0.749954	no
Dbt	8.33579	9.35934	0.167089	0.3088	0.462126	no
Dcaf10	7.51021	6.97162	-0.10736	0.47555	0.631793	no
Dcaf11	32.6288	32.8319	0.00895124	0.9526	0.974596	no
Dcaf12	25.4947	25.6604	0.00934573	0.94825	0.972161	no
Dcaf13	42.5215	41.5225	-0.0342982	0.8199	0.896253	no
Dcaf15	46.2986	49.7247	0.102997	0.47265	0.62916	no
Dcaf17	8.27497	9.35158	0.176457	0.3824	0.541501	no
Dcaf4	10.2944	10.5214	0.0314725	0.856	0.918377	no
Dcaf5	23.5929	24.0918	0.0301871	0.8294	0.902042	no
Dcaf6	6.82353	7.77069	0.187524	0.2604	0.406187	no
Dcaf7	35.3979	35.1208	-0.0113382	0.93545	0.964911	no
Dcaf8	36.2606	38.2951	0.0787599	0.56765	0.712662	no
Dcakd	7.07268	7.23432	0.0326018	0.8653	0.924071	no
Dcdc2c	8.07037	7.44013	-0.117307	0.55395	0.701363	no
Dck	30.7995	30.2679	-0.0251214	0.8627	0.92238	no
Dclk2	2.37833	1.98926	-0.257717	0.21955	0.360689	no
Dclre1a	3.77358	3.74908	-0.00939946	0.9567	0.976752	no
Dclre1b	4.05115	4.63918	0.195538	0.2733	0.421724	no
Dclre1c	10.2135	9.35634	-0.126462	0.4353	0.593375	no
Dcp1a	13.8645	13.8805	0.00165803	0.9907	0.994335	no
Dcp1b	14.577	12.1154	-0.26685	0.07775	0.156865	no
Dcp2	7.35087	8.21373	0.160124	0.27225	0.420434	no
Dcps	69.8356	63.9484	-0.127054	0.5742	0.718254	no
Dctd	1.06462	1.03414	-0.0419128	0.9005	0.94492	no
Dctn1	41.8203	42.27	0.0154295	0.91425	0.953001	no
Dctn2	66.9	70.3175	0.0718778	0.7162	0.827278	no
Dctn3	71.2345	63.8783	-0.157249	0.29995	0.452277	no
Dctn4	18.5334	16.8321	-0.138911	0.3508	0.508161	no
Dctn5	85.1591	79.6372	-0.0967192	0.4942	0.648864	no
Dctn6	73.1559	71.1916	-0.0392673	0.7948	0.880461	no
Dctpp1	46.8717	36.4676	-0.362103	0.0394	0.0888959	no
Dcun1d1	11.1174	10.526	-0.0788683	0.59935	0.739271	no
Dcun1d2	7.43849	8.40659	0.176511	0.4209	0.579374	no

Dcun1d3	4.26069	4.07489	-0.0643243	0.69775	0.814565	no
Dcun1d4	7.61715	7.35503	-0.0505204	0.7472	0.848683	no
Dcun1d5	76.0909	71.3261	-0.0932936	0.52455	0.676234	no
Dcxr	3.80186	3.50085	-0.118999	0.69725	0.814156	no
Dda1	56.767	53.3901	-0.0884818	0.6148	0.751786	no
Ddah2	1.77117	1.44916	-0.289481	0.3959	0.554972	no
Ddb1	73.1215	77.4758	0.0834515	0.5598	0.706287	no
Ddb2	26.5371	34.765	0.389627	0.0215	0.0531323	no
Ddhd1	10.9274	10.5087	-0.056374	0.7102	0.823177	no
Ddhd2	10.5369	11.7696	0.159613	0.2936	0.444993	no
Ddi2	15.4908	15.1793	-0.0293046	0.86485	0.923856	no
Ddit3	20.9015	18.3802	-0.185453	0.62905	0.762841	no
Ddit4	16.2288	14.2578	-0.186803	0.265	0.41186	no
Ddost	73.6638	75.6721	0.0388054	0.78515	0.874258	no
Ddr1	1.39342	1.92242	0.464286	0.0447	0.0989529	no
Ddrgk1	60.9588	61.6554	0.0163948	0.9094	0.950036	no
Ddt	81.5626	84.4818	0.0507316	0.74975	0.85043	no
Ddx1	29.4938	28.3019	-0.0595165	0.6844	0.805052	no
Ddx10	21.4427	22.5003	0.0694585	0.6377	0.769746	no
Ddx11	6.18511	5.75803	-0.103223	0.5362	0.686425	no
Ddx17	143.996	140.973	-0.0306073	0.8376	0.906977	no
Ddx18	32.9463	35.13	0.0925898	0.52865	0.67983	no
Ddx19a	26.7335	26.1938	-0.0294217	0.8377	0.907016	no
Ddx19b	11.9698	12.1703	0.0239648	0.86965	0.926725	no
Ddx20	10.1388	9.71264	-0.061951	0.71345	0.825398	no
Ddx21	53.2753	54.228	0.0255711	0.8557	0.918195	no
Ddx23	51.5288	52.5117	0.0272601	0.8504	0.914663	no
Ddx24	52.2262	53.1375	0.024957	0.86255	0.922292	no
Ddx26b	16.7055	18.2273	0.125778	0.39275	0.551883	no
Ddx27	33.525	37.5803	0.164742	0.2564	0.401376	no
Ddx28	21.9505	25.6856	0.226705	0.14105	0.254541	no
Ddx31	9.08235	8.78155	-0.0485901	0.76635	0.861424	no
Ddx39	72.7444	63.8418	-0.188334	0.68555	0.805733	no
Ddx39b	215.909	210.395	-0.0373173	0.7971	0.881902	no
Ddx3x	79.8308	84.9546	0.0897475	0.53015	0.681102	no
Ddx3y	25.1573	26.4799	0.073917	0.6059	0.74441	no
Ddx41	39.2457	43.0122	0.132213	0.3585	0.516359	no
Ddx42	35.2827	36.5771	0.0519783	0.71855	0.828704	no
Ddx43	1.50189	0.986265	-0.606732	0.05285	0.113922	no
Ddx46	55.0867	57.6823	0.0664231	0.63905	0.770756	no
Ddx47	69.4043	67.8399	-0.0328917	0.81775	0.894804	no
Ddx49	23.0058	22.5282	-0.0302665	0.84915	0.913955	no
Ddx5	653.833	736.303	0.171377	0.3075	0.460698	no
Ddx50	38.0867	37.1161	-0.0372441	0.7931	0.879323	no
Ddx51	5.54477	6.64015	0.260088	0.1114	0.211122	no
Ddx52	32.3676	29.1498	-0.151065	0.2949	0.446407	no
Ddx54	40.5145	43.5044	0.102722	0.4683	0.624949	no
Ddx55	14.2384	15.9139	0.1605	0.30195	0.454525	no
Ddx56	10.3346	12.0833	0.225539	0.1519	0.269959	no
Ddx58	23.7093	22.8029	-0.0562332	0.6969	0.813896	no
Ddx59	6.0344	5.67194	-0.0893697	0.64345	0.774264	no
Ddx6	75.0819	82.7237	0.139835	0.32605	0.481298	no
Deaf1	17.8459	17.1014	-0.0614767	0.72935	0.836091	no
Dear1	3.46217	3.5145	0.0216443	0.98425	0.990848	no
Deb1	22.2804	21.6525	-0.041241	0.85525	0.917949	no
Decr1	4.83201	4.60588	-0.0691458	0.7185	0.828687	no
Decr2	3.50488	3.91528	0.15975	0.4768	0.632977	no

Dedd	17.4538	17.0985	-0.0296751	0.8766	0.930885	no
Dedd2	30.1826	37.0306	0.294999	0.0491	0.107098	no
Def6	134.467	121.78	-0.142971	0.3153	0.469089	no
Def8	6.03507	6.09757	0.0148651	0.93065	0.962082	no
Degs1	53.6342	58.7238	0.130792	0.3614	0.519571	no
Degs2	28.3454	32.053	0.177346	0.49825	0.652591	no
Dek	141.1	107.885	-0.38722	0.01275	0.0337909	yes
Dennd1a	6.85455	7.3595	0.102545	0.51995	0.672046	no
Dennd1b	14.7636	14.5458	-0.0214493	0.8778	0.931626	no
Dennd1c	118.794	122.274	0.0416557	0.77205	0.865283	no
Dennd2d	22.0612	22.7812	0.0463333	0.8052	0.887012	no
Dennd3	3.41001	3.34307	-0.0286022	0.86865	0.926163	no
Dennd4a	14.8495	14.6481	-0.0197008	0.88985	0.938348	no
Dennd4b	26.7843	25.7847	-0.0548734	0.7013	0.816804	no
Dennd4c	23.7954	31.9966	0.427236	0.0019	0.00637506	yes
Dennd5a	26.0378	20.4215	-0.350519	0.01525	0.0394075	yes
Dennd6a	5.3911	5.40854	0.00465834	0.9804	0.988791	no
Dennd6b	2.31752	2.53216	0.127786	0.51935	0.671554	no
Denr	33.2148	33.8333	0.0266165	0.8566	0.918769	no
Depdc1a	12.378	6.63624	-0.899347	5.00E-05	0.000236281	yes
Depdc1b	13.8547	8.32918	-0.734126	5.00E-05	0.000236281	yes
Depdc5	7.96213	8.27518	0.0556368	0.7459	0.847807	no
Dera	34.7929	31.2332	-0.155711	0.29945	0.451629	no
Derl1	46.8682	44.8062	-0.0649094	0.64895	0.778453	no
Derl2	16.1544	13.9249	-0.214265	0.2501	0.39562	no
Desi1	20.483	19.7647	-0.0514998	0.73025	0.836797	no
Desi2	16.9226	17.9453	0.084656	0.57205	0.71639	no
Det1	6.72061	7.86524	0.226899	0.20945	0.348328	no
Dexi	11.1658	11.3705	0.0262025	0.88645	0.936386	no
Dffa	20.01	21.9675	0.134647	0.43315	0.591369	no
Dffb	16.808	16.0091	-0.0702502	0.6627	0.789047	no
Dgat1	16.1091	14.562	-0.145672	0.38595	0.544964	no
Dgcr14	13.01	12.1959	-0.093225	0.55255	0.700168	no
Dgcr2	25.3423	24.0632	-0.0747199	0.60625	0.744672	no
Dgcr6	1.20141	1.82054	0.599635	0.0874	0.172952	no
Dgcr8	15.866	14.9297	-0.0877574	0.64385	0.774573	no
Dgka	267.945	318.421	0.248999	0.0839	0.167217	no
Dgkd	62.4415	59.6215	-0.0666714	0.63975	0.771311	no
Dgke	6.31157	7.57553	0.263347	0.09335	0.182663	no
Dgkh	5.86202	5.92645	0.0157705	0.92325	0.95818	no
Dgkq	8.74895	8.23206	-0.087857	0.6992	0.815516	no
Dgkz	151.373	161.892	0.0969171	0.49965	0.653879	no
Dguok	35.613	40.336	0.179664	0.2528	0.397114	no
Dhcr24	8.00125	7.43812	-0.105288	0.51485	0.667668	no
Dhcr7	19.2283	15.6277	-0.29913	0.05295	0.114106	no
Dhdds	11.5379	10.547	-0.129548	0.41765	0.57626	no
Dhfr	3.70588	2.07948	-0.833594	0.0163	0.0417342	yes
Dhodh	6.84724	7.68355	0.166252	0.35785	0.51577	no
Dhps	30.4696	25.3524	-0.265249	0.17435	0.301432	no
Dhrs1	39.4803	31.5776	-0.322231	0.0338	0.0780192	no
Dhrs11	7.54151	9.005	0.255874	0.199	0.334903	no
Dhrs13	2.09159	3.07803	0.55741	0.03755	0.0853192	no
Dhrs3	1.1525	2.96412	1.36283	0.00015	0.000653255	yes
Dhrs4	15.9558	13.8489	-0.204308	0.27655	0.425566	no
Dhrs7	44.6075	45.2283	0.0199424	0.8946	0.941248	no
Dhrs7b	12.0395	12.5536	0.0603271	0.7321	0.838291	no
Dhx15	105.788	106.576	0.0107145	0.93945	0.967227	no

Dhx16	58.5976	60.1598	0.0379589	0.78865	0.876675	no
Dhx29	11.784	11.3996	-0.0478463	0.7617	0.858274	no
Dhx30	24.2975	25.808	0.0870129	0.5469	0.695368	no
Dhx32	9.97246	9.62621	-0.0509809	0.84075	0.908896	no
Dhx33	6.68912	7.73107	0.208853	0.1841	0.314971	no
Dhx34	11.5236	11.2228	-0.038159	0.8021	0.885061	no
Dhx35	10.5199	10.5268	0.000956017	0.99555	0.997005	no
Dhx36	14.5253	14.2643	-0.0261609	0.85775	0.919612	no
Dhx37	9.94109	9.76806	-0.0253313	0.86395	0.923258	no
Dhx38	28.6362	27.023	-0.0836555	0.5523	0.699965	no
Dhx40	17.9906	16.5792	-0.117876	0.4247	0.583103	no
Dhx57	12.3527	12.2807	-0.00843456	0.9525	0.974593	no
Dhx58	8.91855	7.81407	-0.190734	0.27595	0.424856	no
Dhx8	21.4117	21.8273	0.0277339	0.84885	0.913762	no
Dhx9	66.4027	67.0929	0.0149186	0.9182	0.955228	no
Diablo	28.958	32.1637	0.151469	0.3124	0.466091	no
Diap1	100.252	88.5027	-0.179838	0.2018	0.338397	no
Diap2	2.72146	3.19098	0.229621	0.1722	0.298607	no
Diap3	7.28284	4.60244	-0.662101	0.00015	0.000653255	yes
Dicer1	11.3009	12.5253	0.148411	0.30305	0.455695	no
Dido1	17.6422	17.4011	-0.0198537	0.89455	0.941209	no
Diexf	3.81767	4.83804	0.34173	0.04205	0.0939529	no
Dimt1	6.81781	8.52772	0.322853	0.0695	0.14297	no
Dip2a	3.55626	3.87285	0.123033	0.4635	0.620573	no
Dip2b	8.69882	9.94608	0.193309	0.1774	0.30568	no
Dirc2	2.84849	4.46119	0.647229	3.00E-04	0.00122185	yes
Dis3	6.29488	6.51123	0.0487521	0.8083	0.888984	no
Dis3l	13.3336	12.6925	-0.0710923	0.64125	0.772512	no
Dis3l2	7.67707	8.49794	0.146557	0.37505	0.533748	no
Disc1	1.28584	1.63773	0.348983	0.19985	0.335909	no
Dkc1	19.5382	21.9705	0.169266	0.343	0.499796	no
Dkkl1	6.31621	6.92576	0.132913	0.59645	0.736827	no
Dlat	12.2936	12.008	-0.033905	0.82685	0.900642	no
Dld	37.1327	34.3889	-0.110751	0.4481	0.605548	no
Dlg1	26.3815	27.6796	0.0692937	0.62775	0.761888	no
Dlg3	1.76428	1.98979	0.173531	0.4357	0.593828	no
Dlg4	2.14628	1.19659	-0.842908	0.0012	0.00423153	yes
Dlgap4	30.1776	29.6324	-0.0263033	0.85965	0.92053	no
Dlgap5	14.6531	8.43794	-0.796243	5.00E-05	0.000236281	yes
Dlst	62.203	60.9914	-0.0283793	0.83815	0.90728	no
Dmap1	16.8514	16.7366	-0.0098626	0.9528	0.97473	no
Dmtf1	39.1197	40.7486	0.0588549	0.6938	0.811685	no
Dmwd	1.49128	1.77187	0.248723	0.3471	0.504209	no
Dmxl1	6.33136	6.47712	0.0328379	0.82465	0.899106	no
Dna2	3.61997	2.16585	-0.741048	3.00E-04	0.00122185	yes
Dnaaf2	7.88208	7.38245	-0.0944755	0.62635	0.760795	no
Dnah11	1.19887	0.975388	-0.297631	0.3441	0.500944	no
Dnah8	9.16827	7.2331	-0.342034	0.01735	0.0440075	yes
Dnaja1	56.099	56.6526	0.0141694	0.93185	0.962759	no
Dnaja2	64.4741	63.546	-0.0209171	0.8824	0.934191	no
Dnaja3	16.3503	16.2745	-0.00670532	0.96525	0.98104	no
Dnaja4	9.80844	10.2049	0.0571665	0.7215	0.830898	no
Dnajb1	61.7604	60.1075	-0.039137	0.7835	0.873013	no
Dnajb11	80.0968	75.2951	-0.089188	0.53005	0.68106	no
Dnajb12	20.1277	19.9281	-0.0143845	0.9289	0.96116	no
Dnajb13	4.93236	3.9016	-0.338213	0.1884	0.320843	no
Dnajb14	1.35274	1.06674	-0.342667	0.31145	0.465086	no

Dnajb2	3.00972	4.87872	0.696871	0.0038	0.0117554	yes
Dnajb4	12.6844	11.9166	-0.0900752	0.58055	0.723683	no
Dnajb5	2.85193	3.06304	0.103027	0.64885	0.778413	no
Dnajb6	145.082	144.138	-0.00941854	0.94895	0.972412	no
Dnajb9	39.6325	40.6104	0.0351635	0.80765	0.88856	no
Dnajc1	26.5949	25.4306	-0.0645849	0.7276	0.834994	no
Dnajc10	18.1103	19.4926	0.106114	0.474	0.630386	no
Dnajc11	15.1003	16.8707	0.159942	0.3015	0.454012	no
Dnajc12	1.34315	1.76683	0.39554	0.26395	0.41059	no
Dnajc13	16.4762	16.485	0.000771846	0.99555	0.997005	no
Dnajc14	29.6428	26.585	-0.15707	0.3398	0.49624	no
Dnajc15	441.183	444.675	0.0113757	0.9349	0.964625	no
Dnajc16	5.80794	6.54568	0.172516	0.3022	0.454776	no
Dnajc17	12.2172	11.8656	-0.0421304	0.8924	0.939773	no
Dnajc18	4.18469	4.32494	0.0475564	0.7799	0.870555	no
Dnajc19	40.4643	39.7728	-0.024868	0.94205	0.968604	no
Dnajc2	48.9555	47.4472	-0.0451508	0.75605	0.854486	no
Dnajc21	23.676	21.8382	-0.116572	0.44745	0.604855	no
Dnajc24	10.8313	11.7631	0.119062	0.53425	0.684699	no
Dnajc25	5.45681	5.31965	-0.0367256	0.85855	0.92005	no
Dnajc27	2.63714	2.80787	0.0905063	0.6288	0.762642	no
Dnajc3	43	46.9422	0.126548	0.36755	0.526034	no
Dnajc30	15.1711	15.9394	0.0712734	0.82605	0.900174	no
Dnajc4	10.893	10.3957	-0.0674252	0.7663	0.861395	no
Dnajc5	54.593	53.1895	-0.0375729	0.794	0.87992	no
Dnajc6	0.111004	1.14359	3.36488	5.00E-05	0.000236281	yes
Dnajc7	93.9695	102.572	0.126367	0.369	0.527596	no
Dnajc8	130.179	126.688	-0.0392154	0.7795	0.870328	no
Dnajc9	124.483	121.486	-0.0351668	0.80415	0.886505	no
Dnal4	4.98616	5.9519	0.255423	0.24845	0.393751	no
Dnase1	1.22386	1.58362	0.371786	0.77265	0.865679	no
Dnase1l1	11.5086	11.1298	-0.0482791	0.79145	0.878363	no
Dnase1l2	2.91963	3.23296	0.147068	0.60585	0.74441	no
Dnase2a	2.83105	2.95535	0.061991	0.81115	0.890758	no
Dnlz	9.02253	8.5752	-0.0733624	0.7213	0.830763	no
Dnm1l	28.7792	27.5418	-0.063406	0.66325	0.789382	no
Dnm2	130.01	130.576	0.00626742	0.96625	0.981604	no
Dnmbp	2.33608	2.69131	0.204215	0.2617	0.407845	no
Dnmt1	54.1764	40.6535	-0.414285	0.0036	0.0112159	yes
Dnmt3a	13.6408	14.018	0.0393552	0.78925	0.876955	no
Dnmt3b	4.29846	4.47398	0.0577407	0.75055	0.850913	no
Dnpep	27.2161	26.6456	-0.0305643	0.84455	0.911371	no
Dnttip1	37.6537	38.1553	0.0190909	0.9041	0.947103	no
Dnttip2	35.0389	35.1672	0.00527248	0.97285	0.985048	no
Doc2a	3.57151	3.23115	-0.144484	0.69175	0.810294	no
Doc2g	2.45213	2.53384	0.0472893	0.86405	0.923309	no
Dock10	72.5413	77.4917	0.0952379	0.5025	0.656586	no
Dock11	50.5439	48.7629	-0.0517535	0.71595	0.82703	no
Dock2	216.43	230.563	0.0912589	0.5483	0.696596	no
Dock5	35.6404	31.0211	-0.200265	0.1565	0.276622	no
Dock8	56.661	60.9365	0.104951	0.4646	0.621704	no
Dock9	6.7652	8.50158	0.329599	0.02615	0.0628972	no
Dohh	22.502	22.5206	0.00119238	0.9971	0.997863	no
Dok1	23.4617	22.2387	-0.0772371	0.6145	0.751549	no
Dok2	137.582	134.587	-0.0317546	0.81785	0.894872	no
Dolk	2.65235	3.07069	0.211293	0.3786	0.537576	no
Dolpp1	10.4479	10.3802	-0.00937448	0.9585	0.977656	no

Donson	22.3648	22.2331	-0.008522	0.9526	0.974596	no
Dopey1	5.78062	6.93546	0.262766	0.12755	0.235433	no
Dopey2	19.7863	21.8424	0.142627	0.31875	0.473184	no
Dos	4.32947	4.45099	0.0399348	0.9268	0.960127	no
Dot1l	16.5499	15.1192	-0.130439	0.3614	0.519571	no
Dpagt1	11.5242	11.4652	-0.00740743	0.96375	0.980433	no
Dpcd	20.6156	20.1519	-0.032816	0.88115	0.933399	no
Dpf2	39.5781	40.1875	0.0220442	0.88	0.932727	no
Dph1	6.32344	8.70796	0.461624	0.0978	0.189881	no
Dph2	7.37903	7.48899	0.0213402	0.9051	0.947732	no
Dph3	15.9478	14.7054	-0.117009	0.5274	0.678705	no
Dph5	15.2164	18.31	0.267	0.10855	0.206754	no
Dph6	5.48542	6.34721	0.210521	0.24715	0.392352	no
Dph7	6.23301	6.1106	-0.0286151	0.9075	0.949076	no
Dpm1	17.8707	18.1445	0.021933	0.88545	0.935946	no
Dpm2	68.0363	57.5481	-0.241536	0.11365	0.214534	no
Dpm3	74.6598	68.0427	-0.133891	0.433	0.591233	no
Dpp3	52.7928	49.1522	-0.103085	0.47455	0.630963	no
Dpp4	50.8634	57.7661	0.183596	0.19795	0.333485	no
Dpp7	5.18305	6.26034	0.272439	0.19455	0.328967	no
Dpp8	38.4841	35.3563	-0.122297	0.38135	0.54035	no
Dpp9	35.1016	36.2237	0.0453977	0.7484	0.849445	no
Dpy19l1	32.1663	28.3788	-0.180735	0.1999	0.335953	no
Dpy19l3	7.22392	7.38464	0.0317458	0.8311	0.903098	no
Dpy19l4	3.9619	3.76371	-0.0740362	0.69825	0.814852	no
Dpy30	71.4528	68.4494	-0.0619534	0.6928	0.81104	no
Dpysl2	8.53329	7.58648	-0.16967	0.2824	0.432344	no
Dqx1	4.22389	3.79688	-0.153757	0.417	0.575543	no
Dr1	39.1334	36.7107	-0.0921972	0.52085	0.672902	no
Dram2	8.16695	6.85813	-0.251981	0.1481	0.264424	no
Drap1	121.249	117.902	-0.0403854	0.7856	0.874428	no
Drg1	56.4723	50.7629	-0.153768	0.2882	0.438844	no
Drg2	24.2934	25.9405	0.0946454	0.5353	0.685671	no
Drosha	12.3613	14.2378	0.203896	0.17335	0.30013	no
Dscc1	3.55879	1.94071	-0.874799	0.0046	0.0139281	yes
Dscr3	31.1139	25.586	-0.282205	0.0554	0.11864	no
Dse	6.72513	8.84437	0.395197	0.01355	0.0356024	yes
Dsn1	9.88501	7.68162	-0.363832	0.0386	0.0873254	no
Dstn	43.2083	33.5221	-0.366199	0.0131	0.0345796	yes
Dtd1	15.6698	17.0934	0.125454	0.48	0.635766	no
Dtd2	3.51684	3.61458	0.0395477	0.84175	0.909559	no
Dtl	5.19117	2.8954	-0.842297	1.00E-04	0.000450026	yes
Dtnb	11.343	12.6213	0.154066	0.353	0.510594	no
Dtnbp1	17.6346	15.4194	-0.193654	0.2669	0.414193	no
Dtwd1	4.35144	4.36074	0.00307854	0.9857	0.991728	no
Dtwd2	2.29326	2.2807	-0.00792572	0.9696	0.983591	no
Dtx1	76.6685	83.2355	0.118565	0.40205	0.561064	no
Dtx2	10.5169	9.7458	-0.10986	0.51315	0.66621	no
Dtx3	36.4112	42.8992	0.236571	0.3109	0.464461	no
Dtx3l	26.498	24.5844	-0.108142	0.5557	0.702747	no
Dtymk	60.8416	61.1156	0.00648166	0.96915	0.98329	no
Dus1l	28.8124	28.0375	-0.0393322	0.79735	0.882068	no
Dus2	18.7597	25.4153	0.438063	0.0064	0.018565	yes
Dus3l	22.9668	23.1833	0.0135398	0.9311	0.962421	no
Dus4l	2.92309	3.88357	0.409887	0.0914	0.179479	no
Dusp1	64.5569	75.4239	0.224451	0.1196	0.223654	no
Dusp10	10.707	12.4132	0.213323	0.18855	0.321067	no

Dusp11	51.7625	50.5079	-0.0353988	0.80105	0.884317	no
Dusp12	9.24178	10.5408	0.189736	0.32515	0.480351	no
Dusp14	2.12936	1.606	-0.406946	0.1994	0.33536	no
Dusp19	10.7391	10.104	-0.0879583	0.6377	0.769746	no
Dusp2	337.828	281.395	-0.263692	0.06285	0.131767	no
Dusp22	7.02926	6.21696	-0.177164	0.4626	0.619721	no
Dusp23	1.07024	1.62108	0.599029	0.10375	0.199202	no
Dusp28	3.37989	3.48989	0.0462071	0.8585	0.920038	no
Dusp4	1.61658	7.24736	2.16451	5.00E-05	0.000236281	yes
Dusp5	71.5778	78.2139	0.127912	0.3856	0.544633	no
Dusp6	19.1023	24.3626	0.35092	0.01805	0.0455881	yes
Dusp7	12.5584	17.156	0.450055	0.0033	0.0103917	yes
Dut	34.0291	29.8958	-0.186824	0.20725	0.345443	no
Dvl1	18.3813	20.3716	0.148321	0.31975	0.474399	no
Dvl2	13.5682	14.6663	0.112271	0.46755	0.624314	no
Dvl3	10.6135	11.5562	0.122766	0.4472	0.604668	no
Dxo	21.5873	18.4109	-0.229618	0.16775	0.292509	no
Dym	19.5274	23.9215	0.292807	0.0528	0.113832	no
Dync1h1	51.0244	57.4027	0.16993	0.2498	0.395358	no
Dync1i2	56.658	53.0521	-0.0948719	0.50575	0.659721	no
Dync1li1	40.6482	38.375	-0.0830249	0.56645	0.711713	no
Dync1li2	21.8932	17.9844	-0.283735	0.04925	0.107378	no
Dync2h1	1.03053	1.02918	-0.00189379	0.99305	0.995674	no
Dynll1	103.213	107.354	0.0567462	0.6872	0.806974	no
Dynll2	21.537	17.2443	-0.320699	0.03315	0.0767624	no
Dynlrb1	183.593	170.595	-0.105941	0.46145	0.618544	no
Dynlt1a	3.4675	3.41042	-0.023946	0.92305	0.958146	no
Dynlt1b	9.47227	7.0151	-0.433247	0.08665	0.171709	no
Dynlt3	4.88756	7.62214	0.641083	0.00095	0.00343861	yes
Dyrk1a	22.7062	21.6712	-0.0673094	0.6357	0.768246	no
Dyrk1b	8.22202	7.35355	-0.161051	0.36005	0.518052	no
Dyrk2	20.7463	21.6872	0.0639902	0.67965	0.801495	no
Dyx1c1	4.98259	4.06268	-0.294465	0.18255	0.312835	no
Dzip1	4.72434	5.21368	0.142189	0.3953	0.554418	no
Dzip3	3.89518	4.18806	0.104591	0.53455	0.684984	no
E030030I06Rik	3.25007	3.47356	0.0959421	0.74685	0.848518	no
E130012A19Rik	3.83356	3.83345	-4.35E-05	0.9893	0.993664	no
E130308A19Rik	1.49521	1.3051	-0.196191	0.41745	0.576063	no
E130309D02Rik	24.7357	22.4123	-0.142301	0.34645	0.503524	no
E130311K13Rik	4.88452	5.41476	0.148681	0.5084	0.661942	no
E2f1	5.93312	3.15959	-0.909055	5.00E-05	0.000236281	yes
E2f2	40.3353	30.931	-0.38299	0.00735	0.0209531	yes
E2f3	3.72987	3.34512	-0.157066	0.3841	0.543156	no
E2f4	79.8563	79.2921	-0.0102287	0.93985	0.96747	no
E2f5	6.19471	7.00527	0.177405	0.56115	0.707371	no
E2f6	7.26226	7.97996	0.135963	0.43775	0.595736	no
E2f7	2.42441	1.25834	-0.946112	5.00E-05	0.000236281	yes
E2f8	10.7405	5.72423	-0.907903	5.00E-05	0.000236281	yes
E330009J07Rik	1.62672	2.29685	0.497697	0.07355	0.149692	no
E430018J23Rik	1.36308	1.76641	0.373952	0.1907	0.323845	no
E430025E21Rik	23.0502	22.6836	-0.023132	0.8741	0.929334	no
E4f1	23.0071	25.4089	0.143255	0.33975	0.496208	no
Eaf1	21.8354	24.0243	0.137827	0.3387	0.495105	no
Eapp	61.3153	55.7713	-0.136725	0.36325	0.521455	no
Ears2	3.6735	4.6314	0.334291	0.1322	0.242362	no
Ebag9	16.4175	15.6981	-0.0646512	0.69995	0.815865	no
Ebna1bp2	22.8264	23.3458	0.032461	0.83065	0.902789	no

Ebp	43.9267	39.2677	-0.161754	0.2821	0.431988	no
Ebpl	8.63391	8.12894	-0.0869476	0.63275	0.766038	no
Ecd	18.9355	17.9266	-0.0789968	0.5998	0.739645	no
Ece1	6.33246	8.01267	0.339517	0.032	0.0745101	no
Ece2	5.67891	7.8097	0.459651	0.06115	0.128823	no
Ech1	153.217	141.343	-0.11637	0.41225	0.571075	no
Echdc1	1.19836	1.47858	0.303142	0.6634	0.789494	no
Echdc2	3.95025	8.26653	1.06534	5.00E-05	0.000236281	yes
Echs1	40.4406	42.3514	0.0666051	0.66005	0.787086	no
Eci1	21.3696	30.5369	0.514994	0.0028	0.00898766	yes
Eci2	30.5085	29.0153	-0.0723979	0.6419	0.773031	no
Ecm1	2.31234	2.12936	-0.118934	0.6508	0.77997	no
Ecsit	24.6922	22.4847	-0.135112	0.48345	0.638957	no
Ect2	7.67326	4.5421	-0.756481	5.00E-05	0.000236281	yes
Edaradd	1.31022	2.19956	0.747406	5.00E-05	0.000236281	yes
Edc3	13.2802	13.4523	0.0185657	0.9024	0.946084	no
Edc4	20.9287	22.3145	0.0924962	0.52565	0.677169	no
Edem1	57.6594	54.3362	-0.0856422	0.5474	0.695716	no
Edem2	27.9923	26.7373	-0.0661744	0.65765	0.785193	no
Edem3	10.3306	10.4734	0.019801	0.892	0.939573	no
Edf1	281.364	287.293	0.0300874	0.82915	0.901867	no
Edrf1	7.74387	7.96817	0.0411948	0.78755	0.875839	no
Eea1	4.27374	4.11196	-0.0556735	0.7198	0.829685	no
Eed	40.4491	36.4615	-0.149733	0.30485	0.457671	no
Eef1a1	6882.89	7150.19	0.0549674	0.8959	0.942106	no
Eef1b2	353.797	363.244	0.0380193	0.7906	0.877861	no
Eef1d	127.954	125.145	-0.0320178	0.82195	0.897424	no
Eef1e1	19.3152	22.6933	0.23253	0.18445	0.31541	no
Eef1g	504.728	501.24	-0.0100045	0.9427	0.968976	no
Eef2	1709.61	1599.65	-0.0959103	0.66415	0.789894	no
Eef2k	0.998929	2.17224	1.12073	5.00E-05	0.000236281	yes
Eefsec	8.45718	8.17191	-0.0495032	0.76695	0.861824	no
Efcab14	11.2288	11.1541	-0.0096275	0.9485	0.972248	no
Efcab2	1.12276	0.974232	-0.204709	0.5104	0.663714	no
Efcab4b	28.4381	24.5137	-0.214234	0.19785	0.333349	no
Efcab7	2.43852	3.40908	0.483376	0.0577	0.122768	no
Efemp2	1.92285	1.54082	-0.319548	0.3062	0.45917	no
Efhd2	196.649	178.92	-0.136314	0.3429	0.499692	no
Efr3a	34.4854	34.1092	-0.0158269	0.9092	0.949897	no
Eftud1	7.95739	8.62972	0.11702	0.46595	0.622896	no
Eftud2	45.481	47.0678	0.049479	0.72975	0.836428	no
Egfl8	1.06633	0.787234	-0.437789	0.562	0.708012	no
Egln1	27.6022	24.6083	-0.165638	0.2476	0.392801	no
Egln2	23.2869	23.9701	0.0417131	0.785	0.87416	no
Egr1	41.4758	38.4917	-0.107722	0.45715	0.614683	no
Egr2	0.796956	1.10216	0.467766	0.1237	0.22966	no
Ehbp1l1	46.3759	41.7917	-0.150157	0.305	0.457798	no
Ehd1	24.8924	32.4988	0.384681	0.0081	0.022802	yes
Ehd3	57.8955	60.1732	0.0556708	0.6954	0.812803	no
Ehd4	8.41661	7.28706	-0.207901	0.20775	0.346088	no
Ehhadh	2.76371	2.78711	0.0121598	0.9509	0.973675	no
Ehmt1	15.6657	16.0307	0.033228	0.816	0.893637	no
Ehmt2	71.4566	68.7155	-0.0564312	0.69165	0.810231	no
Ei24	37.9558	39.3774	0.0530478	0.71565	0.826832	no
Eid1	27.0901	28.6765	0.0821028	0.57355	0.717733	no
Eid2	2.40758	3.16142	0.392989	0.17395	0.300925	no
Eid2b	2.11475	2.15623	0.0280242	0.90705	0.948831	no

Eid3	0.953389	1.03865	0.123578	0.7315	0.83774	no
Eif1	646.547	639.815	-0.0150991	0.91965	0.956173	no
Eif1a	13.6438	14.0609	0.0434417	0.7765	0.868297	no
Eif1ad	32.7781	31.3436	-0.0645623	0.775	0.867362	no
Eif1ax	73.5969	69.0058	-0.0929286	0.53855	0.688248	no
Eif1b	65.6427	62.5706	-0.069149	0.6532	0.781943	no
Eif2a	39.796	33.6341	-0.2427	0.3221	0.477003	no
Eif2ak1	39.4627	36.5735	-0.109692	0.48445	0.639895	no
Eif2ak2	7.16152	7.49633	0.065918	0.6795	0.801444	no
Eif2ak3	9.87058	10.3632	0.0702658	0.64395	0.774667	no
Eif2ak4	3.09431	3.28473	0.0861578	0.6228	0.758009	no
Eif2b1	19.59	21.5658	0.138627	0.3782	0.537202	no
Eif2b2	40.4455	40.761	0.0112119	0.9398	0.967432	no
Eif2b3	8.11227	8.62522	0.0884557	0.64895	0.778453	no
Eif2b4	25.8797	25.8629	-0.000940662	0.99925	0.999321	no
Eif2b5	30.9229	30.6397	-0.013275	0.92495	0.958971	no
Eif2d	28.6375	26.4402	-0.115169	0.45085	0.60831	no
Eif2s1	37.4536	35.6356	-0.0717876	0.6173	0.75385	no
Eif2s2	56.3894	51.1688	-0.140159	0.33065	0.486298	no
Eif2s3x	55.694	53.7702	-0.0507152	0.7249	0.833142	no
Eif2s3y	65.3058	50.8228	-0.361736	0.0129	0.0341475	yes
Eif3a	89.7446	89.8947	0.00241076	0.9855	0.991597	no
Eif3b	57.2223	59.1496	0.0477909	0.73525	0.840507	no
Eif3c	121.805	122.174	0.00437054	0.97485	0.986224	no
Eif3d	111.528	108.754	-0.0363445	0.802	0.884978	no
Eif3e	323.351	311.323	-0.0546912	0.69725	0.814156	no
Eif3f	460.104	443.28	-0.0537421	0.7101	0.823088	no
Eif3g	106.091	119.464	0.171268	0.2282	0.371279	no
Eif3h	486.312	456.041	-0.0927187	0.5135	0.66648	no
Eif3i	184.249	175.665	-0.0688264	0.62785	0.761896	no
Eif3j1	21.4517	14.3727	-0.577762	0.38215	0.541201	no
Eif3j2	25.1659	30.8603	0.294285	0.5249	0.676524	no
Eif3k	338.842	322.204	-0.0726389	0.6058	0.744365	no
Eif3l	121.15	114.997	-0.0752027	0.5977	0.737907	no
Eif3m	225.869	213.125	-0.0837896	0.5534	0.700969	no
Eif4a1	230.295	229.442	-0.00535127	0.97015	0.98381	no
Eif4a2	200.256	207.201	0.0491845	0.74785	0.849094	no
Eif4a3	97.0422	99.7438	0.0396152	0.7809	0.871147	no
Eif4b	132.263	134.381	0.0229115	0.8705	0.927099	no
Eif4e	25.8762	24.262	-0.0929234	0.5266	0.677923	no
Eif4e2	67.2498	59.6188	-0.173761	0.24335	0.388214	no
Eif4e3	13.6223	11.5809	-0.234223	0.1464	0.261978	no
Eif4ebp1	13.6351	14.5856	0.0972163	0.6119	0.749213	no
Eif4ebp2	97.7061	104.058	0.0908683	0.51225	0.665396	no
Eif4ebp3	10.3551	9.29293	-0.15613	0.43905	0.597091	no
Eif4enif1	30.1785	30.9169	0.0348769	0.8102	0.890214	no
Eif4g1	75.0128	80.4849	0.10158	0.4649	0.62194	no
Eif4g2	156.311	151.427	-0.045796	0.7612	0.85793	no
Eif4g3	42.3994	44.5766	0.0722436	0.60625	0.744672	no
Eif4h	143.911	130.204	-0.144407	0.30485	0.457671	no
Eif5	75.3827	88.1722	0.22609	0.11205	0.212064	no
Eif5a	328.679	332.524	0.0167814	0.90595	0.948214	no
Eif5a2	3.28763	2.53164	-0.376973	0.0433	0.096322	no
Eif5b	14.9298	15.7011	0.072674	0.6233	0.758448	no
Eif6	65.3913	62.4834	-0.0656254	0.6523	0.78121	no
Elac1	4.68824	5.0128	0.0965702	0.56165	0.707735	no
Elac2	15.0331	13.9082	-0.11221	0.46575	0.622735	no

Elavl1	14.8709	16.1217	0.116503	0.41425	0.573014	no
Elf1	90.0111	97.5565	0.116135	0.435	0.593115	no
Elf2	19.9443	19.4861	-0.0335266	0.82195	0.897424	no
Elf4	99.3758	91.3406	-0.121638	0.39625	0.555276	no
Elk1	5.33311	6.17128	0.210593	0.2183	0.359237	no
Elk3	23.2006	22.0959	-0.0703874	0.6304	0.763992	no
Elk4	16.7902	18.8047	0.163467	0.26795	0.415421	no
Ell	15.4232	15.8138	0.0360747	0.8125	0.891603	no
Ell2	2.70731	2.93419	0.116103	0.5576	0.704468	no
Elmo1	34.157	32.8941	-0.0543537	0.69935	0.815556	no
Elmo2	19.5792	17.7539	-0.141186	0.33975	0.496208	no
Elmo3	1.8643	1.69909	-0.133876	0.58035	0.723536	no
Elmod2	5.99031	5.72071	-0.0664369	0.6849	0.805385	no
Elmod3	6.17461	6.98824	0.178582	0.33095	0.486618	no
Elmsan1	13.1409	14.3489	0.126881	0.3841	0.543156	no
Elof1	37.2481	33.6185	-0.147911	0.35275	0.510337	no
Elovl1	55.1697	46.6371	-0.242398	0.1877	0.319913	no
Elovl5	46.6768	55.6728	0.254269	0.0757	0.153364	no
Elovl6	2.17186	3.79583	0.805484	5.00E-05	0.000236281	yes
Elovl7	3.48731	2.77111	-0.331649	0.06515	0.135551	no
Elp2	29.1581	30.9919	0.0879961	0.54945	0.697593	no
Elp3	15.2963	16.5082	0.110002	0.47595	0.632182	no
Elp4	2.34253	2.42574	0.05036	0.80925	0.889668	no
Elp5	22.3018	19.7226	-0.177312	0.58915	0.730905	no
Elp6	4.59885	4.38603	-0.0683562	0.745	0.847261	no
Emb	84.4283	145.335	0.783583	5.00E-05	0.000236281	yes
Emc1	5.06781	5.91167	0.222203	0.16365	0.286804	no
Emc10	57.4285	58.9866	0.0386208	0.78595	0.874679	no
Emc2	60.1669	56.8508	-0.0817892	0.58165	0.724632	no
Emc3	49.3108	49.3407	0.000875563	0.996	0.997158	no
Emc4	44.8086	44.5428	-0.008583	0.9787	0.988093	no
Emc6	33.0229	31.6114	-0.0630192	0.6861	0.806191	no
Emc7	51.1495	48.4884	-0.0770813	0.60885	0.746696	no
Emc8	7.01528	6.0569	-0.211922	0.18625	0.317879	no
Emc9	10.3408	11.4359	0.14523	0.87415	0.92936	no
Emd	50.5002	45.8973	-0.137881	0.34325	0.500026	no
Eme1	6.32294	4.08546	-0.630096	0.144	0.258529	no
Eme2	4.9911	6.66899	0.41811	0.0553	0.118465	no
Emg1	178.85	161.182	-0.150054	0.3326	0.488301	no
Eml2	21.6548	27.1806	0.327889	0.02995	0.0704362	no
Eml3	79.6518	85.9538	0.109854	0.4339	0.592198	no
Eml4	12.5795	13.4646	0.09809	0.50495	0.658982	no
Eml5	3.73437	4.26995	0.193353	0.4878	0.642865	no
Emp1	26.7784	30.2613	0.176406	0.2295	0.372801	no
Emp3	365.689	304.967	-0.261967	0.0654	0.135991	no
Enc1	7.38464	6.15388	-0.26303	0.0976	0.189576	no
Endod1	15.3129	14.8701	-0.0423416	0.7729	0.865808	no
Endog	4.32267	4.21888	-0.0350607	0.942	0.968581	no
Endov	7.42063	8.47716	0.19204	0.2115	0.350977	no
Engase	2.8235	3.07145	0.121432	0.52815	0.679435	no
Enkd1	4.13669	4.09451	-0.014786	0.95385	0.975253	no
Eno1	249.906	239.58	-0.0608814	0.67435	0.797483	no
Eno3	7.27282	6.92359	-0.0709952	0.7272	0.834752	no
Enoph1	10.8323	12.5489	0.212225	0.2219	0.363585	no
Enox2	2.29151	2.53274	0.144403	0.47435	0.630792	no
Enpp1	2.6865	3.23505	0.268061	0.19515	0.329721	no
Enpp4	1.63096	1.50339	-0.117496	0.5945	0.735319	no

Ensa	60.6054	57.7369	-0.0699529	0.6317	0.765147	no
Enthd2	12.7135	13.0511	0.037809	0.81025	0.890227	no
Entpd1	4.66296	1.77344	-1.3947	5.00E-05	0.000236281	yes
Entpd4	18.0877	23.6617	0.387544	0.0092	0.0255019	yes
Entpd5	19.5494	26.9783	0.464673	0.00085	0.00311418	yes
Entpd6	17.0903	17.0471	-0.00364743	0.98165	0.989458	no
Entpd7	1.18398	1.15196	-0.0395571	0.92265	0.95797	no
Eny2	11.5881	10.7936	-0.10247	0.60805	0.746026	no
Eogt	6.82057	7.68825	0.172763	0.30765	0.460884	no
Eomes	31.4723	21.442	-0.553645	2.00E-04	0.0008488	yes
Ep300	29.6469	30.0972	0.0217507	0.8823	0.934191	no
Ep400	31.1159	31.7779	0.0303709	0.82615	0.900213	no
Epas1	4.9313	4.49371	-0.134061	0.4231	0.581506	no
Epb4.1	61.3991	58.5076	-0.0695924	0.62515	0.759913	no
Epb4.1l2	10.3157	8.50931	-0.277734	0.07255	0.148156	no
Epc1	36.3439	35.4545	-0.0357436	0.8552	0.917923	no
Epc2	14.0181	13.1822	-0.0886959	0.55745	0.704329	no
Epg5	5.85866	6.44748	0.138164	0.35215	0.509605	no
Ephb6	1.30936	1.04494	-0.325441	0.20165	0.338193	no
Ephx1	5.77275	5.74674	-0.00651632	0.9711	0.984179	no
Epm2aip1	6.10689	6.42452	0.0731511	0.63395	0.766885	no
Epn1	65.0557	62.3659	-0.0609174	0.66655	0.791814	no
Eprs	24.0622	27.0592	0.169354	0.2399	0.385016	no
Eps15	25.0084	23.9097	-0.064815	0.6451	0.775548	no
Eps15l1	47.9988	53.1754	0.14776	0.3062	0.45917	no
Eps8l1	19.3171	6.28136	-1.62073	5.00E-05	0.000236281	yes
Epsti1	406.89	316.87	-0.360746	0.0133	0.0350342	yes
Ept1	6.02398	6.57266	0.12576	0.41355	0.572404	no
Eral1	10.8518	11.1141	0.0344608	0.8381	0.907254	no
Erap1	43.9648	44.0099	0.0014824	0.99095	0.994458	no
Erbb2	0.36358	1.24258	1.773	0.04435	0.0982922	no
Erbb2ip	44.6338	45.1344	0.0160914	0.9106	0.950853	no
Erbb3	1.33391	2.13456	0.678277	0.0036	0.0112159	yes
Erc1	1.18248	1.04432	-0.179261	0.3839	0.542982	no
Ercc1	22.5951	21.0173	-0.104436	0.6118	0.749116	no
Ercc2	12.9521	13.925	0.104489	0.52255	0.674419	no
Ercc3	30.9844	29.2507	-0.0830679	0.5709	0.715483	no
Ercc4	8.01621	8.32102	0.0538411	0.7248	0.833108	no
Ercc5	10.7161	10.6534	-0.00846961	0.9552	0.976068	no
Ercc6	8.2418	8.84395	0.101732	0.48465	0.640016	no
Ercc6l	2.6766	1.71935	-0.638535	0.00285	0.00913278	yes
Ercc6l2	7.92032	8.2893	0.0656913	0.6734	0.79684	no
Ercc8	4.61615	5.2132	0.175481	0.38995	0.549063	no
Erf	5.82768	4.95282	-0.23467	0.1852	0.316416	no
Ergic1	9.27595	11.2169	0.274106	0.09945	0.192444	no
Ergic2	23.9958	23.4455	-0.0334723	0.8459	0.911907	no
Ergic3	62.2495	62.7709	0.0120342	0.9361	0.965285	no
Erh	149.888	130.776	-0.196789	0.1721	0.298456	no
Eri1	14.2138	12.3646	-0.201077	0.1721	0.298456	no
Eri2	6.05632	5.94075	-0.0277957	0.87335	0.928845	no
Eri3	7.57649	15.96	1.07486	5.00E-05	0.000236281	yes
Erich1	11.2198	13.7447	0.292826	0.09445	0.184376	no
Erlec1	9.12773	8.96292	-0.0262871	0.87065	0.927217	no
Erlin1	2.88658	3.8369	0.41058	0.0397	0.0894841	no
Erlin2	6.84657	6.14327	-0.156376	0.34795	0.505142	no
Ermard	10.0764	8.75827	-0.202264	0.2763	0.425311	no
Ermp1	15.6423	15.3064	-0.0313154	0.8267	0.900576	no

Ern1	9.8585	9.11761	-0.112713	0.46685	0.62371	no
Ero1l	17.883	21.9745	0.297241	0.03875	0.0876254	no
Ero1lb	4.73153	4.91648	0.055319	0.74735	0.848799	no
Erp27	2.04821	2.66488	0.379705	0.2815	0.43134	no
Erp29	79.2003	77.0779	-0.0391872	0.78405	0.873405	no
Erp44	43.0773	39.9288	-0.109497	0.4441	0.601871	no
Errfi1	22.7411	18.4911	-0.298476	0.0433	0.096322	no
Esco1	26.9177	27.9808	0.0558814	0.69435	0.812046	no
Esco2	8.30193	4.16697	-0.994446	5.00E-05	0.000236281	yes
Esd	65.4258	59.9067	-0.127143	0.36965	0.528225	no
Esf1	8.49375	8.58055	0.0146688	0.928	0.96068	no
Esm1	34.4647	19.2779	-0.838174	5.00E-05	0.000236281	yes
Espl1	7.4671	4.15065	-0.84721	5.00E-05	0.000236281	yes
Esrra	17.6989	18.7067	0.0798915	0.74985	0.850448	no
Esyt1	345.628	345.252	-0.00157053	0.9925	0.99532	no
Esyt2	71.3463	81.5842	0.193451	0.1738	0.300717	no
Etaa1	8.92405	9.09822	0.0278857	0.86125	0.92164	no
Etf1	45.4033	46.867	0.045775	0.7471	0.848652	no
Etfa	50.0638	46.1513	-0.117398	0.4288	0.587077	no
Etfb	109.881	102.342	-0.102548	0.47165	0.628137	no
Etfdh	20.9473	19.7693	-0.0835023	0.5873	0.729379	no
Ethe1	9.80984	8.86896	-0.145464	0.4367	0.594697	no
Etnk1	30.336	29.1381	-0.0581278	0.681	0.80247	no
Etohi1	9.81774	9.93856	0.0176458	0.9317	0.962703	no
Ets1	349.8	330.332	-0.082614	0.60335	0.742402	no
Ets2	3.6415	3.57115	-0.0281475	0.8832	0.934631	no
Etv3	10.9315	11.389	0.0591574	0.6877	0.807305	no
Etv6	10.5302	13.3054	0.337488	0.02235	0.0549252	no
Eva1b	31.996	28.198	-0.182299	0.2487	0.39405	no
Evi2a	38.6355	40.5801	0.0708444	0.83265	0.903961	no
Evi2a-evi2b	2.25582	1.47996	-0.608092	0.56415	0.709897	no
Evi2b	69.3944	64.5622	-0.104129	0.5183	0.670724	no
Evi5l	2.55401	2.32087	-0.138096	0.6161	0.752867	no
Evl	118.001	134.146	0.185007	0.2833	0.433383	no
Ewsr1	208.243	211.282	0.0209038	0.8882	0.937305	no
Exd2	14.4292	14.7247	0.0292415	0.8504	0.914663	no
Exo1	1.37978	0.715356	-0.947703	4.00E-04	0.00158415	yes
Exo5	7.41582	7.9407	0.0986596	0.59275	0.733862	no
Exoc1	19.5148	17.1167	-0.189167	0.2069	0.345006	no
Exoc2	26.6312	24.4679	-0.122229	0.39415	0.553288	no
Exoc3	20.8122	20.415	-0.0277992	0.8455	0.911753	no
Exoc4	14.8046	15.9727	0.109555	0.47005	0.626668	no
Exoc5	22.0035	22.7564	0.0485403	0.7779	0.869229	no
Exoc6	19.1221	19.5332	0.0306882	0.84175	0.909559	no
Exoc6b	3.85737	3.81937	-0.0142827	0.9349	0.964625	no
Exoc7	19.3076	19.1341	-0.0130245	0.9314	0.962533	no
Exoc8	3.5159	3.30888	-0.0875497	0.59575	0.736272	no
Exog	2.2409	2.20484	-0.0234063	0.912	0.951738	no
Exosc1	14.2907	14.7911	0.0496534	0.77265	0.865679	no
Exosc10	37.3977	38.3989	0.0381178	0.7894	0.877095	no
Exosc2	20.5463	22.1305	0.107157	0.5022	0.65634	no
Exosc3	49.5125	46.4975	-0.0906393	0.55885	0.705605	no
Exosc4	19.1102	17.631	-0.116226	0.4706	0.627105	no
Exosc5	36.3821	38.6081	0.085677	0.58745	0.72954	no
Exosc6	17.3871	17.5688	0.0149988	0.9346	0.964438	no
Exosc7	36.7595	35.3877	-0.0548681	0.73425	0.839704	no
Exosc8	61.1532	56.3144	-0.118925	0.4258	0.584193	no

Exosc9	24.7827	23.2127	-0.0944175	0.76015	0.857308	no
Ext1	1.6506	2.70434	0.712285	0.00215	0.00711112	yes
Ext2	24.5356	25.6456	0.0638362	0.667	0.792141	no
Extl2	2.38705	2.75122	0.204844	0.3491	0.506416	no
Extl3	9.02142	10.5599	0.227175	0.12935	0.238021	no
Eya2	6.12929	11.1753	0.866518	5.00E-05	0.000236281	yes
Eya3	16.1399	14.6818	-0.136602	0.3507	0.508069	no
Ezh1	26.3266	27.0236	0.0377012	0.7923	0.878781	no
Ezh2	46.9362	36.2902	-0.371121	0.00975	0.0268451	yes
Ezr	179.96	175.519	-0.0360517	0.80115	0.884386	no
F2r	65.8114	38.3771	-0.778092	5.00E-05	0.000236281	yes
F2rl1	13.283	16.5679	0.318811	0.0419	0.0936534	no
F2rl2	11.8743	7.95628	-0.577673	0.3262	0.481479	no
F2rl3	9.47927	8.31603	-0.188881	0.30875	0.462081	no
F8a	7.25093	7.53622	0.0556757	0.75215	0.851893	no
Faah	23.6609	44.5717	0.913621	5.00E-05	0.000236281	yes
Fabp5	7.21616	5.99652	-0.267105	0.27225	0.420434	no
Fadd	3.88832	4.83053	0.313033	0.10665	0.203745	no
Fads1	5.21332	3.90302	-0.417611	0.0225	0.0552381	no
Faf1	14.2737	13.2529	-0.10705	0.4764	0.632625	no
Faf2	17.6751	17.0566	-0.0513886	0.72795	0.835151	no
Fah	2.71844	1.84795	-0.556851	0.05275	0.113755	no
Fahd2a	6.833	6.15703	-0.150283	0.50675	0.660574	no
Faim	34.7722	33.6712	-0.0464202	0.7743	0.866867	no
Fam101b	1.93098	3.57746	0.889599	5.00E-05	0.000236281	yes
Fam102a	45.0273	60.0722	0.415898	0.0036	0.0112159	yes
Fam102b	5.9643	4.94717	-0.269749	0.0939	0.183495	no
Fam103a1	74.9279	68.3999	-0.131507	0.35835	0.516238	no
Fam104a	17.2976	16.5309	-0.0654068	0.7881	0.876216	no
Fam105a	76.291	69.5062	-0.134371	0.337	0.493202	no
Fam107b	307.269	346.907	0.175051	0.23395	0.377968	no
Fam109a	1.90652	2.28976	0.264256	0.29995	0.452277	no
Fam109b	1.08624	0.71648	-0.60034	0.0659	0.136877	no
Fam110a	6.20513	5.72756	-0.115541	0.55935	0.705971	no
Fam111a	37.4032	35.911	-0.0587353	0.68155	0.80285	no
Fam114a2	35.9037	34.0453	-0.0766757	0.59535	0.735997	no
Fam117a	77.349	64.5734	-0.260443	0.06675	0.138199	no
Fam117b	13.1266	14.5037	0.143934	0.32395	0.47905	no
Fam118a	9.04669	9.03364	-0.00208266	0.9887	0.993358	no
Fam118b	12.692	11.4082	-0.153853	0.7129	0.824965	no
Fam120a	70.3807	71.5883	0.0245424	0.8592	0.920258	no
Fam120b	11.5596	12.9936	0.168714	0.2545	0.399163	no
Fam120c	1.60525	1.86021	0.212667	0.2522	0.396819	no
Fam122a	9.5657	10.6404	0.153604	0.39935	0.558367	no
Fam122b	5.40749	4.78188	-0.177383	0.3082	0.461414	no
Fam126a	10.513	9.04467	-0.217037	0.1519	0.269959	no
Fam126b	2.33103	2.2815	-0.0309879	0.8589	0.920102	no
Fam129a	36.1357	48.6752	0.429764	0.0024	0.00784462	yes
Fam129b	8.45204	10.6763	0.337036	0.0602	0.127251	no
Fam129c	3.71847	5.15923	0.472445	0.01815	0.0458046	yes
Fam132a	1.1844	0.90068	-0.395067	0.33575	0.49176	no
Fam133b	14.017	13.3483	-0.0705277	0.666	0.79138	no
Fam134a	33.8203	35.5775	0.0730732	0.61005	0.747674	no
Fam134b	18.8445	28.0132	0.571962	5.00E-05	0.000236281	yes
Fam134c	61.7331	62.7501	0.0235743	0.8675	0.925454	no
Fam136a	9.50902	11.7247	0.302181	0.10015	0.193517	no
Fam13b	38.6425	40.8845	0.0813647	0.57165	0.716105	no

Fam149b	24.0614	23.2134	-0.0517652	0.736	0.841092	no
Fam151b	1.40095	0.98463	-0.508755	0.1892	0.321964	no
Fam160a2	12.1076	14.3976	0.249913	0.08335	0.166299	no
Fam160b1	16.6733	16.5879	-0.00741144	0.959	0.977954	no
Fam160b2	18.4063	18.788	0.0296074	0.8366	0.906367	no
Fam161a	1.19049	0.980703	-0.279669	0.411	0.569914	no
Fam162a	48.128	54.2184	0.171908	0.3058	0.458716	no
Fam168a	6.6547	5.53569	-0.26561	0.08555	0.169868	no
Fam168b	62.6997	60.8492	-0.0432196	0.75945	0.856929	no
Fam169b	30.2969	43.3726	0.517614	0.00035	0.0014053	yes
Fam172a	18.2082	17.0879	-0.0916115	0.53	0.681045	no
Fam173a	51.1737	47.6411	-0.103195	0.5366	0.686776	no
Fam173b	11.9054	11.7206	-0.0225762	0.9063	0.948468	no
Fam174a	9.40843	10.6951	0.184919	0.28255	0.432527	no
Fam174b	3.08904	6.5917	1.09349	5.00E-05	0.000236281	yes
Fam175a	5.42116	5.55193	0.0343873	0.8556	0.918143	no
Fam175b	11.1182	10.596	-0.0694078	0.66725	0.792259	no
Fam178a	9.06732	9.41051	0.0535967	0.714	0.825791	no
Fam179b	10.2924	11.8588	0.204377	0.1629	0.285738	no
Fam185a	4.85489	4.78512	-0.0208822	0.9105	0.950791	no
Fam188a	22.905	22.2634	-0.0409925	0.78115	0.871302	no
Fam189b	47.9079	52.2947	0.126402	0.3726	0.531258	no
Fam192a	42.1481	39.5453	-0.0919616	0.5332	0.683651	no
Fam193a	12.8108	12.4867	-0.0369772	0.7993	0.88327	no
Fam193b	36.8139	34.8537	-0.0789409	0.5832	0.72601	no
Fam195a	1.74058	2.5276	0.538199	0.17855	0.307256	no
Fam195b	60.8723	59.3863	-0.0356552	0.8054	0.887094	no
Fam199x	2.19924	2.49007	0.179183	0.3094	0.462838	no
Fam19a3	3.27849	2.5196	-0.379836	0.05	0.108782	no
Fam203a	9.0947	10.3488	0.186365	0.29275	0.443981	no
Fam207a	26.6564	26.3227	-0.0181698	0.90265	0.946135	no
Fam209	1.13215	0.941433	-0.26614	0.652	0.780984	no
Fam20b	11.9181	12.4452	0.0624376	0.67675	0.79937	no
Fam21	28.933	29.4827	0.0271491	0.85305	0.916547	no
Fam210a	2.86041	3.24314	0.181171	0.25995	0.405637	no
Fam210b	3.78918	4.50943	0.251057	0.16545	0.289334	no
Fam212a	1.01172	1.35711	0.423727	0.3395	0.495935	no
Fam212b	1.04011	0.954659	-0.123681	0.6181	0.754461	no
Fam213a	2.59566	3.90265	0.588352	0.02445	0.0593789	no
Fam214a	16.3673	12.8777	-0.345934	0.01905	0.0477889	yes
Fam214b	8.48393	7.77454	-0.125975	0.44985	0.607297	no
Fam216a	10.29	12.1189	0.236013	0.2556	0.400479	no
Fam219a	4.56106	4.52864	-0.0102929	0.95985	0.978369	no
Fam219b	10.5198	11.9578	0.184852	0.24535	0.39046	no
Fam220a	11.0158	10.7591	-0.0340113	0.83835	0.907358	no
Fam222b	3.50142	3.51601	0.00600124	0.97665	0.987026	no
Fam26f	13.9891	16.4575	0.234439	0.2013	0.337719	no
Fam32a	79.8661	76.4871	-0.0623662	0.65695	0.784596	no
Fam35a	5.06751	5.55051	0.131344	0.45305	0.610601	no
Fam3a	13.4628	12.4571	-0.112005	0.5128	0.665853	no
Fam3c	14.0094	14.6389	0.0634158	0.68025	0.801948	no
Fam45a	13.7107	11.997	-0.192631	0.22795	0.370975	no
Fam46a	7.93584	7.91061	-0.00459378	0.9758	0.986676	no
Fam46c	10.6788	17.3903	0.70354	5.00E-05	0.000236281	yes
Fam49a	32.9584	25.8446	-0.350785	0.0141	0.0368458	yes
Fam49b	71.0685	66.0874	-0.104835	0.45905	0.616416	no
Fam50a	34.5379	34.1467	-0.0164363	0.914	0.952853	no

Fam53a	24.6397	26.3034	0.0942624	0.53255	0.683041	no
Fam53b	27.541	24.604	-0.162684	0.2585	0.403946	no
Fam53c	16.3514	15.8357	-0.0462324	0.75175	0.851658	no
Fam57a	1.3305	1.83407	0.463084	0.2637	0.410265	no
Fam58b	33.4634	30.1369	-0.15105	0.33725	0.493465	no
Fam60a	17.0751	18.0682	0.0815598	0.59505	0.735806	no
Fam63a	17.3154	16.5582	-0.0645104	0.6809	0.802406	no
Fam63b	13.7997	12.7052	-0.119218	0.40515	0.564141	no
Fam64a	11.2388	6.46469	-0.79783	5.00E-05	0.000236281	yes
Fam65a	7.13535	6.84925	-0.0590374	0.718	0.828395	no
Fam65b	151.999	155.601	0.0337887	0.82505	0.89939	no
Fam65c	1.10838	1.08721	-0.0278304	0.9231	0.958146	no
Fam69a	23.0626	21.0827	-0.129491	0.88135	0.933555	no
Fam69b	0.983552	1.3478	0.454537	0.20765	0.345954	no
Fam71b	9.86878	8.68456	-0.184419	0.295	0.446511	no
Fam72a	2.32031	1.43376	-0.694514	0.0133	0.0350342	yes
Fam73a	4.02009	5.45596	0.440605	0.00775	0.0219463	yes
Fam73b	2.33708	2.73563	0.227169	0.28155	0.43138	no
Fam76a	19.1154	18.5927	-0.0400025	0.7923	0.878781	no
Fam76b	20.0263	18.2419	-0.134636	0.3607	0.518786	no
Fam78a	70.8647	84.9911	0.262245	0.06865	0.141502	no
Fam78b	1.68678	1.79634	0.0907858	0.66125	0.787998	no
Fam83d	2.42298	1.43074	-0.760021	0.00615	0.0179275	yes
Fam84b	2.674	2.82541	0.0794636	0.68785	0.807455	no
Fam86	12.2143	12.9477	0.0841284	0.7015	0.816875	no
Fam89a	3.56143	3.72589	0.0651265	0.7998	0.883559	no
Fam89b	92.6179	74.2729	-0.318454	0.0279	0.0663781	no
Fam92a	24.2442	22.5018	-0.107598	0.50975	0.663088	no
Fam96a	76.4771	72.1831	-0.0833663	0.56355	0.709382	no
Fam96b	60.0701	53.5953	-0.164541	0.31315	0.466894	no
Fam98a	13.6093	13.1776	-0.0465018	0.7657	0.861063	no
Fam98b	32.5475	32.6936	0.00645956	0.9674	0.982263	no
Fam98c	21.0475	20.6994	-0.0240601	0.8986	0.943838	no
Fan1	2.9852	3.02389	0.0185737	0.9245	0.958814	no
Fanca	4.09622	3.01028	-0.444392	0.21675	0.357462	no
Fancb	1.47758	1.19721	-0.303569	0.2565	0.401514	no
Fancc	3.63079	3.01498	-0.268133	0.53445	0.684918	no
Fancd2	2.90646	1.90272	-0.611198	0.00245	0.0079866	yes
Fance	11.5877	10.7154	-0.112902	0.51215	0.665278	no
Fancf	5.04314	3.88944	-0.37476	0.09415	0.183907	no
Fancg	10.858	10.7208	-0.0183544	0.90815	0.949429	no
Fanci	3.60143	2.21595	-0.700643	0.12705	0.234713	no
Fancl	8.25095	6.31848	-0.384982	0.2137	0.353754	no
Fancm	3.51339	2.91397	-0.269878	0.1076	0.205299	no
Far1	31.0315	29.2639	-0.0846137	0.55635	0.703279	no
Far2	2.22276	1.9308	-0.203151	0.6921	0.810556	no
Farp1	3.00459	3.332	0.149218	0.4148	0.573583	no
Fars2	11.1924	11.228	0.00457983	0.9778	0.987609	no
Farsa	32.7309	33.2934	0.0245849	0.86905	0.926349	no
Farsb	22.3851	24.4672	0.128309	0.39745	0.556515	no
Fas	30.5699	27.1178	-0.172867	0.36155	0.519723	no
Fasl	22.1389	13.8052	-0.681369	5.00E-05	0.000236281	yes
Fasn	12.4163	15.0073	0.273433	0.05305	0.11429	no
Fastk	31.7871	33.1341	0.0598786	0.69335	0.811428	no
Fastkd1	4.52906	5.65473	0.320249	0.0851	0.169169	no
Fastkd2	4.89882	6.15688	0.329764	0.06255	0.131251	no
Fastkd3	5.92081	6.11983	0.047696	0.7955	0.880877	no

Fastkd5	3.45769	3.46865	0.00456482	0.9856	0.99167	no
Fau	2806.67	2938.87	0.0664033	0.65375	0.782363	no
Fbf1	3.75354	4.21019	0.165633	0.3353	0.491284	no
Fbl	79.6415	88.8573	0.15797	0.26905	0.416769	no
Fbrs	53.2842	57.4317	0.108139	0.4347	0.592843	no
Fbrsl1	7.70209	7.89121	0.0349964	0.8514	0.915404	no
Fbxl12	19.8388	18.7503	-0.0814107	0.79525	0.880766	no
Fbxl14	29.5586	31.9706	0.113167	0.4272	0.585602	no
Fbxl15	3.89507	3.20033	-0.283428	0.5663	0.711626	no
Fbxl17	1.93409	2.37843	0.298357	0.05465	0.117268	no
Fbxl18	2.86243	3.09887	0.1145	0.586	0.728239	no
Fbxl19	5.27068	4.80946	-0.132114	0.4609	0.618018	no
Fbxl2	5.58679	3.85306	-0.536014	0.30275	0.455399	no
Fbxl20	9.42025	9.32135	-0.0152268	0.91315	0.952493	no
Fbxl22	2.97465	2.60058	-0.193887	0.8071	0.888135	no
Fbxl3	24.7593	25.6216	0.0493894	0.72755	0.834964	no
Fbxl4	5.17428	5.36281	0.051631	0.78275	0.872521	no
Fbxl5	25.2833	25.3273	0.0025085	0.9872	0.992544	no
Fbxl6	38.7359	40.5419	0.0657419	0.65905	0.786332	no
Fbxl8	16.2313	14.0026	-0.213081	0.19675	0.331892	no
Fbxo11	24.4098	24.3082	-0.00601723	0.97095	0.984084	no
Fbxo18	21.2851	20.7029	-0.040006	0.78505	0.874202	no
Fbxo21	6.57837	6.56	-0.00403343	0.97975	0.988568	no
Fbxo22	31.4451	34.5689	0.136639	0.3674	0.525862	no
Fbxo25	13.8565	11.5238	-0.265938	0.1119	0.211888	no
Fbxo27	1.11531	2.1365	0.937804	0.00315	0.00997458	yes
Fbxo28	16.211	15.033	-0.108838	0.4531	0.610657	no
Fbxo3	24.5628	24.1109	-0.0267911	0.87905	0.932336	no
Fbxo30	2.15659	2.28355	0.0825245	0.6759	0.798741	no
Fbxo31	10.5035	9.62054	-0.126681	0.40455	0.563472	no
Fbxo32	3.88776	3.80561	-0.0308108	0.84925	0.913955	no
Fbxo33	20.4684	21.2247	0.052347	0.722	0.831176	no
Fbxo34	8.23454	7.67291	-0.101915	0.54855	0.696814	no
Fbxo38	22.1225	23.6057	0.0936222	0.508	0.661629	no
Fbxo4	4.41771	4.93328	0.159246	0.37965	0.538601	no
Fbxo42	10.0187	9.47927	-0.079849	0.5952	0.735889	no
Fbxo44	1.4954	1.18427	-0.336535	0.3176	0.471844	no
Fbxo45	7.76464	7.75117	-0.00250503	0.9894	0.99368	no
Fbxo46	11.3213	11.0335	-0.0371502	0.81385	0.892265	no
Fbxo48	2.38858	2.00661	-0.25139	0.32915	0.484749	no
Fbxo5	24.7191	13.7365	-0.847614	5.00E-05	0.000236281	yes
Fbxo6	13.6368	16.27	0.254705	0.16365	0.286804	no
Fbxo7	18.2004	19.9246	0.130582	0.4104	0.569351	no
Fbxo8	7.89177	7.51526	-0.0705255	0.6658	0.79123	no
Fbxo9	26.1243	27.0414	0.0497775	0.74655	0.8483	no
Fbxw11	16.5313	16.9125	0.0328892	0.81765	0.894764	no
Fbxw17	9.8415	9.55527	-0.0425823	0.80675	0.887929	no
Fbxw2	43.8984	39.9542	-0.135822	0.3486	0.505857	no
Fbxw4	17.8369	15.526	-0.200181	0.31455	0.468378	no
Fbxw5	26.3189	26.756	0.0237663	0.87285	0.928593	no
Fbxw7	14.7358	14.4682	-0.0264399	0.8599	0.920728	no
Fbxw8	10.1456	11.499	0.18065	0.2374	0.382072	no
Fbxw9	1.43437	1.9724	0.459537	0.60445	0.743289	no
Fcer1g	1.62344	2.03876	0.328641	0.4807	0.636407	no
Fcf1	78.7386	74.8285	-0.073484	0.63365	0.766641	no
Fcgr2b	18.102	12.957	-0.48242	0.00555	0.0163959	yes
Fcgr3	1.12989	1.67984	0.572143	0.1231	0.228751	no

Fcgrt	36.8017	0.107374	-8.42098	0.0058	0.017041	yes
Fcho1	76.1513	71.5859	-0.0891931	0.52345	0.675247	no
Fcho2	17.3222	18.4204	0.0886882	0.5372	0.687294	no
Fchsd1	10.0499	9.40799	-0.0952218	0.5394	0.689047	no
Fchsd2	11.3914	15.1797	0.414197	0.00545	0.0161396	yes
Fcrl1	1.10158	0.991356	-0.152105	0.6529	0.781717	no
Fdft1	10.712	10.4088	-0.0414186	0.91845	0.955375	no
Fdps	21.4796	21.9517	0.0313674	0.8505	0.914715	no
Fdx1	9.17261	9.48812	0.0487887	0.8276	0.900973	no
Fdx1l	17.9007	17.1039	-0.065689	0.91545	0.953604	no
Fdxacb1	6.09523	5.62882	-0.114849	0.5616	0.707698	no
Fdxr	20.1241	18.5091	-0.12069	0.45295	0.610536	no
Fech	8.90691	8.48883	-0.0693605	0.67035	0.79467	no
Fem1a	9.33474	9.65107	0.0480795	0.74495	0.847218	no
Fem1b	7.17863	6.25525	-0.198642	0.1973	0.332692	no
Fem1c	16.7956	18.7377	0.157854	0.30395	0.456727	no
Fen1	20.2134	16.6999	-0.275474	0.0809	0.162085	no
Fermt3	126.85	117.261	-0.113395	0.4175	0.576109	no
Fes	1.17629	1.57622	0.422219	0.1192	0.223072	no
Fez2	11.5116	9.97455	-0.206765	0.45725	0.614736	no
Fgd3	22.6542	22.7641	0.00698523	0.96275	0.980023	no
Fgd6	1.61515	1.81651	0.169494	0.3596	0.517584	no
Fgf13	43.9644	45.4375	0.0475451	0.7457	0.847675	no
Fgfr1op	6.96233	7.62056	0.130328	0.43895	0.597024	no
Fgfr1op2	85.868	84.474	-0.023612	0.86545	0.924119	no
Fgl2	12.3518	5.45449	-1.1792	5.00E-05	0.000236281	yes
Fgr	5.0927	6.04354	0.246964	0.1605	0.282286	no
Fh1	32.9177	33.3815	0.0201823	0.89265	0.939939	no
Fhit	0.741227	1.13512	0.614853	0.21855	0.359573	no
Fhl2	34.8145	31.046	-0.165283	0.27535	0.424182	no
Fhl3	7.25175	8.28123	0.191517	0.3205	0.475212	no
Fhod1	18.2543	18.514	0.0203818	0.887	0.936686	no
Fibp	44.388	40.8467	-0.119948	0.4341	0.592357	no
Ficd	3.62744	3.44367	-0.0750077	0.7011	0.816679	no
Fig4	12.9465	12.2054	-0.0850429	0.5842	0.726847	no
Fignl1	9.07964	4.59187	-0.983553	5.00E-05	0.000236281	yes
Filip1l	7.21086	9.73266	0.432662	0.0123	0.0327694	yes
Fip1l1	50.0153	46.5822	-0.102589	0.48075	0.636449	no
Fis1	168.36	153.857	-0.129959	0.36515	0.523501	no
Fitm2	0.981661	1.25439	0.353686	0.32865	0.484184	no
Fiz1	13.9132	13.7328	-0.018827	0.90715	0.948893	no
Fkbp10	1.9895	0.207879	-3.25859	0.1031	0.198186	no
Fkbp11	1.01413	0.36286	-1.48276	0.074	0.150438	no
Fkbp15	26.1044	26.382	0.0152619	0.914	0.952853	no
Fkbp1a	104.385	110.922	0.0876206	0.53415	0.684621	no
Fkbp2	45.4857	37.9406	-0.261673	0.13365	0.244449	no
Fkbp3	130.387	134.796	0.0479688	0.80985	0.890037	no
Fkbp4	64.5647	69.0792	0.0975064	0.4942	0.648864	no
Fkbp5	41.7273	50.2104	0.266994	0.05825	0.123767	no
Fkbp7	1.44516	1.74479	0.271824	0.5135	0.66648	no
Fkbp8	168.785	169.35	0.00481723	0.97465	0.986135	no
Fkbpl	2.75545	2.79528	0.0207067	0.937	0.965818	no
Fkrp	12.2912	13.4998	0.135312	0.63275	0.766038	no
Fktn	1.14339	1.41162	0.304033	0.24415	0.389129	no
Flad1	27.6259	27.5813	-0.00232766	0.9874	0.992674	no
Flcn	14.8478	14.6651	-0.0178572	0.905	0.947698	no
Fli1	40.3002	43.0797	0.0962193	0.49755	0.651977	no

Flii	75.3944	69.1375	-0.124988	0.42685	0.585258	no
Flna	273.808	258.788	-0.0813963	0.6255	0.760155	no
Flnb	13.4652	15.351	0.189098	0.17795	0.30644	no
Flot1	62.4711	69.9194	0.162504	0.2492	0.394612	no
Flot2	23.0983	20.5415	-0.169243	0.2574	0.402575	no
Flt3l	100.971	61.6584	-0.711568	5.00E-05	0.000236281	yes
Flywch1	9.90834	11.7181	0.242022	0.14685	0.262617	no
Fmnl1	302.34	301.092	-0.0059632	0.969	0.983223	no
Fmnl3	3.61123	4.11935	0.189925	0.2843	0.434499	no
Fmo5	1.79358	1.57719	-0.185486	0.3717	0.530274	no
Fmr1	23.5934	21.8428	-0.111222	0.43925	0.597283	no
Fn3k	1.22602	2.81961	1.20152	0.00505	0.0151078	yes
Fn3krp	3.01897	2.87644	-0.0697731	0.7726	0.865678	no
Fnbp1	110.539	111.868	0.0172452	0.90575	0.948089	no
Fnbp4	39.6013	43.3055	0.129003	0.36425	0.522519	no
Fndc3a	8.34293	9.68097	0.214598	0.1529	0.271415	no
Fndc9	6.94503	6.15336	-0.174609	0.8833	0.934706	no
Fnip1	5.00451	5.44246	0.12103	0.4439	0.60167	no
Fnta	37.9983	34.2062	-0.151679	0.30645	0.459486	no
Fntb	5.21279	5.59518	0.102128	0.58785	0.729857	no
Focad	4.76584	6.11815	0.360366	0.0243	0.0590614	no
Folr4	1.367	1.91261	0.484525	0.1637	0.286877	no
Fopnl	37.6695	34.9929	-0.106337	0.48645	0.641637	no
Fos	131.232	155.121	0.241271	0.0979	0.190043	no
Fosb	49.9084	65.7629	0.397991	0.006	0.0175533	yes
Fosl2	7.84619	8.62974	0.137325	0.36735	0.525822	no
Foxd2	1.90815	1.19302	-0.677554	0.01425	0.0371649	yes
Foxj2	13.2574	14.0082	0.0794704	0.5873	0.729379	no
Foxj3	16.0947	16.635	0.0476393	0.74545	0.847514	no
Foxk1	18.2662	19.2559	0.0761253	0.5845	0.727053	no
Foxk2	12.7115	12.2427	-0.0542079	0.71435	0.825939	no
Foxm1	17.944	10.455	-0.779302	5.00E-05	0.000236281	yes
Foxn2	19.5437	18.8934	-0.0488251	0.73135	0.837636	no
Foxn3	14.8105	13.0895	-0.178212	0.26035	0.406127	no
Foxo1	30.3022	38.1342	0.331661	0.02145	0.0530292	no
Foxo3	13.6224	13.1265	-0.0535006	0.7279	0.835107	no
Foxo4	20.3296	19.7311	-0.0431096	0.7675	0.862126	no
Foxp1	17.2709	17.6749	0.0333599	0.81305	0.891845	no
Foxp4	28.3413	36.2273	0.354172	0.0125	0.0332244	yes
Foxred1	16.2427	17.1112	0.0751503	0.6285	0.762422	no
Foxred2	0.919822	1.79693	0.966105	0.00015	0.000653255	yes
Fpgs	6.41602	7.98819	0.31619	0.08545	0.169712	no
Fpgt	4.87901	4.97766	0.0288776	0.86805	0.925747	no
Fra10ac1	12.0256	13.0216	0.114798	0.5262	0.677593	no
Frat1	5.31307	5.41992	0.0287254	0.87315	0.928702	no
Frat2	23.1006	23.976	0.0536579	0.72095	0.830536	no
Frg1	89.1591	86.1451	-0.0496128	0.73315	0.838935	no
Frmd4a	0.605655	1.03567	0.774004	0.0024	0.00784462	yes
Frmd4b	3.1403	2.51161	-0.322288	0.08025	0.160984	no
Frmd6	2.65552	2.29056	-0.213293	0.27415	0.42274	no
Frmd7	3.81524	4.04473	0.0842723	0.6324	0.765785	no
Frmd8	81.8877	96.9073	0.24296	0.0842	0.167735	no
Frrs1	13.3099	16.8571	0.340863	0.03125	0.0730529	no
Frs2	6.45347	6.50774	0.0120804	0.9378	0.966332	no
Fry	4.28864	4.31306	0.00819025	0.9563	0.97657	no
Fryl	54.7709	57.5225	0.0707172	0.6164	0.75309	no
Fth1	502.51	555.07	0.143515	0.31115	0.464756	no

Ftl1	528.229	520.367	-0.0216334	0.8782	0.931855	no
Fto	12.5684	13.1613	0.0665011	0.6671	0.792188	no
Ftsj1	4.48793	4.24523	-0.0802068	0.659	0.786299	no
Ftsj2	5.86244	6.03347	0.0414879	0.85275	0.916381	no
Ftsj3	29.1821	32.0327	0.134461	0.35145	0.508821	no
Fubp1	19.141	19.9925	0.0627897	0.6635	0.789533	no
Fubp3	5.27333	6.72909	0.351695	0.04515	0.0997989	no
Fuca1	19.0824	19.3809	0.0223956	0.8869	0.936623	no
Fuca2	13.1182	13.9934	0.0931688	0.5415	0.690792	no
Fuk	2.63554	2.94962	0.162432	0.4158	0.574527	no
Fundc1	14.5266	15.6323	0.105838	0.5227	0.674551	no
Fundc2	21.079	19.1545	-0.138128	0.346	0.503015	no
Furin	15.3861	18.3792	0.256445	0.08085	0.161998	no
Fus	137.042	128.022	-0.0982187	0.48715	0.642368	no
Fut11	9.56774	9.26864	-0.0458216	0.77055	0.864328	no
Fut7	5.58132	7.97216	0.514363	0.00905	0.0251275	yes
Fut8	33.9818	50.324	0.566484	5.00E-05	0.000236281	yes
Fuz	9.03085	10.1757	0.172188	0.68835	0.80784	no
Fv1	4.22394	3.84243	-0.136571	0.5885	0.730368	no
Fxn	12.297	12.1246	-0.0203662	0.91675	0.954324	no
Fxr1	47.0254	46.3669	-0.0203472	0.89385	0.940655	no
Fxr2	28.4222	28.8758	0.0228451	0.8795	0.932505	no
Fxyd5	773.875	754.721	-0.0361571	0.80085	0.884221	no
Fxyd7	1.20429	1.11568	-0.110258	0.8389	0.907619	no
Fyb	113.439	123.829	0.126428	0.3734	0.532033	no
Fyco1	18.2386	24.7304	0.439288	0.1006	0.194222	no
Fyn	298.79	230.475	-0.374525	0.0106	0.0288545	yes
Fyttd1	8.48631	8.68329	0.033105	0.83275	0.904041	no
Fzd5	2.95273	2.57169	-0.199337	0.24605	0.391177	no
Fzr1	31.8626	28.5387	-0.158947	0.28575	0.436111	no
G0s2	4.70258	2.65945	-0.822327	0.0143	0.0372788	yes
G2e3	4.6422	3.91225	-0.246812	0.1293	0.237966	no
G3bp1	89.3388	88.7759	-0.00911906	0.9499	0.973004	no
G3bp2	53.0048	53.2814	0.00750779	0.95535	0.976122	no
G6pc3	13.3191	16.8367	0.338111	0.04625	0.101841	no
G6pdx	26.6641	26.947	0.015226	0.942	0.968581	no
Gaa	6.98779	9.57214	0.454005	0.005	0.0149735	yes
Gab3	15.5545	14.8499	-0.0668744	0.67715	0.799615	no
Gabarap	215.821	196.822	-0.132945	0.34495	0.50183	no
Gabarapl1	18.891	15.986	-0.24089	0.13855	0.25085	no
Gabarapl2	258.836	244.016	-0.0850674	0.54315	0.692322	no
Gabbr1	26.4042	24.8452	-0.0877986	0.53455	0.684984	no
Gabpa	26.7231	24.7472	-0.110818	0.58145	0.724485	no
Gabpb1	18.0323	15.5758	-0.211276	0.19325	0.327176	no
Gabpb2	41.3637	41.0632	-0.0105177	0.96665	0.981911	no
Gadd45a	13.7063	11.8673	-0.207843	0.31595	0.469937	no
Gadd45b	49.2746	48.7822	-0.0144893	0.92315	0.958146	no
Gadd45g	28.5747	23.3845	-0.28918	0.08765	0.173408	no
Gadd45gip1	23.3377	25.2254	0.112214	0.6545	0.782849	no
Gak	44.442	44.0215	-0.0137161	0.9221	0.957746	no
Galc	1.06937	1.49863	0.486886	0.05635	0.120393	no
Gale	9.07528	9.01902	-0.00897152	0.9647	0.9808	no
Galk1	4.90811	5.40291	0.13857	0.5395	0.68915	no
Galk2	20.8555	19.7074	-0.0816878	0.58215	0.72505	no
Galm	2.81615	2.87798	0.031334	0.89205	0.939573	no
Galns	7.66773	6.96073	-0.139561	0.4331	0.591335	no
Galnt1	47.4451	51.4948	0.118167	0.39325	0.552344	no

Galnt10	29.6269	35.6836	0.268355	0.0576	0.1226	no
Galnt11	8.00045	9.20868	0.202914	0.2362	0.380697	no
Galnt12	18.4966	19.002	0.0388933	0.79985	0.883587	no
Galnt2	40.1089	40.524	0.0148566	0.91375	0.952775	no
Galnt3	12.0027	9.06777	-0.404538	0.01095	0.0296791	yes
Galnt4	4.71827	4.43806	-0.0883272	0.6722	0.795941	no
Galnt6	35.519	38.644	0.121654	0.38935	0.548503	no
Galnt7	19.7806	18.8355	-0.0706299	0.625	0.759783	no
Galr3	3.06267	4.29139	0.486657	0.1773	0.305574	no
Galt	14.6155	14.4674	-0.0146913	0.92725	0.960312	no
Gan	1.82553	1.74597	-0.0642857	0.79605	0.881265	no
Ganab	80.4365	80.8506	0.00740838	0.9556	0.976279	no
Ganc	7.99801	7.86624	-0.023967	0.92545	0.959306	no
Gapdh	562.568	534.472	-0.0739119	0.60755	0.745733	no
Gapvd1	21.1234	21.4881	0.0246991	0.86155	0.921781	no
Gar1	19.7451	21.1073	0.0962482	0.56605	0.711425	no
Gars	98.02	88.4387	-0.148398	0.28795	0.438577	no
Gart	17.2488	21.3628	0.308599	0.03765	0.0855023	no
Gas2l1	1.15716	0.387623	-1.57786	0.00045	0.0017621	yes
Gas7	3.10153	3.40027	0.132669	0.43355	0.591789	no
Gas8	3.10635	3.622	0.221565	0.37325	0.531884	no
Gata3	16.7055	21.9728	0.39539	0.009	0.0250025	yes
Gatad1	17.1277	17.5372	0.0340891	0.8209	0.896777	no
Gatad2a	37.5244	39.0056	0.0558521	0.6877	0.807305	no
Gatad2b	22.2376	23.1645	0.0589176	0.6992	0.815516	no
Gatc	9.09809	8.868	-0.0369551	0.8452	0.91164	no
Gatsl2	1.12607	1.16973	0.0548796	0.8194	0.895929	no
Gatsl3	10.8633	10.0011	-0.119301	0.51815	0.670579	no
Gba	49.0018	46.4471	-0.0772458	0.59455	0.735355	no
Gba2	24.5965	22.8128	-0.108608	0.6383	0.770352	no
Gbas	10.728	9.52397	-0.171742	0.323	0.478005	no
Gbe1	2.93844	3.86126	0.394023	0.05295	0.114106	no
Gbf1	17.3845	18.0434	0.0536701	0.7132	0.82519	no
Gbp2	44.0442	36.9009	-0.255296	0.0748	0.151807	no
Gbp3	29.6348	25.4667	-0.21868	0.1335	0.2442	no
Gbp4	42.9918	41.0245	-0.0675732	0.6349	0.767706	no
Gbp5	13.2848	12.8964	-0.0428027	0.78655	0.875057	no
Gbp6	6.53979	5.6898	-0.200866	0.2233	0.365378	no
Gbp7	38.7263	36.0579	-0.102998	0.4631	0.620226	no
Gbp8	41.0915	40.9663	-0.00439983	0.97655	0.98701	no
Gbp9	21.9029	23.0158	0.0715032	0.6239	0.758877	no
Gcat	4.46439	5.42765	0.281862	0.2341	0.378106	no
Gcc1	12.7142	12.2266	-0.0564165	0.7134	0.825368	no
Gcc2	8.98237	8.7433	-0.0389182	0.7966	0.881556	no
Gcdh	15.1859	16.0194	0.07709	0.65315	0.781937	no
Gcfc2	3.73103	3.44464	-0.115219	0.71895	0.829003	no
Gch1	3.18209	3.44122	0.112943	0.5813	0.724349	no
Gclc	20.4244	19.5586	-0.0624903	0.67285	0.79639	no
Gclm	14.2232	17.4544	0.29534	0.06785	0.140094	no
Gcn1l1	17.1955	19.3234	0.168323	0.23695	0.381574	no
Gcnt1	1.22342	1.85962	0.604083	0.00785	0.0221974	yes
Gcsh	8.74832	11.2816	0.366894	0.06065	0.127942	no
Gdap2	15.3287	13.8855	-0.14266	0.35585	0.513611	no
Gde1	29.121	31.7643	0.125345	0.4139	0.57272	no
Gdf11	13.007	14.2887	0.135586	0.4212	0.579628	no
Gdi1	88.5986	87.7773	-0.0134365	0.92105	0.957064	no
Gdi2	151.662	141.721	-0.0978121	0.5375	0.687541	no

Gdpd3	1.37084	33.1231	4.59471	5.00E-05	0.000236281	yes
Gdpd5	2.537	2.25673	-0.168886	0.4196	0.578114	no
Gdpgp1	2.72287	2.93782	0.109621	0.58325	0.72601	no
Gem	24.4998	19.2156	-0.350491	0.0222	0.0546175	no
Gemin2	16.3422	16.6486	0.0268027	0.88255	0.934265	no
Gemin4	3.93497	3.94433	0.00342731	0.98195	0.989605	no
Gemin5	6.94907	8.279	0.252638	0.09815	0.190429	no
Gemin6	3.97278	3.52126	-0.17406	0.5316	0.682344	no
Gemin7	55.7734	52.9267	-0.0755808	0.6434	0.774217	no
Gemin8	2.8159	3.40339	0.273375	0.26445	0.411214	no
Gen1	3.18722	2.15775	-0.562766	0.009	0.0250025	yes
Get4	27.2506	23.9486	-0.186341	0.21115	0.350577	no
Gfer	6.90129	6.2816	-0.135733	0.48825	0.643302	no
Gfi1	16.4058	13.9932	-0.229479	0.13415	0.245135	no
Gfm1	19.7743	20.1617	0.0279957	0.8731	0.928702	no
Gfm2	13.1283	13.6547	0.0567156	0.8294	0.902042	no
Gfod1	8.84889	8.53604	-0.0519296	0.72585	0.833799	no
Gfod2	1.10267	2.34209	1.08679	5.00E-04	0.00193907	yes
Gfpt1	19.365	17.4741	-0.148227	0.30185	0.454414	no
Gga1	44.1574	44.7811	0.0202319	0.8866	0.936446	no
Gga2	1.61068	1.68719	0.0669516	0.75385	0.852929	no
Gga3	13.8191	12.6779	-0.124358	0.41025	0.56921	no
Ggact	13.9954	17.0368	0.283697	0.1299	0.23889	no
Ggct	21.3555	20.7208	-0.0435296	0.7959	0.88114	no
Ggcx	5.43196	5.57111	0.0364919	0.83585	0.905916	no
Ggh	44.3602	36.4921	-0.281682	0.0663	0.137425	no
Ggnbp2	70.2976	72.435	0.0432101	0.75945	0.856929	no
Ggps1	12.7845	12.7058	-0.00890142	0.95515	0.976045	no
Ggt1	20.7994	48.3249	1.21622	5.00E-05	0.000236281	yes
Ggta1	13.4214	13.0611	-0.0392564	0.79915	0.883228	no
Ghdc	5.46503	4.35638	-0.3271	0.10085	0.194608	no
Ghitm	70.1235	67.6884	-0.0509891	0.72015	0.829966	no
Gid4	5.69611	7.02124	0.30175	0.06705	0.138698	no
Gid8	20.5844	20.2208	-0.0257094	0.86085	0.921424	no
Gigyf1	17.7777	20.8043	0.22681	0.1126	0.212882	no
Gigyf2	16.6256	16.9892	0.0312102	0.8273	0.900841	no
Gimap1	221.767	191.976	-0.208123	0.14075	0.254129	no
Gimap3	463.292	426.308	-0.120026	0.4146	0.573374	no
Gimap4	963.467	841.265	-0.195674	0.2232	0.365266	no
Gimap5	74.2688	68.2578	-0.121762	0.3926	0.551749	no
Gimap6	488.226	464.851	-0.0707808	0.61505	0.752026	no
Gimap7	203.221	152.653	-0.412792	0.0029	0.00927196	yes
Gimap8	151.499	141.481	-0.0986963	0.4929	0.647651	no
Gimap9	104.503	91.2355	-0.19587	0.16485	0.288492	no
Gin1	6.63455	5.94288	-0.158836	0.4021	0.561123	no
Ginm1	16.7927	18.1921	0.115475	0.49015	0.645155	no
Gins1	5.9842	4.30147	-0.476329	0.114	0.215097	no
Gins2	9.65064	5.71261	-0.756474	0.22655	0.369386	no
Gins3	6.1451	4.84387	-0.343276	0.0714	0.146205	no
Gins4	28.8057	28.2335	-0.0289461	0.85595	0.918337	no
Gipc1	16.7	16.7978	0.00841698	0.96275	0.980023	no
Git1	19.4177	19.1644	-0.0189423	0.89915	0.944177	no
Git2	35.8615	37.1546	0.0511053	0.7155	0.826713	no
Gkap1	1.74785	1.39099	-0.329472	0.3213	0.476208	no
Gla	3.52887	3.30029	-0.0966136	0.6334	0.766483	no
Glb1	15.9166	14.3969	-0.14478	0.48065	0.636353	no
Glb1l	4.24631	4.33755	0.0306675	0.91905	0.955816	no

Glcci1	9.32368	7.88399	-0.241973	0.10955	0.208327	no
Glce	1.18818	1.21226	0.0289466	0.9023	0.946077	no
Gle1	22.397	21.2187	-0.0779675	0.59555	0.736102	no
Glg1	34.082	37.6615	0.144082	0.31135	0.464976	no
Glipr1	141.179	145.284	0.0413528	0.80815	0.888874	no
Glipr2	216.142	235.625	0.124515	0.38275	0.541855	no
Glmn	9.02115	7.70826	-0.226905	0.42245	0.580839	no
Glo1	40.6498	43.3785	0.0937296	0.57395	0.718043	no
Glod4	24.5184	22.6084	-0.117004	0.44095	0.598867	no
Glrx	76.9162	71.5301	-0.104737	0.47715	0.633216	no
Glrx2	7.57573	7.28839	-0.0557837	0.7312	0.837573	no
Glrx3	23.6029	23.1897	-0.0254791	0.91715	0.954586	no
Glrx5	52.4752	55.6426	0.084554	0.58675	0.728863	no
Gls	42.8826	42.2393	-0.021806	0.8809	0.933247	no
Glt25d1	22.2689	24.0808	0.112854	0.4348	0.592934	no
Glt8d1	6.47311	5.60848	-0.206848	0.65665	0.784357	no
Gltp	99.285	86.9161	-0.191952	0.17655	0.30452	no
Gltpd1	4.12807	3.65303	-0.176374	0.373	0.531667	no
Gltscr1	17.2384	20.3061	0.236287	0.09905	0.191827	no
Gltscr1l	21.6325	20.8409	-0.0537833	0.70685	0.820871	no
Gltscr2	406.825	389.412	-0.0631092	0.6517	0.780744	no
Glud1	117.319	123.368	0.0725314	0.6147	0.751702	no
Glul	40.9883	36.7979	-0.15559	0.275	0.423782	no
Glyctk	4.66602	4.6911	0.0077317	0.96355	0.980356	no
Glyr1	59.5757	57.5524	-0.0498494	0.72855	0.835527	no
Gm10094	156.661	150.159	-0.0611484	0.70855	0.82202	no
Gm10767	1.45081	0.879846	-0.721537	0.05075	0.110149	no
Gm11127	1.62046	1.57145	-0.0442998	0.90525	0.947805	no
Gm12250	5.80907	6.53481	0.169838	0.3432	0.499974	no
Gm12657	1.06568	1.20561	0.177985	0.71	0.823039	no
Gm12942	6.36371	6.39337	0.00670752	0.98005	0.988636	no
Gm13051	1.13993	1.55649	0.449359	0.10285	0.197803	no
Gm13139	4.53322	4.09705	-0.145951	0.5451	0.693742	no
Gm13157	19.067	18.8889	-0.0135432	0.92825	0.960812	no
Gm13212	6.45379	7.52172	0.220917	0.2633	0.409805	no
Gm13251	1.94095	2.17295	0.16289	0.45465	0.612395	no
Gm13826	2.46614	1.6517	-0.5783	0.2008	0.337104	no
Gm14295	20.8678	19.063	-0.130502	0.44495	0.602665	no
Gm14325	2.16493	2.53983	0.230412	0.2499	0.395392	no
Gm14326	4.54469	4.38916	-0.0502381	0.85895	0.920102	no
Gm14378	2.34115	2.43551	0.0570035	0.86435	0.923447	no
Gm14420	2.17078	2.22123	0.0331475	0.8652	0.92402	no
Gm14446	26.4761	14.3523	-0.883405	5.00E-05	0.000236281	yes
Gm15800	11.166	12.1948	0.127149	0.3702	0.528786	no
Gm16515	8.26148	7.86071	-0.0717413	0.6506	0.77981	no
Gm166	8.60455	8.10386	-0.08649	0.72645	0.834312	no
Gm17296	2.23351	2.28901	0.0354115	0.8523	0.916023	no
Gm1966	36.9224	39.0478	0.0807455	0.57285	0.717099	no
Gm20604	3.23845	2.89378	-0.162351	0.64935	0.778814	no
Gm2382	26.3533	22.3453	-0.238011	0.1991	0.334983	no
Gm2a	41.4425	48.0325	0.212897	0.1296	0.238413	no
Gm4070	1.69577	1.65069	-0.0388763	0.85855	0.92005	no
Gm4944	2.90157	3.59348	0.308549	0.08875	0.175197	no
Gm5141	1.38524	1.49836	0.113243	0.657	0.784616	no
Gm5148	1.73765	1.96543	0.177712	0.60505	0.743793	no
Gm527	1.18087	0.793436	-0.573667	0.15795	0.278704	no
Gm561	23.5463	24.9856	0.085594	0.71185	0.824263	no

Gm5617	7.99467	7.49539	-0.0930358	0.73635	0.841301	no
Gm5741	0.707345	1.15668	0.709502	0.57905	0.722438	no
Gm5901	1.96743	3.37268	0.777579	0.56685	0.712038	no
Gm608	6.92006	6.50122	-0.0900744	0.53635	0.686568	no
Gm614	14.6745	13.4581	-0.124833	0.5605	0.706804	no
Gm6904	4.94358	3.09139	-0.677301	0.0254	0.0613714	no
Gm7102	3.42232	2.68718	-0.348882	0.17285	0.299433	no
Gm7120	3.17384	3.40843	0.102876	0.7479	0.849109	no
Gm7609	2.04722	1.10271	-0.89261	0.00985	0.0270919	yes
Gm8369	20.9159	22.7359	0.120372	0.4698	0.62643	no
Gm8817	8.4162	6.95318	-0.275495	0.3517	0.509047	no
Gmcl1	16.8769	17.2639	0.0327103	0.8301	0.902483	no
Gmds	13.1725	11.9911	-0.135574	0.4436	0.601367	no
Gmeb1	7.31657	6.67982	-0.131359	0.3911	0.550167	no
Gmeb2	14.959	14.1741	-0.0777605	0.60325	0.742382	no
Gmfb	29.5499	31.0651	0.0721412	0.61535	0.752315	no
Gmfg	435.995	420.713	-0.0514732	0.71935	0.829342	no
Gmip	51.7273	53.8469	0.0579366	0.6823	0.803398	no
Gmnn	37.9489	24.0724	-0.65668	3.00E-04	0.00122185	yes
Gmppa	30.0164	30.8498	0.0395104	0.7926	0.878976	no
Gmppb	10.3238	10.5819	0.0356322	0.94015	0.967637	no
Gmpr2	25.893	22.5211	-0.201288	0.2063	0.344193	no
Gmps	17.0684	15.9102	-0.101374	0.48085	0.636546	no
Gna11	11.6768	12.1222	0.0539958	0.7255	0.8336	no
Gna12	7.69118	9.26168	0.268069	0.14155	0.255188	no
Gna13	43.2995	44.8477	0.0506824	0.7136	0.825504	no
Gna15	51.2025	62.5558	0.288928	0.0432	0.0961207	no
Gnai2	463.466	434.311	-0.0937364	0.522	0.673993	no
Gnai3	98.3006	90.9456	-0.112197	0.43185	0.590074	no
Gnal	5.58166	6.28552	0.171338	0.6534	0.78213	no
Gnaq	1.33641	1.39338	0.0602267	0.78405	0.873405	no
Gnas	463.015	422.143	-0.133328	0.3603	0.518359	no
Gnb1	100.606	95.0686	-0.0816736	0.5647	0.710349	no
Gnb1l	2.48187	2.58422	0.0583049	0.7779	0.869229	no
Gnb2	273.851	274.509	0.00346183	0.98075	0.988975	no
Gnb2l1	1713.55	1690.34	-0.0196722	0.898	0.943447	no
Gnb4	2.40593	2.37812	-0.0167742	0.9437	0.969552	no
Gne	12.2148	13.6726	0.162654	0.3122	0.465851	no
Gng10	79.6834	69.1303	-0.204962	0.162	0.284442	no
Gng12	1.1459	0.979632	-0.226168	0.3709	0.529486	no
Gng2	64.3802	65.7282	0.0298964	0.8326	0.903961	no
Gng3	1.15657	1.03865	-0.155141	0.8996	0.944435	no
Gng5	241.519	203.552	-0.246739	0.09465	0.184725	no
Gngt2	13.6725	10.5981	-0.36747	0.42695	0.585361	no
Gnl1	16.9231	17.5207	0.0500673	0.74805	0.849252	no
Gnl2	23.9643	26.7694	0.159695	0.2843	0.434499	no
Gnl3	13.1826	13.9177	0.0782941	0.80005	0.883697	no
Gnl3l	11.1377	11.8835	0.0935037	0.5417	0.690935	no
Gnpat	35.3036	36.847	0.0617324	0.66835	0.793098	no
Gnpda1	34.1946	26.2668	-0.38053	0.01105	0.0299157	yes
Gnpda2	21.565	19.8358	-0.120585	0.44745	0.604855	no
Gnpnat1	9.65507	10.9136	0.176772	0.50035	0.65454	no
Gnptab	50.2273	43.0753	-0.221611	0.1176	0.220627	no
Gnptg	76.9156	75.2972	-0.03068	0.8884	0.937435	no
Gnrh1	3.96145	3.37134	-0.232704	0.56765	0.712662	no
Gns	16.2808	15.047	-0.113697	0.4471	0.604612	no
Golga1	5.96669	5.18341	-0.203029	0.2189	0.359931	no

Golga2	23.2995	22.8664	-0.0270644	0.84885	0.913762	no
Golga3	16.1687	16.1741	0.000476155	0.9985	0.998741	no
Golga4	18.1024	18.9699	0.0675341	0.633	0.766183	no
Golga5	27.4359	26.7831	-0.0347373	0.81135	0.890853	no
Golga7	62.0222	69.9564	0.173671	0.22295	0.364907	no
Golgb1	12.1976	13.4438	0.140337	0.3277	0.483238	no
Golim4	18.8518	14.1402	-0.414903	0.00455	0.0137968	yes
Golm1	71.719	66.4068	-0.111024	0.4319	0.590085	no
Golph3	55.0469	52.162	-0.0776644	0.58465	0.72715	no
Golph3l	11.8863	9.16702	-0.374781	0.02255	0.055332	no
Golt1b	12.6436	10.5355	-0.263148	0.1022	0.196772	no
Gon4l	13.0089	12.7151	-0.0329614	0.836	0.906023	no
Gopc	16.5981	16.7331	0.0116806	0.9383	0.966678	no
Gorab	3.99123	3.95274	-0.0139801	0.9447	0.970043	no
Gorasp1	4.56053	3.99939	-0.18942	0.5914	0.732653	no
Gorasp2	46.0793	44.7768	-0.041369	0.7686	0.862963	no
Gosr1	29.1809	26.3074	-0.149555	0.29515	0.446671	no
Gosr2	61.077	58.1057	-0.0719501	0.6049	0.743661	no
Got1	18.5124	16.7741	-0.14225	0.3653	0.523642	no
Got2	60.4687	55.9197	-0.112832	0.42315	0.581541	no
Gp49a	16.0416	18.6081	0.214112	0.21195	0.35155	no
Gpaa1	21.7503	20.4927	-0.0859282	0.57505	0.718986	no
Gpalpp1	13.5943	12.7195	-0.0959556	0.5096	0.662979	no
Gpam	3.31765	3.16196	-0.069344	0.71345	0.825398	no
Gpank1	28.16	27.3062	-0.0444146	0.7805	0.870921	no
Gpatch1	7.29447	7.21784	-0.015236	0.9289	0.96116	no
Gpatch11	9.00274	9.06801	0.0104221	0.9458	0.970594	no
Gpatch2	6.29888	6.88572	0.128511	0.434	0.592277	no
Gpatch2l	18.5435	17.3263	-0.0979467	0.50405	0.658124	no
Gpatch3	5.71278	6.23356	0.125864	0.5188	0.671125	no
Gpatch4	8.70539	10.4865	0.268548	0.14575	0.261014	no
Gpatch8	11.3071	11.6016	0.0370947	0.795	0.880586	no
Gpbp1	56.3624	56.5798	0.00555348	0.96635	0.981677	no
Gpbp1l1	45.4001	48.442	0.0935639	0.51525	0.668051	no
Gpc1	6.94143	6.91979	-0.00450574	0.97825	0.987837	no
Gpcpd1	40.0853	39.5732	-0.0185506	0.895	0.941402	no
Gpd1l	25.516	26.5549	0.0575744	0.6887	0.808063	no
Gpd2	10.3355	6.67523	-0.630715	5.00E-05	0.000236281	yes
Gphn	5.89258	5.8296	-0.0155018	0.9294	0.961424	no
Gpi1	211.184	218.133	0.0467051	0.75155	0.851514	no
Gpkow	19.1588	17.5358	-0.127706	0.38615	0.545148	no
Gpld1	6.76774	6.90812	0.0296179	0.85525	0.917949	no
Gpn1	13.7813	13.9803	0.0206859	0.8986	0.943838	no
Gpn2	15.5577	16.5373	0.0880913	0.603	0.742191	no
Gpn3	26.3771	29.2851	0.150884	0.342	0.498607	no
Gpr107	15.0312	15.3403	0.0293656	0.8458	0.911855	no
Gpr108	38.641	36.7189	-0.073612	0.61055	0.748131	no
Gpr114	39.0262	25.9358	-0.589501	5.00E-05	0.000236281	yes
Gpr132	72.4046	77.2659	0.0937502	0.5072	0.660867	no
Gpr137	8.51271	8.19925	-0.0541264	0.8471	0.912671	no
Gpr146	6.74996	8.41064	0.317337	0.0489	0.106734	no
Gpr15	1.95929	3.09165	0.658051	0.0308	0.072142	no
Gpr155	2.01507	1.77276	-0.184833	0.35965	0.517624	no
Gpr160	11.312	12.3717	0.129181	0.44855	0.605948	no
Gpr171	44.5721	38.3621	-0.216458	0.2367	0.381311	no
Gpr174	22.5792	20.636	-0.129831	0.35975	0.517737	no
Gpr18	42.1592	42.1619	9.27E-05	0.99885	0.99902	no

Gpr180	8.23437	9.2376	0.165861	0.3164	0.470477	no
Gpr183	17.9814	30.6775	0.770676	0.0047	0.0141816	yes
Gpr19	2.20609	1.95443	-0.174742	0.5246	0.676249	no
Gpr55	8.88449	6.61145	-0.426321	0.01645	0.0420542	yes
Gpr65	20.5142	19.8971	-0.044066	0.77535	0.867547	no
Gpr68	38.927	43.0348	0.144731	0.31915	0.473658	no
Gpr89	13.6268	13.4944	-0.0140839	0.9358	0.965116	no
Gprasp1	3.29528	3.63163	0.140216	0.5316	0.682344	no
Gprin3	9.58931	11.2027	0.224342	0.1652	0.288997	no
Gps1	38.8569	34.7223	-0.162307	0.2756	0.424465	no
Gps2	93.0958	87.1622	-0.0950142	0.4987	0.652962	no
Gpsm1	5.85206	5.60515	-0.0621936	0.7975	0.882095	no
Gpsm2	9.03618	6.24881	-0.532132	0.03175	0.0740403	no
Gpsm3	171.738	161.663	-0.0872167	0.53175	0.682412	no
Gpt	3.31937	2.90559	-0.192076	0.44165	0.599518	no
Gpx1	86.4662	92.8653	0.103003	0.48115	0.636847	no
Gpx4	105.611	111.053	0.072494	0.6186	0.754811	no
Gpx8	9.18531	2.03432	-2.17478	5.00E-05	0.000236281	yes
Gramd1a	210.181	226.202	0.10598	0.46105	0.618172	no
Gramd1b	34.7903	36.5273	0.0702889	0.62595	0.760532	no
Gramd1c	1.68738	1.9668	0.221061	0.3363	0.492433	no
Gramd3	147.743	147.623	-0.00116356	0.9932	0.995754	no
Gramd4	45.9151	51.8424	0.175164	0.21465	0.354869	no
Grap	187.455	198.205	0.0804492	0.56425	0.70996	no
Grap2	119.541	109.873	-0.121669	0.40265	0.561635	no
Grasp	5.12996	4.91775	-0.0609483	0.77155	0.865023	no
Grb2	104.672	104.883	0.00289605	0.98405	0.990703	no
Grb7	0.953991	1.6372	0.779182	0.01185	0.031749	yes
Grcc10	219.213	226.422	0.0466786	0.7545	0.853431	no
Grhpr	9.2927	10.2748	0.144935	0.4647	0.621755	no
Grifin	0.460441	1.16973	1.34508	0.13405	0.244984	no
Grik5	1.27899	1.98857	0.636721	0.00835	0.0234077	yes
Grina	56.2307	52.4722	-0.099806	0.49075	0.645619	no
Gripap1	34.756	34.6212	-0.00560425	0.96745	0.982271	no
Grk4	1.45104	1.53605	0.0821341	0.7557	0.854227	no
Grk5	1.78864	1.19278	-0.584538	0.02505	0.06064	no
Grk6	139.288	149.64	0.103424	0.4733	0.629752	no
Grn	6.75067	6.0285	-0.163232	0.3739	0.532595	no
Grpel1	17.4559	17.797	0.0279219	0.87665	0.930896	no
Grpel2	6.7068	7.25005	0.112366	0.4881	0.6432	no
Grsf1	24.6133	23.5148	-0.0658661	0.65995	0.78702	no
Grwd1	7.21144	8.75727	0.280193	0.13345	0.244127	no
Gsap	34.0703	22.2718	-0.613294	5.00E-05	0.000236281	yes
Gsdmd	38.1795	35.6698	-0.0980976	0.51385	0.666738	no
Gse1	14.0059	15.7859	0.172595	0.2406	0.385698	no
Gsg2	10.9967	7.90146	-0.476885	0.0086	0.0240265	yes
Gsk3a	82.5386	81.3495	-0.0209354	0.8907	0.938768	no
Gsk3b	17.3732	16.5619	-0.0689958	0.6234	0.758543	no
Gskip	13.1073	12.1516	-0.109232	0.48635	0.641553	no
Gsn	21.3241	25.5166	0.258951	0.0776	0.156638	no
Gspt1	18.0801	17.5111	-0.0461275	0.75025	0.850751	no
Gsr	22.793	23.4954	0.04379	0.7642	0.860034	no
Gss	10.8576	7.7749	-0.481815	0.0079	0.0223218	yes
Gstcd	5.6594	5.72082	0.0155726	0.93085	0.962205	no
Gstk1	3.62905	3.48422	-0.0587524	0.8483	0.91342	no
Gstm1	1.03478	0.764256	-0.4372	0.3239	0.479016	no
Gstm4	1.50689	1.87843	0.317951	0.32525	0.480459	no

Gsto1	11.2153	18.1618	0.695437	1.00E-04	0.000450026	yes
Gstp1	73.5334	80.8447	0.136753	0.3755	0.534227	no
Gstt1	1.01188	1.03409	0.0313285	0.95785	0.97729	no
Gstt2	23.5909	35.0982	0.573168	0.00035	0.0014053	yes
Gstt3	0.911744	1.81065	0.98981	0.00495	0.0148433	yes
Gstz1	5.14521	5.48425	0.0920644	0.7193	0.829325	no
Gtdc1	7.90949	6.95341	-0.185864	0.2918	0.442873	no
Gtf2a1	8.56799	8.01739	-0.0958235	0.5241	0.675814	no
Gtf2a2	40.8154	35.898	-0.185208	0.25955	0.405219	no
Gtf2b	55.0144	50.1982	-0.132174	0.3776	0.536533	no
Gtf2e1	9.78971	9.93506	0.0212628	0.89435	0.941055	no
Gtf2e2	23.1185	21.2649	-0.120569	0.45065	0.608075	no
Gtf2f1	48.3568	48.8225	0.0138264	0.92515	0.959108	no
Gtf2f2	25.3506	24.794	-0.0320309	0.8387	0.9075	no
Gtf2h1	28.8064	28.5488	-0.0129605	0.92795	0.960656	no
Gtf2h2	16.3859	16.0488	-0.0299911	0.85945	0.920358	no
Gtf2h3	13.7336	14.1581	0.0439111	0.77985	0.870554	no
Gtf2h4	8.65311	8.51443	-0.0233079	0.9035	0.946731	no
Gtf2h5	38.5638	36.4503	-0.0813139	0.59815	0.738281	no
Gtf2i	97.7239	101.962	0.0612417	0.6665	0.791781	no
Gtf2ird2	3.61893	4.8219	0.414037	0.0279	0.0663781	no
Gtf3a	47.7238	47.2037	-0.015809	0.93535	0.964864	no
Gtf3c1	15.6305	14.5129	-0.10702	0.45415	0.611838	no
Gtf3c2	29.9635	29.0729	-0.0435314	0.9161	0.953971	no
Gtf3c3	7.86212	8.25046	0.0695569	0.68165	0.802887	no
Gtf3c4	4.59757	4.7743	0.0544175	0.72845	0.83544	no
Gtf3c5	12.7166	11.9258	-0.0926285	0.5533	0.70088	no
Gtf3c6	29.4461	29.5028	0.00277153	0.98825	0.993076	no
Gtl3	16.8042	18.5908	0.14577	0.39595	0.554976	no
Gtpbp1	59.8403	63.7175	0.0905722	0.52565	0.677169	no
Gtpbp10	5.32309	6.36302	0.257447	0.1507	0.268265	no
Gtpbp2	62.1858	60.2988	-0.0444556	0.75895	0.856543	no
Gtpbp3	11.141	10.2865	-0.115127	0.4796	0.63538	no
Gtpbp4	30.079	30.3261	0.011802	0.933	0.963439	no
Gtpbp6	19.5609	19.399	-0.0119964	0.94095	0.968023	no
Gtpbp8	5.52893	5.46237	-0.0174742	0.93455	0.964438	no
Gtse1	8.50776	5.21092	-0.707243	5.00E-05	0.000236281	yes
Gucd1	26.4364	24.3052	-0.121264	0.40455	0.563472	no
Guf1	7.83448	8.34023	0.0902487	0.5771	0.720757	no
Guk1	40.9959	42.79	0.061793	0.7017	0.816974	no
Gusb	25.2762	27.2696	0.109514	0.46155	0.618643	no
Gxylt1	7.90231	7.05971	-0.162666	0.29035	0.44127	no
Gyg	28.6716	29.3202	0.0322742	0.8317	0.90343	no
Gyk	2.40329	2.60301	0.115169	0.55705	0.703975	no
Gypc	12.3787	11.6121	-0.0922341	0.5783	0.721745	no
Gys1	2.37797	3.54278	0.575152	0.00435	0.0132558	yes
Gzf1	5.02381	5.02593	0.000606838	0.9951	0.996682	no
Gzma	3011.63	1235.31	-1.28568	5.00E-05	0.000236281	yes
Gzmb	813.974	505.418	-0.687505	5.00E-05	0.000236281	yes
Gzmc	1.37318	1.20928	-0.183368	0.6828	0.803692	no
Gzmk	258.508	227.857	-0.18208	0.20755	0.34582	no
Gzmm	22.6094	16.3951	-0.463653	0.00645	0.0186894	yes
H13	47.5994	52.4726	0.140621	0.4605	0.617728	no
H1f0	20.8392	21.4137	0.039231	0.79615	0.881293	no
H2-Aa	4.12251	4.97226	0.270379	0.3044	0.457267	no
H2-Ab1	3.81233	4.26418	0.161594	0.54	0.689552	no
H2afj	45.5699	46.7438	0.0366946	0.7961	0.881279	no

H2afv	56.9791	47.174	-0.27244	0.0632	0.132195	no
H2afx	88.9015	60.8707	-0.546461	0.00015	0.000653255	yes
H2afy	267.089	270.173	0.0165612	0.90885	0.949752	no
H2afz	622.87	447.657	-0.476539	0.00065	0.00244919	yes
H2-D1	2067.68	1836.48	-0.171064	0.36575	0.524127	no
H2-DMa	8.72346	10.2316	0.230059	0.2177	0.358559	no
H2-Eb1	2.4233	3.16723	0.386251	0.1386	0.250908	no
H2-K1	2270.19	2104.91	-0.109057	0.56015	0.706615	no
H2-Ke2	82.6768	77.3089	-0.0968487	0.5451	0.693742	no
H2-Ke6	71.4269	78.2143	0.130965	0.37655	0.535376	no
H2-L	175.192	186.133	0.0873979	0.9234	0.958246	no
H2-M3	50.7931	42.9607	-0.241614	0.103	0.198021	no
H2-Oa	5.03713	7.78761	0.628579	0.00965	0.026603	yes
H2-Ob	2.16948	4.57112	1.0752	5.00E-05	0.000236281	yes
H2-Q10	61.9043	50.4613	-0.294862	0.04505	0.0996184	no
H2-Q4	375.025	295.553	-0.34357	0.01875	0.0471368	yes
H2-Q7	626.019	511.812	-0.290593	0.15265	0.271094	no
H2-Q8	309.127	293.164	-0.0764877	0.58635	0.728507	no
H2-Q9	382.917	293.834	-0.382031	0.08075	0.161826	no
H2-T10	20.3177	18.2998	-0.150912	0.3188	0.473228	no
H2-T22	45.6982	43.8778	-0.0586453	0.80415	0.886505	no
H2-T23	327.518	292.432	-0.163473	0.266	0.413033	no
H2-T24	15.8476	12.3403	-0.360886	0.47485	0.63122	no
H2-T9	83.8773	71.9575	-0.221134	0.235	0.379256	no
H3f3a	534.844	499.033	-0.0999826	0.48345	0.638957	no
H3f3b	497.29	497.435	0.000421094	0.9982	0.998525	no
H6pd	7.57251	7.0712	-0.0988165	0.53325	0.683678	no
Haao	34.1811	34.4326	0.0105775	0.94565	0.970482	no
Habp4	9.89626	11.517	0.218811	0.18485	0.315925	no
Hace1	11.0536	8.29093	-0.414904	0.0084	0.0235339	yes
Hacl1	2.94407	2.73748	-0.104963	0.6331	0.766251	no
Hadh	24.7306	28.4976	0.204543	0.18125	0.311053	no
Hadha	51.8822	48.1703	-0.107095	0.44475	0.602498	no
Hadhb	59.7455	53.5414	-0.158175	0.2703	0.418201	no
Hagh	14.7057	16.1674	0.136707	0.4508	0.608265	no
Haghl	7.60255	7.20387	-0.0777097	0.81485	0.892849	no
Harbi1	3.80538	3.74982	-0.0212178	0.918	0.955147	no
Hars	47.3266	43.9172	-0.107863	0.45545	0.613086	no
Hars2	14.6525	14.5446	-0.0106641	0.94605	0.97078	no
Hat1	36.8948	31.2403	-0.240008	0.11145	0.2112	no
Haus1	10.4231	10.5396	0.0160269	0.9337	0.963951	no
Haus2	9.91203	9.19899	-0.107704	0.61155	0.748901	no
Haus3	32.1437	27.7117	-0.214042	0.1522	0.270424	no
Haus4	39.5902	36.1357	-0.131719	0.3795	0.538496	no
Haus5	9.37331	8.8685	-0.0798686	0.6428	0.773706	no
Haus6	8.84936	7.53106	-0.232721	0.14885	0.265561	no
Haus7	12.2006	9.5187	-0.358115	0.0614	0.129284	no
Haus8	33.482	29.973	-0.159722	0.2907	0.441679	no
Havcr2	19.6154	13.7604	-0.511469	0.00095	0.00343861	yes
Hax1	32.773	33.2373	0.0202951	0.8975	0.943174	no
Hba-a1	2.90149	2.89474	-0.00335617	0.96275	0.980023	no
Hbb-b1	4.67053	5.22542	0.161961	0.62025	0.756053	no
Hbb-bt	1.13998	1.66006	0.54222	0.32675	0.482109	no
Hbp1	26.5264	26.4852	-0.0022419	0.99175	0.994922	no
Hbs1l	34.5431	31.8514	-0.117041	0.4299	0.588183	no
Hccs	16.8347	16.0489	-0.0689622	0.659	0.786299	no
Hcfc1	39.0937	37.4584	-0.0616475	0.6614	0.788097	no

Hcfc1r1	29.5426	28.6275	-0.0453976	0.82135	0.897088	no
Hcfc2	6.95113	6.09029	-0.190736	0.29925	0.451414	no
Hcls1	277.906	242.389	-0.197275	0.1646	0.288154	no
Hcst	668.096	628.855	-0.0873268	0.5367	0.686866	no
Hdac1	121.886	106.615	-0.193131	0.1734	0.300165	no
Hdac10	16.5073	19.7581	0.259341	0.08925	0.176006	no
Hdac2	33.351	30.2611	-0.140265	0.34665	0.503711	no
Hdac3	40.7649	38.8391	-0.0698178	0.6386	0.770569	no
Hdac4	5.50897	6.31024	0.195912	0.2431	0.387938	no
Hdac5	23.2195	22.1371	-0.0688702	0.6365	0.768811	no
Hdac6	4.22687	3.24021	-0.383506	0.039	0.0881202	no
Hdac7	114.001	144.764	0.344659	0.02245	0.0551326	no
Hdac8	9.3729	9.95531	0.0869701	0.62975	0.763466	no
Hddc2	24.6269	25.2352	0.0352003	0.84975	0.914255	no
Hddc3	1.92125	3.07494	0.678517	0.0701	0.143986	no
Hdgf	86.7393	77.4614	-0.163208	0.2526	0.396897	no
Hdgfrp2	42.5543	40.1029	-0.0855997	0.5474	0.695716	no
Hdgfrp3	18.9018	15.5549	-0.281158	0.14925	0.266167	no
Hdhd2	9.03529	8.51439	-0.0856674	0.6226	0.757883	no
Hdlbp	29.9251	27.66	-0.113554	0.4213	0.579687	no
Heatr1	8.85444	10.2191	0.20679	0.16355	0.286693	no
Heatr2	19.3934	18.8967	-0.0374353	0.8034	0.8859	no
Heatr3	9.02598	11.4651	0.345089	0.0297	0.0700139	no
Heatr5a	2.68603	3.2593	0.279087	0.09555	0.186188	no
Heatr5b	11.1498	10.1307	-0.138279	0.34235	0.499024	no
Heatr6	12.2752	11.8815	-0.0470292	0.7489	0.849835	no
Heca	48.3481	46.6984	-0.0500835	0.7184	0.82864	no
Hectd1	42.8955	47.5146	0.147544	0.30825	0.461469	no
Hectd3	34.9902	33.4689	-0.064129	0.65015	0.779442	no
Heg1	6.2631	8.17366	0.384106	0.01705	0.0433541	yes
Helb	13.4473	12.3647	-0.121084	0.4097	0.56867	no
Hells	12.7968	10.4009	-0.299076	0.0587	0.124558	no
Helq	6.34968	6.93923	0.128092	0.443	0.600796	no
Helz	15.8018	16.28	0.0430128	0.7659	0.861124	no
Helz2	24.5651	24.9601	0.0230147	0.87	0.926916	no
Hemk1	3.43567	3.37948	-0.0237901	0.9125	0.952077	no
Herc1	21.7103	19.6226	-0.145858	0.3135	0.467238	no
Herc2	24.2579	26.6059	0.133288	0.3451	0.501996	no
Herc3	20.6077	20.7878	0.0125482	0.9307	0.962092	no
Herc4	30.6595	29.7568	-0.0431145	0.7612	0.85793	no
Herc6	10.0495	9.20485	-0.126659	0.4044	0.563352	no
Herpud1	33.5173	33.4844	-0.00141554	0.9927	0.99545	no
Herpud2	30.8422	29.393	-0.0694328	0.63355	0.766572	no
Hes6	43.5348	39.5033	-0.140196	0.3532	0.510799	no
Hexa	26.6554	27.1807	0.0281537	0.8562	0.918508	no
Hexb	65.0074	53.2193	-0.288654	0.0863	0.171126	no
Hexim1	35.1355	37.3807	0.0893644	0.52785	0.679123	no
Hexim2	1.16574	0.796446	-0.5496	0.1182	0.221518	no
Hgs	22.7951	21.2188	-0.103382	0.48085	0.636546	no
Hgsnat	23.9156	22.9705	-0.0581682	0.68995	0.808991	no
Hiat1	29.8433	27.8125	-0.101672	0.48415	0.639607	no
Hiatl1	34.8797	32.9938	-0.0801925	0.569	0.713824	no
Hibadh	27.3014	29.2855	0.101208	0.505	0.658998	no
Hibch	15.0225	17.1005	0.186916	0.25755	0.402765	no
Hic1	2.3711	2.21606	-0.0975607	0.65165	0.780723	no
Hid1	70.5901	44.8569	-0.654136	5.00E-05	0.000236281	yes
Hif1a	29.0952	30.0655	0.0473248	0.73515	0.840433	no

Hif1an	12.351	11.5968	-0.0909014	0.52945	0.680574	no
Higd1a	24.8962	28.0301	0.171052	0.2604	0.406187	no
Higd2a	133.025	126.048	-0.077718	0.60215	0.741507	no
Hilpda	10.8271	12.3547	0.190408	0.35355	0.511127	no
Hinfp	8.77927	8.79596	0.00273853	0.9826	0.989978	no
Hint1	357.554	365.404	0.0313287	0.82335	0.898272	no
Hint2	23.4284	26.0815	0.154769	0.4382	0.596188	no
Hint3	29.9946	32.1219	0.0988531	0.5839	0.726576	no
Hip1	24.6124	22.5518	-0.126143	0.3711	0.529654	no
Hip1r	38.9863	52.8019	0.43762	0.0017	0.005774	yes
Hipk1	35.5841	48.5556	0.448406	0.00245	0.0079866	yes
Hipk2	2.13931	2.64464	0.305922	0.12755	0.235433	no
Hipk3	13.8937	12.6591	-0.134253	0.35695	0.514778	no
Hira	17.236	17.0785	-0.0132469	0.92865	0.961056	no
Hirip3	6.62611	6.29924	-0.0729829	0.6875	0.807191	no
Hist1h1b	2.42834	0.235012	-3.36917	0.01315	0.0346908	yes
Hist1h1c	29.2352	18.2904	-0.67662	6.00E-04	0.00228064	yes
Hist1h1d	3.63048	1.27108	-1.5141	0.002	0.0066678	yes
Hist1h1e	4.20692	2.42178	-0.796696	0.02645	0.063502	no
Hist1h2ab	1.25271	0.0961737	-3.70327	0.25255	0.396863	no
Hist1h2ae	2.42026	0.779653	-1.63426	0.02685	0.064307	no
Hist1h2ak	1.52394	0.50797	-1.58499	0.0679	0.140173	no
Hist1h2bb	1.5361	0.585839	-1.3907	0.11485	0.2164	no
Hist1h2bc	4.70802	2.61977	-0.845679	0.02475	0.0600125	no
Hist1h2be	2.19091	1.04017	-1.07472	0.18825	0.320657	no
Hist1h2bg	3.24311	2.93618	-0.143436	0.76155	0.85816	no
Hist1h2bh	1.59232	0.884575	-0.848076	0.20785	0.346206	no
Hist1h2bj	1.41033	1.0147	-0.47498	0.55515	0.702303	no
Hist1h2bl	1.01393	0.249675	-2.02184	0.17015	0.295706	no
Hist1h2bm	1.48672	0.409967	-1.85855	0.09	0.177228	no
Hist1h2bn	2.00066	0.121417	-4.04243	0.25235	0.396819	no
Hist1h3a	1.51454	1.33374	-0.1834	0.77305	0.865894	no
Hist1h3c	2.35575	0.508175	-2.21279	0.041	0.0919182	no
Hist1h3e	1.95703	0.849052	-1.20474	0.1118	0.211721	no
Hist1h3i	1.04292	0.502593	-1.05316	0.24805	0.393356	no
Hist1h4a	0.939486	1.02294	0.122782	0.9065	0.948565	no
Hist1h4b	1.56476	1.37959	-0.181695	0.7861	0.87475	no
Hist1h4c	3.8092	2.17339	-0.809538	0.18165	0.311641	no
Hist1h4d	3.13327	2.44648	-0.356962	0.56195	0.707987	no
Hist1h4f	2.07717	0.571213	-1.86252	0.13415	0.245135	no
Hist1h4h	1.99188	0.722269	-1.46352	0.0991	0.191903	no
Hist1h4i	19.4115	18.219	-0.0914665	0.73925	0.843304	no
Hist1h4k	2.37293	1.10875	-1.09774	0.1465	0.262131	no
Hist2h2ac	1.1504	1.94298	0.756134	0.2596	0.40527	no
Hist2h2bb	2.61157	1.35974	-0.941586	0.11225	0.212346	no
Hist2h2be	3.95166	4.50107	0.187809	0.34005	0.496512	no
Hist2h4	2.71084	1.4377	-0.914976	0.2025	0.339312	no
Hist3h2a	11.7711	15.0495	0.354473	0.1774	0.30568	no
Hist3h2ba	20.8628	18.6482	-0.161897	0.50765	0.661271	no
Hist4h4	1.56037	0.729517	-1.09687	0.19745	0.332866	no
Hivep1	6.14499	7.06681	0.201649	0.17935	0.308385	no
Hivep2	18.8567	19.9406	0.0806351	0.5659	0.7113	no
Hjurp	7.0761	5.26263	-0.427171	0.09395	0.183582	no
Hk1	43.417	40.8504	-0.0879102	0.53555	0.685904	no
Hk2	2.11782	2.72349	0.362878	0.0539	0.115867	no
Hlcs	5.43152	6.53381	0.266569	0.10285	0.197803	no
Hltf	26.4134	26.3601	-0.00291518	0.98345	0.99041	no

Hmbox1	6.57382	6.90103	0.0700794	0.68975	0.808891	no
Hmbs	15.2954	14.6373	-0.063446	0.7027	0.817693	no
Hmces	21.0743	22.9426	0.122545	0.4521	0.6096	no
Hmg20a	17.5411	18.034	0.0399795	0.78715	0.875546	no
Hmg20b	23.0036	24.8539	0.111608	0.483	0.638614	no
Hmga1	25.3073	28.0174	0.14677	0.6194	0.755434	no
Hmga1-rs1	40.0727	43.4039	0.115204	0.60845	0.746348	no
Hmgb1	191.852	174.954	-0.133015	0.3507	0.508069	no
Hmgb2	398.416	294.619	-0.435428	0.0035	0.010941	yes
Hmgb3	15.4108	13.0439	-0.240563	0.16425	0.28767	no
Hmgcl	24.6344	25.4478	0.0468616	0.771	0.864626	no
Hmgcr	23.6639	21.3979	-0.145221	0.30905	0.462412	no
Hmgn1	57.1984	64.794	0.179885	0.2273	0.370233	no
Hmgn2	384.914	296.859	-0.37476	0.0083	0.0232972	yes
Hmgn5	21.9378	19.7878	-0.148813	0.33895	0.495326	no
Hmgxb3	8.19827	9.0271	0.138942	0.36335	0.521546	no
Hmgxb4	12.5472	11.3238	-0.148014	0.3422	0.498867	no
Hmha1	642.4	640.769	-0.00366701	0.98505	0.991399	no
Hmmr	12.3952	6.82768	-0.86032	5.00E-05	0.000236281	yes
Hmox1	5.27767	5.25882	-0.00516378	0.98795	0.992966	no
Hmox2	39.649	33.7303	-0.233241	0.2297	0.373031	no
Hn1	267.6	213.478	-0.325996	0.0234	0.0571554	no
Hn1l	3.76371	2.83861	-0.406971	0.0578	0.122925	no
Hnrnpa0	105.819	113.78	0.104652	0.4622	0.619303	no
Hnrnpa1	134.757	154.566	0.19787	0.4193	0.577825	no
Hnrnpa2b1	442.126	421.678	-0.0683129	0.6428	0.773706	no
Hnrnpa3	66.5228	57.0487	-0.221656	0.4781	0.634154	no
Hnrnpab	152.693	169.245	0.148474	0.307	0.460096	no
Hnrnpc	152.856	145.291	-0.0732269	0.6092	0.746983	no
Hnrnpd	31.0909	27.5704	-0.173372	0.2299	0.373244	no
Hnrnpdl	71.7254	67.7109	-0.0830966	0.549	0.697159	no
Hnrnpf	537.198	532.534	-0.0125812	0.9319	0.962782	no
Hnrnph1	156.086	147.135	-0.0852064	0.5466	0.695112	no
Hnrnph2	45.6005	45.2195	-0.0121067	0.9312	0.962482	no
Hnrnph3	16.7811	16.3263	-0.0396384	0.8667	0.924908	no
Hnrnpk	366.345	350.982	-0.0618053	0.68145	0.802799	no
Hnrnpl	182.654	175.687	-0.0561059	0.7011	0.816679	no
Hnrnpll	19.8733	16.8211	-0.240559	0.1107	0.210123	no
Hnrnpm	171.302	172.911	0.0134849	0.92465	0.958829	no
Hnrnpr	18.8608	18.66	-0.0154447	0.91045	0.950781	no
Hnrnpu	192.323	189.674	-0.0200078	0.89155	0.939299	no
Hnrnpul1	114.267	110.105	-0.0535222	0.69945	0.815605	no
Hnrnpul2	87.2171	86.1718	-0.017396	0.90275	0.946212	no
Homer1	2.44935	2.94816	0.267417	0.26615	0.413247	no
Homez	1.98912	1.77447	-0.164742	0.40125	0.560269	no
Hook1	2.77432	3.37117	0.281112	0.14265	0.256562	no
Hook2	2.95051	2.80881	-0.0710029	0.74595	0.847836	no
Hook3	5.2408	5.1143	-0.0352514	0.80515	0.887012	no
Hopx	205.455	202.554	-0.0205182	0.8856	0.93602	no
Hoxb4	1.06834	1.10054	0.0428391	0.88975	0.938285	no
Hp1bp3	79.5801	84.7616	0.0910034	0.51915	0.671406	no
Hpcal1	65.234	64.9443	-0.00642141	0.9637	0.980396	no
Hprt	127.389	116.077	-0.134163	0.34175	0.498325	no
Hps1	21.8182	23.2058	0.0889512	0.54455	0.693342	no
Hps3	22.8959	21.9311	-0.0621099	0.6828	0.803692	no
Hps4	6.95547	8.32048	0.258518	0.13095	0.240464	no
Hps5	14.8983	14.0854	-0.0809479	0.58575	0.728005	no

Hps6	8.36555	8.73293	0.0620051	0.7096	0.822684	no
Hpse	1.40435	0.732588	-0.938828	0.0024	0.00784462	yes
Hras	15.4615	15.3064	-0.014538	0.9289	0.96116	no
Hrh2	17.8243	18.0282	0.0164078	0.9247	0.958862	no
Hrsp12	16.557	20.1595	0.284018	0.1184	0.221822	no
Hs1bp3	2.68587	3.30558	0.299514	0.1623	0.284855	no
Hs2st1	4.33547	4.20368	-0.0445363	0.7993	0.88327	no
Hs3st3b1	5.35356	7.1988	0.427259	0.00665	0.0191807	yes
Hs6st1	6.54919	9.04024	0.465045	0.0042	0.0128487	yes
Hsbp1	55.7455	51.6515	-0.110046	0.4629	0.62004	no
Hscb	59.2422	53.1579	-0.156341	0.32035	0.47503	no
Hsd11b1	137.496	127.874	-0.104666	0.4602	0.617443	no
Hsd17b10	59.9419	59.5886	-0.00852928	0.95535	0.976122	no
Hsd17b11	22.8606	34.0309	0.573978	5.00E-05	0.000236281	yes
Hsd17b12	18.7808	23.4169	0.318293	0.04015	0.0903455	no
Hsd17b4	23.426	23.3371	-0.00548589	0.9706	0.983969	no
Hsd17b7	1.31061	1.40704	0.102422	0.65495	0.783148	no
Hsd3b7	1.24817	1.39042	0.155707	0.62645	0.760851	no
Hsdl1	15.784	17.0747	0.113401	0.45925	0.616579	no
Hsdl2	14.9856	14.2966	-0.0679043	0.6615	0.788123	no
Hsf1	37.4671	36.92	-0.0212237	0.88545	0.935946	no
Hsf2	2.46824	2.13118	-0.211831	0.37625	0.535057	no
Hsf4	3.64845	3.03896	-0.263704	0.2817	0.431534	no
Hsh2d	17.3961	18.4492	0.0847989	0.6264	0.760816	no
Hsp90aa1	163.988	164.903	0.00802122	0.95395	0.975285	no
Hsp90ab1	628.692	620.145	-0.0197485	0.89965	0.944435	no
Hsp90b1	219.835	206.276	-0.0918448	0.5259	0.677368	no
Hspa13	4.86179	5.07943	0.0631783	0.71455	0.826089	no
Hspa14	56.1772	48.9091	-0.199882	0.18355	0.314228	no
Hspa2	3.61286	3.99618	0.145478	0.47665	0.632861	no
Hspa4	38.1758	36.8758	-0.0499832	0.71835	0.828622	no
Hspa4l	11.4013	8.87325	-0.361661	0.02235	0.0549252	no
Hspa5	340.957	390.383	0.195301	0.18205	0.312168	no
Hspa8	1664.93	1470.06	-0.179593	0.32385	0.478962	no
Hspa9	40.8166	48.2117	0.240225	0.09045	0.17799	no
Hspb11	16.6853	20.6579	0.308116	0.1755	0.303027	no
Hspb6	2.16969	1.83276	-0.243467	0.44715	0.604612	no
Hspbap1	2.54829	2.9367	0.204666	0.28375	0.433856	no
Hspbp1	15.6006	20.3822	0.385705	0.0229	0.0561128	no
Hspd1	87.8173	84.9929	-0.0471628	0.74065	0.844205	no
Hspe1	145.235	160.397	0.143253	0.32035	0.47503	no
Hsph1	64.8433	68.3205	0.0753617	0.5888	0.730625	no
Htatip2	36.4866	34.4924	-0.0810868	0.61155	0.748901	no
Htatsf1	27.1132	27.8759	0.0400261	0.78715	0.875546	no
Htra2	11.0703	10.7547	-0.0417372	0.81485	0.892849	no
Htt	8.93893	9.44528	0.0794919	0.576	0.719867	no
Hus1	4.25172	4.57237	0.104895	0.54395	0.692878	no
Huwe1	39.7352	45.1232	0.183452	0.19595	0.330819	no
Hvcn1	21.3084	24.3054	0.189856	0.36995	0.528526	no
Hyal2	1.31722	1.39706	0.0849028	0.7914	0.878321	no
Hyi	14.0162	14.4762	0.0465835	0.94155	0.968358	no
Hyls1	8.86578	7.87187	-0.17154	0.56975	0.71446	no
Hyou1	24.2454	27.0387	0.157316	0.2696	0.417365	no
Hypk	197.216	187.52	-0.0727306	0.62015	0.755957	no
I830012O16Rik	1.22453	0.768608	-0.671911	0.0621	0.130455	no
Iah1	49.8046	47.9014	-0.0562102	0.8569	0.918993	no
Iars	17.6838	18.3423	0.0527442	0.70815	0.821722	no

Iars2	26.5748	26.1202	-0.0248947	0.8952	0.941528	no
Iba57	2.78035	2.80161	0.0109892	0.9545	0.975691	no
Ibtk	9.88617	10.6196	0.10325	0.4839	0.639384	no
Ica1	1.11553	0.749274	-0.574166	0.1525	0.270916	no
Icam1	50.8683	47.7504	-0.0912541	0.5239	0.675642	no
Icam2	60.01	60.109	0.00237815	0.9855	0.991597	no
Ick	1.39492	1.74472	0.322815	0.10265	0.197499	no
Icmt	11.7673	11.9608	0.023529	0.8743	0.929435	no
Icos	36.7467	48.1246	0.389161	0.0063	0.0183103	yes
Icosl	3.77092	5.24455	0.475904	0.01475	0.0382892	yes
Ict1	95.1559	79.3237	-0.262541	0.0852	0.169316	no
Id2	641.274	671.386	0.0662015	0.6503	0.779569	no
Id3	6.76446	10.0557	0.571965	0.01595	0.0409554	yes
Ide	31.9716	29.3644	-0.122722	0.3812	0.540192	no
Idh1	4.31623	3.98757	-0.114264	0.577	0.720722	no
Idh2	21.1899	23.6342	0.157496	0.31255	0.466245	no
Idh3a	48.9148	52.3426	0.0977152	0.49625	0.650805	no
Idh3b	117.743	108.493	-0.118043	0.53245	0.68295	no
Idh3g	78.8771	76.5777	-0.0426826	0.76745	0.862111	no
Idi1	11.8399	9.8874	-0.259998	0.1078	0.205619	no
Idnk	31.4229	32.2919	0.0393555	0.7925	0.878934	no
Ids	13.2555	13.4709	0.0232497	0.8724	0.928391	no
Idua	6.876	6.42639	-0.0975616	0.7127	0.824828	no
Ier2	203.572	204.585	0.00716156	0.9596	0.978255	no
Ier3	18.5007	32.5547	0.815287	5.00E-05	0.000236281	yes
Ier3ip1	40.2658	43.7113	0.118449	0.43465	0.592798	no
Ier5	102.123	102.417	0.00415575	0.97595	0.986779	no
Iffo1	16.237	15.7862	-0.0406218	0.7856	0.874428	no
Iffo2	3.71118	3.32946	-0.15659	0.3652	0.523541	no
Ifi203	130.088	125.081	-0.0566273	0.6828	0.803692	no
Ifi27	108.905	91.8177	-0.246232	0.1457	0.26095	no
Ifi27l2a	105.196	69.5113	-0.597761	0.06675	0.138199	no
Ifi30	21.0546	17.1688	-0.294347	0.1089	0.207297	no
Ifi35	46.8917	43.8944	-0.0952934	0.53015	0.681102	no
Ifi47	114.092	97.2921	-0.229806	0.12485	0.231412	no
Ifih1	3.39377	3.24803	-0.0633238	0.715	0.826433	no
Ifit1	3.29997	2.87959	-0.196586	0.3553	0.512964	no
Ifit2	18.3697	10.3625	-0.825956	5.00E-05	0.000236281	yes
Ifit3	7.04173	4.84917	-0.53819	0.00755	0.0214505	yes
Ifitm1	16.4499	79.1406	2.26634	5.00E-05	0.000236281	yes
Ifitm10	9.14807	5.62622	-0.701302	1.00E-04	0.000450026	yes
Ifitm2	1.24983	3.27581	1.39012	0.011	0.0297986	yes
Ifitm3	2.53204	5.10329	1.01113	0.0103	0.0281508	yes
Ifnar1	31.8104	31.8223	0.000536762	0.9971	0.997863	no
Ifnar2	52.6094	48.9047	-0.105346	0.53875	0.688416	no
Ifng	324.236	275.086	-0.23716	0.0989	0.1916	no
Ifngr1	136.138	141.509	0.0558274	0.6901	0.809087	no
Ifrd1	37.0989	40.9849	0.143719	0.33405	0.490033	no
Ifrd2	4.7285	6.70938	0.504796	0.01245	0.0331127	yes
Ift140	5.20102	5.1793	-0.00603632	0.97125	0.984246	no
Ift172	1.79461	2.32645	0.37446	0.4591	0.616471	no
Ift20	52.4179	51.3617	-0.0293653	0.85765	0.919546	no
Ift27	20.1715	15.1954	-0.40868	0.02525	0.0610571	no
Ift46	7.08689	5.41982	-0.386908	0.02575	0.0620811	no
Ift52	33.393	30.6837	-0.122074	0.4079	0.566774	no
Ift57	1.08285	1.45833	0.429489	0.13875	0.251148	no
Ift74	1.36799	1.36907	0.00113796	0.99215	0.995139	no

Ift80	4.68955	5.78101	0.301873	0.07825	0.157662	no
Igbp1	92.703	87.3978	-0.0850187	0.5478	0.696086	no
Igf2bp3	2.90277	2.08193	-0.479508	0.0169	0.0430564	yes
Igf2r	41.4503	51.0266	0.299864	0.0354	0.0811656	no
Igflr1	18.8983	25.6255	0.439326	0.01005	0.0275561	yes
Ighmbp2	4.95247	5.1204	0.0481089	0.76625	0.861366	no
Igip	2.26356	2.47425	0.1284	0.6654	0.790887	no
Igsf10	1.36682	0.732738	-0.899451	0.0977	0.189739	no
Igsf8	5.97767	6.30471	0.0768471	0.68325	0.804007	no
Igtp	39.7332	37.9117	-0.0677036	0.63635	0.768735	no
Ik	102.766	98.4142	-0.0624249	0.65895	0.786266	no
Ikbip	1.99861	1.20796	-0.726426	0.06025	0.12733	no
Ikbkap	5.81082	6.57267	0.177738	0.38965	0.548783	no
Ikbkb	56.5189	65.4411	0.211463	0.1333	0.243891	no
Ikbke	51.9235	69.6512	0.423762	0.00235	0.00770045	yes
Ikbkg	11.071	11.5603	0.0623837	0.74955	0.850353	no
Ikzf1	129.331	120.059	-0.107328	0.45515	0.612858	no
Ikzf2	0.613104	1.69963	1.47102	5.00E-05	0.000236281	yes
Ikzf3	156.27	119.476	-0.387318	0.00675	0.0194405	yes
Ikzf5	13.8647	13.454	-0.0433812	0.7735	0.866246	no
Il10ra	42.125	39.6492	-0.0873846	0.537	0.687126	no
Il10rb	58.6911	54.9912	-0.0939422	0.507	0.660729	no
Il11ra1	2.02948	2.52265	0.313833	0.28125	0.431051	no
Il12rb1	20.8716	22.0285	0.0778316	0.60775	0.745839	no
Il12rb2	22.4613	20.6314	-0.122602	0.4092	0.568222	no
Il15	1.78765	2.60968	0.545807	0.088	0.173998	no
Il15ra	17.0552	16.3666	-0.0594569	0.72575	0.833752	no
Il16	56.1641	54.2834	-0.0491365	0.7264	0.834268	no
Il17ra	100.596	102.512	0.0272177	0.8494	0.914061	no
Il18	2.71697	4.21876	0.634822	0.0529	0.114005	no
Il18bp	2.75196	2.38831	-0.20447	0.4726	0.629153	no
Il18r1	168.659	248.039	0.556462	0.00045	0.0017621	yes
Il18rap	208.488	129.648	-0.685364	5.00E-05	0.000236281	yes
Il1rap	5.98491	5.30208	-0.17477	0.321	0.475844	no
Il1rl2	1.46024	1.88268	0.366574	0.20735	0.345569	no
Il20rb	1.96269	1.94768	-0.0110765	0.9593	0.978091	no
Il21r	78.3096	74.2395	-0.0770019	0.5878	0.729833	no
Il27ra	89.1259	112.17	0.331777	0.02045	0.0508419	no
Il2ra	7.14213	7.6711	0.103081	0.5094	0.662877	no
Il2rb	219.911	247.828	0.17242	0.2353	0.379601	no
Il2rg	526.818	517.588	-0.0255024	0.86075	0.921345	no
Il31ra	0.594796	1.57716	1.40686	5.00E-05	0.000236281	yes
Il3ra	9.94034	8.32187	-0.256388	0.18715	0.319153	no
Il4ra	13.426	13.1964	-0.0248823	0.86455	0.923577	no
Il6ra	5.20126	14.6665	1.49559	5.00E-05	0.000236281	yes
Il6st	8.71063	10.5825	0.280836	0.0637	0.133079	no
Il7r	78.0996	156.146	0.99951	5.00E-05	0.000236281	yes
Ildr1	8.00746	7.70989	-0.0546342	0.7443	0.84682	no
Ilf2	29.9582	27.7621	-0.109834	0.57445	0.718465	no
Ilf3	25.6796	23.6958	-0.11599	0.42545	0.583838	no
Ilk	130.824	115.543	-0.1792	0.3398	0.49624	no
Ilkap	60.933	57.2714	-0.0894099	0.5398	0.689458	no
Ilvbl	17.8389	17.6564	-0.0148362	0.92345	0.958246	no
Immp1l	29.5584	28.5776	-0.0486824	0.77445	0.86698	no
Immp2l	1.74079	1.79339	0.0429456	0.9124	0.952014	no
Immt	37.6939	34.7016	-0.11933	0.4091	0.568128	no
Imp3	90.1384	87.7614	-0.0385543	0.7904	0.877763	no

Imp4	28.1434	29.2862	0.0574246	0.698	0.814708	no
Impa1	42.9027	39.2633	-0.127887	0.37695	0.535826	no
Impa2	12.7971	14.955	0.224813	0.2029	0.339821	no
Impact	9.969	9.99799	0.00418937	0.97925	0.988366	no
Impad1	19.188	18.806	-0.0290149	0.83955	0.908044	no
Impdh1	44.5345	41.0377	-0.117973	0.40715	0.565954	no
Impdh2	49.0221	46.2951	-0.0825748	0.572	0.716352	no
Inadl	0.699754	1.28121	0.872591	0.00455	0.0137968	yes
Incenp	40.4145	26.754	-0.595117	5.00E-05	0.000236281	yes
Inf2	16.4497	17.6367	0.100517	0.48635	0.641553	no
Ing1	32.3349	29.9519	-0.110443	0.4482	0.605626	no
Ing2	11.9294	10.3787	-0.200902	0.2183	0.359237	no
Ing3	12.9929	11.7655	-0.143155	0.34735	0.504489	no
Ing4	43.9805	39.9146	-0.139947	0.3511	0.508471	no
Ing5	9.47651	8.59476	-0.140898	0.36245	0.52072	no
Inip	10.9641	10.0182	-0.130159	0.41665	0.57524	no
Ino80	13.6483	13.5065	-0.0150612	0.91895	0.955755	no
Ino80b	42.7267	39.4432	-0.115362	0.45765	0.615109	no
Ino80c	25.6948	22.3633	-0.200338	0.1866	0.318377	no
Ino80d	11.3087	11.6558	0.0436107	0.7561	0.854502	no
Ino80e	37.2324	37.4953	0.0101539	0.9513	0.973958	no
Inpp1	37.6311	34.2716	-0.134911	0.3714	0.529943	no
Inpp4a	15.7731	12.7913	-0.302304	0.04585	0.101087	no
Inpp4b	5.87154	7.80353	0.410387	0.0132	0.0348032	yes
Inpp5a	1.08321	1.18309	0.127256	0.66045	0.787391	no
Inpp5b	36.142	32.5957	-0.148994	0.29075	0.441745	no
Inpp5d	54.0422	55.5731	0.0402983	0.7746	0.867107	no
Inpp5e	19.4112	19.6114	0.0148016	0.94055	0.967851	no
Inpp5f	3.24137	2.65928	-0.285569	0.12715	0.234836	no
Inpp5k	55.2396	49.6103	-0.155064	0.27685	0.425907	no
Inppl1	4.4636	4.41186	-0.0168217	0.92255	0.957904	no
Insig1	14.4006	12.9837	-0.149428	0.3453	0.502214	no
Insig2	7.03089	6.72412	-0.0643601	0.7151	0.826494	no
Insl3	6.14567	10.4897	0.771332	0.58035	0.723536	no
Insl6	45.6589	22.7227	-1.00676	5.00E-05	0.000236281	yes
Insr	0.651325	1.04833	0.686639	0.00145	0.00501121	yes
Ints1	20.5449	20.8252	0.0195457	0.89095	0.938933	no
Ints10	18.3101	18.927	0.0478012	0.7563	0.854564	no
Ints12	8.33138	7.5269	-0.1465	0.3821	0.541152	no
Ints2	3.45426	4.03441	0.223982	0.19345	0.327413	no
Ints3	24.4254	25.5175	0.0631066	0.6615	0.788123	no
Ints4	15.3227	15.4925	0.0159003	0.91265	0.952149	no
Ints5	11.6052	11.4404	-0.0206242	0.8915	0.939274	no
Ints6	11.1158	12.5661	0.176932	0.2341	0.378106	no
Ints7	21.5866	21.4834	-0.0069074	0.9614	0.979284	no
Ints8	22.5517	21.7508	-0.0521691	0.7272	0.834752	no
Ints9	19.5312	18.142	-0.106445	0.48645	0.641637	no
Invs	4.49044	4.54835	0.0184841	0.95115	0.973847	no
Ip6k1	46.084	46.6808	0.018563	0.8942	0.940925	no
Ip6k2	18.175	17.2194	-0.0779263	0.6255	0.760155	no
Ipcef1	39.2728	43.4398	0.145486	0.33845	0.494831	no
Ipmk	6.615	6.23504	-0.085341	0.5863	0.728458	no
Ipo11	7.18858	7.14807	-0.00815366	0.95905	0.977972	no
Ipo13	11.9345	13.4401	0.1714	0.2644	0.411163	no
Ipo4	5.95278	8.37047	0.491746	0.00325	0.0102567	yes
Ipo5	29.652	28.9103	-0.0365493	0.80195	0.884965	no
Ipo7	35.0557	33.8526	-0.0503818	0.7202	0.829997	no

Ipo8	16.3114	16.6653	0.0309637	0.8295	0.902081	no
Ipo9	14.6754	13.256	-0.146758	0.315	0.46881	no
Ipp	10.1454	9.24101	-0.134704	0.4342	0.592413	no
Ippk	6.05832	7.16424	0.241896	0.1672	0.291758	no
Iqcc	7.60572	8.71234	0.195975	0.2736	0.422104	no
Iqce	13.2339	14.3277	0.114566	0.4376	0.595624	no
Iqcg	10.0618	10.035	-0.00385009	0.988	0.992966	no
Iqgap1	120.111	127.629	0.0875851	0.5549	0.702113	no
Iqgap2	53.4956	61.4205	0.199299	0.1741	0.301118	no
Iqgap3	2.10823	1.06282	-0.988141	5.00E-05	0.000236281	yes
Iqsec1	16.559	14.2921	-0.212401	0.1393	0.252014	no
Irak1	35.9304	35.9041	-0.00105823	0.99325	0.995761	no
Irak2	11.1483	11.8592	0.0891854	0.5693	0.714061	no
Irak3	0.402208	1.67999	2.06244	5.00E-05	0.000236281	yes
Irak4	17.8121	17.8514	0.00317815	0.98445	0.990979	no
Ireb2	14.768	15.7996	0.0974153	0.49695	0.651396	no
Irf1	159.006	156.225	-0.0254518	0.85815	0.919829	no
Irf2	59.3751	49.6248	-0.258797	0.07335	0.149362	no
Irf2bp1	18.4235	18.416	-0.000589709	0.99725	0.997929	no
Irf2bp2	19.6389	25.0193	0.349327	0.01325	0.0349155	yes
Irf2bpl	25.3798	23.586	-0.105754	0.45615	0.613736	no
Irf3	67.1673	64.346	-0.0619084	0.65575	0.783786	no
Irf4	8.68229	9.47048	0.125362	0.40595	0.564909	no
Irf5	3.02955	3.24877	0.100789	0.6608	0.787701	no
Irf7	38.3038	33.8565	-0.178052	0.2333	0.377298	no
Irf8	8.18496	7.85692	-0.0590118	0.7237	0.832372	no
Irf9	41.644	39.0268	-0.0936427	0.5213	0.673372	no
Irgm1	30.5759	28.9843	-0.0771223	0.6079	0.745881	no
Irgm2	6.69691	6.74617	0.0105724	0.9498	0.972973	no
Irgq	4.02122	4.23943	0.0762357	0.64245	0.773482	no
Isca1	28.7677	29.0791	0.0155309	0.9202	0.956519	no
Isca2	26.9116	23.7488	-0.180376	0.31135	0.464976	no
Iscu	43.6018	49.0895	0.171029	0.28045	0.430236	no
Isg15	16.1873	18.4122	0.185801	0.34965	0.506995	no
Isg20	5.91708	6.31761	0.0944939	0.68535	0.805619	no
Isg20l2	31.9315	29.1962	-0.1292	0.36845	0.527001	no
Isl2	14.9449	27.1546	0.861544	5.00E-05	0.000236281	yes
Isoc1	9.46183	11.9594	0.33795	0.0415	0.0928711	no
Isoc2a	18.389	23.0398	0.325285	0.06205	0.130384	no
Isoc2b	16.3101	16.1984	-0.00991318	0.9582	0.977478	no
Ist1	53.6101	50.1323	-0.0967649	0.49875	0.652992	no
Isy1	69.8137	67.7143	-0.0440497	0.75795	0.855743	no
Isyna1	42.2752	49.288	0.221423	0.1324	0.242603	no
Itch	25.2571	26.7737	0.0841273	0.5506	0.698575	no
Itfg1	20.5482	21.4879	0.0645112	0.66635	0.79163	no
Itfg2	9.2552	8.27336	-0.16179	0.3598	0.517766	no
Itfg3	10.7151	11.6772	0.124037	0.4371	0.59507	no
Itga1	13.7167	17.7013	0.367923	0.02155	0.0532373	no
Itga2	3.92997	3.77526	-0.0579453	0.7474	0.848815	no
Itga4	330.06	298.427	-0.145348	0.41915	0.577742	no
Itga6	13.2479	15.1771	0.196138	0.1692	0.29436	no
Itgad	1.87572	1.47236	-0.349313	0.12405	0.230219	no
Itgae	0.868945	2.41284	1.4734	0.0064	0.018565	yes
Itgal	445.374	363.981	-0.291153	0.07585	0.153615	no
Itgam	48.0976	27.647	-0.798842	5.00E-05	0.000236281	yes
Itgav	4.67693	4.42855	-0.0787259	0.6186	0.754811	no
Itgax	249.433	233.856	-0.0930353	0.52975	0.680848	no

Itgb1	612.028	786.278	0.361441	0.0424	0.0946331	no
Itgb1bp1	37.5744	33.3013	-0.174175	0.25875	0.404256	no
Itgb2	597.001	425.541	-0.488435	0.0019	0.00637506	yes
Itgb3	4.65258	11.0521	1.24822	5.00E-05	0.000236281	yes
Itgb3bp	2.65102	3.31931	0.324335	0.13455	0.245688	no
Itgb7	477.65	589.359	0.303192	0.04965	0.108137	no
Itk	158.492	160.988	0.0225494	0.88695	0.936647	no
Itm2a	7.0538	4.86836	-0.534965	0.01065	0.0289694	yes
Itm2b	565.17	542.682	-0.0585769	0.6881	0.807668	no
Itm2c	38.203	43.4793	0.186641	0.20405	0.341294	no
Itpa	48.2001	48.1858	-0.000426804	0.99725	0.997929	no
Itpk1	27.659	28.3261	0.0343857	0.81845	0.895251	no
Itpkb	41.5842	46.6203	0.164921	0.24055	0.385698	no
Itpkc	5.48635	5.65008	0.0424231	0.8121	0.891358	no
Itpr1	11.477	11.5468	0.00874694	0.9521	0.974325	no
Itpr2	13.7737	14.1913	0.0430892	0.75755	0.855483	no
Itpr3	30.0868	36.1665	0.265525	0.0566	0.12081	no
Itprip	16.8697	18.0308	0.0960231	0.51165	0.664813	no
Itpripl1	9.54053	8.64274	-0.142581	0.36995	0.528526	no
Itpripl2	2.75388	3.04639	0.145633	0.39435	0.553525	no
Itsn1	1.46216	2.93635	1.00592	5.00E-05	0.000236281	yes
Itsn2	51.1787	50.5839	-0.016863	0.9125	0.952077	no
Ivd	12.6707	15.2704	0.269247	0.1037	0.199134	no
Ivns1abp	95.2855	99.2757	0.0591837	0.6766	0.799206	no
Iws1	7.6985	7.62649	-0.013559	0.92295	0.958094	no
Izumo4	23.8594	23.0001	-0.0529195	0.76325	0.85939	no
Jade1	5.41586	5.83515	0.107579	0.50235	0.656463	no
Jade2	16.9064	15.2254	-0.151085	0.29135	0.442324	no
Jade3	2.45567	2.4962	0.0236183	0.90415	0.947103	no
Jagn1	16.1666	17.53	0.11681	0.51695	0.669604	no
Jak1	449.391	341.468	-0.396222	0.0142	0.037055	yes
Jak2	41.829	47.2737	0.176534	0.2125	0.352214	no
Jak3	85.4889	90.1714	0.0769335	0.59315	0.734229	no
Jakmip1	42.994	56.1904	0.386189	0.0081	0.022802	yes
Jarid2	12.4796	12.6879	0.0238795	0.86995	0.926916	no
Jazf1	1.20848	0.991484	-0.285531	0.3219	0.476807	no
Jdp2	5.5509	7.35872	0.406732	0.0435	0.0966968	no
Jkamp	16.2477	13.8126	-0.234257	0.1547	0.273997	no
Jmjd1c	16.8861	18.3663	0.121225	0.389	0.548141	no
Jmjd4	4.33682	4.79312	0.144327	0.3982	0.557234	no
Jmjd6	35.8238	36.6711	0.0337241	0.8248	0.899228	no
Jmjd7	13.1122	13.7767	0.071327	0.7845	0.873796	no
Jmjd8	10.5506	11.2287	0.0898656	0.7555	0.854083	no
Jmy	3.19416	3.41198	0.0951723	0.55205	0.699711	no
Josd1	13.3456	15.9301	0.255393	0.088	0.173998	no
Josd2	30.5209	27.6893	-0.140471	0.4106	0.569505	no
Jtb	66.6754	69.6803	0.0635958	0.6685	0.793209	no
Jun	63.0886	80.1364	0.345079	0.01695	0.043162	yes
Junb	392.485	419.784	0.0970109	0.50355	0.657666	no
Jund	140.43	147.949	0.0752422	0.5969	0.737254	no
Jup	1.52924	0.36869	-2.05233	5.00E-05	0.000236281	yes
Kank3	0.9432	1.09652	0.2173	0.47475	0.631146	no
Kansl1	31.6804	33.1494	0.0653957	0.6424	0.773482	no
Kansl1l	2.22323	2.44105	0.134845	0.7037	0.818479	no
Kansl2	18.187	20.1831	0.150241	0.30875	0.462081	no
Kansl3	20.4999	20.9478	0.0311809	0.83265	0.903961	no
Kars	90.0772	81.2513	-0.14877	0.29585	0.447481	no

Kat2a	20.2544	23.2307	0.1978	0.1808	0.310417	no
Kat2b	12.174	13.0963	0.105363	0.4905	0.645459	no
Kat5	29.3798	25.8421	-0.1851	0.226	0.368677	no
Kat6a	28.3487	28.9162	0.028596	0.839	0.907699	no
Kat6b	2.93351	3.42614	0.223956	0.17285	0.299433	no
Kat7	21.3046	18.7678	-0.18291	0.20175	0.338329	no
Kat8	17.4887	18.9517	0.115902	0.48305	0.638632	no
Katna1	47.1937	40.4453	-0.222623	0.1288	0.237263	no
Katnal1	0.883186	1.13581	0.362931	0.1193	0.223229	no
Katnb1	23.8032	19.5752	-0.282128	0.0529	0.114005	no
Katnbl1	4.94194	4.20364	-0.233436	0.22165	0.363302	no
Kbtbd11	33.407	52.5072	0.652367	5.00E-05	0.000236281	yes
Kbtbd2	27.0779	25.5452	-0.0840612	0.55595	0.702988	no
Kbtbd3	4.40991	4.36317	-0.0153743	0.9395	0.96725	no
Kbtbd4	12.0684	11.3108	-0.0935333	0.57215	0.716464	no
Kbtbd7	3.51435	3.23167	-0.120979	0.49965	0.653879	no
Kbtbd8	1.6902	1.78281	0.0769544	0.717	0.82766	no
Kcmf1	20.7301	20.2607	-0.0330406	0.82675	0.900589	no
Kcna3	12.1293	18.1602	0.582281	3.00E-04	0.00122185	yes
Kcnab2	162.846	144.592	-0.171519	0.23345	0.377445	no
Kcnc1	0.661138	1.39142	1.07353	5.00E-05	0.000236281	yes
Kcnj8	7.84622	2.49032	-1.65567	5.00E-05	0.000236281	yes
Kcnk5	29.6901	23.9427	-0.310397	0.0314	0.073338	no
Kcnk6	4.20777	4.28657	0.0267703	0.88165	0.933705	no
Kcnk7	3.34231	2.67203	-0.322906	0.27675	0.425791	no
Kcnmb4	1.243	1.49047	0.261939	0.55195	0.699659	no
Kcnn4	61.0591	71.1041	0.219727	0.12315	0.228808	no
Kctd1	2.49978	2.38165	-0.0698427	0.752	0.851846	no
Kctd10	48.7219	48.6091	-0.00334483	0.9804	0.988791	no
Kctd11	1.87838	2.06767	0.138521	0.57225	0.716538	no
Kctd12	1.14323	2.88713	1.33653	5.00E-05	0.000236281	yes
Kctd13	10.8054	11.1752	0.04855	0.7863	0.874903	no
Kctd17	5.53386	4.90175	-0.17499	0.4135	0.572369	no
Kctd18	17.2269	17.0223	-0.0172323	0.91115	0.95123	no
Kctd2	36.577	28.2787	-0.371221	0.0144	0.0375076	yes
Kctd20	33.7078	30.96	-0.122675	0.3997	0.558712	no
Kctd3	6.54763	6.73548	0.0408069	0.80825	0.888943	no
Kctd5	18.035	17.58	-0.0368647	0.81295	0.891805	no
Kctd6	2.61908	2.37687	-0.139996	0.60205	0.7415	no
Kctd9	13.2884	11.7868	-0.172991	0.26035	0.406127	no
Kdelc1	4.0879	5.17819	0.34109	0.0882	0.17434	no
Kdelc2	2.47604	2.82141	0.188384	0.35645	0.514225	no
Kdelr1	52.9538	44.0545	-0.265443	0.06875	0.141667	no
Kdelr2	18.5186	17.7089	-0.0645001	0.68695	0.806774	no
Kdm1a	29.3778	30.2746	0.0433797	0.7625	0.85886	no
Kdm1b	10.9462	10.7279	-0.0290661	0.93605	0.965275	no
Kdm2a	24.5065	27.6873	0.176059	0.21135	0.350777	no
Kdm2b	14.9094	13.5693	-0.135881	0.37445	0.533142	no
Kdm3a	29.101	28.6087	-0.0246156	0.86655	0.924804	no
Kdm3b	29.8829	32.2435	0.109689	0.43775	0.595736	no
Kdm4a	46.5902	48.2123	0.0493755	0.7281	0.835296	no
Kdm4b	12.7288	13.4066	0.0748454	0.621	0.756653	no
Kdm4c	17.2982	17.1776	-0.0100875	0.94565	0.970482	no
Kdm5a	17.3575	18.4335	0.086771	0.55485	0.702062	no
Kdm5b	5.65317	6.97652	0.303447	0.053	0.1142	no
Kdm5c	13.5679	13.5066	-0.00652722	0.9625	0.979895	no
Kdm5d	13.0888	12.4625	-0.0707341	0.6284	0.76234	no

Kdm6a	14.3797	15.3831	0.0973125	0.49975	0.653973	no
Kdm6b	6.62932	6.69162	0.0134963	0.93275	0.963294	no
Kdm7a	15.8476	17.2699	0.124001	0.37735	0.536232	no
Kdm8	5.74159	4.38696	-0.388229	0.0465	0.102286	no
Kdsr	3.72473	4.23848	0.186411	0.2713	0.419307	no
Keap1	28.9377	26.3877	-0.133088	0.356	0.513775	no
Khdc3	1.67234	1.62284	-0.0433481	0.88655	0.936435	no
Khdrbs1	73.6272	74.8628	0.0240103	0.8654	0.92408	no
Khk	4.17155	4.22206	0.0173624	0.95115	0.973847	no
Khnyn	35.3303	36.8189	0.0595423	0.6756	0.798466	no
Khsrp	33.3956	33.5369	0.00609211	0.9666	0.981874	no
Kidins220	32.7401	31.9071	-0.0371802	0.78895	0.876884	no
Kif11	28.3436	15.8706	-0.836668	5.00E-05	0.000236281	yes
Kif13b	9.79984	10.8607	0.148287	0.3314	0.487087	no
Kif14	3.37121	2.09173	-0.688568	5.00E-05	0.000236281	yes
Kif15	10.9386	5.84334	-0.904558	5.00E-05	0.000236281	yes
Kif16b	5.58922	5.49179	-0.0253718	0.8781	0.931847	no
Kif18a	4.59938	2.60918	-0.817846	5.00E-05	0.000236281	yes
Kif18b	12.1411	6.49798	-0.901839	5.00E-05	0.000236281	yes
Kif1b	7.35797	7.25863	-0.0196095	0.89555	0.941854	no
Kif1c	14.9887	14.7451	-0.0236392	0.86775	0.925637	no
Kif20a	15.4336	8.12216	-0.926139	0.0189	0.0474682	yes
Kif20b	7.08616	5.31859	-0.41396	0.0089	0.0247598	yes
Kif21b	126.013	112.091	-0.168898	0.25765	0.402877	no
Kif22	30.2662	18.9324	-0.676849	5.00E-05	0.000236281	yes
Kif23	19.9777	13.5893	-0.555916	0.00035	0.0014053	yes
Kif2a	28.9455	26.9372	-0.103735	0.46915	0.625787	no
Kif2c	10.312	5.29247	-0.962316	5.00E-05	0.000236281	yes
Kif3b	18.8151	19.3843	0.0429949	0.76015	0.857308	no
Kif3c	3.08631	2.95988	-0.0603405	0.7699	0.863818	no
Kif4	7.53625	4.02408	-0.905187	5.00E-05	0.000236281	yes
Kif5b	42.3411	43.9588	0.0540933	0.70235	0.817447	no
Kif7	1.05841	1.31858	0.317087	0.44295	0.600774	no
Kifap3	0.551297	1.15877	1.0717	0.00055	0.0021091	yes
Kifc1	16.002	9.4599	-0.758352	5.00E-05	0.000236281	yes
Kifc2	3.66444	3.8631	0.0761696	0.6956	0.812956	no
Kifc5b	4.36593	2.83476	-0.62306	0.0029	0.00927196	yes
Kin	27.4784	28.6967	0.0625873	0.6909	0.809662	no
Kiz	7.55908	7.86061	0.0564315	0.7538	0.852913	no
Klc1	33.6256	32.2554	-0.0600208	0.6968	0.813846	no
Klc2	4.58979	4.85259	0.080328	0.66905	0.793649	no
Klc3	5.82891	5.95821	0.031653	0.93165	0.962679	no
Klc4	8.97639	8.37773	-0.0995747	0.5615	0.707609	no
Klf10	15.8715	12.7186	-0.3195	0.03715	0.0845382	no
Klf11	6.63904	5.99721	-0.146685	0.38015	0.539169	no
Klf12	1.65996	2.53306	0.609734	0.00235	0.00770045	yes
Klf13	50.2875	55.5564	0.143752	0.30785	0.461086	no
Klf16	13.9726	13.091	-0.0940176	0.5496	0.697721	no
Klf2	311.208	261.292	-0.252216	0.0736	0.149776	no
Klf3	83.6964	79.2611	-0.0785515	0.5775	0.721078	no
Klf4	6.25354	12.574	1.0077	5.00E-05	0.000236281	yes
Klf6	110.496	123.205	0.157062	0.2692	0.416947	no
Klf7	8.24322	8.8888	0.108781	0.5898	0.731493	no
Klf8	1.51809	1.71253	0.173873	0.43445	0.592628	no
Klhdc1	9.07095	13.4051	0.563459	0.0013	0.00454533	yes
Klhdc10	5.03473	5.01758	-0.0049216	0.975	0.986277	no
Klhdc2	20.3002	23.149	0.189459	0.225	0.367521	no

Klhdc3	29.2892	31.6628	0.112423	0.65795	0.785418	no
Klhdc4	25.6074	25.7678	0.00900723	0.9547	0.975769	no
Klhdc8b	0.837979	1.06491	0.345747	0.5763	0.720115	no
Klhl11	6.3263	6.49328	0.0375849	0.8364	0.906302	no
Klhl12	6.1358	6.83865	0.156461	0.3643	0.522559	no
Klhl15	2.35292	2.51109	0.0938592	0.69165	0.810231	no
Klhl17	11.2753	13.4681	0.256382	0.09785	0.189967	no
Klhl18	10.2467	10.1699	-0.010851	0.94425	0.969778	no
Klhl2	8.59375	9.46579	0.139435	0.38985	0.54901	no
Klhl20	15.678	15.2054	-0.0441602	0.76965	0.863675	no
Klhl21	10.7559	11.8134	0.135306	0.37425	0.532911	no
Klhl22	4.34435	5.33165	0.295442	0.12015	0.224457	no
Klhl24	9.05399	8.74186	-0.0506127	0.73845	0.842773	no
Klhl25	18.5066	17.8818	-0.0495472	0.75055	0.850913	no
Klhl26	4.18793	4.38833	0.0674348	0.71715	0.827725	no
Klhl28	7.62515	7.42674	-0.038037	0.82735	0.900881	no
Klhl3	1.32485	1.91893	0.534474	0.065	0.135282	no
Klhl36	9.97225	10.0824	0.0158501	0.92765	0.960543	no
Klhl42	3.38706	3.98982	0.236287	0.15745	0.277967	no
Klhl5	5.12813	6.75285	0.397065	0.02245	0.0551326	no
Klhl6	112.679	143.535	0.34918	0.01335	0.0351488	yes
Klhl7	3.87429	4.51904	0.222084	0.2269	0.369786	no
Klhl8	1.1399	1.21421	0.0911213	0.73975	0.843547	no
Klhl9	13.3463	12.6176	-0.0810099	0.59515	0.735853	no
Klk8	80.1699	93.7173	0.225254	0.11765	0.220704	no
Klra18	3.19025	1.20806	-1.40098	0.0208	0.0516212	no
Klra3	15.1863	4.70211	-1.69139	5.00E-05	0.000236281	yes
Klra4	2.47711	0.408911	-2.5988	0.02835	0.0673307	no
Klra7	1.72741	0.733294	-1.23615	0.13985	0.252821	no
Klra8	3.88846	1.05909	-1.87638	0.00655	0.0189418	yes
Klra9	3.51878	1.04577	-1.75051	0.01415	0.0369478	yes
Klrb1c	18.5648	12.2267	-0.602529	2.00E-04	0.0008488	yes
Klrb1f	4.26934	3.53008	-0.274315	0.2354	0.379737	no
Klrc1	311.846	236.621	-0.398253	0.0053	0.0157596	yes
Klrc2	21.3221	17.3186	-0.300025	0.1322	0.242362	no
Klrc3	4.43949	4.05788	-0.129667	0.69485	0.812442	no
Klrd1	613.561	590.895	-0.0543051	0.70065	0.81633	no
Klre1	25.9599	3.77743	-2.78081	5.00E-05	0.000236281	yes
Klrg1	204.405	54.5997	-1.90447	5.00E-05	0.000236281	yes
Klrk1	208.414	228.868	0.135064	0.35825	0.516125	no
Kmt2a	30.3904	33.7814	0.152612	0.28775	0.438338	no
Kmt2b	30.1627	32.8523	0.123228	0.39045	0.549526	no
Kmt2c	11.4984	11.8782	0.0468846	0.74235	0.845433	no
Kmt2d	26.8441	30.4047	0.179693	0.21125	0.350677	no
Kmt2e	44.1549	46.7248	0.0816162	0.62285	0.758031	no
Knop1	5.19635	5.11241	-0.0234961	0.8858	0.936091	no
Knstrn	12.3111	7.21931	-0.770023	5.00E-05	0.000236281	yes
Kntc1	3.60625	2.12107	-0.765706	5.00E-05	0.000236281	yes
Kpna1	17.9779	17.0486	-0.0765696	0.59865	0.738692	no
Kpna2	72.3462	46.2859	-0.644345	5.00E-05	0.000236281	yes
Kpna3	13.2277	11.1941	-0.240821	0.1116	0.211428	no
Kpna4	62.1615	58.7079	-0.0824666	0.5604	0.706741	no
Kpna6	13.1002	12.3136	-0.0893447	0.54125	0.690548	no
Kpnb1	59.764	60.0413	0.00667831	0.9629	0.980057	no
Kptn	17.4673	17.7244	0.0210852	0.8983	0.943664	no
Kras	21.219	18.8276	-0.172507	0.23375	0.377757	no
Krba1	3.07789	4.49154	0.545269	0.0011	0.00391273	yes

Krcc1	36.0505	37.2309	0.0464795	0.75485	0.853649	no
Kremen1	8.56633	9.61138	0.166065	0.28195	0.431833	no
Kremen2	5.19338	7.27649	0.486569	0.0129	0.0341475	yes
Kri1	44.5673	45.927	0.0433555	0.80975	0.889968	no
Krit1	17.0402	17.3886	0.0291941	0.84365	0.910734	no
Krr1	10.1768	10.6403	0.0642514	0.80775	0.888614	no
Krtcap2	302.036	262.667	-0.201485	0.16585	0.289911	no
Krtcap3	5.79169	6.5753	0.183071	0.91855	0.955437	no
Ksr1	10.7235	12.7956	0.254884	0.1015	0.195622	no
Kti12	27.8335	31.152	0.1625	0.3996	0.558606	no
Ktn1	8.85908	9.62471	0.119586	0.44005	0.598059	no
Kxd1	85.5719	75.2716	-0.185031	0.201	0.337352	no
L1cam	10.1397	7.68796	-0.399342	0.00955	0.0263592	yes
L3mbtl2	18.7154	19.2122	0.0377945	0.7972	0.881985	no
L3mbtl3	13.9515	13.7412	-0.0219179	0.88585	0.93613	no
l7Rn6	36.2447	35.4535	-0.0318434	0.84435	0.91128	no
Lace1	3.10488	2.65568	-0.225458	0.3171	0.47123	no
Lactb	14.5668	11.9	-0.291717	0.08075	0.161826	no
Lactb2	16.7618	14.6386	-0.195397	0.22885	0.372002	no
Lag3	26.1781	27.9025	0.0920315	0.54975	0.697811	no
Lage3	32.7882	33.4826	0.0302372	0.8623	0.922218	no
Lair1	13.7559	12.1581	-0.178134	0.24455	0.389617	no
Lamb3	1.76078	1.66562	-0.0801584	0.71805	0.828439	no
Lamc1	12.8611	8.98734	-0.517052	0.00025	0.00103712	yes
Lamp1	93.1886	95.9348	0.041901	0.76665	0.861583	no
Lamp2	14.6775	15.8946	0.114932	0.4926	0.647329	no
Lamtor1	71.4623	73.0462	0.0316275	0.8311	0.903098	no
Lamtor2	115.67	114.597	-0.0134424	0.9278	0.960628	no
Lamtor3	38.3636	32.0408	-0.259831	0.09505	0.185393	no
Lamtor4	73.3311	65.288	-0.167609	0.2726	0.420846	no
Lamtor5	93.2422	83.6071	-0.157357	0.2892	0.439977	no
Lancl1	6.90198	8.58519	0.314839	0.0448	0.0991463	no
Lancl2	2.17649	2.29367	0.075655	0.72175	0.831037	no
Lancl3	4.39273	5.92609	0.431962	0.01295	0.0342503	yes
Lap3	7.69526	9.35812	0.282248	0.11065	0.210056	no
Laptm4a	32.3739	27.9154	-0.213771	0.1526	0.271025	no
Laptm5	1272.77	1150.94	-0.145153	0.4081	0.566985	no
Larp1	34.8843	36.0973	0.049314	0.725	0.833216	no
Larp1b	9.53616	10.0508	0.0758261	0.6793	0.801283	no
Larp4	10.5761	12.1073	0.195061	0.18415	0.315026	no
Larp4b	18.4536	18.0709	-0.030236	0.83495	0.905469	no
Larp7	39.7417	35.046	-0.181404	0.2155	0.355883	no
Lars	18.9132	19.9616	0.0778316	0.59495	0.735708	no
Lars2	839.88	201.754	-2.05758	5.00E-05	0.000236281	yes
Las1l	19.3607	21.3172	0.138891	0.35495	0.512574	no
Lasp1	155.002	136.324	-0.185251	0.20015	0.336253	no
Lat	601.718	589.657	-0.0292119	0.8398	0.908203	no
Lat2	5.77193	10.3309	0.839842	5.00E-05	0.000236281	yes
Lats1	7.70467	8.1863	0.0874788	0.56205	0.708062	no
Lats2	23.2638	19.222	-0.275328	0.1217	0.226704	no
Lax1	125.615	125.755	0.00160837	0.9899	0.993885	no
Lbh	67.7498	69.2957	0.0325503	0.8193	0.895847	no
Lbr	108.381	109.928	0.0204539	0.88095	0.933257	no
Lca5	1.28134	2.22315	0.794949	0.00055	0.0021091	yes
Lck	886.166	810.212	-0.129277	0.4154	0.574143	no
Lclat1	1.7877	2.84941	0.672559	8.00E-04	0.0029486	yes
Lcmt1	25.1345	20.363	-0.303717	0.06415	0.133829	no

Lcmt2	4.77137	5.30704	0.153503	0.47455	0.630963	no
Lcn4	17.6799	22.2576	0.332191	0.0846	0.16837	no
Lcor	1.71889	2.21609	0.366536	0.07785	0.15703	no
Lcorl	3.44748	2.78348	-0.308652	0.3329	0.48865	no
Lcp1	603.755	592.55	-0.0270263	0.87035	0.927079	no
Lcp2	133.221	134.601	0.0148762	0.91485	0.953387	no
Ldb1	138.898	131.356	-0.080548	0.56145	0.707584	no
Ldha	348.662	336.109	-0.0529002	0.7073	0.82111	no
Ldlr	3.2583	3.25568	-0.00116138	0.994	0.996202	no
Ldlrad4	2.744	2.90052	0.0800345	0.72075	0.830387	no
Ldlrap1	17.2383	19.8502	0.203539	0.18055	0.310078	no
Ldoc1l	1.40555	2.05006	0.544531	0.0147	0.0381777	yes
Lef1	34.83	40.9625	0.233973	0.11335	0.214042	no
Lemd2	26.209	24.9023	-0.0737832	0.61915	0.755221	no
Lemd3	9.97653	10.9692	0.136846	0.36885	0.527403	no
Lenep	10.8515	8.49015	-0.354038	0.1624	0.284981	no
Leng1	12.7258	12.7825	0.0064117	0.98515	0.991429	no
Leng8	52.6075	49.6319	-0.0840006	0.5563	0.703241	no
Leng9	5.08903	4.85758	-0.0671541	0.75625	0.854548	no
Leo1	11.2574	11.3159	0.00747228	0.96475	0.9808	no
Lepre1	1.67245	1.38278	-0.274397	0.2718	0.419896	no
Leprel4	2.99034	0.968667	-1.62624	5.00E-05	0.000236281	yes
Leprot	38.8647	39.2667	0.0148442	0.9191	0.955826	no
Leprotl1	94.7796	89.3565	-0.0850044	0.545	0.693702	no
Letm1	30.2994	29.1055	-0.0579969	0.686	0.806087	no
Letm2	4.55316	5.30136	0.219495	0.27875	0.428224	no
Letmd1	22.3961	20.7293	-0.111577	0.4568	0.61433	no
Lfng	230.455	206.878	-0.155705	0.27395	0.422477	no
Lgals1	2005.35	1544	-0.377183	0.0127	0.0336825	yes
Lgals3	133.488	124.205	-0.103986	0.4686	0.625278	no
Lgals3bp	78.2139	87.5558	0.162779	0.2555	0.400375	no
Lgals4	14.8929	15.5807	0.0651401	0.7255	0.8336	no
Lgals6	0.805339	1.21311	0.591043	0.7497	0.850401	no
Lgals8	31.079	29.9457	-0.0535885	0.71635	0.827357	no
Lgals9	122.394	117.823	-0.0549042	0.70165	0.816974	no
Lgalsl	4.87138	4.83655	-0.010352	0.95325	0.974964	no
Lhpp	0.829109	1.25184	0.59442	0.1266	0.234023	no
Lias	18.941	18.5849	-0.0273763	0.8661	0.924505	no
Lig1	24.4955	14.7092	-0.735798	5.00E-05	0.000236281	yes
Lig3	6.48926	7.44539	0.198294	0.2684	0.415936	no
Lig4	2.11675	2.19377	0.0515669	0.80835	0.889011	no
Lilrb4	10.717	11.0702	0.0467785	0.80485	0.886917	no
Limd1	42.7381	42.8367	0.00332603	0.98105	0.989178	no
Limd2	220.518	240.552	0.125449	0.3836	0.542666	no
Lime1	192.711	183.636	-0.069586	0.6263	0.76076	no
Limk1	16.2802	16.9681	0.0597052	0.69105	0.809743	no
Limk2	26.5125	31.8728	0.265656	0.06485	0.135026	no
Lims1	27.1492	23.8529	-0.186745	0.19395	0.328101	no
Lin37	37.3076	37.0047	-0.0117598	0.9413	0.968242	no
Lin52	6.7013	6.84605	0.0308311	0.86365	0.923007	no
Lin54	3.89836	3.24591	-0.264243	0.1364	0.248314	no
Lin7c	25.2251	25.9373	0.0401688	0.7796	0.870399	no
Lin9	6.14977	5.4704	-0.168886	0.375	0.533699	no
Lins	2.25167	2.86073	0.345385	0.06705	0.138698	no
Lipa	22.9174	20.5908	-0.154446	0.29915	0.451292	no
Lipe	1.82613	2.09199	0.196087	0.4213	0.579687	no
Lipo1	1.30425	1.22929	-0.0853918	0.7552	0.853895	no

Lipt1	1.11368	1.95242	0.809924	0.02225	0.0547157	no
Litaf	7.06679	4.56188	-0.631429	0.0015	0.00516454	yes
Llgl1	16.5736	15.5626	-0.0908028	0.781	0.871204	no
Llgl2	11.9335	12.5917	0.0774509	0.61935	0.755399	no
Llph	64.2522	66.6007	0.051791	0.71625	0.827322	no
Lman1	10.8826	10.253	-0.085975	0.6273	0.761556	no
Lman2	50.2446	59.8914	0.25338	0.0727	0.148354	no
Lman2l	6.41685	6.00144	-0.0965549	0.60835	0.746264	no
Lmbr1l	43.6187	45.2934	0.0543534	0.7081	0.821714	no
Lmbrd1	6.4448	5.20866	-0.307223	0.0564	0.120485	no
Lmbrd2	2.52161	2.37992	-0.0834303	0.74675	0.848431	no
Lmf1	10.6119	10.9728	0.0482477	0.78315	0.872788	no
Lmf2	28.6463	26.3588	-0.12006	0.4073	0.566107	no
Lmna	2.22412	2.92368	0.394552	0.11875	0.222359	no
Lmnb1	118.345	79.9761	-0.565363	2.00E-04	0.0008488	yes
Lmnb2	6.78816	6.87026	0.0173447	0.9191	0.955826	no
Lmo4	9.92143	9.81341	-0.0157929	0.93265	0.963275	no
Lmtk2	8.57186	9.20445	0.102724	0.48195	0.637536	no
Lmtk3	1.39497	1.8847	0.434099	0.04315	0.0960307	no
Lnp	3.36505	2.99993	-0.165701	0.30835	0.461609	no
Lnpep	50.2496	51.2076	0.0272447	0.84295	0.910312	no
Lnx1	1.98351	1.51001	-0.3935	0.68525	0.805608	no
Lnx2	12.4387	12.35	-0.0103279	0.94245	0.968776	no
LOC547349	2.0752	1.68135	-0.303633	0.86945	0.926554	no
Loh12cr1	13.2702	11.4419	-0.21387	0.21445	0.354638	no
Lonp1	19.0514	20.1232	0.0789642	0.598	0.738122	no
Lonp2	49.1546	47.61	-0.0460622	0.74965	0.850399	no
Lpar2	2.23338	2.53699	0.183888	0.32245	0.477382	no
Lpar5	12.6481	14.6328	0.210291	0.23805	0.382839	no
Lpar6	25.5149	26.3576	0.0468813	0.83725	0.906751	no
Lpcat1	8.31172	7.46867	-0.154296	0.4206	0.579096	no
Lpcat3	12.8807	13.1414	0.0289144	0.9314	0.962533	no
Lpcat4	51.5546	62.5	0.277756	0.05255	0.113396	no
Lpgat1	6.77157	7.36489	0.121176	0.42545	0.583838	no
Lphn1	11.1471	11.2144	0.00869273	0.9505	0.973421	no
Lpin1	24.0644	17.1235	-0.49092	0.00045	0.0017621	yes
Lpin2	10.6134	12.4172	0.226451	0.1234	0.2292	no
Lpp	2.25632	2.21616	-0.0259067	0.8789	0.932261	no
Lpxn	81.778	82.1849	0.00715982	0.9581	0.97746	no
Lrba	12.1002	12.3062	0.0243496	0.86695	0.925021	no
Lrch1	14.0563	13.7232	-0.0345999	0.81235	0.891521	no
Lrch3	17.9978	18.7157	0.0564344	0.73385	0.839409	no
Lrch4	87.7362	80.7767	-0.119233	0.523	0.674765	no
Lrif1	18.5412	17.9411	-0.0474668	0.78215	0.872073	no
Lrig2	5.7096	5.83582	0.0315451	0.83615	0.906116	no
Lrmp	94.2263	81.6043	-0.207486	0.13955	0.252356	no
Lrp10	118.11	120.063	0.0236591	0.86765	0.925544	no
Lrp5	1.187	1.84537	0.636594	0.0037	0.0114853	yes
Lrp6	3.25672	3.53193	0.117037	0.4586	0.616081	no
Lrp8	2.12471	1.38971	-0.612483	0.00175	0.00592472	yes
Lrpap1	17.3171	17.9532	0.0520402	0.7344	0.839821	no
Lrpprc	12.4688	13.6202	0.127425	0.392	0.551114	no
Lrr1	2.74789	1.45642	-0.915897	0.0053	0.0157596	yes
Lrrc1	2.49514	2.85572	0.194736	0.3585	0.516359	no
Lrrc14	7.18512	7.7563	0.110356	0.5383	0.688066	no
Lrrc20	2.85249	2.95206	0.0495013	0.82425	0.898947	no
Lrrc23	2.08864	3.72597	0.835051	0.0038	0.0117554	yes

Lrrc28	6.07539	5.56928	-0.125485	0.49785	0.6522	no
Lrrc29	1.29552	0.899984	-0.525563	0.13285	0.243238	no
Lrrc39	1.13455	1.19765	0.0780896	0.89025	0.938598	no
Lrrc40	5.04599	4.53813	-0.153042	0.4161	0.574784	no
Lrrc41	9.42267	10.4421	0.148209	0.46385	0.6209	no
Lrrc42	13.1529	12.858	-0.0327244	0.84535	0.91169	no
Lrrc45	13.0254	12.5134	-0.0578504	0.7173	0.827817	no
Lrrc47	27.0766	27.5483	0.0249166	0.87905	0.932336	no
Lrrc51	1.38115	1.37558	-0.00583507	0.98915	0.993584	no
Lrrc56	2.08827	2.51437	0.26789	0.3517	0.509047	no
Lrrc57	17.0052	14.6318	-0.216868	0.3161	0.47011	no
Lrrc58	4.13861	4.63003	0.161875	0.2963	0.447988	no
Lrrc59	32.3486	31.7964	-0.0248386	0.8679	0.925713	no
Lrrc61	16.4289	15.8215	-0.0543486	0.7247	0.83302	no
Lrrc8a	21.3674	21.7545	0.0259033	0.8581	0.919819	no
Lrrc8b	4.1632	3.50637	-0.247716	0.20995	0.348979	no
Lrrc8c	67.0104	60.5151	-0.147089	0.29765	0.449577	no
Lrrc8d	5.7852	8.47649	0.5511	0.001	0.00359708	yes
Lrrcc1	7.64547	7.66196	0.00310815	0.98405	0.990703	no
Lrrfip1	135.659	139.497	0.040252	0.78065	0.871034	no
Lrrfip2	8.62608	6.37353	-0.436611	0.0085	0.0237772	yes
Lrrk1	13.9825	7.82052	-0.838289	5.00E-05	0.000236281	yes
Lrsam1	3.40538	3.00446	-0.180713	0.54945	0.697593	no
Lrwd1	46.8078	49.3607	0.0766151	0.59125	0.732493	no
Lsg1	24.1894	25.2692	0.063005	0.67455	0.797572	no
Lsm1	14.8853	13.6628	-0.123637	0.44115	0.59907	no
Lsm10	24.9354	23.9401	-0.0587683	0.73645	0.841374	no
Lsm11	2.41151	2.21457	-0.12291	0.50725	0.660896	no
Lsm12	35.2506	32.6769	-0.10938	0.48305	0.638632	no
Lsm14a	96.5244	90.3499	-0.0953707	0.49665	0.651172	no
Lsm14b	21.6241	22.1941	0.0375339	0.8026	0.885364	no
Lsm2	29.6776	27.4552	-0.112296	0.5174	0.669941	no
Lsm3	68.6241	73.8721	0.106315	0.5072	0.660867	no
Lsm4	139.249	134.88	-0.0459911	0.74995	0.850506	no
Lsm5	114.293	98.2957	-0.21754	0.1767	0.304726	no
Lsm6	15.1132	13.1298	-0.202969	0.1772	0.305439	no
Lsm7	82.7816	96.0887	0.215057	0.1944	0.328752	no
Lsm8	69.9238	66.5522	-0.0712962	0.6478	0.777589	no
Lsp1	1119.48	1182.18	0.0786261	0.62365	0.758764	no
Lsr	2.06363	2.42301	0.231615	0.36465	0.522923	no
Lss	6.12999	7.66661	0.322705	0.0594	0.125854	no
Lst1	6.34815	4.81323	-0.39933	0.3214	0.476267	no
Lta	4.48738	8.86519	0.982278	5.00E-05	0.000236281	yes
Lta4h	80.4667	82.2294	0.0312629	0.82755	0.90096	no
Ltb	402.695	571.682	0.505525	0.00035	0.0014053	yes
Ltb4r1	3.33473	1.76736	-0.915972	0.00305	0.00969839	yes
Ltk	3.67068	4.24545	0.209868	0.31655	0.470651	no
Ltn1	16.9962	16.6151	-0.0327215	0.81395	0.89232	no
Ltv1	21.646	24.3962	0.172558	0.27365	0.422135	no
Luc7l	35.5394	37.6859	0.0846065	0.60095	0.740559	no
Luc7l2	78.3963	76.6913	-0.0317239	0.8305	0.90271	no
Luc7l3	29.9993	27.4489	-0.12818	0.3729	0.531546	no
Luzp1	1.26304	0.961541	-0.393478	0.0629	0.131837	no
Lxn	22.7763	22.6624	-0.00723008	0.9778	0.987609	no
Ly6a	269.323	316.047	0.230801	0.10065	0.194297	no
Ly6c1	12.6205	8.01624	-0.654772	0.01075	0.0292077	yes
Ly6c2	1934.05	1506.13	-0.36078	0.01815	0.0458046	yes

Ly6e	352.058	468.893	0.413445	0.00445	0.0135244	yes
Ly6g5b	2.69784	5.12572	0.925952	0.0028	0.00898766	yes
Ly75	8.95015	12.7422	0.509635	7.00E-04	0.00261609	yes
Ly9	146.876	132.985	-0.143336	0.30885	0.462172	no
Ly96	5.16572	5.3983	0.0635354	0.8453	0.911678	no
Lyar	24.9534	22.1347	-0.172929	0.2755	0.424367	no
Lypd6b	1.11061	2.12802	0.938165	0.0017	0.005774	yes
Lypla1	58.9726	53.9117	-0.129447	0.36325	0.521455	no
Lypla2	133.713	134.298	0.0062954	0.96295	0.980057	no
Lyrm1	6.92989	7.94059	0.196414	0.39565	0.554743	no
Lyrm2	23.768	23.2373	-0.0325752	0.84365	0.910734	no
Lyrm4	5.56704	5.02652	-0.147348	0.5041	0.658153	no
Lyrm5	10.5632	11.1175	0.0737947	0.69085	0.809616	no
Lyrm7	1.40367	1.32726	-0.0807484	0.7932	0.879351	no
Lyrm9	3.36387	3.15477	-0.0925876	0.6077	0.745817	no
Lysmd1	9.22335	9.7496	0.0800524	0.7981	0.882482	no
Lysmd2	9.39527	13.0935	0.478845	0.01415	0.0369478	yes
Lysmd3	12.4607	12.4911	0.00351731	0.9799	0.988598	no
Lysmd4	3.05967	2.35023	-0.380573	0.0723	0.147727	no
Lyst	11.9548	15.3052	0.356437	0.01205	0.0321932	yes
Lzic	19.409	19.1735	-0.0176177	0.91335	0.952555	no
Lztfl1	3.37365	3.93233	0.221072	0.24975	0.395297	no
Lztr1	26.9541	24.8576	-0.116815	0.4243	0.582645	no
M6pr	133.512	124.341	-0.10267	0.46495	0.62196	no
Macf1	115.321	119.534	0.0517664	0.77205	0.865283	no
Macrod1	1.09315	1.29648	0.246109	0.54075	0.690147	no
Mad1l1	10.2504	8.78625	-0.222356	0.1877	0.319913	no
Mad2l1	31.1607	20.997	-0.569541	0.00035	0.0014053	yes
Mad2l1bp	34.3378	29.468	-0.220647	0.1579	0.278622	no
Mad2l2	11.7561	11.4541	-0.0375539	0.84625	0.912062	no
Madd	59.088	65.5757	0.150294	0.28615	0.436598	no
Maea	61.9464	56.8621	-0.123552	0.3832	0.542264	no
Maf	1.96706	2.57177	0.38672	0.0761	0.154033	no
Maf1	90.6153	83.8108	-0.112619	0.42925	0.587533	no
Mafa	1.08733	0.382736	-1.50637	0.02295	0.0562099	no
Mafg	3.04045	3.42524	0.171918	0.48735	0.642511	no
Mafk	21.792	25.3967	0.220842	0.1383	0.250474	no
Maged2	1.07639	1.00711	-0.0959864	0.77075	0.864428	no
Magee1	2.30222	1.91404	-0.266402	0.2329	0.376806	no
Magi3	1.07451	0.813159	-0.402074	0.0832	0.166033	no
Magoh	126.915	119.004	-0.0928466	0.52785	0.679123	no
Magohb	24.1381	23.0002	-0.0696663	0.7107	0.823553	no
Magt1	19.2745	16.2402	-0.247128	0.0888	0.175271	no
Mak16	41.9132	43.4565	0.052167	0.7472	0.848683	no
Mal	0.38029	1.32995	1.8062	1.00E-04	0.000450026	yes
Malsu1	17.7534	16.8028	-0.0793887	0.68585	0.805965	no
Malt1	56.2412	52.8969	-0.0884467	0.52855	0.679751	no
Maml1	13.886	13.9859	0.0103414	0.94385	0.969593	no
Maml2	12.5371	12.3799	-0.0182054	0.9007	0.945017	no
Man1a	30.7336	28.5292	-0.107377	0.44515	0.602843	no
Man1a2	10.3789	10.3322	-0.00649695	0.96365	0.980388	no
Man1b1	26.0506	25.2334	-0.0459824	0.74755	0.848903	no
Man1c1	0.748525	1.88954	1.33591	5.00E-05	0.000236281	yes
Man2a1	30.614	34.1372	0.157152	0.2627	0.409024	no
Man2a2	12.239	15.5026	0.341026	0.01905	0.0477889	yes
Man2b1	60.2853	56.3571	-0.0972068	0.48895	0.643971	no
Man2b2	33.2129	34.0203	0.0346509	0.8061	0.887546	no

Man2c1	14.4003	17.0791	0.246133	0.10635	0.20326	no
Manba	15.226	15.3699	0.013574	0.92785	0.960632	no
Manbal	29.7743	28.4397	-0.0661644	0.68635	0.806378	no
Manea	4.40004	4.05728	-0.117004	0.50455	0.658618	no
Maneal	1.17356	1.06405	-0.141325	0.8093	0.889668	no
Manf	48.7209	48.2583	-0.0137658	0.92345	0.958246	no
Mansc1	1.22349	1.36548	0.158406	0.5849	0.727358	no
Map1lc3a	5.84483	5.60199	-0.0612234	0.7962	0.88132	no
Map1lc3b	139.643	132.846	-0.0719825	0.6016	0.741153	no
Map1s	15.3364	15.3685	0.00301387	0.98335	0.990366	no
Map2k1	44.2389	43.0438	-0.0395104	0.78485	0.874089	no
Map2k2	56.0839	54.2727	-0.047358	0.7407	0.844235	no
Map2k3	35.7456	35.1723	-0.023325	0.87665	0.930896	no
Map2k4	23.1829	21.969	-0.0775917	0.5932	0.734278	no
Map2k5	15.7483	17.5642	0.157446	0.3127	0.4664	no
Map2k6	9.34575	7.2308	-0.370156	0.04135	0.0926	no
Map2k7	31.0793	30.9881	-0.00423942	0.9779	0.987653	no
Map3k1	14.6799	14.4833	-0.0194431	0.89065	0.938743	no
Map3k10	2.4038	2.52548	0.0712404	0.7273	0.834785	no
Map3k11	23.6617	24.1499	0.0294638	0.83525	0.905627	no
Map3k12	12.6294	11.3935	-0.148566	0.6954	0.812803	no
Map3k14	13.1926	16.9905	0.364998	0.0147	0.0381777	yes
Map3k15	1.29726	1.15861	-0.16307	0.83365	0.904573	no
Map3k2	9.84954	9.73125	-0.0174314	0.902	0.945889	no
Map3k3	28.596	32.0654	0.165203	0.24995	0.395436	no
Map3k4	9.84872	10.6678	0.11526	0.44265	0.600494	no
Map3k5	7.15253	9.79117	0.453027	0.00265	0.0085599	yes
Map3k7	22.0144	20.6947	-0.0891852	0.5355	0.685865	no
Map3k8	19.5628	18.7671	-0.0599076	0.6964	0.813581	no
Map4	46.6388	43.8224	-0.0898622	0.52285	0.674633	no
Map4k1	66.5659	62.4359	-0.0924082	0.5074	0.661043	no
Map4k2	108.625	108.352	-0.00363708	0.9816	0.989458	no
Map4k4	62.8823	65.9425	0.0685554	0.62665	0.760976	no
Map4k5	1.21764	1.13395	-0.102729	0.8208	0.896737	no
Map7	1.49916	1.44522	-0.0528638	0.82185	0.897384	no
Map7d1	53.6775	46.1113	-0.219197	0.12225	0.227531	no
Mapk1	95.6478	90.3955	-0.0814806	0.6125	0.749804	no
Mapk11	5.4152	10.0096	0.886301	5.00E-05	0.000236281	yes
Mapk14	51.2001	49.1078	-0.0601958	0.66965	0.79412	no
Mapk1ip1	10.5256	11.1035	0.077105	0.67015	0.79458	no
Mapk1ip1l	29.7443	27.487	-0.113865	0.4214	0.579802	no
Mapk3	40.0818	25.2425	-0.667092	5.00E-05	0.000236281	yes
Mapk6	25.6704	25.7387	0.00383	0.97855	0.987999	no
Mapk7	11.284	9.98336	-0.176681	0.2751	0.423899	no
Mapk8	3.63635	3.8469	0.0812075	0.6365	0.768811	no
Mapk8ip3	33.6912	34.444	0.0318775	0.8278	0.901065	no
Mapk9	12.4701	11.5177	-0.114627	0.44385	0.601637	no
Mapkap1	18.2318	18.9951	0.0591636	0.6951	0.812573	no
Mapkapk2	25.5758	24.4942	-0.0623408	0.6778	0.800142	no
Mapkapk3	35.3119	34.3905	-0.0381471	0.78665	0.875141	no
Mapkapk5	27.9684	28.0651	0.00498262	0.9736	0.985538	no
Mapkbp1	4.11061	4.35836	0.084433	0.59195	0.73318	no
Mapre1	37.4869	33.6053	-0.157699	0.26455	0.411324	no
Mapre2	51.8936	43.0925	-0.268121	0.05985	0.12667	no
Mapre3	2.16715	2.28686	0.0775705	0.7726	0.865678	no
2-Mar	33.178	34.6375	0.0621083	0.6707	0.794951	no
2-Mar	69.5209	68.6543	-0.0180948	0.89595	0.942106	no

3-Mar	2.98123	3.53733	0.246752	0.31285	0.466535	no
5-Mar	39.517	36.1884	-0.126946	0.4604	0.617641	no
6-Mar	19.7865	20.5391	0.0538542	0.70795	0.821648	no
7-Mar	50.5139	53.2861	0.0770795	0.5847	0.727161	no
9-Mar	1.81112	1.44919	-0.321634	0.2682	0.415681	no
Marcksl1	6.9489	5.47093	-0.344998	0.0987	0.191302	no
Marf1	23.2537	22.9352	-0.0198966	0.88825	0.937305	no
Mark2	63.1875	64.105	0.0207987	0.88295	0.934535	no
Mark3	33.8744	31.8587	-0.088508	0.53855	0.688248	no
Mark4	14.873	15.58	0.0669951	0.67545	0.798329	no
Mars	24.9977	24.3366	-0.0386669	0.81095	0.890677	no
Mars2	6.18763	6.68308	0.111128	0.5284	0.679633	no
Marveld1	12.6653	10.5569	-0.262695	0.0977	0.189739	no
Marveld2	2.62187	4.11362	0.649811	0.0022	0.00725609	yes
Mast2	5.13421	3.88854	-0.400915	0.016	0.0410584	yes
Mast3	50.5427	51.2902	0.021183	0.8847	0.935461	no
Mast4	0.680239	1.09822	0.691058	0.00115	0.00407292	yes
Mastl	2.59966	1.39264	-0.900499	5.00E-05	0.000236281	yes
Mat2a	40.9759	41.2656	0.010164	0.94425	0.969778	no
Mat2b	95.5828	85.7192	-0.157132	0.27035	0.41826	no
Matk	9.18328	9.23017	0.00734672	0.96685	0.981973	no
Matr3	51.4468	50.7411	-0.0199273	0.88705	0.936711	no
Mau2	30.0236	30.1105	0.00417069	0.97635	0.986921	no
Mavs	13.6041	13.3414	-0.0281353	0.85405	0.917261	no
Max	48.743	49.0858	0.0101108	0.9441	0.969778	no
Maz	123.16	123.537	0.00441594	0.97455	0.986118	no
Mb21d1	4.37848	3.31388	-0.401908	0.0273	0.0652033	no
Mbd1	21.7761	22.0951	0.0209768	0.88825	0.937305	no
Mbd2	188.417	176.816	-0.091679	0.5205	0.672573	no
Mbd3	58.0683	54.693	-0.0863933	0.54695	0.695407	no
Mbd4	2.98416	2.22722	-0.422078	0.04415	0.0979227	no
Mbd5	1.32395	1.36749	0.0466767	0.8244	0.898984	no
Mbd6	22.4795	25.7772	0.197485	0.37275	0.531407	no
Mbip	15.1466	16.4787	0.121615	0.4773	0.633367	no
Mblac2	2.03032	2.10621	0.0529402	0.8052	0.887012	no
Mbnl1	260.335	283.078	0.120827	0.42735	0.585705	no
Mbnl2	9.15942	7.46391	-0.295326	0.05805	0.123397	no
Mbnl3	4.54054	3.59717	-0.336001	0.03195	0.0744133	no
Mboat1	6.18956	6.57073	0.0862164	0.61985	0.755787	no
Mboat4	1.221	1.54729	0.341684	0.2828	0.432769	no
Mboat7	10.933	11.7156	0.0997363	0.5294	0.680547	no
Mbp	55.0508	57.1889	0.0549716	0.7018	0.817009	no
Mbtd1	9.75248	11.8569	0.281891	0.06245	0.131065	no
Mbtps1	29.9313	33.4738	0.161378	0.24885	0.394243	no
Mbtps2	4.43946	4.70597	0.084108	0.6773	0.799725	no
Mcam	1.20524	0.57791	-1.06041	0.0031	0.00983478	yes
Mcat	9.78287	9.74613	-0.00542758	0.9728	0.985025	no
Mccc1	5.01804	5.91928	0.238296	0.1925	0.326196	no
Mccc2	1.27381	1.90811	0.582999	0.045	0.0995421	no
Mcee	12.945	11.8934	-0.122235	0.5935	0.734507	no
Mcfd2	13.3092	14.8043	0.153597	0.3503	0.507677	no
Mcl1	151.914	147.331	-0.0441905	0.75765	0.855527	no
Mcm10	5.71382	3.06695	-0.897654	5.00E-05	0.000236281	yes
Mcm2	30.5654	23.0281	-0.408507	0.0055	0.0162672	yes
Mcm3	44.4445	33.4536	-0.409842	0.00405	0.012441	yes
Mcm3ap	19.4847	18.8334	-0.0490435	0.7343	0.839734	no
Mcm4	45.9416	37.3434	-0.298949	0.03505	0.0804834	no

Mcm5	46.369	30.3722	-0.610407	5.00E-05	0.000236281	yes
Mcm6	92.4198	69.3995	-0.413277	0.0033	0.0103917	yes
Mcm7	51.8399	36.7401	-0.496707	0.00045	0.0017621	yes
Mcm8	1.80164	1.39291	-0.371208	0.12385	0.229896	no
Mcm9	4.59476	4.87661	0.0858891	0.70255	0.817599	no
Mcmbp	27.4661	27.2736	-0.0101515	0.94315	0.969199	no
Mcoln1	14.8271	13.9847	-0.0843865	0.609	0.746776	no
Mcoln2	7.09052	13.2062	0.897249	5.00E-05	0.000236281	yes
Mcph1	4.02946	3.95663	-0.026314	0.8811	0.933374	no
Mcrs1	41.7211	37.8798	-0.13935	0.3445	0.501371	no
Mctp2	17.2835	15.2937	-0.176456	0.22425	0.366593	no
Mcts1	62.7366	56.7683	-0.14422	0.3437	0.500516	no
Mcts2	7.25408	8.27702	0.190321	0.85065	0.914834	no
Mcu	10.0315	10.0601	0.00411354	0.98185	0.989603	no
Mcur1	9.66969	10.8945	0.17206	0.2631	0.409566	no
Mdc1	12.5483	13.2241	0.0756722	0.5983	0.738389	no
Mdfic	7.72793	6.86148	-0.171562	0.30005	0.452379	no
Mdh1	96.3771	91.7873	-0.0703955	0.6182	0.754531	no
Mdh2	205.849	195.824	-0.072029	0.611	0.748487	no
Mdk	5.07329	4.34321	-0.224162	0.521	0.673046	no
Mdm1	8.2262	4.93297	-0.737769	5.00E-05	0.000236281	yes
Mdm2	44.3455	42.7577	-0.0526023	0.7139	0.825689	no
Mdm4	53.7319	55.2372	0.0398628	0.77635	0.868226	no
Mdn1	6.48914	9.4652	0.544606	0.00025	0.00103712	yes
Mdp1	61.6013	60.7444	-0.0202086	0.89	0.93838	no
Me2	77.0257	67.7817	-0.184445	0.18915	0.321887	no
Mea1	67.2252	63.5086	-0.0820516	0.77615	0.86814	no
Meaf6	23.9301	21.995	-0.121652	0.47015	0.626742	no
Mecp2	7.60126	8.09795	0.0913168	0.535	0.685399	no
Mecr	8.88911	10.6194	0.256587	0.18425	0.315136	no
Med1	22.4237	22.0254	-0.0258576	0.86915	0.92636	no
Med10	90.9725	86.1617	-0.0783829	0.5937	0.734665	no
Med11	46.49	48.2162	0.0525979	0.73785	0.842374	no
Med12	39.2742	39.2607	-0.000495921	0.99775	0.998245	no
Med12l	3.17046	1.87755	-0.755839	0.0274	0.065409	no
Med13	19.6065	19.3357	-0.0200677	0.8864	0.936386	no
Med13l	11.6545	12.9045	0.146987	0.3081	0.461362	no
Med14	15.6225	15.4775	-0.0134538	0.92625	0.959764	no
Med15	38.798	36.0733	-0.105052	0.45735	0.614788	no
Med16	13.6735	13.1848	-0.0525031	0.7348	0.840115	no
Med17	15.0854	16.0726	0.0914566	0.5377	0.68766	no
Med18	2.17033	2.18931	0.0125626	0.9666	0.981874	no
Med19	8.15086	7.463	-0.127196	0.7867	0.875183	no
Med20	18.7605	16.1129	-0.219484	0.1676	0.292362	no
Med21	35.8083	33.4293	-0.099178	0.5656	0.711151	no
Med22	14.392	15.721	0.127422	0.4243	0.582645	no
Med23	8.53678	8.60993	0.012309	0.9346	0.964438	no
Med24	21.9781	21.3693	-0.0405286	0.7774	0.868973	no
Med25	26.5507	29.3538	0.144798	0.3299	0.485559	no
Med26	10.0352	9.753	-0.0411482	0.7999	0.883614	no
Med27	21.0777	20.7715	-0.0211099	0.9001	0.944711	no
Med28	15.5109	13.7143	-0.1776	0.22885	0.372002	no
Med29	6.71096	7.0617	0.0734968	0.6805	0.802095	no
Med30	78.994	66.5763	-0.246734	0.1027	0.197568	no
Med31	4.64814	3.85766	-0.26893	0.299	0.451104	no
Med4	28.5446	25.0003	-0.191273	0.23675	0.381348	no
Med6	20.8154	18.7701	-0.149218	0.4045	0.563425	no

Med7	11.1724	9.8932	-0.175436	0.2877	0.438272	no
Med8	33.6282	30.6702	-0.132832	0.37925	0.538249	no
Med9	5.72968	5.84631	0.0290716	0.8757	0.930307	no
Mef2a	26.1585	23.1469	-0.17646	0.2173	0.358151	no
Mef2d	50.0559	55.8793	0.158773	0.2652	0.412071	no
Megf9	2.36052	1.83653	-0.36212	0.12225	0.227531	no
Meis3	20.9229	20.7766	-0.0101278	0.95155	0.974058	no
Melk	5.50396	3.06766	-0.843333	5.00E-05	0.000236281	yes
Memo1	31.0915	28.5926	-0.120878	0.4273	0.585659	no
Men1	23.1861	21.8289	-0.0870224	0.5594	0.705971	no
Mepce	21.2189	21.5518	0.0224581	0.88055	0.933044	no
Mesdc1	7.90937	7.41572	-0.0929763	0.5735	0.717722	no
Mesdc2	6.67007	7.85181	0.235324	0.1381	0.250474	no
Metap1	16.7553	17.6726	0.0768941	0.61315	0.75034	no
Metap1d	9.65347	11.5025	0.252823	0.1923	0.32592	no
Metap2	29.886	28.8083	-0.0529835	0.71055	0.823433	no
Metrn	18.6382	14.2902	-0.383232	0.0313	0.0731359	no
Mettl1	3.55474	5.56807	0.647433	0.0271	0.0647848	no
Mettl10	23.5807	22.2328	-0.0849193	0.61815	0.754496	no
Mettl13	3.78442	4.55906	0.268665	0.16535	0.289202	no
Mettl14	12.928	12.976	0.00535584	0.9723	0.984802	no
Mettl15	3.01939	2.9345	-0.041142	0.8683	0.925972	no
Mettl16	11.107	12.1999	0.135399	0.40075	0.559737	no
Mettl17	22.394	23.3523	0.0604539	0.70565	0.819909	no
Mettl18	4.59074	4.41423	-0.0565657	0.81215	0.891385	no
Mettl2	11.1529	12.0619	0.113035	0.4837	0.639216	no
Mettl20	5.21638	6.497	0.316724	0.13745	0.249659	no
Mettl21a	7.35616	7.15277	-0.0404501	0.8168	0.894236	no
Mettl22	5.50312	6.71125	0.286331	0.19895	0.33485	no
Mettl23	129.3	114.482	-0.17559	0.2166	0.357281	no
Mettl25	3.94251	3.71369	-0.0862597	0.69365	0.811604	no
Mettl3	27.8064	25.1441	-0.145197	0.59095	0.73234	no
Mettl4	8.87536	8.48894	-0.0642209	0.7226	0.831649	no
Mettl5	18.2343	17.8022	-0.0345944	0.9537	0.975156	no
Mettl6	13.2451	12.4683	-0.0872021	0.6059	0.74441	no
Mettl7a1	17.3054	19.5066	0.17274	0.2812	0.431012	no
Mettl8	8.73152	10.6041	0.280316	0.1031	0.198186	no
Mettl9	20.4492	24.4076	0.255287	0.10375	0.199202	no
Mex3b	5.56544	4.89343	-0.185649	0.2925	0.443678	no
Mex3c	10.8484	10.4828	-0.0494629	0.75305	0.852379	no
Mex3d	2.29037	2.36693	0.0474377	0.8306	0.902777	no
Mfap1a	15.8442	14.902	-0.0884517	0.55275	0.700334	no
Mfap1b	4.9217	4.95612	0.0100542	0.9596	0.978255	no
Mfap3	9.4446	8.98869	-0.0713788	0.63885	0.770726	no
Mff	45.6565	41.3051	-0.144497	0.3194	0.47397	no
Mfge8	12.229	11.4508	-0.0948591	0.5707	0.715295	no
Mfhas1	4.61804	4.11289	-0.167126	0.30775	0.461005	no
Mfn1	6.86957	6.65643	-0.0454708	0.7773	0.868875	no
Mfn2	16.6886	17.3231	0.0538317	0.7091	0.822415	no
Mfng	75.5397	89.8398	0.250119	0.076	0.153866	no
Mfsd1	41.6556	40.5623	-0.0383725	0.7912	0.878168	no
Mfsd10	19.0964	19.8686	0.0571875	0.71745	0.82795	no
Mfsd11	9.72108	9.50194	-0.0328946	0.8375	0.906924	no
Mfsd12	1.34127	2.09546	0.643666	0.0487	0.10635	no
Mfsd2a	0.69719	1.43827	1.04471	0.00385	0.0118893	yes
Mfsd3	1.70262	1.82158	0.0974293	0.766	0.861167	no
Mfsd4	5.75197	6.22447	0.113896	0.5258	0.677276	no

Mfsd5	24.7477	24.7556	0.000459171	0.99735	0.998	no
Mfsd6	7.54808	7.71823	0.0321602	0.83995	0.908323	no
Mfsd7b	5.55663	5.68467	0.0328661	0.85505	0.917912	no
Mfsd8	8.37264	7.93883	-0.0767576	0.64435	0.774937	no
Mga	8.32918	9.7454	0.226547	0.1083	0.206383	no
Mgat1	41.1411	40.2311	-0.0322684	0.8174	0.894657	no
Mgat2	45.0245	47.5808	0.0796677	0.5784	0.721806	no
Mgat4a	5.11178	4.90369	-0.0599591	0.7003	0.816057	no
Mgat4b	3.07188	4.02893	0.391274	0.0664	0.137579	no
Mgat5	2.80193	3.4094	0.283098	0.16505	0.28877	no
Mgea5	100.526	104.653	0.0580368	0.6923	0.810683	no
Mgme1	3.50371	4.00576	0.193191	0.33385	0.48979	no
Mgrn1	5.24698	7.0708	0.430388	0.1664	0.290707	no
Mgst2	68.0504	64.453	-0.0783552	0.6401	0.771535	no
Mgst3	4.67721	4.15325	-0.17141	0.6167	0.753339	no
Mib1	5.92652	7.1799	0.27678	0.098	0.190196	no
Mib2	13.5345	13.5576	0.00246499	0.9856	0.99167	no
Mical1	105.784	94.2138	-0.16711	0.2449	0.390017	no
Mical2	2.01474	1.39289	-0.532516	0.00805	0.0226766	yes
Micall1	8.30975	9.11759	0.133847	0.38205	0.541114	no
Micu1	14.1195	13.3158	-0.0845577	0.59715	0.737447	no
Micu2	55.097	53.2949	-0.0479762	0.73635	0.841301	no
Micu3	1.91797	2.34274	0.288619	0.2011	0.337447	no
Mid1ip1	22.1893	18.7085	-0.246172	0.1075	0.205141	no
Midn	27.6604	30.9805	0.163539	0.2528	0.397114	no
Mief1	8.68418	8.77842	0.015572	0.91755	0.954805	no
Mief2	10.8259	10.0366	-0.109216	0.51205	0.665173	no
Mien1	133.604	133.327	-0.00299869	0.984	0.990695	no
Mier1	38.9352	37.8436	-0.0410232	0.7723	0.865479	no
Mier2	6.30713	6.17732	-0.0300014	0.8737	0.929049	no
Mier3	4.44693	3.93531	-0.176332	0.30145	0.453957	no
Mif	191.572	246.922	0.366165	0.012	0.0320694	yes
Mif4gd	45.8542	51.6093	0.170579	0.2558	0.400712	no
Miip	39.3487	35.98	-0.129121	0.38765	0.546839	no
Milr1	4.52197	5.20411	0.202701	0.51015	0.663486	no
Mina	60.7586	57.3709	-0.0827691	0.65295	0.781737	no
Mink1	38.7729	38.281	-0.0184206	0.90045	0.944896	no
Minos1	30.2897	28.8782	-0.0688433	0.6422	0.773313	no
Minpp1	18.3253	18.3528	0.00216253	0.9881	0.993038	no
Mios	9.5874	9.36033	-0.0345805	0.82545	0.899714	no
Mipep	5.15644	5.54969	0.106032	0.57835	0.721769	no
Mipol1	1.02248	1.1976	0.22807	0.83725	0.906751	no
Mis12	13.2951	11.6789	-0.186987	0.43095	0.58922	no
Mis18a	32.3979	28.3312	-0.193509	0.2193	0.360396	no
Mis18bp1	6.22256	3.80178	-0.710832	0.00015	0.000653255	yes
Mitd1	27.956	30.1262	0.107858	0.50485	0.6589	no
Mki67	63.2054	38.2537	-0.724447	5.00E-05	0.000236281	yes
Mkks	8.04815	7.2419	-0.152289	0.43075	0.589004	no
Mkl1	53.3364	51.4626	-0.0515962	0.72225	0.831409	no
Mkl2	3.67974	4.21484	0.195874	0.2425	0.387295	no
Mkln1	19.0546	18.4067	-0.049906	0.73185	0.838059	no
Mknk1	30.5493	28.9436	-0.0778938	0.58885	0.730661	no
Mknk2	231.98	228.101	-0.0243315	0.8691	0.926349	no
Mkrn1	58.7711	57.2638	-0.0374826	0.78885	0.876842	no
Mkrn2	18.0539	16.8495	-0.0996055	0.7907	0.877931	no
Mks1	6.36067	7.80327	0.2949	0.1054	0.20175	no
Mlec	36.2204	38.3069	0.0808007	0.5665	0.711763	no

Mlf1	1.26479	0.788866	-0.681047	0.12895	0.237483	no
Mlf2	87.1709	80.5666	-0.113664	0.42815	0.586437	no
Mlh1	10.6364	11.1044	0.0621193	0.70675	0.820782	no
Mlh3	2.0238	2.12948	0.0734343	0.70505	0.819563	no
Mlkl	3.30013	3.43519	0.057869	0.8118	0.891125	no
Mllt1	34.3603	34.6435	0.0118449	0.93555	0.964971	no
Mllt10	19.6159	19.2873	-0.0243697	0.91345	0.952575	no
Mllt11	6.20947	6.73113	0.11638	0.92575	0.95949	no
Mllt3	15.8131	15.6418	-0.0157181	0.9129	0.952325	no
Mllt4	1.06038	1.41072	0.411856	0.0475	0.104139	no
Mllt6	49.5372	53.4167	0.108778	0.44715	0.604612	no
Mlst8	11.933	12.0619	0.0155018	0.92115	0.957136	no
Mlx	43.3555	42.4176	-0.0315523	0.83255	0.903961	no
Mlxip	15.8385	18.0529	0.1888	0.19075	0.323899	no
Mlycd	11.006	12.0203	0.127181	0.4505	0.607965	no
Mmaa	2.47626	2.75014	0.151339	0.50135	0.655472	no
Mmab	2.48087	3.00478	0.276412	0.1933	0.327229	no
Mmachc	1.96397	2.42599	0.304802	0.24975	0.395297	no
Mmadhc	42.6842	40.9392	-0.0602171	0.69145	0.810091	no
Mmd	14.2575	16.2476	0.188504	0.21905	0.360102	no
Mmgt1	7.83747	6.32617	-0.309056	0.05395	0.115957	no
Mmgt2	15.1306	11.5077	-0.394866	0.0206	0.0511824	no
Mms19	27.7841	25.1496	-0.143723	0.36365	0.521902	no
Mms22l	7.80135	6.00127	-0.378456	0.0172	0.0436852	yes
Mnat1	6.099	5.91227	-0.0448613	0.8069	0.888011	no
Mndal	191.022	172.69	-0.145557	0.30505	0.457844	no
Mns1	18.0028	15.1949	-0.244642	0.2243	0.366649	no
Mnt	11.676	11.6072	-0.0085245	0.95345	0.975084	no
Moap1	0.79827	1.02086	0.354833	0.68355	0.804239	no
Mob1a	106.29	98.5999	-0.108346	0.44125	0.599148	no
Mob1b	6.53044	6.74953	0.0476059	0.77935	0.870257	no
Mob2	40.6763	44.5678	0.131812	0.3875	0.546672	no
Mob3a	99.9008	100.891	0.0142302	0.91945	0.956049	no
Mob3c	9.09086	10.2143	0.168098	0.3119	0.465551	no
Mob4	18.6587	17.5912	-0.0849905	0.5712	0.715719	no
Mocos	3.29796	3.57436	0.116109	0.56295	0.708817	no
Mocs1	8.70852	8.67342	-0.00582661	0.97145	0.98428	no
Mocs2	18.177	17.5814	-0.0480682	0.7661	0.861239	no
Mocs3	7.15613	6.55829	-0.12586	0.5181	0.670564	no
Mogs	24.8872	30.0797	0.27339	0.0601	0.127104	no
Mon1a	17.306	17.0456	-0.0218753	0.8948	0.941332	no
Mon1b	15.7025	14.0596	-0.159435	0.27425	0.422884	no
Mon2	14.6427	14.5316	-0.0109815	0.93495	0.964634	no
Morc2a	11.3097	14.0468	0.312681	0.03455	0.0794645	no
Morc3	36.421	33.7687	-0.109083	0.44405	0.601827	no
Morf4l1	144.439	131.393	-0.136573	0.33815	0.494475	no
Morf4l2	34.0631	30.3972	-0.164274	0.2863	0.436752	no
Morn2	0.94304	1.30303	0.466481	0.7503	0.85078	no
Mospd1	10.2589	7.32494	-0.485985	0.0061	0.0177994	yes
Mospd2	4.60572	4.274	-0.107841	0.5616	0.707698	no
Mospd3	57.1618	53.9102	-0.0844954	0.56745	0.712487	no
Mov10	8.24533	9.33359	0.178855	0.2703	0.418201	no
Mpc1	54.7188	50.5229	-0.115098	0.639	0.770756	no
Mpc2	40.9298	41.0913	0.00568074	0.97245	0.98484	no
Mpdu1	53.8752	53.9046	0.000786542	0.9936	0.995999	no
Mpg	9.92428	8.99302	-0.142156	0.5837	0.726417	no
Mphosph10	15.1708	16.7074	0.139195	0.37695	0.535826	no

Mphosph6	20.2004	19.4553	-0.0542192	0.7554	0.854011	no
Mphosph8	15.4005	15.161	-0.0226063	0.89355	0.940507	no
Mphosph9	5.912	5.6202	-0.0730259	0.62995	0.763617	no
Mpi	5.76233	5.18702	-0.151747	0.46375	0.620802	no
Mplkip	11.6199	12.5302	0.108812	0.5033	0.657436	no
Mpnd	56.3622	47.2952	-0.253032	0.31735	0.471512	no
Mpp1	11.9702	11.1767	-0.0989591	0.7119	0.824281	no
Mpp6	11.3103	10.4347	-0.116249	0.48785	0.642918	no
Mpp7	1.40565	1.47385	0.068354	0.78895	0.876884	no
Mppe1	48.034	51.9761	0.113793	0.6073	0.745534	no
Mprip	11.342	13.2517	0.224506	0.11285	0.213292	no
Mpst	26.3032	23.4793	-0.163849	0.3099	0.463389	no
Mpv17	14.8532	14.2319	-0.0616402	0.94545	0.970455	no
Mpv17l	8.04734	9.37142	0.219756	0.1771	0.305319	no
Mpv17l2	28.7838	25.7476	-0.16082	0.35785	0.51577	no
Mpzl3	4.79874	5.3928	0.168379	0.52105	0.673098	no
Mr1	7.13319	6.5457	-0.123998	0.49175	0.646465	no
Mre11a	10.2743	10.0525	-0.0314781	0.8403	0.908618	no
Mrfap1	78.316	88.9485	0.183663	0.19645	0.331465	no
Mrgbp	18.1416	17.6143	-0.0425561	0.8101	0.890159	no
Mri1	17.6833	20.1789	0.190458	0.2166	0.357281	no
Mrm1	6.74652	5.92208	-0.188039	0.2969	0.448713	no
Mroh1	11.9722	13.3927	0.161757	0.44235	0.600226	no
Mrpl1	8.13294	7.87924	-0.0457192	0.81385	0.892266	no
Mrpl10	47.8336	44.5426	-0.102838	0.48045	0.63616	no
Mrpl11	7.8556	7.88169	0.00478313	0.9776	0.987534	no
Mrpl12	60.3641	58.6141	-0.0424434	0.78165	0.871722	no
Mrpl13	55.4092	50.6566	-0.129376	0.4136	0.572462	no
Mrpl14	43.506	37.528	-0.213246	0.23375	0.377757	no
Mrpl15	16.3793	14.9689	-0.129903	0.4411	0.599013	no
Mrpl16	29.8529	26.8151	-0.154824	0.3431	0.49988	no
Mrpl17	3.09319	3.65082	0.239127	0.16155	0.283835	no
Mrpl18	94.1947	86.4772	-0.123326	0.3993	0.558319	no
Mrpl19	8.2566	8.28111	0.00427629	0.9794	0.988433	no
Mrpl2	26.6556	27.8226	0.0618169	0.7055	0.819802	no
Mrpl20	73.6902	70.5701	-0.0624148	0.69695	0.813914	no
Mrpl21	31.9347	32.6489	0.0319094	0.8456	0.911779	no
Mrpl22	26.3576	28.399	0.107622	0.55955	0.706072	no
Mrpl23	42.9404	47.0436	0.131663	0.45935	0.616666	no
Mrpl24	91.5317	98.977	0.112822	0.5252	0.6768	no
Mrpl27	56.9418	49.5293	-0.201207	0.216	0.356516	no
Mrpl28	31.6982	29.6064	-0.0984944	0.5515	0.699365	no
Mrpl3	30.6961	29.9426	-0.0358527	0.8118	0.891125	no
Mrpl30	110.097	88.9736	-0.307323	0.0421	0.0940408	no
Mrpl32	59.6626	55.4974	-0.104407	0.51445	0.667284	no
Mrpl33	307.14	284.552	-0.110203	0.44875	0.606137	no
Mrpl34	60.6888	60.6225	-0.00157638	0.99275	0.995486	no
Mrpl35	6.74735	6.73879	-0.00183076	0.9871	0.992486	no
Mrpl36	51.9162	53.7955	0.051301	0.7408	0.844294	no
Mrpl37	29.5232	28.6071	-0.0454766	0.76535	0.860738	no
Mrpl38	18.3781	19.0147	0.0491255	0.77165	0.86508	no
Mrpl39	20.4971	22.2363	0.117499	0.45655	0.614099	no
Mrpl4	70.2907	63.6794	-0.142505	0.3237	0.47878	no
Mrpl40	46.9697	44.4803	-0.0785645	0.60785	0.745881	no
Mrpl41	11.2387	10.2629	-0.131041	0.51815	0.670579	no
Mrpl42	80.2269	70.4916	-0.186635	0.22795	0.370975	no
Mrpl43	49.4509	47.9235	-0.0452646	0.76835	0.862724	no

Mrpl44	20.8745	20.7085	-0.0115169	0.94485	0.970126	no
Mrpl45	11.6371	11.6314	-0.000700673	0.9955	0.997005	no
Mrpl46	27.1989	26.8556	-0.0183255	0.9134	0.952565	no
Mrpl47	11.6944	12.05	0.043222	0.8367	0.906434	no
Mrpl48	37.0636	35.28	-0.0711524	0.81775	0.894804	no
Mrpl49	24.8861	24.0802	-0.0474922	0.841	0.909041	no
Mrpl50	11.5815	10.5735	-0.131372	0.43195	0.59013	no
Mrpl51	26.7506	28.3537	0.0839649	0.58925	0.730965	no
Mrpl52	164.385	183.884	0.161716	0.3021	0.454664	no
Mrpl53	32.032	29.3624	-0.125545	0.64025	0.771584	no
Mrpl54	74.3098	63.486	-0.227113	0.1691	0.294222	no
Mrpl55	16.5694	16.729	0.0138317	0.9443	0.969787	no
Mrpl9	88.7523	87.4082	-0.0220154	0.87725	0.931183	no
Mrps10	13.1152	13.4826	0.0398504	0.8372	0.906751	no
Mrps11	19.6301	19.9227	0.0213444	0.9035	0.946731	no
Mrps12	40.4582	44.3031	0.130977	0.4396	0.597586	no
Mrps14	21.0877	21.6231	0.0361717	0.8172	0.894549	no
Mrps15	51.7574	51.9566	0.00554297	0.97045	0.983916	no
Mrps16	245.514	226.329	-0.117382	0.4118	0.570698	no
Mrps17	31.855	33.3501	0.0661703	0.67385	0.797138	no
Mrps18a	32.3961	31.5728	-0.037138	0.83005	0.902457	no
Mrps18b	21.7211	25.5882	0.236383	0.16515	0.288923	no
Mrps18c	105.085	96.2086	-0.127315	0.4279	0.586231	no
Mrps2	13.1403	13.7827	0.0688539	0.67235	0.796011	no
Mrps21	181.227	170.678	-0.0865134	0.5846	0.727126	no
Mrps22	9.99138	8.68602	-0.201987	0.3174	0.471567	no
Mrps23	68.2829	61.5548	-0.149654	0.3241	0.479191	no
Mrps24	50.7033	55.3656	0.12691	0.41375	0.572591	no
Mrps25	17.0008	15.2934	-0.152693	0.40965	0.568634	no
Mrps26	66.2207	68.6621	0.0522324	0.72985	0.836447	no
Mrps27	6.81211	7.71984	0.180469	0.3543	0.511887	no
Mrps28	13.6146	17.68	0.376958	0.07855	0.158126	no
Mrps30	27.2531	25.4817	-0.0969583	0.53565	0.685983	no
Mrps31	11.3786	10.0269	-0.182442	0.3199	0.474542	no
Mrps33	42.2124	42.9256	0.0241724	0.88025	0.932877	no
Mrps34	34.7786	36.9836	0.0886861	0.5969	0.737254	no
Mrps35	36.0472	37.3337	0.050592	0.7536	0.852755	no
Mrps36	53.7864	48.8005	-0.140344	0.38815	0.547348	no
Mrps5	25.4835	32.2855	0.341323	0.0313	0.0731359	no
Mrps6	26.6811	35.0151	0.392163	0.0413	0.0924997	no
Mrps7	33.1712	33.7375	0.0244223	0.87245	0.928391	no
Mrps9	35.6858	33.59	-0.0873182	0.57065	0.715258	no
Mrrf	6.00823	6.23515	0.0534832	0.8653	0.924071	no
Mrs2	16.385	16.7348	0.0304765	0.84915	0.913955	no
Mrto4	18.3954	19.0212	0.0482587	0.7785	0.869611	no
Ms4a4b	1787.89	1645.28	-0.119919	0.45525	0.612934	no
Ms4a4c	9.5711	10.8778	0.184628	0.3081	0.461362	no
Ms4a4d	2.11306	2.10317	-0.00677029	0.97935	0.988425	no
Ms4a6b	492.203	512.927	0.0594987	0.6818	0.802997	no
Ms4a6c	16.0591	14.0878	-0.188943	0.3851	0.544091	no
Ms4a6d	10.0592	8.49165	-0.244392	0.21115	0.350577	no
Msantd2	9.15408	9.21997	0.0103476	0.9546	0.975709	no
Msantd4	7.73552	7.11045	-0.121558	0.4813	0.63695	no
Msc	8.7115	3.2711	-1.41314	5.00E-05	0.000236281	yes
Msh2	28.0024	28.1002	0.00503013	0.973	0.985143	no
Msh3	11.1709	10.5059	-0.0885383	0.63335	0.766435	no
Msh6	10.7326	9.42799	-0.186971	0.4734	0.629814	no

Msi2	9.52803	11.1055	0.221022	0.13145	0.241206	no
Msl1	32.133	31.9083	-0.0101268	0.9595	0.978196	no
Msl2	39.9472	40.4824	0.0191997	0.892	0.939573	no
Msl3	15.9999	14.4421	-0.147783	0.33255	0.488258	no
Msmp	13.4085	15.7046	0.228046	0.7623	0.85869	no
Msn	431.531	414.659	-0.0575378	0.7182	0.828558	no
Msra	10.2545	10.2886	0.00479303	0.9782	0.987801	no
Msrb1	51.942	44.5962	-0.21998	0.16425	0.28767	no
Msrb2	3.68732	3.94491	0.0974208	0.71665	0.827514	no
Mss51	2.24737	2.34355	0.0604559	0.82045	0.896521	no
Msto1	17.6978	17.961	0.0212944	0.9443	0.969787	no
Mt1	36.0256	34.6265	-0.0571451	0.7649	0.860534	no
Mt2	14.5005	13.0193	-0.155455	0.5384	0.688131	no
Mt3	9.93827	6.61089	-0.58815	0.0516	0.111667	no
Mta1	44.5103	44.575	0.00209724	0.988	0.992966	no
Mta2	144.246	147.196	0.0292007	0.8385	0.907423	no
Mta3	76.6464	80.661	0.0736547	0.616	0.752796	no
Mtap	18.4399	17.7774	-0.0527829	0.73125	0.837589	no
Mtap7d3	0.58187	1.11308	0.935782	0.00835	0.0234077	yes
Mtbp	9.13159	8.19072	-0.156875	0.3381	0.494422	no
Mtch1	62.4213	59.5841	-0.0671123	0.6367	0.768973	no
Mtch2	32.0858	35.5602	0.148328	0.31295	0.466655	no
Mtcp1	10.272	10.1818	-0.0127258	0.9524	0.974547	no
Mtdh	37.5522	46.5737	0.310619	0.03	0.0705373	no
Mterf1a	4.35805	4.40217	0.0145306	0.95305	0.974859	no
Mterf1b	2.38256	2.27325	-0.0677544	0.8188	0.895453	no
Mterfd1	27.6919	28.1368	0.022997	0.93275	0.963294	no
Mterfd2	3.39471	3.40499	0.0043625	0.9771	0.987283	no
Mterfd3	7.87851	7.84058	-0.00696198	0.9747	0.986157	no
Mtf1	5.56042	5.88643	0.0821995	0.6118	0.749116	no
Mtf2	12.3471	11.9294	-0.0496546	0.74585	0.847777	no
Mtfmt	12.8043	13.2187	0.0459614	0.7798	0.870512	no
Mtfp1	0.981654	1.04301	0.0874615	0.8278	0.901065	no
Mtfr1	12.211	12.1519	-0.00699819	0.96395	0.980433	no
Mtfr1l	33.4341	34.0426	0.0260236	0.8619	0.921932	no
Mtfr2	4.01669	2.21211	-0.860583	5.00E-04	0.00193907	yes
Mtg1	10.1314	10.4154	0.0398913	0.8243	0.898947	no
Mtg2	17.0144	17.9369	0.0761775	0.64765	0.777449	no
Mthfd1	9.44213	10.6457	0.173083	0.271	0.418962	no
Mthfd1l	15.8016	14.6618	-0.108013	0.47305	0.629514	no
Mthfd2	26.8534	28.2486	0.0730763	0.6333	0.766414	no
Mthfd2l	1.20163	1.41967	0.240561	0.41435	0.573129	no
Mthfr	4.18928	3.95003	-0.0848404	0.6141	0.751177	no
Mthfs	19.37	18.7938	-0.0435685	0.8235	0.89838	no
Mthfsd	7.37169	7.28018	-0.0180202	0.929	0.961217	no
Mtif2	19.4096	19.8565	0.0328421	0.8242	0.898921	no
Mtif3	8.08761	8.24842	0.0284044	0.89925	0.94424	no
Mtm1	1.01447	0.980368	-0.0493256	0.86095	0.921489	no
Mtmr1	27.6188	30.9023	0.162064	0.25415	0.398747	no
Mtmr10	2.11038	1.95822	-0.107964	0.58035	0.723536	no
Mtmr12	29.7883	26.8832	-0.148044	0.29735	0.449239	no
Mtmr14	20.2801	18.3595	-0.143531	0.34175	0.498325	no
Mtmr2	16.4802	15.9791	-0.0445502	0.774	0.866696	no
Mtmr3	27.5379	27.5245	-0.000698871	0.9967	0.997633	no
Mtmr4	9.23193	9.47449	0.0374167	0.8047	0.886863	no
Mtmr6	37.0535	34.7229	-0.0937198	0.5113	0.664492	no
Mtmr7	1.82185	1.85302	0.0244768	0.97135	0.984263	no

Mtmr9	17.9376	15.5118	-0.209622	0.1748	0.302062	no
Mto1	8.81266	8.57621	-0.0392368	0.82115	0.896953	no
Mtor	7.22587	8.42793	0.222007	0.129	0.237557	no
Mtpap	19.1146	18.5327	-0.0446001	0.77115	0.864712	no
Mtpn	101.729	88.7312	-0.197219	0.166	0.290101	no
Mtr	8.30603	10.1429	0.288238	0.06415	0.133829	no
Mtrf1	3.21211	3.17237	-0.0179585	0.93815	0.966566	no
Mtrf1l	7.15408	7.25858	0.0209217	0.90875	0.949752	no
Mtrr	6.17011	6.4126	0.0556139	0.73825	0.84264	no
Mturn	3.33112	3.11333	-0.0975473	0.6018	0.741309	no
Mtx1	18.0451	17.811	-0.0188401	0.9089	0.949752	no
Mtx2	14.3027	13.6803	-0.0641828	0.7226	0.831649	no
Mtx3	1.94611	2.22742	0.194784	0.29725	0.449107	no
Mul1	5.09392	5.00603	-0.0251097	0.88465	0.935436	no
Mum1	20.0184	20.2499	0.016592	0.9095	0.950084	no
Mus81	20.3766	19.0806	-0.0948093	0.54655	0.695074	no
Mut	9.05637	9.71015	0.100562	0.523	0.674765	no
Mutyh	6.25233	5.60194	-0.158466	0.45235	0.609879	no
Mvb12a	55.5776	52.9926	-0.0687132	0.66145	0.788123	no
Mvb12b	3.01057	5.34787	0.828926	5.00E-05	0.000236281	yes
Mvd	16.4525	14.3229	-0.199988	0.2234	0.3655	no
Mvk	3.39409	3.51975	0.0524494	0.8348	0.905362	no
Mvp	15.1151	14.8764	-0.0229659	0.87885	0.93225	no
Mx1	24.1225	19.385	-0.315442	0.0339	0.0782142	no
Mxd1	7.37869	6.6905	-0.14125	0.3773	0.536204	no
Mxd3	21.0291	12.5977	-0.739224	0.26145	0.407519	no
Mxd4	57.715	44.0005	-0.391426	0.00925	0.0256214	yes
Mxi1	7.16685	5.76212	-0.31474	0.0483	0.105617	no
Myadm	20.1885	17.8861	-0.174693	0.24785	0.3931	no
Myb	6.98682	6.85942	-0.0265491	0.8751	0.929879	no
Mybbp1a	30.0292	35.2306	0.230465	0.11075	0.21019	no
Mybl1	1.06276	0.599986	-0.824818	0.00325	0.0102567	yes
Mybl2	7.34144	6.11123	-0.2646	0.1107	0.210123	no
Myc	13.8707	17.5158	0.336621	0.03375	0.0779242	no
Mycbp	9.11766	7.9746	-0.193251	0.31565	0.46957	no
Mycbp2	27.1406	34.1779	0.332609	0.021	0.0520425	no
Myd88	53.6996	51.5441	-0.059105	0.6794	0.801374	no
Myef2	13.39	12.9555	-0.0475919	0.76525	0.860736	no
Myeov2	224.578	221.947	-0.0169961	0.91185	0.951623	no
Myg1	27.2766	25.1408	-0.117634	0.45945	0.61673	no
Myh9	377.849	418.758	0.148308	0.40685	0.565693	no
Myl12a	392.541	356.132	-0.140431	0.33455	0.490511	no
Myl12b	650.283	657.386	0.0156742	0.91365	0.952699	no
Myl4	3.96342	4.56156	0.202778	0.4853	0.640587	no
Myl6	1983.99	1845.06	-0.104736	0.4764	0.632625	no
Myl6b	2.40771	1.76757	-0.445894	0.21185	0.351425	no
Mylip	23.2986	20.5076	-0.184081	0.21785	0.358714	no
Mylpf	6.50634	6.43502	-0.0159023	0.9498	0.972973	no
Mynn	7.71466	7.97587	0.0480397	0.75665	0.854822	no
Myo18a	22.1877	19.2667	-0.203648	0.155	0.274438	no
Myo19	2.64892	3.15625	0.252809	0.3049	0.457687	no
Myo1c	6.30591	6.21682	-0.020528	0.8965	0.942516	no
Myo1e	1.44264	1.7189	0.252766	0.23855	0.383425	no
Myo1f	232.164	240.775	0.0525379	0.723	0.831852	no
Myo1g	211.473	199.978	-0.0806261	0.572	0.716352	no
Myo5a	4.82166	4.33188	-0.154538	0.3048	0.457634	no
Myo9a	1.32512	1.13443	-0.224149	0.2206	0.361951	no

Myo9b	53.7647	60.7336	0.175836	0.2175	0.358339	no
Mypop	3.37636	3.29667	-0.0344585	0.87825	0.931866	no
Mysm1	10.4711	10.4435	-0.00380285	0.98	0.988614	no
Mzt1	27.0983	26.7514	-0.0185895	0.90055	0.944944	no
Mzt2	7.09348	7.05254	-0.00834892	0.96575	0.981322	no
N4bp1	25.6122	18.9466	-0.434896	0.00255	0.00827285	yes
N4bp2	4.58096	10.6745	1.22044	5.00E-05	0.000236281	yes
N4bp2l1	28.0445	29.3825	0.0672439	0.65925	0.786465	no
N4bp2l2	7.26211	7.53077	0.0524072	0.72125	0.830732	no
N4bp3	7.71081	7.76502	0.0101071	0.95655	0.976726	no
N6amt1	8.95177	10.4602	0.224659	0.2165	0.357175	no
N6amt2	31.4147	29.6786	-0.0820149	0.63285	0.766106	no
Naa10	66.4107	60.7926	-0.127521	0.40235	0.561294	no
Naa15	20.1685	19.4105	-0.0552669	0.7014	0.816826	no
Naa16	13.0192	11.3172	-0.202124	0.18565	0.317039	no
Naa20	36.094	36.3119	0.00868341	0.95535	0.976122	no
Naa25	7.22154	9.40586	0.381254	0.0223	0.0548291	no
Naa30	12.7295	13.7392	0.110132	0.45385	0.611481	no
Naa35	20.2616	20.4948	0.0165131	0.9146	0.953169	no
Naa38	67.9818	59.8298	-0.184286	0.3011	0.453536	no
Naa40	19.5585	18.7987	-0.0571624	0.69385	0.81173	no
Naa50	20.0307	17.5846	-0.187903	0.1944	0.328752	no
Naa60	69.4355	62.6413	-0.148558	0.2927	0.443915	no
Naaa	5.71715	5.26007	-0.120213	0.5223	0.674207	no
Nab1	42.037	33.562	-0.324829	0.0227	0.0556652	no
Nab2	11.7668	11.5612	-0.0254237	0.9625	0.979895	no
Nabp1	36.745	29.0816	-0.337442	0.0204	0.0507373	no
Nabp2	77.1566	70.4566	-0.131055	0.3717	0.530274	no
Naca	747.905	757.711	0.0187932	0.89845	0.943765	no
Nacc1	31.5836	32.8707	0.0576273	0.68615	0.806237	no
Nadk	39.3774	40.9278	0.0557137	0.69325	0.811338	no
Nadk2	6.49931	6.96389	0.0996072	0.57775	0.721212	no
Nadsyn1	7.50032	6.68034	-0.16703	0.3475	0.504634	no
Nae1	32.2014	31.7695	-0.0194832	0.89655	0.942555	no
Naf1	3.32917	3.91928	0.235424	0.23395	0.377968	no
Naga	19.3798	17.2928	-0.164379	0.30335	0.456049	no
Nagk	7.54236	6.73214	-0.163952	0.43395	0.592232	no
Naglu	4.80173	5.39448	0.16793	0.37895	0.537954	no
Nagpa	14.6397	14.4334	-0.0204677	0.8986	0.943838	no
Naif1	2.01009	1.88531	-0.0924584	0.7932	0.879351	no
Naip2	3.34515	2.77019	-0.272089	0.1394	0.252143	no
Nampt	7.33824	6.64573	-0.143007	0.37115	0.529704	no
Nanos1	1.90528	1.88423	-0.0160291	0.94485	0.970126	no
Nanp	7.35486	6.91468	-0.0890349	0.66965	0.79412	no
Nans	59.2014	49.4351	-0.260098	0.07025	0.144244	no
Nap1l1	216.278	182.457	-0.245334	0.1033	0.198522	no
Nap1l4	93.6423	94.5423	0.0137997	0.9246	0.958829	no
Napa	38.2267	40.1421	0.0705359	0.6229	0.758052	no
Napepld	1.32411	1.45455	0.135549	0.57785	0.721285	no
Napg	19.857	18.0148	-0.140464	0.3453	0.502214	no
Napsa	1.76341	1.41004	-0.322636	0.32985	0.485516	no
Narf	10.4314	11.5235	0.143646	0.34035	0.496796	no
Narfl	10.8025	10.4523	-0.0475473	0.7944	0.880197	no
Narg2	6.31952	7.30282	0.208638	0.20635	0.34426	no
Nars	52.5785	51.5978	-0.0271648	0.8492	0.913955	no
Nars2	1.451	1.70126	0.229553	0.3318	0.487502	no
Nasp	32.2232	24.8645	-0.374014	0.0142	0.037055	yes

Nat10	20.054	24.1462	0.267903	0.0638	0.133252	no
Nat14	1.77338	1.66443	-0.0914736	0.7666	0.86154	no
Nat2	8.88577	8.23885	-0.109053	0.5726	0.716862	no
Nat6	4.35464	4.74826	0.124846	0.5535	0.701032	no
Nat9	6.55098	7.04211	0.104295	0.65345	0.782157	no
Nbas	4.36141	5.56038	0.350391	0.02725	0.0650993	no
Nbeal1	1.43114	1.70439	0.252095	0.1464	0.261978	no
Nbeal2	106.818	114.23	0.0967897	0.51175	0.664893	no
Nbn	6.25592	6.09313	-0.038039	0.83635	0.906263	no
Nbr1	28.2367	28.5581	0.0163287	0.90905	0.949825	no
Ncald	31.8902	27.3855	-0.219703	0.1256	0.232643	no
Ncapd2	38.8633	28.1115	-0.467247	0.00095	0.00343861	yes
Ncapd3	16.9793	13.6119	-0.31891	0.02795	0.0664836	no
Ncapg	14.0441	8.00014	-0.811863	0.00055	0.0021091	yes
Ncapg2	7.93824	4.62934	-0.778011	5.00E-05	0.000236281	yes
Ncaph	21.2479	12.0506	-0.818219	5.00E-05	0.000236281	yes
Ncaph2	47.3339	41.17	-0.201278	0.19695	0.332181	no
Ncbp1	50.8315	49.8527	-0.0280509	0.84175	0.909559	no
Ncbp2	30.9934	30.1037	-0.0420186	0.7762	0.868154	no
Nccrp1	3.29822	4.60024	0.480024	0.0471	0.103403	no
Ncdn	21.2004	23.3508	0.139379	0.32785	0.483419	no
Nceh1	6.20984	7.57569	0.286822	0.0739	0.1503	no
Ncf1	6.92208	7.26049	0.0688608	0.70515	0.819625	no
Ncf4	39.0943	33.6514	-0.216292	0.15975	0.281275	no
Nck1	37.4473	33.9873	-0.139865	0.3528	0.510378	no
Nck2	17.6528	20.6554	0.226628	0.13555	0.247066	no
Nckap1	5.54281	6.0843	0.134474	0.41605	0.574749	no
Nckap1l	103.846	93.8443	-0.146099	0.303	0.455659	no
Nckap5l	8.56503	10.5004	0.293908	0.05225	0.112842	no
Nckipsd	5.12545	5.47695	0.0956946	0.5982	0.73833	no
Ncl	70.6395	67.4595	-0.0664535	0.64415	0.774815	no
Ncln	56.82	55.0959	-0.0444559	0.7545	0.853431	no
Ncoa1	9.50345	8.90202	-0.0943176	0.5222	0.674152	no
Ncoa2	21.3882	21.7751	0.0258651	0.85105	0.915125	no
Ncoa3	19.3778	18.8424	-0.0404233	0.77675	0.868536	no
Ncoa4	28.2079	25.9196	-0.122053	0.3973	0.556393	no
Ncoa5	25.4226	28.0457	0.141668	0.33525	0.491242	no
Ncoa6	14.9423	15.1843	0.0231834	0.8719	0.92803	no
Ncoa7	6.33443	6.42255	0.0199315	0.9067	0.948633	no
Ncor1	66.7574	72.4586	0.118231	0.43865	0.596743	no
Ncor2	13.6976	16.1088	0.233921	0.0997	0.192785	no
Ncstn	38.2039	34.5276	-0.145973	0.66195	0.78854	no
Ndc1	3.79876	3.30269	-0.201886	0.3347	0.490609	no
Ndc80	18.5464	11.7027	-0.664298	5.00E-05	0.000236281	yes
Nde1	55.1352	46.5181	-0.245181	0.08725	0.172699	no
Ndel1	23.1218	22.4985	-0.0394285	0.794	0.87992	no
Ndfip1	246.861	239.828	-0.0417011	0.7689	0.863149	no
Ndfip2	31.7221	29.5244	-0.103582	0.48325	0.638819	no
Ndnl2	21.8117	18.99	-0.199865	0.22495	0.367448	no
Ndor1	16.8047	18.8218	0.163546	0.26505	0.411902	no
Ndrg1	2.73139	2.66946	-0.0330879	0.87755	0.931417	no
Ndrg2	2.39176	1.48952	-0.683223	0.0129	0.0341475	yes
Ndrg3	40.9735	36.025	-0.185692	0.1978	0.333288	no
Ndst1	8.80196	9.74721	0.147165	0.32455	0.479696	no
Ndst2	7.54517	8.40539	0.155761	0.33005	0.48574	no
Ndufa1	216.01	186.855	-0.209179	0.16045	0.282254	no
Ndufa10	110.332	106.285	-0.0539075	0.7054	0.819753	no

Ndufa11	15.4076	14.7296	-0.0649244	0.67445	0.797534	no
Ndufa12	69.8815	79.8179	0.191802	0.24065	0.385698	no
Ndufa13	105.706	100.367	-0.0747774	0.6005	0.740198	no
Ndufa2	175.556	167.267	-0.0697852	0.6913	0.809942	no
Ndufa3	427.015	413.224	-0.0473633	0.7521	0.851891	no
Ndufa4	412.197	368.523	-0.16158	0.2609	0.406841	no
Ndufa5	49.7073	50.7355	0.0295364	0.8665	0.924779	no
Ndufa6	252.852	219.792	-0.202154	0.1604	0.282173	no
Ndufa7	154.513	162.283	0.070789	0.63085	0.764406	no
Ndufa8	104.146	99.1441	-0.0710052	0.6335	0.766551	no
Ndufa9	78.2872	77.8971	-0.00720539	0.9623	0.979819	no
Ndufab1	37.5938	39.516	0.0719436	0.67355	0.796951	no
Ndufaf1	12.1669	11.7214	-0.0538194	0.7647	0.860364	no
Ndufaf2	15.7459	15.2949	-0.0419243	0.84455	0.911371	no
Ndufaf3	16.0882	16.2706	0.0162641	0.9287	0.96108	no
Ndufaf4	4.96794	5.66358	0.189067	0.2773	0.426451	no
Ndufaf5	5.16529	5.61266	0.119837	0.5165	0.669254	no
Ndufaf6	3.93066	4.64433	0.240698	0.36955	0.528115	no
Ndufaf7	10.1154	11.8831	0.232354	0.1667	0.291087	no
Ndufb10	186.779	191.187	0.0336517	0.81975	0.896145	no
Ndufb11	195.925	179.481	-0.126472	0.52535	0.676906	no
Ndufb2	82.7674	87.4573	0.0795166	0.6438	0.77454	no
Ndufb3	130.812	129.468	-0.0148977	0.927	0.960222	no
Ndufb4	110.288	113.951	0.0471402	0.76925	0.863377	no
Ndufb5	95.3018	90.4666	-0.0751186	0.60455	0.74336	no
Ndufb6	90.5665	86.4924	-0.0664033	0.6772	0.799648	no
Ndufb7	257.437	227.371	-0.179172	0.21535	0.355743	no
Ndufb8	110.006	101.991	-0.109141	0.47865	0.634574	no
Ndufb9	163.982	149.198	-0.136309	0.3555	0.513179	no
Ndufc1	78.5564	87.902	0.162166	0.35635	0.514133	no
Ndufc2	82.6797	73.7873	-0.16416	0.29315	0.444464	no
Ndufs1	38.5266	36.9398	-0.0606763	0.6864	0.806383	no
Ndufs2	115.426	112.351	-0.0389576	0.7807	0.871048	no
Ndufs3	84.7519	87.4633	0.0454315	0.7563	0.854564	no
Ndufs4	41.7379	38.0643	-0.13292	0.37775	0.536714	no
Ndufs5	88.0597	81.8737	-0.105083	0.5298	0.680875	no
Ndufs6	110.096	100.079	-0.137617	0.3787	0.537685	no
Ndufs7	76.6559	75.4865	-0.0221777	0.8844	0.935354	no
Ndufs8	90.9272	86.2144	-0.0767825	0.6083	0.746216	no
Ndufv1	45.7794	45.7629	-0.000520875	0.9985	0.998741	no
Ndufv2	56.0878	51.7884	-0.115059	0.4217	0.580102	no
Ndufv3	189.503	182.169	-0.0569482	0.72765	0.835024	no
Nebl	7.48633	5.84741	-0.356462	0.039	0.0881202	no
Necap1	9.35378	7.85326	-0.252258	0.151	0.268684	no
Necap2	43.0372	41.5043	-0.0523232	0.71025	0.823207	no
Nedd1	11.8766	10.7921	-0.138141	0.373	0.531667	no
Nedd4	27.1366	23.8388	-0.186929	0.1912	0.324476	no
Nedd4l	1.82531	2.48161	0.443133	0.0117	0.0313912	yes
Nedd8	236.792	231.281	-0.033973	0.8168	0.894236	no
Nedd9	35.5776	36.0542	0.0191967	0.8877	0.937033	no
Nefh	2.202	4.20929	0.934766	0.12895	0.237483	no
Neil1	10.7565	12.0398	0.162608	0.3976	0.556681	no
Neil3	9.33891	4.33958	-1.1057	5.00E-05	0.000236281	yes
Nek1	2.58975	3.19196	0.301631	0.1119	0.211888	no
Nek2	12.0377	6.53083	-0.882219	5.00E-05	0.000236281	yes
Nek3	3.43074	2.99716	-0.194923	0.39265	0.551764	no
Nek7	36.2192	39.8236	0.136871	0.3393	0.495684	no

Nek8	3.72295	3.40287	-0.129694	0.5175	0.670021	no
Nek9	28.2519	29.8354	0.0786812	0.56785	0.71285	no
Nelfa	16.7605	16.4586	-0.0262205	0.86805	0.925747	no
Nelfb	63.2058	60.2435	-0.0692514	0.6175	0.75399	no
Nelfcd	24.8361	25.5688	0.0419456	0.8255	0.899755	no
Nelfe	18.3049	16.1768	-0.17831	0.29515	0.446671	no
Nemf	25.845	26.2912	0.0246982	0.865	0.92389	no
Nenf	5.6818	4.10908	-0.467533	0.1519	0.269959	no
Net1	9.26329	10.1929	0.137975	0.3743	0.532971	no
Neu1	13.4338	15.7993	0.23399	0.1212	0.225976	no
Neu3	1.4568	1.18851	-0.293651	0.2556	0.400479	no
Neurl2	2.47397	1.87161	-0.402543	0.82885	0.901694	no
Neurl3	24.6889	28.5805	0.211169	0.1572	0.277582	no
Neurl4	13.6076	15.2549	0.164856	0.26115	0.407159	no
Nf2	17.783	22.108	0.314072	0.0372	0.0846301	no
Nfat5	12.9155	12.8147	-0.011301	0.94035	0.967782	no
Nfatc1	51.4299	58.9029	0.195729	0.17015	0.295706	no
Nfatc2	24.3399	28.0238	0.203333	0.1515	0.269377	no
Nfatc2ip	11.571	11.2664	-0.0384873	0.79975	0.883518	no
Nfatc3	125.343	124.629	-0.00824481	0.9557	0.976338	no
Nfe2	1.13958	2.58323	1.18067	0.00095	0.00343861	yes
Nfe2l1	21.3763	22.1608	0.0519974	0.71985	0.829729	no
Nfe2l2	19.2071	26.6091	0.470283	0.0021	0.00696828	yes
Nfic	8.73625	8.09143	-0.110618	0.46075	0.617899	no
Nfil3	6.04718	6.43587	0.0898723	0.6423	0.773394	no
Nfkb1	67.7272	76.8274	0.181886	0.199	0.334903	no
Nfkb2	23.9096	27.3104	0.191864	0.20855	0.347118	no
Nfkbia	328.09	389.763	0.248503	0.08315	0.165947	no
Nfkbib	26.1732	26.3374	0.00902291	0.95305	0.974859	no
Nfkbid	28.609	27.5268	-0.0556314	0.7142	0.825928	no
Nfkbie	17.4479	17.4044	-0.00360697	0.98225	0.98978	no
Nfkbil1	14.8606	14.6762	-0.0180146	0.9153	0.953532	no
Nfkbiz	38.0013	50.6638	0.414906	0.0046	0.0139281	yes
Nfrkb	21.582	22.7988	0.0791288	0.58455	0.72709	no
Nfs1	23.6861	24.2943	0.0365748	0.83645	0.906302	no
Nfu1	41.2958	42.6346	0.0460307	0.76955	0.86359	no
Nfx1	25.6166	26.4538	0.0463958	0.739	0.843196	no
Nfxl1	4.72602	4.67635	-0.0152423	0.93025	0.961894	no
Nfya	25.1327	22.4859	-0.160544	0.2672	0.414586	no
Nfyb	33.5903	30.5265	-0.137983	0.3488	0.506106	no
Nfyc	36.2079	31.4855	-0.201618	0.1755	0.303027	no
Ngdn	70.2943	72.9963	0.0544153	0.71195	0.824312	no
Ngfrap1	44.0735	38.3291	-0.201471	0.2119	0.351492	no
Ngly1	32.4502	37.2899	0.200559	0.1955	0.330217	no
Ngrn	20.7465	22.8315	0.138158	0.399	0.558043	no
Nhej1	5.04651	4.13309	-0.288067	0.29145	0.442447	no
Nhlrc2	10.7869	11.2649	0.0625509	0.6844	0.805052	no
Nhlrc3	8.41371	8.96482	0.0915325	0.63695	0.76917	no
Nhp2	59.0485	58.7384	-0.00759588	0.97605	0.986787	no
Nhp2l1	98.0941	91.927	-0.0936782	0.5113	0.664492	no
Nhsl2	3.77157	4.44665	0.237552	0.12625	0.23351	no
Nif3l1	10.5264	11.5061	0.12838	0.4454	0.603054	no
Nifk	23.235	23.7781	0.0333295	0.8238	0.898568	no
Nin	26.1228	27.5039	0.0743273	0.59765	0.737871	no
Ninj1	4.53123	4.76118	0.0714161	0.78215	0.872073	no
Nip7	12.6191	13.7654	0.125434	0.42355	0.581955	no
Nipa2	17.2432	16.319	-0.079475	0.6805	0.802095	no

Nipal1	1.15836	1.71675	0.567597	0.0181	0.0456948	yes
Nipal3	40.86	39.3692	-0.0536228	0.70775	0.821483	no
Nipbl	29.5696	30.1584	0.0284457	0.8435	0.910669	no
Nipsnap1	13.4012	15.8011	0.237665	0.15005	0.267344	no
Nipsnap3b	26.5188	23.8573	-0.152584	0.33805	0.49438	no
Nisch	41.8238	48.8251	0.223297	0.11245	0.212667	no
Nit1	24.6742	22.3287	-0.144104	0.5502	0.698193	no
Nit2	9.60297	11.8442	0.302627	0.1136	0.214463	no
Nkap	6.95032	6.72268	-0.0480425	0.76335	0.859475	no
Nkg7	1975.35	1724.96	-0.195545	0.1985	0.334252	no
Nkiras1	2.37821	2.37657	-0.000996044	0.99445	0.996356	no
Nkiras2	5.99049	5.50164	-0.122811	0.6498	0.779129	no
Nkrf	2.65539	3.21583	0.276267	0.17495	0.302233	no
Nktr	33.7929	33.2367	-0.0239445	0.86315	0.922654	no
Nle1	7.04597	8.566	0.281824	0.14045	0.253698	no
Nlk	15.5778	16.8574	0.113888	0.43515	0.593227	no
Nln	5.52993	5.88792	0.0904976	0.59745	0.737753	no
Nlrc3	45.6078	45.5135	-0.00298613	0.98395	0.990673	no
Nlrc5	94.6258	80.7377	-0.228991	0.10985	0.20879	no
Nlrp1a	1.80916	1.59533	-0.181467	0.3971	0.556202	no
Nlrx1	3.18159	4.2291	0.410604	0.02975	0.0701013	no
Nmb	27.8326	23.7672	-0.227803	0.23045	0.37394	no
Nmd3	30.7704	30.6524	-0.00554284	0.97	0.983728	no
Nme1	73.751	72.7832	-0.0190576	0.8979	0.94337	no
Nme2	457.294	529.485	0.211466	0.13945	0.252201	no
Nme3	40.1099	44.1838	0.139561	0.7499	0.850477	no
Nme6	2.64113	2.42752	-0.121672	0.71975	0.829654	no
Nme7	9.02951	8.03162	-0.168957	0.39595	0.554976	no
Nmi	57.3052	54.5789	-0.0703232	0.6327	0.766017	no
Nmnat1	5.19066	5.56045	0.099284	0.7019	0.817085	no
Nmnat2	1.26563	0.783952	-0.691016	0.00805	0.0226766	yes
Nmnat3	1.48022	1.69033	0.191491	0.4985	0.652797	no
Nmral1	10.4765	7.10161	-0.560941	0.00505	0.0151078	yes
Nmrk1	11.5436	13.1236	0.185077	0.29105	0.442011	no
Nmt1	62.2418	54.4231	-0.193665	0.1748	0.302062	no
Nmt2	9.21792	9.09241	-0.0197777	0.89855	0.943838	no
Nnt	6.64714	6.23802	-0.0916444	0.5881	0.730077	no
Noa1	13.9287	14.837	0.0911368	0.5729	0.717136	no
Nob1	26.1175	27.8529	0.0928113	0.5432	0.692323	no
Noc3l	7.44657	8.31268	0.158737	0.32315	0.478177	no
Noc4l	16.8405	18.3736	0.125695	0.43005	0.588343	no
Nod1	69.4521	53.1856	-0.384983	0.0062	0.0180576	yes
Nol10	11.2366	9.89999	-0.182709	0.2545	0.399163	no
Nol11	13.3309	14.1788	0.0889588	0.5588	0.70558	no
Nol12	14.7748	14.519	-0.0251966	0.87345	0.928895	no
Nol6	15.5282	18.1758	0.227125	0.1186	0.222108	no
Nol7	84.7494	77.4555	-0.129836	0.53005	0.68106	no
Nol8	12.4307	11.9368	-0.0584962	0.69915	0.815511	no
Nol9	15.3268	16.2078	0.0806353	0.597	0.737326	no
Nolc1	19.3771	22.1673	0.194082	0.18825	0.320657	no
Nom1	14.602	14.8269	0.022054	0.88565	0.936042	no
Nomo1	17.3449	19.6103	0.1771	0.2174	0.358274	no
Nono	203.025	202.71	-0.00223964	0.9879	0.992965	no
Nop10	71.7408	74.4794	0.0540474	0.7274	0.834859	no
Nop14	16.1773	17.6404	0.124916	0.4125	0.57131	no
Nop16	9.34702	11.8673	0.344408	0.05645	0.120577	no
Nop2	14.4698	15.9158	0.137413	0.38185	0.540917	no

Nop56	71.0316	67.4572	-0.0744904	0.7249	0.833142	no
Nop58	41.4489	47.707	0.202869	0.158	0.278778	no
Nop9	13.1043	13.401	0.0323002	0.8326	0.903961	no
Nosip	42.6179	38.9942	-0.1282	0.37015	0.528747	no
Notch1	24.3474	26.2293	0.107414	0.4466	0.604181	no
Notch2	13.6704	13.9444	0.0286239	0.8427	0.910112	no
Notch3	2.86493	0.71543	-2.00162	5.00E-05	0.000236281	yes
Nox1	1.63145	1.30764	-0.319191	0.6679	0.792797	no
Npat	6.01375	5.75757	-0.0628057	0.67875	0.800728	no
Npc1	14.135	16.2271	0.199133	0.32655	0.481865	no
Npc2	97.2639	96.3046	-0.0142996	0.9188	0.955641	no
Npdc1	0.99665	2.2688	1.18677	0.0019	0.00637506	yes
Npepl1	35.7453	37.2883	0.0609676	0.67295	0.796468	no
Npepps	13.1106	12.1026	-0.115415	0.45475	0.612471	no
Npff	3.11659	3.68585	0.242031	0.5844	0.726993	no
Nploc4	21.6047	20.8142	-0.0537722	0.7085	0.821976	no
Npm1	478.715	479.858	0.00343906	0.98105	0.989178	no
Npm3	179.455	152.847	-0.231536	0.10185	0.196206	no
Nprl2	26.4774	24.5681	-0.107975	0.4999	0.654145	no
Nprl3	9.44559	11.1573	0.240272	0.20685	0.344939	no
Nptn	109.798	132.182	0.267672	0.05905	0.125199	no
Nqo2	15.0104	14.987	-0.00225479	0.991	0.994494	no
Nr1d2	6.59802	6.67448	0.0166204	0.917	0.9545	no
Nr1h2	65.8666	60.9319	-0.112349	0.4405	0.598498	no
Nr1h3	1.10845	0.920236	-0.268472	0.45255	0.610102	no
Nr2c1	1.4086	1.81967	0.369409	0.11465	0.216075	no
Nr2c2	9.5254	8.97748	-0.0854692	0.69095	0.809693	no
Nr2c2ap	31.2024	34.291	0.136174	0.5232	0.674974	no
Nr2f6	3.82682	4.08863	0.0954708	0.65475	0.782976	no
Nr3c1	23.1688	19.1687	-0.273433	0.056	0.119757	no
Nr4a1	24.3045	24.69	0.0226997	0.8796	0.932569	no
Nr4a2	5.74913	6.25091	0.120722	0.49805	0.65239	no
Nr4a3	2.06863	1.73277	-0.255599	0.35745	0.515299	no
Nradd	12.0444	10.4996	-0.19802	0.30515	0.457965	no
Nrarp	8.02894	3.1399	-1.35449	5.00E-05	0.000236281	yes
Nras	33.553	31.4399	-0.0938488	0.51375	0.666645	no
Nrbf2	26.9807	23.2029	-0.217621	0.15685	0.277088	no
Nrbp1	76.0213	71.9489	-0.07943	0.82805	0.901199	no
Nrbp2	1.41407	1.90674	0.43126	0.1046	0.200469	no
Nrd1	40.2742	39.5376	-0.026628	0.89045	0.938644	no
Nrde2	9.65046	9.32611	-0.0493232	0.75405	0.8531	no
Nrf1	26.1354	24.706	-0.0811436	0.5903	0.731817	no
Nrg4	3.70654	3.53945	-0.0665482	0.8928	0.94004	no
Nrip1	8.76018	12.1418	0.470947	0.00225	0.00740543	yes
Nrm	37.7752	29.3509	-0.364032	0.0201	0.050067	no
Nrn1l	1.07128	0.342564	-1.64488	0.0872	0.172624	no
Nrp1	18.3766	27.3232	0.572262	5.00E-05	0.000236281	yes
Nrp2	2.30043	2.12123	-0.117004	0.5196	0.671705	no
Nrros	36.6453	38.2168	0.0605788	0.6619	0.788507	no
Nsa2	127.261	120.153	-0.0829161	0.5595	0.706035	no
Nsd1	19.7878	22.2683	0.170383	0.2326	0.376398	no
Nsdhl	12.7758	11.6019	-0.139052	0.39605	0.555039	no
Nsf	28.26	24.6051	-0.199807	0.1604	0.282173	no
Nsfl1c	54.8479	53.0549	-0.0479505	0.74225	0.845387	no
Nsg2	7.98696	14.0655	0.816439	5.00E-05	0.000236281	yes
Nsl1	4.01033	2.46713	-0.700888	0.00105	0.00375607	yes
Nsmaf	88.1508	86.5367	-0.0266603	0.8501	0.91448	no

Nsmce1	44.5597	40.2411	-0.147069	0.34775	0.504935	no
Nsmce2	26.0436	23.9471	-0.12108	0.467	0.623816	no
Nsmce4a	65.708	58.125	-0.17691	0.2198	0.36099	no
Nsmf	7.22237	6.34033	-0.187914	0.27655	0.425566	no
Nsun2	25.2756	27.6557	0.129828	0.3684	0.526962	no
Nsun3	6.63621	8.07202	0.28257	0.17985	0.309124	no
Nsun4	3.54441	3.75918	0.084871	0.65425	0.782736	no
Nsun5	6.95753	7.66754	0.14019	0.7433	0.84601	no
Nsun6	5.05868	5.63797	0.156414	0.3954	0.554503	no
Nt5c	129.636	137.248	0.0823227	0.7718	0.865207	no
Nt5c2	5.84345	5.74405	-0.0247508	0.88605	0.936215	no
Nt5c3	36.553	34.9704	-0.063857	0.6655	0.790993	no
Nt5c3b	68.6976	57.6036	-0.2541	0.0822	0.164298	no
Nt5dc1	21.3625	20.6326	-0.0501547	0.73585	0.841002	no
Nt5dc2	1.50587	0.824401	-0.86918	0.03335	0.0771723	no
Nt5dc3	3.41844	4.22376	0.30519	0.07325	0.14918	no
Nt5e	10.1856	11.9045	0.224981	0.15015	0.267482	no
Nt5m	12.5529	13.343	0.0880518	0.6299	0.763596	no
Ntan1	71.4356	70.2364	-0.0244245	0.89175	0.939411	no
Nthl1	1.63071	1.78114	0.127305	0.7332	0.838951	no
Ntmt1	9.58595	9.4826	-0.0156387	0.9438	0.969593	no
Ntng2	5.12001	5.86344	0.1956	0.6198	0.755739	no
Ntpcr	13.8014	13.3215	-0.0510596	0.7877	0.875895	no
Nub1	59.1826	53.1492	-0.155124	0.26945	0.417242	no
Nubp1	28.7569	25.1057	-0.195893	0.21775	0.358608	no
Nubp2	52.5865	52.7761	0.00519204	0.9698	0.983653	no
Nubpl	2.38088	2.79349	0.230572	0.45125	0.608721	no
Nucb1	85.6558	87.6023	0.0324165	0.81045	0.890336	no
Nucb2	1.81645	2.14239	0.238099	0.41595	0.574656	no
Nucks1	18.162	15.8574	-0.195768	0.1691	0.294222	no
Nudc	76.4615	80.0279	0.0657689	0.6445	0.775078	no
Nudcd1	4.9963	4.61702	-0.113898	0.65955	0.786663	no
Nudcd2	18.2049	18.2514	0.00368026	0.9818	0.989581	no
Nudcd3	27.2287	21.8135	-0.319908	0.02675	0.0641066	no
Nudt1	17.9299	13.9009	-0.367188	0.0554	0.11864	no
Nudt13	3.75477	4.00282	0.0922904	0.65815	0.785591	no
Nudt14	19.1527	19.2072	0.00410169	0.9774	0.987431	no
Nudt15	2.29414	1.88064	-0.286729	0.2447	0.389804	no
Nudt16	16.5154	16.4969	-0.00162087	0.99455	0.996371	no
Nudt16l1	60.9549	58.3635	-0.0626752	0.7034	0.818264	no
Nudt17	2.68482	2.51823	-0.092417	0.8835	0.934836	no
Nudt18	7.04954	7.01845	-0.00637682	0.9696	0.983591	no
Nudt19	17.3504	18.2932	0.0763369	0.63225	0.765669	no
Nudt2	5.53999	5.79906	0.0659366	0.8095	0.889777	no
Nudt21	108.687	100.15	-0.11802	0.4105	0.569445	no
Nudt22	10.2893	8.19134	-0.328978	0.1298	0.238737	no
Nudt3	43.1493	41.7068	-0.0490547	0.7335	0.839077	no
Nudt4	25.9361	18.6445	-0.476213	0.00115	0.00407292	yes
Nudt5	20.2946	19.68	-0.0443639	0.7849	0.87409	no
Nudt6	9.52246	9.92106	0.0591597	0.77585	0.867942	no
Nudt7	9.73744	11.2188	0.204305	0.37435	0.533021	no
Nudt8	8.22976	11.1503	0.438157	0.0637	0.133079	no
Nudt9	25.2148	26.5733	0.0757074	0.6327	0.766017	no
Nuf2	14.2401	7.76698	-0.874535	5.00E-05	0.000236281	yes
Nufip1	6.93654	7.43867	0.100829	0.53745	0.68749	no
Nufip2	10.7878	11.4424	0.0849881	0.55505	0.702214	no
Numa1	49.9895	50.8563	0.0248022	0.8631	0.922629	no

Numb	9.04139	8.39282	-0.107389	0.50025	0.654458	no
Numbl	2.74519	2.11176	-0.37846	0.0994	0.192357	no
Nup107	19.419	16.5575	-0.229979	0.1283	0.236527	no
Nup133	4.0706	3.18868	-0.352284	0.03825	0.0866614	no
Nup153	45.6794	45.8008	0.00382823	0.9796	0.988479	no
Nup155	5.94901	5.3887	-0.142713	0.36215	0.520374	no
Nup160	8.27152	7.78092	-0.0882112	0.5645	0.71016	no
Nup188	16.4453	16.3414	-0.0091463	0.9507	0.973541	no
Nup205	12.4009	11.9595	-0.0522876	0.7164	0.827374	no
Nup210	100.422	96.0002	-0.0649613	0.65065	0.779843	no
Nup214	13.4537	12.9552	-0.0544679	0.7036	0.818403	no
Nup35	8.81613	8.28321	-0.0899566	0.5939	0.734848	no
Nup37	14.0344	13.1372	-0.0953149	0.60115	0.740754	no
Nup43	6.9505	5.92761	-0.229666	0.29785	0.449802	no
Nup50	37.1052	29.5382	-0.329043	0.0203	0.050517	no
Nup54	19.3886	17.5793	-0.141327	0.35655	0.514316	no
Nup62	31.5555	25.1602	-0.326752	0.10375	0.199202	no
Nup85	25.9014	24.5378	-0.0780237	0.6042	0.743072	no
Nup88	39.8317	36.1469	-0.140047	0.42865	0.586894	no
Nup93	21.4185	18.5796	-0.205139	0.1779	0.306377	no
Nup98	15.2796	15.4271	0.0138552	0.93125	0.962491	no
Nupl1	27.1135	28.7816	0.0861318	0.55925	0.705908	no
Nupl2	2.59319	3.11226	0.263234	0.21665	0.35733	no
Nus1	20.7285	21.2194	0.033769	0.8113	0.890853	no
Nusap1	23.9552	13.0563	-0.875594	5.00E-05	0.000236281	yes
Nutf2	1.6977	2.02265	0.252662	0.68295	0.803802	no
Nvl	28.4847	33.9008	0.251133	0.0807	0.161767	no
Nxf1	95.0321	97.5483	0.0377008	0.7962	0.88132	no
Nxn	9.06139	8.69686	-0.0592375	0.72225	0.831409	no
Nxnl1	0.38733	1.19029	1.61968	3.00E-04	0.00122185	yes
Nxpe3	10.3882	10.6968	0.0422226	0.77635	0.868226	no
Nxt1	36.0516	26.3226	-0.45376	0.0066	0.0190676	yes
Nxt2	2.10482	1.58457	-0.409601	0.107	0.20432	no
Nyap1	3.59202	2.60116	-0.465639	0.02015	0.0501845	no
Oard1	41.7615	37.7657	-0.145098	0.3636	0.521852	no
Oas1a	4.37701	3.14895	-0.475075	0.0374	0.0850194	no
Oas1b	9.53723	9.11373	-0.0655273	0.71505	0.826464	no
Oas1c	10.3092	9.76642	-0.0780288	0.6437	0.774472	no
Oas3	17.6944	18.0499	0.0286971	0.8448	0.911458	no
Oat	20.1173	16.2409	-0.308804	0.04875	0.106446	no
Oaz1	232.663	203.806	-0.191048	0.4465	0.604103	no
Oaz2	15.5617	14.7641	-0.0759067	0.6475	0.777348	no
Obfc1	18.3123	17.8051	-0.0405165	0.8057	0.887327	no
Ocel1	11.7281	10.0217	-0.226839	0.20425	0.341531	no
Ociad1	141.068	148.868	0.0776467	0.58835	0.730259	no
Ocrl	1.90664	2.09952	0.139028	0.4801	0.635815	no
Odc1	80.743	85.0275	0.0745926	0.60335	0.742402	no
Odf2	39.3241	36.8718	-0.0928954	0.5266	0.677923	no
Odf2l	3.15324	2.73799	-0.203721	0.3683	0.526883	no
Ofd1	7.0686	7.29994	0.0464606	0.7743	0.866867	no
Ogdh	50.7673	53.9464	0.0876272	0.54155	0.690806	no
Ogfod1	8.34257	8.25207	-0.0157358	0.9146	0.953169	no
Ogfod2	14.2766	14.1887	-0.00890808	0.95575	0.976347	no
Ogfod3	1.01551	1.08269	0.0924163	0.93315	0.963496	no
Ogfr	60.1148	61.0572	0.0224397	0.87565	0.930296	no
Ogg1	7.61522	6.90808	-0.1406	0.4861	0.641355	no
Ogt	75.6535	78.9889	0.0622426	0.6579	0.785372	no

Oip5	3.34908	1.81197	-0.886202	0.1678	0.292509	no
Ola1	47.5639	45.2201	-0.0729021	0.65505	0.783215	no
Olfr613	7.76431	7.05699	-0.137804	0.37655	0.535376	no
Olfr99	1.61849	1.48198	-0.127129	0.7681	0.86258	no
Oma1	15.7138	16.5767	0.0771318	0.6415	0.772721	no
Omd	2.72719	3.042	0.157603	0.64015	0.771569	no
Opa1	14.6338	14.1762	-0.0458306	0.74875	0.849774	no
Opa3	10.3661	8.66844	-0.258027	0.1377	0.249978	no
Oplah	2.65818	2.05186	-0.373509	0.1478	0.263981	no
Orai1	13.6582	13.9722	0.0327863	0.84175	0.909559	no
Orai2	75.6975	81.6996	0.110083	0.4358	0.593907	no
Orai3	14.703	17.0052	0.209865	0.1951	0.32966	no
Oraov1	14.6081	13.0039	-0.167828	0.31105	0.464646	no
Orc1	1.76376	0.80786	-1.12647	0.00035	0.0014053	yes
Orc2	10.962	11.5595	0.0765714	0.61565	0.752538	no
Orc3	11.0609	10.9224	-0.0181737	0.92105	0.957064	no
Orc4	15.393	14.2356	-0.112776	0.52195	0.67399	no
Orc5	21.5364	19.9778	-0.108378	0.4841	0.639589	no
Orc6	19.4903	14.4806	-0.428642	0.00875	0.0243973	yes
Ormdl1	33.2962	30.1779	-0.141866	0.3476	0.504738	no
Ormdl2	24.4585	22.4002	-0.126823	0.63455	0.767414	no
Ormdl3	18.0293	17.1641	-0.0709556	0.6546	0.782916	no
Os9	81.0808	81.768	0.0121769	0.9347	0.964461	no
Osbp	22.2993	21.5103	-0.0519716	0.71765	0.828153	no
Osbpl11	11.5606	11.2303	-0.04182	0.7819	0.871904	no
Osbpl2	11.8762	11.1681	-0.0886869	0.5856	0.727883	no
Osbpl3	32.4969	21.1842	-0.617313	5.00E-05	0.000236281	yes
Osbpl5	30.9644	36.8147	0.24967	0.07975	0.160153	no
Osbpl7	15.4774	16.2901	0.0738257	0.62115	0.756758	no
Osbpl8	19.4626	17.4349	-0.158724	0.2568	0.401868	no
Osbpl9	54.3776	51.1778	-0.0874938	0.6679	0.792797	no
Oser1	53.8409	50.7921	-0.0840982	0.5645	0.71016	no
Osgep	21.2592	25.2134	0.246102	0.12105	0.225762	no
Osgepl1	4.25147	3.98636	-0.0928895	0.6747	0.79771	no
Osgin2	8.88471	9.32749	0.0701628	0.66795	0.792797	no
Osm	6.11123	6.90609	0.176406	0.36685	0.525266	no
Ost4	203.823	191.542	-0.0896526	0.5838	0.72649	no
Ostc	116.351	115.207	-0.0142559	0.92	0.956396	no
Ostf1	253.781	242.326	-0.0666315	0.639	0.770756	no
Ostm1	32.9791	27.9039	-0.241085	0.0953	0.185799	no
Otub1	51.0645	47.8653	-0.0933422	0.525	0.676591	no
Otub2	1.33746	1.55039	0.21313	0.41455	0.57335	no
Otud1	7.75934	8.09719	0.0614882	0.72665	0.834446	no
Otud3	7.69622	7.72429	0.00525213	0.9768	0.987093	no
Otud4	28.2256	28.938	0.0359598	0.80065	0.884125	no
Otud5	57.3835	57.7968	0.0103534	0.94085	0.967977	no
Otud6b	9.8921	9.52678	-0.0542874	0.73705	0.841815	no
Otud7b	6.1527	5.62289	-0.129909	0.39135	0.550431	no
Ovca2	10.7808	10.0587	-0.100015	0.61415	0.751212	no
Oxa1l	30.8431	28.3027	-0.124005	0.39525	0.55438	no
Oxct1	25.4852	27.4509	0.107198	0.45225	0.609756	no
Oxld1	6.20318	4.83571	-0.359279	0.2001	0.336209	no
Oxnad1	6.5757	6.65803	0.0179503	0.92095	0.957003	no
Oxr1	17.0075	17.2683	0.0219558	0.8837	0.934961	no
Oxsm	5.40753	5.55878	0.0397969	0.90885	0.949752	no
Oxsr1	16.3988	16.3382	-0.00534513	0.97185	0.984473	no
P2rx4	1.07151	1.36766	0.352064	0.27865	0.428098	no

P2ry10	33.1194	41.1953	0.314802	0.03015	0.0708499	no
P2ry12	5.21555	2.59186	-1.00883	0.1072	0.204624	no
P2ry14	4.14475	4.28447	0.0478307	0.94715	0.971428	no
P4ha1	9.56252	8.65459	-0.143926	0.35165	0.509017	no
P4hb	119.312	117.423	-0.0230308	0.87265	0.928534	no
P4htm	2.59621	3.28159	0.337989	0.17245	0.298872	no
Pa2g4	55.6003	52.3048	-0.0881492	0.5381	0.687959	no
Pabpc1	672.226	586.585	-0.196606	0.2206	0.361951	no
Pabpc4	25.6577	27.0973	0.0787534	0.5956	0.736112	no
Pabpn1	91.4618	91.5037	0.000660614	0.9952	0.996768	no
Pacrgl	4.83562	4.946	0.0325614	0.88535	0.935946	no
Pacs1	50.6053	51.3114	0.0199919	0.889	0.937844	no
Pacs2	17.8091	20.4	0.195957	0.1713	0.297339	no
Pacsin1	1.3023	1.26112	-0.0463599	0.84595	0.911947	no
Pacsin2	25.5957	23.8469	-0.102101	0.48495	0.640268	no
Padi2	40.3833	37.9338	-0.0902736	0.5306	0.681469	no
Paf1	42.2012	43.1538	0.032203	0.82375	0.898541	no
Pafah1b1	56.7044	53.6705	-0.0793318	0.5776	0.721101	no
Pafah1b2	19.2321	18.6876	-0.0414351	0.78105	0.871246	no
Pafah1b3	7.02671	7.9364	0.175635	0.47375	0.630149	no
Pag1	21.4601	25.5223	0.2501	0.0752	0.152509	no
Pagr1a	48.6868	47.6869	-0.0299378	0.84125	0.909214	no
Paics	65.8894	62.0236	-0.087231	0.5766	0.720362	no
Paip1	9.09396	8.92167	-0.0275946	0.8582	0.919829	no
Paip2	129.502	128.901	-0.00671779	0.9634	0.980274	no
Paip2b	14.4091	14.6479	0.0237089	0.87605	0.930497	no
Pak1ip1	45.5593	44.5036	-0.0338219	0.81855	0.895291	no
Pak2	63.7338	65.1768	0.0322996	0.81975	0.896145	no
Pak4	3.77926	3.70406	-0.0289966	0.888	0.937209	no
Pak6	1.31519	1.59583	0.279037	0.249	0.394375	no
Palb2	2.14319	1.51206	-0.503249	0.03475	0.0798699	no
Pald1	2.76107	2.49148	-0.148224	0.44525	0.602932	no
Palm	16.9213	23.4707	0.472025	0.0024	0.00784462	yes
Palm3	4.41521	5.29681	0.262642	0.70945	0.822649	no
Pam16	42.964	42.1001	-0.0293064	0.87635	0.930717	no
Pan2	15.9455	15.6256	-0.0292377	0.8444	0.911306	no
Pan3	32.7924	34.7234	0.0825463	0.5614	0.707547	no
Pank2	20.249	18.5193	-0.128821	0.39725	0.556368	no
Pank3	8.88673	9.44527	0.0879386	0.5529	0.700474	no
Pank4	17.3731	18.4505	0.0868105	0.5775	0.721078	no
Panx1	46.1489	47.8219	0.0513757	0.72195	0.831132	no
Paox	8.2999	8.47143	0.029512	0.8751	0.929879	no
Papd4	37.5468	33.8918	-0.147756	0.32215	0.477068	no
Papd5	24.4425	22.9122	-0.093273	0.5196	0.671705	no
Papd7	6.5822	5.89164	-0.159899	0.331	0.486681	no
Papola	64.083	62.9404	-0.0259546	0.85285	0.91646	no
Papolg	12.2705	12.4424	0.0200745	0.89355	0.940507	no
Papss1	13.3009	13.0385	-0.0287531	0.85715	0.919206	no
Paqr4	9.29349	9.51638	0.0341926	0.8849	0.935616	no
Paqr7	11.1184	10.1409	-0.132758	0.38995	0.549063	no
Pard6a	6.97033	5.24837	-0.409358	0.0726	0.14825	no
Pard6b	0.734078	1.01785	0.471525	0.1157	0.217763	no
Parg	20.6795	20.3303	-0.0245679	0.8649	0.923867	no
Park7	172.401	161.899	-0.0906747	0.52845	0.679635	no
Parl	32.8326	32.6696	-0.0071782	0.96615	0.981559	no
Parm1	1.39422	0.353509	-1.97964	0.00025	0.00103712	yes
Parn	15.8583	16.2574	0.0358562	0.8127	0.891697	no

Parp1	33.4023	33.5023	0.00431343	0.97635	0.986921	no
Parp10	32.0968	32.5204	0.0189147	0.89295	0.9401	no
Parp11	4.8399	4.64216	-0.0601809	0.7267	0.834477	no
Parp12	2.29229	2.61196	0.188338	0.3817	0.540759	no
Parp14	25.1629	23.5697	-0.0943639	0.50555	0.659521	no
Parp16	3.88212	3.89881	0.00618894	0.97385	0.985692	no
Parp2	25.7678	26.3786	0.0338011	0.8309	0.902978	no
Parp3	24.0979	25.2142	0.0653245	0.65975	0.786848	no
Parp4	21.9593	23.3274	0.087192	0.5399	0.689474	no
Parp6	15.2516	18.4823	0.277179	0.07365	0.149869	no
Parp8	13.2871	21.4292	0.689548	5.00E-05	0.000236281	yes
Parp9	25.4778	25.3773	-0.00570336	0.9788	0.98811	no
Parpbp	4.39498	2.18253	-1.00986	5.00E-05	0.000236281	yes
Pars2	2.19162	1.94437	-0.172693	0.49615	0.650722	no
Parvg	71.3595	79.9163	0.163384	0.24775	0.392977	no
Pask	1.60234	0.832801	-0.944134	1.00E-04	0.000450026	yes
Patl1	18.5963	17.7754	-0.0651334	0.65445	0.782802	no
Patz1	10.9833	12.8861	0.230506	0.1429	0.256933	no
Paxbp1	24.264	24.0504	-0.012759	0.9499	0.973004	no
Paxip1	13.5535	12.3657	-0.132322	0.3691	0.527707	no
Pbdc1	13.6047	12.3248	-0.142549	0.4164	0.575018	no
Pbk	8.43719	4.04528	-1.06052	5.00E-05	0.000236281	yes
Pbrm1	26.4667	25.5765	-0.0493561	0.72495	0.833185	no
Pbx2	18.7761	16.4307	-0.1925	0.2038	0.340948	no
Pbx4	1.83469	1.81945	-0.0120337	0.97635	0.986921	no
Pbxip1	55.2382	69.8461	0.338513	0.01665	0.0425054	yes
Pcbd2	49.8263	41.1387	-0.27641	0.1292	0.23782	no
Pcbp1	141.569	137.668	-0.0403132	0.7748	0.867207	no
Pcbp2	137.773	128.941	-0.0955817	0.5383	0.688066	no
Pcbp4	7.16245	6.87981	-0.0580837	0.75535	0.853982	no
Pcca	5.33	6.85289	0.362576	0.0452	0.0998906	no
Pccb	7.60027	10.7839	0.504761	0.0044	0.0133909	yes
Pcdhgc3	6.46285	5.97638	-0.112899	0.6398	0.771331	no
Pcdhgc4	1.43339	0.584887	-1.2932	0.0622	0.130614	no
Pced1a	12.4122	12.4674	0.00640969	0.98265	0.990014	no
Pced1b	42.4495	35.3832	-0.262685	0.0726	0.14825	no
Pcf11	32.3324	34.3217	0.0861399	0.54115	0.690446	no
Pcgf1	20.4792	20.9109	0.0300954	0.87055	0.927138	no
Pcgf2	5.21669	5.6779	0.122223	0.5192	0.671446	no
Pcgf3	5.4811	5.71357	0.0599253	0.7291	0.835927	no
Pcgf5	11.1342	11.9258	0.0990938	0.58555	0.727859	no
Pcgf6	7.50647	7.88097	0.0702399	0.7033	0.818216	no
Pcid2	16.9917	18.4797	0.121107	0.4382	0.596188	no
Pcif1	40.9479	38.7767	-0.0785981	0.58875	0.730589	no
Pck2	1.51919	1.4573	-0.0600085	0.81135	0.890853	no
Pcm1	10.415	11.4083	0.131415	0.36015	0.518175	no
Pcmt1	29.6932	29.5114	-0.00885695	0.9518	0.974159	no
Pcmtd1	14.7422	16.5369	0.165734	0.2517	0.396819	no
Pcmtd2	27.4589	27.5612	0.00536474	0.9718	0.98445	no
Pcna	272.973	214.446	-0.348143	0.01335	0.0351488	yes
Pcnp	74.6246	66.7285	-0.161348	0.2541	0.398677	no
Pcnt	11.9793	11.5247	-0.0558198	0.69765	0.814489	no
Pcnx	2.50791	2.81083	0.164511	0.3028	0.455445	no
Pcnxl3	27.8452	28.145	0.0154511	0.91225	0.9519	no
Pcnxl4	1.72873	2.12602	0.298442	0.15525	0.274798	no
Pcsk7	24.2168	24.0783	-0.00827171	0.9607	0.978896	no
Pcyox1	2.28284	2.56111	0.165938	0.40235	0.561294	no

Pcyox1l	3.48398	3.90506	0.164608	0.46355	0.620617	no
Pcyt1a	7.45294	6.7968	-0.132953	0.4015	0.560496	no
Pcyt2	26.5563	29.2902	0.14136	0.35345	0.511056	no
Pdap1	42.105	40.0457	-0.0723453	0.6185	0.754728	no
Pdcd1	10.429	14.5563	0.481043	0.0057	0.016787	yes
Pdcd10	70.6815	62.7253	-0.172286	0.2276	0.370516	no
Pdcd11	11.7038	13.6172	0.218455	0.137	0.249239	no
Pdcd2	36.8463	38.1892	0.0516457	0.7395	0.843398	no
Pdcd2l	13.0539	12.4193	-0.0718973	0.6941	0.811888	no
Pdcd4	155.246	145.897	-0.0896033	0.59625	0.736683	no
Pdcd5	86.5342	82.3183	-0.0720585	0.64265	0.773631	no
Pdcd6	68.6927	69.9226	0.0256014	0.8594	0.920318	no
Pdcd6ip	79.4532	82.0016	0.0455467	0.7528	0.852246	no
Pdcd7	12.1023	11.3393	-0.0939615	0.5728	0.717062	no
Pdcl	14.6146	13.0666	-0.16153	0.2992	0.451348	no
Pdcl3	33.5277	31.5142	-0.0893546	0.56125	0.707421	no
Pddc1	36.3374	35.9393	-0.0158939	0.9155	0.953642	no
Pde12	13.3947	13.0853	-0.0337221	0.82515	0.899457	no
Pde1b	12.1016	9.13677	-0.405444	0.0121	0.0323073	yes
Pde2a	29.7944	17.6464	-0.755672	5.00E-05	0.000236281	yes
Pde3b	25.3655	24.8806	-0.0278456	0.8505	0.914715	no
Pde4a	2.78021	2.19736	-0.339427	0.0849	0.168834	no
Pde4b	21.5923	24.285	0.169549	0.2488	0.394182	no
Pde4d	5.45234	5.96178	0.128867	0.3982	0.557234	no
Pde4dip	9.08975	10.5258	0.211611	0.1526	0.271025	no
Pde6d	19.07	16.5398	-0.205366	0.25495	0.399673	no
Pde7a	41.5124	42.2709	0.0261238	0.8552	0.917923	no
Pde8a	4.90989	4.31024	-0.187922	0.32785	0.483419	no
Pdf	15.3523	12.5806	-0.287254	0.10185	0.196206	no
Pdgfb	6.08717	4.048	-0.588561	0.0019	0.00637506	yes
Pdha1	55.3042	53.8802	-0.0376324	0.8005	0.884028	no
Pdhb	71.8743	66.137	-0.120019	0.40555	0.564553	no
Pdhx	6.7351	6.81798	0.0176453	0.92225	0.957832	no
Pdia3	202.9	237.597	0.227747	0.1139	0.214949	no
Pdia4	51.2994	54.5515	0.0886764	0.52715	0.678445	no
Pdia6	61.3268	68.8149	0.166204	0.24505	0.390123	no
Pdik1l	4.42694	4.17559	-0.0843318	0.6188	0.754963	no
Pdk1	6.06158	4.89853	-0.307346	0.05655	0.120762	no
Pdk2	1.8049	2.15082	0.252971	0.332	0.487664	no
Pdk3	20.5412	17.7647	-0.20951	0.182	0.312105	no
Pdlim1	70.719	83.9609	0.247619	0.087	0.172315	no
Pdlim2	65.3304	60.2458	-0.116895	0.4193	0.577825	no
Pdlim5	17.2941	18.1846	0.0724369	0.63505	0.767782	no
Pdlim7	5.81398	6.07539	0.0634511	0.7786	0.869667	no
Pdp1	2.35763	2.19185	-0.10519	0.59985	0.739681	no
Pdp2	2.96621	2.98393	0.00859387	0.9627	0.980023	no
Pdpk1	23.2396	22.8964	-0.0214651	0.87985	0.932638	no
Pdpr	8.91585	9.23902	0.0513672	0.76815	0.862623	no
Pdrg1	64.1764	66.4096	0.04935	0.7298	0.836444	no
Pds5a	49.9109	50.0737	0.00469849	0.9746	0.986135	no
Pds5b	13.7436	13.1839	-0.0599876	0.67435	0.797483	no
Pdss1	4.31939	2.89561	-0.576962	0.61025	0.74788	no
Pdss2	2.83214	2.97618	0.0715677	0.7794	0.870285	no
Pdxdc1	11.433	11.6727	0.0299266	0.901	0.945163	no
Pdxk	4.90415	5.81335	0.245366	0.1344	0.245503	no
Pdxp	11.5811	11.2424	-0.0428212	0.79855	0.882786	no
Pdzd11	25.494	25.2351	-0.0147229	0.932	0.962815	no

Pdzd8	10.5613	13.8627	0.392424	0.00645	0.0186894	yes
Pdzk1	2.61276	2.44142	-0.0978557	0.666	0.79138	no
Pea15a	63.0064	59.0481	-0.0936082	0.51155	0.664695	no
Peak1	19.9088	16.8811	-0.237993	0.08865	0.175058	no
Pear1	13.0321	14.8928	0.192547	0.20115	0.337523	no
Pebp1	92.3745	119.124	0.366898	0.01085	0.0294352	yes
Pecam1	10.8123	19.9989	0.887247	5.00E-05	0.000236281	yes
Pef1	27.8324	27.8563	0.00123856	0.9915	0.994869	no
Peli1	37.2487	39.4171	0.0816319	0.5703	0.714921	no
Pelo	18.3171	19.9426	0.122664	0.6926	0.810886	no
Pelp1	9.53896	10.2418	0.10256	0.51935	0.671554	no
Peo1	3.84278	4.87344	0.342789	0.06295	0.131918	no
Pepd	29.4778	27.8713	-0.0808495	0.6008	0.740426	no
Per1	10.2807	11.1582	0.118163	0.4311	0.58938	no
Perp	2.77031	1.98981	-0.477411	0.07185	0.146982	no
Pes1	26.2402	29.0238	0.145457	0.3136	0.467328	no
Pet100	16.7963	15.4374	-0.121715	0.4968	0.651333	no
Pet112	3.92159	3.70936	-0.0802711	0.70615	0.820368	no
Pet117	6.65217	8.77726	0.399945	0.7604	0.857521	no
Pex1	2.78971	3.13569	0.168668	0.5115	0.664679	no
Pex10	3.08902	3.36946	0.125369	0.61315	0.75034	no
Pex11b	18.9322	21.9051	0.210419	0.2835	0.433605	no
Pex11g	2.20398	3.08294	0.484195	0.12385	0.229896	no
Pex12	3.96292	4.20614	0.0859332	0.6868	0.806665	no
Pex13	6.1708	5.74066	-0.104242	0.6475	0.777348	no
Pex14	11.6629	11.1256	-0.0680462	0.69185	0.810385	no
Pex16	19.3208	19.0921	-0.0171787	0.92085	0.956941	no
Pex19	16.0975	16.1384	0.00366735	0.97995	0.988614	no
Pex2	15.7002	16.8134	0.0988286	0.5324	0.682923	no
Pex26	8.9739	15.0544	0.746381	5.00E-05	0.000236281	yes
Pex3	6.36783	8.19077	0.363198	0.0851	0.169169	no
Pex5	19.1746	20.8342	0.119761	0.4197	0.578207	no
Pex6	17.8175	18.2094	0.0313862	0.831	0.903058	no
Pex7	11.2958	11.6833	0.0486634	0.7792	0.870158	no
Pfas	3.22634	3.07241	-0.0705284	0.67955	0.801444	no
Pfdn1	42.6317	41.4561	-0.0403416	0.79835	0.882662	no
Pfdn2	64.3706	69.118	0.102659	0.53465	0.685038	no
Pfdn4	23.7128	20.8891	-0.182914	0.31525	0.469034	no
Pfdn5	418.742	403.19	-0.0546006	0.699	0.815377	no
Pfkfb1	2.03867	2.26472	0.151706	0.58365	0.726393	no
Pfkfb2	3.83755	4.51324	0.233979	0.22105	0.362562	no
Pfkfb3	15.4805	17.0301	0.13763	0.3512	0.508543	no
Pfkl	11.2619	11.2588	-0.000397636	0.99755	0.998087	no
Pfkm	2.26701	1.37428	-0.722112	0.23205	0.375698	no
Pfkp	91.4322	72.9224	-0.32634	0.0351	0.080572	no
Pfn1	2803.06	2595.03	-0.11125	0.496	0.650622	no
Pgam1	81.8407	84.635	0.0484366	0.73285	0.838741	no
Pgam2	2.76973	3.28002	0.243959	0.45315	0.610677	no
Pgam5	27.382	26.4511	-0.0499004	0.7408	0.844294	no
Pgap1	1.53964	1.91316	0.313366	0.0744	0.151143	no
Pgap2	17.2269	14.0778	-0.291241	0.07805	0.157357	no
Pgap3	7.02589	9.16689	0.383752	0.0315	0.0735497	no
Pgd	37.3447	40.7173	0.124741	0.3926	0.551749	no
Pggt1b	8.36546	9.2734	0.148654	0.37405	0.53269	no
Pgk1	145.896	132.69	-0.136885	0.33685	0.493054	no
Pgls	75.821	84.1265	0.149964	0.32225	0.477176	no
Pglyrp1	170.648	190.503	0.158793	0.26775	0.415184	no

Pglyrp2	45.9795	49.338	0.101709	0.47515	0.631452	no
Pgm1	23.0248	24.4591	0.0871832	0.5672	0.712325	no
Pgm2	13.6026	14.8602	0.127574	0.4158	0.574527	no
Pgm2l1	9.53738	7.01038	-0.444101	0.00235	0.00770045	yes
Pgm3	4.92717	5.1358	0.0598298	0.8295	0.902081	no
Pgp	22.1507	19.6568	-0.17232	0.33025	0.485923	no
Pgrmc1	42.9392	40.2029	-0.0949953	0.5196	0.671705	no
Pgrmc2	11.195	11.1586	-0.00469539	0.97835	0.987896	no
Pgs1	20.1487	22.1721	0.13806	0.3627	0.520941	no
Phactr4	6.32474	5.35978	-0.238833	0.14345	0.257725	no
Phax	33.0763	28.9948	-0.190004	0.237	0.381629	no
Phb	32.4544	35.5388	0.130979	0.3864	0.545458	no
Phb2	131.542	133.356	0.019764	0.8854	0.935946	no
Phc1	10.4499	8.53459	-0.292092	0.06415	0.133829	no
Phc2	39.6687	41.3754	0.0607721	0.6726	0.796214	no
Phc3	14.0307	15.0076	0.0971075	0.49765	0.652059	no
Phf1	55.0079	52.2627	-0.0738578	0.60655	0.744885	no
Phf10	17.2485	18.4333	0.0958439	0.68065	0.802218	no
Phf11a	28.1464	19.6374	-0.519345	0.00135	0.00470245	yes
Phf11b	107.547	90.8306	-0.243722	0.09085	0.178643	no
Phf11c	19.0843	19.9696	0.0654157	0.688	0.80759	no
Phf12	31.6979	32.8015	0.0493773	0.73	0.836592	no
Phf13	17.5052	17.7581	0.0206968	0.8903	0.938598	no
Phf14	29.9617	29.5175	-0.0215462	0.88205	0.934016	no
Phf19	4.76713	3.72132	-0.357304	0.04785	0.104766	no
Phf2	24.7608	25.2571	0.028633	0.84235	0.909915	no
Phf20	7.04063	7.31853	0.0558482	0.72025	0.830041	no
Phf20l1	44.9984	40.5088	-0.151638	0.2853	0.435584	no
Phf21a	26.3172	27.5834	0.0677926	0.6329	0.766127	no
Phf21b	5.9247	4.93205	-0.264557	0.12755	0.235433	no
Phf23	33.1887	31.8136	-0.0610485	0.6783	0.800478	no
Phf3	19.6046	21.0212	0.100655	0.47855	0.634525	no
Phf5a	42.8239	35.5447	-0.268784	0.07225	0.147659	no
Phf6	13.6603	13.528	-0.0140387	0.92425	0.958705	no
Phf7	6.91207	6.41034	-0.108718	0.5691	0.713886	no
Phf8	15.8424	17.1191	0.111812	0.4465	0.604103	no
Phgdh	41.3354	39.8806	-0.0516901	0.72245	0.831572	no
Phip	11.1513	12.0158	0.107724	0.4491	0.606517	no
Phka2	5.29048	4.68584	-0.175088	0.30815	0.461398	no
Phkb	7.95932	8.7693	0.139817	0.364	0.522277	no
Phkg2	23.0914	23.0022	-0.00558821	0.9779	0.987653	no
Phlda1	18.9768	21.2503	0.163246	0.29095	0.441925	no
Phldb3	5.70166	8.28685	0.539442	0.0038	0.0117554	yes
Phlpp1	1.54565	1.68997	0.128783	0.52355	0.675351	no
Phlpp2	3.4708	3.47187	0.000445158	0.9958	0.997114	no
Phospho2	13.1572	14.1487	0.104822	0.5273	0.678601	no
Phpt1	34.5387	42.4771	0.298471	0.1045	0.200332	no
Phrf1	25.6521	25.8315	0.0100528	0.9436	0.969464	no
Phtf1	31.4964	30.1489	-0.0630829	0.73695	0.841741	no
Phtf2	2.07019	2.49776	0.270873	0.16525	0.28907	no
Phyh	19.3098	21.5176	0.156183	0.33855	0.494937	no
Phyhd1	12.0005	12.6005	0.0703908	0.7055	0.819802	no
Phykpl	6.51762	5.17429	-0.332984	0.69375	0.811681	no
Pi4k2a	12.5951	13.3589	0.0849433	0.57755	0.721101	no
Pi4k2b	2.05137	1.87494	-0.129742	0.5793	0.722648	no
Pi4ka	28.7431	26.6429	-0.109463	0.50425	0.658275	no
Pi4kb	23.6145	21.6947	-0.122326	0.471	0.627496	no

Pias1	13.1205	12.0025	-0.128492	0.3911	0.550167	no
Pias2	11.3805	11.8254	0.0553212	0.76445	0.860206	no
Pias3	14.5338	14.5308	-0.000302986	0.99815	0.998504	no
Pias4	21.2429	20.2193	-0.0712459	0.65855	0.785895	no
Pibf1	4.77575	4.52888	-0.0765747	0.76165	0.858232	no
Picalm	129.203	120.383	-0.101997	0.4776	0.633694	no
Pick1	6.67633	8.98809	0.428959	0.0211	0.0522701	no
Piezo1	28.9434	29.18	0.0117482	0.9398	0.967432	no
Pif1	4.28061	2.85026	-0.586721	0.0022	0.00725609	yes
Piga	6.95636	7.60934	0.129438	0.4331	0.591335	no
Pigb	6.93659	6.64215	-0.0625764	0.88815	0.937284	no
Pigc	7.25288	6.04015	-0.263969	0.12085	0.225484	no
Pigf	12.031	15.3927	0.355482	0.1727	0.299232	no
Pigg	1.46043	1.50065	0.0391936	0.87405	0.929309	no
Pigh	7.86215	8.16022	0.0536838	0.76305	0.859247	no
Pigk	10.8515	10.7613	-0.0120422	0.93435	0.964382	no
Pigl	11.496	10.4392	-0.139113	0.42985	0.588138	no
Pigm	11.2604	10.9393	-0.041738	0.7714	0.86491	no
Pign	4.24133	4.12781	-0.03914	0.8043	0.886588	no
Pigo	13.0542	12.5561	-0.0561269	0.70795	0.821648	no
Pigp	23.0393	24.6308	0.0963635	0.7631	0.859289	no
Pigq	24.3491	22.9962	-0.082477	0.56255	0.708503	no
Pigs	48.342	48.3404	-5.01E-05	0.999	0.999127	no
Pigt	54.4727	51.418	-0.0832591	0.55775	0.704569	no
Pigu	15.953	16.1004	0.0132635	0.93685	0.965734	no
Pigv	11.8546	11.3825	-0.058629	0.7228	0.831757	no
Pigw	1.95504	2.19105	0.164422	0.6829	0.803769	no
Pigx	40.5803	47.0852	0.214496	0.17775	0.306171	no
Pigyl	19.4939	18.6809	-0.0614559	0.76265	0.858961	no
Pih1d1	40.2018	30.3207	-0.406955	0.06235	0.13089	no
Pik3ap1	39.2051	26.7997	-0.548821	0.00015	0.000653255	yes
Pik3c2a	4.47932	4.90075	0.129722	0.40395	0.562914	no
Pik3c3	25.737	23.2078	-0.149234	0.3085	0.461765	no
Pik3ca	13.385	13.5054	0.0129169	0.9321	0.962876	no
Pik3cb	1.12196	1.08032	-0.0545744	0.82125	0.897021	no
Pik3cd	175.744	192.655	0.132548	0.37105	0.529625	no
Pik3cg	20.9583	19.6698	-0.0915351	0.5177	0.670194	no
Pik3ip1	19.3454	41.811	1.11189	5.00E-05	0.000236281	yes
Pik3r1	33.6972	32.9423	-0.0326842	0.81585	0.893487	no
Pik3r2	2.08945	2.64685	0.341154	0.12465	0.231114	no
Pik3r4	12.8187	13.7362	0.0997372	0.49505	0.649738	no
Pik3r5	99.6588	115.795	0.2165	0.13315	0.243673	no
Pikfyve	3.39469	4.35642	0.359863	0.01985	0.0495212	yes
Pim1	184.946	234.622	0.343235	0.0162	0.0415068	yes
Pim2	38.9578	59.4826	0.610557	5.00E-05	0.000236281	yes
Pim3	19.4427	19.9145	0.0345901	0.81865	0.895331	no
Pin1	9.22067	9.37097	0.0233268	0.87705	0.931124	no
Pin4	89.9469	97.2116	0.112055	0.5006	0.654746	no
Pink1	45.4567	38.9326	-0.223517	0.1176	0.220627	no
Pinx1	10.9902	12.7007	0.208694	0.25945	0.405081	no
Pip4k2a	35.191	39.3181	0.159988	0.26525	0.412131	no
Pip4k2b	27.0913	29.2225	0.109251	0.4392	0.597226	no
Pip4k2c	33.0839	32.111	-0.043062	0.8127	0.891697	no
Pip5k1a	22.904	23.9159	0.0623732	0.67605	0.798824	no
Pip5k1c	32.6517	34.0638	0.0610809	0.66805	0.792889	no
Pisd	17.3748	17.9488	0.0468857	0.76735	0.862081	no
Pithd1	33.7313	35.2034	0.0616284	0.6845	0.805134	no

Pitpna	68.3791	63.2813	-0.111776	0.427	0.585407	no
Pitpnb	30.971	28.9083	-0.0994301	0.49495	0.649619	no
Pitpnc1	44.6943	44.469	-0.00728855	0.9608	0.978922	no
Pitpnm1	68.1967	71.4519	0.0672705	0.6996	0.815699	no
Pitpnm2	7.62176	7.20455	-0.0812152	0.59375	0.734714	no
Pitrm1	13.8803	16.0832	0.212514	0.5695	0.71421	no
Pja1	18.0171	18.1512	0.0106974	0.94575	0.970556	no
Pja2	6.732	6.33499	-0.0876923	0.5862	0.728359	no
Pkd1	9.37344	10.3832	0.147598	0.4222	0.58062	no
Pkd2l2	2.63896	2.82649	0.099044	0.8977	0.943244	no
Pkig	3.0267	3.28044	0.116145	0.69845	0.814978	no
Pkm	219.267	220.292	0.00672567	0.96225	0.979811	no
Pkmyt1	15.779	13.5116	-0.223799	0.2815	0.43134	no
Pkn1	81.8649	89.973	0.136248	0.4175	0.576109	no
Pkn2	12.6076	13.1197	0.0574392	0.68185	0.803016	no
Pknox1	22.17	20.4773	-0.114585	0.4212	0.579628	no
Pkp3	35.1482	43.1788	0.296873	0.0399	0.0898546	no
Pkp4	7.69205	8.36091	0.120292	0.44235	0.600226	no
Pla2g12a	5.28315	5.50906	0.0604072	0.7786	0.869667	no
Pla2g15	3.47939	3.18811	-0.126134	0.5456	0.694153	no
Pla2g16	22.1058	20.2934	-0.123415	0.40105	0.560067	no
Pla2g6	3.15526	3.44044	0.124837	0.5225	0.674391	no
Plaa	16.1122	15.2765	-0.0768346	0.59715	0.737447	no
Plac8	347.606	420.964	0.276245	0.05315	0.114464	no
Plagl2	11.0838	10.3736	-0.0955353	0.5232	0.674974	no
Plbd2	10.4259	11.1442	0.096115	0.53175	0.682412	no
Plcb2	32.4003	33.0325	0.0278789	0.8394	0.907951	no
Plcb3	3.63909	3.33642	-0.125277	0.49555	0.650165	no
Plcd1	2.2917	2.40392	0.0689725	0.77425	0.866852	no
Plcg1	88.5356	99.0806	0.162346	0.2509	0.396574	no
Plcg2	5.14687	4.77625	-0.107818	0.5276	0.678888	no
Plcl2	30.2566	29.3966	-0.0416028	0.7705	0.864285	no
Plcxd1	1.10457	1.04858	-0.0750498	0.8852	0.935836	no
Plcxd2	10.7495	12.9227	0.265634	0.06395	0.133491	no
Pld2	5.22908	3.634	-0.525001	0.0035	0.010941	yes
Pld3	105.193	97.1798	-0.114314	0.42125	0.579663	no
Pld4	3.03245	1.24504	-1.28429	5.00E-05	0.000236281	yes
Plec	50.211	53.0193	0.078514	0.597	0.737326	no
Plek	136.871	108.022	-0.341503	0.0169	0.0430564	yes
Plekha1	6.24445	8.66598	0.472789	0.00605	0.0176784	yes
Plekha2	36.7612	40.6933	0.146605	0.302	0.454581	no
Plekha3	18.4578	18.2961	-0.0126952	0.93255	0.963228	no
Plekha5	11.7735	12.6275	0.10103	0.4976	0.65203	no
Plekha6	1.76395	1.94173	0.138537	0.45625	0.613777	no
Plekhb2	45.9356	42.9275	-0.0977113	0.49405	0.648751	no
Plekhf1	7.30029	4.13288	-0.820808	0.00025	0.00103712	yes
Plekhf2	15.9764	13.6175	-0.230481	0.13545	0.246948	no
Plekhg2	24.119	25.0981	0.0574059	0.6823	0.803398	no
Plekhg3	9.29034	8.45631	-0.135703	0.37345	0.532083	no
Plekhg5	4.08482	5.34869	0.388913	0.0287	0.0680223	no
Plekhj1	61.8436	56.8464	-0.121554	0.40995	0.568872	no
Plekhm1	15.441	15.0472	-0.0372707	0.798	0.882386	no
Plekhm2	10.5937	10.9611	0.049184	0.7521	0.851891	no
Plekhm3	2.30428	3.33443	0.533126	0.00175	0.00592472	yes
Plekhn1	6.16479	7.20231	0.224409	0.22425	0.366593	no
Plekho1	15.9739	8.27001	-0.949754	5.00E-05	0.000236281	yes
Plekho2	32.8234	31.2252	-0.0720172	0.60895	0.746776	no

Plgrkt	91.3862	73.4836	-0.314554	0.03795	0.0860922	no
Plin2	6.26357	5.35075	-0.227242	0.25125	0.396819	no
Plin3	13.7782	16.7329	0.280296	0.0822	0.164298	no
Plk1	24.5063	14.4723	-0.759854	5.00E-05	0.000236281	yes
Plk3	13.9036	13.9118	0.000851858	0.99635	0.997367	no
Plk4	8.53307	5.89543	-0.533468	0.0016	0.00547227	yes
Plod1	5.36929	5.62185	0.0663145	0.7094	0.822641	no
Plod2	0.887723	1.14957	0.372907	0.1699	0.295337	no
Plod3	4.16152	4.77948	0.199743	0.38765	0.546839	no
Plp2	120.162	110.237	-0.124361	0.4962	0.650763	no
Plrg1	28.8343	27.5642	-0.0649869	0.6701	0.794547	no
Pls1	1.54273	2.18471	0.501955	0.02865	0.0679243	no
Plscr1	11.667	13.6516	0.226637	0.16855	0.293468	no
Plscr3	1.53835	1.51998	-0.0173331	0.94995	0.973027	no
Pltp	6.28079	6.72051	0.0976248	0.922	0.957671	no
Plxdc1	3.83391	5.43511	0.503492	0.0091	0.0252515	yes
Plxnc1	8.73856	9.67578	0.146983	0.3163	0.470348	no
Pmaip1	30.5598	30.1022	-0.0217641	0.88255	0.934265	no
Pmel	2.64699	2.81422	0.088378	0.77755	0.869031	no
Pmf1	33.7396	23.8061	-0.503111	0.0031	0.00983478	yes
Pml	19.5809	19.083	-0.0371544	0.80175	0.884827	no
Pmm1	18.4299	18.537	0.00836252	0.961	0.979004	no
Pmm2	54.2817	59.4116	0.13028	0.36265	0.520901	no
Pmpca	23.033	23.6053	0.0354087	0.86655	0.924804	no
Pmpcb	29.1044	27.9158	-0.060153	0.69655	0.813702	no
Pms1	5.53407	5.08177	-0.12301	0.48835	0.643373	no
Pms2	9.61002	9.26214	-0.0531944	0.74505	0.847264	no
Pmvk	17.395	17.5339	0.0114708	0.9449	0.970135	no
Pnkd	34.0482	34.532	0.020354	0.9224	0.957889	no
Pnkp	35.6447	35.0842	-0.0228665	0.87585	0.930424	no
Pnn	66.0069	65.8959	-0.00242926	0.9858	0.991758	no
Pno1	17.9545	18.232	0.0221251	0.89225	0.939658	no
Pnp	20.1555	19.5855	-0.0413894	0.77765	0.869129	no
Pnpla2	32.4267	32.3718	-0.00244584	0.98615	0.991912	no
Pnpla6	10.2805	11.6314	0.178116	0.24515	0.390247	no
Pnpla7	21.2914	27.8897	0.389462	0.00605	0.0176784	yes
Pnpla8	5.61994	5.20234	-0.111395	0.50515	0.659121	no
Pnpo	12.8331	14.0921	0.135017	0.41605	0.574749	no
Pnpt1	18.083	20.0799	0.151117	0.3149	0.468711	no
Pnrc1	105.373	104.219	-0.0158882	0.9078	0.949278	no
Pnrc2	84.0673	75.7809	-0.149711	0.29575	0.447358	no
Poc1a	9.16309	5.85168	-0.646983	0.00155	0.00531723	yes
Poc1b	8.467	7.56219	-0.163047	0.4755	0.631762	no
Poc5	7.88259	8.09735	0.0387807	0.8225	0.897733	no
Podnl1	6.77195	11.4121	0.752924	0.00015	0.000653255	yes
Pofut1	7.01445	6.47652	-0.115112	0.4652	0.622165	no
Pofut2	16.1772	16.3235	0.0129917	0.93585	0.96514	no
Pogk	21.5277	18.7424	-0.199889	0.2293	0.372562	no
Poglut1	11.032	12.5341	0.184156	0.2566	0.401617	no
Pogz	6.95968	7.4303	0.0943992	0.5239	0.675642	no
Pola1	8.32691	6.36117	-0.388489	0.0141	0.0368458	yes
Pola2	23.3801	21.4537	-0.124055	0.41215	0.571015	no
Polb	26.4428	30.4665	0.204349	0.2596	0.40527	no
Pold1	20.433	16.7625	-0.28566	0.0558	0.11938	no
Pold2	24.1644	22.4498	-0.106179	0.4941	0.648793	no
Pold3	21.8038	21.4609	-0.0228673	0.87715	0.931161	no
Pold4	57.7198	53.3834	-0.112675	0.47075	0.627258	no

Poldip2	13.7171	13.5669	-0.0158872	0.9232	0.95817	no
Poldip3	45.2453	43.041	-0.0720572	0.6064	0.744778	no
Pole	5.62848	3.50878	-0.681774	5.00E-05	0.000236281	yes
Pole2	10.9816	9.3761	-0.228025	0.20675	0.344821	no
Pole3	31.593	31.1575	-0.0200262	0.9165	0.954219	no
Pole4	45.1238	37.7384	-0.257856	0.08365	0.166775	no
Polg	21.3445	22.0245	0.0452475	0.75995	0.857192	no
Polg2	7.12137	8.2989	0.220764	0.35085	0.508213	no
Polh	11.3773	9.0973	-0.32265	0.05385	0.11577	no
Poli	14.4705	15.9892	0.143975	0.3583	0.516187	no
Polk	8.69335	8.39186	-0.0509214	0.7388	0.843077	no
Poll	11.3783	11.7417	0.0453547	0.7854	0.874343	no
Polm	23.6121	20.894	-0.176433	0.23655	0.381148	no
Polq	2.29463	1.42171	-0.690637	1.00E-04	0.000450026	yes
Polr1a	7.57976	9.02449	0.251693	0.10055	0.194152	no
Polr1b	5.3959	6.67525	0.306959	0.0683	0.140888	no
Polr1c	31.3492	30.6504	-0.0325241	0.8355	0.905703	no
Polr1d	225.257	194.389	-0.212625	0.13745	0.249659	no
Polr1e	7.78069	8.49381	0.126513	0.4364	0.594438	no
Polr2a	69.2863	63.1534	-0.13371	0.34	0.49646	no
Polr2b	29.7152	29.5507	-0.00800735	0.9536	0.975139	no
Polr2c	32.4703	32.8507	0.0168052	0.91525	0.953522	no
Polr2d	47.0559	50.77	0.1096	0.4767	0.632916	no
Polr2e	67.1564	69.8095	0.0558984	0.7023	0.817402	no
Polr2f	85.2898	92.7405	0.120826	0.45865	0.616125	no
Polr2g	82.7629	77.6708	-0.0916111	0.54115	0.690446	no
Polr2h	31.602	34.6973	0.134809	0.4351	0.593194	no
Polr2i	28.6007	27.1624	-0.0744403	0.69175	0.810294	no
Polr2j	107.868	97.7851	-0.141584	0.35915	0.517052	no
Polr2k	81.5982	80.4964	-0.0196142	0.90845	0.949549	no
Polr2l	21.9652	27.1525	0.305865	0.04985	0.108516	no
Polr2m	84.3142	79.5269	-0.0843315	0.5519	0.699659	no
Polr3a	10.8848	11.5674	0.0877464	0.564	0.709784	no
Polr3b	14.0984	14.8839	0.0782193	0.591	0.732364	no
Polr3c	67.0399	58.4814	-0.197041	0.17195	0.298255	no
Polr3d	7.35875	8.49718	0.207522	0.25785	0.403145	no
Polr3e	8.27337	10.5117	0.345448	0.027	0.0645939	no
Polr3f	9.88552	9.06611	-0.124833	0.4159	0.57462	no
Polr3g	4.87967	4.88655	0.00203142	0.994	0.996202	no
Polr3gl	19.9316	17.5725	-0.181736	0.2796	0.429184	no
Polr3h	3.02179	4.00825	0.40757	0.56035	0.706741	no
Polr3k	17.9262	19.0067	0.0844413	0.5752	0.71916	no
Polrmt	10.4124	10.9079	0.0670599	0.66605	0.79138	no
Pom121	15.0167	15.9854	0.0901929	0.5757	0.719594	no
Pomc	1.641	1.6609	0.0173851	0.96185	0.979615	no
Pomgnt1	8.38304	8.1127	-0.047292	0.7834	0.872957	no
Pomk	3.51108	4.48216	0.35228	0.05865	0.124463	no
Pomp	250.362	236.119	-0.0844988	0.5502	0.698193	no
Pomt1	2.65541	2.05546	-0.369476	0.4558	0.613347	no
Pomt2	1.26462	1.58045	0.321634	0.23535	0.379673	no
Pon2	43.8373	41.1378	-0.0916935	0.52905	0.680221	no
Pop1	5.30236	7.0254	0.405945	0.01915	0.0480107	yes
Pop4	34.2438	34.422	0.00748782	0.9615	0.979344	no
Pop5	38.0427	40.1606	0.0781616	0.6233	0.758448	no
Pop7	37.5381	35.0965	-0.0970287	0.5583	0.705087	no
Popdc2	3.51997	4.32091	0.295772	0.1639	0.287206	no
Por	21.2679	21.3236	0.0037679	0.97945	0.988455	no

Pot1a	15.4032	15.6799	0.0256906	0.86785	0.925713	no
Pot1b	14.2644	13.315	-0.0993649	0.51375	0.666645	no
Pou2af1	1.50857	2.32759	0.625658	0.0146	0.0379571	yes
Pou2f1	2.92642	2.78189	-0.073069	0.8874	0.936912	no
Pou2f2	8.89934	10.6952	0.265195	0.1026	0.197413	no
Pou6f1	14.3367	19.591	0.45048	0.00215	0.00711112	yes
Pp2d1	1.09466	1.07546	-0.0255258	0.97425	0.98597	no
Ppa1	18.208	19.2392	0.0794789	0.6389	0.770741	no
Ppa2	20.0986	18.9742	-0.0830581	0.6329	0.766127	no
Ppan	17.2874	21.3895	0.307186	0.0601	0.127104	no
Ppap2c	1.43189	1.34394	-0.0914438	0.7872	0.87556	no
Ppapdc1b	4.21869	5.25372	0.316546	0.17295	0.299562	no
Ppapdc2	8.67969	9.28437	0.0971607	0.55835	0.705125	no
Ppard	5.8741	6.93774	0.240098	0.16855	0.293468	no
Ppargc1b	1.67221	2.44086	0.545628	0.01655	0.0422807	yes
Ppat	8.50658	9.36715	0.139032	0.66735	0.792338	no
Ppcdc	16.0656	16.9113	0.0740123	0.63015	0.763768	no
Ppcs	15.5397	17.8962	0.203698	0.2361	0.380562	no
Ppdpf	76.9667	81.5455	0.0833721	0.58495	0.727408	no
Ppfia1	18.7047	19.127	0.032208	0.8224	0.897707	no
Ppfibp1	1.46664	1.34984	-0.119727	0.58935	0.73105	no
Pphln1	11.9287	12.029	0.0120721	0.9368	0.965724	no
Ppia	2626.32	2345	-0.163451	0.2835	0.433605	no
Ppib	309.168	285.704	-0.11387	0.5062	0.660149	no
Ppic	13.2209	17.1771	0.377672	0.0324	0.0752751	no
Ppid	40.5888	39.0403	-0.0561185	0.70165	0.816974	no
Ppie	31.1652	36.1464	0.213916	0.1773	0.305574	no
Ppif	26.7189	24.8945	-0.102038	0.5315	0.682265	no
Ppig	19.1858	18.0203	-0.090411	0.51735	0.6699	no
Ppih	40.6041	34.025	-0.255033	0.14155	0.255188	no
Ppil1	52.8452	47.0531	-0.167481	0.26595	0.412991	no
Ppil2	82.2344	79.9698	-0.0402868	0.78455	0.873838	no
Ppil3	37.7521	36.9526	-0.0308801	0.8497	0.914245	no
Ppil4	35.4307	34.4209	-0.0417159	0.76875	0.863077	no
Ppip5k1	2.64575	2.42179	-0.127601	0.50535	0.659346	no
Ppip5k2	14.8693	15.9324	0.0996249	0.492	0.646685	no
Ppm1a	32.1767	30.1224	-0.0951806	0.5073	0.660937	no
Ppm1b	42.119	40.0589	-0.0723452	0.6331	0.766251	no
Ppm1d	12.6519	12.7163	0.00732549	0.96225	0.979811	no
Ppm1f	9.50464	11.3658	0.25799	0.0887	0.175127	no
Ppm1g	109.349	101.352	-0.109568	0.73345	0.839077	no
Ppm1h	24.2235	26.7334	0.142233	0.3323	0.487993	no
Ppm1j	45.1888	42.1779	-0.0994768	0.50015	0.654375	no
Ppm1k	3.20292	3.71991	0.21588	0.2163	0.356895	no
Ppm1m	42.8992	45.2185	0.0759624	0.609	0.746776	no
Ppm1n	0.60165	1.14722	0.931145	0.03	0.0705373	no
Ppme1	25.0399	23.3959	-0.0979745	0.5173	0.669872	no
Ppox	16.4434	16.8465	0.034946	0.8303	0.902603	no
Ppp1ca	638.125	624.058	-0.0321593	0.83205	0.903643	no
Ppp1cb	61.463	56.9383	-0.11032	0.43495	0.593081	no
Ppp1cc	193.418	182.687	-0.0823453	0.5643	0.71001	no
Ppp1r10	12.3481	13.0185	0.0762717	0.6082	0.746168	no
Ppp1r11	27.1959	26.9615	-0.0124864	0.93655	0.965608	no
Ppp1r12a	127.869	128.566	0.00784524	0.9565	0.976703	no
Ppp1r12b	4.23128	3.84274	-0.138958	0.4647	0.621755	no
Ppp1r12c	52.6659	49.8169	-0.0802352	0.5684	0.713312	no
Ppp1r13b	11.3671	11.7757	0.0509468	0.7361	0.841138	no

Ppp1r14b	42.9344	47.6596	0.150631	0.34155	0.498115	no
Ppp1r15a	132.686	148.285	0.16036	0.25795	0.403274	no
Ppp1r15b	51.9492	51.7618	-0.00521416	0.9714	0.984271	no
Ppp1r16a	5.79594	6.07603	0.0680865	0.7059	0.820118	no
Ppp1r16b	14.9542	17.1409	0.196893	0.17215	0.298528	no
Ppp1r18	195.745	183.568	0	1	1	no
Ppp1r2	16.1111	14.4757	-0.154425	0.57765	0.721138	no
Ppp1r21	24.8891	23.5731	-0.0783746	0.5877	0.729748	no
Ppp1r35	36.9327	33.4451	-0.143101	0.38565	0.54466	no
Ppp1r37	12.6199	13.5581	0.103457	0.50955	0.662938	no
Ppp1r3e	2.36363	2.74305	0.214776	0.4097	0.56867	no
Ppp1r3f	1.22068	1.87386	0.618331	0.16185	0.284228	no
Ppp1r7	13.3245	12.046	-0.145533	0.338	0.494358	no
Ppp1r8	35.7994	34.0205	-0.0735335	0.61415	0.751212	no
Ppp1r9b	89.3476	99.6034	0.156765	0.26925	0.417006	no
Ppp2ca	147.725	144.502	-0.031825	0.82285	0.897976	no
Ppp2cb	66.279	64.172	-0.0466076	0.77975	0.870483	no
Ppp2r1a	109.602	114.572	0.06398	0.6457	0.775979	no
Ppp2r1b	17.4792	16.909	-0.0478514	0.77265	0.865679	no
Ppp2r2a	21.9929	20.7526	-0.0837509	0.56425	0.70996	no
Ppp2r2d	25.9662	24.5899	-0.0785692	0.6052	0.743887	no
Ppp2r3c	16.204	14.9753	-0.113763	0.5073	0.660937	no
Ppp2r4	74.334	70.434	-0.0777511	0.58335	0.726109	no
Ppp2r5a	159.14	160.875	0.0156397	0.9141	0.952915	no
Ppp2r5b	12.2614	11.5602	-0.0849625	0.59745	0.737753	no
Ppp2r5c	122.883	105.642	-0.218105	0.14255	0.256492	no
Ppp2r5d	29.7282	28.0812	-0.0822288	0.69715	0.81408	no
Ppp2r5e	19.8705	18.6918	-0.0882218	0.5359	0.686191	no
Ppp3ca	41.3727	43.2421	0.0637561	0.6603	0.787291	no
Ppp3cb	47.0389	43.3534	-0.117708	0.4192	0.577777	no
Ppp3cc	91.497	91.1637	-0.00526407	0.97075	0.984008	no
Ppp3r1	61.9881	57.1708	-0.116714	0.41105	0.569928	no
Ppp4c	160.175	146.357	-0.130154	0.35425	0.511846	no
Ppp4r1	21.6733	21.2819	-0.0262914	0.85735	0.919364	no
Ppp4r2	63.3812	51.7815	-0.291618	0.03805	0.0862719	no
Ppp5c	42.2176	44.5946	0.0790247	0.5903	0.731817	no
Ppp6c	45.2699	37.8721	-0.257415	0.0856	0.169958	no
Ppp6r1	129.11	152.286	0.238186	0.09995	0.193183	no
Ppp6r2	12.1197	11.9926	-0.0152142	0.92505	0.95906	no
Ppp6r3	35.165	32.9109	-0.0955724	0.50015	0.654375	no
Pprc1	5.76178	7.0404	0.289142	0.0694	0.142789	no
Ppt1	28.9322	29.5442	0.0301996	0.8392	0.907818	no
Ppt2	8.83454	9.52863	0.109113	0.55225	0.699914	no
Pptc7	12.4818	11.3412	-0.138257	0.3614	0.519571	no
Ppwd1	6.66119	6.92882	0.0568299	0.7877	0.875895	no
Pqbp1	72.5046	70.9707	-0.0308483	0.8353	0.90564	no
Pqlc1	6.81269	7.19821	0.0794143	0.67655	0.799174	no
Pqlc2	2.44295	1.8708	-0.384967	0.1444	0.259102	no
Pqlc3	54.298	51.1908	-0.0850147	0.5518	0.699582	no
Pradc1	9.3238	8.58003	-0.119935	0.87585	0.930424	no
Praf2	14.2584	14.1351	-0.0125351	0.94275	0.968999	no
Pramef8	12.0247	11.7196	-0.0370736	0.81025	0.890227	no
Prc1	29.1053	16.7546	-0.79672	5.00E-05	0.000236281	yes
Prcc	32.0861	33.6852	0.0701635	0.63685	0.769075	no
Prcp	8.41022	7.92527	-0.0856846	0.6046	0.743396	no
Prdm1	10.8953	8.6861	-0.326926	0.0333	0.0770819	no
Prdm10	3.26412	3.19513	-0.0308193	0.87275	0.928543	no

Prdm11	2.38923	3.23053	0.435226	0.0821	0.164121	no
Prdm15	5.73331	5.58469	-0.0378923	0.809	0.889504	no
Prdm2	23.561	23.771	0.0127978	0.92745	0.960434	no
Prdm4	12.7419	15.6922	0.300465	0.0438	0.0972473	no
Prdm9	1.1196	1.43343	0.356484	0.176	0.303705	no
Prdx1	204.528	177.471	-0.204708	0.1442	0.258822	no
Prdx2	110.279	109.477	-0.0105348	0.94065	0.967884	no
Prdx3	51.5015	55.0568	0.0963041	0.5126	0.665679	no
Prdx4	23.4109	17.7058	-0.402958	0.02465	0.0598153	no
Prdx5	120.156	113.496	-0.0822738	0.58285	0.725743	no
Prdx6	46.5242	57.7755	0.312478	0.0293	0.0692096	no
Preb	21.0433	21.196	0.0104338	0.9823	0.989788	no
Prelid1	334.124	305.628	-0.128607	0.37405	0.53269	no
Prep	19.9716	20.0577	0.00620324	0.96725	0.982195	no
Prepl	7.11577	7.42601	0.0615675	0.7136	0.825504	no
Prex1	164.487	178.475	0.117746	0.42975	0.588024	no
Prf1	90.5748	69.5792	-0.380453	0.0081	0.022802	yes
Prg4	1.47404	1.50844	0.0332826	0.9887	0.993358	no
Prickle3	15.1672	16.7931	0.146912	0.5938	0.734763	no
Prim1	25.1542	16.6781	-0.592844	0.00035	0.0014053	yes
Prim2	11.0601	8.64926	-0.354715	0.04595	0.101282	no
Primpol	9.40634	9.41118	0.000741347	0.9967	0.997633	no
Prkaa1	16.1563	16.1932	0.00329792	0.98205	0.98965	no
Prkab1	44.8562	37.3275	-0.26507	0.06645	0.137663	no
Prkab2	5.36628	5.22895	-0.0373998	0.8244	0.898984	no
Prkaca	22.0455	20.5043	-0.104555	0.4878	0.642865	no
Prkacb	86.3509	84.763	-0.026776	0.848	0.913251	no
Prkag1	110.65	99.5458	-0.152568	0.2845	0.434739	no
Prkag2	19.3073	17.3561	-0.153706	0.32815	0.48368	no
Prkar1a	181.698	200.171	0.139688	0.3341	0.490086	no
Prkar2a	10.2209	11.2995	0.144734	0.3457	0.502703	no
Prkar2b	1.37761	0.87423	-0.656086	0.0196	0.0489684	yes
Prkca	13.0071	12.4476	-0.0634345	0.66215	0.788645	no
Prkcb	38.189	38.3015	0.00424097	0.9765	0.986974	no
Prkcd	33.8361	31.8636	-0.0866517	0.55045	0.698435	no
Prkce	2.53905	2.36865	-0.100225	0.5794	0.72276	no
Prkch	123.36	113.372	-0.121818	0.39305	0.552206	no
Prkci	3.6094	3.67247	0.0249911	0.89105	0.938954	no
Prkcq	141.006	136.616	-0.0456274	0.74695	0.848577	no
Prkcsh	65.0803	67.4818	0.0522788	0.7153	0.826577	no
Prkcz	7.88586	9.49934	0.26856	0.08615	0.170881	no
Prkd2	85.2334	96.1976	0.174581	0.22005	0.361309	no
Prkd3	16.4757	22.0329	0.419321	0.00395	0.0121693	yes
Prkdc	9.23603	10.7386	0.217458	0.12935	0.238021	no
Prkra	2.47714	2.22889	-0.152354	0.59035	0.731854	no
Prkrip1	10.7295	11.8266	0.140453	0.4272	0.585602	no
Prkrir	27.9433	28.3139	0.0190062	0.891	0.938944	no
Prkx	32.1747	35.9189	0.15882	0.26655	0.413732	no
Prmt1	62.8255	65.412	0.0582064	0.68915	0.808362	no
Prmt10	13.6307	13.1347	-0.0534672	0.7334	0.839077	no
Prmt2	4.15908	5.22626	0.329515	0.1101	0.209209	no
Prmt3	11.2569	12.7765	0.182682	0.25515	0.399916	no
Prmt5	40.8666	43.2862	0.0829821	0.5577	0.704519	no
Prmt6	10.5037	11.2894	0.104077	0.52605	0.67745	no
Prmt7	10.405	10.6575	0.0345853	0.8355	0.905703	no
Prnp	193.476	87.8406	-1.1392	5.00E-05	0.000236281	yes
Prorsd1	8.06974	9.15953	0.182752	0.30615	0.459124	no

Prosc	19.1127	18.695	-0.0318769	0.8531	0.916547	no
Proser1	14.6116	13.9385	-0.0680386	0.6501	0.779435	no
Proz	2.35611	2.22309	-0.0838403	0.72755	0.834964	no
Prpf18	32.6864	31.7922	-0.0400181	0.78265	0.872451	no
Prpf19	20.6746	21.2451	0.0392723	0.78315	0.872788	no
Prpf3	17.4488	17.2176	-0.0192452	0.90065	0.945017	no
Prpf31	15.8791	15.7492	-0.0118488	0.9405	0.967814	no
Prpf38a	33.4928	31.416	-0.0923514	0.5438	0.69275	no
Prpf38b	79.4849	82.2598	0.0495067	0.72925	0.836058	no
Prpf39	22.8966	23.4662	0.0354502	0.86125	0.92164	no
Prpf4	10.8011	10.6896	-0.0149697	0.91945	0.956049	no
Prpf40a	36.8699	33.6796	-0.130567	0.3537	0.511261	no
Prpf4b	48.8785	47.0668	-0.0544909	0.69455	0.812172	no
Prpf6	33.6461	33.4425	-0.00875744	0.95205	0.974325	no
Prpf8	63.5806	63.4658	-0.0026067	0.9852	0.991465	no
Prps1	26.1887	31.2896	0.256739	0.092	0.180451	no
Prps1l3	16.0312	16.4056	0.0333031	0.82215	0.897559	no
Prps2	20.4766	19.5216	-0.0689099	0.63595	0.76837	no
Prpsap1	43.8528	43.7207	-0.00435212	0.97465	0.986135	no
Prpsap2	23.2643	23.9547	0.0421898	0.79265	0.879004	no
Prr11	9.4839	5.60428	-0.758951	5.00E-05	0.000236281	yes
Prr12	11.4951	11.8562	0.0446333	0.75755	0.855483	no
Prr13	334.809	359.406	0.102275	0.4664	0.623262	no
Prr14	61.829	56.6503	-0.126199	0.37325	0.531884	no
Prr14l	9.99678	10.2833	0.040764	0.77425	0.866852	no
Prr24	9.02183	8.47903	-0.0895204	0.6365	0.768811	no
Prr3	16.5243	17.986	0.122285	0.50585	0.659815	no
Prr5	3.73556	5.73898	0.619469	0.00605	0.0176784	yes
Prr5l	1.59753	2.30249	0.527354	0.01435	0.0373953	yes
Prr7	11.2836	18.1379	0.684776	0.00015	0.000653255	yes
Prrc1	8.88064	9.23121	0.0558564	0.71895	0.829003	no
Prrc2a	89.2252	96.023	0.105928	0.45725	0.614736	no
Prrc2b	71.468	73.2363	0.0352608	0.81235	0.891521	no
Prrc2c	44.6701	49.4849	0.147679	0.3038	0.45658	no
Prrg2	1.4562	1.23506	-0.237622	0.52905	0.680221	no
Prrt1	0.912531	1.47799	0.695693	0.1451	0.26012	no
Prrt2	1.34347	2.23175	0.732205	0.0061	0.0177994	yes
Prss12	0.490741	1.27588	1.37846	0.00055	0.0021091	yes
Prss16	3.35082	5.79813	0.791074	4.00E-04	0.00158415	yes
Prss2	1.02342	1.04521	0.0304	0.95265	0.974605	no
Prss30	2.97182	5.66236	0.930056	3.00E-04	0.00122185	yes
Prss53	4.16035	4.26778	0.0367833	0.94325	0.969259	no
Prss57	1.44349	1.09491	-0.398748	0.1899	0.322883	no
Prune	24.8916	22.9132	-0.119484	0.4106	0.569505	no
Psap	265.607	237.648	-0.160471	0.263	0.409437	no
Psat1	24.7782	20.1778	-0.296305	0.0503	0.109327	no
Psd	1.49739	1.65499	0.144365	0.79045	0.877778	no
Psd4	66.666	65.3836	-0.0280219	0.8408	0.908936	no
Psen1	32.8686	31.7641	-0.0493123	0.7334	0.839077	no
Psen2	19.8017	20.6124	0.0578909	0.7073	0.82111	no
Psenen	182.924	167.349	-0.128386	0.37405	0.53269	no
Psip1	61.8883	52.8464	-0.227863	0.12105	0.225762	no
Pskh1	12.8667	14.9867	0.220036	0.1535	0.27224	no
Psma1	131.363	122.996	-0.094946	0.50455	0.658618	no
Psma2	202.129	181.693	-0.153772	0.27555	0.424425	no
Psma3	122.589	121.665	-0.0109156	0.943	0.969172	no
Psma4	147.762	137.093	-0.108119	0.4511	0.608577	no

Psma5	86.9543	80.1468	-0.117614	0.4149	0.573677	no
Psma6	149.463	135.084	-0.145929	0.3059	0.458837	no
Psma7	152.935	151.912	-0.009681	0.94655	0.971104	no
Psmb1	280.179	262.607	-0.0934452	0.5094	0.662877	no
Psmb10	156.546	152.424	-0.038495	0.78495	0.874132	no
Psmb2	127.622	118.545	-0.106447	0.4614	0.618489	no
Psmb3	274.208	247.648	-0.146978	0.30365	0.456403	no
Psmb4	228.973	224.65	-0.0275014	0.84625	0.912062	no
Psmb5	120.062	121.795	0.0206847	0.88445	0.935379	no
Psmb6	90.8258	82.1297	-0.145198	0.31985	0.474498	no
Psmb7	140.915	123.874	-0.185953	0.19525	0.329874	no
Psmb8	299.256	308.712	0.0448839	0.75425	0.853272	no
Psmb9	266.209	238.796	-0.156781	0.2706	0.418509	no
Psmc1	80.5105	74.4808	-0.112309	0.4359	0.593997	no
Psmc2	63.1623	56.219	-0.168007	0.23815	0.382939	no
Psmc3	92.7492	89.3515	-0.0538442	0.7028	0.817755	no
Psmc3ip	4.04979	3.90085	-0.054058	0.9405	0.967814	no
Psmc4	80.4241	76.2004	-0.077829	0.58045	0.723609	no
Psmc5	76.1277	76.0352	-0.00175381	0.9904	0.994175	no
Psmc6	61.233	56.9062	-0.105725	0.4663	0.623187	no
Psmd1	29.6001	28.3893	-0.0602534	0.67165	0.79553	no
Psmd10	24.5105	22.6755	-0.112266	0.47705	0.63313	no
Psmd11	42.4501	39.8936	-0.0896105	0.5436	0.692607	no
Psmd12	45.3035	39.9422	-0.181709	0.2196	0.360746	no
Psmd13	87.4255	83.9641	-0.0582816	0.6848	0.805334	no
Psmd14	80.4427	71.7189	-0.165608	0.24395	0.388916	no
Psmd2	81.6817	82.1807	0.008787	0.95205	0.974325	no
Psmd3	59.975	60.9581	0.0234562	0.8762	0.930614	no
Psmd4	119.614	113.418	-0.0767387	0.5924	0.73357	no
Psmd5	24.7908	24.3798	-0.024121	0.8723	0.92833	no
Psmd6	68.1139	65.6221	-0.0537675	0.7189	0.828972	no
Psmd7	44.1117	44.4368	0.0105922	0.94145	0.968284	no
Psmd8	142.808	130.717	-0.127625	0.36885	0.527403	no
Psmd9	10.8686	10.8115	-0.00760009	0.9639	0.980433	no
Psme1	374.811	376.95	0.00820976	0.9537	0.975156	no
Psme2	295.933	296.684	0.00365568	0.9799	0.988598	no
Psme2b	22.2799	24.4783	0.135762	0.71445	0.826014	no
Psme3	66.414	69.2071	0.0594308	0.67405	0.797222	no
Psme4	19.6447	18.7066	-0.0705963	0.62255	0.757849	no
Psmf1	13.7226	13.5604	-0.0171515	0.9116	0.951475	no
Psmg1	15.9746	18.8917	0.241973	0.1924	0.326066	no
Psmg2	35.2368	44.0757	0.3229	0.0419	0.0936534	no
Psmg3	19.5556	17.2147	-0.183939	0.3342	0.490161	no
Psmg4	39.7616	40.4969	0.0264384	0.8926	0.939914	no
Pspc1	19.1546	18.4155	-0.0567723	0.7132	0.82519	no
Psph	24.1821	23.4819	-0.0423891	0.79255	0.878976	no
Psrc1	3.39852	2.53636	-0.422147	0.09145	0.179557	no
Pstk	26.7413	28.9026	0.11213	0.4961	0.650693	no
Pstpip1	133.19	141.047	0.0826895	0.55515	0.702303	no
Ptar1	7.12792	6.69838	-0.089669	0.632	0.765445	no
Ptbp1	152.665	150.741	-0.0182937	0.90475	0.94752	no
Ptbp2	9.41892	8.67096	-0.11937	0.46005	0.617312	no
Ptbp3	75.5993	65.5993	-0.204693	0.1424	0.256459	no
Ptcd1	16.2377	16.4496	0.0187098	0.92385	0.958492	no
Ptcd2	33.244	32.1715	-0.0473082	0.7599	0.857177	no
Ptcd3	19.1	19.3501	0.0187675	0.90495	0.947673	no
Ptch1	6.78357	7.3915	0.123824	0.43985	0.597857	no

Ptdss1	28.914	28.9177	0.000183118	0.99875	0.998948	no
Ptdss2	5.75837	6.87547	0.255796	0.17445	0.301561	no
Pten	21.0428	25.1383	0.256557	0.0678	0.140008	no
Ptger1	51.6846	49.9981	-0.0478615	0.8651	0.923927	no
Ptger2	0.531842	1.16962	1.13698	6.00E-04	0.00228064	yes
Ptger4	25.0064	18.9936	-0.396783	0.0068	0.0195677	yes
Ptges2	7.22736	6.96764	-0.0527992	0.77155	0.865023	no
Ptges3	49.581	47.942	-0.0484997	0.7388	0.843077	no
Ptges3l	1.33785	1.6738	0.323211	0.6728	0.796357	no
Ptgr1	1.33844	0.463158	-1.53097	4.00E-04	0.00158415	yes
Ptgr2	13.016	13.7547	0.0796404	0.61265	0.749884	no
Ptk2b	200.102	84.4987	-1.24374	5.00E-05	0.000236281	yes
Ptma	918.617	838.787	-0.131158	0.37545	0.534178	no
Ptms	98.8141	67.7532	-0.544427	0.00025	0.00103712	yes
Ptov1	1.46086	1.79885	0.300256	0.30395	0.456727	no
Ptp4a1	5.76616	7.45437	0.370476	0.32135	0.476252	no
Ptp4a2	146.777	130.408	-0.170593	0.2285	0.371604	no
Ptp4a3	51.6076	40.1883	-0.36081	0.0122	0.0325386	yes
Ptplad1	19.9809	22.8539	0.193822	0.1939	0.328056	no
Ptplb	4.70277	4.13278	-0.186395	0.30185	0.454413	no
Ptpmt1	10.5664	11.7767	0.156452	0.40715	0.565954	no
Ptpn1	52.6438	48.7458	-0.110986	0.4313	0.589516	no
Ptpn11	23.9018	24.633	0.0434733	0.76015	0.857308	no
Ptpn12	31.5759	22.0271	-0.519548	3.00E-04	0.00122185	yes
Ptpn13	6.20231	6.63386	0.0970428	0.51875	0.671098	no
Ptpn18	110.034	102.528	-0.10192	0.47705	0.63313	no
Ptpn2	68.4669	61.0526	-0.165355	0.2782	0.427574	no
Ptpn21	1.33574	1.70115	0.348872	0.0944	0.184293	no
Ptpn22	202.054	196.205	-0.0423819	0.76935	0.863448	no
Ptpn23	9.07714	8.83156	-0.039571	0.7936	0.879656	no
Ptpn3	1.05366	1.54216	0.549543	0.0106	0.0288545	yes
Ptpn4	18.5962	16.8605	-0.141358	0.3282	0.483723	no
Ptpn6	334.333	285.127	-0.229683	0.11125	0.210895	no
Ptpn7	55.617	56.0851	0.0120915	0.9307	0.962092	no
Ptpn9	9.46975	9.46484	-0.00074747	0.99755	0.998087	no
Ptpra	35.5319	36.5489	0.040713	0.7764	0.868254	no
Ptprc	839.057	741.407	-0.178502	0.38035	0.539355	no
Ptprcap	703.071	678.779	-0.0507285	0.7255	0.8336	no
Ptpre	10.9557	10.0693	-0.121717	0.46025	0.617499	no
Ptprj	8.70254	10.0162	0.202825	0.1667	0.291087	no
Ptprs	3.1332	3.94076	0.330836	0.05135	0.111205	no
Ptprv	2.94577	3.1757	0.108432	0.5318	0.682439	no
Ptrh1	3.70817	4.20753	0.182265	0.5455	0.694076	no
Ptrh2	6.36963	6.08819	-0.065197	0.71105	0.823756	no
Ptrhd1	2.45259	2.36872	-0.0501975	0.92275	0.95797	no
Pts	46.7131	50.0024	0.0981676	0.526	0.677422	no
Pttg1	86.1607	89.7203	0.0584044	0.7058	0.820029	no
Pttg1ip	44.9872	41.8763	-0.10338	0.46755	0.624314	no
Puf60	116.545	113.276	-0.0410501	0.773	0.865892	no
Pum1	25.0874	26.6541	0.0873906	0.55	0.69804	no
Pum2	59.9875	58.032	-0.047811	0.74225	0.845387	no
Pura	12.2333	14.5877	0.253942	0.1446	0.259382	no
Purb	29.266	29.1302	-0.00671313	0.9609	0.97893	no
Purg	2.07233	1.82654	-0.182134	0.5671	0.712238	no
Pus1	22.5699	25.1071	0.153693	0.3335	0.489388	no
Pus10	9.51109	9.74484	0.0350268	0.8662	0.924556	no
Pus3	15.0673	15.4336	0.0346516	0.8548	0.917841	no

Pus7	3.31608	4.75751	0.520727	0.0045	0.0136593	yes
Pus7l	5.5594	6.04525	0.120872	0.50135	0.655472	no
Pusl1	9.96043	10.6081	0.0908867	0.82375	0.898541	no
Pvr	14.7599	12.7828	-0.207483	0.1838	0.314527	no
Pwp1	14.9477	14.4832	-0.0455484	0.771	0.864626	no
Pwp2	7.35231	8.99271	0.290559	0.07065	0.144922	no
Pwwp2a	11.3418	12.1543	0.0998155	0.51885	0.671141	no
Pxk	26.1817	24.6438	-0.087337	0.5534	0.700969	no
Pxmp2	3.22267	2.80882	-0.198292	0.52225	0.674192	no
Pxmp4	15.0297	12.0982	-0.31302	0.07445	0.151222	no
Pxn	45.3013	43.1332	-0.0707538	0.62295	0.7581	no
Pycard	151.934	113.561	-0.41998	0.00295	0.00941349	yes
Pycr2	29.8266	32.6172	0.129035	0.39775	0.556825	no
Pycrl	14.9554	12.8442	-0.219557	0.2153	0.355685	no
Pydc3	55.4551	57.1068	0.0423429	0.76085	0.857796	no
Pydc4	13.9922	14.5872	0.0600861	0.6998	0.815802	no
Pygb	37.6312	36.6749	-0.037139	0.795	0.880586	no
Pygm	1.32159	1.56329	0.242302	0.35915	0.517052	no
Pygo2	23.1601	24.6217	0.0882915	0.54645	0.694959	no
Pyhin1	89.8588	91.8199	0.031147	0.8234	0.898312	no
Pyroxd1	6.89752	6.87539	-0.00463758	0.9898	0.993841	no
Pyurf	1.31681	1.4377	0.126715	0.5626	0.708528	no
Qars	75.7734	71.6559	-0.080606	0.56645	0.711713	no
Qdpr	38.9801	31.9099	-0.288734	0.0585	0.124212	no
Qk	13.6758	13.8484	0.0180978	0.90355	0.946769	no
Qpct	1.51451	3.31866	1.13175	1.00E-04	0.000450026	yes
Qpctl	5.5704	6.3066	0.179081	0.34705	0.504147	no
Qprt	13.6194	10.2812	-0.405658	0.0328	0.0760742	no
Qrich1	40.8302	40.5972	-0.00825683	0.95455	0.9757	no
Qrsl1	10.7231	10.6587	-0.00868502	0.95985	0.978369	no
Qsox1	26.1865	27.1233	0.0507071	0.73265	0.83858	no
Qsox2	2.99576	3.59235	0.262005	0.2247	0.367158	no
Qtrt1	15.3473	19.5363	0.348173	0.0452	0.0998906	no
Qtrtd1	3.31452	4.70641	0.505826	0.01255	0.0333435	yes
R3hcc1	12.0603	12.3105	0.0296287	0.86445	0.923526	no
R3hcc1l	10.9301	11.2685	0.0439951	0.784	0.873391	no
R3hdm1	15.7567	15.6615	-0.00874136	0.9537	0.975156	no
R3hdm2	23.3586	22.6043	-0.0473551	0.74025	0.843872	no
R3hdm4	88.5386	88.0507	-0.00797172	0.95735	0.977108	no
Rab1	43.8217	37.1814	-0.237065	0.1007	0.194372	no
Rab10	61.2989	60.0983	-0.0285371	0.8447	0.911421	no
Rab11a	27.6072	25.5986	-0.108979	0.46655	0.623415	no
Rab11b	32.633	30.1878	-0.11237	0.4257	0.584124	no
Rab11fip1	5.67548	7.45879	0.3942	0.01345	0.0353818	yes
Rab11fip2	4.14014	4.75098	0.198546	0.22555	0.368181	no
Rab11fip3	2.39506	2.67686	0.160477	0.39765	0.556729	no
Rab11fip4	26.7982	25.5432	-0.0691966	0.63865	0.770603	no
Rab12	10.1849	10.0648	-0.0171105	0.92175	0.957467	no
Rab14	45.7575	41.5527	-0.139065	0.3345	0.490509	no
Rab18	24.2997	23.5863	-0.0429867	0.76415	0.860034	no
Rab19	47.3697	49.5772	0.0657112	0.6636	0.789572	no
Rab1b	135.757	130.166	-0.0606731	0.6641	0.789874	no
Rab20	3.31486	3.54636	0.0973928	0.78265	0.872451	no
Rab21	50.2239	51.2719	0.0297929	0.841	0.909041	no
Rab22a	22.7379	22.2558	-0.0309183	0.8392	0.907818	no
Rab23	2.04789	2.02089	-0.0191496	0.92495	0.958971	no
Rab24	29.5225	29.6527	0.00634833	0.96545	0.981181	no

Rab27a	56.071	54.809	-0.0328437	0.81765	0.894764	no
Rab28	14.4165	17.8037	0.304456	0.0689	0.14193	no
Rab2a	50.1245	42.5715	-0.235629	0.1063	0.203198	no
Rab2b	7.44902	5.98376	-0.315999	0.49735	0.651763	no
Rab31	2.39547	2.65087	0.146157	0.48465	0.640016	no
Rab32	1.26287	2.00129	0.66422	0.0246	0.0597062	no
Rab33b	16.1612	12.405	-0.381611	0.0127	0.0336825	yes
Rab35	77.1419	70.7723	-0.124329	0.3804	0.539404	no
Rab37	50.0141	46.4876	-0.105489	0.45535	0.613022	no
Rab39b	1.04118	1.05409	0.0177745	0.94565	0.970482	no
Rab3a	1.83612	1.85114	0.0117543	0.9854	0.991539	no
Rab3d	5.97888	5.51903	-0.11546	0.49695	0.651396	no
Rab3gap1	19.3459	19.9288	0.0428279	0.76835	0.862724	no
Rab3gap2	8.42452	8.55157	0.0215942	0.92595	0.959669	no
Rab3ip	6.71068	6.47357	-0.0518972	0.76925	0.863377	no
Rab40c	9.20997	9.49394	0.0438103	0.7978	0.882247	no
Rab43	18.9989	18.821	-0.0135727	0.92805	0.960717	no
Rab4a	3.21378	11.2043	1.80171	5.00E-05	0.000236281	yes
Rab4b	78.2679	77.2904	-0.0181304	0.9004	0.944896	no
Rab5a	37.6864	34.156	-0.141904	0.3416	0.498157	no
Rab5b	28.346	27.7985	-0.0281397	0.84815	0.913356	no
Rab5c	143.972	128.614	-0.162736	0.2488	0.394182	no
Rab6a	32.9543	30.0207	-0.134506	0.4063	0.565285	no
Rab6b	1.49899	2.07624	0.469985	0.02985	0.0702924	no
Rab7	215.35	209.633	-0.0388182	0.7922	0.878781	no
Rab7l1	34.628	29.4736	-0.232517	0.1472	0.26311	no
Rab8a	195.306	199.362	0.02965	0.8341	0.904922	no
Rab8b	65.6002	59.8009	-0.133532	0.34665	0.503711	no
Rab9	15.8409	15.3963	-0.0410684	0.80735	0.888368	no
Rabac1	105.721	112.024	0.0835508	0.56845	0.713362	no
Rabep1	9.03746	8.17885	-0.14402	0.5629	0.708779	no
Rabep2	30.9074	29.039	-0.0899628	0.54925	0.697439	no
Rabepk	9.64517	10.4965	0.122035	0.48655	0.641732	no
Rabgap1	8.88428	8.89416	0.001602	0.99255	0.995342	no
Rabgap1l	28.7004	33.5529	0.225363	0.13695	0.249161	no
Rabgef1	10.2883	9.81156	-0.0684527	0.67535	0.798238	no
Rabggta	21.1039	21.3166	0.01447	0.924	0.958591	no
Rabggtb	33.1138	38.5222	0.218256	0.16115	0.283224	no
Rabif	30.9425	27.3386	-0.178648	0.2413	0.385894	no
Rabl3	3.966	3.78024	-0.0692066	0.75285	0.852262	no
Rabl6	22.0316	22.2382	0.013462	0.9261	0.95974	no
Rac1	190.984	173.858	-0.135542	0.3441	0.500944	no
Rac2	753.949	668.406	-0.173742	0.2955	0.447047	no
Rac3	1.08274	0.639984	-0.758575	0.1409	0.254348	no
Racgap1	138.157	93.6978	-0.560227	5.00E-05	0.000236281	yes
Rad1	13.8196	14.6172	0.0809475	0.66715	0.792194	no
Rad17	15.2175	15.0276	-0.0181157	0.90565	0.947998	no
Rad18	5.44418	5.01456	-0.118591	0.52455	0.676234	no
Rad21	108.615	87.7515	-0.307733	0.03295	0.0763493	no
Rad23a	41.5208	40.5849	-0.0328929	0.8502	0.914573	no
Rad23b	35.1929	37.9714	0.109627	0.4341	0.592357	no
Rad50	8.76951	8.40682	-0.0609346	0.69855	0.815067	no
Rad51	14.6261	7.90843	-0.887085	5.00E-05	0.000236281	yes
Rad51ap1	8.9599	4.53158	-0.983469	5.00E-05	0.000236281	yes
Rad51c	2.38257	1.2714	-0.906101	0.00075	0.00278555	yes
Rad51d	8.97284	9.25896	0.0452851	0.8264	0.900347	no
Rad52	11.8212	11.684	-0.016844	0.9246	0.958829	no

Rad54b	2.68916	1.35453	-0.989361	0.00035	0.0014053	yes
Rad54l	6.03042	3.73947	-0.689423	0.0569	0.121351	no
Rad54l2	9.75429	8.83493	-0.142819	0.3278	0.483366	no
Rad9a	51.8492	48.6304	-0.0924621	0.8272	0.900787	no
Rad9b	2.08726	2.69635	0.369395	0.19245	0.326142	no
Rae1	50.6873	53.2038	0.0699033	0.6229	0.758052	no
Raf1	73.3069	78.5106	0.0989378	0.5103	0.66362	no
Rai1	11.5918	14.0597	0.278469	0.2055	0.343166	no
Rala	19.2708	18.1306	-0.0879847	0.5658	0.711238	no
Ralb	2.21822	4.18838	0.916987	2.00E-04	0.0008488	yes
Ralbp1	33.696	31.3635	-0.103488	0.4657	0.62268	no
Ralgapa1	12.7825	14.4049	0.172389	0.23055	0.374016	no
Ralgapa2	9.88788	9.75994	-0.0187886	0.8989	0.944041	no
Ralgapb	22.7731	22.9232	0.00947813	0.9432	0.969222	no
Ralgds	4.45308	7.37832	0.728488	5.00E-05	0.000236281	yes
Raly	270.838	254.715	-0.0885444	0.5402	0.68972	no
Ran	142.518	122.876	-0.213946	0.1312	0.240848	no
Ranbp1	91.8496	82.1755	-0.160566	0.28265	0.432614	no
Ranbp10	22.1634	32.9945	0.574049	1.00E-04	0.000450026	yes
Ranbp2	25.4591	25.5084	0.00279225	0.98535	0.991539	no
Ranbp3	39.8108	38.6887	-0.0412452	0.78005	0.870653	no
Ranbp6	9.0544	9.13721	0.013136	0.9342	0.964269	no
Ranbp9	56.2532	57.9999	0.0441171	0.8201	0.896347	no
Rangap1	64.3221	62.8581	-0.0332151	0.8139	0.892306	no
Rangrf	4.59811	6.16584	0.423258	0.2181	0.359009	no
Rap1a	99.5566	95.9879	-0.052664	0.7093	0.822552	no
Rap1b	576.063	421.349	-0.451213	0.0027	0.00870467	yes
Rap1gap2	51.9213	41.4481	-0.325022	0.0227	0.0556652	no
Rap1gds1	47.2728	44.068	-0.101279	0.47775	0.633845	no
Rap2a	16.8854	10.3209	-0.710203	5.00E-05	0.000236281	yes
Rap2b	23.5644	19.6763	-0.260153	0.0721	0.147378	no
Rap2c	35.7175	33.5999	-0.0881723	0.5298	0.680875	no
Rapgef1	60.2795	55.4149	-0.121395	0.38495	0.543955	no
Rapgef2	13.243	14.9906	0.178826	0.2147	0.354919	no
Rapgef4	3.83633	5.3406	0.477276	0.00685	0.0196859	yes
Rapgef6	32.8007	38.065	0.214738	0.1258	0.232923	no
Raph1	2.86905	3.79381	0.403075	0.01285	0.0340304	yes
Rara	41.7014	51.5346	0.305446	0.02975	0.0701013	no
Rarg	7.80677	9.79665	0.327563	0.05645	0.120577	no
Rarres2	1.52124	1.28303	-0.245695	0.85885	0.920092	no
Rars	28.4919	28.4443	-0.00241359	0.987	0.99247	no
Rars2	13.4685	14.0459	0.0605628	0.7201	0.829963	no
Rasa1	33.371	29.7256	-0.166892	0.2327	0.376526	no
Rasa2	6.985	8.62395	0.30409	0.04985	0.108516	no
Rasa3	212.225	222.976	0.0712931	0.6263	0.76076	no
Rasa4	2.44185	2.56961	0.0735749	0.7376	0.842198	no
Rasal1	9.08325	8.06996	-0.170647	0.29335	0.444681	no
Rasal3	193.533	204.854	0.0820169	0.5907	0.732133	no
Rasd1	2.57862	2.69485	0.0636028	0.88235	0.934191	no
Rasgrp1	58.7191	64.7225	0.140436	0.32145	0.476301	no
Rasgrp2	282.245	260.498	-0.115678	0.4191	0.57773	no
Rasgrp4	1.65243	1.4477	-0.190829	0.59355	0.734543	no
Rasl11a	2.55539	3.18488	0.317695	0.2914	0.44238	no
Rasl11b	6.35237	4.90748	-0.372313	0.0769	0.155412	no
Rasl12	1.57157	0.694792	-1.17755	0.0014	0.00485767	yes
Rassf1	59.2136	57.486	-0.0427185	0.76405	0.859975	no
Rassf2	41.8085	49.1225	0.232588	0.10175	0.19605	no

Rassf3	9.88822	10.887	0.138821	0.3798	0.538781	no
Rassf5	120.25	120.11	-0.0016748	0.9899	0.993885	no
Rassf7	5.30781	6.34229	0.256886	0.2299	0.373244	no
Raver1	54.5269	52.6363	-0.0509086	0.72775	0.835057	no
Raver2	2.12115	2.87006	0.436238	0.8413	0.90924	no
Rb1	19.5369	18.03	-0.115803	0.544	0.692929	no
Rb1cc1	12.9069	12.0229	-0.102353	0.47515	0.631452	no
Rbak	6.45093	6.93486	0.104359	0.53705	0.687165	no
Rbbp4	53.7219	50.2998	-0.0949596	0.505	0.658998	no
Rbbp5	19.6602	19.6727	0.000918412	0.99435	0.99634	no
Rbbp6	26.8952	28.871	0.102268	0.48605	0.641325	no
Rbbp7	77.7027	65.2979	-0.250928	0.15895	0.280125	no
Rbbp8	12.8826	11.1336	-0.210509	0.17125	0.297274	no
Rbbp9	1.50528	1.8112	0.266907	0.3405	0.496964	no
Rbck1	125.706	125.902	0.00224608	0.98825	0.993076	no
Rbfa	41.0502	40.4597	-0.0209035	0.89285	0.940051	no
Rbks	7.60316	7.81441	0.0395386	0.8562	0.918508	no
Rbl1	22.9996	20.1883	-0.188088	0.21395	0.354052	no
Rbl2	53.9566	58.2264	0.109874	0.4407	0.598585	no
Rbm10	37.084	36.6487	-0.0170329	0.932	0.962815	no
Rbm12	12.4031	12.4243	0.00246836	0.9943	0.996304	no
Rbm12b1	3.6354	3.57889	-0.0226018	0.90565	0.947998	no
Rbm12b2	4.94519	4.95189	0.00195533	0.99485	0.996573	no
Rbm14	19.9024	18.3581	-0.116526	0.51215	0.665278	no
Rbm14-rbm4	1.61682	0.107258	-3.914	0.18115	0.310934	no
Rbm15	10.8502	10.9875	0.0181318	0.90845	0.949549	no
Rbm15b	16.4118	17.2486	0.0717469	0.63885	0.770726	no
Rbm17	78.0957	75.6385	-0.0461225	0.7446	0.847025	no
Rbm18	19.9383	20.2985	0.0258329	0.88105	0.933335	no
Rbm19	7.32436	8.23063	0.1683	0.3049	0.457687	no
Rbm22	58.8907	57.7758	-0.0275751	0.8447	0.911421	no
Rbm25	50.0444	47.367	-0.079326	0.5744	0.718415	no
Rbm26	13.4357	14.4634	0.106331	0.5387	0.688377	no
Rbm27	14.5818	14.0344	-0.0551933	0.7002	0.816048	no
Rbm28	26.5289	25.3956	-0.0629832	0.65995	0.78702	no
Rbm3	508.094	453.842	-0.162906	0.25925	0.404867	no
Rbm33	24.2084	24.6829	0.0280073	0.84515	0.91164	no
Rbm34	10.4957	11.6697	0.152965	0.32925	0.484866	no
Rbm38	80.7202	71.7995	-0.168954	0.23665	0.381257	no
Rbm39	218.491	220.657	0.0142327	0.9217	0.95743	no
Rbm4	9.28822	9.71707	0.0651184	0.83765	0.907003	no
Rbm41	5.59976	5.86314	0.0663089	0.67765	0.800018	no
Rbm42	80.863	81.2382	0.00667934	0.96175	0.979528	no
Rbm43	27.3376	27.6473	0.0162482	0.9127	0.952173	no
Rbm45	31.8283	29.6539	-0.102088	0.49375	0.648466	no
Rbm48	9.76958	9.19924	-0.0867813	0.6683	0.793066	no
Rbm4b	7.57014	6.87638	-0.138672	0.46775	0.624463	no
Rbm5	74.8502	83.9009	0.164679	0.24275	0.387511	no
Rbm6	31.8155	31.4572	-0.0163421	0.9113	0.951289	no
Rbm7	38.5298	33.6296	-0.196241	0.1826	0.312913	no
Rbm8a	29.2659	28.2365	-0.0516595	0.7235	0.832197	no
Rbms1	67.7854	61.0424	-0.151163	0.2825	0.43246	no
Rbms2	7.33012	6.25229	-0.229453	0.14245	0.256492	no
Rbmx	26.3387	25.1736	-0.0652755	0.66575	0.791197	no
Rbmx2	7.22735	6.96702	-0.0529249	0.7833	0.872886	no
Rbmxl1	38.5921	34.7136	-0.152807	0.3101	0.46362	no
Rbpj	10.3969	12.9949	0.321794	0.0297	0.0700139	no

Rbpms	0.124776	1.22906	3.30014	0.00015	0.000653255	yes
Rbx1	52.2872	47.8636	-0.127529	0.37585	0.534596	no
Rc3h1	11.5724	11.6017	0.0036485	0.97945	0.988455	no
Rc3h2	6.19725	6.52263	0.0738268	0.6203	0.756088	no
Rcan1	2.98545	3.01116	0.0123704	0.95555	0.976242	no
Rcan3	3.97004	3.58555	-0.146961	0.3942	0.553337	no
Rcbtb1	20.7079	19.9205	-0.0559275	0.69795	0.814691	no
Rcbtb2	18.7663	16.4238	-0.192355	0.1918	0.325314	no
Rcc1	17.0777	14.5111	-0.23496	0.13675	0.248855	no
Rcc2	32.9044	34.2269	0.056851	0.6893	0.808484	no
Rccd1	5.28209	6.45032	0.288262	0.1261	0.233282	no
Rce1	21.5426	21.3158	-0.0152711	0.9292	0.961287	no
Rchy1	49.2433	45.7597	-0.10585	0.4932	0.647937	no
Rcl1	10.3997	12.9932	0.321218	0.06545	0.136078	no
Rcn1	18.8015	21.7967	0.213264	0.152	0.270102	no
Rcn2	14.3502	14.0038	-0.0352442	0.8282	0.901292	no
Rcn3	33.7488	0.29079	-6.85872	5.00E-05	0.000236281	yes
Rcor1	18.5764	19.9597	0.10362	0.4936	0.648366	no
Rcor2	1.2439	1.05679	-0.235191	0.4787	0.634628	no
Rcor3	5.69134	6.01993	0.0809795	0.68535	0.805619	no
Rcsd1	59.8446	88.2567	0.560484	1.00E-04	0.000450026	yes
Rdh10	6.78023	7.01733	0.0495882	0.7871	0.875545	no
Rdh11	12.3153	11.7567	-0.0669684	0.7026	0.817617	no
Rdh12	3.40515	4.36863	0.359461	0.1282	0.236404	no
Rdh13	3.24849	3.98322	0.294168	0.13785	0.250179	no
Rdh14	7.77008	8.27426	0.0907011	0.644	0.774701	no
Rdm1	30.8972	26.8098	-0.204717	0.21005	0.349137	no
Rdx	44.01	41.0774	-0.0994848	0.4899	0.64491	no
Reck	5.0915	5.69042	0.160445	0.33755	0.493812	no
Recql	32.2705	29.1485	-0.146793	0.35915	0.517052	no
Recql4	1.8936	1.08545	-0.802839	0.0039	0.0120284	yes
Recql5	9.49791	9.73243	0.0351893	0.8258	0.900013	no
Reep3	8.13675	8.87249	0.124886	0.4096	0.568598	no
Reep4	29.0348	28.3788	-0.0329665	0.8304	0.902643	no
Reep5	88.821	69.1184	-0.36183	0.01075	0.0292077	yes
Rel	10.3393	11.7955	0.190103	0.24505	0.390123	no
Rela	56.6346	55.8386	-0.0204195	0.88315	0.93462	no
Relb	18.1849	20.694	0.186471	0.2259	0.36859	no
Rell1	6.9059	9.78185	0.502277	0.00245	0.0079866	yes
Relt	15.3379	17.4778	0.188423	0.218	0.358911	no
Repin1	1.50218	1.84942	0.300023	0.21375	0.353812	no
Reps1	46.7198	40.3341	-0.212036	0.1419	0.255689	no
Rer1	98.6999	94.5828	-0.0614712	0.65775	0.785273	no
Rere	10.3767	12.4356	0.26112	0.0701	0.143986	no
Rest	6.35078	7.91843	0.318279	0.04925	0.107378	no
Ret	2.28312	3.18186	0.478861	0.0059	0.0172945	yes
Retsat	1.53802	1.43735	-0.0976612	0.741	0.844386	no
Rev1	2.97581	2.72655	-0.126205	0.79635	0.881431	no
Rev3l	10.2089	10.3286	0.0168097	0.90645	0.94854	no
Rexo1	27.6725	27.2688	-0.0212028	0.8793	0.932391	no
Rexo2	78.4714	67.8518	-0.20978	0.15415	0.27318	no
Rexo4	39.8037	39.2144	-0.0215192	0.8807	0.933133	no
Rfc1	18.6157	17.7333	-0.0700581	0.6308	0.764385	no
Rfc2	49.7673	45.8115	-0.11949	0.41775	0.576353	no
Rfc3	15.1914	10.1504	-0.581714	0.0034	0.0106624	yes
Rfc4	16.6406	12.5099	-0.411635	0.6547	0.782942	no
Rfc5	23.4381	17.5468	-0.417646	0.0086	0.0240265	yes

Rfesd	5.35959	5.34979	-0.00263888	0.9894	0.99368	no
Rffl	4.51186	4.40884	-0.0333203	0.91425	0.953001	no
Rfk	21.5522	23.479	0.123535	0.41745	0.576063	no
Rfng	17.8184	17.6375	-0.0147224	0.9263	0.959764	no
Rft1	6.43547	6.73436	0.0654953	0.72065	0.830312	no
Rftn1	33.6392	47.4427	0.496044	0.00065	0.00244919	yes
Rfwd2	11.6276	11.5717	-0.00695009	0.9622	0.979811	no
Rfwd3	21.396	17.8057	-0.265	0.0661	0.137204	no
Rfx1	26.0998	38.2322	0.550749	5.00E-05	0.000236281	yes
Rfx2	2.43555	2.24133	-0.119895	0.5797	0.722981	no
Rfx3	3.68448	4.57068	0.310947	0.04505	0.0996184	no
Rfx5	8.7039	6.99737	-0.314849	0.0511	0.110762	no
Rfx7	20.2393	20.8272	0.0413084	0.77195	0.865279	no
Rfxank	9.57226	9.77772	0.0306386	0.8843	0.935318	no
Rfxap	15.7913	15.0292	-0.0713645	0.654	0.782569	no
Rgcc	5.3136	6.07352	0.192844	0.4621	0.619204	no
Rgl2	43.9176	47.6123	0.116535	0.4121	0.570957	no
Rgp1	14.4442	15.5697	0.108245	0.625	0.759783	no
Rgs1	75.8724	66.7958	-0.183817	0.2076	0.345879	no
Rgs10	14.2212	19.1856	0.431982	0.02735	0.0653051	no
Rgs11	2.89738	3.87327	0.418804	0.07665	0.155013	no
Rgs14	55.6079	59.9061	0.107413	0.45065	0.608075	no
Rgs16	1.96924	0.895626	-1.13667	8.00E-04	0.0029486	yes
Rgs19	82.6679	90.9348	0.137504	0.36275	0.520992	no
Rgs2	25.6641	29.2662	0.189486	0.19205	0.325621	no
Rgs3	70.7825	63.3064	-0.161042	0.25585	0.400764	no
Rhbdd1	8.46173	8.42796	-0.00576875	0.97025	0.983869	no
Rhbdd2	2.57999	3.01795	0.226203	0.24445	0.389493	no
Rhbdd3	2.0518	2.36163	0.202898	0.45265	0.610202	no
Rhbdf2	16.0432	15.1678	-0.0809456	0.58755	0.7296	no
Rhbdl1	7.01027	5.75455	-0.284767	0.1687	0.293635	no
Rheb	48.4215	42.2186	-0.19777	0.1859	0.317382	no
Rhebl1	5.90067	5.57523	-0.0818462	0.7229	0.831818	no
Rhoa	261.544	237.206	-0.14091	0.32985	0.485516	no
Rhob	3.82817	2.96656	-0.367861	0.1018	0.196136	no
Rhobtb2	6.62584	8.81106	0.411211	0.008	0.0225529	yes
Rhoc	16.8579	25.0143	0.569329	0.0014	0.00485767	yes
Rhof	91.7092	75.5126	-0.280349	0.0494	0.107649	no
Rhog	185.756	152.647	-0.283211	0.04755	0.104226	no
Rhoh	26.3279	30.2714	0.201363	0.1556	0.275314	no
Rhoq	3.55378	2.9491	-0.269081	0.24985	0.395366	no
Rhot1	13.7294	12.7917	-0.102059	0.5024	0.656504	no
Rhot2	41.2266	40.0403	-0.0421228	0.79055	0.877833	no
Ric8	51.699	56.3095	0.123243	0.40575	0.564776	no
Ric8b	7.4943	8.16526	0.123704	0.4272	0.585602	no
Rictor	16.9161	18.0819	0.0961425	0.4912	0.645994	no
Rif1	11.7938	12.8098	0.119218	0.40115	0.560185	no
Rilpl2	75.9753	88.7118	0.223597	0.1215	0.226433	no
Rin3	33.3431	36.8313	0.143545	0.3092	0.462568	no
Ring1	15.7666	16.8249	0.0937252	0.5574	0.704316	no
Rinl	225.528	218.714	-0.0442615	0.7601	0.857308	no
Rint1	6.62294	6.84001	0.0465252	0.78655	0.875057	no
Riok1	23.7211	25.379	0.0974654	0.5062	0.660149	no
Riok2	9.53129	10.4896	0.138211	0.3912	0.550242	no
Riok3	44.5645	43.6892	-0.028619	0.84445	0.911333	no
Ripk1	26.4182	25.6631	-0.0418364	0.7729	0.865808	no
Ripk2	8.306	7.97004	-0.0595682	0.7418	0.84512	no

Ripk3	38.8288	36.2037	-0.100989	0.4916	0.646364	no
Rit1	18.6454	15.7044	-0.24765	0.10695	0.204241	no
Rita1	1.95393	1.94203	-0.00881294	0.9758	0.986676	no
Rlf	13.5785	14.0499	0.049239	0.72835	0.835379	no
Rlim	6.40791	6.75233	0.0755324	0.6157	0.752547	no
Rln3	0.617176	1.03551	0.746595	0.38825	0.547434	no
Rltpr	37.4277	48.5988	0.376814	0.0273	0.0652033	no
Rmdn1	3.48576	4.14676	0.250513	0.271	0.418962	no
Rmdn2	2.19798	2.18065	-0.0114183	0.96785	0.982494	no
Rmdn3	9.32848	10.8913	0.223462	0.1909	0.324068	no
Rmi1	15.6063	15.0058	-0.0566026	0.91675	0.954324	no
Rmi2	1.19782	0.786511	-0.606867	0.0304	0.0713542	no
Rmnd1	12.8761	13.3556	0.0527495	0.75615	0.854504	no
Rmnd5a	30.1402	28.4	-0.0857997	0.54345	0.692504	no
Rmnd5b	52.9961	62.7367	0.243424	0.15825	0.279128	no
Rnaseh1	17.3878	19.2267	0.145036	0.378	0.536994	no
Rnaseh2a	12.8894	13.4795	0.0645812	0.6952	0.812609	no
Rnaseh2b	27.1929	19.7469	-0.461605	0.005	0.0149735	yes
Rnaseh2c	61.8159	54.9065	-0.171003	0.30085	0.453314	no
Rnasek	138.154	130.605	-0.0810588	0.58995	0.731602	no
Rnasel	22.2517	27.3874	0.299595	0.03635	0.0829933	no
Rnaset2a	11.2856	9.59937	-0.233471	0.69215	0.810561	no
Rnaset2b	18.3251	19.5479	0.0931942	0.6062	0.744662	no
Rnf10	69.6735	68.7314	-0.019641	0.89105	0.938954	no
Rnf103	6.29355	6.76144	0.103456	0.54445	0.693265	no
Rnf11	10.9429	9.90551	-0.143691	0.4009	0.559891	no
Rnf111	22.6301	23.2932	0.0416641	0.77245	0.865592	no
Rnf113a1	5.16004	4.64792	-0.150796	0.54975	0.697811	no
Rnf113a2	28.261	28.3228	0.00315511	0.98535	0.991539	no
Rnf114	69.7634	63.9829	-0.124785	0.42915	0.587442	no
Rnf115	56.4546	56.1683	-0.00733491	0.9589	0.977909	no
Rnf121	18.2825	19.8457	0.118361	0.4816	0.637216	no
Rnf123	33.7807	38.1335	0.174859	0.25535	0.400167	no
Rnf125	44.5186	53.252	0.258429	0.0887	0.175127	no
Rnf126	30.9227	30.5143	-0.0191763	0.90095	0.945139	no
Rnf13	39.0022	37.6313	-0.0516235	0.7398	0.843563	no
Rnf130	6.17108	7.72484	0.323981	0.11315	0.213745	no
Rnf135	3.94973	4.52451	0.19601	0.3665	0.524914	no
Rnf138	43.1273	50.8308	0.237101	0.09215	0.180675	no
Rnf139	13.6576	13.5435	-0.0121005	0.94065	0.967884	no
Rnf14	33.427	32.0031	-0.0628008	0.66805	0.792889	no
Rnf141	4.88626	5.4005	0.144365	0.58615	0.728348	no
Rnf144a	11.6683	18.7382	0.683388	5.00E-05	0.000236281	yes
Rnf145	45.9569	48.4617	0.0765623	0.58705	0.729184	no
Rnf146	18.9363	18.739	-0.0151052	0.92245	0.957899	no
Rnf149	11.5232	11.8134	0.0358869	0.8287	0.9016	no
Rnf157	1.14472	0.837188	-0.451371	0.0811	0.162453	no
Rnf166	156.499	135.218	-0.210871	0.1382	0.250474	no
Rnf167	136.377	153.169	0.167526	0.23585	0.380271	no
Rnf168	5.33834	5.1703	-0.0461448	0.778	0.869258	no
Rnf169	7.59042	7.42375	-0.0320311	0.8349	0.905456	no
Rnf170	8.58596	8.21282	-0.0641022	0.6862	0.806255	no
Rnf181	59.8739	61.3703	0.0356115	0.80655	0.887792	no
Rnf185	5.67994	5.2763	-0.106349	0.55645	0.70338	no
Rnf187	96.362	93.4407	-0.0444137	0.7518	0.85166	no
Rnf19a	15.8958	13.953	-0.188066	0.20325	0.34023	no
Rnf19b	27.1556	29.8623	0.137076	0.35	0.507346	no

Rnf2	16.4306	19.7953	0.26877	0.07905	0.159006	no
Rnf20	37.2586	36.6794	-0.0226011	0.87295	0.928629	no
Rnf214	8.53923	7.8156	-0.127749	0.6683	0.793065	no
Rnf215	12.9621	14.129	0.124358	0.5055	0.659468	no
Rnf216	15.6747	15.7461	0.00655985	0.96255	0.979918	no
Rnf219	4.28985	4.5211	0.0757475	0.68365	0.804344	no
Rnf220	32.8674	31.1819	-0.0759474	0.59635	0.736768	no
Rnf24	1.87341	1.5509	-0.272557	0.5527	0.700283	no
Rnf25	16.9123	17.7016	0.0658077	0.6981	0.814744	no
Rnf26	13.4005	11.2423	-0.253348	0.12445	0.230816	no
Rnf31	29.8914	29.7841	-0.005189	0.9715	0.984288	no
Rnf34	17.5291	24.3846	0.476217	0.0023	0.0075545	yes
Rnf38	28.8649	30.8251	0.0947875	0.5055	0.659468	no
Rnf4	47.4943	44.0322	-0.109196	0.44245	0.600304	no
Rnf40	35.2854	35.3541	0.00280578	0.98465	0.991109	no
Rnf41	6.1714	6.73541	0.126169	0.4349	0.593047	no
Rnf43	1.99216	2.02078	0.0205733	0.9201	0.956443	no
Rnf44	57.6467	59.4152	0.0435955	0.75905	0.856601	no
Rnf5	24.5046	27.558	0.169419	0.3064	0.459441	no
Rnf6	26.9918	26.8769	-0.00615811	0.96515	0.981023	no
Rnf7	51.6102	59.1736	0.197297	0.1954	0.330088	no
Rnf8	15.9722	15.906	-0.00598582	0.97035	0.983894	no
Rnft1	6.88243	6.68992	-0.0409311	0.80055	0.884028	no
Rngtt	11.1955	10.3047	-0.119617	0.4327	0.590915	no
Rnh1	59.0235	61.5752	0.061059	0.67145	0.795399	no
Rnls	0.812692	1.09046	0.424154	0.298	0.449961	no
Rnmt	6.60903	6.35496	-0.0565566	0.71705	0.827677	no
Rnmtl1	2.38865	2.78513	0.221545	0.43065	0.588935	no
Rnpc3	15.2563	13.4021	-0.186945	0.24795	0.393232	no
Rnpep	38.763	40.5589	0.0653355	0.6551	0.783261	no
Rnpepl1	34.3271	34.856	0.0220579	0.87915	0.932366	no
Rnps1	89.3238	76.5083	-0.223429	0.12115	0.225889	no
Rock1	30.3969	32.7712	0.108502	0.4466	0.604181	no
Rock2	9.02327	8.98692	-0.00582336	0.9709	0.984084	no
Rogdi	2.72943	3.63782	0.414475	0.1266	0.234022	no
Rom1	31.983	35.9093	0.167056	0.2714	0.419434	no
Romo1	75.9832	80.5445	0.0841062	0.76995	0.863847	no
Ropn1l	1.29039	1.81217	0.489914	0.245	0.39007	no
Rora	20.5278	20.8923	0.0253935	0.8579	0.919703	no
Rp2h	8.25379	7.14404	-0.208317	0.32295	0.477961	no
Rp9	60.1839	60.4196	0.00564036	0.9715	0.984288	no
Rpa1	91.9703	78.7982	-0.223006	0.11425	0.215482	no
Rpa2	79.6991	56.2343	-0.503113	5.00E-04	0.00193907	yes
Rpa3	49.9008	39.0043	-0.355433	0.041	0.0919182	no
Rpain	9.95541	9.3188	-0.0953367	0.7984	0.882703	no
Rpap1	12.6375	12.8655	0.0257899	0.8626	0.922304	no
Rpap2	14.5089	13.6339	-0.0897397	0.6321	0.76554	no
Rpap3	7.10904	7.43566	0.0648076	0.7239	0.832507	no
Rpe	19.6111	19.7218	0.00812611	0.957	0.976917	no
Rpf1	37.2442	38.1281	0.0338381	0.82715	0.900787	no
Rpf2	15.137	16.5804	0.131395	0.4414	0.599271	no
Rpgr	1.91336	1.84718	-0.0507811	0.8365	0.906314	no
Rpgrip1	1.67146	2.33969	0.485208	0.3894	0.548541	no
Rph3al	2.29324	2.91119	0.344221	0.15715	0.2775	no
Rpia	32.3212	34.8461	0.108514	0.46325	0.620356	no
Rpl10	1857.26	1890.17	0.02534	0.863	0.92255	no
Rpl10a	1245.21	1248.65	0.00398635	0.97765	0.987556	no

Rpl11	2646.67	2679.57	0.0178259	0.90295	0.946337	no
Rpl12	2171.35	2332.29	0.103155	0.4804	0.636141	no
Rpl13	1979.69	1996	0.0118313	0.93895	0.966952	no
Rpl13a	3752.32	2679.99	-0.485555	0.0058	0.017041	yes
Rpl14	1049.79	1070.7	0.0284507	0.84445	0.911333	no
Rpl15	451.406	453.378	0.00628925	0.96685	0.981973	no
Rpl17	2863.6	2869.47	0.00295261	0.9854	0.991539	no
Rpl18	2373.21	2612.78	0.138744	0.36145	0.519611	no
Rpl18a	1328.33	1329.14	0.000883594	0.9956	0.997041	no
Rpl19	2597.16	2682.66	0.0467253	0.7573	0.855392	no
Rpl21	704.882	742.76	0.0755145	0.6218	0.757223	no
Rpl22	183.109	191.142	0.0619391	0.66305	0.78925	no
Rpl22l1	448.413	388.122	-0.20832	0.1459	0.261249	no
Rpl23	1206.34	1259.9	0.0626783	0.67225	0.79596	no
Rpl23a	3573.63	3539.34	-0.0139117	0.92615	0.95975	no
Rpl24	1628.83	1501.81	-0.117132	0.41595	0.574656	no
Rpl26	31.479	12.0643	-1.38365	0.48165	0.63727	no
Rpl27	1357.34	1344.54	-0.013668	0.92655	0.959953	no
Rpl27a	1089.77	1074.06	-0.0209513	0.8886	0.937548	no
Rpl28	2281	2242.48	-0.0245681	0.86635	0.924646	no
Rpl29	1719.73	2033.38	0.241697	0.104	0.199606	no
Rpl3	1250.29	1257.64	0.00845908	0.9563	0.97657	no
Rpl30	56.7422	59.342	0.0646306	0.9567	0.976752	no
Rpl31	758.393	718.207	-0.0785467	0.5862	0.728359	no
Rpl32	4263.11	4026.08	-0.0825286	0.5891	0.730869	no
Rpl34	262.891	242.922	-0.113968	0.8042	0.886533	no
Rpl34-ps1	3034.51	3027.29	-0.00343954	0.98295	0.990175	no
Rpl35	2038.24	2071.73	0.0235124	0.87135	0.927752	no
Rpl35a	2117.3	2095.27	-0.0150875	0.9177	0.954919	no
Rpl36	1981.48	2015.75	0.0247384	0.86155	0.921781	no
Rpl36a	901.359	995.47	0.143277	0.312	0.465661	no
Rpl36al	840.008	821.025	-0.0329764	0.8131	0.891845	no
Rpl37	635.092	624.939	-0.0232511	0.86925	0.926453	no
Rpl37a	1713.65	1740.07	0.0220694	0.8823	0.934191	no
Rpl38	4155.9	4095.24	-0.0212132	0.8854	0.935946	no
Rpl39	4131.36	4129.93	-0.000501517	0.99745	0.998044	no
Rpl39l	1.15105	1.17218	0.0262537	0.9514	0.974004	no
Rpl4	2176.16	2167.23	-0.00593353	0.97545	0.986548	no
Rpl41	4242.07	4216.44	-0.00874295	0.9525	0.974593	no
Rpl5	844.802	828.598	-0.0279406	0.8603	0.920989	no
Rpl6	1254.25	1238.23	-0.0185438	0.90255	0.946129	no
Rpl7	2118.9	2068.74	-0.0345616	0.82675	0.900589	no
Rpl7a	1761.4	1680.7	-0.0676638	0.65705	0.784623	no
Rpl7l1	27.1606	26.4799	-0.0366171	0.8031	0.885749	no
Rpl8	1834.57	1830.08	-0.00353296	0.98215	0.989722	no
Rpl9	2356.11	2400.16	0.0267207	0.8617	0.921858	no
Rplp0	2705.77	2777.67	0.0378328	0.83845	0.907423	no
Rplp1	4485.27	4865.78	0.117476	0.4454	0.603054	no
Rplp2	3509.43	3617.02	0.0435634	0.76375	0.859817	no
Rpn1	46.7651	45.4716	-0.0404672	0.7764	0.868254	no
Rpn2	85.3328	85.5931	0.00439464	0.9734	0.985449	no
Rpp14	8.31404	8.70633	0.0665142	0.70345	0.818282	no
Rpp21	80.5523	74.6836	-0.109135	0.5133	0.666343	no
Rpp25l	28.2399	26.8138	-0.0747586	0.6706	0.794859	no
Rpp30	9.70397	9.67065	-0.00496251	0.9749	0.986246	no
Rpp38	2.26335	2.2615	-0.00118321	0.97295	0.985135	no
Rpp40	0.653123	1.06091	0.699873	0.038	0.0861807	no

Rprd1a	2.89999	3.017	0.0570688	0.7668	0.861724	no
Rprd1b	12.4776	11.019	-0.179342	0.23245	0.37619	no
Rprd2	9.4381	10.5343	0.158523	0.2739	0.422428	no
Rps10	2894.98	2791.86	-0.0523268	0.7273	0.834785	no
Rps11	3812.8	2667.56	-0.515332	9.00E-04	0.00327966	yes
Rps12	3107.79	3171.73	0.0293798	0.8406	0.908831	no
Rps13	2471.8	2456.56	-0.00892256	0.95325	0.974964	no
Rps14	2799.17	2837.27	0.0195041	0.893	0.940125	no
Rps15	3217.91	3412.56	0.0847289	0.56245	0.708402	no
Rps15a	229.631	235.264	0.0349665	0.81885	0.895494	no
Rps16	2843.42	2967.68	0.0617123	0.67925	0.801237	no
Rps17	1742.54	1625.28	-0.100501	0.47485	0.63122	no
Rps18	3264.93	3342.25	0.0337676	0.822	0.897451	no
Rps19	1571.51	1709.31	0.121258	0.4016	0.560602	no
Rps19bp1	17.8841	19.3071	0.11046	0.55865	0.705416	no
Rps2	1513.3	1497.02	-0.015601	0.92085	0.956941	no
Rps20	2195.57	2328.48	0.0847899	0.5536	0.701084	no
Rps21	2645.96	2915	0.139701	0.32225	0.477176	no
Rps23	2532.91	2576.81	0.0247914	0.8694	0.926543	no
Rps24	2203.55	2183.61	-0.0131196	0.93235	0.96305	no
Rps25	1271.42	1266.86	-0.00518679	0.9727	0.984995	no
Rps26	2296.91	2203.18	-0.0601086	0.6709	0.795108	no
Rps27	126.391	126.398	7.92E-05	0.9993	0.999343	no
Rps27a	1580.45	1678.6	0.0869218	0.55165	0.69948	no
Rps27l	160.512	155.835	-0.0426632	0.7775	0.869002	no
Rps27rt	7692.66	7591.59	-0.0190815	0.8978	0.943293	no
Rps28	3215.38	3656.98	0.185664	0.19265	0.326364	no
Rps29	2861.97	2872.63	0.00536368	0.9704	0.983894	no
Rps3	1016.81	990.586	-0.0376958	0.8079	0.888752	no
Rps3a1	2430.84	2416.71	-0.00841452	0.9599	0.978377	no
Rps4x	2559.7	2507.91	-0.0294894	0.8517	0.915587	no
Rps5	1913.02	1909.1	-0.00295546	0.98345	0.99041	no
Rps6	1651.88	1627.45	-0.0214934	0.8926	0.939914	no
Rps6ka1	119.968	118.51	-0.0176482	0.89965	0.944435	no
Rps6ka3	41.3759	40.4068	-0.0341918	0.80815	0.888874	no
Rps6ka4	30.3183	29.8108	-0.0243535	0.85865	0.920073	no
Rps6ka5	3.61065	4.53769	0.329698	0.061	0.128572	no
Rps6kb1	16.4586	16.5595	0.00882576	0.9516	0.97409	no
Rps6kb2	26.7502	25.5199	-0.0679274	0.66645	0.791735	no
Rps6kc1	2.12804	2.21182	0.0557102	0.7883	0.876397	no
Rps7	1696.3	1725.66	0.024763	0.8691	0.926349	no
Rps8	2201.52	2231.06	0.0192274	0.8982	0.943615	no
Rps9	2302.64	2424.52	0.07441	0.6228	0.758009	no
Rpsa	1827.47	1727.31	-0.0813228	0.6485	0.778165	no
Rptor	16.9241	19.3608	0.194059	0.16995	0.295395	no
Rpusd1	4.09029	3.3589	-0.284215	0.2288	0.371955	no
Rpusd2	0.996517	1.41732	0.508195	0.0313	0.0731359	no
Rpusd3	6.26381	5.73068	-0.128335	0.5731	0.717374	no
Rpusd4	25.5154	26.2814	0.0426723	0.78075	0.871049	no
Rqcd1	21.5386	19.4455	-0.147488	0.5113	0.664492	no
Rrad	12.3634	13.3185	0.107357	0.5376	0.687582	no
Rraga	38.5102	35.4008	-0.121456	0.4131	0.571882	no
Rragb	4.44857	6.35361	0.514236	0.00895	0.0248832	yes
Rragc	37.6721	36.466	-0.046947	0.74715	0.848667	no
Rras	23.3444	18.863	-0.307515	0.09255	0.181324	no
Rras2	28.3146	23.034	-0.29778	0.0485	0.105972	no
Rrbp1	27.5028	27.8118	0.0161187	0.91205	0.951748	no

Rreb1	4.3847	5.49928	0.326765	0.03285	0.0761651	no
Rrm1	33.0674	21.9155	-0.593457	1.00E-04	0.000450026	yes
Rrm2	79.5377	46.0088	-0.78973	5.00E-05	0.000236281	yes
Rrm2b	10.4894	9.87027	-0.0877768	0.56385	0.709646	no
Rrn3	16.1174	16.232	0.0102265	0.94645	0.971035	no
Rrnad1	15.0187	15.7312	0.0668697	0.777	0.868719	no
Rrp1	89.5199	93.0462	0.055739	0.69295	0.811108	no
Rrp12	6.38795	8.98702	0.492489	0.0025	0.00813232	yes
Rrp15	11.97	14.5272	0.27933	0.13275	0.243118	no
Rrp1b	10.6937	10.6374	-0.00761033	0.9621	0.979771	no
Rrp36	35.2759	34.7752	-0.0206261	0.89135	0.9392	no
Rrp7a	44.211	46.396	0.0695941	0.66205	0.788579	no
Rrp8	14.8948	15.3724	0.0455314	0.9128	0.952249	no
Rrp9	18.3522	21.2956	0.214604	0.18905	0.321732	no
Rrs1	19.9978	21.1996	0.0841921	0.58345	0.726195	no
Rsad1	0.931092	1.14048	0.292646	0.2557	0.4006	no
Rsad2	0.86193	1.17754	0.45014	0.09685	0.188331	no
Rsbn1	5.97809	6.15475	0.042016	0.88075	0.933144	no
Rsbn1l	24.8325	23.3285	-0.0901344	0.54305	0.692219	no
Rsc1a1	3.87322	5.13789	0.40764	0.44275	0.600595	no
Rsf1	8.16528	7.9251	-0.043073	0.76645	0.861509	no
Rsl1	4.60366	4.7086	0.0325163	0.87525	0.929983	no
Rsl1d1	65.6908	65.7795	0.00194668	0.99125	0.99466	no
Rsl24d1	38.5663	37.9457	-0.0234046	0.876	0.930458	no
Rsph1	1.07512	0.816435	-0.397085	0.3858	0.544829	no
Rsph3a	3.66996	3.35947	-0.127533	0.5843	0.726907	no
Rsph3b	1.72758	1.39017	-0.313494	0.3312	0.486884	no
Rspry1	14.1321	14.7358	0.0603498	0.6875	0.807192	no
Rsrc1	5.28443	5.81937	0.139115	0.43045	0.588765	no
Rsrc2	74.4421	75.5324	0.0209769	0.88095	0.933257	no
Rsrp1	226.813	210.943	-0.104648	0.46545	0.622405	no
Rsu1	45.0966	45.5629	0.0148405	0.92275	0.957971	no
Rtca	27.1661	26.6543	-0.027439	0.86045	0.921093	no
Rtcb	82.0332	81.2462	-0.0139082	0.9227	0.95797	no
Rtel1	15.9276	15.2729	-0.0605508	0.6891	0.808357	no
Rtf1	27.5707	25.9978	-0.084743	0.55855	0.70534	no
Rtfdc1	64.6888	64.9145	0.00502477	0.9713	0.984255	no
Rtn3	79.1036	68.3205	-0.211425	0.1532	0.271845	no
Rtn4	28.6969	27.5732	-0.0576315	0.69415	0.811933	no
Rtn4ip1	4.59009	4.6382	0.0150405	0.9372	0.965939	no
Rtn4rl1	13.2461	16.0472	0.276752	0.06955	0.143052	no
Rtp4	3.28787	3.22218	-0.0291161	0.904	0.947072	no
Rttn	2.86469	2.76878	-0.0491322	0.7739	0.866598	no
Rufy1	12.2636	13.2827	0.115167	0.47055	0.627074	no
Rufy2	5.68538	5.66816	-0.00437689	0.9812	0.989259	no
Rufy3	3.11007	2.20694	-0.494896	0.01615	0.0413937	yes
Rundc1	13.6987	14.9427	0.125411	0.42675	0.585167	no
Rundc3a	5.2788	6.45931	0.291168	0.11775	0.220868	no
Rundc3b	4.44957	3.16535	-0.491302	0.008	0.0225529	yes
Runx1	16.8739	13.2582	-0.347908	0.01705	0.0433541	yes
Runx2	27.642	34.519	0.320529	0.03135	0.0732382	no
Runx3	66.7992	64.1529	-0.0583167	0.6784	0.800569	no
Rusc1	35.5416	37.2228	0.0666767	0.65015	0.779442	no
Ruvbl1	19.7449	21.6981	0.136085	0.3938	0.552929	no
Ruvbl2	24.7779	26.6458	0.104855	0.49465	0.649346	no
Rwdd1	78.4739	75.5891	-0.0540355	0.71025	0.823207	no
Rwdd2a	1.56836	1.12327	-0.481544	0.20745	0.345686	no

Rwdd2b	8.74741	7.21268	-0.278321	0.12555	0.232569	no
Rwdd4a	13.4971	13.5968	0.010624	0.9455	0.970455	no
Rxra	11.4275	11.6867	0.0323598	0.8261	0.900187	no
Rxrb	38.9276	38.7125	-0.00799349	0.95725	0.977087	no
Rybp	10.2878	10.2241	-0.00894978	0.95395	0.975285	no
Ryk	4.37411	3.68207	-0.248474	0.18525	0.316494	no
S100a1	6.37429	6.29393	-0.0183053	0.93775	0.966332	no
S100a10	2341.24	2287.03	-0.0337974	0.82115	0.896953	no
S100a11	1185.56	1153.66	-0.0393573	0.78175	0.87182	no
S100a13	221.797	182.721	-0.279599	0.05155	0.111566	no
S100a2	0.73012	1.27402	0.80318	0.53595	0.68623	no
S100a3	1.47317	1.32305	-0.155058	0.76515	0.860664	no
S100a4	2998.03	3396.01	0.179827	0.22205	0.363763	no
S100a6	1864.69	2089.47	0.1642	0.27355	0.422045	no
S100pbp	15.7136	13.7103	-0.196754	0.25325	0.397617	no
S1pr1	156.545	177.033	0.177435	0.21945	0.360558	no
S1pr2	5.57139	4.76031	-0.226982	0.22555	0.368181	no
S1pr4	157.844	150.96	-0.0643343	0.657	0.784616	no
S1pr5	110.575	21.4333	-2.3671	5.00E-05	0.000236281	yes
Saal1	4.58307	4.12664	-0.151349	0.37955	0.538545	no
Sac3d1	7.31948	6.78172	-0.110089	0.5995	0.739405	no
Sacm1l	31.1238	30.1189	-0.0473466	0.74035	0.843959	no
Sacs	3.27776	3.36443	0.0376523	0.8498	0.914255	no
Sae1	70.5891	64.2806	-0.135061	0.34645	0.503524	no
Safb	46.9154	47.9264	0.0307587	0.82875	0.901627	no
Safb2	43.1908	44.7183	0.0501421	0.72095	0.830536	no
Samd1	17.3425	14.1483	-0.293681	0.0654	0.135991	no
Samd10	5.81723	6.20438	0.0929547	0.61945	0.755482	no
Samd3	22.6612	12.1762	-0.896161	5.00E-05	0.000236281	yes
Samd4b	13.6552	14.0827	0.0444768	0.7658	0.861107	no
Samd8	3.3658	3.42342	0.0244882	0.88495	0.935641	no
Samd9l	51.9022	50.0122	-0.0535162	0.70395	0.818702	no
Samhd1	227.18	270.385	0.25118	0.0869	0.172142	no
Samm50	66.408	64.6884	-0.0378495	0.7919	0.878683	no
Samsn1	27.2769	24.0607	-0.181002	0.23335	0.377353	no
Sap130	23.1864	20.8043	-0.156401	0.27155	0.419592	no
Sap18	9.08099	8.24547	-0.139247	0.6394	0.77106	no
Sap25	40.0887	35.3827	-0.180154	0.67685	0.799435	no
Sap30	9.9518	8.00587	-0.313899	0.1324	0.242603	no
Sap30bp	20.1429	20.5973	0.0321839	0.83295	0.904133	no
Sap30l	25.9138	23.299	-0.15345	0.32555	0.480811	no
Sapcd2	2.92898	1.96412	-0.576513	0.0094	0.0259848	yes
Sar1a	54.1019	51.7058	-0.0653512	0.64435	0.774937	no
Sar1b	47.6021	41.2953	-0.205048	0.17465	0.301855	no
Sarnp	102.754	94.6756	-0.118125	0.40935	0.568374	no
Sars	59.3654	52.2167	-0.185111	0.19255	0.326273	no
Sars2	14.3082	14.4593	0.0151576	0.9303	0.961904	no
Sart1	32.9507	32.7462	-0.00898269	0.94965	0.972875	no
Sart3	21.4812	21.3947	-0.00581982	0.96985	0.983661	no
Sash3	222.062	219.163	-0.0189609	0.89565	0.941903	no
Sass6	4.0149	3.27984	-0.291741	0.24375	0.388659	no
Sat1	74.6395	72.0528	-0.0508857	0.7273	0.834785	no
Satb1	47.1061	47.2701	0.00501172	0.97305	0.985165	no
Sav1	15.3218	15.2398	-0.00774561	0.9624	0.979878	no
Saysd1	5.50001	5.06807	-0.117997	0.53915	0.688778	no
Sbds	34.2665	39.7592	0.21449	0.15995	0.281543	no
Sbf1	67.3391	67.1446	-0.00417341	0.9766	0.987018	no

Sbk1	33.8506	32.5613	-0.0560245	0.69635	0.813563	no
Sbno1	19.6415	18.415	-0.0930179	0.50945	0.662906	no
Sbno2	22.506	22.4773	-0.00183712	0.9905	0.994205	no
Sc5d	9.08037	10.4991	0.209446	0.2255	0.368133	no
Scaf1	30.0584	29.2674	-0.0384731	0.78575	0.874526	no
Scaf11	23.1895	23.2205	0.00192518	0.98975	0.993841	no
Scaf4	17.9556	18.8645	0.0712393	0.6208	0.756488	no
Scaf8	27.7004	28.9119	0.0617592	0.66375	0.789644	no
Scai	4.1364	5.66118	0.452725	0.00305	0.00969839	yes
Scamp2	38.0242	34.6793	-0.132843	0.35765	0.515534	no
Scamp3	103.787	105.262	0.0203602	0.88785	0.937107	no
Scamp4	41.3109	43.1254	0.0620149	0.6822	0.803321	no
Scand1	114.764	113.392	-0.0173523	0.9108	0.951016	no
Scap	22.1306	26.3782	0.253307	0.0784	0.157901	no
Scaper	4.72484	5.59709	0.244411	0.13395	0.244864	no
Scarb1	5.64238	5.32394	-0.0838088	0.6597	0.786802	no
Scarb2	29.6081	36.5032	0.302034	0.0405	0.0910054	no
Scd2	26.8312	26.3051	-0.0285719	0.84175	0.909559	no
Scfd1	29.8693	28.0708	-0.0895919	0.54985	0.697887	no
Scfd2	9.46547	9.2146	-0.0387529	0.8098	0.89001	no
Scimp	1.42078	0.819831	-0.793288	0.0861	0.170801	no
Sclt1	3.20957	3.40214	0.0840643	0.6815	0.802831	no
Scly	25.0904	25.7596	0.0379738	0.8044	0.886642	no
Scmh1	1.10669	1.43789	0.377691	0.14635	0.261929	no
Scml4	18.5344	18.2013	-0.0261614	0.85375	0.917009	no
Scnm1	44.4163	41.146	-0.11034	0.4978	0.652171	no
Sco1	3.13251	3.31503	0.0817024	0.65575	0.783786	no
Sco2	24.6079	25.9191	0.0748929	0.84415	0.911134	no
Scoc	8.9981	7.6044	-0.242786	0.18845	0.32092	no
Scp2	83.5108	85.1514	0.0280689	0.8375	0.906924	no
Scpep1	17.318	23.2229	0.423278	0.0058	0.017041	yes
Scrib	18.0074	19.0035	0.0776721	0.58785	0.729857	no
Scrn2	5.00209	5.06097	0.016881	0.94225	0.968641	no
Scrn3	4.17184	3.5275	-0.242038	0.20555	0.343233	no
Scyl1	26.661	27.0289	0.0197759	0.89	0.93838	no
Scyl2	22.7543	19.9875	-0.187041	0.19925	0.335172	no
Scyl3	16.3668	15.6566	-0.0640013	0.67375	0.797067	no
Sdad1	11.311	13.8496	0.292122	0.0502	0.109143	no
Sdcbp	99.9024	86.9502	-0.20033	0.15215	0.270348	no
Sdcbp2	27.1551	25.6562	-0.0819176	0.6036	0.742593	no
Sdccag3	75.499	66.8902	-0.174663	0.2241	0.366399	no
Sdccag8	13.4751	12.1426	-0.150212	0.33955	0.495967	no
Sde2	14.194	14.945	0.0743804	0.6351	0.767829	no
Sdf2	65.7218	60.9383	-0.109022	0.45565	0.613239	no
Sdf2l1	43.7581	39.0123	-0.165619	0.2847	0.435007	no
Sdf4	53.2265	55.9532	0.072077	0.60515	0.743864	no
Sdha	85.4205	87.2136	0.0299708	0.83085	0.902951	no
Sdhaf1	14.4802	15.2448	0.0742305	0.70355	0.818358	no
Sdhaf2	27.014	23.9957	-0.170934	0.2433	0.388152	no
Sdhb	152.222	128.158	-0.24825	0.08475	0.168602	no
Sdhc	70.0201	64.5492	-0.11737	0.4217	0.580102	no
Sdhd	80.9657	82.6887	0.0303793	0.8324	0.903856	no
Sdr39u1	43.7447	44.1868	0.0145075	0.92235	0.957865	no
Sdr42e1	1.18989	1.28175	0.107284	0.6984	0.81496	no
Sec11a	144.446	150.915	0.0632073	0.65825	0.78567	no
Sec11c	171.799	164.405	-0.0634692	0.65695	0.784596	no
Sec13	73.4864	71.4737	-0.0400647	0.7845	0.873796	no

Sec14l1	15.6156	14.7443	-0.082829	0.5976	0.737871	no
Sec16a	18.6579	20.687	0.14894	0.29205	0.443177	no
Sec22a	1.49134	1.49646	0.00494367	0.9862	0.99192	no
Sec22b	38.8038	36.2776	-0.0971188	0.51695	0.669604	no
Sec22c	5.2973	5.14935	-0.0408685	0.79645	0.881486	no
Sec23a	8.30914	6.67903	-0.315061	0.05065	0.109962	no
Sec23b	31.446	29.9962	-0.0680954	0.6401	0.771535	no
Sec23ip	21.5143	22.7294	0.079266	0.5876	0.729649	no
Sec24a	8.92875	9.35807	0.067753	0.6442	0.774849	no
Sec24b	29.4755	29.9359	0.0223606	0.87445	0.929494	no
Sec24c	58.7317	55.3095	-0.0866127	0.54265	0.691797	no
Sec24d	10.0678	10.4763	0.0573802	0.7092	0.822477	no
Sec31a	37.6321	39.0274	0.052522	0.70805	0.821714	no
Sec31b	0.998046	1.10092	0.141537	0.59735	0.737655	no
Sec61a1	86.0412	83.7098	-0.039632	0.78305	0.872787	no
Sec61a2	3.14901	2.95158	-0.0934097	0.6787	0.800728	no
Sec61b	343.181	323.177	-0.0866444	0.54215	0.691334	no
Sec61g	152.102	143.879	-0.0801798	0.5759	0.719781	no
Sec62	31.8444	31.2137	-0.0288583	0.84075	0.908896	no
Sec63	16.9428	17.0224	0.00676085	0.9644	0.980642	no
Secisbp2	26.2278	25.9977	-0.0127137	0.93015	0.961819	no
Secisbp2l	15.1967	14.8051	-0.0376625	0.79555	0.880905	no
Seh1l	21.4106	21.1443	-0.0180536	0.90695	0.948796	no
Sel1l	17.5474	17.3498	-0.0163404	0.90755	0.949115	no
Selk	112.077	105.444	-0.0880186	0.53935	0.688996	no
Sell	19.2039	26.6628	0.473428	0.00185	0.00622713	yes
Selm	7.51807	8.77573	0.223157	0.35685	0.514644	no
Selo	20.081	18.2102	-0.141084	0.56645	0.711713	no
Selplg	710.147	701.754	-0.0171534	0.91655	0.954243	no
Selt	74.2957	78.2406	0.0746396	0.59535	0.735997	no
Sema4a	178.658	172.023	-0.0545985	0.7101	0.823088	no
Sema4b	6.55212	7.439	0.183147	0.26385	0.410453	no
Sema4d	100.098	112.678	0.170789	0.2258	0.36847	no
Sema4f	14.3357	12.3024	-0.220674	0.14055	0.253839	no
Sema7a	2.96993	3.11589	0.0692163	0.7397	0.843531	no
Senp1	12.7964	12.0521	-0.0864548	0.5715	0.715955	no
Senp2	23.7228	23.6233	-0.00606307	0.9669	0.98201	no
Senp3	43.6305	42.9509	-0.0226479	0.87275	0.928543	no
Senp5	11.5703	11.734	0.020269	0.8891	0.93788	no
Senp6	34.8033	38.3868	0.141387	0.3181	0.472438	no
Senp7	24.2088	21.9629	-0.140463	0.4372	0.595171	no
15-Sep	125.225	122.675	-0.0296817	0.83295	0.904133	no
Sephs1	9.78453	9.57974	-0.0305146	0.8404	0.908712	no
Sephs2	33.0089	28.3301	-0.220519	0.1396	0.25244	no
Sepn1	4.72826	4.59162	-0.0423059	0.81515	0.893039	no
Sepp1	36.1043	50.1923	0.475294	0.00115	0.00407292	yes
Sepsecs	5.64756	6.07151	0.104426	0.59685	0.737231	no
1-Sep	592.952	541.098	-0.132025	0.36045	0.518522	no
10-Sep	1.04286	0.869289	-0.26264	0.43695	0.594946	no
11-Sep	58.0346	49.7294	-0.222814	0.1161	0.21833	no
2-Sep	45.128	39.1905	-0.203517	0.15565	0.275354	no
4-Sep	1.36348	1.30394	-0.0644098	0.8523	0.916023	no
6-Sep	95.4275	87.4865	-0.125346	0.3831	0.542187	no
7-Sep	115.701	102.471	-0.175187	0.2146	0.354828	no
8-Sep	1.29411	1.29878	0.00519669	0.98165	0.989458	no
9-Sep	231.605	236.681	0.0312754	0.82745	0.900921	no
Sepw1	254.152	252.708	-0.00822203	0.9536	0.975139	no

Serac1	1.54419	1.5707	0.024559	0.93215	0.962914	no
Serbp1	79.4221	76.401	-0.0559503	0.6959	0.813212	no
Serf1	1.63103	1.74379	0.0964398	0.84625	0.912062	no
Serf2	118.604	114.597	-0.049582	0.73915	0.843258	no
Sergef	7.76687	8.33485	0.101822	0.60615	0.74464	no
Serhl	15.2824	14.5216	-0.0736721	0.83355	0.90452	no
Serinc1	43.645	40.381	-0.112139	0.42765	0.586014	no
Serinc3	127.599	133.841	0.0689019	0.634	0.76692	no
Serinc4	17.8501	19.5845	0.133779	0.75235	0.851996	no
Serinc5	4.02304	4.38037	0.122767	0.4679	0.624592	no
Serp1	69.2364	67.0754	-0.0457478	0.78825	0.876355	no
Serp2	14.0954	12.6822	-0.152421	0.46125	0.618358	no
Serpina3g	11.4657	9.63389	-0.251134	0.1605	0.282286	no
Serpinb1a	13.7377	14.8738	0.11463	0.4939	0.648627	no
Serpinb1b	1.13389	0.710439	-0.674503	0.09085	0.178643	no
Serpinb6a	29.422	29.3776	-0.00218052	0.99205	0.995095	no
Serpinb6b	106.393	88.2416	-0.26987	0.0572	0.121917	no
Serpinb9	32.327	20.8208	-0.634713	5.00E-05	0.000236281	yes
Serpini1	6.418	11.8315	0.882443	5.00E-05	0.000236281	yes
Sertad1	40.044	38.3946	-0.060679	0.69935	0.815556	no
Sertad2	8.85678	8.04555	-0.138592	0.3644	0.522649	no
Sertad3	10.407	9.15764	-0.184501	0.35345	0.511056	no
Sesn1	15.8363	16.9994	0.102247	0.50675	0.660574	no
Sesn2	3.2421	4.30412	0.408789	0.0451	0.0997164	no
Sesn3	18.8765	20.5039	0.119312	0.43625	0.594291	no
Sestd1	0.232211	1.15794	2.31805	5.00E-05	0.000236281	yes
Set	116.81	117.419	0.0075084	0.95805	0.977423	no
Setd1a	15.9621	15.813	-0.0135435	0.9298	0.961598	no
Setd1b	11.6413	11.8899	0.0304848	0.8329	0.904133	no
Setd2	24.5666	23.9792	-0.034913	0.8063	0.887697	no
Setd3	44.1595	44.8036	0.0208904	0.8806	0.933083	no
Setd4	4.79153	5.87081	0.293072	0.1607	0.282595	no
Setd5	31.1199	32.3071	0.0540127	0.7025	0.817554	no
Setd6	15.9603	15.4245	-0.0492727	0.76445	0.860206	no
Setd7	17.5137	17.954	0.0358239	0.8043	0.886588	no
Setd8	39.7286	38.1211	-0.0595892	0.67535	0.798238	no
Setdb1	15.3034	15.7602	0.042431	0.873	0.928655	no
Setdb2	14.2977	14.5341	0.0236555	0.8782	0.931855	no
Setmar	1.3095	1.33934	0.0325137	0.92355	0.958322	no
Setx	33.7791	37.2327	0.14044	0.3248	0.480005	no
Sf1	86.6499	84.2408	-0.040679	0.7784	0.869554	no
Sf3a1	25.1053	26.9223	0.100809	0.4848	0.640142	no
Sf3a2	53.8768	56.7959	0.0761229	0.59515	0.735853	no
Sf3a3	46.935	44.5173	-0.0762967	0.59705	0.737362	no
Sf3b1	165.347	169.369	0.0346726	0.8183	0.895142	no
Sf3b2	133.126	125.616	-0.0837746	0.55075	0.698678	no
Sf3b3	52.9517	55.0628	0.0564016	0.6864	0.806383	no
Sf3b4	53.2727	50.3532	-0.0813147	0.57285	0.717099	no
Sf3b5	117.829	107.764	-0.128815	0.3904	0.549467	no
Sfi1	10.1511	8.98579	-0.175922	0.5409	0.690239	no
Sfmbt1	1.78075	1.51922	-0.229157	0.22355	0.365703	no
Sfmbt2	0.669232	1.19669	0.838471	0.0024	0.00784462	yes
Sfn	32.0825	33.6356	0.0682021	0.6542	0.782716	no
Sfpq	171.141	188.226	0.137277	0.34985	0.507202	no
Sfr1	71.2668	70.6635	-0.0122647	0.92935	0.9614	no
Sfswap	25.446	25.407	-0.00221096	0.9868	0.992339	no
Sft2d1	82.7362	74.6259	-0.148842	0.4106	0.569505	no

Sft2d2	17.0911	18.1878	0.0897282	0.5356	0.685944	no
Sft2d3	2.51445	2.44006	-0.043326	0.8488	0.913762	no
Sfxn1	28.0474	29.1332	0.054796	0.7073	0.82111	no
Sfxn2	4.5115	4.06599	-0.149999	0.4231	0.581506	no
Sfxn3	42.1309	38.5585	-0.127831	0.3748	0.5335	no
Sgcb	6.49302	5.35648	-0.277606	0.1042	0.199903	no
Sgk1	97.4997	85.777	-0.184807	0.19915	0.335051	no
Sgk3	2.31906	2.80211	0.272976	0.1439	0.258389	no
Sgms1	19.4662	31.3661	0.688235	5.00E-05	0.000236281	yes
Sgol1	6.65195	3.81276	-0.80294	5.00E-05	0.000236281	yes
Sgol2	4.47954	2.69982	-0.730489	1.00E-04	0.000450026	yes
Sgpl1	19.6934	18.4218	-0.0963002	0.51355	0.666496	no
Sgpp1	24.0945	23.6797	-0.025054	0.8638	0.923153	no
Sgsh	13.3417	17.1614	0.363227	0.0129	0.0341475	yes
Sgsm2	2.0782	2.24377	0.110585	0.57725	0.720894	no
Sgsm3	1.96507	1.98731	0.0162338	0.98735	0.992652	no
Sgta	39.8033	39.7327	-0.00256086	0.9854	0.991539	no
Sh2b1	62.3286	64.1512	0.0415818	0.7714	0.86491	no
Sh2b3	14.3163	15.8922	0.15066	0.3271	0.482505	no
Sh2d1a	185.347	176.532	-0.0703044	0.6265	0.760899	no
Sh2d2a	76.1066	67.5214	-0.172676	0.2199	0.361113	no
Sh2d3c	108.651	99.3562	-0.129015	0.3632	0.521415	no
Sh2d5	1.06193	2.071	0.963636	0.00065	0.00244919	yes
Sh3bgrl	43.4909	43.3882	-0.0034127	0.9817	0.989495	no
Sh3bgrl3	1356.58	1192.3	-0.186223	0.20375	0.340873	no
Sh3bp1	70.3156	72.5519	0.0451668	0.751	0.851219	no
Sh3bp2	9.14032	6.48899	-0.494251	0.004	0.0123078	yes
Sh3bp5	15.3885	26.5106	0.784721	5.00E-05	0.000236281	yes
Sh3bp5l	12.1238	11.5128	-0.0745957	0.63505	0.767782	no
Sh3gl1	85.6929	78.5253	-0.126018	0.4751	0.631445	no
Sh3glb1	46.4318	40.304	-0.20419	0.18225	0.312435	no
Sh3glb2	38.6298	36.6836	-0.0745791	0.6161	0.752867	no
Sh3kbp1	131.515	136.162	0.0501022	0.7294	0.836135	no
Sh3rf1	1.9133	1.66622	-0.199483	0.32955	0.485176	no
Sharpin	54.8093	54.4454	-0.00961218	0.94925	0.972663	no
Shc1	38.0353	37.7307	-0.0115991	0.93525	0.964803	no
Shcbp1	11.8167	6.04519	-0.966973	5.00E-05	0.000236281	yes
Shf	1.67799	1.54854	-0.115832	0.71565	0.826832	no
Shfm1	384.213	371.399	-0.0489371	0.7335	0.839077	no
Shisa5	955.196	1015.75	0.0886696	0.5637	0.709508	no
Shkbp1	41.0356	40.4377	-0.0211752	0.8857	0.936042	no
Shmt1	2.91907	2.73062	-0.096281	0.69235	0.810692	no
Shmt2	31.7526	34.8201	0.133042	0.3582	0.516064	no
Shoc2	15.5592	15.2292	-0.0309254	0.9316	0.962642	no
Shpk	3.67049	3.78008	0.0424439	0.82985	0.902323	no
Shprh	6.50756	6.46404	-0.00968044	0.94955	0.972844	no
Shq1	2.61983	2.8736	0.133383	0.58725	0.729368	no
Siae	5.19376	5.71604	0.138238	0.4371	0.59507	no
Siah1a	11.203	11.4115	0.0266101	0.87125	0.927688	no
Siah1b	2.16269	2.02229	-0.0968411	0.73455	0.839952	no
Siah2	14.8371	15.4031	0.0540102	0.72955	0.836253	no
Sidt1	88.2607	119.083	0.43213	0.00205	0.00681807	yes
Sidt2	78.1366	77.9153	-0.00409224	0.9778	0.987609	no
Sigirr	38.0119	40.9753	0.108304	0.45	0.60743	no
Sigmar1	9.28881	11.8576	0.352241	0.0512	0.110945	no
Sik1	10.1272	12.1918	0.267675	0.0792	0.159257	no
Sik2	6.20119	6.42231	0.0505478	0.8587	0.920085	no

Sik3	18.2729	18.4545	0.0142712	0.91985	0.95631	no
Sike1	55.4431	44.8899	-0.304618	0.03295	0.0763493	no
Sil1	4.63881	5.45839	0.234721	0.2695	0.417265	no
Simc1	1.98517	2.07794	0.0658899	0.86755	0.925479	no
Sin3a	22.2308	21.1361	-0.0728511	0.61065	0.748228	no
Sin3b	120.066	112.834	-0.0896355	0.5841	0.726773	no
Sipa1	129.283	132.528	0.0357622	0.80515	0.887012	no
Sipa1l1	9.34298	13.768	0.559361	1.00E-04	0.000450026	yes
Sipa1l2	1.09691	0.996826	-0.138028	0.54475	0.693497	no
Sipa1l3	5.65813	6.23788	0.140731	0.35385	0.511446	no
Sirt1	16.2088	16.282	0.0065004	0.96425	0.98056	no
Sirt2	77.913	69.9076	-0.156415	0.28725	0.437785	no
Sirt3	13.8076	13.0331	-0.0832871	0.8668	0.924931	no
Sirt4	3.1745	3.22636	0.0233792	0.9254	0.959282	no
Sirt5	2.68966	2.96402	0.140132	0.61035	0.747977	no
Sirt6	15.2215	16.3476	0.102966	0.53875	0.688416	no
Sirt7	117.064	117.232	0.00207472	0.98755	0.99274	no
Sit1	38.3187	45.0811	0.234477	0.12475	0.231251	no
Siva1	21.0299	16.8343	-0.321039	0.10345	0.198724	no
Ska1	4.24428	2.37981	-0.834671	1.00E-04	0.000450026	yes
Ska2	16.4342	13.4746	-0.286458	0.10525	0.201529	no
Ska3	7.8421	4.34691	-0.85125	5.00E-05	0.000236281	yes
Skap1	126.682	129.638	0.0332819	0.8124	0.891562	no
Skap2	9.4548	9.17856	-0.042779	0.82235	0.897707	no
Ski	20.9766	22.6421	0.110232	0.4463	0.603972	no
Skil	6.77147	7.93188	0.228194	0.1291	0.237679	no
Skiv2l	54.5332	54.2196	-0.00832033	0.9518	0.974159	no
Skiv2l2	25.5975	23.8295	-0.103258	0.47415	0.630574	no
Skp1a	84.809	92.9498	0.132235	0.3581	0.515972	no
Skp2	1.53435	1.22254	-0.327747	0.338	0.494358	no
Sla	193.188	157.525	-0.294426	0.0403	0.0906108	no
Sla2	45.3011	48.6153	0.101866	0.54525	0.693846	no
Slain1	29.663	27.9165	-0.0875427	0.5531	0.700664	no
Slain2	25.0318	26.7203	0.0941706	0.50645	0.660293	no
Slamf1	15.1251	16.3798	0.114973	0.45015	0.607586	no
Slamf6	47.9473	65.7266	0.455029	0.00115	0.00407292	yes
Slamf7	83.57	71.0894	-0.23335	0.0954	0.185947	no
Slbp	68.3913	52.3592	-0.38537	0.00675	0.0194405	yes
Slc10a3	12.7953	11.6155	-0.139563	0.5803	0.723525	no
Slc10a3-ubl4	14.5445	12.352	-0.235729	0.4936	0.648366	no
Slc10a7	9.03923	8.98962	-0.00793978	0.96065	0.978873	no
Slc11a2	15.387	25.8505	0.748485	5.00E-05	0.000236281	yes
Slc12a2	1.80317	2.00982	0.156533	0.40095	0.55995	no
Slc12a4	11.7341	10.8347	-0.11505	0.45225	0.609756	no
Slc12a6	20.7322	20.5265	-0.0143855	0.92685	0.960151	no
Slc12a7	31.7842	29.3254	-0.116161	0.41035	0.569315	no
Slc12a9	7.87002	7.69282	-0.0328543	0.8385	0.907423	no
Slc14a1	5.50414	5.87239	0.0934296	0.59065	0.73211	no
Slc15a2	31.1022	20.5171	-0.60019	5.00E-05	0.000236281	yes
Slc15a3	1.44866	1.87483	0.372036	0.1846	0.315597	no
Slc15a4	9.70985	9.08181	-0.0964691	0.56025	0.706678	no
Slc16a1	8.88093	10.9535	0.302605	0.05165	0.111748	no
Slc16a10	1.59332	2.30127	0.53039	0.0159	0.0408449	yes
Slc16a13	2.20551	1.9505	-0.17727	0.47335	0.629783	no
Slc16a3	2.66333	2.42175	-0.137185	0.5483	0.696596	no
Slc16a6	18.6308	18.361	-0.021039	0.88995	0.93838	no
Slc17a5	4.30405	4.54134	0.0774217	0.6766	0.799206	no

Slc17a9	26.1239	38.3384	0.553422	2.00E-04	0.0008488	yes
Slc19a1	3.9634	4.59035	0.211864	0.29515	0.446671	no
Slc19a2	2.79837	2.24418	-0.318397	0.12595	0.233047	no
Slc1a5	145.559	160.084	0.137222	0.3337	0.489631	no
Slc20a1	166.423	145.081	-0.197997	0.1759	0.303577	no
Slc20a2	4.12315	3.77455	-0.127443	0.60825	0.746168	no
Slc22a5	7.87076	7.16377	-0.135786	0.4247	0.583103	no
Slc23a2	11.0726	9.32956	-0.247118	0.09365	0.183123	no
Slc25a1	9.52594	8.76043	-0.12086	0.51075	0.664022	no
Slc25a10	2.13	1.86182	-0.194137	0.3664	0.524792	no
Slc25a11	57.994	54.8491	-0.0804357	0.5749	0.718824	no
Slc25a12	28.1251	25.3633	-0.149118	0.303	0.455659	no
Slc25a13	1.74247	1.775	0.0266805	0.9088	0.949752	no
Slc25a14	5.92455	5.09886	-0.216532	0.3147	0.468532	no
Slc25a15	2.24839	2.56675	0.191052	0.3601	0.518114	no
Slc25a16	9.27778	9.14529	-0.0207519	0.8972	0.942971	no
Slc25a17	31.1832	29.2717	-0.0912624	0.5502	0.698193	no
Slc25a19	15.9557	17.3158	0.118013	0.4494	0.606806	no
Slc25a20	20.2477	19.504	-0.0539857	0.73265	0.83858	no
Slc25a22	13.5498	15.0104	0.147683	0.34515	0.502038	no
Slc25a23	1.75284	1.89166	0.10996	0.64585	0.776106	no
Slc25a24	29.0318	32.5683	0.165832	0.24345	0.388338	no
Slc25a25	6.49703	6.83164	0.0724511	0.69935	0.815556	no
Slc25a26	2.51597	2.75619	0.131559	0.6473	0.777227	no
Slc25a27	0.979256	1.3347	0.446755	0.132	0.242071	no
Slc25a28	16.4975	16.1964	-0.0265764	0.87555	0.930232	no
Slc25a29	3.44026	3.38482	-0.0234381	0.9214	0.957217	no
Slc25a3	230.049	217.732	-0.0793872	0.5795	0.722795	no
Slc25a30	20.1796	19.5631	-0.0447609	0.7808	0.871077	no
Slc25a32	9.57549	9.13454	-0.0680138	0.67865	0.800728	no
Slc25a33	11.5833	15.7884	0.44682	0.00795	0.022437	yes
Slc25a35	3.90072	3.41284	-0.192766	0.4425	0.600337	no
Slc25a36	11.4668	12.5963	0.135533	0.3847	0.543743	no
Slc25a37	12.8487	15.4226	0.263425	0.0788	0.158543	no
Slc25a38	27.7158	28.1282	0.0213094	0.8909	0.938922	no
Slc25a39	27.5058	27.8626	0.0185941	0.90185	0.945774	no
Slc25a4	204.701	202.553	-0.0152229	0.91325	0.952493	no
Slc25a40	16.1787	15.7737	-0.036575	0.80655	0.887792	no
Slc25a42	1.87979	2.10274	0.161698	0.49035	0.64537	no
Slc25a44	12.6856	11.9619	-0.0847382	0.58555	0.727859	no
Slc25a45	47.6408	45.3219	-0.0719898	0.62185	0.757245	no
Slc25a46	18.3788	18.1633	-0.0170177	0.9088	0.949752	no
Slc25a5	415.574	390.116	-0.0912023	0.52685	0.678158	no
Slc25a51	30.5458	27.8551	-0.133034	0.34915	0.506457	no
Slc25a53	11.1688	10.776	-0.0516547	0.8143	0.892523	no
Slc26a10	1.42768	1.51194	0.0827303	0.7752	0.86749	no
Slc26a11	12.3885	22.2324	0.843667	5.00E-05	0.000236281	yes
Slc26a2	3.16308	3.40759	0.107418	0.56605	0.711425	no
Slc26a6	3.80286	4.21926	0.149904	0.4304	0.588708	no
Slc27a1	1.22695	1.95873	0.674836	0.01225	0.0326559	yes
Slc27a3	1.04182	0.656542	-0.666142	0.06535	0.135915	no
Slc27a4	14.891	13.1193	-0.182754	0.2251	0.367608	no
Slc28a2	54.2772	48.0227	-0.176629	0.2149	0.355174	no
Slc29a1	33.5829	25.4188	-0.401834	0.007	0.0200761	yes
Slc29a3	6.70186	5.84443	-0.197498	0.22025	0.361536	no
Slc29a4	1.81537	2.11914	0.223219	0.36065	0.518725	no
Slc2a1	29.1067	31.021	0.0918905	0.5254	0.676934	no

Slc2a3	46.7624	47.2934	0.0162909	0.9112	0.951255	no
Slc2a8	2.82326	3.92224	0.474314	0.04085	0.0916489	no
Slc2a9	2.22254	2.41715	0.121101	0.5589	0.705605	no
Slc30a1	5.1267	5.10131	-0.00716218	0.96305	0.980074	no
Slc30a4	2.56647	3.49224	0.444367	0.0121	0.0323073	yes
Slc30a5	20.1394	20.341	0.0143722	0.92335	0.958227	no
Slc30a6	8.6171	8.15252	-0.0799562	0.6498	0.779129	no
Slc30a7	7.02049	6.92106	-0.0205788	0.8903	0.938598	no
Slc30a9	22.749	22.1804	-0.0365154	0.7973	0.882054	no
Slc31a1	3.6709	3.37021	-0.123297	0.503	0.65713	no
Slc31a2	3.43918	3.43083	-0.00350378	0.9877	0.992848	no
Slc33a1	10.419	9.1639	-0.185178	0.24985	0.395366	no
Slc35a1	9.40702	9.93544	0.0788455	0.6533	0.782023	no
Slc35a2	12.9073	11.5229	-0.163683	0.32305	0.478059	no
Slc35a3	13.6099	13.0754	-0.0578079	0.69815	0.814789	no
Slc35a4	28.4994	27.4831	-0.0523846	0.7466	0.848329	no
Slc35a5	4.39319	3.83795	-0.194932	0.2772	0.426362	no
Slc35b1	41.4952	42.9892	0.0510287	0.7389	0.843136	no
Slc35b2	13.947	13.7421	-0.0213518	0.8934	0.940434	no
Slc35b3	19.6917	20.0846	0.0285049	0.8574	0.919376	no
Slc35b4	12.0746	11.9559	-0.0142524	0.9254	0.959282	no
Slc35c1	38.6482	43.4297	0.168278	0.2453	0.390416	no
Slc35c2	23.3484	24.7067	0.081577	0.596	0.736491	no
Slc35d1	3.33888	3.69975	0.148062	0.4617	0.618797	no
Slc35d2	17.334	14.8297	-0.22511	0.15565	0.275354	no
Slc35e1	15.9815	17.6709	0.144967	0.31055	0.464096	no
Slc35e2	4.50584	4.33761	-0.0548959	0.741	0.844386	no
Slc35e3	6.33022	6.30956	-0.0047162	0.9777	0.987578	no
Slc35e4	3.3487	1.86016	-0.848171	2.00E-04	0.0008488	yes
Slc35f2	7.46644	8.01871	0.10295	0.5494	0.69758	no
Slc35f6	4.80003	4.82244	0.00672038	0.9699	0.983692	no
Slc35g1	8.18504	8.69231	0.0867499	0.59525	0.735925	no
Slc36a1	12.3104	12.3453	0.00409189	0.977	0.987224	no
Slc36a4	1.14796	1.28916	0.167364	0.6164	0.75309	no
Slc37a1	30.4632	30.7415	0.0131172	0.9303	0.961904	no
Slc37a2	1.04624	0.607923	-0.783254	0.0067	0.0193115	yes
Slc37a3	10.5395	11.4713	0.122221	0.4328	0.59104	no
Slc37a4	15.845	15.6206	-0.0205796	0.8977	0.943244	no
Slc38a1	26.127	28.1855	0.109412	0.4416	0.599485	no
Slc38a10	37.467	36.5358	-0.0363116	0.80635	0.88771	no
Slc38a2	32.7658	30.3356	-0.111176	0.42725	0.585636	no
Slc38a6	3.59851	4.04684	0.169393	0.38075	0.539749	no
Slc38a7	4.40237	4.48341	0.0263146	0.8881	0.937245	no
Slc38a9	4.34633	4.25207	-0.03163	0.8369	0.906539	no
Slc39a1	19.1394	18.9439	-0.0148163	0.92095	0.957003	no
Slc39a10	4.46833	4.64642	0.0563828	0.741	0.844386	no
Slc39a11	3.52388	4.82131	0.45226	0.02015	0.0501845	no
Slc39a13	6.0362	5.83226	-0.0495849	0.79745	0.882095	no
Slc39a14	3.0489	3.492	0.195764	0.2766	0.425634	no
Slc39a3	7.83119	7.56319	-0.0502381	0.76095	0.857881	no
Slc39a4	6.18787	7.31662	0.241734	0.1897	0.322613	no
Slc39a6	15.9362	18.499	0.215141	0.145	0.25998	no
Slc39a7	62.0295	61.0407	-0.023182	0.86675	0.924919	no
Slc39a9	14.0648	13.6739	-0.0406628	0.7795	0.870328	no
Slc3a2	51.8186	56.1632	0.116154	0.41905	0.577683	no
Slc41a1	8.17675	8.01192	-0.0293809	0.85335	0.916732	no
Slc43a1	2.3865	3.34127	0.485499	0.03085	0.0722327	no

Slc43a2	9.49643	10.1005	0.0889641	0.54425	0.693135	no
Slc43a3	10.5032	7.41236	-0.50283	0.00345	0.0108043	yes
Slc44a1	1.79899	2.36108	0.392261	0.13495	0.246252	no
Slc44a2	155.588	132.958	-0.226756	0.10955	0.208327	no
Slc45a4	4.81648	4.71485	-0.0307678	0.85935	0.920307	no
Slc46a3	10.9169	10.2486	-0.0911296	0.5889	0.730698	no
Slc48a1	17.684	15.6271	-0.17839	0.74565	0.847673	no
Slc4a1ap	15.5112	16.2105	0.0636213	0.6848	0.805334	no
Slc4a2	19.4069	18.2349	-0.0898641	0.54335	0.692451	no
Slc4a7	16.4698	20.4496	0.312252	0.02885	0.068348	no
Slc4a8	1.71131	1.42458	-0.264565	0.12775	0.235716	no
Slc50a1	102.721	102.02	-0.00987814	0.94545	0.970455	no
Slc52a2	6.23512	6.26108	0.00599413	0.9711	0.984179	no
Slc52a3	2.68771	2.32488	-0.209221	0.3683	0.526883	no
Slc5a6	2.7044	2.19169	-0.303261	0.86275	0.92238	no
Slc6a12	1.30422	1.617	0.310135	0.26125	0.407279	no
Slc6a13	379.971	637.913	0.747469	5.00E-05	0.000236281	yes
Slc6a6	12.2671	11.3247	-0.115317	0.42985	0.588138	no
Slc7a1	7.70785	7.5321	-0.0332767	0.8204	0.896508	no
Slc7a5	7.43286	7.3312	-0.0198679	0.9017	0.945645	no
Slc7a6	15.1699	16.4103	0.113396	0.4499	0.607341	no
Slc7a6os	34.4569	37.0969	0.106505	0.4819	0.637505	no
Slc8b1	10.9342	11.8865	0.120473	0.45	0.60743	no
Slc9a1	20.427	20.7027	0.0193441	0.88935	0.937989	no
Slc9a3r1	195.879	177.063	-0.145696	0.30315	0.455807	no
Slc9a6	5.82325	4.35955	-0.417645	0.0122	0.0325386	yes
Slc9a7	13.5129	13.2644	-0.0267772	0.86845	0.926062	no
Slc9a8	13.4822	13.4989	0.00177898	0.98995	0.993893	no
Slc9a9	19.3202	25.9657	0.426496	0.0035	0.010941	yes
Slco3a1	57.5483	55.5982	-0.0497351	0.7328	0.838711	no
Slco4a1	1.82126	1.95972	0.10571	0.6629	0.789178	no
Slfn1	46.7229	30.1165	-0.633579	5.00E-05	0.000236281	yes
Slfn2	206.248	195.235	-0.0791695	0.57365	0.717782	no
Slfn3	5.33928	4.09413	-0.383087	0.0762	0.154209	no
Slfn5	2.76973	4.91578	0.827674	5.00E-05	0.000236281	yes
Slfn8	17.0238	18.161	0.0932861	0.53135	0.682147	no
Slfn9	3.58157	2.90565	-0.301731	0.1109	0.210401	no
Slirp	59.0704	54.2566	-0.122637	0.82035	0.896495	no
Slk	33.9916	34.2079	0.00914766	0.94555	0.970464	no
Slmap	18.5512	19.8124	0.0948939	0.5092	0.66269	no
Slmo2	44.6225	42.6289	-0.0659406	0.66195	0.78854	no
Sltm	50.3497	47.2409	-0.0919493	0.51275	0.665813	no
Slu7	34.4311	31.4816	-0.129206	0.3711	0.529654	no
Slx1b	3.36518	3.07983	-0.127833	0.5074	0.661043	no
Slx4	11.8898	11.5172	-0.045934	0.74965	0.850399	no
Slx4ip	3.70199	3.70843	0.00250445	0.99375	0.996079	no
Smad1	2.95892	3.88204	0.391744	0.0504	0.109514	no
Smad2	8.49458	7.89231	-0.106095	0.4664	0.623262	no
Smad3	51.6275	46.251	-0.158656	0.25995	0.405637	no
Smad4	34.892	34.9848	0.00383016	0.97815	0.987778	no
Smad5	4.44515	4.81592	0.115579	0.4693	0.625928	no
Smad7	22.9496	27.531	0.26259	0.06745	0.139379	no
Smap1	82.7655	81.8359	-0.0162961	0.9104	0.950781	no
Smap2	157.432	189.04	0.263963	0.06515	0.135551	no
Smarca2	23.5999	23.5093	-0.00555226	0.9723	0.984802	no
Smarca4	35.7726	34.4884	-0.0527443	0.7136	0.825504	no
Smarca5	39.8419	37.7605	-0.0774109	0.58	0.723253	no

Smarcad1	5.83737	5.8722	0.00858372	0.95575	0.976347	no
Smarcal1	4.80077	4.79786	-0.000877201	0.9963	0.997331	no
Smarcb1	40.7089	38.6781	-0.0738278	0.62185	0.757245	no
Smarcc1	14.0188	16.4152	0.227662	0.11315	0.213745	no
Smarcc2	36.4099	35.2914	-0.0450145	0.75685	0.854993	no
Smarcd1	19.7633	18.8841	-0.0656493	0.6557	0.78378	no
Smarcd2	102.6	105.767	0.0438492	0.7602	0.857351	no
Smarce1	55.7083	56.5887	0.0226226	0.8716	0.92785	no
Smc1a	57.7274	55.3034	-0.0618872	0.6584	0.785796	no
Smc2	21.8014	15.2078	-0.519613	0.00015	0.000653255	yes
Smc3	39.0035	35.7351	-0.126261	0.3753	0.533986	no
Smc4	82.1307	74.6338	-0.138092	0.32175	0.476635	no
Smc5	17.2141	15.6376	-0.138572	0.33455	0.490511	no
Smc6	34.3436	32.0548	-0.0994997	0.48155	0.637174	no
Smchd1	36.3337	32.6692	-0.153378	0.2765	0.425527	no
Smco4	8.52932	16.1087	0.917336	5.00E-05	0.000236281	yes
Smcr8	12.7935	13.224	0.0477432	0.74315	0.845934	no
Smdt1	205.744	196.966	-0.0629031	0.66435	0.790038	no
Smek1	40.6714	42.4883	0.06305	0.6832	0.803975	no
Smek2	46.185	46.4463	0.0081367	0.9547	0.975769	no
Smg1	47.5299	56.4957	0.249306	0.0907	0.178382	no
Smg5	37.5555	37.15	-0.0156603	0.91365	0.952699	no
Smg6	15.7998	16.4115	0.0547965	0.70535	0.819722	no
Smg7	26.5687	25.3287	-0.0689566	0.63325	0.766367	no
Smg8	9.1431	8.84314	-0.0481246	0.76645	0.861509	no
Smg9	30.6071	29.3592	-0.0600533	0.6827	0.803655	no
Smim1	8.6114	8.62654	0.00253467	0.9949	0.996594	no
Smim11	68.9792	64.7068	-0.0922454	0.5952	0.735889	no
Smim12	21.063	22.1333	0.0715062	0.68125	0.802671	no
Smim13	2.4331	2.64671	0.121406	0.53585	0.686139	no
Smim14	29.2004	27.2427	-0.100123	0.5049	0.658941	no
Smim15	53.2711	47.2218	-0.1739	0.2299	0.373244	no
Smim19	14.7217	14.3224	-0.039664	0.8596	0.92049	no
Smim20	41.0788	36.2792	-0.179254	0.2715	0.419551	no
Smim3	9.51662	9.98895	0.0698838	0.68545	0.805688	no
Smim7	21.8951	21.4207	-0.0316008	0.8322	0.903735	no
Smim8	21.4779	20.1366	-0.0930313	0.6055	0.744126	no
Smn1	23.0985	22.7716	-0.0205658	0.9046	0.947419	no
Smndc1	65.5667	62.985	-0.0579543	0.6825	0.80358	no
Smox	4.20995	5.25947	0.321112	0.1344	0.245503	no
Smpd1	30.1252	28.8312	-0.0633373	0.6683	0.793065	no
Smpd2	14.1891	13.4559	-0.0765486	0.66115	0.787959	no
Smpd4	11.7336	12.5208	0.0936786	0.5379	0.687841	no
Smpd5	6.53014	7.00328	0.100916	0.6603	0.787291	no
Smpdl3a	74.6374	74.2393	-0.0077149	0.9591	0.977972	no
Smpdl3b	37.6808	20.5492	-0.87475	5.00E-05	0.000236281	yes
Sms	41.6938	44.2989	0.0874397	0.5383	0.688066	no
Smtn	3.86156	2.90531	-0.410492	0.0358	0.0819417	no
Smu1	39.3737	37.7576	-0.0604641	0.8352	0.905615	no
Smug1	2.86251	2.60626	-0.135297	0.50425	0.658275	no
Smurf1	9.2349	8.08739	-0.191421	0.2056	0.343301	no
Smurf2	7.2973	6.11153	-0.255829	0.1053	0.201608	no
Smyd1	4.83848	4.25437	-0.18561	0.3077	0.460939	no
Smyd2	3.04723	4.18123	0.456427	0.06285	0.131767	no
Smyd3	3.05741	3.41014	0.157524	0.40805	0.566937	no
Smyd4	3.71381	3.53983	-0.0692185	0.70975	0.822804	no
Smyd5	4.01919	5.10658	0.345452	0.0786	0.158213	no

Snai3	17.9061	23.5981	0.398219	0.01425	0.0371649	yes
Snap23	21.0203	20.8262	-0.0133899	0.9291	0.961226	no
Snap29	19.3084	16.9662	-0.186564	0.4979	0.652242	no
Snap47	7.80508	6.75379	-0.208716	0.27615	0.425118	no
Snapc1	12.896	13.3796	0.0531129	0.74505	0.847264	no
Snapc2	14.1924	12.7149	-0.158598	0.3747	0.533444	no
Snapc3	19.4877	17.7714	-0.133004	0.43285	0.591085	no
Snapc4	9.38358	10.9725	0.225682	0.14035	0.253569	no
Snapc5	80.4304	74.2421	-0.115503	0.4427	0.600551	no
Snapin	27.4431	25.9962	-0.0781457	0.7082	0.821722	no
Snd1	41.8114	43.5915	0.0601503	0.67025	0.794631	no
Snf8	87.9634	83.8896	-0.0684101	0.6431	0.773988	no
Snip1	25.8414	24.96	-0.0500668	0.74395	0.846545	no
Snrk	21.185	24.4997	0.209722	0.14335	0.257631	no
Snrnp200	46.3296	46.2513	-0.00243976	0.9864	0.992008	no
Snrnp25	25.1066	23.5083	-0.0948962	0.5983	0.738389	no
Snrnp27	49.7189	47.7905	-0.0570689	0.72055	0.830278	no
Snrnp35	4.90783	4.58115	-0.0993742	0.6984	0.81496	no
Snrnp40	55.6888	53.1577	-0.0671091	0.63775	0.769754	no
Snrnp48	30.7871	30.0405	-0.0354185	0.81435	0.892536	no
Snrnp70	212.626	211.226	-0.00953215	0.94705	0.971396	no
Snrpa	51.2532	47.2106	-0.11853	0.44775	0.605168	no
Snrpa1	36.7135	32.848	-0.160501	0.3024	0.454989	no
Snrpb	212.511	203.384	-0.0633313	0.6469	0.776945	no
Snrpb2	68.7039	68.8528	0.00312274	0.9815	0.989434	no
Snrpc	105.694	91.6821	-0.205186	0.17135	0.297411	no
Snrpd1	75.502	60.2727	-0.32501	0.03435	0.0790638	no
Snrpd2	277.505	256.201	-0.115238	0.42535	0.583769	no
Snrpd3	226.332	220.733	-0.0361403	0.80515	0.887012	no
Snrpe	384.936	353.076	-0.12464	0.3861	0.545111	no
Snrpf	87.9343	89.8625	0.0312924	0.8421	0.90977	no
Snrpg	631.631	587.606	-0.104233	0.46055	0.617772	no
Sntb1	13.1938	19.613	0.571947	0.00025	0.00103712	yes
Sntb2	17.4454	23.2107	0.41194	0.00405	0.012441	yes
Snupn	8.19286	8.63919	0.076529	0.70495	0.819514	no
Snw1	59.7639	59.2769	-0.0118059	0.94145	0.968284	no
Snx1	59.9026	59.1531	-0.018164	0.89845	0.943765	no
Snx10	56.0968	35.2253	-0.671307	5.00E-05	0.000236281	yes
Snx11	15.4186	13.872	-0.152489	0.328	0.483549	no
Snx12	31.8772	29.0293	-0.135015	0.4406	0.598519	no
Snx13	5.09371	5.42931	0.0920509	0.5601	0.70659	no
Snx14	27.7781	30.1497	0.118196	0.4111	0.569974	no
Snx15	12.9896	12.8779	-0.0124557	0.9449	0.970135	no
Snx16	1.57219	2.08773	0.409162	0.1056	0.202062	no
Snx17	57.8774	55.326	-0.0650422	0.8869	0.936623	no
Snx18	8.88403	6.54008	-0.441906	0.006	0.0175533	yes
Snx19	12.1718	12.8167	0.0744876	0.6127	0.749932	no
Snx2	57.7832	56.2572	-0.0386112	0.79085	0.877973	no
Snx20	36.6317	33.92	-0.110954	0.46925	0.625874	no
Snx21	1.37014	1.40808	0.0394045	0.9471	0.971419	no
Snx22	47.0363	46.0705	-0.0299304	0.89975	0.944512	no
Snx25	15.1465	14.6977	-0.0433921	0.77805	0.869301	no
Snx27	12.5136	11.2899	-0.148467	0.31305	0.466774	no
Snx29	1.87721	1.75542	-0.09678	0.70455	0.819184	no
Snx3	112.913	94.7195	-0.253475	0.07495	0.152072	no
Snx30	1.63351	1.55403	-0.0719635	0.7038	0.818541	no
Snx32	6.46008	7.44391	0.204508	0.3117	0.465361	no

Snx33	4.00217	3.48018	-0.201619	0.2591	0.404705	no
Snx4	80.8727	84.384	0.0613168	0.66285	0.789178	no
Snx5	200.813	174.323	-0.204087	0.1583	0.279196	no
Snx6	53.131	51.1354	-0.0552316	0.69795	0.814691	no
Snx8	1.27016	1.57265	0.308181	0.26265	0.408983	no
Snx9	2.0645	3.19035	0.62792	0.01385	0.0362772	yes
Soat1	5.72312	7.76284	0.439783	0.00645	0.0186894	yes
Soat2	16.0632	15.9963	-0.00602906	0.96915	0.98329	no
Socs1	8.23612	11.5049	0.482217	0.0167	0.0426207	yes
Socs2	1.51198	1.22866	-0.299355	0.30695	0.46007	no
Socs3	9.13774	17.4203	0.93086	5.00E-05	0.000236281	yes
Socs4	13.9299	12.9192	-0.10867	0.50755	0.661177	no
Socs5	0.692984	1.10301	0.670558	0.01585	0.0407327	yes
Socs6	6.14591	6.21588	0.0163331	0.9269	0.960189	no
Socs7	5.58152	5.48929	-0.0240389	0.8792	0.932366	no
Sod1	320.98	286.277	-0.165076	0.2423	0.387064	no
Sod2	10.6134	14.8214	0.481804	0.00185	0.00622713	yes
Son	74.7646	76.7328	0.037488	0.79475	0.880461	no
Sorbs1	6.02648	5.72488	-0.0740684	0.6364	0.768782	no
Sorbs3	1.50022	1.72372	0.200351	0.4571	0.614639	no
Sord	7.76403	2.89436	-1.42356	5.00E-05	0.000236281	yes
Sorl1	43.69	52.2191	0.257276	0.0697	0.143306	no
Sos1	5.84277	5.50337	-0.0863391	0.5669	0.712088	no
Sos2	11.6983	13.0713	0.160114	0.27485	0.423578	no
Sowahc	1.57193	1.62146	0.0447569	0.8405	0.908779	no
Sp1	26.0199	26.8391	0.0447215	0.7539	0.852971	no
Sp100	412.834	333.16	-0.309348	0.03265	0.0757811	no
Sp110	202.003	176.021	-0.198626	0.21445	0.354638	no
Sp140	43.956	34.2118	-0.361564	0.23825	0.383074	no
Sp2	34.4862	30.5895	-0.172985	0.23335	0.377353	no
Sp3	53.0542	49.3156	-0.105423	0.45925	0.616579	no
Sp4	18.336	18.2407	-0.00751445	0.9563	0.97657	no
Spag1	4.37815	2.93233	-0.578271	0.00465	0.0140505	yes
Spag5	14.3863	8.29559	-0.794278	5.00E-05	0.000236281	yes
Spag7	45.3625	38.7601	-0.226926	0.1422	0.256157	no
Spag9	12.2076	11.4972	-0.0865053	0.5485	0.696775	no
Spast	15.8853	14.3763	-0.144008	0.328	0.483549	no
Spata1	2.59624	2.13056	-0.285187	0.4396	0.597586	no
Spata13	66.8863	59.7109	-0.163717	0.253	0.397349	no
Spata2	14.0208	13.3245	-0.0734875	0.8123	0.891508	no
Spata24	3.29811	3.26777	-0.0133346	0.97175	0.984428	no
Spata2l	3.58856	3.63197	0.0173486	0.95945	0.978173	no
Spata5	9.84533	9.23619	-0.0921413	0.57205	0.71639	no
Spata5l1	16.702	19.561	0.22796	0.18515	0.316361	no
Spata6	18.0657	19.2618	0.0924886	0.5484	0.696673	no
Spc24	20.5324	11.0671	-0.89162	5.00E-05	0.000236281	yes
Spc25	18.5081	9.42697	-0.973293	5.00E-05	0.000236281	yes
Spcs1	90.9283	91.0036	0.00119463	0.9932	0.995754	no
Spcs2	53.2945	51.3205	-0.054452	0.6981	0.814744	no
Spcs3	38.9323	36.5719	-0.09023	0.529	0.680194	no
Spdl1	4.398	2.99678	-0.553434	0.01155	0.0310715	yes
Specc1	1.0029	0.806013	-0.315309	0.1679	0.292589	no
Specc1l	12.7206	13.9503	0.133132	0.35775	0.515657	no
Spef1	2.31761	1.95834	-0.243006	0.3367	0.492875	no
Spef2	1.01261	1.57357	0.635971	0.0219	0.0539961	no
Spen	13.0058	14.3621	0.143118	0.3697	0.528275	no
Spg11	11.9564	11.7089	-0.0301887	0.88295	0.934535	no

Spg20	3.83093	4.21425	0.137579	0.4555	0.613095	no
Spg21	33.7727	30.2391	-0.159444	0.2748	0.42352	no
Spg7	31.5868	32.7751	0.0532791	0.71855	0.828704	no
Sphk2	12.99	14.0368	0.111814	0.48155	0.637174	no
Spice1	17.2234	15.7967	-0.124749	0.3934	0.552488	no
Spidr	4.58395	4.35521	-0.073849	0.68755	0.807196	no
Spin1	16.8352	16.2528	-0.0507945	0.7202	0.829997	no
Spin2c	1.693	1.76819	0.0626926	0.85315	0.916587	no
Spint2	20.0895	38.051	0.921494	5.00E-05	0.000236281	yes
Spn	418.202	353.477	-0.242583	0.13755	0.24977	no
Spns1	20.3562	19.4673	-0.0644145	0.6717	0.795562	no
Spop	42.3754	44.8439	0.0816846	0.56745	0.712487	no
Spopl	6.26883	6.00432	-0.0621942	0.73015	0.836709	no
Sppl2a	24.7149	22.8078	-0.115856	0.4123	0.571111	no
Sppl2b	15.4371	16.6747	0.111261	0.4713	0.627789	no
Sppl3	47.0103	42.3818	-0.14953	0.2991	0.451236	no
Spr	20.9114	25.7265	0.298965	0.0704	0.144493	no
Spred2	2.8569	3.70139	0.373615	0.07465	0.151542	no
Sprtn	13.4947	13.4976	0.000311288	0.99675	0.997655	no
Spry2	4.60413	5.3468	0.215748	0.28365	0.43374	no
Spryd3	32.3879	33.8354	0.0630796	0.66305	0.78925	no
Spryd4	4.77812	4.24725	-0.169916	0.4525	0.610058	no
Spryd7	5.69961	6.81037	0.25687	0.2067	0.344754	no
Spsb2	3.57323	3.14312	-0.185029	0.78235	0.872213	no
Spsb3	46.3842	47.1951	0.0250029	0.8661	0.924505	no
Sptan1	79.7386	81.708	0.0351996	0.809	0.889504	no
Sptbn1	35.9525	38.3039	0.091399	0.5135	0.66648	no
Sptlc1	25.0074	25.5663	0.0318856	0.8245	0.898984	no
Sptlc2	31.1594	28.7161	-0.117809	0.41225	0.571075	no
Sptssa	55.4837	50.8423	-0.126036	0.38595	0.544964	no
Spty2d1	26.311	26.0875	-0.0123024	0.9305	0.96204	no
Sqle	6.92748	7.1531	0.0462367	0.79545	0.880835	no
Sqstm1	100.243	102.662	0.034401	0.8063	0.887697	no
Sra1	62.1658	57.0054	-0.125022	0.4105	0.569445	no
Srbd1	11.9386	9.97158	-0.259746	0.0962	0.187269	no
Srcin1	1.18807	0.168012	-2.82198	5.00E-05	0.000236281	yes
Srd5a3	30.5515	29.5426	-0.0484477	0.7504	0.850866	no
Srebf1	28.5389	30.1792	0.0806288	0.66635	0.79163	no
Srebf2	51.7563	61.5846	0.250835	0.075	0.152156	no
Srek1	12.1526	12.04	-0.0134277	0.9295	0.961471	no
Srek1ip1	7.84926	8.12755	0.0502639	0.78635	0.874932	no
Srf	46.8825	48.5231	0.049622	0.72525	0.833395	no
Srfbp1	14.5146	15.7547	0.118282	0.5026	0.656704	no
Srgap2	13.5274	13.2334	-0.0317009	0.82085	0.89675	no
Srgn	564.31	555.448	-0.0228347	0.8702	0.927017	no
Sri	114.252	114.746	0.00621431	0.9633	0.980229	no
Srm	11.4653	13.2054	0.203851	0.2651	0.411971	no
Srp14	180.606	172.08	-0.0697623	0.62605	0.760575	no
Srp19	111.711	94.7043	-0.238269	0.1022	0.196772	no
Srp54a	1.20594	1.14754	-0.0716113	0.7779	0.869229	no
Srp54b	1.54571	1.67506	0.115948	0.75245	0.852029	no
Srp54c	6.7409	6.06883	-0.151524	0.45775	0.615197	no
Srp68	56.2403	54.0197	-0.0581182	0.6783	0.800478	no
Srp72	53.1742	50.8449	-0.064624	0.64665	0.776737	no
Srp9	82.2022	74.8443	-0.135285	0.3443	0.501163	no
Srpk1	82.206	108.021	0.393998	0.00575	0.0169187	yes
Srpk2	19.167	20.403	0.090155	0.7397	0.843531	no

Srpr	57.1084	53.7273	-0.0880488	0.5541	0.701528	no
Srprb	14.9434	15.1708	0.0217888	0.88905	0.937855	no
Srr	6.81713	7.1391	0.0665757	0.78055	0.870963	no
Srrd	17.6788	16.5634	-0.0940177	0.7834	0.872957	no
Srrm1	66.8001	66.886	0.0018552	0.98955	0.993761	no
Srrm2	114.438	122.3	0.0958522	0.5356	0.685944	no
Srrt	71.7959	75.2147	0.0671136	0.6354	0.768008	no
Srsf1	53.2161	47.8403	-0.153636	0.2783	0.427709	no
Srsf10	51.6774	46.9714	-0.13775	0.33275	0.488481	no
Srsf11	75.7401	74.5225	-0.0233809	0.8705	0.927099	no
Srsf2	153.749	143.096	-0.103589	0.4653	0.622275	no
Srsf3	131.784	121.901	-0.112466	0.4336	0.591846	no
Srsf4	16.9658	14.9947	-0.178176	0.48715	0.642368	no
Srsf5	405.763	410.445	0.0165504	0.91205	0.951748	no
Srsf6	84.9043	88.7622	0.0641088	0.64735	0.777247	no
Srsf7	93.6836	87.5659	-0.0974284	0.49275	0.64749	no
Srsf9	38.5138	36.5186	-0.0767418	0.6198	0.755739	no
Ss18	57.7687	57.7179	-0.00126912	0.9927	0.99545	no
Ss18l1	1.16233	1.55836	0.423008	0.074	0.150438	no
Ssb	121.458	115.194	-0.0763834	0.59845	0.738535	no
Ssbp1	29.1953	28.3408	-0.042857	0.79365	0.87967	no
Ssbp3	30.7941	40.0968	0.380832	0.0067	0.0193115	yes
Ssbp4	78.0099	81.4745	0.0626919	0.65815	0.785591	no
Ssfa2	5.96234	5.6137	-0.0869275	0.5931	0.734205	no
Ssh1	11.0029	9.38631	-0.22926	0.15115	0.268876	no
Ssh2	19.713	28.2801	0.520635	2.00E-04	0.0008488	yes
Ssh3	6.69094	9.57198	0.516609	0.00255	0.00827285	yes
Ssna1	82.2198	77.4243	-0.0866987	0.5682	0.713137	no
Ssr1	16.2882	16.1533	-0.0120022	0.93465	0.964438	no
Ssr2	77.7597	70.5502	-0.140372	0.3372	0.493423	no
Ssr3	83.0525	78.9375	-0.0733119	0.60145	0.741046	no
Ssr4	105.699	100.197	-0.0771354	0.60765	0.745782	no
Ssrp1	97.952	87.6941	-0.159596	0.26315	0.409626	no
Sssca1	16.2329	16.9031	0.0583664	0.7824	0.872255	no
Ssu72	65.1828	58.8843	-0.146608	0.31455	0.468378	no
Ssx2ip	1.55333	1.3849	-0.165586	0.49185	0.646548	no
St13	71.9894	69.9654	-0.0411423	0.77745	0.869002	no
St14	2.27503	4.35454	0.936634	5.00E-05	0.000236281	yes
St3gal1	38.4557	37.9542	-0.0189387	0.89125	0.939137	no
St3gal2	1.04933	0.72384	-0.535719	0.0478	0.104686	no
St3gal3	5.06695	5.08273	0.00448623	0.9805	0.988836	no
St3gal4	52.3352	42.1266	-0.31305	0.117	0.219724	no
St3gal6	110.048	110.76	0.00931438	0.94775	0.97186	no
St6gal1	1.52714	4.18535	1.45451	5.00E-05	0.000236281	yes
St6galnac4	12.1457	11.6994	-0.0540066	0.719	0.829047	no
St6galnac6	3.89173	4.31814	0.149997	0.464	0.621066	no
St7	4.29122	4.79788	0.161008	0.43425	0.592447	no
St7l	5.04187	5.08821	0.0131984	0.9314	0.962533	no
St8sia4	19.7725	19.6844	-0.0064404	0.9709	0.984084	no
Stag1	18.0529	16.4034	-0.138232	0.33485	0.490798	no
Stag2	44.3318	42.6224	-0.0567287	0.695	0.812483	no
Stam	7.27894	7.40692	0.0251463	0.87965	0.932594	no
Stam2	10.7233	10.0977	-0.0867164	0.57815	0.721609	no
Stambp	10.148	10.4129	0.0371759	0.8279	0.901119	no
Stambpl1	11.5121	11.1858	-0.0414783	0.80935	0.889695	no
Stap1	39.1635	41.1519	0.0714487	0.64955	0.778974	no
Star	1.30188	1.61888	0.314395	0.19035	0.32343	no

Stard10	57.3519	51.3308	-0.160017	0.28725	0.437785	no
Stard3	26.947	24.1163	-0.160118	0.29475	0.446228	no
Stard3nl	26.5641	30.4101	0.195073	0.21725	0.358094	no
Stard4	11.5291	10.5565	-0.127154	0.42635	0.584743	no
Stard5	4.18096	4.56434	0.126572	0.5236	0.675404	no
Stard6	1.09104	0.913925	-0.255556	0.74815	0.849325	no
Stard7	31.3678	33.2337	0.0833652	0.5544	0.701769	no
Stat1	63.5024	57.8197	-0.135249	0.3395	0.495935	no
Stat2	17.0222	16.2478	-0.0671738	0.6443	0.774903	no
Stat3	50.2352	49.4523	-0.0226609	0.87655	0.930846	no
Stat4	103.4	100.694	-0.038258	0.79535	0.880808	no
Stat5a	23.4246	24.4905	0.0641988	0.65605	0.783919	no
Stat5b	42.99	52.6573	0.292633	0.03805	0.0862719	no
Stat6	86.8646	90.6714	0.0618795	0.6762	0.798935	no
Stau1	14.9655	12.5812	-0.250368	0.10415	0.199835	no
Steap3	0.824485	1.04117	0.336636	0.27665	0.425674	no
Stil	4.04219	2.30874	-0.808029	5.00E-05	0.000236281	yes
Stim1	51.0018	50.5048	-0.0141274	0.91815	0.95519	no
Stim2	11.4165	12.2417	0.100689	0.50755	0.661177	no
Stip1	69.6893	64.3701	-0.114547	0.4231	0.581506	no
Stk10	106.951	104.753	-0.0299494	0.83655	0.906341	no
Stk11	60.0054	62.4174	0.0568553	0.71125	0.82392	no
Stk11ip	15.8317	16.5462	0.0636812	0.6635	0.789533	no
Stk16	21.7018	23.3688	0.106767	0.6473	0.777227	no
Stk17b	289.357	283.567	-0.0291633	0.8427	0.910112	no
Stk19	30.0349	32.541	0.115621	0.47645	0.632679	no
Stk24	133.566	127.985	-0.061577	0.6615	0.788123	no
Stk25	29.746	29.6788	-0.00326292	0.9803	0.988747	no
Stk32c	7.7043	5.30567	-0.538129	0.00765	0.0217092	yes
Stk35	5.374	5.82073	0.115204	0.4714	0.627887	no
Stk38	110.225	111.883	0.0215453	0.87965	0.932594	no
Stk38l	2.69904	2.65622	-0.0230706	0.90605	0.948291	no
Stk39	14.9936	14.1414	-0.0844199	0.5816	0.724582	no
Stk4	115.628	109.586	-0.0774388	0.58535	0.727687	no
Stk40	18.8299	17.8734	-0.0752067	0.6135	0.750638	no
Stmn1	298.896	161.927	-0.884298	5.00E-05	0.000236281	yes
Stmn2	1.13847	0.953673	-0.255533	0.46825	0.624929	no
Stoml1	9.72094	12.3745	0.348198	0.03715	0.0845382	no
Stoml2	30.2744	31.7635	0.0692726	0.65825	0.78567	no
Stra13	62.6113	52.3249	-0.258925	0.11715	0.219935	no
Strada	17.6894	18.2047	0.0414301	0.78535	0.874329	no
Stradb	8.9485	8.11469	-0.141111	0.4239	0.582231	no
Strap	55.2457	52.9487	-0.0612653	0.662	0.788559	no
Strbp	1.19409	1.43255	0.262678	0.12	0.22423	no
Strip1	23.8988	22.8984	-0.0616904	0.66855	0.793242	no
Strn	14.0905	13.0439	-0.111346	0.4594	0.616698	no
Strn3	10.3465	10.6852	0.0464829	0.80295	0.885653	no
Strn4	31.4078	32.1488	0.033642	0.8356	0.90577	no
Stt3a	54.9932	51.0163	-0.108294	0.45035	0.607809	no
Stt3b	25.0094	24.7712	-0.0138034	0.92385	0.958492	no
Stub1	45.0055	43.1069	-0.0621823	0.7072	0.821075	no
Stx11	19.0931	15.5666	-0.294601	0.05845	0.124135	no
Stx12	15.214	12.9096	-0.236954	0.1376	0.249835	no
Stx16	38.6211	40.3163	0.0619752	0.669	0.793616	no
Stx17	7.36218	7.22096	-0.0279418	0.85795	0.919728	no
Stx18	10.2509	9.8798	-0.0532003	0.7462	0.848025	no
Stx1a	9.02364	10.6422	0.238009	0.167	0.291517	no

Stx2	10.4336	9.04815	-0.205548	0.20385	0.341016	no
Stx4a	75.8622	73.0614	-0.0542705	0.70845	0.821945	no
Stx5a	50.0379	50.6682	0.0180574	0.9001	0.944711	no
Stx6	24.0428	23.9902	-0.00315612	0.9837	0.990506	no
Stx7	12.8689	12.5188	-0.0397944	0.8022	0.885102	no
Stx8	7.47783	7.40164	-0.0147754	0.9307	0.962092	no
Stxbp2	45.5399	44.692	-0.0271142	0.84845	0.913526	no
Stxbp3a	24.2424	22.3806	-0.115288	0.44145	0.599327	no
Stxbp5	8.79386	8.92481	0.0213239	0.88485	0.935578	no
Styx	13.5987	12.2044	-0.156069	0.3734	0.532033	no
Sub1	119.422	102.257	-0.223874	0.1121	0.212113	no
Sucla2	35.9101	33.4252	-0.10345	0.48685	0.642032	no
Suclg1	62.5314	68.7392	0.136552	0.3481	0.505298	no
Suclg2	13.1907	12.1893	-0.113903	0.4587	0.616145	no
Suco	17.8308	18.2508	0.0335822	0.8136	0.892116	no
Suds3	50.0159	47.615	-0.0709704	0.6201	0.755957	no
Sufu	11.9713	12.1894	0.0260496	0.8561	0.918456	no
Sugp1	32.0457	31.3785	-0.0303524	0.8374	0.906858	no
Sugp2	13.9113	13.6225	-0.0302625	0.8529	0.9165	no
Sugt1	74.7081	73.8597	-0.016477	0.90435	0.947228	no
Sult2b1	0.84468	1.24547	0.560212	0.21245	0.352156	no
Sumf1	10.8763	10.6298	-0.0330735	0.843	0.910324	no
Sumf2	8.79059	9.75044	0.149507	0.4002	0.55919	no
Sumo1	121.512	115.972	-0.0673218	0.6335	0.766551	no
Sumo2	390.373	344.367	-0.180907	0.1949	0.329424	no
Sumo3	37.7295	31.2566	-0.271533	0.0585	0.124212	no
Sun1	33.7878	30.8252	-0.132391	0.3543	0.511887	no
Sun2	140.899	153.97	0.127985	0.3829	0.54198	no
Suox	14.4063	12.0425	-0.258565	0.10935	0.208025	no
Supt16	32.3337	31.4607	-0.039486	0.7889	0.876856	no
Supt20	60.7466	64.4071	0.0844156	0.54645	0.694959	no
Supt3	5.02542	5.41446	0.107574	0.8888	0.937703	no
Supt4a	304.402	286.187	-0.0890212	0.53235	0.682884	no
Supt5	94.7481	94.2461	-0.00766297	0.95495	0.975954	no
Supt6	31.6472	33.2564	0.0715547	0.609	0.746776	no
Supt7l	7.89628	6.98433	-0.177051	0.30755	0.460764	no
Supv3l1	9.52808	9.26468	-0.040444	0.821	0.896872	no
Surf1	61.0417	55.6594	-0.133171	0.72355	0.832241	no
Surf2	31.2465	34.0193	0.122659	0.8009	0.884262	no
Surf4	108.077	106.206	-0.0252019	0.8737	0.929049	no
Surf6	18.5853	21.192	0.189358	0.2087	0.347318	no
Susd1	1.22681	0.735196	-0.738708	0.01275	0.0337909	yes
Susd2	1.12737	0.905522	-0.31614	0.2637	0.410265	no
Susd3	12.8013	11.7314	-0.125919	0.5097	0.66306	no
Suv39h1	12.8785	10.6191	-0.278304	0.0785	0.158043	no
Suv420h1	28.2259	30.0264	0.0892132	0.5382	0.688037	no
Suv420h2	17.2102	17.8778	0.0549012	0.72665	0.834446	no
Suz12	44.8884	39.5231	-0.183647	0.1949	0.329424	no
Svil	12.4338	12.457	0.00268744	0.9861	0.99189	no
Swap70	10.4111	19.7076	0.920632	5.00E-05	0.000236281	yes
Swi5	128.895	132.868	0.0437976	0.7642	0.860034	no
Swsap1	6.09021	6.66727	0.130603	0.507	0.660729	no
Swt1	9.23489	9.09811	-0.0215279	0.89175	0.939411	no
Syap1	37.1227	32.8497	-0.176421	0.21835	0.359311	no
Syce2	5.59968	4.73436	-0.242177	0.7132	0.82519	no
Syf2	141.461	136.326	-0.0533513	0.70455	0.819184	no
Sympk	44.7418	44.3365	-0.0131287	0.92335	0.958227	no

Syncrip	53.0696	56.683	0.0950327	0.53985	0.689474	no
Syne2	1.82036	1.81245	-0.00628259	0.96625	0.981604	no
Syne3	7.74736	8.38674	0.114405	0.45515	0.612858	no
Syngr2	45.1573	45.6641	0.0160984	0.9157	0.953738	no
Synj1	12.2271	12.5292	0.0352129	0.86585	0.92435	no
Synj2	1.75794	2.61542	0.573162	0.0184	0.0463692	yes
Synj2bp	7.05566	7.12493	0.0140946	0.92255	0.957904	no
Synrg	19.5195	18.9344	-0.043906	0.75515	0.853866	no
Sypl	6.93693	7.14147	0.0419226	0.79055	0.877833	no
Sys1	57.3355	49.4145	-0.214494	0.1401	0.253176	no
Syt11	11.6934	9.63462	-0.279389	0.06965	0.143229	no
Sytl1	23.2205	30.6823	0.402009	0.0097	0.0267231	yes
Sytl2	44.1607	37.2254	-0.246476	0.08215	0.164212	no
Sytl3	58.4235	57.3709	-0.0262276	0.8525	0.916196	no
Syvn1	18.2898	17.4212	-0.0701931	0.7152	0.826529	no
Szrd1	31.8781	31.9224	0.00200296	0.98885	0.993467	no
Szt2	14.3675	14.8727	0.0498656	0.73125	0.837589	no
Tab1	10.8774	10.9105	0.00437949	0.9775	0.987475	no
Tab2	58.0699	58.6687	0.0148005	0.91515	0.953502	no
Tab3	5.32337	5.09876	-0.0621939	0.69315	0.811261	no
Tacc1	38.8865	42.2832	0.120816	0.39555	0.554647	no
Tacc2	2.36796	4.61317	0.962112	5.00E-05	0.000236281	yes
Tacc3	26.3671	17.4434	-0.59606	5.00E-05	0.000236281	yes
Taco1	10.7927	12.7742	0.243177	0.1905	0.323599	no
Tada1	26.46	27.4529	0.0531481	0.85145	0.91543	no
Tada2a	4.02541	3.8039	-0.0816549	0.705	0.819531	no
Tada2b	8.32938	9.29113	0.157645	0.5118	0.664946	no
Tada3	21.4637	20.8455	-0.042161	0.789	0.876912	no
Taf1	14.0244	12.8775	-0.123085	0.39035	0.54944	no
Taf10	200.112	189.038	-0.0821306	0.74825	0.849397	no
Taf11	27.2799	27.4587	0.00942046	0.9545	0.975691	no
Taf12	49.2916	45.6003	-0.112297	0.452	0.609477	no
Taf13	30.9089	28.2406	-0.130253	0.41205	0.570944	no
Taf15	116.072	119.82	0.0458503	0.7383	0.842656	no
Taf1b	30.8667	28.4903	-0.115583	0.4324	0.590585	no
Taf1c	13.7353	15.2063	0.146782	0.33075	0.486395	no
Taf1d	50.2772	48.0107	-0.0665477	0.76385	0.859819	no
Taf2	15.2935	15.0667	-0.0215478	0.87985	0.932638	no
Taf3	5.84466	5.90936	0.0158846	0.9212	0.957136	no
Taf4a	20.5573	20.8167	0.0180886	0.89735	0.943087	no
Taf4b	2.6035	3.67839	0.498621	0.0064	0.018565	yes
Taf5	4.75157	4.70064	-0.0155478	0.96025	0.978593	no
Taf5l	29.1594	31.9528	0.131978	0.36055	0.518602	no
Taf6	26.2331	24.4152	-0.103608	0.5389	0.688571	no
Taf6l	2.39281	2.75254	0.202055	0.67195	0.795765	no
Taf7	12.1613	12.6773	0.0599503	0.771	0.864626	no
Taf8	25.4853	24.43	-0.061015	0.6826	0.803591	no
Taf9	54.3245	53.5386	-0.0210236	0.8951	0.941465	no
Taf9b	21.1676	18.8798	-0.165017	0.27975	0.429358	no
Tagap	38.4946	48.5342	0.334347	0.0196	0.0489684	yes
Tagap1	6.49418	7.818	0.267653	0.14045	0.253698	no
Tagln	4.14678	3.7808	-0.1333	0.8458	0.911855	no
Tagln2	442.711	558.58	0.335399	0.01955	0.0488608	yes
Taldo1	186.88	185.543	-0.0103595	0.9462	0.970877	no
Tamm41	11.3272	11.905	0.0717694	0.71065	0.823509	no
Tanc1	5.72904	7.58436	0.404733	0.00745	0.0212022	yes
Tango2	20.4894	18.7886	-0.125016	0.44165	0.599518	no

Tango6	1.88076	1.77924	-0.0800578	0.70905	0.822384	no
Tank	25.1264	23.6217	-0.0890924	0.5597	0.706199	no
Taok1	12.3453	12.0699	-0.0325489	0.81455	0.892672	no
Taok2	34.6868	33.7711	-0.0385971	0.7826	0.872423	no
Taok3	41.13	39.412	-0.0615537	0.6587	0.786034	no
Tap1	133.641	122.388	-0.126894	0.3709	0.529486	no
Tap2	132.883	145.596	0.131818	0.3525	0.510038	no
Tapbp	107.875	119.251	0.144641	0.3049	0.457687	no
Tapbpl	66.693	72.1363	0.113189	0.64235	0.773441	no
Tapt1	26.6694	25.822	-0.0465816	0.7464	0.848198	no
Tarbp2	19.0261	19.3931	0.0275629	0.86155	0.921781	no
Tardbp	33.2674	31.9272	-0.0593231	0.67265	0.796233	no
Tars	32.3916	29.8986	-0.115542	0.4211	0.579547	no
Tars2	19.884	23.4474	0.237816	0.1146	0.216021	no
Tarsl2	2.18889	2.43802	0.155512	0.4788	0.634689	no
Tasp1	6.41466	8.89372	0.471416	0.0111	0.0300337	yes
Tatdn1	6.39393	5.46703	-0.225946	0.62225	0.757575	no
Tatdn2	39.5116	43.9774	0.154488	0.2795	0.429058	no
Tatdn3	4.37265	4.058	-0.107737	0.6737	0.797035	no
Tax1bp1	93.1821	86.3925	-0.109146	0.43815	0.596154	no
Tax1bp3	24.4162	15.5574	-0.650236	5.00E-05	0.000236281	yes
Taz	27.1782	27.152	-0.00139377	0.9934	0.995883	no
Tbc1d1	19.7453	20.8372	0.0776559	0.5862	0.728359	no
Tbc1d10a	29.4283	32.6653	0.150558	0.3188	0.473228	no
Tbc1d10b	39.3276	33.6604	-0.224488	0.11425	0.215482	no
Tbc1d10c	321.238	317.885	-0.0151379	0.9198	0.9563	no
Tbc1d13	23.3168	24.0309	0.0435223	0.76065	0.857653	no
Tbc1d14	27.6645	24.902	-0.151774	0.2945	0.446003	no
Tbc1d15	17.3816	16.1496	-0.10607	0.4702	0.626797	no
Tbc1d17	19.3338	19.5934	0.0192448	0.9351	0.964747	no
Tbc1d2	4.3871	3.96334	-0.146548	0.4005	0.559498	no
Tbc1d20	37.793	37.4701	-0.0123778	0.9318	0.96275	no
Tbc1d22a	20.8444	19.2795	-0.112585	0.4482	0.605626	no
Tbc1d22b	42.2605	41.7997	-0.0158189	0.9134	0.952565	no
Tbc1d23	7.1159	6.84929	-0.0550914	0.7376	0.842198	no
Tbc1d25	7.65359	6.84427	-0.16124	0.3565	0.514265	no
Tbc1d2b	20.783	20.5997	-0.0127807	0.92675	0.96009	no
Tbc1d31	4.2801	3.28528	-0.381631	0.04555	0.100535	no
Tbc1d4	0.945001	2.4281	1.36144	5.00E-05	0.000236281	yes
Tbc1d5	5.4306	5.96842	0.136237	0.39475	0.553954	no
Tbc1d7	7.14676	5.4865	-0.381403	0.08865	0.175058	no
Tbc1d8b	6.08574	6.16762	0.019281	0.9008	0.945066	no
Tbc1d9b	21.6462	23.759	0.13436	0.33955	0.495967	no
Tbca	239.661	223.076	-0.103465	0.47725	0.633313	no
Tbcb	52.1587	49.9718	-0.0617934	0.6903	0.809267	no
Tbcc	12.2807	10.7076	-0.197762	0.25745	0.402618	no
Tbccd1	6.83168	7.69181	0.171083	0.32295	0.477961	no
Tbcd	21.8958	19.5707	-0.161964	0.33195	0.487621	no
Tbce	23.8822	23.023	-0.0528578	0.80935	0.889695	no
Tbcel	14.0231	19.9163	0.506151	0.00065	0.00244919	yes
Tbck	4.27602	4.5635	0.0938723	0.6069	0.74525	no
Tbk1	33.435	32.0875	-0.0593464	0.67865	0.800728	no
Tbkbp1	22.4375	11.7648	-0.931432	5.00E-05	0.000236281	yes
Tbl1x	14.1965	15.0112	0.0804994	0.613	0.750208	no
Tbl1xr1	19.1302	20.3681	0.090463	0.5248	0.676408	no
Tbl2	14.8686	16.0032	0.106094	0.47165	0.628137	no
Tbl3	13.7374	13.9692	0.0241401	0.88075	0.933144	no

Tbp	21.9171	20.5193	-0.0950741	0.54045	0.689927	no
Tbpl1	19.6791	16.7651	-0.231209	0.12295	0.228556	no
Tbrg1	79.2503	78.0495	-0.0220256	0.8772	0.931183	no
Tbrg4	22.5448	23.7016	0.0721863	0.6316	0.765078	no
Tbx21	157.406	113.878	-0.467003	7.00E-04	0.00261609	yes
Tbx6	4.03786	4.4036	0.125091	0.5833	0.72606	no
Tbxa2r	13.0248	13.2391	0.0235424	0.8883	0.937344	no
Tcaim	3.28442	4.20635	0.35693	0.0646	0.134613	no
Tcea1	65.8667	62.7829	-0.0691758	0.62	0.755905	no
Tcea2	6.46421	7.80732	0.272355	0.2271	0.370052	no
Tceanc	2.82312	2.53723	-0.154033	0.4284	0.586609	no
Tceanc2	7.65209	4.11707	-0.894235	5.00E-05	0.000236281	yes
Tceb1	65.9995	55.2918	-0.255389	0.09395	0.183582	no
Tceb2	329.895	306.806	-0.104681	0.83615	0.906116	no
Tceb3	32.2365	34.7979	0.110307	0.4406	0.598519	no
Tcerg1	21.8131	22.3432	0.0346458	0.8122	0.891426	no
Tcf12	23.5738	27.8136	0.238607	0.09955	0.192584	no
Tcf19	35.9297	20.3502	-0.820136	5.00E-05	0.000236281	yes
Tcf20	16.1389	16.7752	0.0557853	0.7034	0.818264	no
Tcf25	104.832	103.793	-0.0143691	0.92045	0.956694	no
Tcf3	70.3589	71.8274	0.0298019	0.83445	0.905176	no
Tcf7	185.949	302.995	0.704387	5.00E-05	0.000236281	yes
Tchp	8.63898	7.49741	-0.204468	0.2185	0.359525	no
Tcirg1	51.6811	50.823	-0.0241562	0.86425	0.92341	no
Tcn2	11.371	6.65757	-0.772287	5.00E-05	0.000236281	yes
Tcof1	36.4485	38.9764	0.0967408	0.49515	0.649833	no
Tcp1	92.4745	96.483	0.0612191	0.68675	0.80662	no
Tcp11	2.57916	2.7322	0.0831618	0.7361	0.841138	no
Tcp11l2	60.3629	72.7674	0.269631	0.057	0.121535	no
Tcta	8.50871	8.5891	0.0135661	0.93915	0.967073	no
Tctn1	15.3077	18.0652	0.238949	0.2257	0.368366	no
Tctn3	3.73789	3.75593	0.00694594	0.9756	0.986629	no
Tdg	6.726	6.53408	-0.0417657	0.89415	0.940886	no
Tdp1	16.3063	14.0081	-0.219166	0.1791	0.30803	no
Tdp2	15.551	13.8536	-0.166749	0.30965	0.463104	no
Tdrd3	5.17509	5.99956	0.213273	0.24905	0.394445	no
Tdrp	1.45907	3.25859	1.1592	0.00015	0.000653255	yes
Tec	5.75923	7.38943	0.359586	0.04445	0.0984705	no
Tecpr1	70.0596	87.4512	0.319895	0.024	0.0584268	no
Tecpr2	1.96239	2.29074	0.223202	0.20485	0.342291	no
Tecr	124.026	122.218	-0.0211962	0.8828	0.934446	no
Tef	8.88946	7.50309	-0.244611	0.1207	0.225264	no
Tefm	3.76666	4.463	0.244725	0.3324	0.488099	no
Telo2	4.52377	5.27971	0.222934	0.2091	0.34782	no
Ten1	50.9797	48.1025	-0.0838129	0.70655	0.820617	no
Tenc1	0.906188	1.05274	0.216271	0.40285	0.561836	no
Tep1	8.16823	9.42621	0.206654	0.15565	0.275354	no
Terf1	13.7718	13.0545	-0.0771713	0.63135	0.764867	no
Terf2	23.3628	25.1246	0.104886	0.482	0.637578	no
Terf2ip	13.7462	12.8246	-0.10012	0.51095	0.664184	no
Tes	104.978	97.3555	-0.10875	0.43945	0.597462	no
Tesc	28.8231	27.0574	-0.0912011	0.60185	0.741357	no
Tesk1	9.2703	9.80017	0.0801909	0.699	0.815377	no
Tesk2	1.74082	1.69905	-0.0350391	0.9442	0.969778	no
Tespa1	33.7021	43.98	0.38401	0.0103	0.0281508	yes
Tet1	1.03633	1.27336	0.297161	0.0992	0.192049	no
Tet2	6.86231	7.51848	0.131748	0.3749	0.53361	no

Tet3	8.73497	10.5062	0.26637	0.0629	0.131837	no
Tex10	23.2213	26.5999	0.19597	0.2381	0.382885	no
Tex2	2.97489	2.8302	-0.0719314	0.69865	0.815076	no
Tex261	46.5909	44.9528	-0.051635	0.72925	0.836058	no
Tex264	40.235	44.712	0.152213	0.3099	0.463389	no
Tex30	16.6337	16.1132	-0.0458659	0.7903	0.877708	no
Tex9	5.3962	4.90713	-0.137064	0.54975	0.697811	no
Tfam	16.1899	14.1069	-0.198694	0.1823	0.312483	no
Tfap4	2.47288	2.93879	0.249033	0.3112	0.464801	no
Tfb1m	8.03318	8.33169	0.0526375	0.80515	0.887012	no
Tfb2m	13.2569	14.705	0.149561	0.349	0.506302	no
Tfcp2	1.76793	2.29769	0.378124	0.097	0.188592	no
Tfdp1	40.4585	34.8729	-0.214335	0.1425	0.256492	no
Tfdp2	1.12849	1.0744	-0.0708674	0.73955	0.843401	no
Tfe3	10.488	10.2131	-0.0383245	0.81455	0.892672	no
Tfeb	12.0735	9.87072	-0.290614	0.07755	0.156542	no
Tfg	28.2531	26.1699	-0.110499	0.4668	0.623667	no
Tfip11	22.5329	21.3844	-0.0754733	0.636	0.768391	no
Tfpi	1.50094	1.26009	-0.252339	0.4492	0.606582	no
Tfpt	17.2702	18.0872	0.0666865	0.70455	0.819184	no
Tfrc	7.8715	8.30786	0.0778381	0.61345	0.750603	no
Tgds	16.9278	17.5369	0.0510003	0.7498	0.850432	no
Tgfb1	56.1165	56.555	0.0112286	0.9386	0.966747	no
Tgfbr1	5.15041	5.73811	0.155889	0.3369	0.493107	no
Tgfbr2	63.1354	66.8074	0.0815599	0.5685	0.713387	no
Tgfbrap1	15.035	14.7406	-0.0285248	0.8443	0.91124	no
Tgif1	12.12	14.8586	0.293902	0.0992	0.192049	no
Tgif2	1.11663	1.21531	0.122171	0.72505	0.83326	no
Tgm4	1.41121	1.64907	0.224711	0.3978	0.556884	no
Tgoln1	50.4535	46.4605	-0.118948	0.4435	0.601243	no
Tgoln2	19.7577	23.4639	0.248029	0.50705	0.660782	no
Tgs1	10.0343	9.58623	-0.0659033	0.6698	0.794258	no
Tgtp1	0.729657	1.04409	0.516951	0.2916	0.442608	no
Tha1	3.96569	7.13997	0.848348	0.00015	0.000653255	yes
Thada	8.35252	10.1156	0.276297	0.0585	0.124212	no
Thap1	7.01642	7.2285	0.0429612	0.8181	0.895034	no
Thap11	23.4094	23.2955	-0.00703986	0.9617	0.979491	no
Thap2	4.64847	4.72868	0.0246829	0.88845	0.93746	no
Thap3	102.454	100.837	-0.0229515	0.87715	0.931161	no
Thap4	13.3699	11.3557	-0.235567	0.1577	0.278332	no
Thap7	18.5905	18.7108	0.00930832	0.95585	0.976393	no
Them4	1.71172	2.58474	0.594568	0.0375	0.0852193	no
Them6	19.1689	19.8215	0.0482954	0.7719	0.865251	no
Themis	58.5744	67.1367	0.19683	0.16475	0.288367	no
Themis2	15.2192	14.4749	-0.0723435	0.64085	0.772175	no
Thg1l	2.67107	3.27017	0.291948	0.13775	0.250056	no
Thnsl1	1.13276	1.29415	0.192168	0.4388	0.596855	no
Thoc1	14.2257	15.0632	0.0825286	0.68865	0.808044	no
Thoc2	20.5482	20.3307	-0.015351	0.91345	0.952575	no
Thoc3	16.0243	16.3671	0.0305404	0.84345	0.910643	no
Thoc5	26.8975	27.9305	0.0543701	0.70725	0.821092	no
Thoc6	16.7959	14.2555	-0.236587	0.34515	0.502038	no
Thoc7	68.2182	65.6159	-0.0561103	0.69965	0.815717	no
Thop1	3.41921	3.82765	0.162796	0.42955	0.587841	no
Thra	1.73658	2.68425	0.628264	0.01535	0.0396209	yes
Thrap3	76.8175	82.4874	0.102738	0.46715	0.623957	no
Thtpa	15.1953	16.0726	0.0809785	0.78095	0.871189	no

Thumpd1	25.0105	28.0456	0.165243	0.2559	0.400833	no
Thumpd2	5.71076	6.59642	0.208002	0.29545	0.447	no
Thumpd3	27.1319	27.9897	0.0449054	0.78145	0.871582	no
Thy1	1053.21	1018.03	-0.0490118	0.74525	0.847396	no
Thyn1	22.3185	23.3695	0.066383	0.88365	0.934939	no
Tia1	18.248	17.9503	-0.0237354	0.86955	0.926633	no
Tial1	21.3328	21.0768	-0.0174193	0.90595	0.948214	no
Ticam1	9.88211	8.96118	-0.141129	0.3907	0.549714	no
Ticrr	3.688	2.50211	-0.559692	0.0047	0.0141816	yes
Tigd2	12.2387	13.07	0.0948072	0.5458	0.694295	no
Tigd3	1.254	0.794359	-0.658675	0.05735	0.122174	no
Tigit	65.2647	90.1782	0.466476	0.0021	0.00696828	yes
Timeless	8.14026	5.47236	-0.572912	0.00065	0.00244919	yes
Timm10	11.2709	11.0735	-0.0254917	0.9122	0.95189	no
Timm10b	28.0184	25.362	-0.143707	0.3924	0.551512	no
Timm13	44.6018	45.5638	0.0307861	0.84115	0.909133	no
Timm17a	41.7954	46.6386	0.158181	0.3213	0.476208	no
Timm17b	18.0987	16.5584	-0.12832	0.4365	0.594517	no
Timm21	15.0966	15.3181	0.021013	0.90735	0.94899	no
Timm22	8.35508	8.67654	0.0544657	0.7389	0.843136	no
Timm23	81.2757	84.4688	0.0555943	0.7002	0.816048	no
Timm44	32.7647	32.2566	-0.0225475	0.88015	0.932844	no
Timm50	24.3663	24.7462	0.0223171	0.8973	0.943062	no
Timm8a1	13.3978	15.9574	0.252237	0.16645	0.290787	no
Timm8b	46.3487	52.6084	0.182764	0.55295	0.700512	no
Timm9	12.0804	12.8006	0.083545	0.6924	0.810692	no
Timmdc1	11.4143	11.9484	0.0659704	0.69865	0.815076	no
Timp2	4.04387	2.85965	-0.499895	0.0089	0.0247598	yes
Tinf2	20.3877	18.7638	-0.119746	0.4487	0.606092	no
Tiparp	8.04428	8.53712	0.0857858	0.59385	0.734799	no
Tipin	34.8477	22.2814	-0.645222	5.00E-05	0.000236281	yes
Tiprl	16.0783	13.966	-0.203193	0.16965	0.294976	no
Tjap1	16.3924	16.1348	-0.0228568	0.88445	0.935379	no
Tjp2	2.45576	3.0458	0.310651	0.1024	0.197061	no
Tjp3	5.64024	5.51607	-0.0321141	0.8952	0.941528	no
Tk1	25.5261	13.2071	-0.950662	0.0011	0.00391273	yes
Tk2	25.3842	26.3871	0.0559017	0.70925	0.822508	no
Tkt	74.6047	73.5511	-0.0205187	0.88525	0.93586	no
Tktl1	0.70278	1.30376	0.891533	0.0093	0.0257386	yes
Tldc1	3.46082	3.99235	0.206123	0.31135	0.464976	no
Tle1	4.35759	5.42926	0.317227	0.16875	0.293707	no
Tle3	66.5288	81.5572	0.293833	0.038	0.0861807	no
Tle4	23.5742	23.9459	0.0225682	0.8748	0.929645	no
Tlk1	46.8838	45.7644	-0.0348648	0.79615	0.881293	no
Tlk2	12.5314	13.0315	0.0564543	0.691	0.809711	no
Tln1	188.185	185.016	-0.0245001	0.87825	0.931866	no
Tlr1	10.3999	15.069	0.535007	6.00E-04	0.00228064	yes
Tlr12	2.03787	1.58299	-0.364405	0.13135	0.241079	no
Tlr2	0.850795	1.05495	0.310292	0.3144	0.468243	no
Tlr6	1.4972	1.51143	0.0136441	0.94945	0.972783	no
Tm2d1	21.1731	21.8984	0.0485927	0.78475	0.874033	no
Tm2d2	16.1981	16.6453	0.0392879	0.826	0.900147	no
Tm2d3	18.5195	19.0434	0.0402418	0.8272	0.900787	no
Tm6sf1	52.2456	33.6322	-0.635466	0.003	0.00955705	yes
Tm7sf2	2.13608	2.64983	0.310937	0.62785	0.761896	no
Tm7sf3	13.401	13.4593	0.0062596	0.97155	0.984324	no
Tm9sf1	31.2675	30.3555	-0.0427055	0.7659	0.861124	no

Tm9sf2	43.1737	42.8252	-0.0116928	0.93695	0.96578	no
Tm9sf3	48.3651	49.5923	0.0361518	0.79675	0.88168	no
Tm9sf4	16.698	16.8272	0.0111166	0.93895	0.966952	no
Tma16	5.97368	6.68226	0.161716	0.4972	0.651651	no
Tma7	350.876	331.292	-0.0828545	0.5536	0.701084	no
Tmbim1	3.68966	2.91635	-0.339323	0.2696	0.417365	no
Tmbim4	30.4314	33.949	0.157808	0.3603	0.518359	no
Tmbim6	252.982	232.201	-0.123656	0.3894	0.548541	no
Tmc6	89.3639	90.984	0.0259205	0.8623	0.922218	no
Tmc8	63.4692	58.5328	-0.116812	0.4122	0.571062	no
Tmcc1	2.78667	3.08155	0.145115	0.41755	0.576156	no
Tmco1	13.3381	13.3837	0.00492592	0.975	0.986277	no
Tmco3	3.6504	3.24261	-0.170899	0.5174	0.669941	no
Tmco4	15.8733	13.1273	-0.274032	0.07275	0.148354	no
Tmco6	19.0166	18.5249	-0.0377881	0.88395	0.935074	no
Tmed1	7.55985	8.60578	0.186948	0.8431	0.910391	no
Tmed10	43.5565	43.6803	0.00409426	0.97795	0.987661	no
Tmed2	168.579	164.254	-0.0375014	0.79015	0.87761	no
Tmed3	33.0176	31.3387	-0.0752888	0.6308	0.764385	no
Tmed4	19.9928	21.3563	0.095183	0.55415	0.701554	no
Tmed5	32.4878	33.192	0.0309397	0.82635	0.90032	no
Tmed7	34.9001	32.476	-0.103855	0.4681	0.624777	no
Tmed8	7.58156	7.40599	-0.0338012	0.82425	0.898947	no
Tmed9	170.09	174.952	0.0406615	0.7705	0.864285	no
Tmem101	8.38572	8.4306	0.00770055	0.96525	0.98104	no
Tmem104	8.25685	7.53938	-0.131146	0.40195	0.560947	no
Tmem106a	1.34302	1.00225	-0.422239	0.1714	0.297491	no
Tmem106b	17.4261	18.2396	0.0658252	0.6491	0.778607	no
Tmem106c	3.9242	3.55838	-0.141181	0.55905	0.705744	no
Tmem107	3.11065	2.38501	-0.383219	0.2275	0.370464	no
Tmem109	36.1362	36.24	0.00414025	0.977	0.987224	no
Tmem11	15.9613	15.0829	-0.0816644	0.64565	0.775932	no
Tmem110	6.16296	6.2098	0.0109229	0.9473	0.971568	no
Tmem115	15.644	14.9177	-0.0685905	0.6713	0.795315	no
Tmem120a	30.0342	25.9449	-0.211155	0.1836	0.314299	no
Tmem120b	2.35564	2.45996	0.0625197	0.8113	0.890853	no
Tmem123	88.6801	95.2717	0.103438	0.4681	0.624777	no
Tmem126a	57.5313	58.4266	0.0222779	0.8858	0.936091	no
Tmem126b	17.9835	18.9479	0.0753623	0.69275	0.810995	no
Tmem127	36.6786	37.0487	0.0144832	0.91825	0.955266	no
Tmem128	52.1505	50.3134	-0.0517383	0.7336	0.839178	no
Tmem129	10.913	10.4892	-0.0571478	0.7352	0.840477	no
Tmem131	15.5272	16.2253	0.0634522	0.65425	0.782736	no
Tmem134	61.7596	62.7967	0.0240254	0.8717	0.927887	no
Tmem135	9.02386	7.9727	-0.178676	0.264	0.41065	no
Tmem138	10.43	9.25684	-0.172142	0.43395	0.592232	no
Tmem141	0.678149	2.8028	2.04719	0.0016	0.00547227	yes
Tmem143	4.81064	4.74728	-0.0191276	0.92475	0.958862	no
Tmem147	40.0965	41.4525	0.0479844	0.7683	0.862723	no
Tmem14a	2.19394	1.94298	-0.175253	0.59165	0.732925	no
Tmem14c	133.493	125.094	-0.0937599	0.50665	0.660468	no
Tmem151a	2.01029	1.00494	-1.00029	0.00015	0.000653255	yes
Tmem154	20.0615	23.8579	0.250034	0.0872	0.172624	no
Tmem159	2.22171	3.03792	0.45141	0.11195	0.211932	no
Tmem160	65.579	60.467	-0.117085	0.4487	0.606092	no
Tmem161a	15.2848	15.6683	0.0357508	0.81685	0.894263	no
Tmem161b	12.1289	12.0338	-0.0113613	0.944	0.969705	no

Tmem163	15.0267	9.69562	-0.632125	2.00E-04	0.0008488	yes
Tmem164	12.7434	11.9566	-0.0919329	0.5604	0.706741	no
Tmem165	40.0087	34.5701	-0.210789	0.15705	0.277351	no
Tmem167	9.57143	9.0775	-0.0764389	0.59265	0.733777	no
Tmem167b	23.887	22.6873	-0.0743384	0.6107	0.748259	no
Tmem168	7.22172	7.12173	-0.0201133	0.8967	0.942642	no
Tmem170	1.68668	1.7151	0.0241056	0.9401	0.967614	no
Tmem170b	1.63916	1.68667	0.0412238	0.82795	0.901131	no
Tmem171	1.63625	1.46621	-0.158301	0.68035	0.802039	no
Tmem173	47.795	52.0021	0.121711	0.39925	0.55826	no
Tmem175	10.9328	9.74211	-0.166362	0.53745	0.68749	no
Tmem176a	2.88951	4.368	0.596145	0.03995	0.0899413	no
Tmem176b	2.98812	4.55182	0.607208	0.02775	0.0660836	no
Tmem177	1.66601	1.7315	0.055619	0.8201	0.896347	no
Tmem179b	82.7325	82.503	-0.00400779	0.98305	0.990219	no
Tmem18	11.3796	10.5213	-0.113141	0.478	0.634057	no
Tmem180	1.99518	2.86817	0.523608	0.0338	0.0780192	no
Tmem181a	5.78357	5.79699	0.00334362	0.9861	0.99189	no
Tmem183a	21.2991	20.5906	-0.0488119	0.741	0.844386	no
Tmem184b	13.9828	14.424	0.0448162	0.7703	0.86413	no
Tmem184c	21.7477	21.5842	-0.0108815	0.94225	0.968641	no
Tmem185b	9.2967	10.4704	0.171525	0.29085	0.44185	no
Tmem186	7.46739	8.94102	0.259835	0.1265	0.233887	no
Tmem189	50.6978	49.1586	-0.0444798	0.758	0.855758	no
Tmem19	14.3987	13.2539	-0.119524	0.43225	0.590414	no
Tmem191c	4.83922	8.18669	0.758505	0.00085	0.00311418	yes
Tmem192	38.5314	37.5897	-0.0356957	0.8183	0.895142	no
Tmem194	3.6819	3.16691	-0.217373	0.28465	0.43494	no
Tmem194b	18.529	16.2488	-0.189451	0.1997	0.335752	no
Tmem198	4.11777	4.9824	0.274978	0.18235	0.312546	no
Tmem198b	3.05663	3.14293	0.0401673	0.85225	0.915997	no
Tmem199	13.3639	12.9496	-0.0454323	0.80415	0.886506	no
Tmem2	2.30843	3.07696	0.414592	0.0182	0.0459177	yes
Tmem201	5.76474	6.00346	0.0585377	0.7266	0.834416	no
Tmem203	14.2002	12.3618	-0.200023	0.31785	0.472176	no
Tmem206	1.9634	2.04065	0.0556737	0.96825	0.982744	no
Tmem208	58.5145	55.5495	-0.0750196	0.6314	0.764915	no
Tmem209	19.0249	17.7376	-0.10108	0.4977	0.652089	no
Tmem214	19.2166	19.913	0.0513643	0.73805	0.842521	no
Tmem216	2.47935	3.25416	0.392323	0.2105	0.34972	no
Tmem218	1.84877	1.46687	-0.333827	0.39515	0.554317	no
Tmem219	30.5021	31.0401	0.025226	0.88635	0.936364	no
Tmem221	4.74554	6.09032	0.359944	0.10435	0.200115	no
Tmem222	35.1374	35.7606	0.025362	0.86385	0.923165	no
Tmem223	28.6066	34.575	0.27338	0.12315	0.228808	no
Tmem229b	21.9478	23.1116	0.074542	0.60065	0.740306	no
Tmem230	24.4722	23.1417	-0.0806505	0.60605	0.744556	no
Tmem231	1.41601	1.08994	-0.377588	0.17495	0.302233	no
Tmem234	141.312	129.459	-0.126391	0.38505	0.544074	no
Tmem237	4.78762	3.66037	-0.387321	0.07685	0.155337	no
Tmem238	6.19287	7.42043	0.260894	0.26015	0.405896	no
Tmem241	4.00405	3.98576	-0.00660516	0.9718	0.98445	no
Tmem242	34.8788	34.524	-0.0147508	0.9312	0.962482	no
Tmem243	15.8126	17.2336	0.124147	0.8012	0.884414	no
Tmem245	4.65467	5.22449	0.166613	0.2822	0.432085	no
Tmem248	19.5836	17.8921	-0.130324	0.3763	0.535096	no
Tmem251	26.1859	27.5199	0.0716835	0.7367	0.841578	no

Tmem255a	1.75576	1.75273	-0.00249501	0.9965	0.997475	no
Tmem256	60.7772	56.1272	-0.11483	0.53555	0.685904	no
Tmem258	242.63	218.329	-0.152254	0.3146	0.468422	no
Tmem259	61.3195	64.2488	0.0673238	0.6385	0.770501	no
Tmem260	7.89671	8.07768	0.0326895	0.8297	0.902201	no
Tmem261	69.4218	75.7884	0.126589	0.42135	0.579744	no
Tmem29	5.40065	3.79769	-0.508013	0.0367	0.0836436	no
Tmem30a	61.6794	55.9789	-0.139905	0.32295	0.477961	no
Tmem33	9.73507	8.74894	-0.154084	0.29945	0.451629	no
Tmem37	9.37647	6.7829	-0.467142	0.0393	0.0886901	no
Tmem38b	2.55317	2.22457	-0.198767	0.3884	0.54758	no
Tmem39a	9.42626	9.52099	0.014426	0.93085	0.962205	no
Tmem39b	7.11387	7.19314	0.0159876	0.93375	0.96396	no
Tmem41a	4.37714	4.27294	-0.0347603	0.88715	0.936789	no
Tmem41b	6.13437	5.27055	-0.218962	0.2063	0.344193	no
Tmem42	9.42536	12.9898	0.462758	0.0247	0.0599119	no
Tmem43	28.6388	26.9772	-0.086231	0.54835	0.696647	no
Tmem5	6.74937	6.92823	0.0377356	0.86205	0.922037	no
Tmem50a	291.241	288.017	-0.0160576	0.9093	0.949974	no
Tmem50b	15.4724	15.4318	-0.00378775	0.98135	0.989348	no
Tmem51	1.45771	1.23604	-0.237969	0.46515	0.622145	no
Tmem55a	9.17061	9.26817	0.0152675	0.9317	0.962703	no
Tmem55b	12.9166	13.3669	0.0494363	0.7637	0.859801	no
Tmem57	8.05595	7.15754	-0.17059	0.29045	0.441384	no
Tmem59	76.4118	74.1366	-0.0436105	0.768	0.862537	no
Tmem60	17.5232	16.799	-0.0608912	0.74785	0.849094	no
Tmem62	11.1095	12.2802	0.144534	0.36195	0.520139	no
Tmem63a	21.3647	25.8158	0.273022	0.0638	0.133252	no
Tmem63b	3.79081	3.62931	-0.0628097	0.74515	0.847323	no
Tmem64	3.1879	3.88111	0.283864	0.1121	0.212113	no
Tmem65	5.63249	5.83969	0.0521196	0.7582	0.855916	no
Tmem66	347.994	369.883	0.0880091	0.5383	0.688066	no
Tmem68	9.45321	9.0236	-0.0671025	0.6887	0.808063	no
Tmem69	7.52946	7.55563	0.00500581	0.97675	0.987057	no
Tmem70	19.2617	18.9654	-0.0223681	0.8866	0.936446	no
Tmem71	101.314	89.9414	-0.171779	0.2226	0.364393	no
Tmem79	1.09778	1.27635	0.217432	0.46915	0.625787	no
Tmem8	8.0341	9.32406	0.214822	0.1838	0.314527	no
Tmem80	12.3325	9.54431	-0.369748	0.35345	0.511056	no
Tmem81	7.42249	7.50448	0.0158478	0.93535	0.964864	no
Tmem86b	8.23283	8.78726	0.0940259	0.6535	0.782157	no
Tmem87a	28.6216	28.1932	-0.0217593	0.9043	0.947189	no
Tmem87b	8.22785	8.57698	0.0599532	0.70035	0.816102	no
Tmem88	1.99788	1.44017	-0.472232	0.38085	0.539826	no
Tmem9	4.72529	4.95008	0.0670513	0.7694	0.863463	no
Tmem97	11.3446	9.81164	-0.209435	0.2657	0.412658	no
Tmem9b	57.6299	55.5996	-0.0517433	0.71705	0.827677	no
Tmf1	14.5184	13.7888	-0.0743917	0.60515	0.743864	no
Tmod3	69.3581	69.1368	-0.00461062	0.9741	0.985861	no
Tmod4	1.18823	0.965815	-0.298995	0.4729	0.629422	no
Tmpo	93.3497	69.0747	-0.434488	0.00235	0.00770045	yes
Tmppe	3.83464	3.64921	-0.0715062	0.7644	0.860191	no
Tmprss13	3.08636	4.37175	0.502302	0.00975	0.0268451	yes
Tmsb10	2903.21	2706.51	-0.101216	0.50205	0.656192	no
Tmsb15b1	14.2851	16.4527	0.203807	0.5652	0.710775	no
Tmsb15l	11.0179	8.74932	-0.332604	0.20435	0.341658	no
Tmsb4x	13953.3	12015.5	-0.215705	0.4643	0.621361	no

Tmtc4	3.5497	2.95561	-0.264238	0.18365	0.314369	no
Tmub1	20.6383	20.8936	0.0177333	0.91515	0.953502	no
Tmub2	26.7339	25.0314	-0.0949338	0.74355	0.84624	no
Tmx1	37.2999	34.5906	-0.108792	0.4577	0.615153	no
Tmx2	19.5447	20.282	0.053419	0.8508	0.91494	no
Tmx3	29.3813	26.7965	-0.132853	0.34705	0.504147	no
Tmx4	6.49956	6.27992	-0.0495976	0.7562	0.854533	no
Tnf	13.7415	18.4671	0.426415	0.01115	0.0301552	yes
Tnfaip1	5.74486	5.57261	-0.0439185	0.79895	0.88309	no
Tnfaip3	231.525	254.774	0.13805	0.35905	0.516972	no
Tnfaip8	24.4584	24.7847	0.0191216	0.8939	0.940693	no
Tnfaip8l1	12.2754	10.8905	-0.172691	0.2911	0.442049	no
Tnfaip8l2	85.7773	81.6377	-0.0713616	0.65405	0.782602	no
Tnfrsf10b	1.07251	1.62197	0.596751	0.0267	0.0639998	no
Tnfrsf12a	1.91956	1.78573	-0.104262	0.9251	0.95907	no
Tnfrsf13b	7.58797	6.12929	-0.307994	0.2487	0.39405	no
Tnfrsf13c	9.07497	7.9024	-0.199603	0.27325	0.421665	no
Tnfrsf14	17.0866	17.7452	0.0545647	0.7728	0.865765	no
Tnfrsf18	35.6659	44.8211	0.329632	0.0383	0.0867579	no
Tnfrsf1a	21.0495	16.6368	-0.339406	0.03125	0.0730529	no
Tnfrsf1b	37.9322	40.8805	0.107989	0.4442	0.601972	no
Tnfrsf21	0.811365	1.18989	0.552406	0.05075	0.110149	no
Tnfrsf22	2.86061	4.61431	0.689792	0.0018	0.00607738	yes
Tnfrsf23	1.24533	1.71158	0.458805	0.0712	0.145842	no
Tnfrsf25	14.2111	18.5351	0.383241	0.02135	0.0528116	no
Tnfrsf26	12.2744	16.2133	0.401528	0.00905	0.0251275	yes
Tnfrsf4	2.8107	2.60499	-0.109652	0.71815	0.828527	no
Tnfrsf9	4.20617	3.21292	-0.388622	0.0743	0.150979	no
Tnfsf10	10.6214	11.9127	0.165531	0.27435	0.422992	no
Tnfsf14	22.3977	26.6016	0.248157	0.1077	0.205468	no
Tnfsf8	2.53946	5.47327	1.10788	5.00E-05	0.000236281	yes
Tnfsf9	3.42421	4.23855	0.307802	0.40145	0.560448	no
Tnik	12.376	13.1383	0.0862381	0.5637	0.709508	no
Tnip1	55.0159	54.679	-0.00886212	0.95155	0.974058	no
Tnip2	7.0457	5.9307	-0.248541	0.19095	0.32413	no
Tnk2	35.4061	32.8053	-0.110069	0.44495	0.602665	no
Tnks	7.39106	7.93553	0.102545	0.5048	0.658859	no
Tnks1bp1	5.63236	5.25622	-0.0997143	0.53015	0.681102	no
Tnks2	52.4656	54.5728	0.0568108	0.6953	0.812713	no
Tnnt1	1.84829	0.962253	-0.941702	0.03285	0.0761651	no
Tnnt3	1.57034	1.65975	0.0798924	0.9611	0.979063	no
Tnpo1	20.0435	20.5174	0.0337073	0.8144	0.892563	no
Tnpo2	20.4073	21.2501	0.0583846	0.6886	0.808013	no
Tnpo3	28.8735	29.7435	0.0428297	0.76985	0.863803	no
Tnrc18	19.4023	18.9596	-0.033299	0.81765	0.894764	no
Tnrc6a	28.5442	28.8737	0.016556	0.90785	0.949288	no
Tnrc6b	9.49117	9.623	0.0199002	0.892	0.939573	no
Tnrc6c	13.4354	14.2857	0.0885288	0.53175	0.682412	no
Tob1	47.2648	60.3339	0.352201	0.0146	0.0379571	yes
Tob2	29.6204	28.0191	-0.0801808	0.57555	0.719471	no
Toe1	23.148	23.8332	0.0420808	0.7921	0.878739	no
Tollip	28.2998	28.0386	-0.0133776	0.9241	0.958677	no
Tom1	11.0646	15.0441	0.443241	0.00635	0.0184367	yes
Tom1l2	5.46179	6.12693	0.165789	0.3464	0.503503	no
Tomm20	40.0143	37.9914	-0.0748427	0.5945	0.735319	no
Tomm22	161.925	148.422	-0.125628	0.378	0.536994	no
Tomm34	50.4225	49.8832	-0.0155111	0.91735	0.954667	no

Tomm40	23.8073	27.3355	0.199372	0.1993	0.33524	no
Tomm40l	12.4788	11.9047	-0.0679509	0.66655	0.791814	no
Tomm5	102.092	99.4718	-0.0375058	0.8111	0.890717	no
Tomm6	90.499	89.9728	-0.00841401	0.9574	0.977108	no
Tomm7	96.8566	87.2249	-0.151109	0.2969	0.448713	no
Tomm70a	30.5276	29.592	-0.0449084	0.75395	0.853001	no
Tomt	1.16582	1.0111	-0.205423	0.88655	0.936435	no
Tonsl	8.60383	7.89421	-0.124184	0.43295	0.591187	no
Top1	32.4029	33.8686	0.0638265	0.65645	0.784238	no
Top1mt	1.71306	1.87565	0.130809	0.6438	0.77454	no
Top2a	74.1419	44.0084	-0.75251	5.00E-05	0.000236281	yes
Top2b	63.4645	71.0558	0.163004	0.25095	0.396618	no
Top3a	6.16123	7.1097	0.206571	0.2154	0.355784	no
Top3b	20.5094	20.569	0.0041861	0.9787	0.988093	no
Topbp1	34.5071	31.7873	-0.118441	0.40125	0.560269	no
Topors	20.448	19.8952	-0.0395435	0.79435	0.880156	no
Tor1a	30.4166	32.0982	0.0776357	0.61195	0.749248	no
Tor1aip1	41.1652	39.8852	-0.0455712	0.80595	0.88745	no
Tor1aip2	25.9441	26.9607	0.0554465	0.81975	0.896145	no
Tor1b	28.7084	28.8043	0.00481113	0.9744	0.986023	no
Tor2a	39.7481	40.4218	0.024249	0.86915	0.92636	no
Tor3a	7.1438	8.02351	0.167541	0.3118	0.465451	no
Tor4a	35.3167	31.0693	-0.184861	0.19635	0.331359	no
Tox	3.46166	5.13501	0.568901	0.00315	0.00997458	yes
Tox4	26.6853	26.5869	-0.00533318	0.9767	0.987034	no
Tpcn1	12.5732	14.6254	0.218123	0.1411	0.254624	no
Tpcn2	6.99989	7.02696	0.00556825	0.97355	0.985502	no
Tpd52	1.81283	2.02187	0.157442	0.5417	0.690935	no
Tpd52l2	9.75807	8.90292	-0.132318	0.40985	0.568778	no
Tpgs1	29.2208	27.571	-0.0838478	0.6093	0.747079	no
Tpgs2	8.53549	8.30331	-0.0397875	0.8014	0.884538	no
Tpi1	31.3164	27.6387	-0.18023	0.268	0.415462	no
Tpk1	2.76181	3.02937	0.133405	0.54055	0.690017	no
Tpm1	30.1108	33.4818	0.153094	0.3086	0.461905	no
Tpm3	419.384	397.399	-0.0776834	0.5912	0.732483	no
Tpm4	203.65	170.894	-0.252995	0.07395	0.150371	no
Tpp1	21.2893	18.6994	-0.187137	0.2006	0.336848	no
Tpp2	43.3634	39.9874	-0.116934	0.4098	0.568764	no
Tppp3	11.6715	11.2717	-0.0502857	0.79925	0.88327	no
Tpr	60.5879	61.1233	0.0126934	0.92915	0.96125	no
Tpra1	20.741	20.8512	0.00765034	0.9642	0.98056	no
Tprgl	155.86	141.734	-0.137065	0.33235	0.488036	no
Tprkb	19.4154	17.3463	-0.162571	0.30015	0.452491	no
Tprn	6.52914	6.31283	-0.0486051	0.7865	0.875057	no
Tpst1	1.26675	1.27241	0.00642793	0.98195	0.989605	no
Tpst2	173.882	170.838	-0.025482	0.86215	0.92213	no
Tpt1	5741.05	5814.76	0.0184035	0.92725	0.960312	no
Tpx2	19.9928	11.1241	-0.84579	5.00E-05	0.000236281	yes
Tra2a	75.2661	75.2988	0.000628227	0.9974	0.998022	no
Tra2b	113.783	110.662	-0.040127	0.77465	0.867108	no
Trabd	45.6357	45.8192	0.00578737	0.96775	0.982463	no
Tradd	46.189	43.5213	-0.0858275	0.56415	0.709897	no
Traf1	67.597	71.8174	0.0873734	0.53125	0.682093	no
Traf2	21.1522	20.2809	-0.0606828	0.67965	0.801495	no
Traf3	12.8817	13.4064	0.057606	0.6855	0.805688	no
Traf3ip1	10.7904	6.43271	-0.746251	5.00E-05	0.000236281	yes
Traf3ip2	24.3789	27.9655	0.198015	0.1816	0.31157	no

Traf3ip3	112.708	103.621	-0.121265	0.4014	0.560412	no
Traf4	8.80989	9.84835	0.160758	0.3221	0.477003	no
Traf5	23.6361	23.5287	-0.00657335	0.966	0.981463	no
Traf6	16.591	14.9161	-0.153526	0.2932	0.444511	no
Traf7	57.0295	53.2706	-0.0983692	0.4971	0.651581	no
Trafd1	32.6197	33.7947	0.0510537	0.72455	0.832902	no
Traip	4.78417	2.40576	-0.991774	5.00E-05	0.000236281	yes
Trak1	19.5128	18.9599	-0.0414755	0.77305	0.865894	no
Trak2	23.3265	22.0733	-0.079666	0.5757	0.719594	no
Tram1	108.409	94.1368	-0.203651	0.15495	0.274357	no
Tram2	2.94448	2.17028	-0.440132	0.12905	0.237599	no
Trap1	52.1947	58.0261	0.152799	0.2897	0.440586	no
Trappc1	62.633	51.9366	-0.270171	0.09245	0.181173	no
Trappc10	25.8913	23.9939	-0.109805	0.4375	0.595511	no
Trappc11	16.9723	17.7494	0.064591	0.6535	0.782157	no
Trappc12	23.3363	23.1938	-0.00883978	0.95265	0.974605	no
Trappc13	15.4547	15.5506	0.00893055	0.95685	0.976835	no
Trappc2	17.8143	14.011	-0.346481	0.10025	0.193689	no
Trappc2l	53.8607	48.1095	-0.162909	0.3423	0.498972	no
Trappc3	39.4895	37.6028	-0.0706292	0.6426	0.773597	no
Trappc4	50.1143	44.7769	-0.162467	0.2921	0.443205	no
Trappc5	19.4599	19.9005	0.0322987	0.8385	0.907423	no
Trappc6a	32.6228	35.7707	0.132896	0.44035	0.598329	no
Trappc6b	51.4102	50.1801	-0.0349404	0.81365	0.892129	no
Trappc8	32.2513	30.9402	-0.0598765	0.6762	0.798935	no
Trappc9	12.8104	13.5903	0.0852662	0.6472	0.777199	no
Trat1	14.9875	36.4757	1.28317	5.00E-05	0.000236281	yes
Trdmt1	5.31253	4.83562	-0.1357	0.4737	0.630142	no
Treml2	1.14707	2.72622	1.24895	5.00E-05	0.000236281	yes
Trerf1	4.80505	4.97841	0.0511345	0.7641	0.860018	no
Trex1	49.8268	33.0899	-0.590529	0.00025	0.00103712	yes
Triap1	38.7053	37.4481	-0.047641	0.7622	0.85866	no
Trib1	4.36124	6.81705	0.644409	0.00035	0.0014053	yes
Trib2	6.86033	9.43754	0.460133	0.0045	0.0136593	yes
Trib3	1.36041	1.56728	0.204215	0.49045	0.645441	no
Trim11	28.7432	29.9995	0.0617173	0.6769	0.79944	no
Trim12a	78.4097	76.6526	-0.0326962	0.82165	0.897332	no
Trim12c	28.7017	27.6525	-0.0537296	0.7049	0.819496	no
Trim13	4.45386	4.46648	0.00408291	0.99125	0.99466	no
Trim14	26.8617	23.8792	-0.169799	0.24275	0.387511	no
Trim21	21.1885	20.4904	-0.0483323	0.74365	0.846285	no
Trim23	4.83646	4.76579	-0.0212365	0.90265	0.946135	no
Trim24	7.83207	6.54495	-0.259012	0.0867	0.171774	no
Trim25	24.8268	25.8069	0.0558593	0.69205	0.810538	no
Trim26	41.981	42.0797	0.00338695	0.98245	0.989911	no
Trim27	19.3892	19.1465	-0.0181721	0.9042	0.947141	no
Trim28	51.2442	49.4377	-0.0517775	0.69945	0.815605	no
Trim3	1.86214	1.66785	-0.158975	0.51	0.66334	no
Trim30a	44.4044	43.0104	-0.0460163	0.7471	0.848652	no
Trim30d	3.98113	3.40994	-0.223431	0.2349	0.379138	no
Trim32	2.90452	3.57665	0.300308	0.13235	0.242568	no
Trim33	14.3052	13.5424	-0.0790559	0.5851	0.72753	no
Trim34a	14.8609	13.126	-0.179098	0.25455	0.399197	no
Trim35	26.3898	28.0238	0.0866739	0.5519	0.699659	no
Trim36	1.5349	1.31479	-0.223314	0.3229	0.477917	no
Trim37	8.35108	7.53389	-0.148568	0.33265	0.488365	no
Trim39	26.7894	30.4051	0.18265	0.2045	0.341828	no

Trim41	16.6395	16.4336	-0.017961	0.90285	0.94626	no
Trim44	15.8474	16.3668	0.0465279	0.74615	0.847995	no
Trim46	5.98073	5.17042	-0.21004	0.25215	0.396819	no
Trim56	12.1959	14.2796	0.22756	0.136	0.247707	no
Trim59	27.9304	24.4051	-0.194653	0.18635	0.318027	no
Trim62	2.64689	2.51146	-0.0757744	0.7211	0.830668	no
Trim65	7.9104	7.74214	-0.0310186	0.85415	0.917327	no
Trim68	7.64073	8.67661	0.18342	0.2994	0.451602	no
Trim8	42.9752	40.2978	-0.0928045	0.5097	0.66306	no
Triobp	37.1293	35.6556	-0.0584311	0.6844	0.805052	no
Trip10	2.12152	2.27438	0.100376	0.6796	0.801489	no
Trip11	14.8248	15.4693	0.0613875	0.67245	0.796103	no
Trip12	49.9004	55.1429	0.144125	0.30845	0.46171	no
Trip13	6.50397	4.5169	-0.52599	0.0081	0.022802	yes
Trip4	17.7133	17.9647	0.0203261	0.92935	0.9614	no
Trip6	4.74944	4.183	-0.183219	0.40425	0.56321	no
Trit1	8.4263	8.98356	0.092389	0.605	0.743758	no
Trmt1	24.5447	28.1653	0.19851	0.17875	0.307548	no
Trmt10a	2.43304	2.16114	-0.170974	0.4123	0.571111	no
Trmt10b	5.92079	5.2348	-0.177655	0.3738	0.532506	no
Trmt10c	23.1194	23.3	0.0112245	0.9393	0.967115	no
Trmt11	6.65787	6.75213	0.0202806	0.9124	0.952014	no
Trmt112	98.1084	96.8347	-0.0188528	0.91705	0.954524	no
Trmt12	1.6	1.73796	0.119315	0.5983	0.738389	no
Trmt13	5.22203	5.4933	0.0730623	0.7518	0.85166	no
Trmt1l	20.2996	20.6951	0.027838	0.8547	0.917778	no
Trmt2a	14.3979	13.9737	-0.0431483	0.8398	0.908203	no
Trmt2b	14.2908	13.3563	-0.0975635	0.6427	0.773651	no
Trmt44	3.65863	3.60781	-0.0201821	0.916	0.953909	no
Trmt5	8.02987	8.37249	0.0602792	0.74655	0.8483	no
Trmt6	16.3099	14.5516	-0.164574	0.2877	0.438272	no
Trmt61a	4.74261	5.90041	0.315133	0.11045	0.20979	no
Trmu	7.06381	7.99339	0.17836	0.36985	0.528426	no
Trnau1ap	25.7892	25.9904	0.0112127	0.94695	0.971322	no
Trnt1	18.985	18.0577	-0.0722417	0.79815	0.882524	no
Troap	5.3365	2.88899	-0.885328	1.00E-04	0.000450026	yes
Trp53	51.821	55.1113	0.0888106	0.5425	0.69168	no
Trp53bp1	10.5428	11.3132	0.101751	0.55865	0.705416	no
Trp53bp2	11.0301	11.3472	0.0408846	0.7908	0.877959	no
Trp53i11	25.2967	27.0353	0.0958947	0.51625	0.66909	no
Trp53i13	12.5473	15.4377	0.299079	0.09565	0.186347	no
Trp53inp1	57.9717	51.7041	-0.165069	0.2439	0.388854	no
Trp53inp2	2.15794	2.06639	-0.0625455	0.76465	0.860349	no
Trpc1	1.0802	1.15414	0.0955219	0.72605	0.833988	no
Trpc4ap	75.4521	76.3254	0.0166035	0.9082	0.949429	no
Trpm4	2.80472	2.5804	-0.120264	0.53795	0.68788	no
Trpm7	24.2327	24.1006	-0.00788163	0.9573	0.977108	no
Trps1	1.0975	1.35855	0.307854	0.1042	0.199903	no
Trpt1	3.04361	4.20549	0.466489	0.8317	0.90343	no
Trpv2	66.5742	71.9864	0.11276	0.42205	0.580447	no
Trrap	20.7346	21.0984	0.0250907	0.85875	0.920092	no
Trub1	1.93679	3.20354	0.725999	0.00045	0.0017621	yes
Trub2	16.8082	16.9885	0.0153979	0.92445	0.958805	no
Tsacc	2.74938	2.00817	-0.453221	0.38445	0.54352	no
Tsc1	5.82928	6.35781	0.125211	0.40595	0.564909	no
Tsc2	15.2236	14.9552	-0.0256689	0.91135	0.951327	no
Tsc22d1	1.60705	2.14614	0.417329	0.15285	0.271367	no

Tsc22d2	5.20623	5.65784	0.120014	0.43125	0.589482	no
Tsc22d3	44.041	53.2403	0.273673	0.05955	0.126122	no
Tsc22d4	125.66	118.355	-0.0864128	0.5368	0.686932	no
Tsen15	7.4645	7.37569	-0.0172691	0.942	0.968581	no
Tsen2	2.2217	2.18541	-0.0237571	0.93235	0.96305	no
Tsen34	6.43201	6.6654	0.0514232	0.81065	0.8905	no
Tsen54	14.493	16.6217	0.197709	0.228	0.371039	no
Tsfm	18.0404	16.6482	-0.115865	0.5222	0.674152	no
Tsg101	33.5941	30.1852	-0.154366	0.3084	0.461665	no
Tsga10	2.36626	0.980593	-1.27088	1.00E-04	0.000450026	yes
Tshz1	9.83917	11.2651	0.195252	0.1909	0.324068	no
Tsn	39.6232	39.1048	-0.0189991	0.89465	0.941258	no
Tsnax	39.8446	42.4493	0.0913551	0.5248	0.676408	no
Tspan13	35.7065	48.2081	0.43309	0.0028	0.00898766	yes
Tspan14	39.089	39.6004	0.0187528	0.8995	0.944432	no
Tspan2	6.6837	8.77113	0.392115	0.01395	0.0365039	yes
Tspan3	8.37189	9.44596	0.174144	0.3424	0.499076	no
Tspan31	36.6024	45.0864	0.300755	0.0459	0.101181	no
Tspan32	51.7504	42.1897	-0.29468	0.04995	0.10869	no
Tspan4	2.00599	3.64742	0.86256	0.093	0.182069	no
Tspan5	10.1115	10.637	0.0730881	0.6504	0.779663	no
Tspo	240.02	219.228	-0.130723	0.3571	0.51491	no
Tspyl1	55.8201	59.7759	0.0987794	0.48595	0.641229	no
Tspyl2	7.42905	8.12294	0.128824	0.44295	0.600774	no
Tspyl3	16.686	16.4781	-0.0180906	0.90585	0.948152	no
Tspyl4	5.99251	6.62505	0.144771	0.38855	0.547682	no
Tsr1	10.5792	13.2415	0.323837	0.0894	0.176253	no
Tsr2	2.8901	3.47196	0.264632	0.1723	0.298743	no
Tsr3	36.0555	33.7506	-0.0953073	0.84695	0.912565	no
Tssc1	22.0345	22.5277	0.0319346	0.83865	0.9075	no
Tssc4	12.5671	10.1889	-0.302642	0.10925	0.207852	no
Tssk4	1.28161	1.31757	0.0399164	0.9159	0.953833	no
Tssk6	1.96782	2.22876	0.179647	0.86805	0.925747	no
Tst	1.53968	1.18799	-0.374113	0.35205	0.509481	no
Tsta3	23.9688	23.7125	-0.015508	0.9255	0.959344	no
Tstd1	1.45703	1.12859	-0.368516	0.55175	0.699544	no
Tstd2	12.3238	12.0308	-0.0347169	0.8208	0.896737	no
Tstd3	24.7239	20.7691	-0.25147	0.25385	0.398329	no
Ttbk2	0.925852	1.42504	0.622153	0.00085	0.00311418	yes
Ttc1	30.2308	26.2871	-0.201666	0.1998	0.335873	no
Ttc13	35.7043	40.4313	0.179377	0.21125	0.350677	no
Ttc14	14.4537	14.3102	-0.0143927	0.92045	0.956694	no
Ttc17	14.9733	15.1338	0.0153837	0.91695	0.954462	no
Ttc19	5.57698	5.38464	-0.050633	0.9535	0.975121	no
Ttc21b	5.52119	5.68798	0.0429362	0.78915	0.876956	no
Ttc27	3.98732	5.05567	0.342484	0.07495	0.152072	no
Ttc3	6.65635	7.19473	0.112209	0.5027	0.656823	no
Ttc32	9.26337	8.97242	-0.0460391	0.83215	0.90371	no
Ttc33	19.6405	17.8796	-0.135515	0.3998	0.558841	no
Ttc37	5.65504	6.67228	0.238643	0.128	0.236116	no
Ttc38	12.156	13.775	0.180384	0.2351	0.379366	no
Ttc39b	27.9031	29.5935	0.0848534	0.55165	0.69948	no
Ttc39c	39.645	37.0034	-0.0994811	0.48605	0.641325	no
Ttc4	20.3562	17.9275	-0.183293	0.2259	0.36859	no
Ttc5	36.8227	37.7429	0.0356085	0.80835	0.889011	no
Ttc7	70.8862	67.7466	-0.0653564	0.64595	0.776174	no
Ttc7b	33.7338	31.7909	-0.0855836	0.5534	0.700969	no

Ttc9c	21.1442	19.2377	-0.136324	0.35325	0.510851	no
Ttf1	18.159	18.4585	0.0236034	0.87125	0.927688	no
Ttf2	11.2848	9.25309	-0.286369	0.06085	0.128294	no
Tti1	10.5383	9.94411	-0.0837318	0.59435	0.735237	no
Tti2	7.78842	8.08798	0.0544483	0.866	0.924455	no
Ttk	5.22022	2.69187	-0.955499	5.00E-05	0.000236281	yes
Ttl	2.47514	2.48027	0.00298379	0.99265	0.995428	no
Ttll1	4.38242	4.64709	0.0846013	0.689	0.80828	no
Ttll12	14.9087	15.001	0.00890367	0.9537	0.975156	no
Ttll3	12.3623	11.341	-0.124395	0.8405	0.908779	no
Ttll4	5.70397	6.92342	0.279517	0.0889	0.175424	no
Ttll5	4.12283	4.33348	0.0718937	0.6764	0.799091	no
Ttpal	15.1922	15.1701	-0.0021055	0.9867	0.992253	no
Ttyh3	20.1928	21.7871	0.109633	0.44615	0.603838	no
Tuba1a	96.8282	77.1735	-0.327321	0.02195	0.054091	no
Tuba1b	152.905	93.7863	-0.70519	5.00E-05	0.000236281	yes
Tuba1c	36.2073	25.6233	-0.498824	7.00E-04	0.00261609	yes
Tuba4a	47.199	55.7233	0.239527	0.1888	0.321392	no
Tubb2a	3.85946	2.51751	-0.6164	0.0171	0.0434656	yes
Tubb3	4.20025	2.97364	-0.498244	0.0407	0.0913676	no
Tubb4a	2.95136	3.81849	0.371622	0.09795	0.190114	no
Tubb4b	152.156	117.686	-0.3706	0.00945	0.0261128	yes
Tubb5	266.81	205.762	-0.374833	0.00855	0.0239029	yes
Tubb6	0.976503	1.32905	0.444695	0.2078	0.346155	no
Tubd1	7.01934	6.59059	-0.0909267	0.63685	0.769075	no
Tube1	1.85577	1.5319	-0.276699	0.26595	0.412991	no
Tubg1	26.3581	20.7189	-0.3473	0.02975	0.0701013	no
Tubgcp2	15.7993	14.6533	-0.108634	0.5747	0.718637	no
Tubgcp3	24.1054	22.3181	-0.111144	0.436	0.594076	no
Tubgcp4	16.8831	17.0757	0.0163623	0.9489	0.972389	no
Tubgcp5	9.6787	9.52664	-0.0228463	0.8829	0.934524	no
Tubgcp6	12.7692	12.8256	0.00636165	0.97215	0.984706	no
Tufm	58.5388	59.3523	0.0199091	0.89035	0.938609	no
Tuft1	1.41697	2.32028	0.711493	0.00705	0.0201982	yes
Tulp3	1.48362	1.24647	-0.251275	0.3285	0.484034	no
Tulp4	19.7817	18.4138	-0.103385	0.463	0.620139	no
Tusc1	0.683073	1.09797	0.684728	0.12055	0.225049	no
Tusc2	14.0342	14.0227	-0.00118998	0.99595	0.997151	no
Tusc3	24.047	25.592	0.089835	0.5758	0.719681	no
Tut1	10.4444	12.676	0.279374	0.08435	0.167948	no
Tvp23a	2.36652	1.99621	-0.245502	0.6007	0.740354	no
Tvp23b	15.7561	16.4549	0.0626067	0.7065	0.820613	no
Twf1	21.3278	20.4357	-0.0616417	0.6827	0.803655	no
Twf2	183.439	178.43	-0.0399432	0.77835	0.869526	no
Twistnb	14.235	15.592	0.131365	0.41045	0.569409	no
Twsg1	4.28195	4.91045	0.197586	0.26175	0.407905	no
Txk	35.9908	31.3368	-0.199769	0.1751	0.302403	no
Txlna	24.8268	23.7097	-0.0664211	0.6408	0.772128	no
Txlng	19.9903	21.7086	0.118973	0.65405	0.782603	no
Txn1	219.552	188.303	-0.221503	0.12005	0.224294	no
Txn2	104.937	102.465	-0.034393	0.80945	0.889736	no
Txndc11	21.8054	20.9568	-0.0572681	0.73385	0.839409	no
Txndc12	28.6079	26.6944	-0.0998812	0.65295	0.781737	no
Txndc15	25.2692	27.2216	0.107373	0.49175	0.646465	no
Txndc16	5.29149	4.7375	-0.159548	0.3555	0.513179	no
Txndc17	65.3693	53.6722	-0.284437	0.05165	0.111748	no
Txndc5	90.461	83.4897	-0.115697	0.41605	0.574749	no

Txndc9	17.0108	14.7202	-0.20866	0.15605	0.275917	no
Txnip	194.617	196.734	0.015611	0.91445	0.953069	no
Txnl1	28.9441	25.1149	-0.204722	0.16695	0.291437	no
Txnl4a	81.1434	79.0724	-0.0372996	0.8051	0.887012	no
Txnl4b	4.88263	4.26633	-0.194665	0.3726	0.531258	no
Txnrd1	52.0689	53.8062	0.0473485	0.74	0.843682	no
Txnrd2	5.39548	6.21773	0.204638	0.5089	0.662459	no
Tyk2	47.8812	41.3594	-0.211246	0.13705	0.249298	no
Tyms	17.3614	15.8905	-0.127718	0.3877	0.546855	no
Tyro3	2.02601	1.29073	-0.650463	0.0044	0.0133909	yes
Tyrobp	5.41259	4.26209	-0.344758	0.33685	0.493054	no
Tysnd1	23.31	21.1283	-0.141772	0.50215	0.656299	no
Tyw1	14.5078	13.9957	-0.0518447	0.73635	0.841301	no
Tyw5	9.57461	9.20568	-0.0566901	0.82695	0.900668	no
U2af1	120.32	124.131	0.0449843	0.7527	0.852201	no
U2af1l4	39.8506	38.3769	-0.0543647	0.7401	0.843755	no
U2af2	149.014	148.671	-0.00331841	0.98255	0.98997	no
U2surp	14.2681	13.9456	-0.032987	0.8127	0.891697	no
Uap1	22.8401	19.9526	-0.194987	0.20015	0.336253	no
Uap1l1	9.63717	11.2766	0.226656	0.20305	0.339984	no
Uba1	143.981	139.577	-0.0448114	0.7538	0.852913	no
Uba2	66.8069	63.4367	-0.0746795	0.5997	0.739535	no
Uba3	35.0911	29.7696	-0.237266	0.1065	0.203497	no
Uba5	22.5805	20.3763	-0.148189	0.32005	0.474665	no
Uba52	4001.74	3991.66	-0.00363818	0.9796	0.988479	no
Uba6	5.02713	4.87576	-0.0441097	0.7891	0.876941	no
Uba7	36.6355	37.8746	0.0479879	0.7395	0.843398	no
Ubac1	11.5744	11.7787	0.0252421	0.87005	0.926941	no
Ubac2	58.2211	51.8802	-0.166358	0.405	0.563976	no
Ubald1	71.2813	70.0368	-0.0254097	0.86245	0.922241	no
Ubald2	26.9548	23.5951	-0.192058	0.52465	0.676301	no
Ubap1	21.3875	20.5084	-0.0605536	0.686	0.806087	no
Ubap2	20.8747	22.4313	0.103756	0.4728	0.629324	no
Ubap2l	66.7636	66.1318	-0.0137185	0.93495	0.964634	no
Ubash3a	48.5481	50.1728	0.0474892	0.73615	0.841168	no
Ubash3b	14.5778	20.7284	0.507834	0.00065	0.00244919	yes
Ubb	1039.33	1043.13	0.00526143	0.97175	0.984428	no
Ubc	119.331	114.052	-0.0652766	0.6458	0.776073	no
Ube2a	36.7283	32.6242	-0.170951	0.25285	0.397166	no
Ube2b	85.7142	82.0041	-0.0638376	0.6537	0.78233	no
Ube2c	81.2846	44.6212	-0.865252	5.00E-05	0.000236281	yes
Ube2d1	43.3265	44.3181	0.0326477	0.82805	0.901198	no
Ube2d2a	91.6652	91.8524	0.00294255	0.98345	0.99041	no
Ube2d3	226.965	215.348	-0.0758017	0.59885	0.738848	no
Ube2e1	37.1514	39.6767	0.0948737	0.56635	0.711663	no
Ube2e3	13.8209	11.6817	-0.242595	0.1685	0.293395	no
Ube2f	40.2817	40.1398	-0.00508971	0.97355	0.985502	no
Ube2g1	16.8698	14.3675	-0.231628	0.12105	0.225762	no
Ube2g2	112.426	97.5395	-0.204924	0.1439	0.258389	no
Ube2h	69.8116	70.4525	0.013184	0.92535	0.959259	no
Ube2i	158.41	153.694	-0.0436041	0.75985	0.857148	no
Ube2j1	41.7373	38.7315	-0.107828	0.4551	0.612849	no
Ube2j2	6.27798	5.82423	-0.108233	0.7217	0.831006	no
Ube2k	14.6946	13.16	-0.159118	0.2795	0.429058	no
Ube2l3	85.4154	85.7695	0.00596904	0.9667	0.981919	no
Ube2l6	3.13793	2.64166	-0.248368	0.3685	0.527062	no
Ube2m	74.9994	69.9025	-0.101535	0.49175	0.646465	no

Ube2n	32.1382	29.1909	-0.138769	0.3284	0.483947	no
Ube2o	6.98825	7.43751	0.0898874	0.5663	0.711626	no
Ube2q1	65.2475	62.9401	-0.0519445	0.7142	0.825928	no
Ube2q2	10.8085	11.3348	0.0685888	0.66495	0.790472	no
Ube2r2	21.532	22.6152	0.0708103	0.6278	0.761888	no
Ube2s	179.234	141.396	-0.342096	0.01685	0.0429523	yes
Ube2t	12.3468	8.26222	-0.579538	0.00585	0.01717	yes
Ube2v1	98.4762	87.9712	-0.162745	0.254	0.398556	no
Ube2v2	7.53417	7.17556	-0.0703561	0.64605	0.776254	no
Ube2w	6.22531	5.56199	-0.162545	0.3626	0.520861	no
Ube2z	29.2941	29.7036	0.0200265	0.8897	0.938246	no
Ube3a	11.429	11.9224	0.0609812	0.6893	0.808484	no
Ube3b	24.3867	24.5564	0.0100077	0.94345	0.96938	no
Ube3c	14.5655	14.0469	-0.052297	0.72035	0.830115	no
Ube4a	28.4028	29.1472	0.0373274	0.7899	0.877415	no
Ube4b	18.5969	19.1775	0.044359	0.75785	0.855685	no
Ubfd1	7.11562	7.97693	0.164843	0.2943	0.445805	no
Ubiad1	4.77714	4.55702	-0.0680536	0.7184	0.82864	no
Ubl3	40.516	41.0133	0.0175993	0.89945	0.944408	no
Ubl4	12.828	11.0961	-0.209249	0.3715	0.530064	no
Ubl5	69.2689	74.6023	0.107012	0.50215	0.656299	no
Ubl7	50.6265	50.0452	-0.0166594	0.91305	0.952439	no
Ublcp1	14.1395	14.3834	0.0246789	0.8781	0.931847	no
Ubn1	30.7642	30.8999	0.00635022	0.9609	0.97893	no
Ubn2	7.10347	7.80486	0.135849	0.3367	0.492875	no
Ubox5	2.99108	3.47099	0.214679	0.37515	0.533858	no
Ubp1	43.3939	40.7447	-0.0908799	0.54465	0.69342	no
Ubqln1	39.2496	39.3913	0.00519947	0.9706	0.983969	no
Ubqln2	19.8298	20.4523	0.044595	0.75955	0.857015	no
Ubqln4	14.0046	15.6407	0.1594	0.2945	0.446003	no
Ubr1	13.9112	16.019	0.203532	0.15095	0.268608	no
Ubr2	30.4954	33.6085	0.140235	0.324	0.479093	no
Ubr3	9.55293	9.65144	0.0148008	0.9167	0.954324	no
Ubr4	34.8839	39.8038	0.190343	0.18725	0.319293	no
Ubr5	24.2913	26.5407	0.127767	0.36685	0.525266	no
Ubr7	25.3344	24.3193	-0.0589932	0.6848	0.805334	no
Ubtd1	4.78396	4.13772	-0.209371	0.37425	0.532911	no
Ubtd2	1.42276	1.24617	-0.191199	0.47135	0.627844	no
Ubtf	67.0955	64.0846	-0.0662389	0.6425	0.773503	no
Ubxn1	131.427	120.412	-0.126289	0.37955	0.538545	no
Ubxn11	20.2286	17.8839	-0.177737	0.27675	0.425791	no
Ubxn2a	17.0688	20.1929	0.242487	0.1105	0.209845	no
Ubxn2b	9.76619	8.00577	-0.286756	0.06125	0.129007	no
Ubxn4	34.917	36.0055	0.0442863	0.75445	0.853416	no
Ubxn6	47.193	45.9154	-0.0395954	0.78165	0.871722	no
Ubxn7	8.17134	8.1262	-0.00799181	0.95685	0.976835	no
Ubxn8	13.342	12.5936	-0.0832819	0.7785	0.869611	no
Uchl1	0.595523	1.11989	0.911124	0.07325	0.14918	no
Uchl3	33.159	31.2136	-0.0872253	0.6031	0.742275	no
Uchl5	19.9182	18.865	-0.0783716	0.62005	0.755913	no
Uck1	37.7864	36.7805	-0.0389262	0.7946	0.88035	no
Uck2	46.1226	45.1135	-0.0319148	0.8318	0.903524	no
Uckl1	40.1036	38.152	-0.0719735	0.70775	0.821483	no
Ucp2	193.902	166.041	-0.223788	0.12335	0.229125	no
Uevld	2.94378	2.64408	-0.154905	0.4068	0.565679	no
Ufc1	92.4526	83.3289	-0.149896	0.30845	0.46171	no
Ufd1l	37.0071	35.9985	-0.0398655	0.7891	0.876941	no

Ufl1	10.7827	11.4333	0.0845256	0.58125	0.724325	no
Ufm1	13.7378	13.0178	-0.0776618	0.5949	0.735685	no
Ufsp1	1.27274	1.02187	-0.31673	0.48375	0.639258	no
Ufsp2	16.0011	14.154	-0.176965	0.3141	0.467895	no
Ugcg	70.837	52.6298	-0.428622	0.0031	0.00983478	yes
Ugdh	26.0304	22.8091	-0.190585	0.2054	0.343015	no
Uggt1	26.185	25.9531	-0.012834	0.9272	0.960312	no
Ugp2	18.839	15.6111	-0.271152	0.36625	0.524631	no
Uhmk1	23.065	23.3438	0.0173287	0.9076	0.949153	no
Uhrf1	27.2549	17.3357	-0.652775	5.00E-05	0.000236281	yes
Uhrf1bp1l	15.8266	18.5064	0.225669	0.1155	0.217433	no
Uhrf2	29.7262	29.9939	0.0129322	0.92865	0.961056	no
Uimc1	45.0119	43.3457	-0.0544174	0.7012	0.816742	no
Ulk1	7.23557	7.9559	0.136918	0.3709	0.529486	no
Ulk2	14.3089	14.0985	-0.0213679	0.88305	0.934556	no
Ulk3	20.2591	21.5373	0.088265	0.555	0.702214	no
Umps	12.3015	11.5736	-0.0880035	0.57475	0.718674	no
Unc119	35.1869	30.7486	-0.194521	0.209	0.347711	no
Unc119b	47.4859	53.5082	0.172262	0.2262	0.368935	no
Unc13d	69.3656	70.0517	0.0141993	0.9227	0.95797	no
Unc45a	26.5667	32.9343	0.309971	0.0299	0.0703491	no
Unc50	28.0807	28.278	0.0101035	0.9485	0.972248	no
Unc5a	1.21009	1.27142	0.0713281	0.78535	0.874329	no
Unc5cl	7.89825	3.24565	-1.28303	5.00E-05	0.000236281	yes
Unc93b1	12.6011	11.3723	-0.148023	0.36885	0.527403	no
Ung	4.9888	3.44218	-0.535372	0.0156	0.0401619	yes
Unk	12.0153	11.8982	-0.0141351	0.92185	0.957529	no
Unkl	27.8224	25.7911	-0.109374	0.5692	0.713973	no
Upf1	37.7617	39.7445	0.0738292	0.6006	0.740283	no
Upf2	12.3534	12.8653	0.0585823	0.6939	0.811762	no
Upf3a	37.7176	36.6578	-0.0411174	0.78655	0.875057	no
Upf3b	20.4906	18.6831	-0.133229	0.3922	0.55133	no
Upk1a	1.76793	1.80917	0.0332699	0.9249	0.958971	no
Uprt	5.38436	6.06156	0.170914	0.4782	0.634239	no
Uqcc1	10.6532	11.5868	0.121195	0.45635	0.613876	no
Uqcc2	85.655	83.1797	-0.0423061	0.80425	0.886574	no
Uqcr10	235.589	236.762	0.00716292	0.9608	0.978922	no
Uqcr11	146.095	136.764	-0.0952225	0.55085	0.698754	no
Uqcrb	167.046	167.318	0.00234859	0.98835	0.993134	no
Uqcrc1	113.244	108.67	-0.0594842	0.6729	0.796422	no
Uqcrc2	112.533	107.606	-0.0645814	0.652	0.780984	no
Uqcrfs1	106.837	93.4079	-0.19379	0.17495	0.302233	no
Uqcrh	646.311	600.753	-0.105457	0.45925	0.616579	no
Uqcrq	179.941	175.863	-0.0330695	0.82255	0.89776	no
Urb1	4.34882	5.77858	0.410091	0.0089	0.0247598	yes
Urb2	3.66238	4.67468	0.352086	0.0371	0.0844435	no
Urgcp	5.35172	5.79434	0.114642	0.5028	0.656929	no
Uri1	13.9696	19.8891	0.509689	5.00E-04	0.00193907	yes
Urm1	19.6363	18.6017	-0.078088	0.60725	0.745511	no
Urod	39.2136	36.6324	-0.0982339	0.52425	0.67597	no
Uros	12.3867	12.4053	0.00216684	0.9862	0.99192	no
Usb1	25.8256	25.2197	-0.0342492	0.8245	0.898984	no
Use1	187.24	193.118	0.0445924	0.7548	0.853607	no
Usf1	39.8676	38.7976	-0.0392483	0.79495	0.880586	no
Usf2	64.8312	66.4555	0.0356998	0.80105	0.884317	no
Usmg5	318.12	290.529	-0.130891	0.44905	0.606461	no
Uso1	30.1372	30.5414	0.0192208	0.89485	0.941342	no

Usp1	43.2628	35.6633	-0.278687	0.05175	0.111927	no
Usp10	12.9113	13.0732	0.017983	0.9049	0.947635	no
Usp11	14.8163	14.1935	-0.0619526	0.6877	0.807305	no
Usp12	17.7002	18.8701	0.0923309	0.5246	0.676249	no
Usp14	14.5496	14.0435	-0.0510841	0.7957	0.881002	no
Usp15	39.5151	42.4731	0.104148	0.46345	0.62053	no
Usp16	26.7948	25.7036	-0.0599783	0.8391	0.907766	no
Usp18	59.8667	92.2416	0.623664	5.00E-05	0.000236281	yes
Usp19	42.9727	42.8039	-0.00567985	0.96805	0.982626	no
Usp20	15.8766	17.0641	0.104062	0.4769	0.63305	no
Usp21	17.3352	17.4201	0.00704864	0.9649	0.980867	no
Usp22	15.8353	16.0945	0.0234183	0.87505	0.929854	no
Usp24	22.9325	25.0866	0.12952	0.3647	0.522962	no
Usp25	66.1492	62.6941	-0.0773945	0.57675	0.720499	no
Usp28	6.2469	9.56455	0.614557	2.00E-04	0.0008488	yes
Usp3	78.1509	66.7886	-0.22666	0.1123	0.212423	no
Usp30	13.0089	11.0045	-0.241401	0.1313	0.241006	no
Usp31	2.66734	2.84954	0.0953287	0.55825	0.70505	no
Usp32	7.65825	8.11689	0.083912	0.58055	0.723683	no
Usp33	9.11407	9.92345	0.122748	0.4211	0.579547	no
Usp34	20.4898	21.8979	0.095888	0.55955	0.706072	no
Usp35	1.58216	1.24829	-0.341943	0.1623	0.284855	no
Usp36	14.3214	15.2963	0.095007	0.5136	0.666524	no
Usp37	5.14767	5.16745	0.00553496	0.98585	0.991766	no
Usp38	27.1476	26.5295	-0.0332258	0.81325	0.891885	no
Usp39	33.0037	31.4687	-0.0687073	0.6349	0.767706	no
Usp4	50.9561	48.317	-0.0767233	0.58275	0.725656	no
Usp40	2.4769	2.40376	-0.0432405	0.81735	0.89463	no
Usp42	10.0077	9.90644	-0.0146762	0.9227	0.95797	no
Usp45	8.02595	7.44854	-0.107714	0.47825	0.63427	no
Usp46	4.23654	3.59823	-0.235603	0.213	0.352885	no
Usp47	25.1563	23.6434	-0.0894859	0.5288	0.679961	no
Usp48	82.1444	86.3812	0.072555	0.6104	0.748025	no
Usp49	2.14555	1.84447	-0.218141	0.2707	0.418618	no
Usp5	32.9984	31.1619	-0.0826126	0.56315	0.709005	no
Usp53	2.76473	4.30985	0.640499	9.00E-04	0.00327966	yes
Usp54	1.05578	1.04456	-0.0154088	0.9835	0.990418	no
Usp6nl	1.21786	0.783035	-0.637202	0.0134	0.035266	yes
Usp7	40.782	41.6152	0.0291789	0.8332	0.904321	no
Usp8	23.3245	22.4715	-0.0537456	0.71085	0.823632	no
Usp9x	10.963	11.0556	0.0121263	0.93375	0.96396	no
Uspl1	16.2477	16.4997	0.0222066	0.8875	0.93692	no
Ust	4.53033	6.2096	0.454884	0.00895	0.0248832	yes
Utf1	0.816997	1.31401	0.685577	0.1215	0.226433	no
Utp11l	48.9348	52.2633	0.0949375	0.53815	0.687986	no
Utp14a	18.3825	18.0736	-0.0244492	0.8839	0.935049	no
Utp15	9.82122	10.5792	0.107251	0.4838	0.6393	no
Utp18	18.5686	19.4945	0.0701964	0.64195	0.773065	no
Utp20	3.73747	4.80701	0.363078	0.0209	0.0518292	no
Utp23	8.23177	7.23673	-0.185865	0.2709	0.418835	no
Utp3	48.8002	48.9359	0.00400619	0.97875	0.98811	no
Utp6	13.9829	14.2715	0.0294796	0.8452	0.91164	no
Utrn	26.6782	30.8656	0.210342	0.1387	0.251076	no
Uty	4.96911	5.02686	0.0166699	0.9201	0.956443	no
Uvrag	18.6289	18.449	-0.0140043	0.92535	0.959259	no
Uvssa	4.06765	4.89213	0.266268	0.0946	0.184643	no
Uxs1	7.05405	6.07494	-0.215582	0.3316	0.487279	no

Uxt	20.3072	17.5294	-0.212216	0.2903	0.441223	no
Vac14	23.5353	24.027	0.029828	0.8368	0.906486	no
Vamp1	5.95678	6.00845	0.0124608	0.9416	0.968362	no
Vamp2	47.5391	46.8875	-0.0199121	0.88765	0.937008	no
Vamp3	43.9261	42.627	-0.0433083	0.7643	0.860119	no
Vamp4	12.3599	12.2856	-0.00870989	0.9573	0.977108	no
Vamp5	7.00364	8.25485	0.237136	0.21795	0.358862	no
Vamp8	99.8538	125.298	0.327469	0.02725	0.0650993	no
Vapa	57.4012	54.1734	-0.0834961	0.56705	0.712213	no
Vapb	14.77	14.1934	-0.0574501	0.68845	0.807904	no
Vars	29.7045	35.2725	0.247862	0.08335	0.166299	no
Vars2	6.12862	7.22808	0.238049	0.14575	0.261014	no
Vasn	7.15249	10.1084	0.499032	0.3187	0.47313	no
Vasp	355.662	369.977	0.0569273	0.69985	0.815802	no
Vat1	10.807	10.4708	-0.0455917	0.77305	0.865894	no
Vav1	59.0225	59.8903	0.021059	0.8875	0.93692	no
Vav2	2.04829	1.01244	-1.01659	0.00015	0.000653255	yes
Vav3	3.0853	4.67732	0.60027	0.00095	0.00343861	yes
Vbp1	45.3399	46.5601	0.0383117	0.7922	0.878781	no
Vcl	3.96606	3.50978	-0.176326	0.3108	0.464351	no
Vcp	117.495	114.106	-0.0422232	0.76385	0.859819	no
Vcpip1	18.6985	19.9656	0.0945903	0.53415	0.684621	no
Vcpkmt	19.3025	14.2797	-0.434819	0.02005	0.0499671	yes
Vdac1	62.1773	60.6485	-0.035915	0.8005	0.884028	no
Vdac2	114.2	107.468	-0.0876614	0.54405	0.692956	no
Vdac3	67.3186	60.0929	-0.163812	0.25855	0.403998	no
Vegfb	3.95183	5.08464	0.363626	0.13875	0.251148	no
Vezf1	23.3078	22.9825	-0.020278	0.88785	0.937107	no
Vezt	3.36422	3.08837	-0.123426	0.49555	0.650165	no
Vgll4	26.3999	23.9688	-0.139375	0.3976	0.556681	no
Vhl	12.0208	13.4809	0.165382	0.295	0.446511	no
Vim	863.705	818.685	-0.0772305	0.6157	0.752547	no
Vimp	68.9662	65.3991	-0.0766194	0.60065	0.740306	no
Vipas39	18.8054	18.365	-0.0341865	0.8243	0.898947	no
Vipr1	5.26904	8.80487	0.74076	5.00E-05	0.000236281	yes
Vkorc1	12.9117	14.8967	0.206311	0.3392	0.495599	no
Vkorc1l1	5.0166	4.72652	-0.0859319	0.60605	0.744556	no
Vma21	8.19886	7.90399	-0.0528425	0.7316	0.837813	no
Vmac	5.32036	7.0792	0.412062	0.0334	0.0772728	no
Vmn1r58	2.96629	3.12003	0.0728978	0.74345	0.84614	no
Vmn2r96	1.81405	1.85197	0.0298407	0.91305	0.952439	no
Vmp1	29.2267	28.4555	-0.038578	0.79155	0.878391	no
Vopp1	30.3117	21.4205	-0.500885	0.00075	0.00278555	yes
Vprbp	12.1509	13.0481	0.102773	0.4778	0.633887	no
Vps11	21.0072	20.2418	-0.0535502	0.71145	0.824016	no
Vps13a	11.1845	12.9382	0.21014	0.13945	0.252201	no
Vps13b	13.3327	14.9361	0.163842	0.2415	0.386065	no
Vps13c	8.21486	9.26708	0.17388	0.22205	0.363763	no
Vps13d	16.1892	17.3254	0.0978638	0.4925	0.647222	no
Vps16	36.3614	33.4913	-0.118621	0.475	0.631348	no
Vps18	3.64781	4.12858	0.178615	0.3214	0.476267	no
Vps25	88.0554	86.5673	-0.0245892	0.86915	0.92636	no
Vps26a	18.1198	17.8878	-0.0185868	0.9025	0.946118	no
Vps26b	24.827	25.2112	0.0221571	0.8823	0.934191	no
Vps28	208.781	206.4	-0.0165452	0.91065	0.950891	no
Vps29	77.0015	68.1926	-0.175271	0.24215	0.386903	no
Vps33a	17.2711	18.4229	0.0931415	0.5255	0.67705	no

Vps33b	17.1096	18.3396	0.100156	0.5104	0.663714	no
Vps35	42.9938	39.5936	-0.118863	0.3991	0.558128	no
Vps36	58.1755	51.5488	-0.174473	0.2362	0.380697	no
Vps37a	6.56417	6.94365	0.0810818	0.74835	0.849415	no
Vps37b	32.0278	27.9638	-0.195763	0.1797	0.308911	no
Vps37c	11.7883	12.5968	0.0957016	0.55785	0.704658	no
Vps39	18.421	18.1332	-0.0227168	0.87925	0.932366	no
Vps41	28.3014	27.6482	-0.0336912	0.8132	0.891872	no
Vps45	8.71828	8.38648	-0.0559787	0.74495	0.847218	no
Vps4a	42.5755	36.2647	-0.231455	0.11635	0.218742	no
Vps4b	45.6211	43.3758	-0.0728084	0.59785	0.73804	no
Vps51	20.3991	20.0417	-0.025501	0.86795	0.925724	no
Vps52	41.1166	40.3919	-0.0256564	0.861	0.921514	no
Vps53	24.3063	24.586	0.0165055	0.9114	0.951351	no
Vps54	29.0566	34.6569	0.254274	0.1195	0.223509	no
Vps72	41.4877	41.4212	-0.00231523	0.98825	0.993076	no
Vps8	4.95168	4.52435	-0.130205	0.4371	0.59507	no
Vps9d1	11.5186	11.1838	-0.042549	0.8111	0.890717	no
Vrk1	18.7774	20.597	0.133439	0.3659	0.5243	no
Vrk2	21.4411	18.2346	-0.233701	0.1872	0.319223	no
Vrk3	83.5907	72.5919	-0.203535	0.1513	0.269075	no
Vsig10l	4.48556	4.10282	-0.128672	0.48905	0.644079	no
Vta1	43.1433	37.2481	-0.211968	0.1607	0.282595	no
Vti1a	8.84381	9.36199	0.0821466	0.61085	0.748343	no
Vti1b	63.9891	61.4698	-0.0579473	0.69475	0.812339	no
Vwa5a	2.47357	4.66611	0.91563	5.00E-05	0.000236281	yes
Vwa8	3.713	4.72782	0.348588	0.05425	0.116502	no
Vwa9	20.2831	19.0757	-0.0885405	0.55765	0.704494	no
Wac	37.7155	37.9381	0.00848898	0.951	0.97375	no
Wapal	32.7728	31.6532	-0.0501493	0.72335	0.832119	no
Wars	35.0297	31.6233	-0.147591	0.3101	0.46362	no
Wars2	5.1608	4.60321	-0.164954	0.33475	0.490672	no
Was	117.778	110.47	-0.0924236	0.51485	0.667668	no
Wasf2	60.5568	59.2545	-0.0313657	0.82445	0.898984	no
Wash	22.9199	22.6524	-0.0169388	0.90735	0.94899	no
Wasl	10.5849	10.7998	0.0289848	0.8729	0.928604	no
Wbp1	21.9549	21.7817	-0.0114252	0.94235	0.968687	no
Wbp11	41.4762	43.4058	0.0656055	0.6417	0.772883	no
Wbp1l	44.0058	35.9573	-0.291411	0.04255	0.0949259	no
Wbp2	86.7936	77.1366	-0.170174	0.23075	0.374168	no
Wbp4	10.512	9.70959	-0.114555	0.81615	0.89376	no
Wbp5	27.3808	21.9179	-0.321052	0.06415	0.133829	no
Wbscr16	4.90104	6.194	0.337783	0.078	0.157275	no
Wbscr22	30.5887	31.0697	0.02251	0.90325	0.946553	no
Wbscr27	2.03491	3.05238	0.58497	0.01155	0.0310715	yes
Wdfy1	10.946	14.3524	0.390887	0.0877	0.173502	no
Wdfy2	3.03081	4.68858	0.629447	0.0052	0.0154863	yes
Wdhd1	9.1712	7.08782	-0.371769	0.019	0.0476821	yes
Wdr1	223.705	197.319	-0.181071	0.2068	0.344888	no
Wdr11	12.5286	13.4572	0.103147	0.49545	0.650118	no
Wdr12	5.47048	5.73654	0.0685119	0.7257	0.833735	no
Wdr13	10.8809	12.3601	0.183888	0.2273	0.370233	no
Wdr18	10.2731	11.4318	0.154178	0.32865	0.484184	no
Wdr20	8.20424	8.22522	0.00368389	0.98525	0.991501	no
Wdr24	15.479	15.0285	-0.0426037	0.7748	0.867207	no
Wdr25	1.50201	1.75537	0.224881	0.3884	0.54758	no
Wdr26	55.8103	61.7266	0.145361	0.3059	0.458837	no

Wdr3	21.522	22.901	0.0895957	0.54015	0.689681	no
Wdr31	1.86152	0.737584	-1.3356	5.00E-05	0.000236281	yes
Wdr33	27.6084	29.389	0.0901717	0.67395	0.797143	no
Wdr36	20.725	22.4793	0.117222	0.4319	0.590085	no
Wdr37	16.1623	18.2291	0.173607	0.2338	0.377803	no
Wdr4	6.56798	7.05473	0.103142	0.5419	0.691078	no
Wdr41	7.54769	8.22455	0.123901	0.44765	0.605056	no
Wdr43	25.2266	27.3142	0.114709	0.42295	0.581368	no
Wdr44	12.3559	12.0136	-0.0405258	0.7871	0.875545	no
Wdr45	28.8145	27.0476	-0.0912961	0.55535	0.702443	no
Wdr45b	26.7673	24.5958	-0.122059	0.40695	0.565799	no
Wdr46	12.2378	14.8722	0.28127	0.0843	0.167877	no
Wdr47	5.37323	5.49654	0.0327339	0.8478	0.913147	no
Wdr48	20.1425	18.4774	-0.124477	0.4318	0.590039	no
Wdr5	29.6999	26.9838	-0.138366	0.3415	0.498073	no
Wdr53	13.5795	11.4677	-0.243854	0.19695	0.332181	no
Wdr55	23.0837	24.2228	0.0694958	0.66215	0.788645	no
Wdr59	4.33675	4.6547	0.102075	0.5432	0.692323	no
Wdr5b	6.09506	5.61422	-0.118554	0.5516	0.699442	no
Wdr6	11.0337	11.4213	0.0498137	0.82155	0.897237	no
Wdr61	29.0226	28.2313	-0.0398826	0.806	0.887491	no
Wdr62	3.36965	2.02739	-0.732976	1.00E-04	0.000450026	yes
Wdr7	6.05307	6.10234	0.0116949	0.94015	0.967637	no
Wdr70	8.00466	7.57675	-0.07926	0.6624	0.788823	no
Wdr73	9.22055	9.41089	0.0294788	0.8619	0.921932	no
Wdr74	24.7053	26.5736	0.10517	0.5228	0.674606	no
Wdr75	13.6905	15.1207	0.143349	0.3567	0.514501	no
Wdr76	6.31552	4.12542	-0.614359	0.00025	0.00103712	yes
Wdr77	13.8307	13.0599	-0.0827352	0.8118	0.891125	no
Wdr78	2.42412	2.03932	-0.249374	0.23995	0.385061	no
Wdr8	17.2758	17.2367	-0.00326918	0.9861	0.99189	no
Wdr81	11.3703	11.9798	0.0753329	0.6068	0.745166	no
Wdr82	57.804	53.6364	-0.107959	0.44575	0.603412	no
Wdr83	20.3197	19.9399	-0.0272156	0.90725	0.948941	no
Wdr89	2.29223	2.77474	0.275603	0.24495	0.390035	no
Wdr90	4.45313	4.55993	0.0341903	0.9173	0.954657	no
Wdr91	12.7193	13.4714	0.0828819	0.60195	0.741403	no
Wdr92	7.77121	9.49441	0.28894	0.0782	0.157588	no
Wdr95	26.0306	18.8797	-0.463372	0.00245	0.0079866	yes
Wdsub1	3.19086	3.25	0.0264912	0.90585	0.948152	no
Wdtc1	20.5112	22.0239	0.102662	0.4793	0.63509	no
Wdyhv1	31.6536	31.4933	-0.00732504	0.96285	0.980057	no
Wee1	4.18633	3.18792	-0.393072	0.0427	0.0951973	no
Wfikkn1	1.28272	1.46813	0.194768	0.48735	0.642511	no
Wfikkn2	18.1067	44.3874	1.29363	5.00E-05	0.000236281	yes
Whamm	16.8738	16.3225	-0.047918	0.7493	0.850193	no
Whsc1	20.3542	16.1052	-0.337805	0.01775	0.0449043	yes
Whsc1l1	62.9586	68.4203	0.120022	0.43405	0.592311	no
Wibg	76.4527	82.9017	0.116836	0.4277	0.586048	no
Wipf1	126.244	126.148	-0.00109923	0.9943	0.996304	no
Wipf2	7.98892	8.10784	0.0213159	0.88615	0.936237	no
Wipi2	17.0928	17.5496	0.0380517	0.79225	0.878781	no
Wiz	13.029	13.3141	0.0312285	0.83495	0.905469	no
Wls	7.31234	14.6437	1.00188	5.00E-05	0.000236281	yes
Wnk1	54.7199	54.8732	0.00403741	0.97715	0.987291	no
Wrap53	16.8628	16.229	-0.0552679	0.73695	0.841741	no
Wrb	2.18781	2.43471	0.154264	0.5201	0.672191	no

Wrn	14.0082	13.7752	-0.0241949	0.86495	0.92389	no
Wrnip1	41.3591	43.3506	0.0678494	0.64	0.771493	no
Wsb1	32.1689	28.8097	-0.159116	0.2805	0.430285	no
Wsb2	20.3354	19.9701	-0.0261523	0.85675	0.918888	no
Wscd2	1.92535	1.59587	-0.270779	0.2164	0.357035	no
Wtap	68.097	65.7172	-0.0513206	0.74185	0.845123	no
Wwox	2.57335	2.74493	0.0931192	0.6915	0.810136	no
Wwp1	8.9813	7.91164	-0.182946	0.2239	0.366131	no
Wwp2	25.1015	24.6618	-0.0254962	0.8588	0.920092	no
Xab2	44.3873	44.4214	0.00110921	0.99345	0.995905	no
Xaf1	13.2886	11.4217	-0.218406	0.1735	0.300308	no
Xbp1	29.1864	29.7929	0.0296755	0.83365	0.904573	no
Xcl1	3.83824	3.11621	-0.300652	0.48845	0.643493	no
Xcr1	1.53239	1.39266	-0.137942	0.54995	0.697989	no
Xdh	21.8567	15.9642	-0.453237	0.0017	0.005774	yes
Xiap	24.3052	21.7211	-0.162166	0.24985	0.395366	no
Xk	1.56377	1.27085	-0.299235	0.17995	0.309258	no
Xkr8	3.19988	2.3455	-0.448123	0.02095	0.0519313	no
Xlr	1.79646	2.695	0.585128	0.0938	0.18335	no
Xlr3b	1.09902	2.10774	0.939485	0.00765	0.0217092	yes
Xlr4a	21.3802	19.1994	-0.155209	0.36665	0.525044	no
Xlr4b	16.5439	15.4719	-0.0966458	0.58735	0.729428	no
Xlr4c	9.43986	8.57094	-0.139312	0.4937	0.648437	no
Xndc1	10.5449	10.5462	0.000177904	0.9992	0.999285	no
Xntrpc	1.31891	1.67298	0.343071	0.3955	0.55461	no
Xpa	34.7	29.9549	-0.212144	0.2057	0.343451	no
Xpc	8.64632	12.6881	0.55332	0.00035	0.0014053	yes
Xpnpep1	30.95	28.4569	-0.121158	0.408	0.566879	no
Xpnpep3	3.58181	3.38652	-0.0808878	0.671	0.795147	no
Xpo1	41.499	45.185	0.122765	0.3757	0.534436	no
Xpo4	7.31937	7.30604	-0.00262841	0.98755	0.99274	no
Xpo5	16.6529	17.3052	0.0554363	0.70785	0.821559	no
Xpo6	48.8473	45.7554	-0.094336	0.50565	0.659615	no
Xpo7	8.6601	7.93175	-0.126744	0.37175	0.530303	no
Xpot	12.9937	13.5304	0.0583875	0.6886	0.808013	no
Xpr1	6.95974	6.48253	-0.102476	0.50125	0.655438	no
Xrcc1	19.0193	19.8892	0.0645226	0.6753	0.798219	no
Xrcc2	1.71149	1.44454	-0.244644	0.326	0.481244	no
Xrcc3	3.39706	2.87395	-0.241253	0.61955	0.755552	no
Xrcc4	12.1278	10.8648	-0.158664	0.3832	0.542264	no
Xrcc5	10.9684	12.8544	0.228897	0.1587	0.279789	no
Xrcc6	16.3001	17.3729	0.0919608	0.55465	0.701973	no
Xrcc6bp1	4.08064	4.91726	0.269061	0.31035	0.463885	no
Xrn1	18.6115	19.5732	0.072687	0.6169	0.753518	no
Xrn2	150.628	151.795	0.0111341	0.934	0.964119	no
Xxylt1	5.18057	5.4829	0.0818274	0.8798	0.932627	no
Xylt1	4.03561	6.74748	0.741561	0.00015	0.000653255	yes
Xylt2	13.4283	15.4525	0.202566	0.18195	0.312049	no
Yae1d1	2.68456	2.84564	0.0840667	0.67415	0.7973	no
Yaf2	11.1865	11.566	0.0481323	0.7724	0.86555	no
Yars	17.8686	16.6679	-0.100356	0.5088	0.66239	no
Yars2	9.57639	10.6597	0.154618	0.4073	0.566107	no
Ybey	1.49525	1.46626	-0.0282447	0.96365	0.980388	no
Ybx1	196.358	181.235	-0.115626	0.41815	0.576747	no
Ybx3	109.103	103.177	-0.0805783	0.5687	0.713562	no
Ydjc	5.82318	6.75819	0.214829	0.33255	0.488258	no
Yeats2	11.0973	13.2508	0.255876	0.0788	0.158543	no

Yeats4	50.1498	47.2527	-0.0858485	0.5573	0.704228	no
Yif1a	14.0324	15.2044	0.115729	0.51125	0.664488	no
Yif1b	14.8729	16.1569	0.119468	0.51575	0.668577	no
Yipf1	59.5098	54.9732	-0.114399	0.4434	0.601165	no
Yipf2	11.8116	12.6027	0.0935231	0.7753	0.867546	no
Yipf3	56.6658	54.4359	-0.0579202	0.68145	0.802799	no
Yipf4	32.4589	32.2111	-0.0110551	0.93885	0.966934	no
Yipf5	18.189	17.5916	-0.0481773	0.7557	0.854227	no
Yipf6	4.8202	4.59026	-0.0705166	0.6752	0.79814	no
Ykt6	60.6436	60.4469	-0.00468796	0.9726	0.984908	no
Ylpm1	12.738	15.1008	0.245484	0.08625	0.171041	no
Yme1l1	36.5641	35.6802	-0.0353029	0.79825	0.882593	no
Yod1	4.03635	3.97786	-0.0210585	0.9366	0.965617	no
Ypel1	3.52103	3.07759	-0.194196	0.7615	0.858131	no
Ypel3	162.789	149.205	-0.125707	0.3767	0.535557	no
Ypel5	26.9791	24.7272	-0.125739	0.38675	0.545875	no
Yrdc	27.2618	29.4237	0.110099	0.4842	0.639637	no
Ythdc1	46.6934	46.5964	-0.00299922	0.98385	0.990629	no
Ythdc2	5.3473	5.38568	0.0103175	0.9466	0.971104	no
Ythdf1	32.3632	31.5883	-0.0349629	0.8058	0.887382	no
Ythdf2	14.9799	14.3629	-0.0606788	0.6866	0.806564	no
Ythdf3	20.4943	20.6446	0.0105411	0.9435	0.969403	no
Ywhab	136.587	123.809	-0.14171	0.3191	0.473614	no
Ywhae	185.449	165.747	-0.162048	0.2542	0.398798	no
Ywhag	29.5223	31.96	0.114462	0.42735	0.585705	no
Ywhah	202.658	180.71	-0.165367	0.2493	0.394744	no
Ywhaq	242.447	216.261	-0.164896	0.25125	0.396819	no
Ywhaz	222.737	208.705	-0.0938736	0.5182	0.670607	no
Yy1	54.1169	52.4903	-0.04403	0.75255	0.852072	no
Yy2	4.87112	4.92857	0.0169155	0.9501	0.973139	no
Zadh2	13.8652	13.0072	-0.0921609	0.54715	0.695574	no
Zap70	168.856	149.498	-0.175664	0.2319	0.375533	no
Zbed3	6.22991	8.03864	0.36774	0.05565	0.119114	no
Zbed4	11.1485	11.7234	0.0725448	0.62515	0.759913	no
Zbed6	18.2213	19.0557	0.0645963	0.82405	0.898799	no
Zbp1	73.1292	75.4548	0.0451651	0.791	0.877988	no
Zbtb1	22.2583	21.5705	-0.0452883	0.75095	0.851189	no
Zbtb11	13.044	13.0066	-0.0041455	0.9782	0.987801	no
Zbtb12	1.29498	1.25564	-0.0445093	0.8821	0.934027	no
Zbtb14	11.6008	10.8671	-0.0942486	0.54525	0.693846	no
Zbtb17	14.859	14.8718	0.00124182	0.997	0.99782	no
Zbtb18	1.73675	1.7931	0.0460614	0.836	0.906023	no
Zbtb2	18.1389	15.4518	-0.231309	0.12465	0.231114	no
Zbtb21	4.75011	4.74241	-0.00233867	0.9878	0.992906	no
Zbtb22	14.098	15.5296	0.139533	0.3687	0.527295	no
Zbtb24	15.9941	14.541	-0.137412	0.37805	0.537043	no
Zbtb25	25.5339	25.7969	0.0147871	0.926	0.959693	no
Zbtb26	1.22585	1.33268	0.120541	0.59255	0.733692	no
Zbtb32	28.7172	31.4636	0.131766	0.3837	0.542743	no
Zbtb33	8.39928	8.76221	0.0610278	0.6969	0.813896	no
Zbtb34	2.06044	2.06213	0.00118412	0.9948	0.996551	no
Zbtb37	3.01043	3.751	0.317304	0.0943	0.184149	no
Zbtb38	7.65156	5.51025	-0.473636	0.0035	0.010941	yes
Zbtb39	2.24982	2.47506	0.137655	0.45685	0.614362	no
Zbtb4	4.34555	4.15385	-0.0650895	0.67695	0.799459	no
Zbtb40	4.45045	4.64309	0.0611339	0.70355	0.818358	no
Zbtb41	4.28869	3.94359	-0.12103	0.439	0.597058	no

Zbtb42	4.17193	3.95078	-0.0785788	0.66555	0.790999	no
Zbtb43	6.05151	5.5806	-0.116876	0.4682	0.624875	no
Zbtb44	6.37447	6.69063	0.0698369	0.63885	0.770726	no
Zbtb45	12.8701	11.4011	-0.174849	0.2965	0.448214	no
Zbtb48	6.98534	7.95465	0.187468	0.30055	0.452968	no
Zbtb49	3.18223	3.6928	0.214679	0.28705	0.437565	no
Zbtb5	3.89634	3.63471	-0.100281	0.57155	0.716005	no
Zbtb6	6.15238	6.35326	0.0463536	0.77595	0.868012	no
Zbtb7a	24.4108	23.1743	-0.0749944	0.5963	0.736732	no
Zbtb7b	18.154	17.6461	-0.0409382	0.78185	0.87189	no
Zbtb8a	5.10941	4.5515	-0.166816	0.3992	0.558235	no
Zbtb8os	84.9047	79.711	-0.0910654	0.55555	0.702645	no
Zbtb9	11.4584	11.4843	0.00324787	0.98395	0.990673	no
Zc3h10	14.866	12.5141	-0.248455	0.13145	0.241206	no
Zc3h11a	65.4801	64.2877	-0.0265119	0.8728	0.928582	no
Zc3h12a	14.9692	18.4373	0.300627	0.05105	0.110677	no
Zc3h12d	11.8851	15.284	0.362871	0.0153	0.0395135	yes
Zc3h13	15.8881	16.982	0.0960571	0.50025	0.654458	no
Zc3h14	31.9338	31.7729	-0.00728784	0.9655	0.981181	no
Zc3h15	75.5826	68.7167	-0.137395	0.33745	0.493676	no
Zc3h18	33.0914	34.1259	0.0444079	0.7567	0.854865	no
Zc3h3	12.2891	11.6703	-0.0745428	0.62775	0.761888	no
Zc3h4	34.9982	34.1259	-0.0364147	0.7997	0.88349	no
Zc3h6	1.6922	1.45634	-0.216555	0.3153	0.469089	no
Zc3h7a	58.0737	57.0453	-0.0257774	0.8517	0.915587	no
Zc3h7b	8.96616	9.54282	0.0899253	0.551	0.698907	no
Zc3h8	4.12462	4.44856	0.109079	0.64665	0.776737	no
Zc3hav1	156.693	162.526	0.0527296	0.7129	0.824965	no
Zc3hav1l	0.995866	1.02639	0.0435486	0.84515	0.91164	no
Zc3hc1	12.4809	13.1779	0.0784009	0.64125	0.772512	no
Zc4h2	1.27997	1.40281	0.132206	0.65105	0.78019	no
Zcchc10	12.4051	11.2296	-0.143617	0.4381	0.596098	no
Zcchc11	29.8684	27.7674	-0.105224	0.45725	0.614736	no
Zcchc17	37.1058	36.371	-0.0288551	0.85235	0.916048	no
Zcchc18	12.575	9.01121	-0.480761	0.02575	0.0620811	no
Zcchc2	13.8952	16.4856	0.246618	0.0881	0.174162	no
Zcchc3	1.53364	1.59895	0.0601589	0.81235	0.891521	no
Zcchc4	4.99949	5.16795	0.0478101	0.81625	0.893828	no
Zcchc6	34.2992	34.9413	0.026757	0.8488	0.913762	no
Zcchc7	29.1966	32.5553	0.157092	0.28815	0.438796	no
Zcchc8	20.4727	20.3717	-0.00713454	0.96195	0.979661	no
Zcchc9	18.6243	17.2451	-0.111003	0.4992	0.653411	no
Zcrb1	53.1427	50.6814	-0.0684126	0.66555	0.790999	no
Zcwpw1	2.23479	2.02499	-0.142224	0.5986	0.738656	no
Zdhhc1	1.15438	0.987603	-0.225112	0.50385	0.657948	no
Zdhhc12	12.4345	14.3059	0.202261	0.2908	0.441793	no
Zdhhc13	10.7078	10.8262	0.0158645	0.9243	0.958705	no
Zdhhc15	8.88633	6.8993	-0.365139	0.01735	0.0440075	yes
Zdhhc16	13.0971	13.113	0.0017574	0.9965	0.997475	no
Zdhhc17	8.95565	9.12897	0.0276531	0.8591	0.920178	no
Zdhhc18	104.465	105.205	0.0101872	0.9431	0.96919	no
Zdhhc2	15.8774	13.5435	-0.229373	0.14565	0.260874	no
Zdhhc20	50.9962	44.433	-0.19876	0.16125	0.283351	no
Zdhhc21	8.14543	7.62189	-0.0958424	0.50715	0.660867	no
Zdhhc24	3.22463	2.74292	-0.233422	0.29885	0.450936	no
Zdhhc3	15.7814	16.5038	0.0645748	0.65265	0.781497	no
Zdhhc4	31.9457	30.0469	-0.088406	0.57315	0.717398	no

Zdhhc5	22.0413	22.0263	-0.000981299	0.99505	0.996674	no
Zdhhc6	21.5101	20.9026	-0.0413316	0.78775	0.875923	no
Zdhhc7	31.824	30.5947	-0.0568364	0.6993	0.815556	no
Zdhhc8	11.8038	12.5277	0.085872	0.56665	0.71185	no
Zdhhc9	7.62667	5.85617	-0.381094	0.02995	0.0704362	no
Zeb1	5.442	7.47788	0.458492	0.0031	0.00983478	yes
Zeb2	28.8002	10.8786	-1.40459	5.00E-05	0.000236281	yes
Zer1	22.029	21.759	-0.0177946	0.89985	0.944589	no
Zfand1	15.2197	15.3285	0.0102756	0.95245	0.974584	no
Zfand2a	12.9786	12.4835	-0.0561215	0.7168	0.827592	no
Zfand2b	28.6477	29.8191	0.0578197	0.7167	0.827517	no
Zfand3	26.648	24.224	-0.137586	0.35755	0.515401	no
Zfand4	3.17111	2.54317	-0.318357	0.1289	0.23741	no
Zfand5	10.7184	9.41347	-0.187287	0.19825	0.333919	no
Zfand6	80.3267	80.3005	-0.000471333	0.99745	0.998044	no
Zfat	1.99399	1.65045	-0.272802	0.2006	0.336848	no
Zfc3h1	19.5237	18.189	-0.102156	0.4625	0.619599	no
Zfhx2	7.87695	8.29314	0.074282	0.63275	0.766038	no
Zfml	21.3219	23.0833	0.11451	0.43015	0.588446	no
Zfp1	6.05817	6.09217	0.0080742	0.96745	0.982271	no
Zfp101	2.40104	2.10802	-0.187768	0.42725	0.585636	no
Zfp106	19.6401	20.416	0.0559003	0.69915	0.815511	no
Zfp109	0.731594	1.08864	0.573416	0.07755	0.156542	no
Zfp11	1.10307	0.821577	-0.425063	0.1171	0.219865	no
Zfp110	18.8893	19.4169	0.0397426	0.7864	0.874973	no
Zfp113	2.6123	3.03641	0.217044	0.214	0.35411	no
Zfp119a	1.10287	1.10065	-0.00291236	0.97745	0.987453	no
Zfp12	8.23597	8.6245	0.0665027	0.6647	0.790268	no
Zfp120	7.64232	6.26512	-0.28667	0.0805	0.161448	no
Zfp128	0.773376	1.05898	0.45343	0.1089	0.207297	no
Zfp142	6.80313	6.74699	-0.0119526	0.96435	0.980619	no
Zfp143	13.8673	12.8834	-0.106176	0.49465	0.649346	no
Zfp146	16.8741	18.2952	0.116652	0.46775	0.624463	no
Zfp148	12.6877	12.2842	-0.0466228	0.7448	0.847102	no
Zfp157	2.87907	2.76731	-0.0571204	0.74865	0.849687	no
Zfp160	5.29542	5.02547	-0.0754883	0.66135	0.788077	no
Zfp169	5.14571	5.71644	0.151748	0.3703	0.528887	no
Zfp180	15.5601	13.2057	-0.236694	0.1094	0.208098	no
Zfp182	10.6038	9.74119	-0.122407	0.40075	0.559737	no
Zfp189	1.86624	1.61875	-0.205256	0.40325	0.562227	no
Zfp191	13.421	13.5002	0.008487	0.955	0.975976	no
Zfp207	90.9101	88.3199	-0.0417035	0.7733	0.866119	no
Zfp212	12.6651	12.5644	-0.0115151	0.94195	0.968581	no
Zfp213	7.00246	6.3123	-0.149697	0.4042	0.563151	no
Zfp217	18.3568	22.172	0.272429	0.05745	0.12235	no
Zfp219	3.25469	3.47632	0.0950442	0.64975	0.779129	no
Zfp235	2.26736	2.1784	-0.0577441	0.79095	0.877987	no
Zfp236	12.5279	13.444	0.101824	0.47835	0.634367	no
Zfp239	0.999057	1.06574	0.0932193	0.76055	0.857622	no
Zfp251	11.9383	11.3432	-0.0737664	0.6257	0.760333	no
Zfp26	4.04988	4.4975	0.151244	0.31825	0.472591	no
Zfp260	20.6682	18.751	-0.140447	0.33195	0.487621	no
Zfp263	19.3936	20.5463	0.0832997	0.5721	0.716414	no
Zfp266	7.93512	8.53718	0.105507	0.4848	0.640142	no
Zfp27	2.30409	1.93206	-0.254062	0.24405	0.389032	no
Zfp273	2.31856	2.20909	-0.0697715	0.77335	0.866147	no
Zfp275	6.85335	7.00706	0.0320005	0.83255	0.903961	no

Zfp276	10.8128	11.1841	0.0487129	0.7777	0.86913	no
Zfp277	19.7547	18.3064	-0.10985	0.4825	0.63812	no
Zfp28	0.950914	1.00022	0.0729248	0.7837	0.873167	no
Zfp280b	6.34373	6.99194	0.14036	0.3757	0.534436	no
Zfp280c	9.42373	10.1568	0.108079	0.49115	0.645965	no
Zfp280d	9.2306	9.99761	0.115158	0.453	0.61058	no
Zfp281	7.68583	7.70571	0.00372715	0.97985	0.98859	no
Zfp282	4.53047	5.62966	0.313388	0.05755	0.122537	no
Zfp287	3.43302	3.02957	-0.180369	0.32585	0.481113	no
Zfp292	12.9644	14.7525	0.186406	0.1954	0.330088	no
Zfp296	1.78256	2.67176	0.583845	0.049	0.106913	no
Zfp3	7.57127	8.0995	0.097298	0.60265	0.741838	no
Zfp317	16.4557	15.9761	-0.0426686	0.7759	0.86797	no
Zfp318	3.8419	4.49004	0.22491	0.2482	0.39354	no
Zfp319	8.77484	8.2547	-0.0881579	0.57925	0.722599	no
Zfp322a	4.90138	4.52862	-0.114118	0.49715	0.651622	no
Zfp324	1.15832	1.07966	-0.101453	0.68255	0.803591	no
Zfp326	8.54698	9.73421	0.187649	0.2715	0.419551	no
Zfp329	2.90443	2.89131	-0.00653462	0.96995	0.983692	no
Zfp330	36.4356	34.2974	-0.0872468	0.56285	0.708754	no
Zfp335	24.3375	26.4119	0.118006	0.4114	0.570301	no
Zfp341	3.5678	3.57329	0.00222066	0.9916	0.994884	no
Zfp346	11.4603	11.2207	-0.0304819	0.842	0.909746	no
Zfp35	9.2641	9.46607	0.0311143	0.8471	0.912671	no
Zfp358	4.04403	3.67321	-0.138751	0.53645	0.686658	no
Zfp36	303.77	352.018	0.212672	0.136	0.247707	no
Zfp362	16.4132	19.1826	0.224944	0.1381	0.250474	no
Zfp367	7.55721	5.67978	-0.412018	0.01785	0.0451298	yes
Zfp369	5.99227	7.25322	0.275518	0.1178	0.22095	no
Zfp36l1	71.0675	75.6198	0.0895738	0.5254	0.676934	no
Zfp36l2	417.351	357.68	-0.222592	0.14425	0.258886	no
Zfp382	5.77438	4.21353	-0.454639	0.02505	0.06064	no
Zfp383	1.43441	1.34526	-0.092577	0.73265	0.83858	no
Zfp384	38.8573	38.1863	-0.0251288	0.86125	0.92164	no
Zfp385c	4.7862	4.14649	-0.206988	0.31815	0.472482	no
Zfp386	16.2272	20.0877	0.307894	0.0341	0.0786166	no
Zfp39	4.51927	4.58479	0.0207666	0.903	0.946347	no
Zfp395	5.23805	7.44563	0.507365	0.00245	0.0079866	yes
Zfp397	5.56326	5.62539	0.0160216	0.9205	0.956718	no
Zfp398	4.89236	5.48214	0.164211	0.3581	0.515972	no
Zfp40	3.49741	3.02003	-0.211727	0.2592	0.404807	no
Zfp407	3.90036	3.84098	-0.0221334	0.89205	0.939573	no
Zfp408	7.64747	7.52988	-0.0223547	0.89755	0.943199	no
Zfp41	2.35307	1.98696	-0.243977	0.2551	0.399846	no
Zfp410	16.0138	16.4651	0.0400964	0.7953	0.88078	no
Zfp414	27.4207	26.0375	-0.0746738	0.6462	0.776382	no
Zfp420	2.11809	2.33388	0.13997	0.53305	0.683496	no
Zfp422	10.2722	11.8185	0.202311	0.1997	0.335752	no
Zfp426	13.3218	13.231	-0.00987029	0.94815	0.972115	no
Zfp429	11.077	10.1695	-0.123315	0.47455	0.630963	no
Zfp438	1.07189	1.08343	0.0154515	0.9486	0.972294	no
Zfp444	5.42554	5.49477	0.018292	0.91125	0.951265	no
Zfp445	23.8665	26.0057	0.123838	0.38025	0.539235	no
Zfp446	1.19317	1.08217	-0.140867	0.54915	0.697325	no
Zfp451	12.4089	14.1263	0.187013	0.25195	0.396819	no
Zfp456	4.30008	3.72005	-0.209042	0.2475	0.392686	no
Zfp459	1.40588	1.87904	0.418527	0.08565	0.170033	no

Zfp46	4.12129	4.08367	-0.0132298	0.93935	0.967152	no
Zfp472	11.2853	10.9236	-0.0470002	0.77585	0.867942	no
Zfp488	23.0885	22.3526	-0.0467352	0.74645	0.848241	no
Zfp507	7.83758	9.18448	0.22879	0.12455	0.230977	no
Zfp51	14.4564	13.8508	-0.0617366	0.698	0.814708	no
Zfp511	37.7142	35.323	-0.0945006	0.67065	0.794905	no
Zfp512	10.7355	8.78307	-0.289598	0.07065	0.144922	no
Zfp513	13.1804	12.0215	-0.132786	0.909	0.949801	no
Zfp518a	9.1891	9.24163	0.00822453	0.95745	0.977108	no
Zfp52	14.6356	14.4892	-0.0145084	0.9237	0.958407	no
Zfp523	2.7052	3.7031	0.452999	0.03335	0.0771723	no
Zfp524	25.214	21.3034	-0.243139	0.15115	0.268876	no
Zfp526	4.80457	5.171	0.106036	0.5543	0.701693	no
Zfp53	17.6499	16.6782	-0.0816979	0.58865	0.730516	no
Zfp54	4.32869	3.33197	-0.377553	0.0662	0.13725	no
Zfp553	7.49144	8.1415	0.120053	0.49235	0.647061	no
Zfp560	1.71422	1.35451	-0.339784	0.12445	0.230816	no
Zfp566	3.34722	4.37423	0.386068	0.10455	0.200401	no
Zfp568	4.02216	4.73182	0.234425	0.1422	0.256157	no
Zfp574	8.55201	7.84634	-0.124244	0.43725	0.595217	no
Zfp579	6.15772	6.80766	0.144763	0.43905	0.597091	no
Zfp58	3.38936	3.82524	0.174539	0.41515	0.573921	no
Zfp580	2.89601	3.58375	0.307405	0.29455	0.44604	no
Zfp59	2.09732	2.53835	0.275348	0.19355	0.327558	no
Zfp592	19.3116	20.1697	0.0627212	0.66355	0.789572	no
Zfp593	6.08821	7.37432	0.276492	0.1883	0.320727	no
Zfp595	3.37446	3.5668	0.0799711	0.67165	0.79553	no
Zfp597	6.7627	5.7963	-0.222467	0.15745	0.277967	no
Zfp598	30.2545	29.3616	-0.0432227	0.7623	0.85869	no
Zfp60	3.44305	3.49549	0.0218061	0.90955	0.950122	no
Zfp605	3.88648	3.75255	-0.0505934	0.79015	0.87761	no
Zfp606	2.10826	2.45451	0.219384	0.29895	0.451058	no
Zfp607	3.61029	3.9847	0.142356	0.4402	0.598194	no
Zfp609	1.79027	2.21469	0.306923	0.08375	0.166951	no
Zfp61	4.8191	4.95024	0.0387336	0.84095	0.909015	no
Zfp617	7.32756	6.69919	-0.129348	0.44585	0.603502	no
Zfp62	20.4755	21.011	0.037245	0.79945	0.883352	no
Zfp622	38.7526	37.0093	-0.0664058	0.64005	0.771514	no
Zfp623	2.08643	3.26532	0.646186	0.0068	0.0195677	yes
Zfp628	8.80627	8.82234	0.00263084	0.98985	0.993863	no
Zfp637	10.4555	8.77209	-0.253264	0.224	0.366269	no
Zfp639	19.2595	17.969	-0.100062	0.52445	0.676166	no
Zfp64	9.151	9.85705	0.107226	0.51595	0.668799	no
Zfp644	5.87639	5.59171	-0.0716395	0.6535	0.782157	no
Zfp646	20.7072	20.6797	-0.00191649	0.9905	0.994205	no
Zfp65	4.92011	5.23418	0.0892745	0.61115	0.748567	no
Zfp652	14.6196	18.1225	0.309879	0.04625	0.101841	no
Zfp653	9.85103	10.4007	0.0783348	0.75975	0.857117	no
Zfp654	17.7988	17.7107	-0.00716267	0.9625	0.979895	no
Zfp655	19.783	16.6028	-0.252833	0.12425	0.23053	no
Zfp658	5.13182	5.56856	0.117831	0.5033	0.657436	no
Zfp661	1.5422	1.49612	-0.0437657	0.8634	0.922824	no
Zfp664	18.2289	20.2214	0.149652	0.2995	0.451685	no
Zfp667	1.10041	1.14535	0.0577544	0.823	0.89807	no
Zfp668	6.92201	6.25392	-0.146429	0.38955	0.548708	no
Zfp672	19.9968	18.1848	-0.137038	0.669	0.793616	no
Zfp677	8.6129	8.24455	-0.0630578	0.71205	0.824373	no

Zfp68	12.8345	12.3262	-0.0582989	0.69635	0.813563	no
Zfp687	6.99397	7.34481	0.070613	0.79885	0.883021	no
Zfp688	12.8411	11.9362	-0.105429	0.60115	0.740754	no
Zfp689	1.93818	1.85354	-0.064417	0.7728	0.865765	no
Zfp69	9.9758	2.54915	-1.96842	5.00E-05	0.000236281	yes
Zfp691	3.28029	2.80528	-0.225678	0.3839	0.542982	no
Zfp692	12.441	11.6018	-0.100753	0.55075	0.698678	no
Zfp7	11.2478	10.804	-0.0580697	0.72055	0.830278	no
Zfp703	6.57585	6.16564	-0.0929268	0.59015	0.731747	no
Zfp706	29.5217	28.5609	-0.0477383	0.73945	0.843396	no
Zfp707	11.0796	11.0632	-0.00213684	0.9938	0.9961	no
Zfp708	1.0297	0.870942	-0.24157	0.4798	0.635573	no
Zfp709	2.8032	2.97982	0.0881527	0.6481	0.77783	no
Zfp710	3.07955	3.30597	0.102352	0.5799	0.723179	no
Zfp712	2.58739	3.05625	0.240265	0.2131	0.353018	no
Zfp715	9.31462	10.0775	0.113575	0.46365	0.620692	no
Zfp719	1.58	1.5608	-0.0176436	0.9314	0.962533	no
Zfp738	3.85611	3.76958	-0.032742	0.8537	0.916983	no
Zfp74	3.40225	3.7691	0.147732	0.4334	0.591619	no
Zfp740	34.8834	33.6693	-0.051108	0.7163	0.827339	no
Zfp746	18.4509	20.1916	0.130065	0.37335	0.531994	no
Zfp747	4.7729	4.80148	0.0086136	0.96475	0.9808	no
Zfp748	3.41904	3.40095	-0.00764987	0.96715	0.98215	no
Zfp758	7.99177	8.17483	0.0326741	0.84815	0.913356	no
Zfp759	1.01762	1.0554	0.0525903	0.84655	0.91226	no
Zfp760	1.49262	1.01549	-0.555669	0.0281	0.0667976	no
Zfp763	1.95809	2.02336	0.0473036	0.83635	0.906263	no
Zfp764	3.90754	4.70339	0.267438	0.183	0.313485	no
Zfp770	3.20839	2.96796	-0.112381	0.5449	0.693625	no
Zfp771	4.92205	5.81358	0.240168	0.31825	0.472591	no
Zfp772	1.48211	1.54273	0.057836	0.8274	0.900908	no
Zfp775	2.71845	2.62957	-0.0479589	0.8092	0.889655	no
Zfp777	11.7992	13.5633	0.201018	0.19135	0.324691	no
Zfp780b	2.87307	2.66244	-0.109842	0.54265	0.691797	no
Zfp784	3.53969	3.11441	-0.184662	0.41905	0.577683	no
Zfp787	7.96744	8.79934	0.143279	0.4361	0.594155	no
Zfp788	3.76726	4.28323	0.185183	0.3101	0.46362	no
Zfp790	4.0499	4.47301	0.143359	0.40665	0.56556	no
Zfp799	2.69884	2.85777	0.0825496	0.6679	0.792797	no
Zfp800	13.4558	13.5652	0.0116757	0.9384	0.96671	no
Zfp808	3.75314	3.27	-0.198806	0.3181	0.472438	no
Zfp809	18.0556	15.2468	-0.243939	0.1471	0.262964	no
Zfp81	3.34875	3.67782	0.135229	0.53655	0.686724	no
Zfp810	6.06509	6.11905	0.0127789	0.9406	0.967884	no
Zfp82	5.83742	5.76924	-0.0169485	0.9297	0.961579	no
Zfp821	4.98791	5.67654	0.186576	0.3446	0.501444	no
Zfp825	12.7789	11.952	-0.0965072	0.60265	0.741838	no
Zfp827	2.12393	2.90466	0.45163	0.0107	0.0290897	yes
Zfp830	7.86615	7.57574	-0.05427	0.74945	0.850295	no
Zfp831	6.13957	4.51506	-0.443391	0.0039	0.0120284	yes
Zfp839	6.794	7.82526	0.203878	0.21565	0.35608	no
Zfp84	6.55256	6.1576	-0.0896898	0.56255	0.708503	no
Zfp846	3.40063	3.46347	0.0264159	0.90095	0.945139	no
Zfp85	3.5009	3.31759	-0.0775926	0.7271	0.834692	no
Zfp850	1.9643	2.18473	0.153442	0.4082	0.567079	no
Zfp865	13.9315	15.152	0.121162	0.41895	0.577602	no
Zfp866	4.14485	4.03331	-0.039355	0.8129	0.891792	no

Zfp867	1.31642	1.37277	0.0604728	0.81025	0.890227	no
Zfp868	14.5967	13.3815	-0.125402	0.4306	0.58889	no
Zfp869	17.5458	18.9684	0.112472	0.4588	0.616244	no
Zfp87	8.34374	7.08924	-0.235064	0.17445	0.301561	no
Zfp871	15.3135	15.2474	-0.00624413	0.9662	0.981581	no
Zfp873	2.41138	1.68675	-0.515616	0.04605	0.10148	no
Zfp874a	5.03602	5.00061	-0.010178	0.9546	0.975709	no
Zfp874b	3.54677	3.3746	-0.0717889	0.7021	0.817251	no
Zfp882	1.25198	1.28697	0.0397666	0.85855	0.92005	no
Zfp90	2.48423	2.3676	-0.069376	0.7629	0.859133	no
Zfp91	34.2786	32.6393	-0.0706981	0.62015	0.755957	no
Zfp930	5.63405	5.43104	-0.0529458	0.76965	0.863675	no
Zfp931	0.954726	1.39002	0.541945	0.1229	0.228506	no
Zfp932	15.6283	14.4795	-0.11015	0.4982	0.65255	no
Zfp933	1.96062	1.88586	-0.0560848	0.7923	0.878781	no
Zfp934	1.26042	1.00469	-0.327161	0.2934	0.444747	no
Zfp935	9.24606	7.64818	-0.273724	0.1133	0.213976	no
Zfp938	1.86253	2.25337	0.274819	0.301	0.453424	no
Zfp94	1.17716	1.44317	0.293925	0.3023	0.454858	no
Zfp940	2.25589	2.29128	0.0224569	0.9212	0.957136	no
Zfp942	15.3066	15.8797	0.0530248	0.7309	0.837351	no
Zfp943	9.53715	8.63852	-0.142774	0.3896	0.548735	no
Zfp944	7.91236	8.56252	0.113927	0.4945	0.649197	no
Zfp945	3.2901	3.4162	0.0542612	0.7452	0.847366	no
Zfp946	0.882768	1.3591	0.622547	0.03325	0.0769788	no
Zfp948	3.62576	2.90591	-0.319295	0.1381	0.250474	no
Zfp949	5.83985	6.15065	0.074807	0.66205	0.788579	no
Zfp951	3.49067	2.41489	-0.531546	0.0139	0.0363906	yes
Zfp952	9.17143	7.91364	-0.212805	0.1947	0.329181	no
Zfp953	3.31007	2.92411	-0.178862	0.33065	0.486298	no
Zfp954	15.3925	14.5319	-0.0830023	0.6073	0.745534	no
Zfp955a	5.208	4.84041	-0.1056	0.54345	0.692504	no
Zfp955b	4.61216	4.82739	0.0658003	0.7136	0.825504	no
Zfp956	7.59073	9.24633	0.284642	0.11655	0.219042	no
Zfp958	5.50676	5.72629	0.0563967	0.7652	0.860707	no
Zfp959	6.48157	6.17539	-0.0698131	0.7119	0.824281	no
Zfp961	8.27035	8.28146	0.00193772	0.99305	0.995674	no
Zfp963	2.18858	2.38338	0.123011	0.62505	0.759817	no
Zfp97	1.74152	1.88494	0.114175	0.6475	0.777348	no
Zfpl1	22.5839	20.1265	-0.166201	0.32205	0.476989	no
Zfpm1	22.2242	24.3612	0.132456	0.35925	0.517165	no
Zfr	20.3473	19.5743	-0.0558757	0.69835	0.814928	no
Zfx	11.3817	11.1892	-0.0246039	0.8659	0.924376	no
Zfyve1	11.4826	11.5895	0.0133672	0.92785	0.960632	no
Zfyve16	3.40384	3.34507	-0.0251267	0.88635	0.936364	no
Zfyve19	13.5958	12.4517	-0.126811	0.51075	0.664022	no
Zfyve20	9.69465	9.50826	-0.0280077	0.8551	0.917912	no
Zfyve21	1.6153	1.93676	0.26184	0.44015	0.598149	no
Zfyve26	5.78348	6.46041	0.159689	0.28175	0.431602	no
Zfyve27	11.5234	11.8264	0.0374497	0.79705	0.881874	no
Zgpat	67.8523	72.5083	0.0957492	0.59875	0.738738	no
Zgrf1	1.73136	1.11369	-0.636548	0.0016	0.00547227	yes
Zhx1	13.4756	12.7307	-0.0820346	0.5771	0.720757	no
Zhx2	5.40107	7.29298	0.433262	0.0093	0.0257386	yes
Zhx3	1.35221	1.54894	0.195969	0.88685	0.936612	no
Zkscan1	3.37904	3.93284	0.21896	0.17165	0.297807	no
Zkscan14	8.12268	7.74348	-0.0689737	0.7058	0.820029	no

Zkscan17	11.9116	10.8697	-0.132058	0.41835	0.576932	no
Zkscan3	21.4329	24.0304	0.165036	0.2711	0.419034	no
Zkscan5	2.96927	3.2589	0.134273	0.4833	0.638819	no
Zkscan6	14.0743	14.1639	0.00915912	0.95505	0.975999	no
Zkscan7	2.38018	3.27053	0.458455	0.05375	0.115565	no
Zkscan8	2.02372	2.71095	0.421785	0.06015	0.127164	no
Zmat2	47.2368	44.6381	-0.0816346	0.5686	0.713462	no
Zmat3	3.6406	4.21249	0.210498	0.1865	0.318229	no
Zmat5	54.2427	53.9913	-0.00670155	0.96555	0.981203	no
Zmiz1	40.1242	33.3733	-0.26578	0.0569	0.121351	no
Zmiz2	41.0857	44.9479	0.129617	0.36165	0.519824	no
Zmpste24	19.5287	20.3561	0.0598612	0.68635	0.806378	no
Zmym1	5.08208	4.95797	-0.0356679	0.83405	0.904896	no
Zmym2	12.5226	13.6939	0.128993	0.3722	0.530838	no
Zmym3	9.57132	9.13094	-0.0679559	0.7095	0.822649	no
Zmym4	3.23624	3.25785	0.00960238	0.9764	0.986943	no
Zmym5	13.3046	13.7494	0.047437	0.7454	0.847498	no
Zmym6	7.72622	9.73611	0.333582	0.0319	0.0743238	no
Zmynd11	38.5749	38.8456	0.0100878	0.94425	0.969778	no
Zmynd19	12.3227	11.5991	-0.0873081	0.64495	0.775434	no
Zmynd8	21.7313	22.0892	0.0235725	0.8788	0.932239	no
Znf512b	7.60322	8.45324	0.152894	0.51185	0.664987	no
Znfx1	16.0239	14.9209	-0.102898	0.5757	0.719594	no
Znhit1	14.5897	16.0968	0.141823	0.5566	0.70352	no
Znhit2	19.4813	23.2945	0.257897	0.12675	0.234257	no
Znhit3	12.7374	9.38944	-0.439958	0.12695	0.234553	no
Znhit6	5.68032	6.30809	0.151231	0.39885	0.557878	no
Znrd1	40.339	37.8008	-0.0937577	0.58325	0.72601	no
Znrd1as	2.48212	3.25056	0.389114	0.22815	0.371223	no
Znrf1	14.61	17.0715	0.22464	0.12195	0.227092	no
Znrf2	91.8077	96.9686	0.0789032	0.5773	0.720943	no
Znrf3	5.11607	4.5595	-0.166159	0.28965	0.440539	no
Zpbp2	3.94044	3.07562	-0.35748	0.16735	0.291983	no
Zranb1	13.3762	13.7196	0.0365738	0.82175	0.897384	no
Zranb2	26.4927	22.0019	-0.267964	0.07195	0.14714	no
Zranb3	1.96419	1.53632	-0.354453	0.10565	0.20213	no
Zrsr1	5.20809	5.69011	0.127703	0.57905	0.722438	no
Zrsr2	14.9515	15.7874	0.0784836	0.6098	0.747523	no
Zscan12	1.11573	1.82421	0.70928	0.0037	0.0114853	yes
Zscan2	3.78335	4.1444	0.131501	0.52255	0.674419	no
Zscan20	3.40522	3.29899	-0.045723	0.7951	0.880655	no
Zscan21	7.2027	7.59736	0.0769591	0.663	0.789244	no
Zscan22	3.47396	3.32758	-0.0621097	0.75515	0.853866	no
Zscan25	11.3683	13.0806	0.202415	0.2286	0.37169	no
Zscan26	14.2154	16.3794	0.204429	0.168	0.29272	no
Zscan29	14.7033	14.9211	0.021209	0.8999	0.944627	no
Zswim1	6.6295	7.12956	0.104914	0.5476	0.695882	no
Zswim3	2.50783	2.24102	-0.162282	0.4853	0.640587	no
Zswim4	4.9201	5.65759	0.201499	0.2376	0.382325	no
Zswim6	12.3958	11.5285	-0.104636	0.4733	0.629752	no
Zswim7	4.28325	4.10684	-0.0606765	0.8485	0.913538	no
Zswim8	33.8441	34.1688	0.013773	0.9207	0.956856	no
Zufsp	14.2978	14.0028	-0.030082	0.8496	0.914179	no
Zw10	25.7052	24.4604	-0.0716102	0.6254	0.760099	no
Zwilch	5.81728	3.95178	-0.557843	0.0038	0.0117554	yes
Zwint	15.6226	15.1337	-0.0458631	0.7706	0.864343	no
Zxdb	1.82413	1.76329	-0.0489349	0.8033	0.885887	no

Zxdc	8.86526	9.54229	0.106172	0.48535	0.640641	no
Zyg11b	13.6223	14.9269	0.131948	0.35365	0.511209	no
Zyx	392.687	386.785	-0.0218485	0.88675	0.936576	no
Zzef1	16.7379	16.5973	-0.0121636	0.9304	0.961965	no
Zzz3	11.4213	11.8827	0.057142	0.68735	0.807083	no

Supplemental Table S2. Expressed genes and FPKM values for WT and miR-$150^{-/-}$(KO) effector CD8+ T cells.
